# The effects of a 3-day mountain bike cycling race on the autonomic nervous system (ANS) and heart rate variability in amateur cyclists: a prospective quantitative research design

**DOI:** 10.1186/s13102-022-00614-y

**Published:** 2023-01-02

**Authors:** Anton Swart, Demitri Constantinou

**Affiliations:** 1 Department of Exercise Science and Sports Medicine, Faculty of Health Sciences, University of the Witwatersrand, Impilo Block, 27 St Andrews Road, Parktown, Johannesburg, 2193 South Africa; 2 Utopia Medi-Spa, 64 Nelson Mandeala Ave., Maerua Mall, PO Box. 97765, Windhoek, Namibia

**Keywords:** Autonomic nervous system, Heart rate variability, Amateur athletes

## Abstract

**Background:**

The acute and chronic adaptation of endurance athletes’ hearts shows that increased volume of endurance exercise might cause an acute reduction in cardiac function, causing a physiological cascade that leads to the release of cardiac biomarkers specific to cardiomyocyte stress. Heart rate variability (HRV) is a valuable tool used as a physiological measurement to evaluate the autonomic nervous system (ANS). It is frequently used to assess cardiac autonomic regulation, determining a patient’s risk for unfavorable events. This study set out to determine the changes in the ANS by participating in a 3-day mountain bike cycling race in amateur cyclists using HRV as an outcome measure.

**Methods:**

Sixteen healthy participants (male and female) participating in a 3-day mountain bike cycling race underwent five-minute resting electrocardiography recordings in a supine position 2 days before the race (baseline testing). In addition, HRV measurements were recorded after each race day and 24 h post-race (recovery).

**Results:**

Time-domain and frequency-domain measures showed significant changes from baseline HRV parameters after each race day (p ≤ 0.05). In addition, our data revealed that the mean heart rate and R–R variability intervals did not return to baseline values after 24 h of recovery. Thus, autonomic nervous system (ANS) alterations may be due to changes in cardiac sympatho-vagal balance.

**Conclusions:**

The main strength of this study is using HRV as a measuring and screening tool to assess cardiac autonomic activity, whereby the state of the ANS before and after endurance races can be measured. Thus, physicians, athletes, and coaches can determine the stress of endurance races on the ANS and plan recovery strategies. The reasoning is that if the ANS is in a state of sub-optimal function, susceptible amateur athletes might be at risk for a cardiovascular event or maladaptation due to the endurance race.

**Supplementary Information:**

The online version contains supplementary material available at 10.1186/s13102-022-00614-y.

**What is already known on this topic:**

- Heart rate variability is a suitable measuring and screening tool to assess cardiac autonomic activity and is frequently used to assess cardiac autonomic regulation, determining a patient's risk for unfavourable cardiovascular events.

**What this study adds:**

- Our data show that 24 h is not an adequate period for full recovery of HR and R–R variability intervals after an endurance race. Therefore, it may be useful to evaluate recovery beyond 48 h when assessing recovery after an endurance race.
- Our study showed that HRV decreased significantly after each race day, revealing a decrease in vagal tone resulting in altered ANS function.

**How this study might affect research, practice, or policy:**

- We recommend HRV analysis be used not only to assess recovery or training adaptations/maladaptation but also to be part of a screening tool where sports physicians can assess the state of the ANS before an endurance race.
- We provide a basis for future research in the field of HRV to support this statement showing that cardiac events can be reduced during sporting events when assessing the state of the ANS through HRV measurements, especially when individuals present with an increased risk of cardiac disease.

## Background

The acute and chronic adaptation of endurance athletes’ hearts shows that an increased volume of endurance exercise might cause an acute reduction in cardiac function, causing a physiological cascade that leads to the release of cardiac biomarkers specific to cardiomyocyte stress [1]. This evidence indicates that some endurance athletes may develop a physiological cascade by which cardiomyocyte releases cardiac troponin (cTn) concentrations and that exercise cause changes in pH, temperature, and oxidative and mechanical stress [1]. Athletes and clinicians should be mindful of this and react adequately to this pathophysiological phenomenon [1].

We know that the autonomic nervous system (ANS) regulates the body’s internal functions; the central nervous system (CNS) transmits impulses to peripheral organs, one of which is the cardiovascular system [2]. Furthermore, research has consistently shown that the ANS helps modulate heart rate (HR). With the ANS divided into two central systems, the sympathetic and parasympathetic (vagal) systems transmit automatic signals to the organs. The sympathetic system increases metabolic function to cope with challenges outside the body, while the parasympathetic (vagal) system increases processes associated with growth and repair [3–6].

On the other hand, allostasis represents the adaptive process of maintaining homeostasis through complex physiological changes and achieving a stable body environment [7, 8]. A deteriorated and cumulative allostatic process refers to an allostatic load to which the physiological system cannot adapt [7, 9]. In addition, endurance challenges cause impairment of physiological regulating systems, which influences the sympathetic nervous and immune systems [10].

Often, overreaching is a reaction to this physiological change directly related to several warning signs, including ANS dysfunction and imbalances [4].

Heart rate variability (HRV) is a valuable method used to assess cardiac autonomic regulation, determining an individual’s risk for unfavourable cardiovascular events [4, 11–13]. Potentially, one may determine an athlete's cardiac autonomic risk before an extreme race through an objective baseline of biological variability leading up to the race, along with a history of cardiovascular health.

The evidence from Dong [6] and Hautala et al. [14] suggesting that athletes who present with diminished vagal activity, indicated by a decrease in HRV, with the variability becoming constant, have an increased risk of sudden cardiac death.

Furthermore, it has previously been observed that HRV monitoring is a valuable indicator in diagnosing and preventing overreaching in athletes [15]. HRV analysis may be quantified using time-domain and frequency-domain methods. Time-domain methods, use the time between the R–R intervals of the QRS complex on an electrocardiogram (ECG) recording and determine the variability between the consecutive R waves [4, 11, 12]. Frequency-domain measures consist of various spectral methods to calculate the R–R intervals in series, which evaluates the spread of absolute and relative power into different frequency bands [3, 15–19]. A high HRV occurs in response to exercise training and indicates an improvement in ANS function. Conversely, a lower HRV suggests a lack of or inadequate adaptation to exercise, impairing the ANS [15].

Considering the potential for exercise to cause significant cardiac conduction variations due to stimulation from the ANS, this study set out to determine the effects of participation in a 3-day mountain bike cycling race in amateur cyclists using HRV as an outcome measure. The hypothesis would be that there are significant variations in HRV after each race day, and HRV does not fully recover to baseline values within 24 h.

## Methods

### Study design and population

All participants were amateur mountain bike cyclists with an experience of at least 3 years and a weekly training volume greater than eight hours per week at a moderate intensity greater than 6 METs. Sixteen participants (13 male and three female) took part in a 3-day mountain bike cycling race through a sample of convenience. The Standard Bank Klein-Aus Vista Mountain bike cycling challenge (Namibia) race organizers approved and provided written permission to conduct the study during their race.

All participants were encouraged to avoid drinking alcoholic beverages before and during the study. In addition, no participant reported taking any medication, sympathomimetic drugs, or smoking that could have affected the cardiovascular system.

### Characteristics of the 3-day mountain bike cycling race

The race consisted of a 3-day mountain bike cycling race covering 12 km with an elevation of 398 m on day one, 65 km with an elevation of 1415 m on day two, and 65 km with an elevation of 1258 m on day three. This race is considered highly technical and only advised for highly experienced riders. The race occurred during high temperatures of 37–40 degrees Celsius.

### Data collection

All recordings took place under controlled thermoneutral and calm conditions. Before baseline testing measures, the researcher informed all participants about the testing procedures and what to expect during the testing. Participants were requested to refrain from physical activities two days before the baseline testing to prevent the accumulation of physical fatigue two days before the race; participants’ body composition was measured using the International Society for the Advancement of Kinanthropometry (ISAK) standards [20].

During early morning hours (i.e., from 7:00 to 9:00 am), baseline and 24-h post-race testing procedures were performed. In contrast, daily procedures were performed directly after each race, depending on the individual athlete’s finishing time. Participants presented to a designated area once crossing the finishing line after each stage for the post-race measurement procedures. They were escorted to the testing facility two minutes after crossing the finishing line for analysis. Electrode placement followed the standard 12-lead ECG electrode placement method [21]. The day after the participants completed the third and final stage of the race, the researchers recorded the 24-h post-race measurement. Before all ECG recordings in a supine position, participants underwent a five-minute pre-reading stabilization rest period to ensure heart rate stabilization and decrease interference and noise. In addition, the researcher instructed participants to breathe spontaneously during all recordings to avoid any influence on HRV [22–24].

Knowing that single time-point HRV reading may not fully represent a participant's objective baseline of biological variability over time along with a history of cardiovascular health, a single value/one baseline test was used for the logical purpose of the race being hosted in a remote area.

### HRV analysis

The Welch Allyn PC-based stress ECG system obtained resting 12-lead standard ECG tracing. In addition, the HRV module in Cardio Perfect (CP) software (version 1.6.6.1146) analyses short-term (five-minute recording) HRV in a series of heartbeat intervals up to a maximal resting ECG recording of five minutes. The CP system provides valid and reliable measures of HRV as established from previous validity and reliability studies [25–27]. The sampling rate for the recording for the study was set at 600 Hz.

The CP module automatically detected the QRS complexes and the R–R intervals. Before data processing, all ECG tracings were automatically corrected for artefact removal through CP software for ectopic and missed beats, providing normal-to-normal intervals (NN). The CP system’s default setting for beat rejection is set for a difference of 10% between sequential beats. Therefore, the CP system replaced abnormal beats with linearly interpolated NN intervals based on preceding intervals [25].

### Statistical analysis

The descriptive data are expressed and summarized as the means ± SD and were analysed using the statistical package STATISTICA (version 13.2). The Shapiro–Wilk normality test confirmed non-normality of all HRV parameters. In addition, the study used non-parametric tests for all HRV parameters, including Friedman’s two-way analysis of variance and the Wilcoxon signed-rank test, with results expressed as median and interquartile ranges. Statistical significance was set at p ≤ 0.05.

## Result

The descriptive data of the 16 participants (13 male and three female) included age (48.75 ± 7.41 years), weight (78.48 ± 12.18 kg), height (174.3 ± 6.90 cm), BMI (25.71 ± 2.64), body fat (12.9 ± 3.28%), and waist-to-hip ratio (0.86 ± 0.06).

### Time-domain measures

**Fig. 1:**
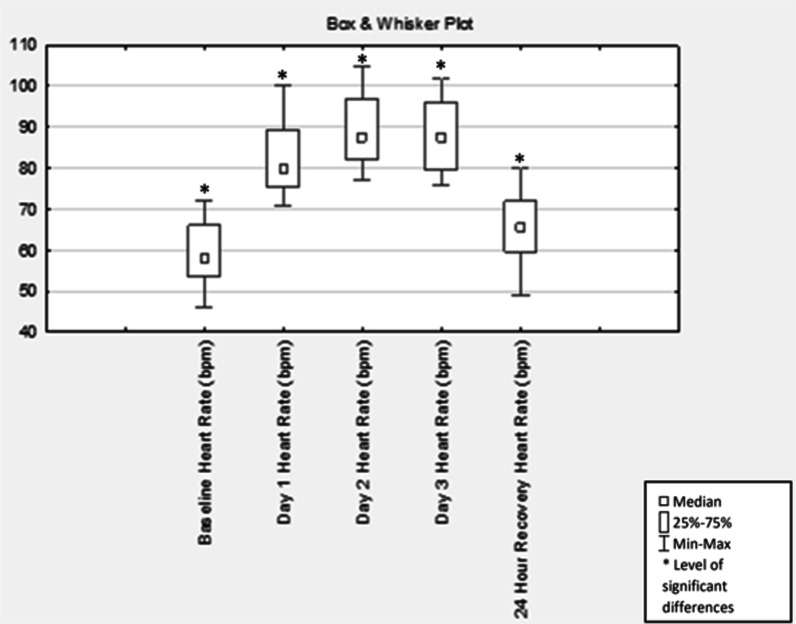
Box & Whisker plot of heart rate (n = 16)

**Fig. 2:**
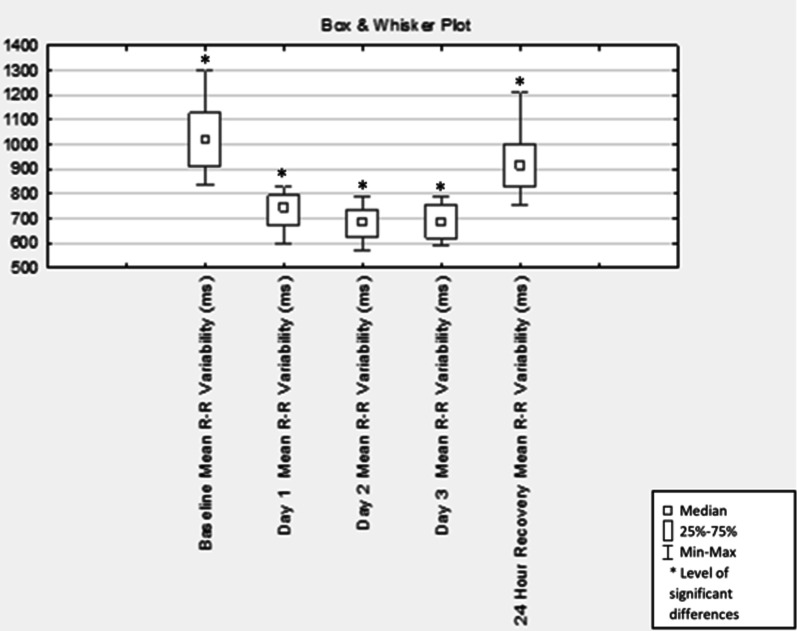
Box & Whisker plot of the mean R–R variability of HRV (n = 16)

Compared with baseline testing, mean HR increased, and HRV decreased significantly (Table 1) and remained increased and decreased, respectively, on day two (p < 0.001) and three (p < 0.001) of the race and did not recover back to baseline measurements at 24 h post-race (p < 0.03). At 24 h post-race, the mean HR and R–R intervals were significantly higher and lower, respectively, than the baseline measurements (p < 0.03) (Table 1, Figs. 1, 2).

**Table 1:** Changes in Time domain measures from baseline to 24-h post-race (n = 16)

	HR (bpm)		RR-intervals (ms)		SDNN (ms)		NN50 (no.)		RMSSD (ms)	
Measurement	Mean ± SD	p value	Mean ± SD	p value	Mean ± SD	p value	Mean ± SD	p value	Mean ± SD	p value
*Time domain measures*										
Baseline	59.50 ± 8.45	–	1031.81 ± 150.00	–	47.63 ± 21.57	–	47.25 ± 50.01	–	43.69 ± 30.16	–
Day 1	82.00 ± 8.66	< 0.001*	738.90 ± 71.83	< 0.001*	23.81 ± 10.18	< 0.001*	5.56 ± 13.79	< 0.01*	38.06 ± 103.32	0.84
Day 2	89.00 ± 9.00	< 0.001*	681.75 ± 69.04	< 0.001*	20.44 ± 11.12	< 0.001*	2.56 ± 3.58	< 0.001*	11.56 ± 5.53	< 0.001*
Day 3	88.06 ± 8.65	< 0.001*	688.88 ± 70.23	< 0.001*	22.06 ± 17.33	< 0.001*	7.69 ± 19.31	< 0.01*	16.50 ± 17.49	< 0.01*
24-Hour post-race	65.38 ± 8.40	0.03*	931.75 ± 125.36	0.03*	43.44 ± 21.69	0.57	27.60 ± 41.07	0.10	34.44 ± 25.18	0.23

*HR* heart rate; *SDNN* standard deviation of the sequence of normal-to-normal intervals; *NN50* number of pairs of successive R–R interval which differ more than 50 ms; *RMSSD* square root of the mean squared differences between successive R–R intervals, *ms* milliseconds, *no.* number

*Level of significant differences between baseline and each race day (p ≤ 0.05)

SDNN and NN50 decreased significantly on day one of the competition compared to baseline (Table 1) and continued to decrease until after day two. After day three, there was a increase compared to day two but significantly lower than baseline values (Table 1). The 24-h recovery revealed no significant difference between baseline and 24-h post-race.

The RMSSD decreased significantly (Table 1) on day two compared to baseline and decreased after day three of the competition. The 24-h post-race RMSSD value revealed no significant difference from the baseline (Table 1).

### Frequency domain measures

Changes in TP, HF, VLF, and LF/HF, indicative of cardiac conduction variations, were associated with a significant (Table 2) change in HRV from day one compared to baseline and continued to remain significantly altered until after day three. There were no statistically significant differences between baseline and 24-h post-race. The LF component decreased significantly (Table 2) from baseline after day one of the competition, but no further changes were observed after days two, and three, and values returned to baseline by 24-h hours post-race.

**Table 2:** Changes in Frequency domain measures from baseline to 24-h post-race (n = 16)

	TP (ms)		HF (ms)		LF (ms)		VLF (ms)		LF/HF (%)	
Measurement	Mean ± SD	p value	Mean ± SD	p value	Mean ± SD	p value	Mean ± SD	p value	Mean ± SD	p value
*Frequency domain measures*										
Baseline	2847.06 ± 3127.77	–	930.56 ± 1133.74	–	1012.44 ± 1271.96	–	904.13 ± 1230.69	–	1.43 ± 0.88	–
Day 1	512.56 ± 499.47	< 0.001*	60.25 ± 67.39	< 0.01*	215.69 ± 180.74	< 0.01*	236.56 ± 268.88	0.02*	6.23 ± 6.73	0.02*
Day 2	465.06 ± 624.08	< 0.01*	35.19 ± 38.23	< 0.01*	299.31 ± 534.66	0.07	130.69 ± 135.55	0.03*	8.24 ± 6.93	< 0.001*
Day 3	562.94 ± 945.72	< 0.01*	137.94 ± 347.65	0.02*	296.13 ± 522.87	0.07	129.00 ± 133.87	0.02*	5.14 ± 4.37	< 0.001*
24-Hour post-race	1807.38 ± 1770.36	0.23	523.88 ± 705.22	0.19	739.75 ± 1175.16	0.50	543.75 ± 445.89	0.30	2.09 ± 1.46	0.11

*TP* total power of overall RR-intervals; *HF* high-frequency power; *LF* low-frequency power; *VLF* very low-frequency power; *LF/HF* ratio between low-frequency power and high-frequency power, *ms* milliseconds

*Level of significant differences between baseline and each race (p ≤ 0.05)

## Discussion

Our findings show that the time-domain and frequency-domain methods measured significant changes at rest immediately after each race day until the final day, showing a decrease in HRV and a decrease in vagal tone resulting in a disturbance in ANS function. Both Dong [6] and Hautala et al. [14] have addressed the implication that may arise during persistent abnormal changes in autonomic regulation. They suggest an increased risk of sudden cardiac death in athletes with diminished vagal activity, indicated by a decrease in HRV [6]. Thus, recovery to baseline is prudently significant. Decrease in R–R variability intervals, SDNN, NN50, and RMSSD, during endurance races indicates a decreased vagal tone [28–31] in line with the findings of our study.

Resting HR after each stage of the race in our study remained higher than the baseline values, which are consistent with data obtained from Brown & Brown [29] Barak et al. [30] Picanço et al. [31]. The increased workload, physiological stress on the body, and sympathetic dominance lead to an increased resting heart rate. Thus, the resultant relative tachycardia reduced TP, which is in agreement with Brown & Brown [29] Barak et al. [30] and Picanço et al. [31] leading to a significant decrease in HRV across the three days of racing.

Low VLF power not only predicts autonomic dysfunction but also indicates an increase in inflammation [19]. This increase in inflammation is often seen due to the sympathetic response and promotes the repair of exercise-induced skeletal muscle damage [19]. Therefore, although there was a decrease in power throughout the study period, and the power started to plateau, one may link this to the increase in inflammation reflected in the VLF power.

The LF component of HRV does not provide an accurate index of the cardiac sympathetic activity but reflects a complex mix of sympathetic and parasympathetic nerve activation along with other unidentified factors. [13] LF variability may even be modulated by parasympathetic influences more substantially than sympathetic activity [13]. While solid evidence exists that there is a strong relationship between HF power and cardiac parasympathetic activity, whereby 90% of HF power contributes to parasympathetic activity and 10% to sympathetic activity [13]. Hence, the LF/HF ratio does not represent cardiac sympatho-vagal balance [13, 32]. Thus, a focus on the HF aspect of the spectral analysis of HRV will be the focus.

An important finding in our study showed a decrease in HF power indicative of decreased vagal activity, which is in accord with the results of others. [29–31] Thus, reflecting an undesirable stimulus due to extreme event conditions and the duration and intensity of the ultra-endurance race. Contrary to the studies of Kaikkonen et al. [33] and Perkins et al. [34] and Earnest et al. [35] we found a significant decrease in HF from after day one, which remained significantly decreased until after day three, and at 24 h of recovery, HF recovered to baseline values. Comparison of the findings with those of other studies by Javorka et al. [36], Brown & Brown [29] Barak et al. [30] Picanço et al. [31].This confirms reduced HRV values compared to baseline values, which is in line with our results.

Therefore the decrease in vagal activity was due to the exercise stimuli. In our study, the duration of exercise stimuli accumulated, and the exercise intensity during the three days varied from low to moderate to high.

## Limitations

Our study has several limitations. First, there was a small number of participants. Second, we only assessed HRV up to 24 h post-race and not beyond. Our Hautala et al. [14], Bernardi et al. [37] and Gratze et al. [38] showed that cardiac parasympathetic activity returned to baseline levels after 24 h of recovery and that complete cardiac autonomic recovery can take up to 48 h of recovery. Thirdly, we confirm that not having an objective baseline of the participant's biological variability before the race over an extended period would have been more beneficial than a single baseline test. The study only used the time-domain and frequency-domain measures. Perhaps nonlinear HRV analysis would have added higher merit to the study outcome. Finally, all HRV datasets (Additional file 1: Individual data of Participants) during the competition were taken immediately after the exercise bout and may have been substantially influenced by the acuteness of the measure, the hydration status of the participants, and the extreme environmental conditions of 37–40 degrees Celcius conditions.

## Conclusion

Our study provides the following information regarding HRV and a 3-day mountain bike cycling race:

- Our data show that 24 h is not an adequate period for the full recovery of the mean HR and R–R interval after an endurance race. Therefore, when assessing recovery after an endurance race, one should be wise to evaluate recovery beyond 24 h.
- Our study showed that the HRV decreased significantly on each race day, revealing a decrease in vagal tone resulting in a disturbance in ANS function.

Thus far, previous studies have reported that changes in autonomic regulation could increase the risk of sudden cardiac death in athletes due to diminished vagal tone and decreased HRV [2, 6, 14]. Thus, assessing heart rate variability is a potentially useful indirect method to determine and assess cardiovascular system control. Therefore, we recommend using HRV as a measuring tool to evaluate cardiac autonomic activity. As a result, sports physicians, athletes, and coaches can assess the stress of endurance events on the ANS and plan for correct recovery strategies after a single endurance race. Furthermore, we recommend that HRV be used not only to assess recovery or training adaptations/maladaptation but also to be part of a screening tool where sports physicians can assess the state of the ANS before an endurance race. The reasoning is that if the ANS is in the form of sub-optimal function, susceptible amateur athletes might be at risk for a cardiovascular event. As a result, we would provide a basis for future research in the field of HRV to support this statement showing that cardiac events can be reduced during sporting events when assessing the state of the ANS through HRV measurements, especially when individuals present with an increased risk of cardiac disease. Thus, having an objective baseline of biological variability over time and a history of cardiovascular health would be more beneficial as an accurate baseline instead of a single value/one baseline test (Additional file 1: Individual data of Participants).

## Supplementary Information

**Additional file 1**. Individual data of Participants.

## Data Availability

All data generated or analysed during this study are included in this published article [and its supplementary information files].

## Abbreviations

cTn: Cardiac troponin
HRV: Heart rate variability
ANS: Autonomic nervous system
ECG: Electrocardiography
CNS: Central nervous system
ISAK: International Society for the Advancement of Kinanthropometry
CP: Cardio perfect
NN: Normal-to-normal interval
SDNN: Standard deviation of the sequence of NN intervals
NN50: Number of pairs of successive RR intervals that differ more than 50 ms
TP: Total power
HF: High-frequency power
LF: Low-frequency power
VLF: Very low-frequency power
