# Supplementary material for: The effects of a 3-day mountain bike cycling race on the autonomic nervous system (ANS) and heart rate variability in amateur cyclists: a prospective quantitative research design
Source: BMC Sports Sci Med Rehabil. 2023 Jan 2;15:2. doi: 10.1186/s13102-022-00614-y (PMC9808932; doi:10.1186/s13102-022-00614-y)
Supplement: Supplementary file 1 — Additional file 1. Individual data of Participants. [file 13102_2022_614_MOESM1_ESM.zip › Individual data of Participants/Body Com. Data/010/010.pdf]

UNIVERSITY OF THE WITWATERSRAND, JOHANNESBURG

METABOLIC REPORT

Research Number of Participant	011
Evaluation Date:	01-May-18

BODY COMPOSITION

Body Weight [kg]	79.8
Body fat [%]	11.6
Ideal body mass [kg]	65.6
Body mass index [m/kg2]	27.9
Waist:Hip ratio	0.92
Bone structure size [S M L]	Large

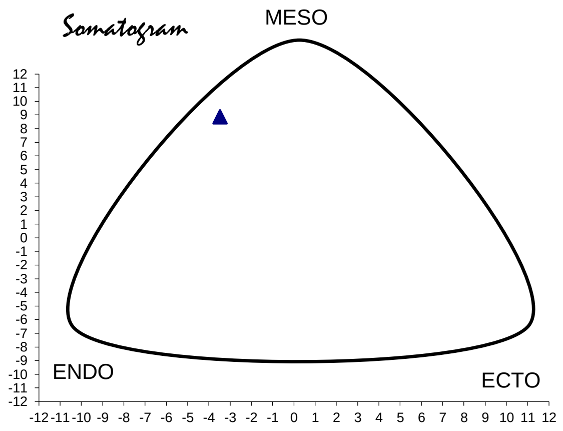

Somatotyping

Endomorph [fat component]	3.8
Mesomorph [muscle compononet]	6.5
Ectomorph [lean component]	0.4

BMI Body mass index is used to estimate a healthy boby weight, based on a persons weight and height. This is also used as a diagnostic tool to identify weight problem. Healthy BMI 18-25 Overweight 26-30 Obese 31-35

Waist-hip-ratio Women with a normal WHR have optimal levels of estrogen and are less susceptible to major diseases such as diabetes, cardiovascular disorders and ovarian cancers. Healthy WHR 0.85-0.90

Fat% Fat% is a better measurement of an individual's fitness level, as it is the only measurement which directly calculates body composition without regard to the individual's height or weight.

	Men	Women
Ideal	5-15%	15-25%
High	15-18%	25-30%
Dangerous	>18 %	>30%
