# Supplementary material for: The effects of a 3-day mountain bike cycling race on the autonomic nervous system (ANS) and heart rate variability in amateur cyclists: a prospective quantitative research design
Source: BMC Sports Sci Med Rehabil. 2023 Jan 2;15:2. doi: 10.1186/s13102-022-00614-y (PMC9808932; doi:10.1186/s13102-022-00614-y)
Supplement: Supplementary file 1 — Additional file 1. Individual data of Participants. [file 13102_2022_614_MOESM1_ESM.zip › Individual data of Participants/HRV Data/001/ECG_001_20180501162140_.PDF]

Anton Swart Biokinetic Rehabilitation Practice

Name:	001 001 001	Recorded:	01/05/2018 16:21:40	UNCONFIRMED INTERPRETATION - MD SHOULD REVIEW
Number:	001	Recorded by:	Mr. Anton Swart	
Gender:	Male	Referring physician:		
Birthdate:	01/06/1967 50 years	Ordering physician:		
		Attending physician:		
P / PQ:	110 ms / 155 ms	Location:	Anton Swart Biokinetic Rehabilitation Practi	
QRS:	82 ms	Comment:		
QT / QTc / QTd:	450 ms / 446 ms / -			
P/QRS/T axis:	62° / 76° / 57°			
Heartrate:	58 bpm			

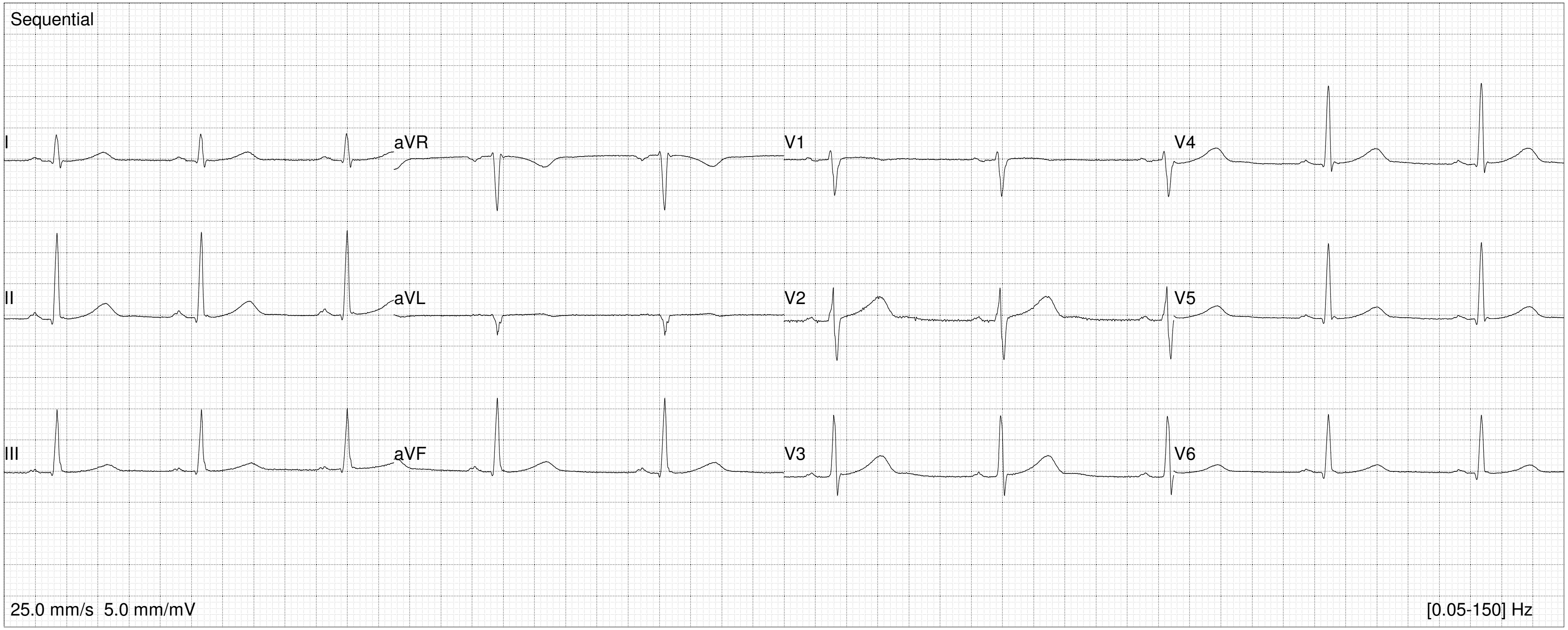

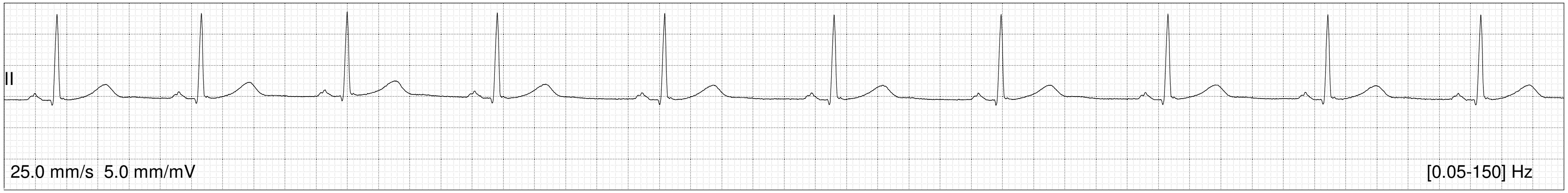

Anton Swart Biokinetic Rehabilitation Practice

Name:	001 001 001	Recorded:	01/05/2018 16:21:40	UNCONFIRMED INTERPRETATION - MD SHOULD REVIEW
Number:	001	Recorded by:	Mr. Anton Swart	
Gender:	Male	Referring physician:		
Birthdate:	01/06/1967 50 years	Location:	Anton Swart Biokinetic Rehabilitation Practice	
		Ordering physician:		
		Attending physician:		
		Comment:		

P / PQ:	110 ms / 155 ms
QRS:	82 ms
QT / QTc / QTd:	450 ms / 446 ms / -
P/QRS/T axis:	62° / 76° / 57°
Heartrate:	58 bpm

**Beats**		**RR**	
Total:	292	Minimum	843 ms
Normal:	292	Maximum	1195 ms
Other:	0	Mean:	1023 ms
		SD:	66 ms

R-R Trend

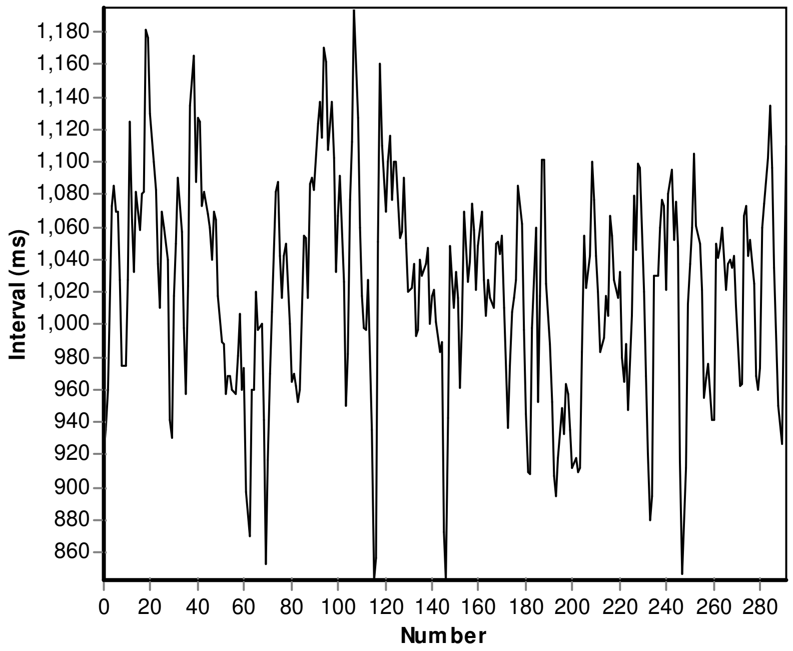

R-R Histogram

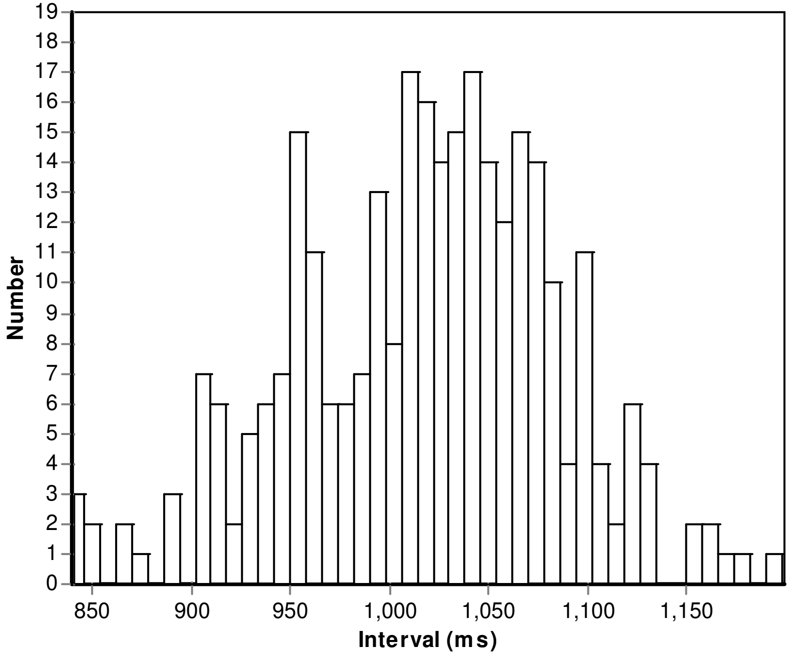

Cardio Control NV. User: Anton

Heart Rate Variability: Time Domain Analysis

**Name:**	001, 001 001	**Birthdate:**	01/06/1967
**Number:**	001	**Recorded:**	01/05/2018 16:21:40
**Gender:**	Male		

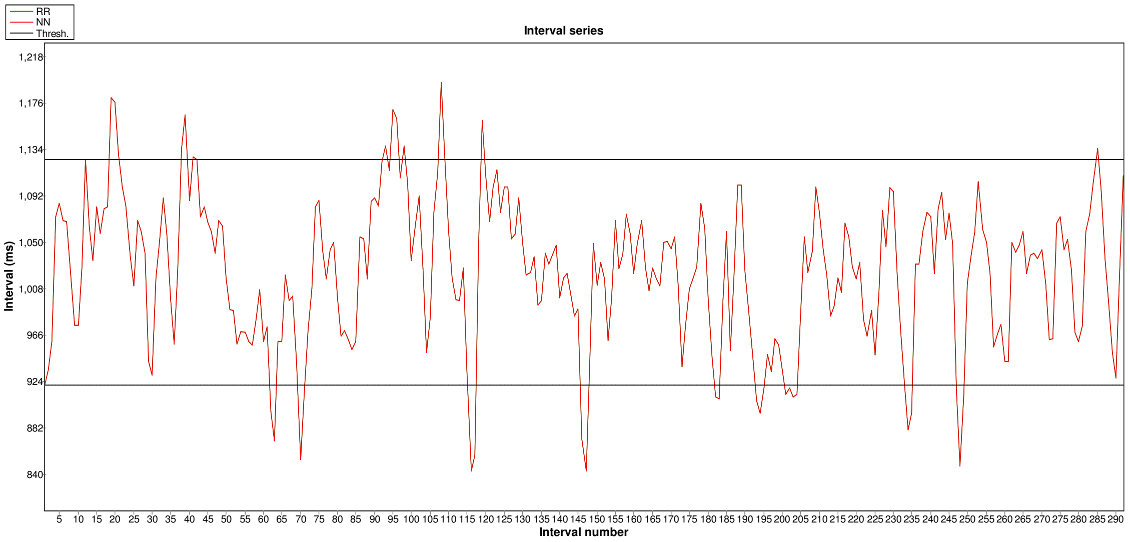

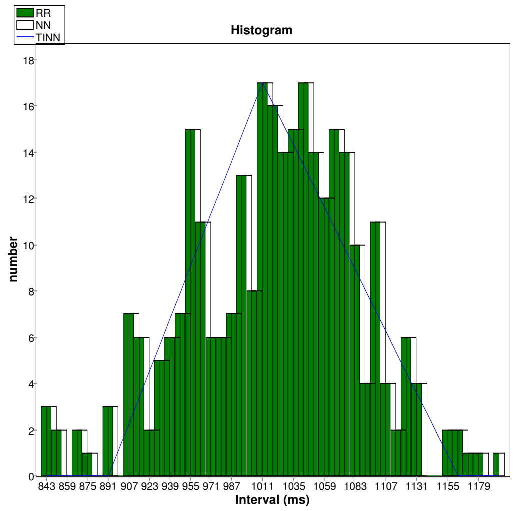

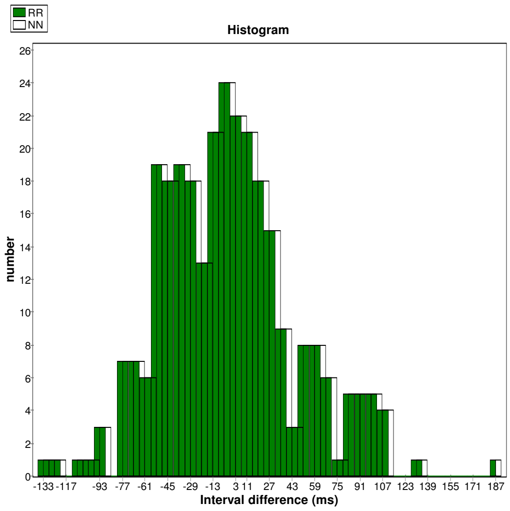

Binsize (ms) = 8

**HRV parameters**	**NN**	**RR**
SDNN (ms)	66	66
Triangular Interpolation (ms)	272	272
Triangular Index	17.18	17.18

**HRV parameters**	**NN**	**RR**
SDSD (ms)	48	48
RMSSD (ms)	48	48
NN50	77	77
NN50(1)	33	33
NN50(2)	44	44
pNN50	0.26	0.26
pNN50(1)	0.11	0.11
pNN50(2)	0.15	0.15
Logarithmic Index	0.22	0.22
SD(Logarithmic Index)	0.02	0.02

**Interval statistics**	**NN**	**RR**
Number	292	292
Minimum (ms)	843	843
Maximum (ms)	1195	1195
Range (ms)	352	352
Avg (ms)	1023	1023
SD (ms)	66	66
AvgDev (ms)	52	52
p5 (ms)	910	910
p50 (ms)	1028	1028
p95 (ms)	1126	1126
Skewness	-0.26	-0.26
Kurtosis	3.00	3.00

**Interval statistics**	**NN**	**RR**
Number	291	291
Minimum (ms)	-133	-133
Maximum (ms)	194	194
Range (ms)	327	327
Avg (ms)	1	1
SD (ms)	48	48
AvgDev (ms)	37	37
p5 (ms)	-69	-69
p50 (ms)	-1	-1
p95 (ms)	95	95
Skewness	0.48	0.48
Kurtosis	3.65	3.65

Heart Rate Variability: Frequency Domain Analysis

**Name:**	001, 001 001	**Birthdate:**	01/06/1967
**Number:**	001	**Recorded:**	01/05/2018 16:21:40
**Gender:**	Male		

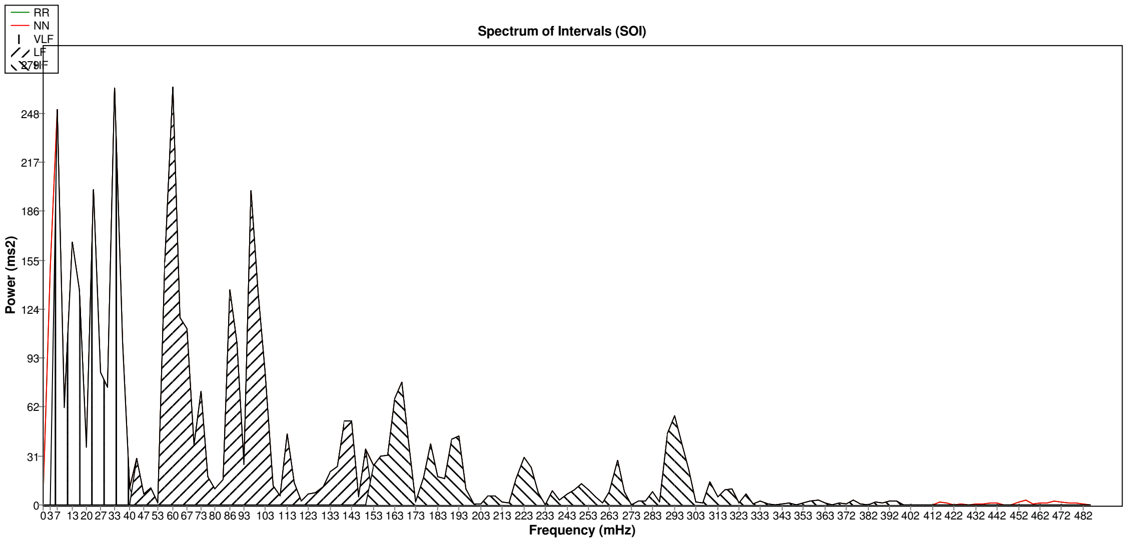

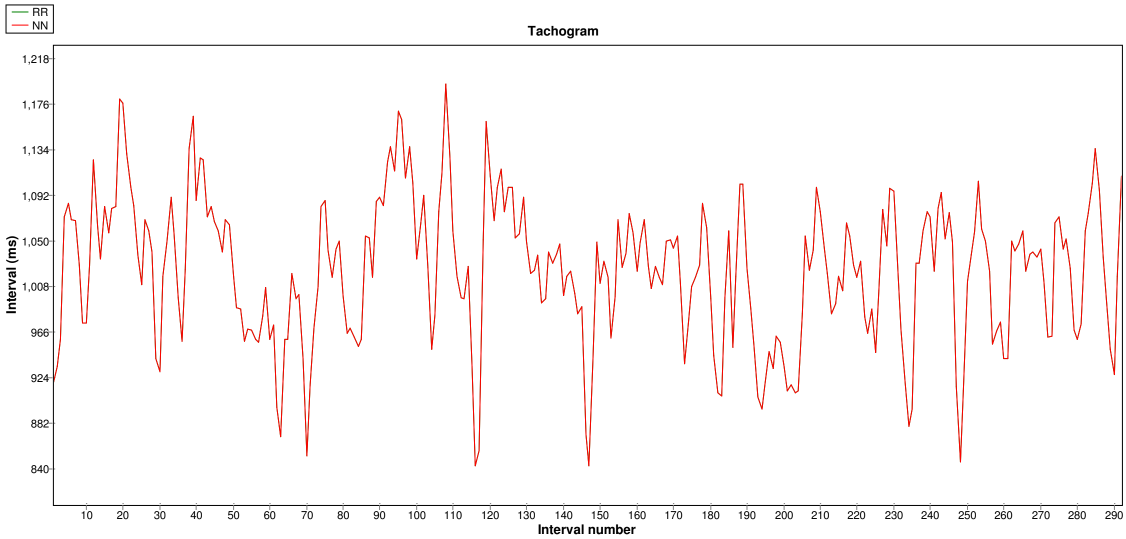

HRV parameters	NN	RR
TP (ms2)	4140	4140
VLF (ms2)	1387	1387
LF (ms2)	1834	1834
HF (ms2)	919	919
LF/HF	2.00	2.00
LF normalized	66.61	66.61
HF normalized	33.39	33.39
VLF peak (mHz)	33	33
LF peak (mHz)	60	60
HF peak (mHz)	166	166

HRV spectral settings	
Spectrum of Intervals (SOI)	
Frequency resolution (mHz)	3
VLF lower boundary (mHz)	3
VLF upper boundary (mHz)	40
LF upper boundary (mHz)	150
HF upper boundary (mHz)	400
Smoothing factor	1
Tapering	Hann
Fourier transform	DFT
Sample frequency (Hz)	0.98
Interval correction	Annotation
Interval threshold (%)	10
