# Supplementary material for: The effects of a 3-day mountain bike cycling race on the autonomic nervous system (ANS) and heart rate variability in amateur cyclists: a prospective quantitative research design
Source: BMC Sports Sci Med Rehabil. 2023 Jan 2;15:2. doi: 10.1186/s13102-022-00614-y (PMC9808932; doi:10.1186/s13102-022-00614-y)
Supplement: Supplementary file 1 — Additional file 1. Individual data of Participants. [file 13102_2022_614_MOESM1_ESM.zip › Individual data of Participants/HRV Data/001/ECG_001_20180503165225_.PDF]

# Anton Swart Biokinetic Rehabilitation Practice

| | | | | |
|---|---|---|---|---|
| Name: | 001 001 001 | Recorded: | 03/05/2018 16:52:25 | UNCONFIRMED INTERPRETATION - MD SHOULD REVIEW |
| Number: | 001 | Recorded by: | Mr. Anton Swart | |
| Gender: | Male | Referring physician: | | |
| Birthdate: | 01/06/1967 50 years | Ordering physician: | | |
| | | Attending physician: | | |
| P / PQ: | 120 ms / 170 ms | Location: | Anton Swart Biokinetic Rehabilitation Practi | |
| QRS: | 82 ms | Comment: | | |
| QT / QTc / QTd: | 363 ms / 433 ms / - | | | |
| P/QRS/T axis: | 73° / 82° / 61° | | | |
| Heartrate: | 100 bpm | | | |

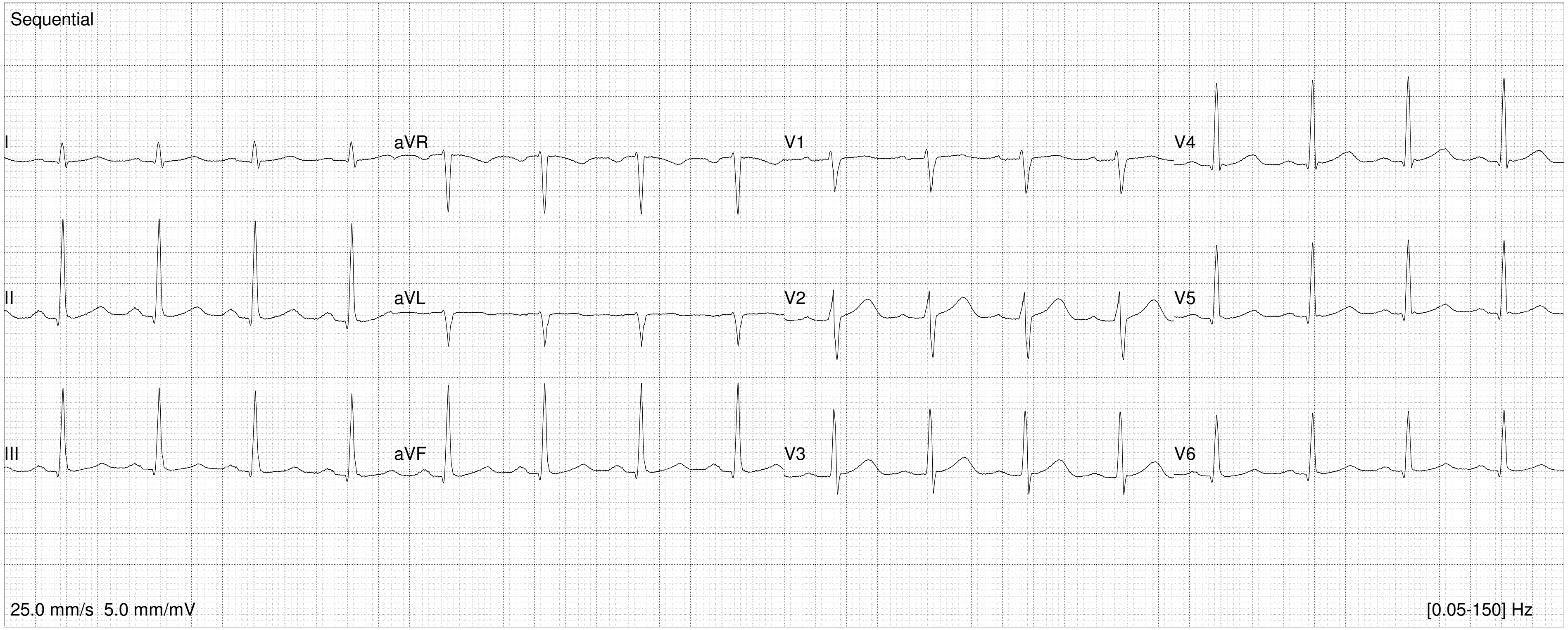


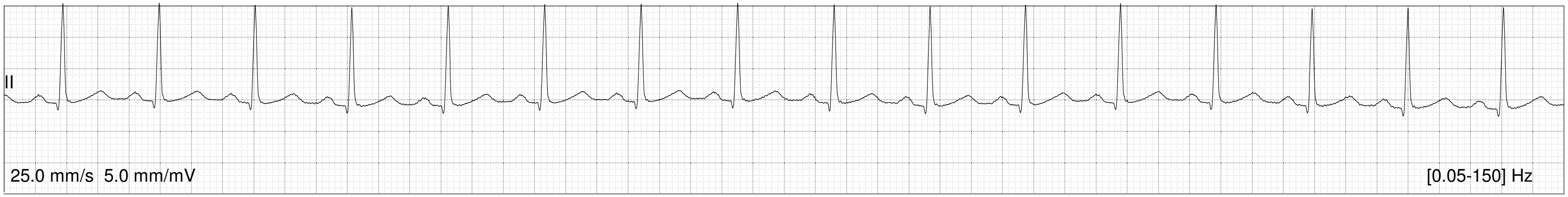

# Anton Swart Biokinetic Rehabilitation Practice

| | | | | |
|---|---|---|---|---|
| Name: | 001 001 001 | Recorded: | 03/05/2018 16:52:25 | UNCONFIRMED INTERPRETATION - MD SHOULD REVIEW |
| Number: | 001 | Recorded by: | Mr. Anton Swart | |
| Gender: | Male | Referring physician: | | |
| Birthdate: | 01/06/1967 50 years | Location: | Anton Swart Biokinetic Rehabilitation Practice | |
| | | Ordering physician: | | |
| | | Attending physician: | | |
| | | Comment: | | |

| | |
|---|---|
| P / PQ: | 120 ms / 170 ms |
| QRS: | 82 ms |
| QT / QTc / QTd: | 363 ms / 433 ms / - |
| P/QRS/T axis: | 73° / 82° / 61° |
| Heartrate: | 100 bpm |

**Beats**

| | |
|---|---|
| Total: | 497 |
| Normal: | 497 |
| Other: | 0 |

**RR**

| | |
|---|---|
| Minimum | 570 ms |
| Maximum | 623 ms |
| Mean: | 602 ms |
| SD: | 7 ms |

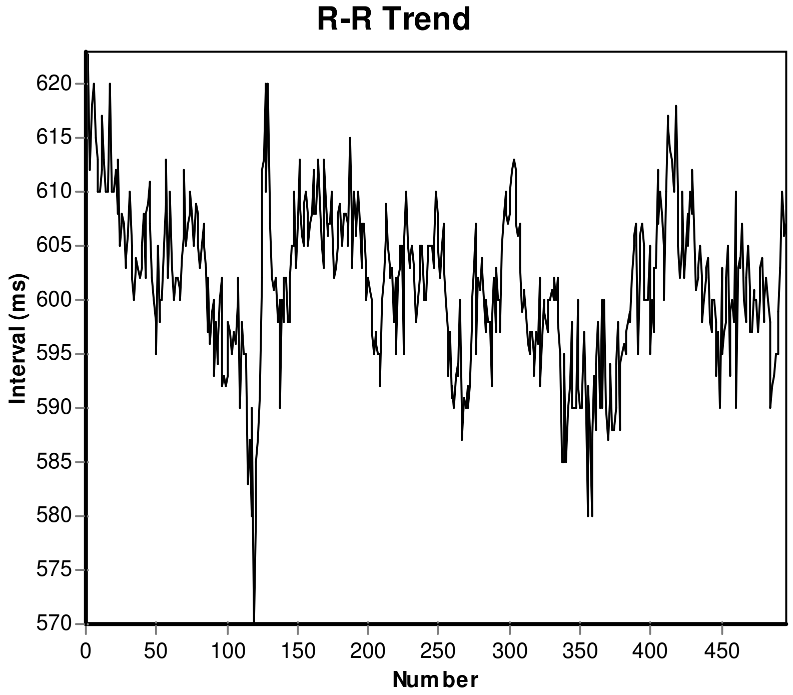


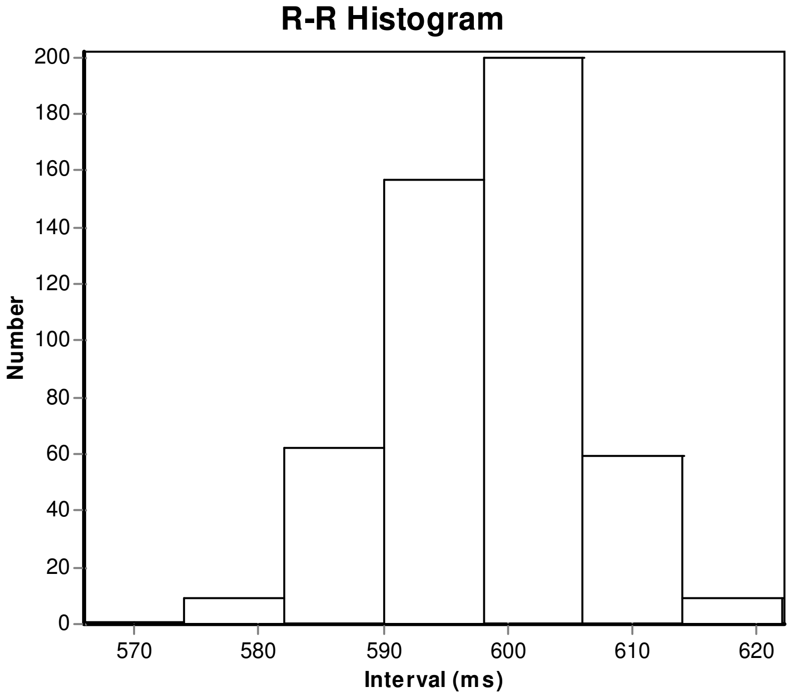

# Cardio Control NV. User: Anton

## Heart Rate Variability: Time Domain Analysis

| | | | |
|---|---|---|---|
| **Name:** | 001, 001 001 | **Birthdate:** | 01/06/1967 |
| **Number:** | 001 | **Recorded:** | 03/05/2018 16:52:25 |
| **Gender:** | Male | | |

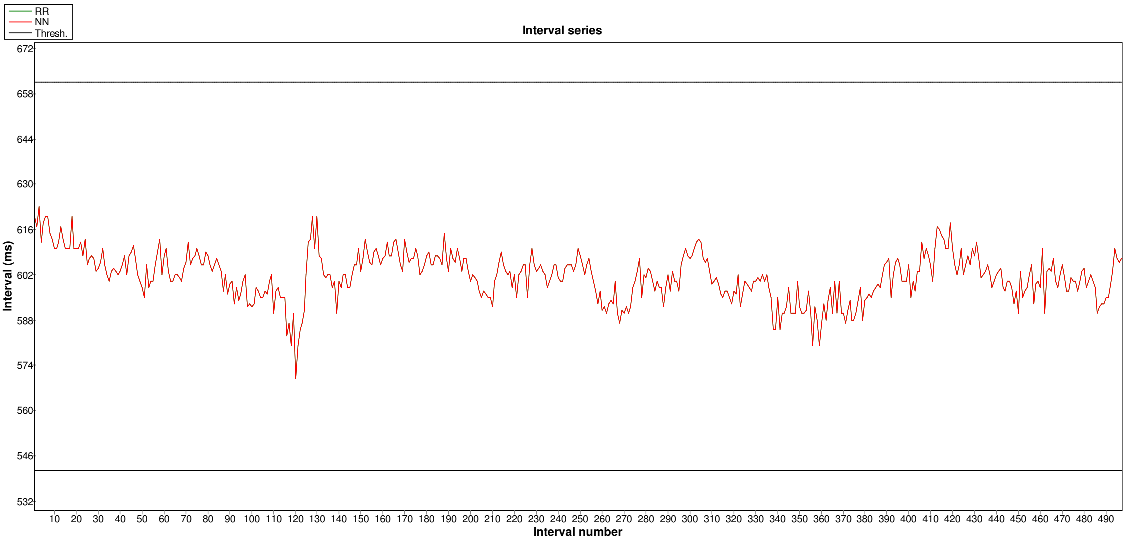


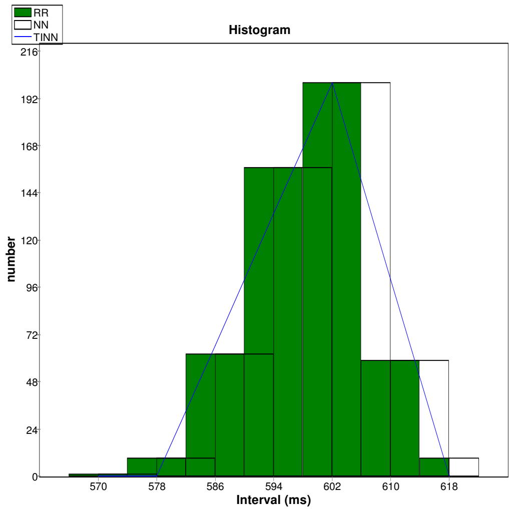


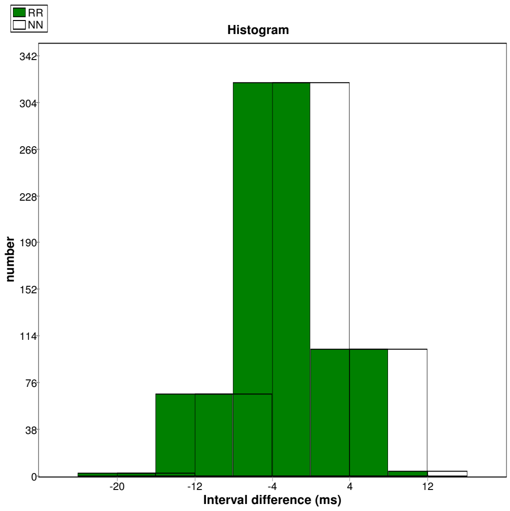


Binsize (ms) = 8

| **HRV parameters** | **NN** | **RR** |
|---|---|---|
| SDNN (ms) | 7 | 7 |
| Triangular Interpolation (ms) | 40 | 40 |
| Triangular Index | 2.48 | 2.48 |

| **HRV parameters** | **NN** | **RR** |
|---|---|---|
| SDSD (ms) | 5 | 5 |
| RMSSD (ms) | 5 | 5 |
| NN50 | 0 | 0 |
| NN50(1) | 0 | 0 |
| NN50(2) | 0 | 0 |
| pNN50 | 0.00 | 0.00 |
| pNN50(1) | 0.00 | 0.00 |
| pNN50(2) | 0.00 | 0.00 |
| Logarithmic Index | 2.69 | 2.69 |
| SD(Logarithmic Index) | 0.37 | 0.37 |

| **Interval statistics** | **NN** | **RR** |
|---|---|---|
| Number | 497 | 497 |
| Minimum (ms) | 570 | 570 |
| Maximum (ms) | 623 | 623 |
| Range (ms) | 53 | 53 |
| Avg (ms) | 602 | 602 |
| SD (ms) | 7 | 7 |
| AvgDev (ms) | 6 | 6 |
| p5 (ms) | 590 | 590 |
| p50 (ms) | 602 | 602 |
| p95 (ms) | 613 | 613 |
| Skewness | -0.27 | -0.27 |
| Kurtosis | 3.64 | 3.64 |

| **Interval statistics** | **NN** | **RR** |
|---|---|---|
| Number | 496 | 496 |
| Minimum (ms) | -20 | -20 |
| Maximum (ms) | 13 | 13 |
| Range (ms) | 33 | 33 |
| Avg (ms) | -0 | -0 |
| SD (ms) | 5 | 5 |
| AvgDev (ms) | 4 | 4 |
| p5 (ms) | -9 | -9 |
| p50 (ms) | 0 | 0 |
| p95 (ms) | 8 | 8 |
| Skewness | -0.35 | -0.35 |
| Kurtosis | 4.12 | 4.12 |

## Heart Rate Variability: Frequency Domain Analysis

| | | | |
|---|---|---|---|
| **Name:** | 001, 001 001 | **Birthdate:** | 01/06/1967 |
| **Number:** | 001 | **Recorded:** | 03/05/2018 16:52:25 |
| **Gender:** | Male | | |

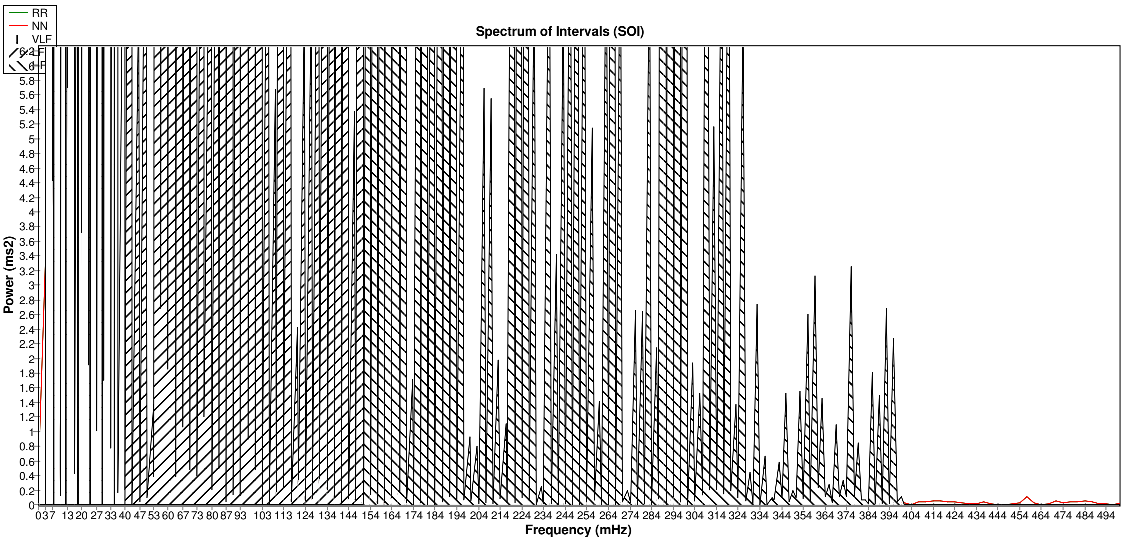


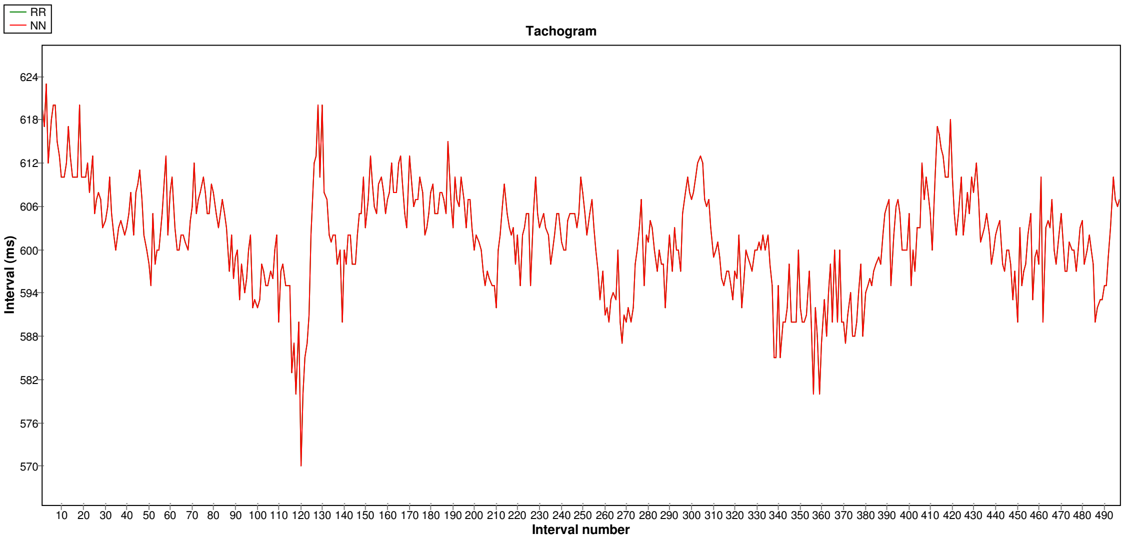


| HRV parameters | NN | RR |
|---|---|---|
| TP (ms2) | 37 | 37 |
| VLF (ms2) | 20 | 20 |
| LF (ms2) | 13 | 13 |
| HF (ms2) | 4 | 4 |
| LF/HF | 3.12 | 3.12 |
| LF normalized | 75.71 | 75.71 |
| HF normalized | 24.29 | 24.29 |
| VLF peak (mHz) | 13 | 13 |
| LF peak (mHz) | 57 | 57 |
| HF peak (mHz) | 361 | 361 |

| HRV spectral settings | |
|---|---|
| Spectrum of Intervals (SOI) | |
| Frequency resolution (mHz) | 3 |
| VLF lower boundary (mHz) | 3 |
| VLF upper boundary (mHz) | 40 |
| LF upper boundary (mHz) | 150 |
| HF upper boundary (mHz) | 400 |
| Smoothing factor | 1 |
| Tapering | Hann |
| Fourier transform | DFT |
| Sample frequency (Hz) | 1.66 |
| Interval correction | Annotation |
| Interval threshold (%) | 10 |
