# Supplementary material for: The effects of a 3-day mountain bike cycling race on the autonomic nervous system (ANS) and heart rate variability in amateur cyclists: a prospective quantitative research design
Source: BMC Sports Sci Med Rehabil. 2023 Jan 2;15:2. doi: 10.1186/s13102-022-00614-y (PMC9808932; doi:10.1186/s13102-022-00614-y)
Supplement: Supplementary file 1 — Additional file 1. Individual data of Participants. [file 13102_2022_614_MOESM1_ESM.zip › Individual data of Participants/HRV Data/001/ECG_001_20180504124507_.PDF]

Anton Swart Biokinetic Rehabilitation Practice

Name:	001 001 001	Recorded:	04/05/2018 12:45:07	UNCONFIRMED INTERPRETATION - MD SHOULD REVIEW
Number:	001	Recorded by:	Mr. Anton Swart	
Gender:	Male	Referring physician:		
Birthdate:	01/06/1967 50 years	Ordering physician:		
		Attending physician:		
P / PQ:	105 ms / 148 ms	Location:	Anton Swart Biokinetic Rehabilitation Practi	
QRS:	79 ms	Comment:		
QT / QTc / QTd:	359 ms / 429 ms / -			
P/QRS/T axis:	70° / 82° / 66°			
Heartrate:	100 bpm			

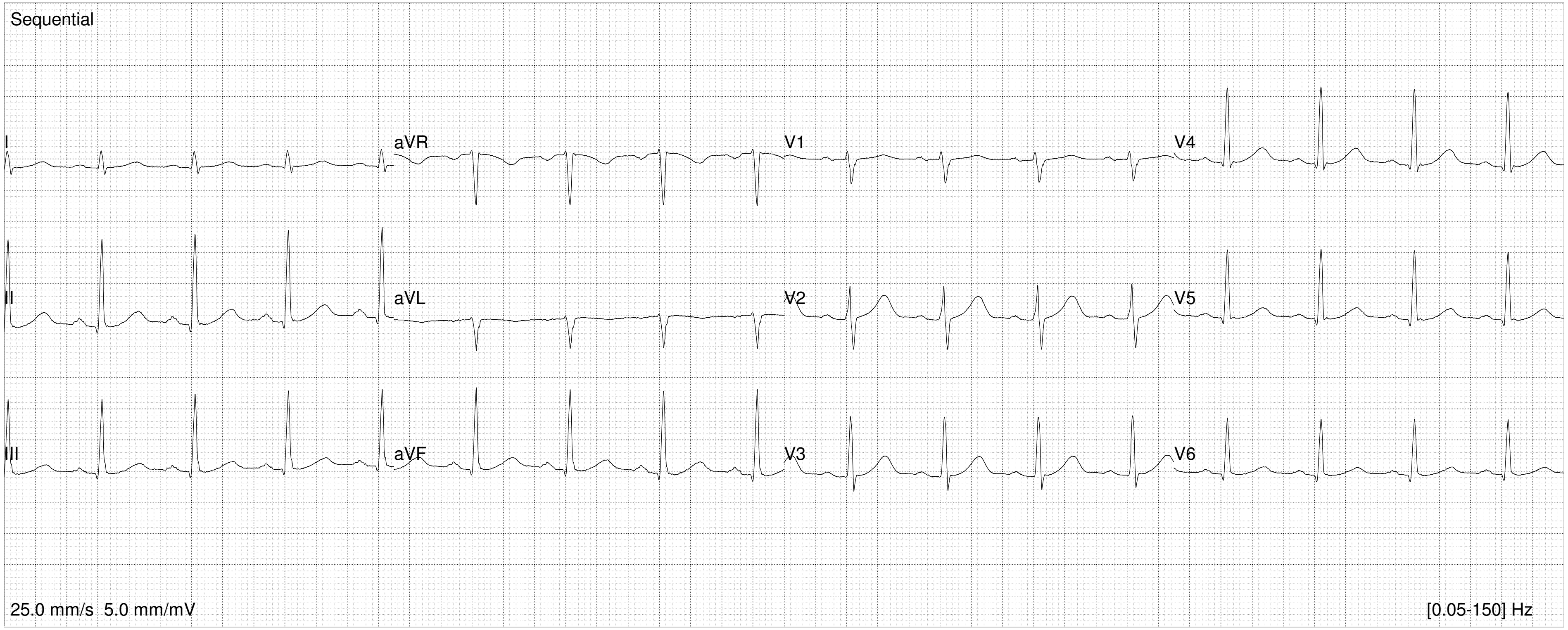

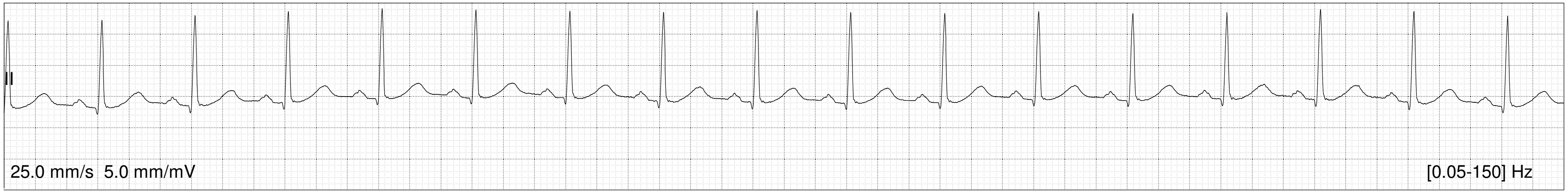

Anton Swart Biokinetic Rehabilitation Practice

Name:	001 001 001	Recorded:	04/05/2018 12:45:07	UNCONFIRMED INTERPRETATION - MD SHOULD REVIEW
Number:	001	Recorded by:	Mr. Anton Swart	
Gender:	Male	Referring physician:		
Birthdate:	01/06/1967 50 years	Location:	Anton Swart Biokinetic Rehabilitation Practice	
		Ordering physician:		
		Attending physician:		
		Comment:		

P / PQ:	105 ms / 148 ms
QRS:	79 ms
QT / QTc / QTd:	359 ms / 429 ms / -
P/QRS/T axis:	70° / 82° / 66°
Heartrate:	100 bpm

Beats

Total:	503
Normal:	503
Other:	0

RR

Minimum	580 ms
Maximum	620 ms
Mean:	595 ms
SD:	7 ms

R-R Trend

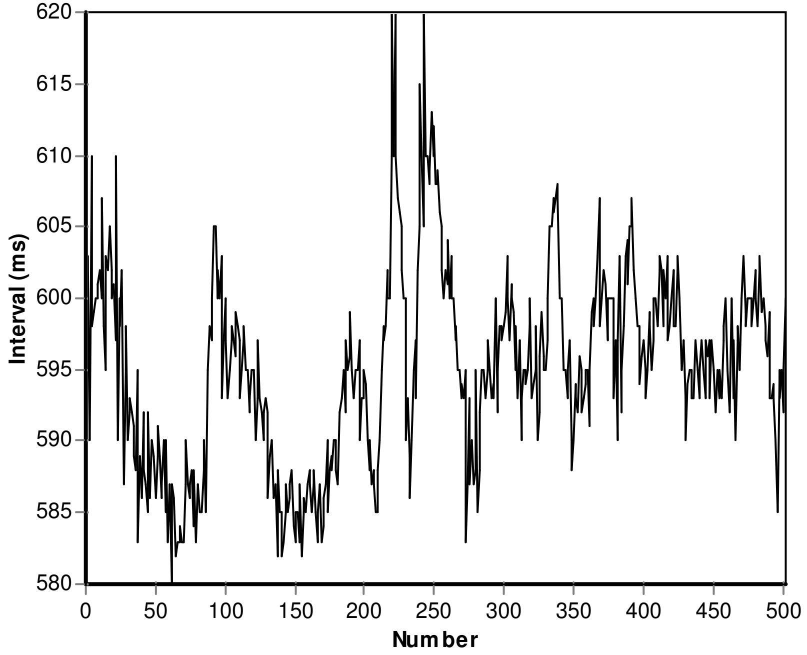

R-R Histogram

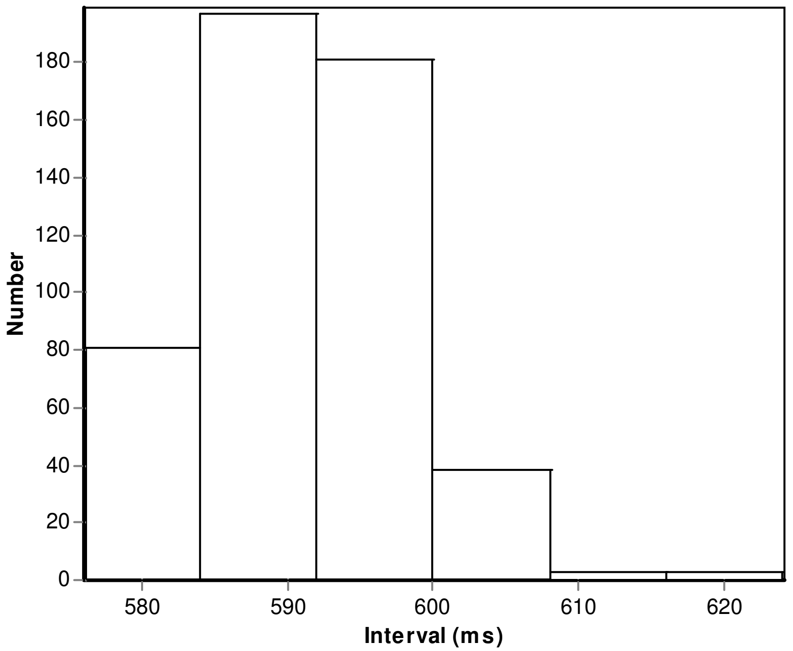

Heart Rate Variability: Time Domain Analysis

**Name:**	001, 001 001	**Birthdate:**	01/06/1967
**Number:**	001	**Recorded:**	04/05/2018 12:45:07
**Gender:**	Male		

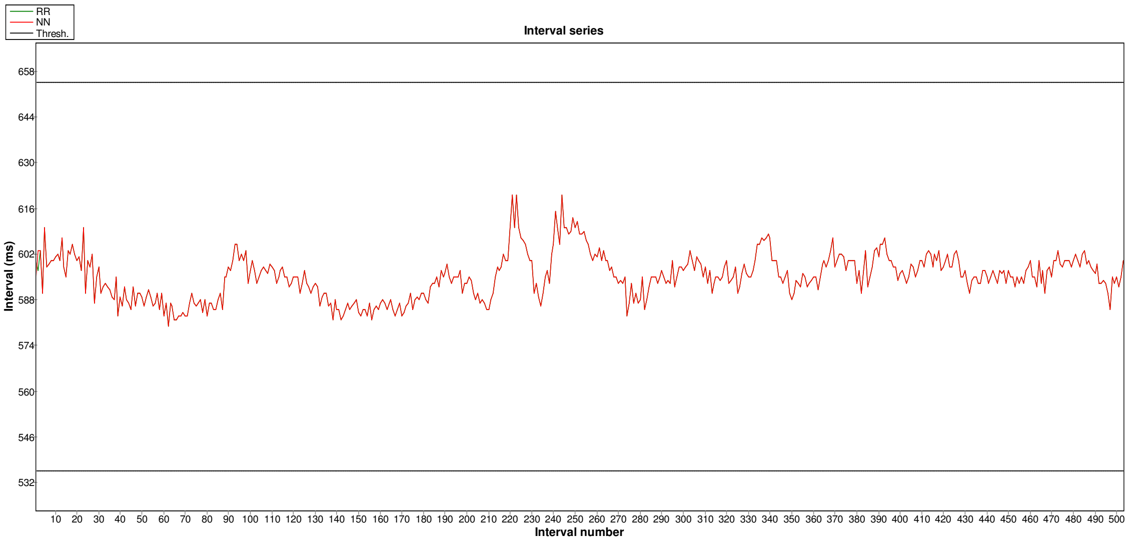

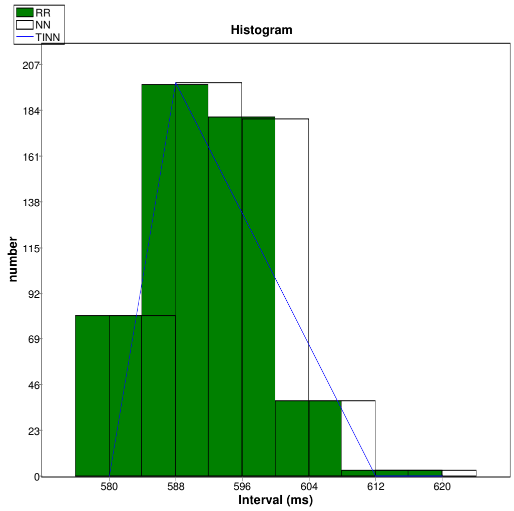

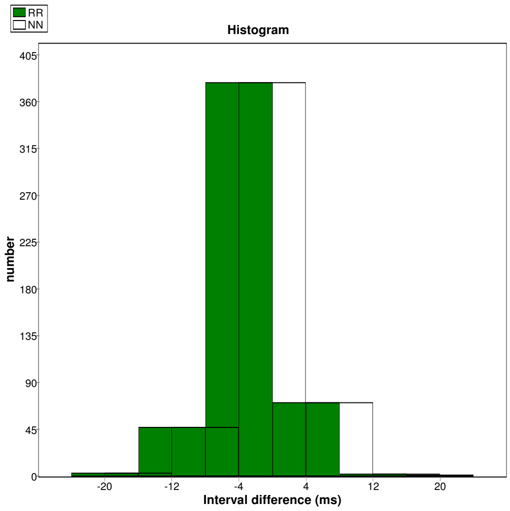

Binsize (ms) = 8

**HRV parameters**	**NN**	**RR**
SDNN (ms)	7	7
Triangular Interpolation (ms)	32	32
Triangular Index	2.54	2.55

**HRV parameters**	**NN**	**RR**
SDSD (ms)	4	4
RMSSD (ms)	4	4
NN50	0	0
NN50(1)	0	0
NN50(2)	0	0
pNN50	0.00	0.00
pNN50(1)	0.00	0.00
pNN50(2)	0.00	0.00
Logarithmic Index	2.73	2.73
SD(Logarithmic Index)	0.01	0.01

**Interval statistics**	**NN**	**RR**
Number	503	503
Minimum (ms)	580	580
Maximum (ms)	620	620
Range (ms)	40	40
Avg (ms)	595	595
SD (ms)	7	7
AvgDev (ms)	5	5
p5 (ms)	585	585
p50 (ms)	595	595
p95 (ms)	607	607
Skewness	0.36	0.36
Kurtosis	3.40	3.41

**Interval statistics**	**NN**	**RR**
Number	502	502
Minimum (ms)	-20	-20
Maximum (ms)	20	20
Range (ms)	40	40
Avg (ms)	0	0
SD (ms)	4	4
AvgDev (ms)	3	3
p5 (ms)	-7	-7
p50 (ms)	0	0
p95 (ms)	6	6
Skewness	-0.20	-0.20
Kurtosis	6.02	6.03

Heart Rate Variability: Frequency Domain Analysis

**Name:**	001, 001 001	**Birthdate:**	01/06/1967
**Number:**	001	**Recorded:**	04/05/2018 12:45:07
**Gender:**	Male		

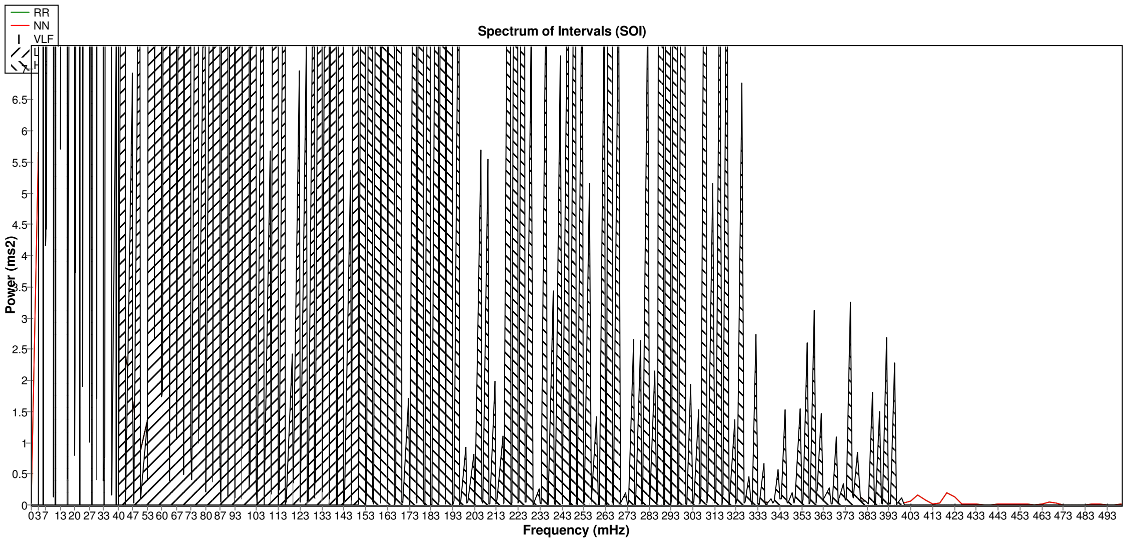

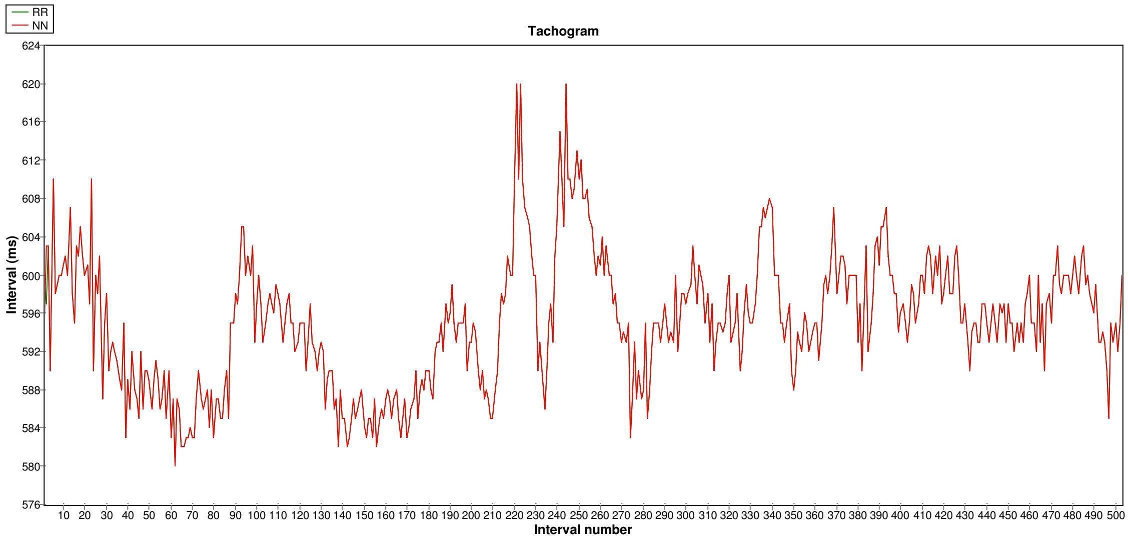

HRV parameters	NN	RR
TP (ms2)	51	51
VLF (ms2)	28	28
LF (ms2)	20	20
HF (ms2)	3	3
LF/HF	7.94	7.94
LF normalized	88.81	88.81
HF normalized	11.19	11.19
VLF peak (mHz)	13	13
LF peak (mHz)	57	57
HF peak (mHz)	377	377

HRV spectral settings	
Spectrum of Intervals (SOI)	
Frequency resolution (mHz)	3
VLF lower boundary (mHz)	3
VLF upper boundary (mHz)	40
LF upper boundary (mHz)	150
HF upper boundary (mHz)	400
Smoothing factor	1
Tapering	Hann
Fourier transform	DFT
Sample frequency (Hz)	1.68
Interval correction	Annotation
Interval threshold (%)	10
