# Supplementary material for: The effects of a 3-day mountain bike cycling race on the autonomic nervous system (ANS) and heart rate variability in amateur cyclists: a prospective quantitative research design
Source: BMC Sports Sci Med Rehabil. 2023 Jan 2;15:2. doi: 10.1186/s13102-022-00614-y (PMC9808932; doi:10.1186/s13102-022-00614-y)
Supplement: Supplementary file 1 — Additional file 1. Individual data of Participants. [file 13102_2022_614_MOESM1_ESM.zip › Individual data of Participants/HRV Data/001/ECG_001_20180505123343_.PDF]

Anton Swart Biokinetic Rehabilitation Practice

Name:	001 001 001	Recorded:	05/05/2018 12:33:43	UNCONFIRMED INTERPRETATION - MD SHOULD REVIEW
Number:	001	Recorded by:	Mr. Anton Swart	
Gender:	Male	Referring physician:		
Birthdate:	01/06/1967 50 years	Ordering physician:		
		Attending physician:		
P / PQ:	103 ms / 142 ms	Location:	Anton Swart Biokinetic Rehabilitation Practi	
QRS:	76 ms	Comment:		
QT / QTc / QTd:	358 ms / 432 ms / -			
P/QRS/T axis:	76° / 83° / 71°			
Heartrate:	102 bpm			

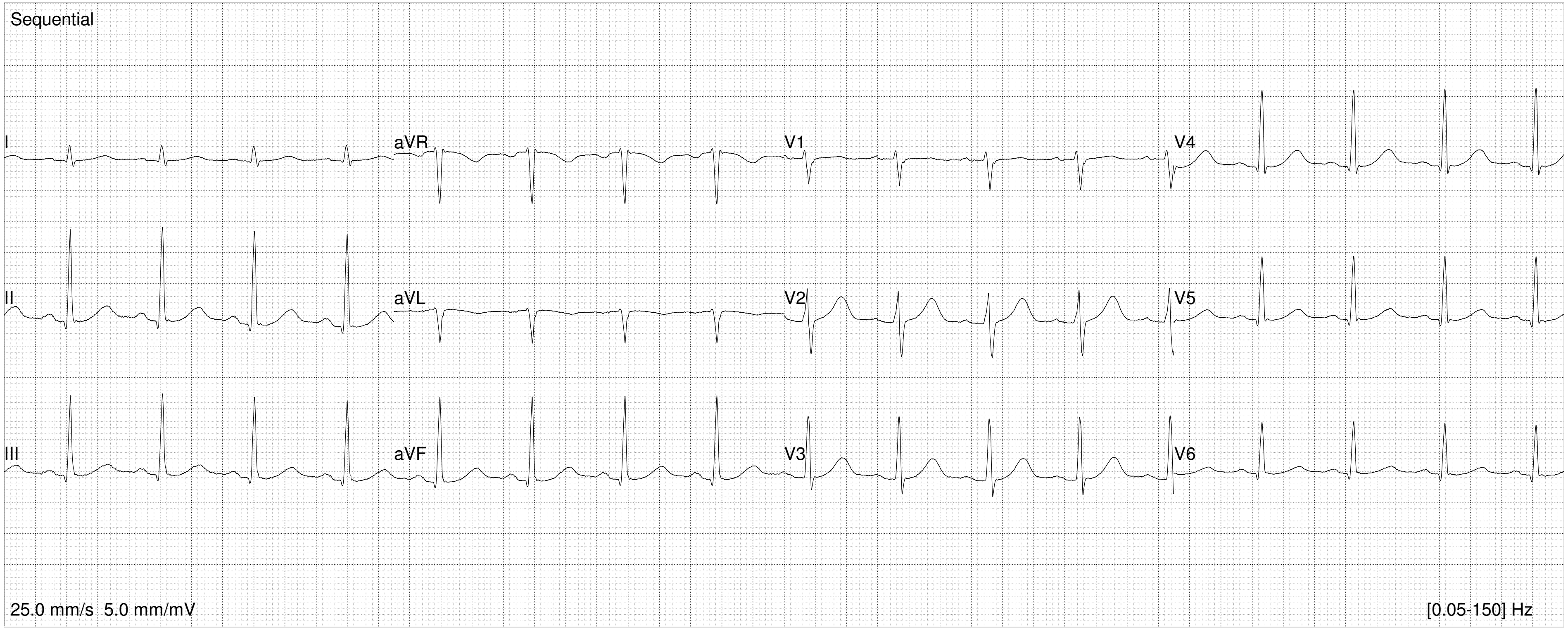

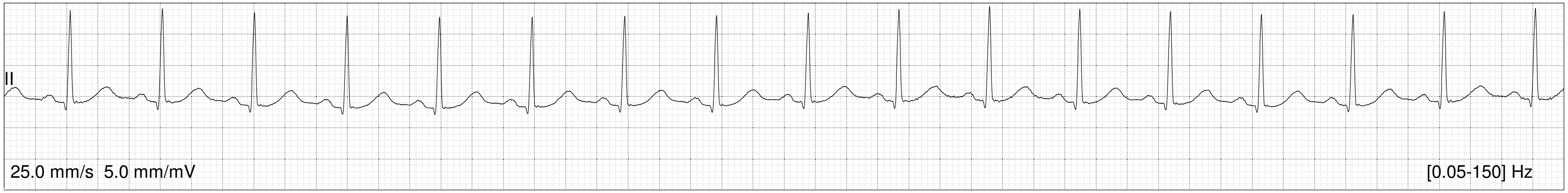

Anton Swart Biokinetic Rehabilitation Practice

Name:	001 001 001	Recorded:	05/05/2018 12:33:43	UNCONFIRMED INTERPRETATION - MD SHOULD REVIEW
Number:	001	Recorded by:	Mr. Anton Swart	
Gender:	Male	Referring physician:		
Birthdate:	01/06/1967 50 years	Location:	Anton Swart Biokinetic Rehabilitation Practice	
P / PQ:	103 ms / 142 ms	Ordering physician:		
QRS:	76 ms	Attending physician:		
QT / QTc / QTd:	358 ms / 432 ms / -	Comment:		
P/QRS/T axis:	76° / 83° / 71°			
Heartrate:	102 bpm			

**Beats**		**RR**	
Total:	508	Minimum	573 ms
Normal:	508	Maximum	612 ms
Other:	0	Mean:	589 ms
		SD:	6 ms

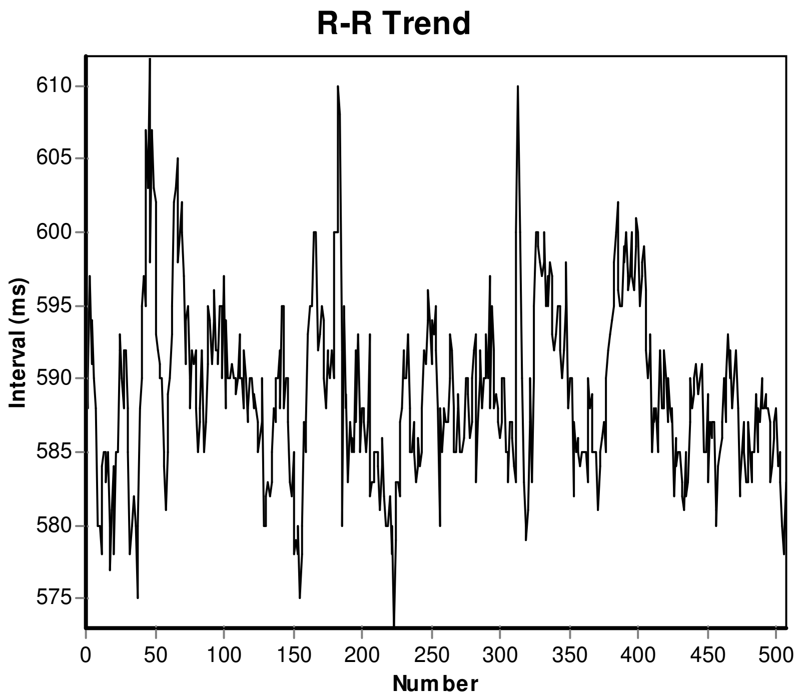

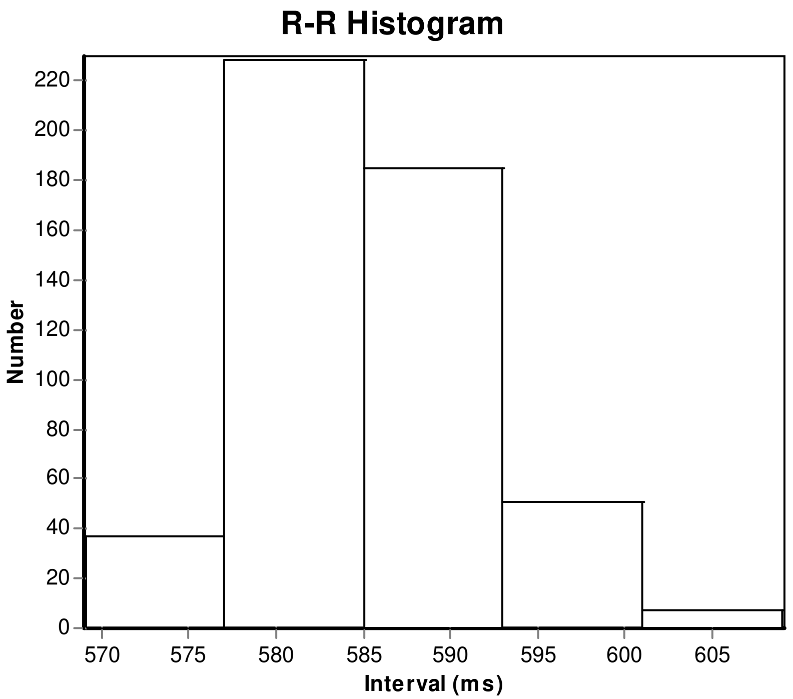

Cardio Control NV. User: Anton

Heart Rate Variability: Time Domain Analysis

**Name:**	001, 001 001	**Birthdate:**	01/06/1967
**Number:**	001	**Recorded:**	05/05/2018 12:33:43
**Gender:**	Male		

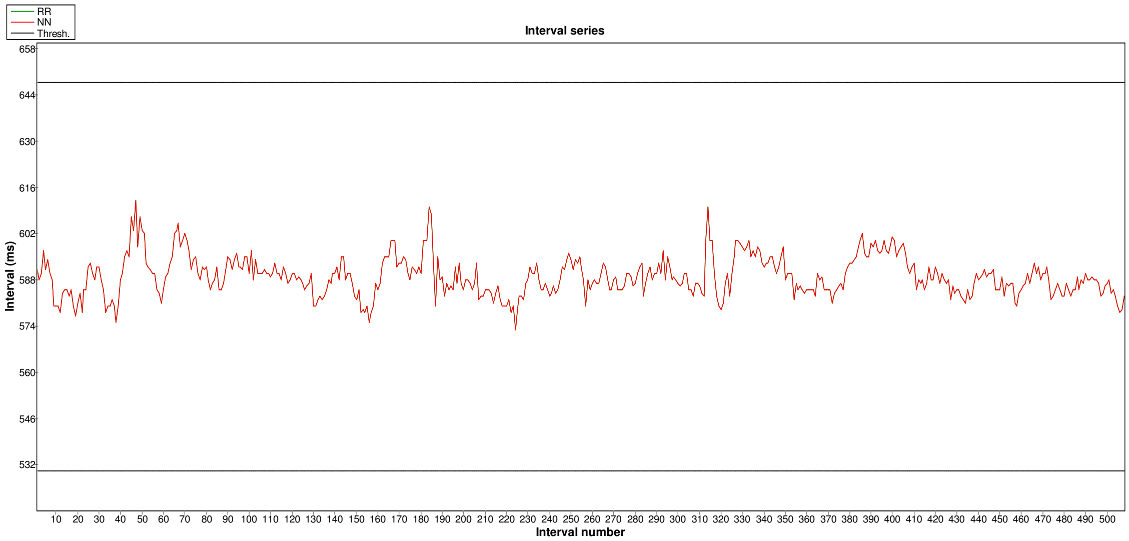

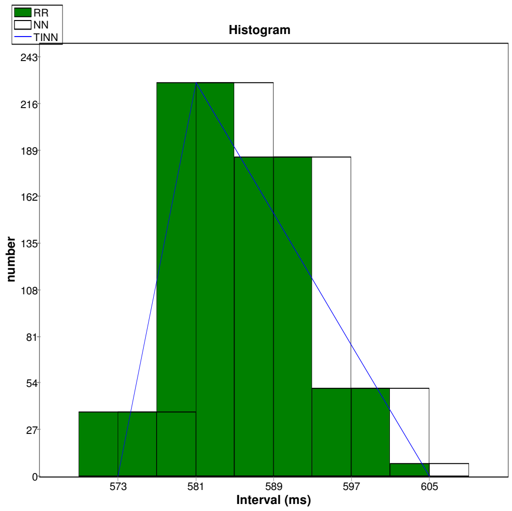

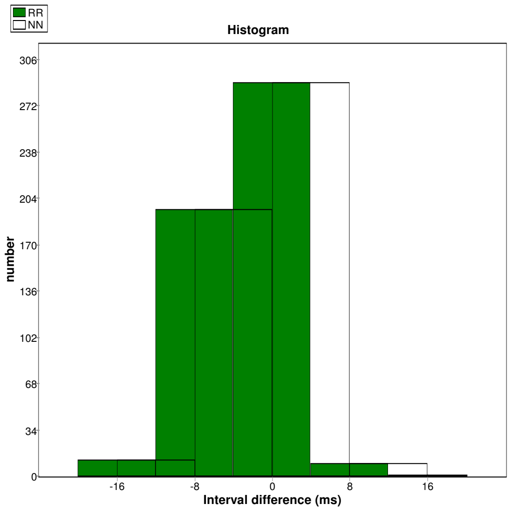

Binsize (ms) = 8

**HRV parameters**	**NN**	**RR**
SDNN (ms)	6	6
Triangular Interpolation (ms)	32	32
Triangular Index	2.23	2.23

**HRV parameters**	**NN**	**RR**
SDSD (ms)	4	4
RMSSD (ms)	4	4
NN50	0	0
NN50(1)	0	0
NN50(2)	0	0
pNN50	0.00	0.00
pNN50(1)	0.00	0.00
pNN50(2)	0.00	0.00
Logarithmic Index	2.74	2.74
SD(Logarithmic Index)	0.12	0.12

**Interval statistics**	**NN**	**RR**
Number	508	508
Minimum (ms)	573	573
Maximum (ms)	612	612
Range (ms)	39	39
Avg (ms)	589	589
SD (ms)	6	6
AvgDev (ms)	5	5
p5 (ms)	580	580
p50 (ms)	588	588
p95 (ms)	600	600
Skewness	0.58	0.58
Kurtosis	3.74	3.74

**Interval statistics**	**NN**	**RR**
Number	507	507
Minimum (ms)	-16	-16
Maximum (ms)	17	17
Range (ms)	33	33
Avg (ms)	-0	-0
SD (ms)	4	4
AvgDev (ms)	3	3
p5 (ms)	-7	-7
p50 (ms)	0	0
p95 (ms)	6	6
Skewness	-0.12	-0.12
Kurtosis	5.19	5.19

Cardio Control NV. User: Anton

Heart Rate Variability: Frequency Domain Analysis

**Name:**	001, 001 001	**Birthdate:**	01/06/1967
**Number:**	001	**Recorded:**	05/05/2018 12:33:43
**Gender:**	Male		

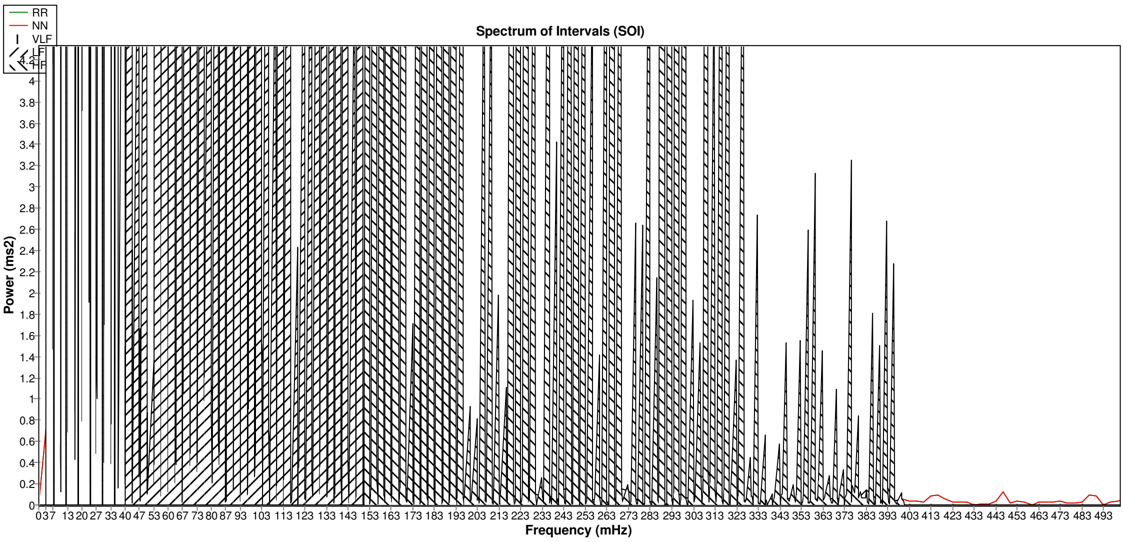

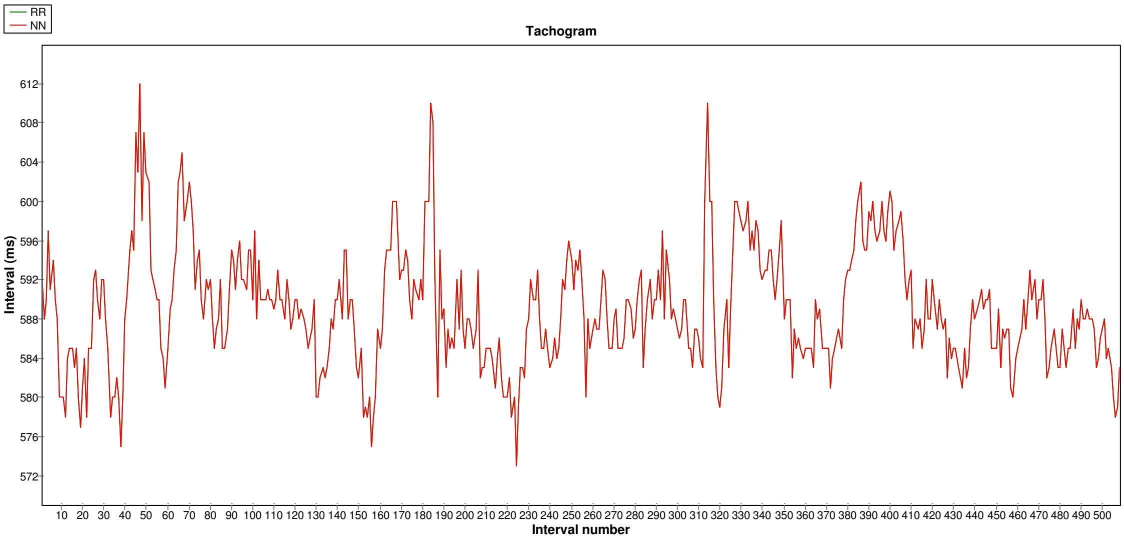

**HRV parameters**	**NN**	**RR**
TP (ms2)	29	29
VLF (ms2)	12	12
LF (ms2)	12	12
HF (ms2)	5	5
LF/HF	2.25	2.25
LF normalized	69.27	69.27
HF normalized	30.73	30.73
VLF peak (mHz)	23	23
LF peak (mHz)	103	103
HF peak (mHz)	310	310

**HRV spectral settings**	
Spectrum of Intervals (SOI)	
Frequency resolution (mHz)	3
VLF lower boundary (mHz)	3
VLF upper boundary (mHz)	40
LF upper boundary (mHz)	150
HF upper boundary (mHz)	400
Smoothing factor	1
Tapering	Hann
Fourier transform	DFT
Sample frequency (Hz)	1.70
Interval correction	Annotation
Interval threshold (%)	10
