# Supplementary material for: The effects of a 3-day mountain bike cycling race on the autonomic nervous system (ANS) and heart rate variability in amateur cyclists: a prospective quantitative research design
Source: BMC Sports Sci Med Rehabil. 2023 Jan 2;15:2. doi: 10.1186/s13102-022-00614-y (PMC9808932; doi:10.1186/s13102-022-00614-y)
Supplement: Supplementary file 1 — Additional file 1. Individual data of Participants. [file 13102_2022_614_MOESM1_ESM.zip › Individual data of Participants/HRV Data/001/ECG_001_20180506071748_.PDF]

Anton Swart Biokinetic Rehabilitation Practice

Name:	001 001 001	Recorded:	06/05/2018 07:17:48	UNCONFIRMED INTERPRETATION - MD SHOULD REVIEW
Number:	001	Recorded by:	Mr. Anton Swart	
Gender:	Male	Referring physician:		
Birthdate:	01/06/1967 50 years	Ordering physician:		
		Attending physician:		
P / PQ:	107 ms / 152 ms	Location:	Anton Swart Biokinetic Rehabilitation Practi	
QRS:	80 ms	Comment:		
QT / QTc / QTd:	406 ms / 441 ms / -			
P/QRS/T axis:	72° / 81° / 68°			
Heartrate:	80 bpm			

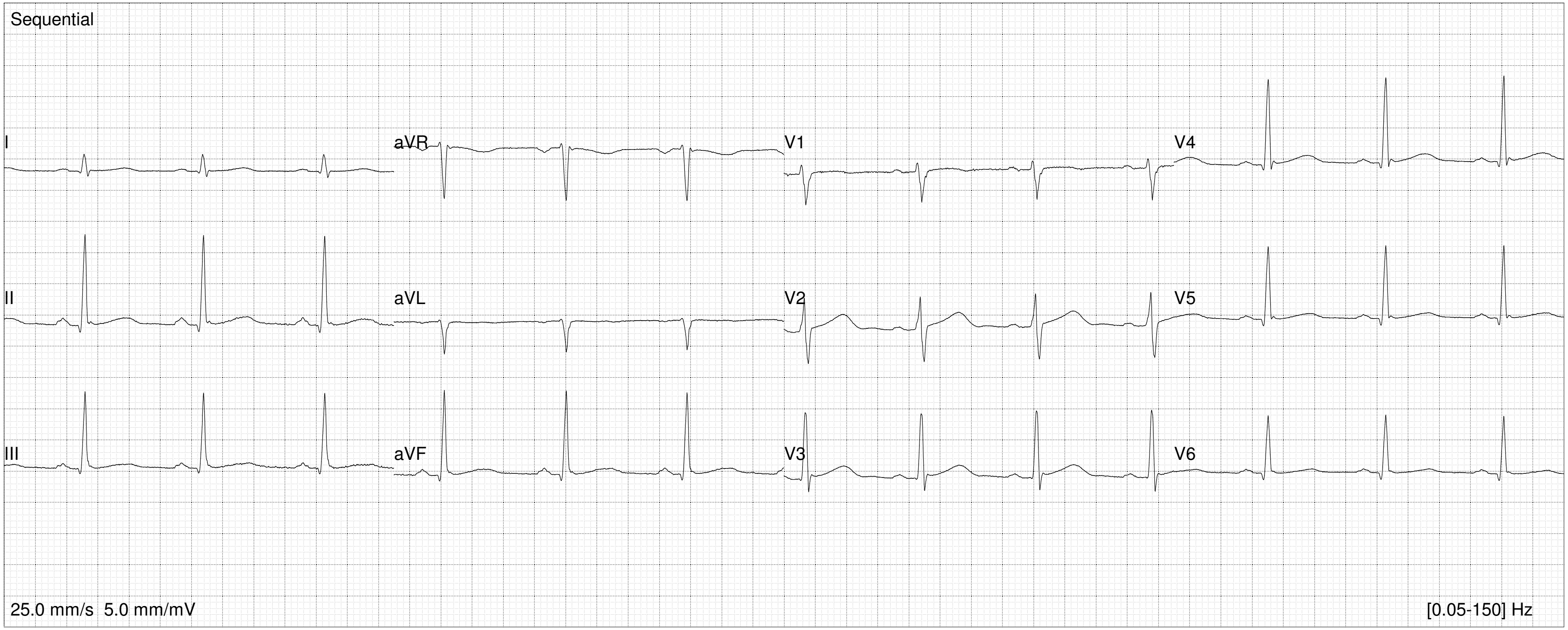

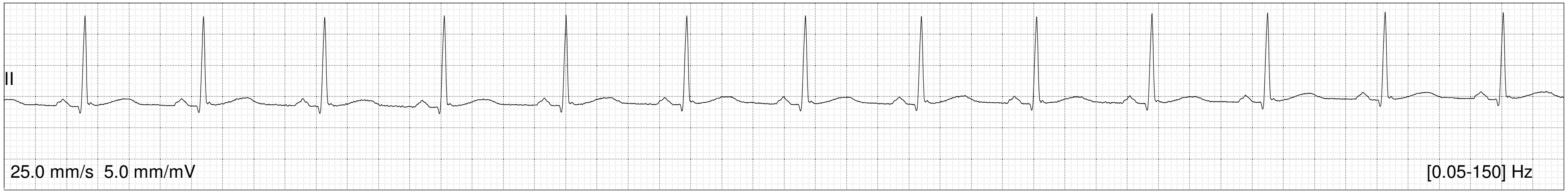

Anton Swart Biokinetic Rehabilitation Practice

Name:	001 001 001	Recorded:	06/05/2018 07:17:48	UNCONFIRMED INTERPRETATION - MD SHOULD REVIEW
Number:	001	Recorded by:	Mr. Anton Swart	
Gender:	Male	Referring physician:		
Birthdate:	01/06/1967 50 years	Location:	Anton Swart Biokinetic Rehabilitation Practice	
P / PQ:	107 ms / 152 ms	Ordering physician:		
QRS:	80 ms	Attending physician:		
QT / QTc / QTd:	406 ms / 441 ms / -	Comment:		
P/QRS/T axis:	72° / 81° / 68°			
Heartrate:	80 bpm			

Beats

Total:	396
Normal:	396
Other:	0

RR

Minimum	700 ms
Maximum	820 ms
Mean:	756 ms
SD:	18 ms

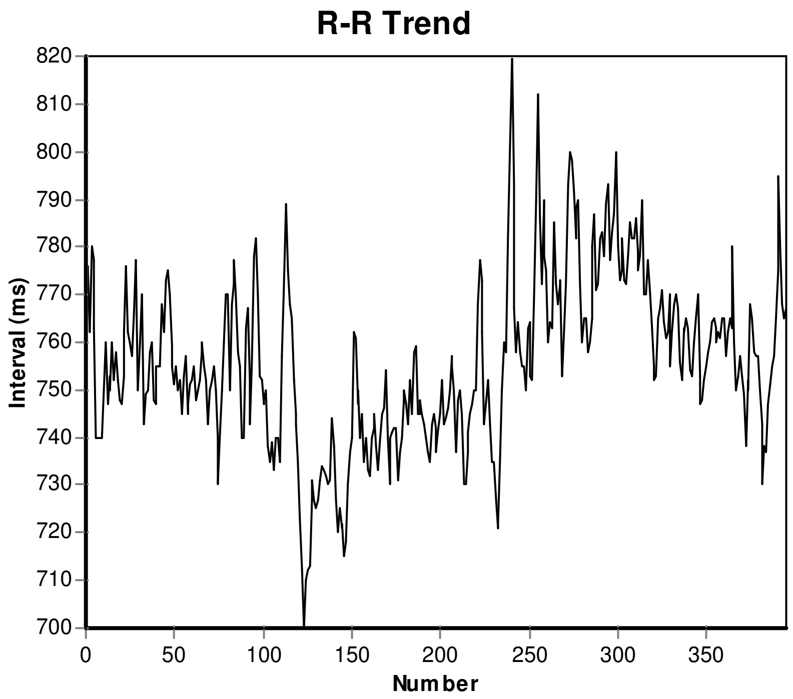

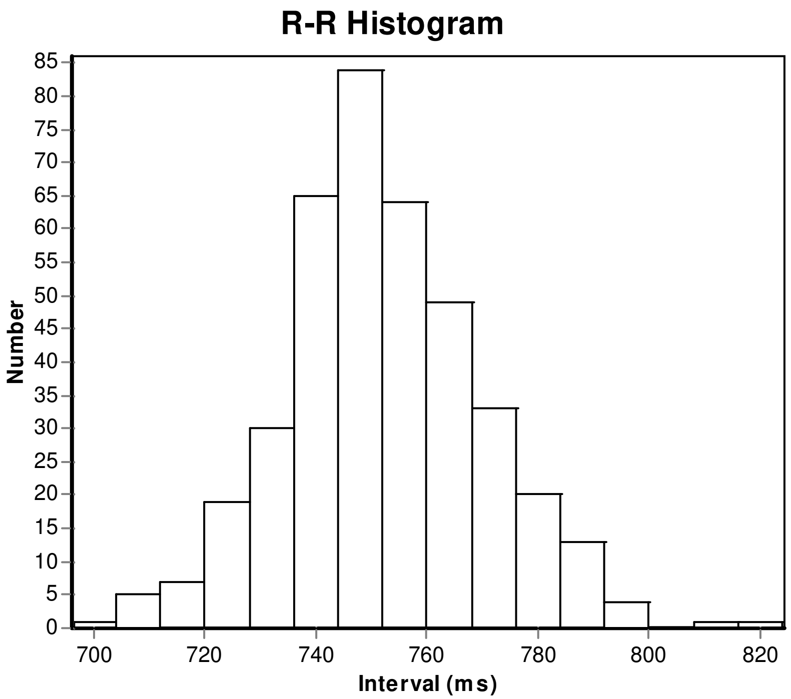

Cardio Control NV. User: Anton

Heart Rate Variability: Time Domain Analysis

**Name:**	001, 001 001	**Birthdate:**	01/06/1967
**Number:**	001	**Recorded:**	06/05/2018 07:17:48
**Gender:**	Male		

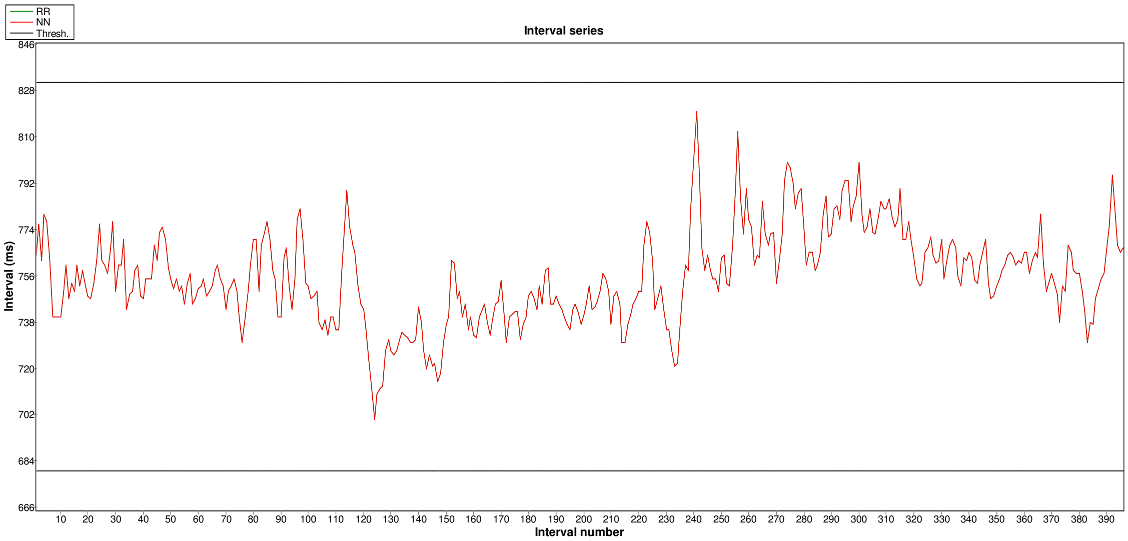

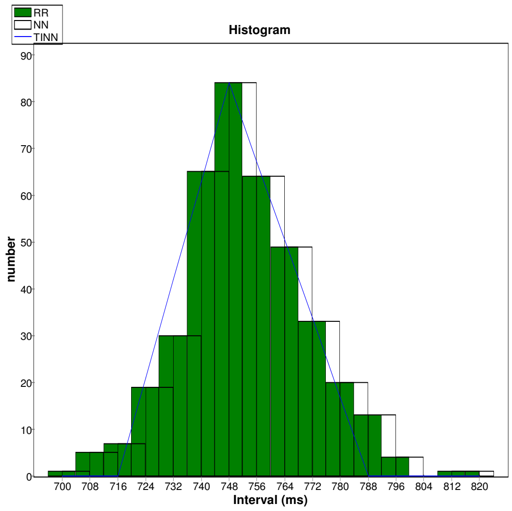

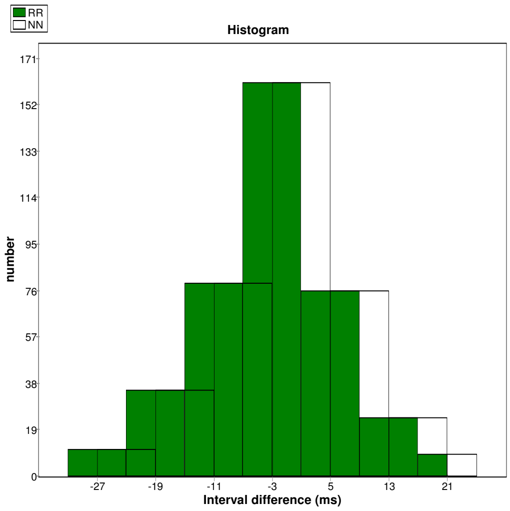

Binsize (ms) = 8

**HRV parameters**	**NN**	**RR**
SDNN (ms)	18	18
Triangular Interpolation (ms)	72	72
Triangular Index	4.71	4.71

**HRV parameters**	**NN**	**RR**
SDSD (ms)	9	9
RMSSD (ms)	9	9
NN50	0	0
NN50(1)	0	0
NN50(2)	0	0
pNN50	0.00	0.00
pNN50(1)	0.00	0.00
pNN50(2)	0.00	0.00
Logarithmic Index	1.24	1.24
SD(Logarithmic Index)	0.13	0.13

**Interval statistics**	**NN**	**RR**
Number	396	396
Minimum (ms)	700	700
Maximum (ms)	820	820
Range (ms)	120	120
Avg (ms)	756	756
SD (ms)	18	18
AvgDev (ms)	14	14
p5 (ms)	727	727
p50 (ms)	755	755
p95 (ms)	788	788
Skewness	0.20	0.20
Kurtosis	3.38	3.38

**Interval statistics**	**NN**	**RR**
Number	395	395
Minimum (ms)	-27	-27
Maximum (ms)	24	24
Range (ms)	51	51
Avg (ms)	0	0
SD (ms)	9	9
AvgDev (ms)	7	7
p5 (ms)	-15	-15
p50 (ms)	1	1
p95 (ms)	17	17
Skewness	-0.12	-0.12
Kurtosis	3.33	3.33

Heart Rate Variability: Frequency Domain Analysis

**Name:**	001, 001 001	**Birthdate:**	01/06/1967
**Number:**	001	**Recorded:**	06/05/2018 07:17:48
**Gender:**	Male		

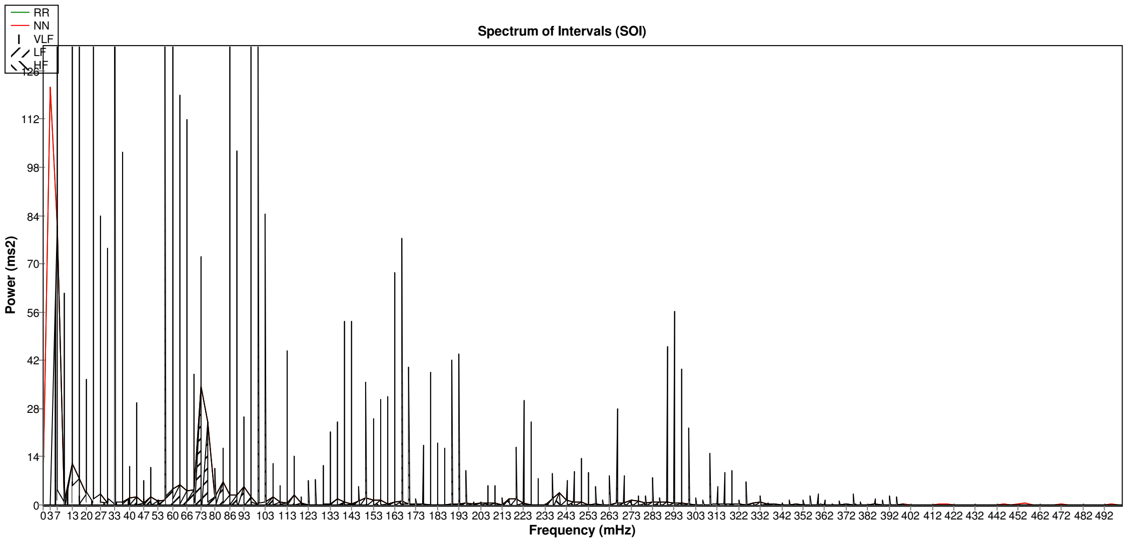

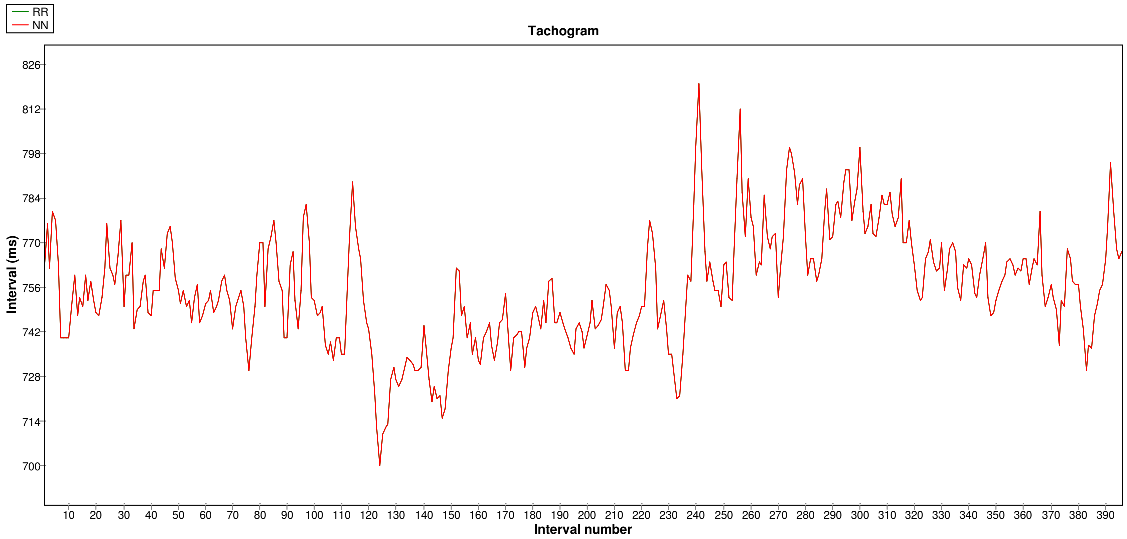

**HRV parameters**	**NN**	**RR**
TP (ms2)	273	273
VLF (ms2)	113	113
LF (ms2)	123	123
HF (ms2)	37	37
LF/HF	3.37	3.37
LF normalized	77.10	77.10
HF normalized	22.90	22.90
VLF peak (mHz)	7	7
LF peak (mHz)	73	73
HF peak (mHz)	239	239

**HRV spectral settings**	
Spectrum of Intervals (SOI)	
Frequency resolution (mHz)	3
VLF lower boundary (mHz)	3
VLF upper boundary (mHz)	40
LF upper boundary (mHz)	150
HF upper boundary (mHz)	400
Smoothing factor	1
Tapering	Hann
Fourier transform	DFT
Sample frequency (Hz)	1.32
Interval correction	Annotation
Interval threshold (%)	10
