# Supplementary material for: The effects of a 3-day mountain bike cycling race on the autonomic nervous system (ANS) and heart rate variability in amateur cyclists: a prospective quantitative research design
Source: BMC Sports Sci Med Rehabil. 2023 Jan 2;15:2. doi: 10.1186/s13102-022-00614-y (PMC9808932; doi:10.1186/s13102-022-00614-y)
Supplement: Supplementary file 1 — Additional file 1. Individual data of Participants. [file 13102_2022_614_MOESM1_ESM.zip › Individual data of Participants/HRV Data/002/ECG_002_20180501164336_.PDF]

Anton Swart Biokinetic Rehabilitation Practice

Name: 002 002 002
Number: 002
Gender: Male
Birthdate: 04/02/1978 40 years

P / PQ: 100 ms / 165 ms
QRS: 98 ms
QT / QTc / QTd: 432 ms / 408 ms / -
P/QRS/T axis: 32° / 36° / 63°
Heartrate: 46 bpm

Recorded: 01/05/2018 16:43:36
Recorded by: Mr. Anton Swart
Referring physician:
Ordering physician:
Attending physician:
Location: Anton Swart Biokinetic Rehabilitation Practi
Comment:

UNCONFIRMED INTERPRETATION - MD SHOULD REVIEW

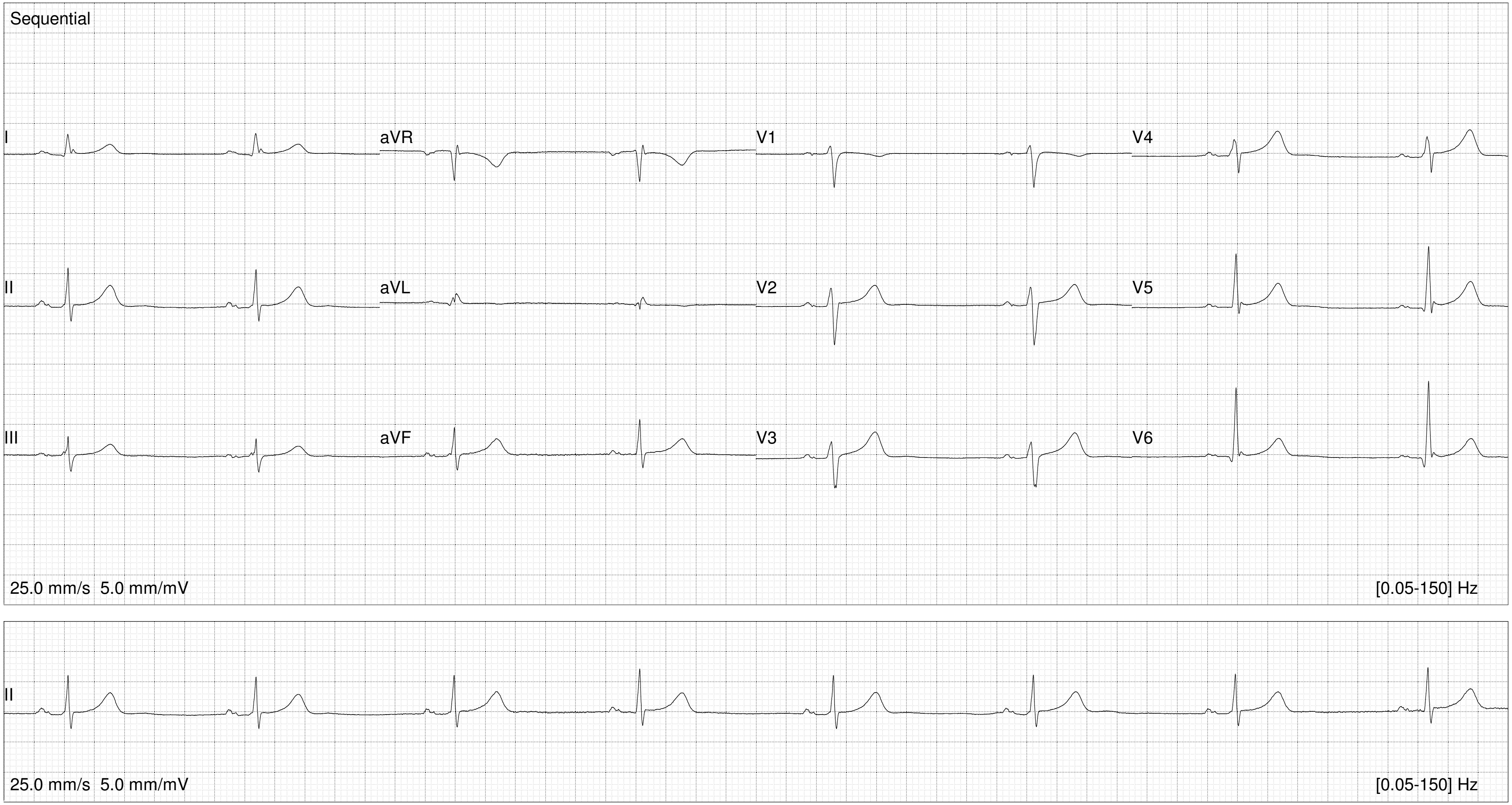

Anton Swart Biokinetic Rehabilitation Practice

Name:	002 002 002	Recorded:	01/05/2018 16:43:36	UNCONFIRMED INTERPRETATION - MD SHOULD REVIEW
Number:	002	Recorded by:	Mr. Anton Swart	
Gender:	Male	Referring physician:		
Birthdate:	04/02/1978 40 years	Location:	Anton Swart Biokinetic Rehabilitation Practice	
P / PQ:	100 ms / 165 ms	Ordering physician:		
QRS:	98 ms	Attending physician:		
QT / QTc / QTd:	432 ms / 408 ms / -	Comment:		
P/QRS/T axis:	32° / 36° / 63°			
Heartrate:	46 bpm			

Beats

Total:	229
Normal:	229
Other:	0

RR

Minimum	950 ms
Maximum	1518 ms
Mean:	1303 ms
SD:	86 ms

R-R Trend

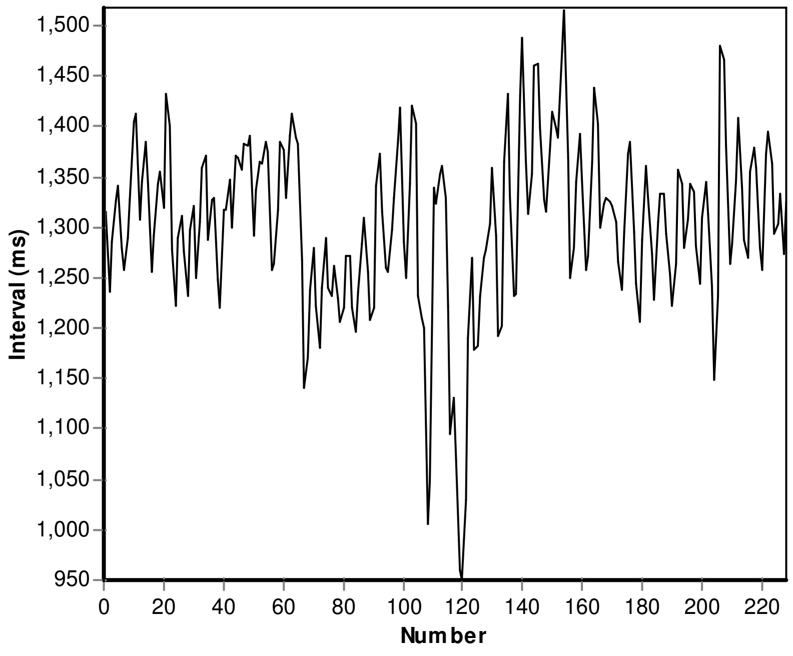

R-R Histogram

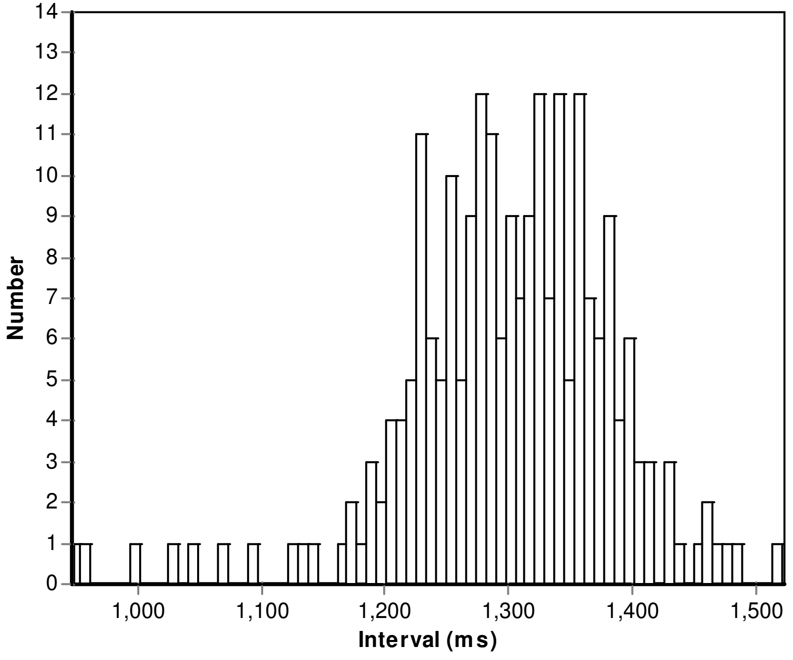

Cardio Control NV. User: Anton

Heart Rate Variability: Time Domain Analysis

**Name:**	002, 002 002	**Birthdate:**	04/02/1978
**Number:**	002	**Recorded:**	01/05/2018 16:43:36
**Gender:**	Male		

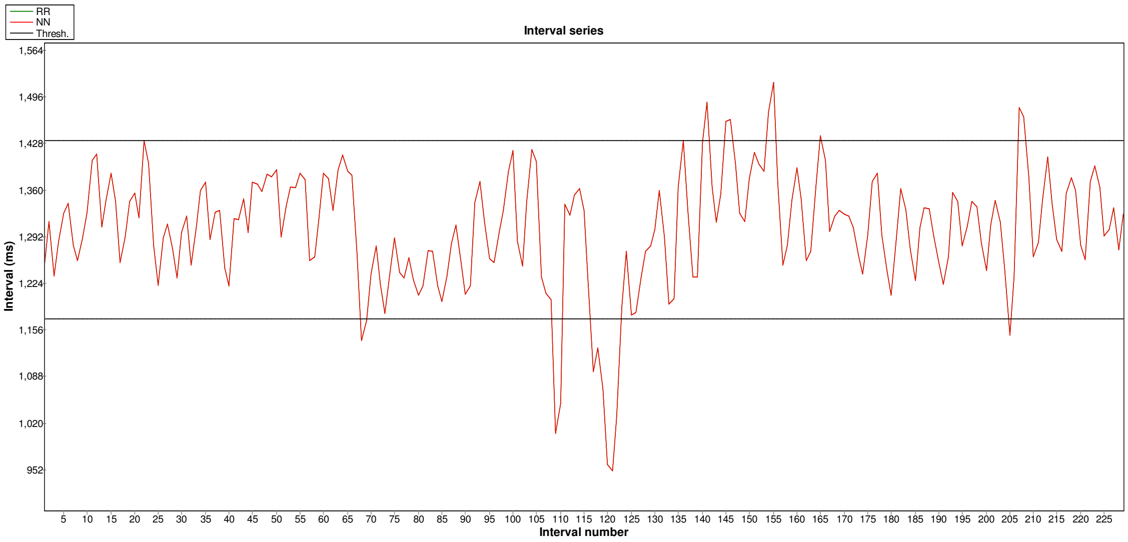

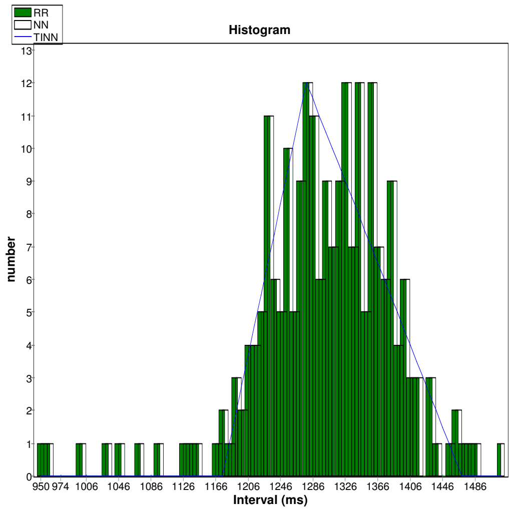

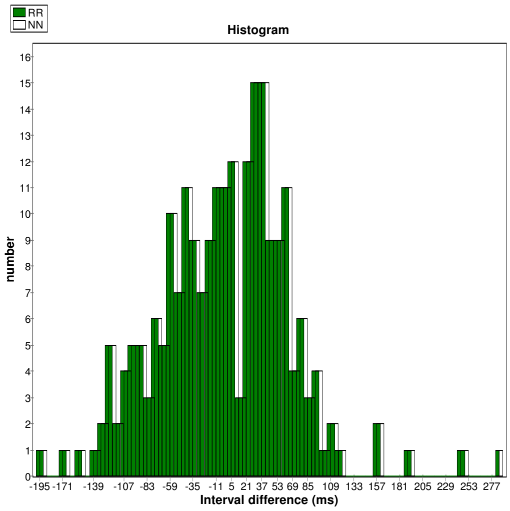

Binsize (ms) = 8

**HRV parameters**	**NN**	**RR**
SDNN (ms)	86	86
Triangular Interpolation (ms)	296	296
Triangular Index	19.08	19.08

**HRV parameters**	**NN**	**RR**
SDSD (ms)	68	68
RMSSD (ms)	68	68
NN50	101	101
NN50(1)	52	52
NN50(2)	49	49
pNN50	0.44	0.44
pNN50(1)	0.23	0.23
pNN50(2)	0.21	0.21
Logarithmic Index	0.17	0.17
SD(Logarithmic Index)	0.01	0.01

**Interval statistics**	**NN**	**RR**
Number	229	229
Minimum (ms)	950	950
Maximum (ms)	1518	1518
Range (ms)	568	568
Avg (ms)	1303	1303
SD (ms)	86	86
AvgDev (ms)	64	64
p5 (ms)	1178	1178
p50 (ms)	1309	1309
p95 (ms)	1430	1430
Skewness	-0.92	-0.92
Kurtosis	5.53	5.53

**Interval statistics**	**NN**	**RR**
Number	228	228
Minimum (ms)	-195	-195
Maximum (ms)	292	292
Range (ms)	487	487
Avg (ms)	0	0
SD (ms)	68	68
AvgDev (ms)	53	53
p5 (ms)	-116	-116
p50 (ms)	3	3
p95 (ms)	94	94
Skewness	0.33	0.33
Kurtosis	4.51	4.51

Heart Rate Variability: Frequency Domain Analysis

**Name:**	002, 002 002	**Birthdate:**	04/02/1978
**Number:**	002	**Recorded:**	01/05/2018 16:43:36
**Gender:**	Male		

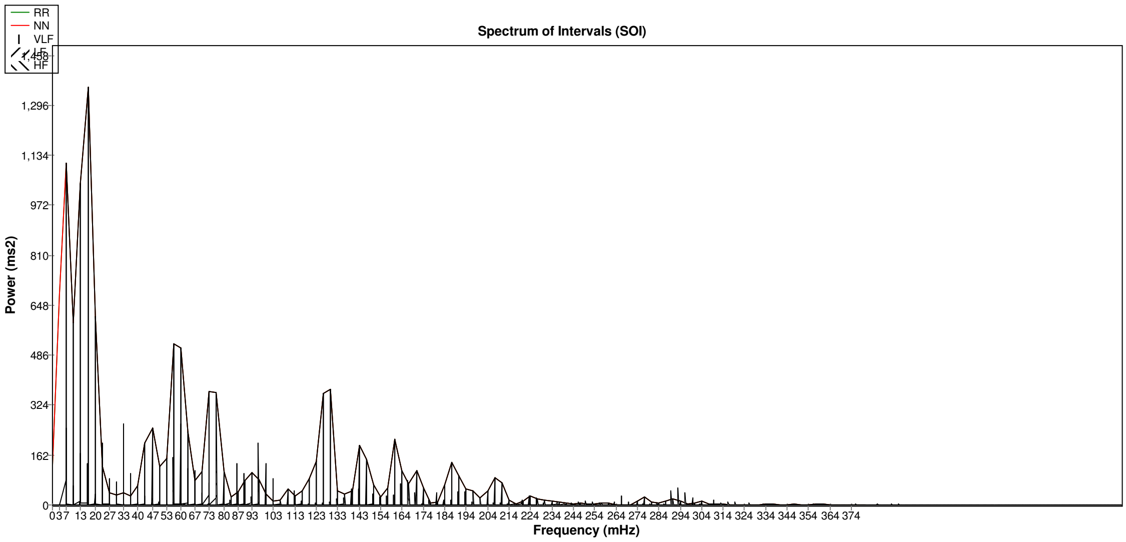

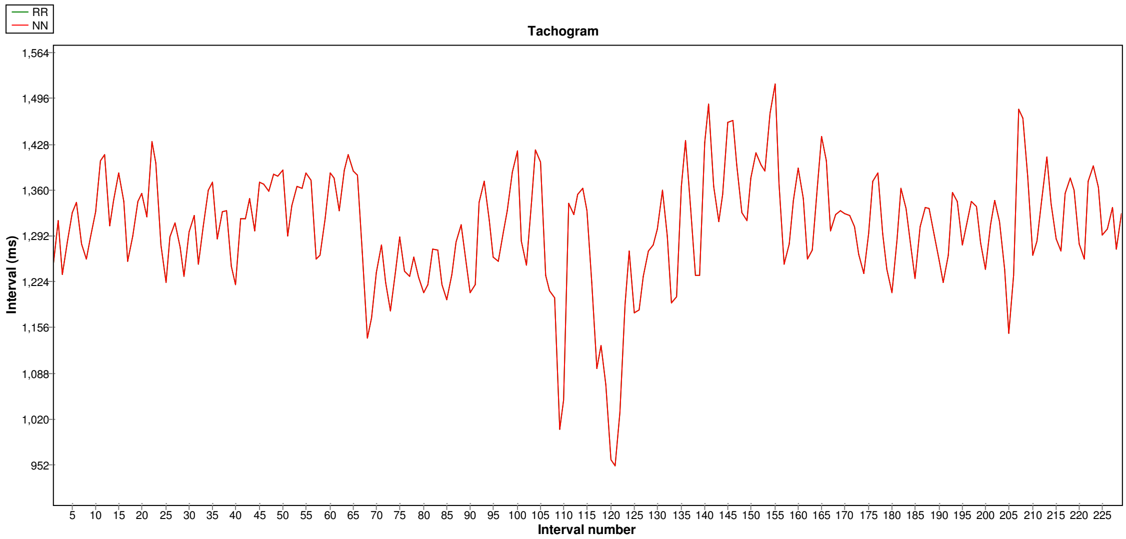

HRV parameters	NN	RR
TP (ms2)	11710	11710
VLF (ms2)	5043	5043
LF (ms2)	4994	4994
HF (ms2)	1673	1673
LF/HF	2.99	2.99
LF normalized	74.91	74.91
HF normalized	25.09	25.09
VLF peak (mHz)	17	17
LF peak (mHz)	57	57
HF peak (mHz)	160	160

HRV spectral settings	
Spectrum of Intervals (SOI)	
Frequency resolution (mHz)	3
VLF lower boundary (mHz)	3
VLF upper boundary (mHz)	40
LF upper boundary (mHz)	150
HF upper boundary (mHz)	400
Smoothing factor	1
Tapering	Hann
Fourier transform	DFT
Sample frequency (Hz)	0.77
Interval correction	Annotation
Interval threshold (%)	10
