# Supplementary material for: The effects of a 3-day mountain bike cycling race on the autonomic nervous system (ANS) and heart rate variability in amateur cyclists: a prospective quantitative research design
Source: BMC Sports Sci Med Rehabil. 2023 Jan 2;15:2. doi: 10.1186/s13102-022-00614-y (PMC9808932; doi:10.1186/s13102-022-00614-y)
Supplement: Supplementary file 1 — Additional file 1. Individual data of Participants. [file 13102_2022_614_MOESM1_ESM.zip › Individual data of Participants/HRV Data/002/ECG_002_20180503172231_.PDF]

# Anton Swart Biokinetic Rehabilitation Practice

| | | | | |
|---|---|---|---|---|
| Name: | 002 002 002 | Recorded: | 03/05/2018 17:22:31 | UNCONFIRMED INTERPRETATION - MD SHOULD REVIEW |
| Number: | 002 | Recorded by: | Mr. Anton Swart | |
| Gender: | Male | Referring physician: | | |
| Birthdate: | 04/02/1978  40 years | Ordering physician: | | |
| | | Attending physician: | | |
| | | Location: | Anton Swart Biokinetic Rehabilitation Practi | |
| | | Comment: | | |

| | |
|---|---|
| P / PQ: | 123 ms / 188 ms |
| QRS: | 94 ms |
| QT / QTc / QTd: | 370 ms / 398 ms / - |
| P/QRS/T axis: | 66° / 46° / 67° |
| Heartrate: | 76 bpm |

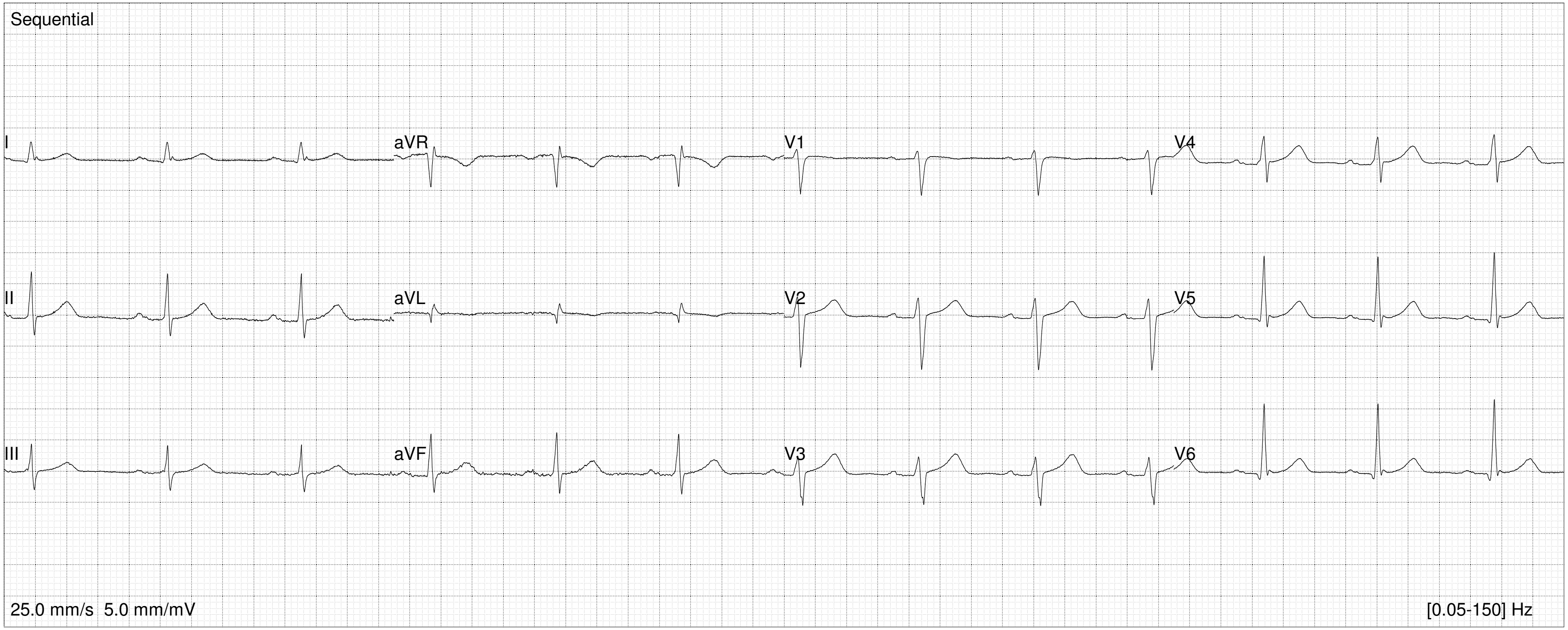


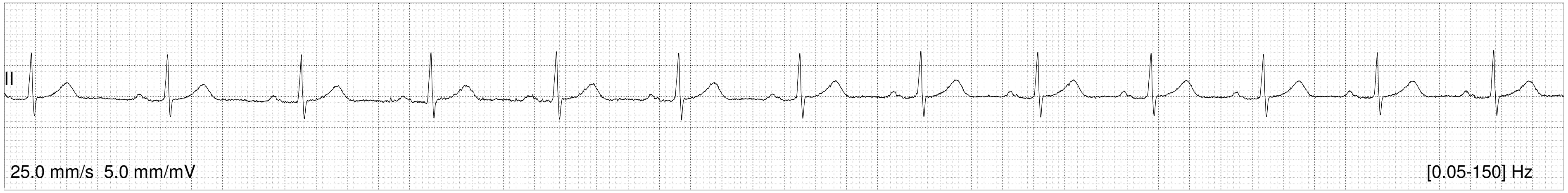

# Anton Swart Biokinetic Rehabilitation Practice

| | | | | |
|---|---|---|---|---|
| Name: | 002 002 002 | Recorded: | 03/05/2018 17:22:31 | UNCONFIRMED INTERPRETATION - MD SHOULD REVIEW |
| Number: | 002 | Recorded by: | Mr. Anton Swart | |
| Gender: | Male | Referring physician: | | |
| Birthdate: | 04/02/1978 40 years | Location: | Anton Swart Biokinetic Rehabilitation Practice | |
| | | Ordering physician: | | |
| | | Attending physician: | | |
| | | Comment: | | |

| | |
|---|---|
| P / PQ: | 123 ms / 188 ms |
| QRS: | 94 ms |
| QT / QTc / QTd: | 370 ms / 398 ms / - |
| P/QRS/T axis: | 66° / 46° / 67° |
| Heartrate: | 76 bpm |

**Beats**

| | |
|---|---|
| Total: | 381 |
| Normal: | 381 |
| Other: | 0 |

**RR**

| | |
|---|---|
| Minimum | 650 ms |
| Maximum | 918 ms |
| Mean: | 785 ms |
| SD: | 49 ms |

## R-R Trend

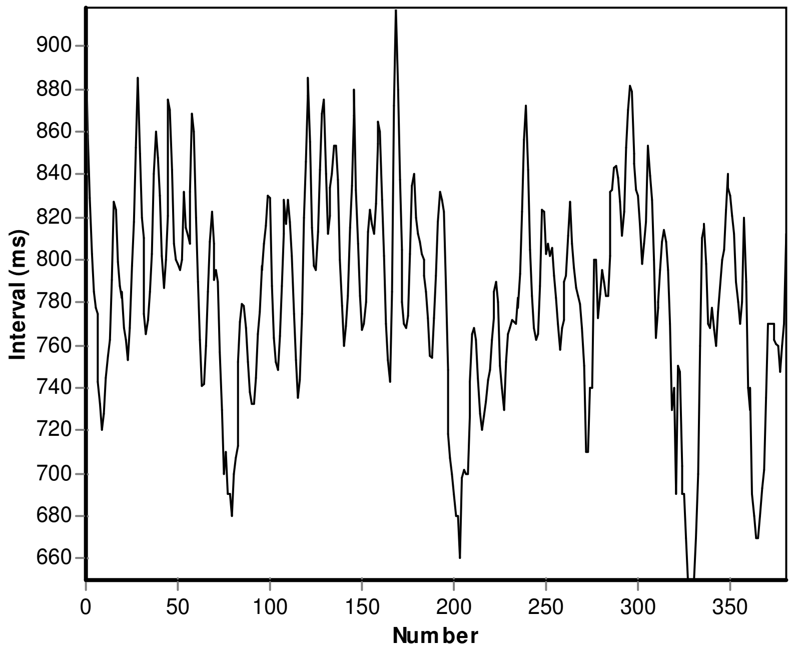


## R-R Histogram

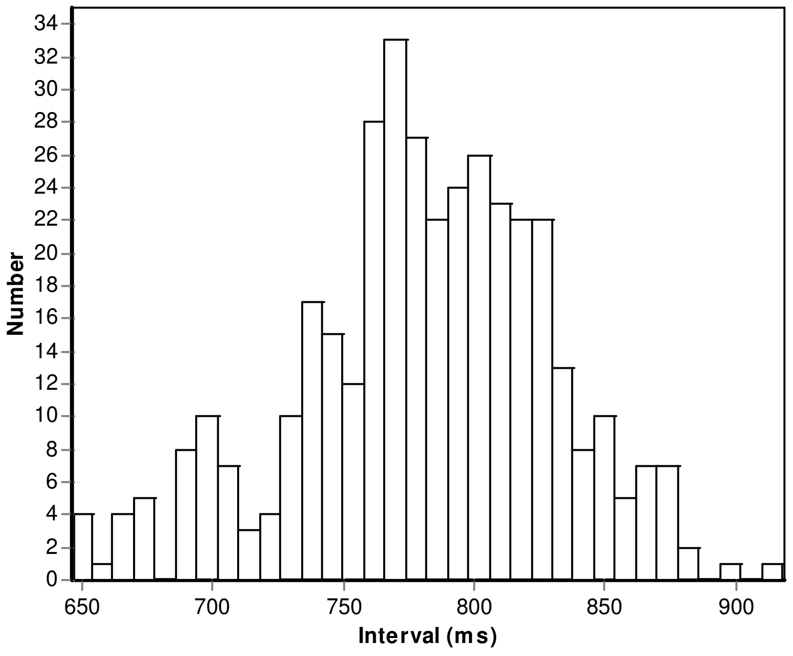

# Cardio Control NV. User: Anton

## Heart Rate Variability: Time Domain Analysis

| | | | |
|---|---|---|---|
| **Name:** | 002, 002 002 | **Birthdate:** | 04/02/1978 |
| **Number:** | 002 | **Recorded:** | 03/05/2018 17:22:31 |
| **Gender:** | Male | | |

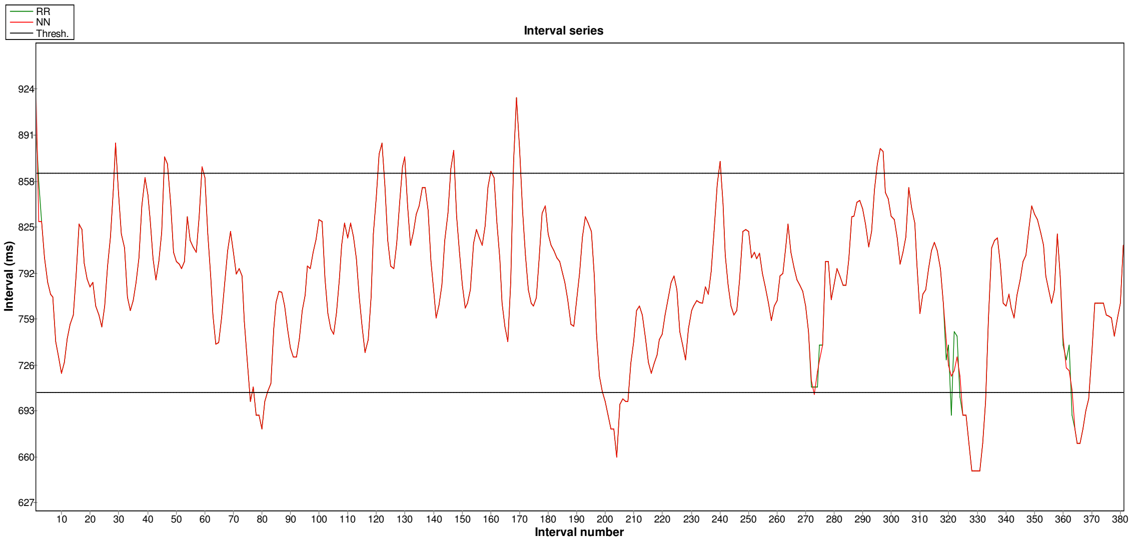


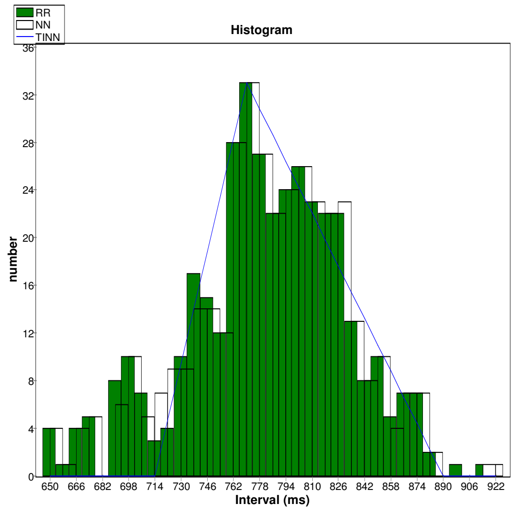


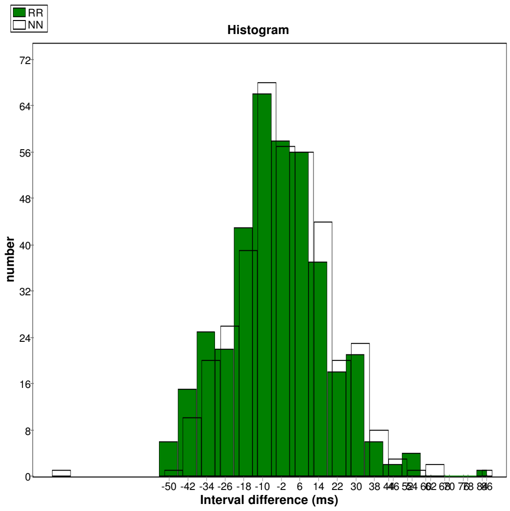


Binsize (ms) = 8

| **HRV parameters** | **NN** | **RR** |
|---|---|---|
| SDNN (ms) | 49 | 49 |
| Triangular Interpolation (ms) | 176 | 176 |
| Triangular Index | 11.55 | 11.55 |

| **HRV parameters** | **NN** | **RR** |
|---|---|---|
| SDSD (ms) | 21 | 21 |
| RMSSD (ms) | 21 | 21 |
| NN50 | 5 | 5 |
| NN50(1) | 1 | 0 |
| NN50(2) | 4 | 5 |
| pNN50 | 0.01 | 0.01 |
| pNN50(1) | 0.00 | 0.00 |
| pNN50(2) | 0.01 | 0.01 |
| Logarithmic Index | 0.62 | 0.59 |
| SD(Logarithmic Index) | 0.06 | 0.05 |

| **Interval statistics** | **NN** | **RR** |
|---|---|---|
| Number | 381 | 381 |
| Minimum (ms) | 650 | 650 |
| Maximum (ms) | 929 | 918 |
| Range (ms) | 279 | 268 |
| Avg (ms) | 785 | 785 |
| SD (ms) | 49 | 49 |
| AvgDev (ms) | 38 | 38 |
| p5 (ms) | 693 | 690 |
| p50 (ms) | 787 | 787 |
| p95 (ms) | 865 | 865 |
| Skewness | -0.32 | -0.36 |
| Kurtosis | 3.16 | 3.10 |

| **Interval statistics** | **NN** | **RR** |
|---|---|---|
| Number | 380 | 380 |
| Minimum (ms) | -100 | -50 |
| Maximum (ms) | 87 | 87 |
| Range (ms) | 187 | 137 |
| Avg (ms) | -0 | -0 |
| SD (ms) | 21 | 21 |
| AvgDev (ms) | 16 | 17 |
| p5 (ms) | -32 | -35 |
| p50 (ms) | -1 | -1 |
| p95 (ms) | 34 | 34 |
| Skewness | 0.08 | 0.28 |
| Kurtosis | 4.44 | 3.47 |

Cardio Control NV. User: Anton

## Heart Rate Variability: Frequency Domain Analysis

| | | | |
|---|---|---|---|
| **Name:** | 002, 002 002 | **Birthdate:** | 04/02/1978 |
| **Number:** | 002 | **Recorded:** | 03/05/2018 17:22:31 |
| **Gender:** | Male | | |

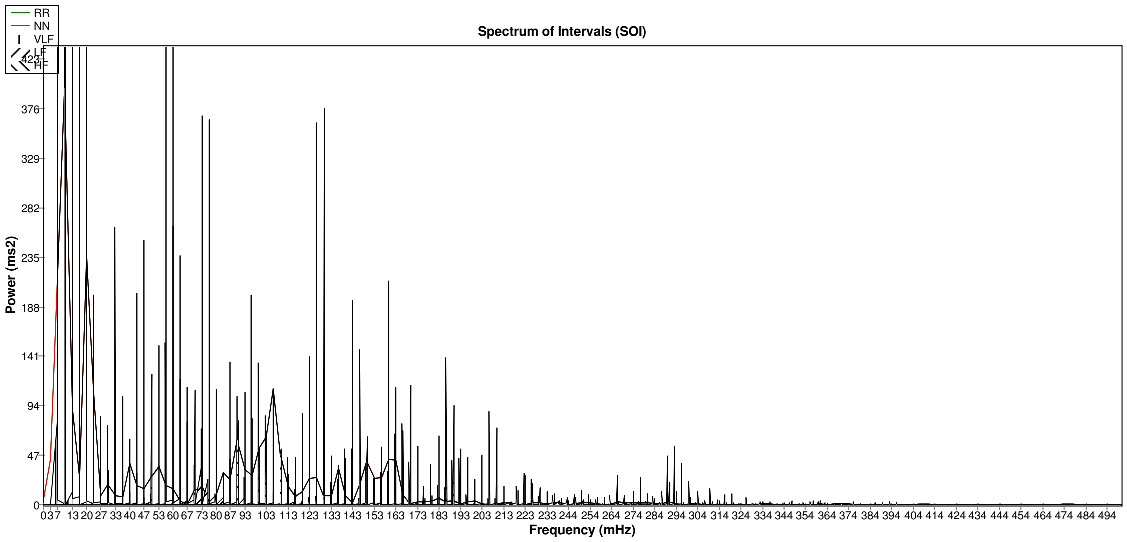


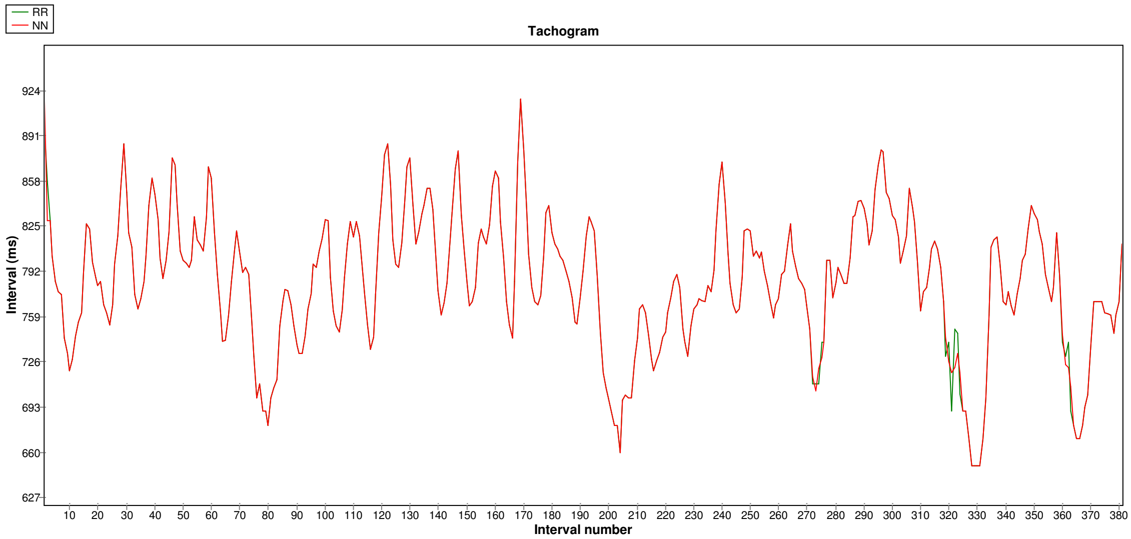


| **HRV parameters** | **NN** | **RR** |
|---|---|---|
| TP (ms2) | 2223 | 2224 |
| VLF (ms2) | 1154 | 1154 |
| LF (ms2) | 803 | 804 |
| HF (ms2) | 266 | 266 |
| LF/HF | 3.02 | 3.02 |
| LF normalized | 75.12 | 75.12 |
| HF normalized | 24.88 | 24.88 |
| VLF peak (mHz) | 10 | 10 |
| LF peak (mHz) | 107 | 107 |
| HF peak (mHz) | 160 | 160 |

| **HRV spectral settings** | |
|---|---|
| Spectrum of Intervals (SOI) | |
| Frequency resolution (mHz) | 3 |
| VLF lower boundary (mHz) | 3 |
| VLF upper boundary (mHz) | 40 |
| LF upper boundary (mHz) | 150 |
| HF upper boundary (mHz) | 400 |
| Smoothing factor | 1 |
| Tapering | Hann |
| Fourier transform | DFT |
| Sample frequency (Hz) | 1.27 |
| Interval correction | Annotation |
| Interval threshold (%) | 10 |
