# Supplementary material for: The effects of a 3-day mountain bike cycling race on the autonomic nervous system (ANS) and heart rate variability in amateur cyclists: a prospective quantitative research design
Source: BMC Sports Sci Med Rehabil. 2023 Jan 2;15:2. doi: 10.1186/s13102-022-00614-y (PMC9808932; doi:10.1186/s13102-022-00614-y)
Supplement: Supplementary file 1 — Additional file 1. Individual data of Participants. [file 13102_2022_614_MOESM1_ESM.zip › Individual data of Participants/HRV Data/002/ECG_002_20180504120937_.PDF]

Anton Swart Biokinetic Rehabilitation Practice

Name:	002 002 002	Recorded:	04/05/2018 12:09:37	UNCONFIRMED INTERPRETATION - MD SHOULD REVIEW
Number:	002	Recorded by:	Mr. Anton Swart	
Gender:	Male	Referring physician:		
Birthdate:	04/02/1978 40 years	Ordering physician:		
		Attending physician:		
P / PQ:	125 ms / 177 ms	Location:	Anton Swart Biokinetic Rehabilitation Practi	
QRS:	93 ms	Comment:		
QT / QTc / QTd:	351 ms / 421 ms / -			
P/QRS/T axis:	73° / 53° / 71°			
Heartrate:	100 bpm			

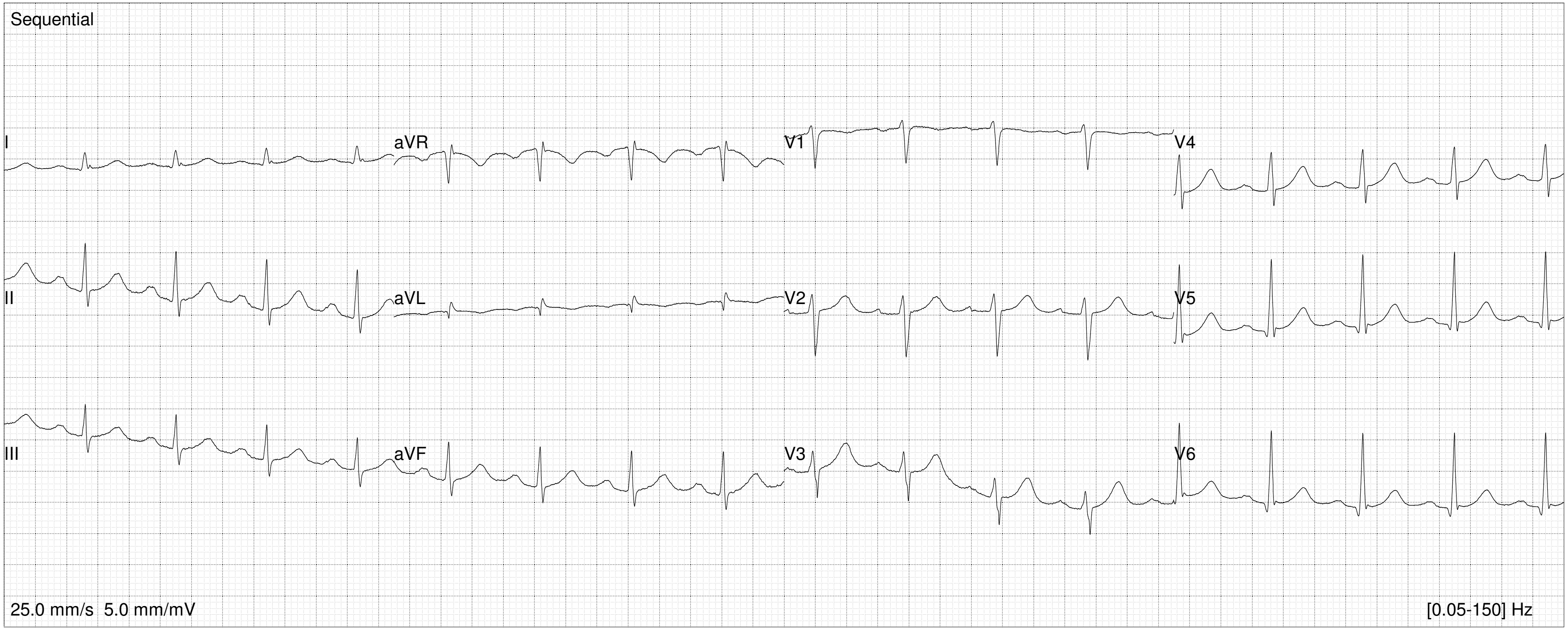

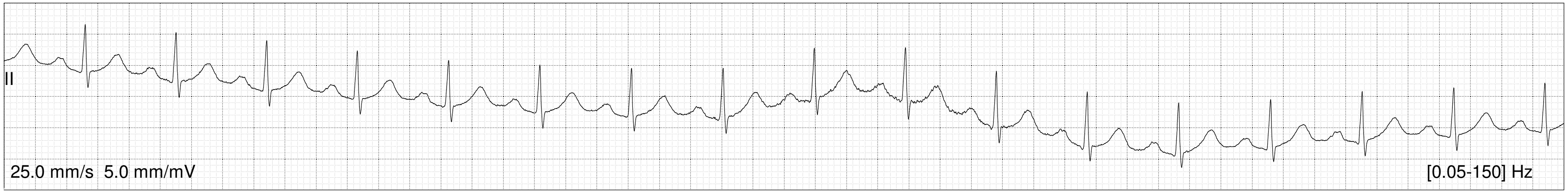

Anton Swart Biokinetic Rehabilitation Practice

Name:	002 002 002	Recorded:	04/05/2018 12:09:37	UNCONFIRMED INTERPRETATION - MD SHOULD REVIEW
Number:	002	Recorded by:	Mr. Anton Swart	
Gender:	Male	Referring physician:		
Birthdate:	04/02/1978 40 years	Location:	Anton Swart Biokinetic Rehabilitation Practice	
		Ordering physician:		
		Attending physician:		
		Comment:		

P / PQ:	125 ms / 177 ms
QRS:	93 ms
QT / QTc / QTd:	351 ms / 421 ms / -
P/QRS/T axis:	73° / 53° / 71°
Heartrate:	100 bpm

Beats

Total:	502
Normal:	502
Other:	0

RR

Minimum	570 ms
Maximum	623 ms
Mean:	596 ms
SD:	10 ms

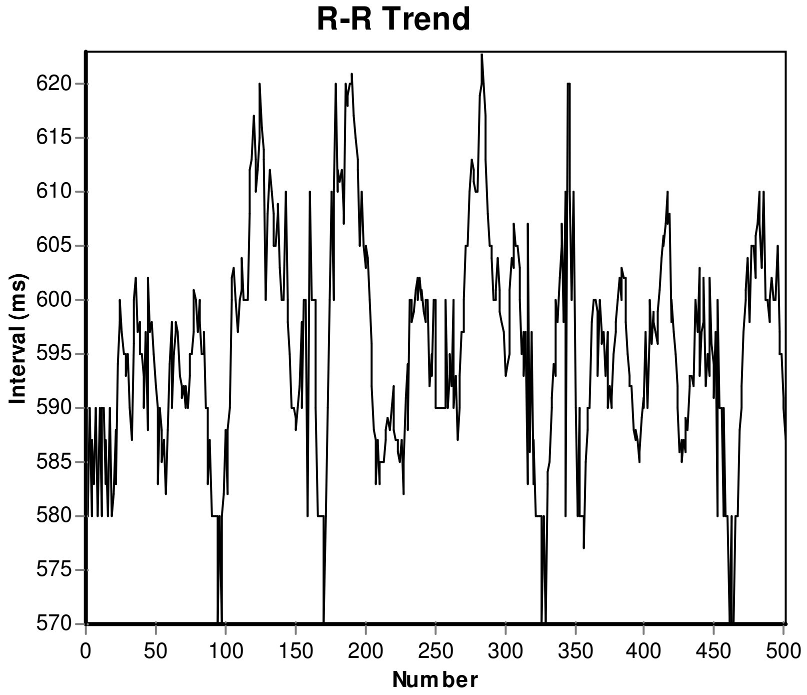

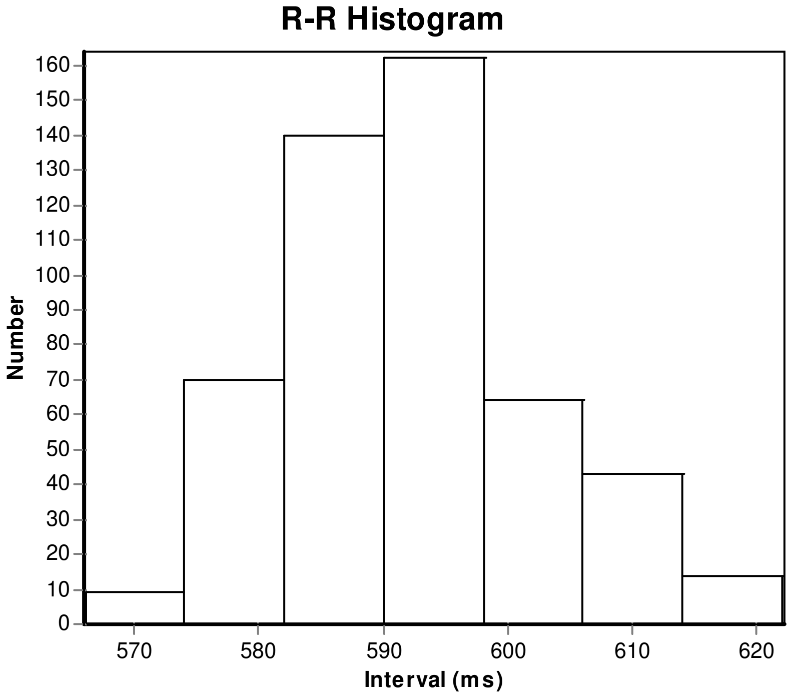

Cardio Control NV. User: Anton

Heart Rate Variability: Time Domain Analysis

**Name:**	002, 002 002	**Birthdate:**	04/02/1978
**Number:**	002	**Recorded:**	04/05/2018 12:09:37
**Gender:**	Male		

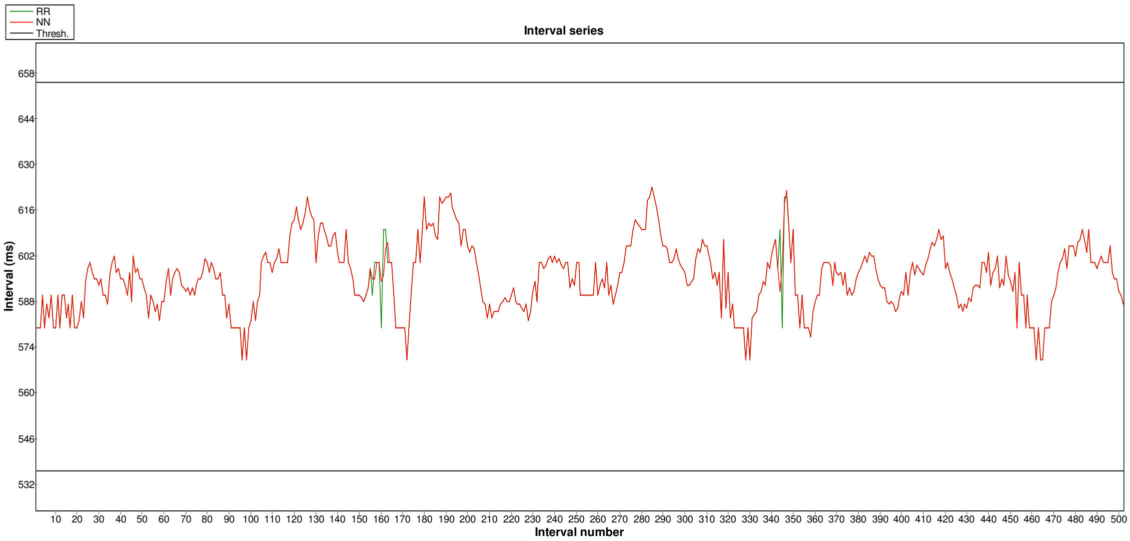

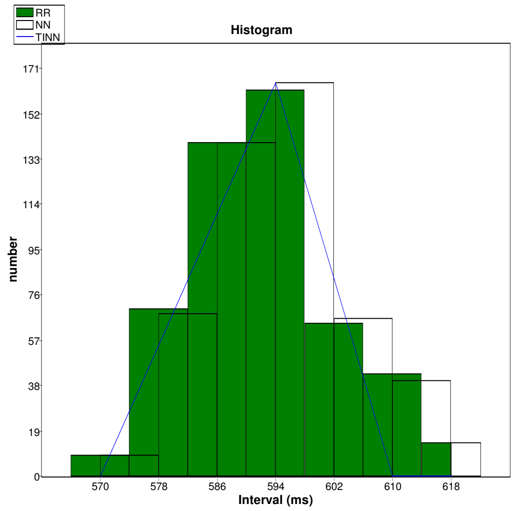

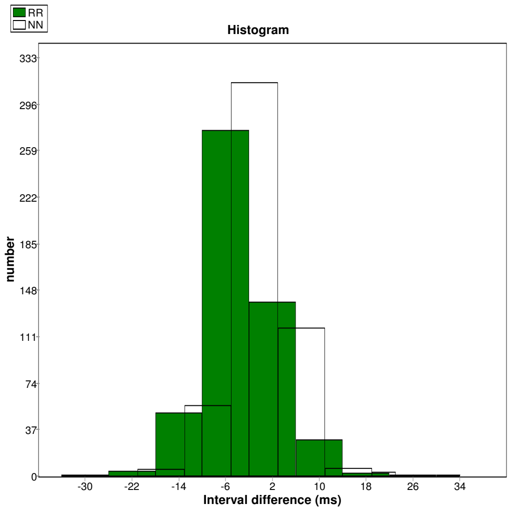

Binsize (ms) = 8

**HRV parameters**	**NN**	**RR**
SDNN (ms)	10	10
Triangular Interpolation (ms)	40	48
Triangular Index	3.04	3.10

**HRV parameters**	**NN**	**RR**
SDSD (ms)	5	6
RMSSD (ms)	5	6
NN50	0	0
NN50(1)	0	0
NN50(2)	0	0
pNN50	0.00	0.00
pNN50(1)	0.00	0.00
pNN50(2)	0.00	0.00
Logarithmic Index	2.08	1.53
SD(Logarithmic Index)	0.15	0.21

**Interval statistics**	**NN**	**RR**
Number	502	502
Minimum (ms)	570	570
Maximum (ms)	623	623
Range (ms)	53	53
Avg (ms)	596	596
SD (ms)	10	10
AvgDev (ms)	8	8
p5 (ms)	580	580
p50 (ms)	596	596
p95 (ms)	613	613
Skewness	0.13	0.13
Kurtosis	2.99	2.92

**Interval statistics**	**NN**	**RR**
Number	501	501
Minimum (ms)	-21	-30
Maximum (ms)	24	40
Range (ms)	45	70
Avg (ms)	0	0
SD (ms)	5	6
AvgDev (ms)	4	4
p5 (ms)	-10	-10
p50 (ms)	0	0
p95 (ms)	10	10
Skewness	0.05	0.51
Kurtosis	4.98	9.93

Cardio Control NV. User: Anton

Heart Rate Variability: Frequency Domain Analysis

**Name:**	002, 002 002	**Birthdate:**	04/02/1978
**Number:**	002	**Recorded:**	04/05/2018 12:09:37
**Gender:**	Male		

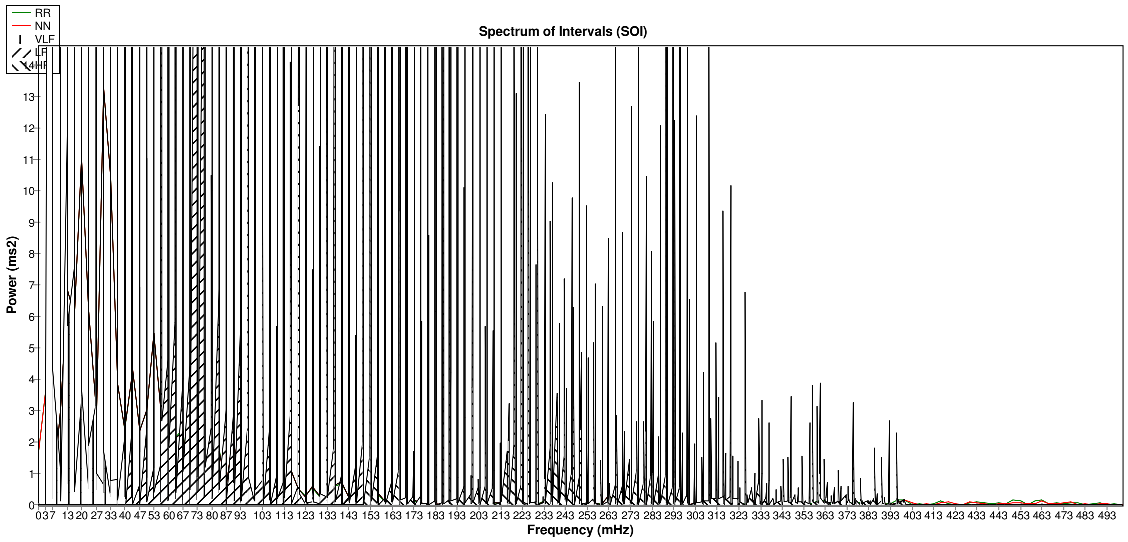

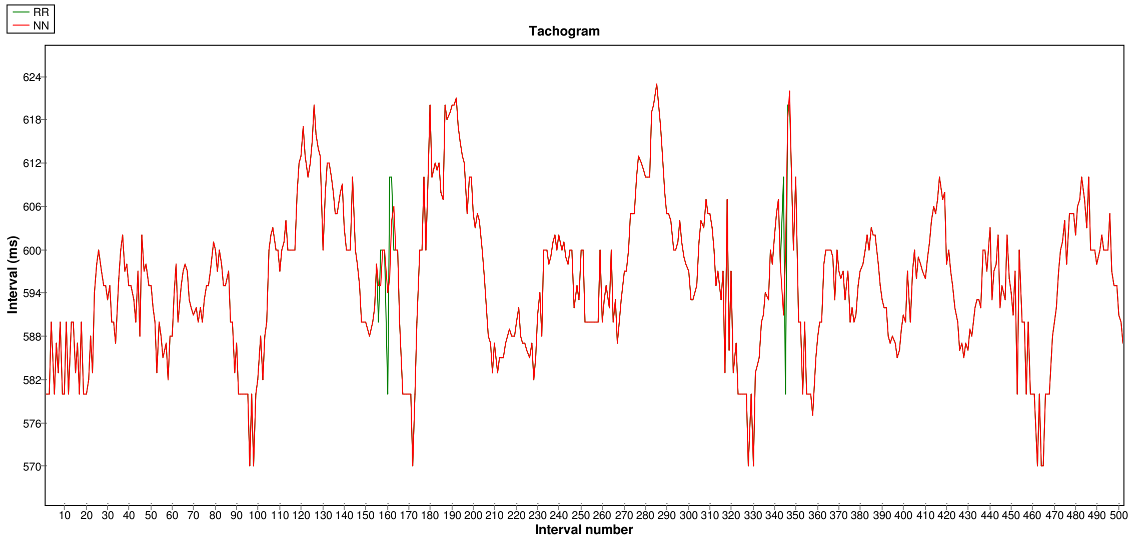

**HRV parameters**	**NN**	**RR**
TP (ms2)	107	106
VLF (ms2)	66	66
LF (ms2)	35	35
HF (ms2)	5	5
LF/HF	6.36	6.69
LF normalized	86.41	87.00
HF normalized	13.59	13.00
VLF peak (mHz)	30	30
LF peak (mHz)	53	53
HF peak (mHz)	157	153

**HRV spectral settings**	
Spectrum of Intervals (SOI)	
Frequency resolution (mHz)	3
VLF lower boundary (mHz)	3
VLF upper boundary (mHz)	40
LF upper boundary (mHz)	150
HF upper boundary (mHz)	400
Smoothing factor	1
Tapering	Hann
Fourier transform	DFT
Sample frequency (Hz)	1.68
Interval correction	Annotation
Interval threshold (%)	10
