# Supplementary material for: The effects of a 3-day mountain bike cycling race on the autonomic nervous system (ANS) and heart rate variability in amateur cyclists: a prospective quantitative research design
Source: BMC Sports Sci Med Rehabil. 2023 Jan 2;15:2. doi: 10.1186/s13102-022-00614-y (PMC9808932; doi:10.1186/s13102-022-00614-y)
Supplement: Supplementary file 1 — Additional file 1. Individual data of Participants. [file 13102_2022_614_MOESM1_ESM.zip › Individual data of Participants/HRV Data/002/ECG_002_20180505122625_.PDF]

Anton Swart Biokinetic Rehabilitation Practice

Name: 002 002 002
Number: 002
Gender: Male
Birthdate: 04/02/1978 40 years

P / PQ: 120 ms / 177 ms
QRS: 91 ms
QT / QTc / QTd: 353 ms / 418 ms / -
P/QRS/T axis: 75° / 47° / 73°
Heartrate: 97 bpm

Recorded: 05/05/2018 12:26:25
Recorded by: Mr. Anton Swart
Referring physician:
Ordering physician:
Attending physician:
Location: Anton Swart Biokinetic Rehabilitation Practi
Comment:

UNCONFIRMED INTERPRETATION - MD SHOULD REVIEW

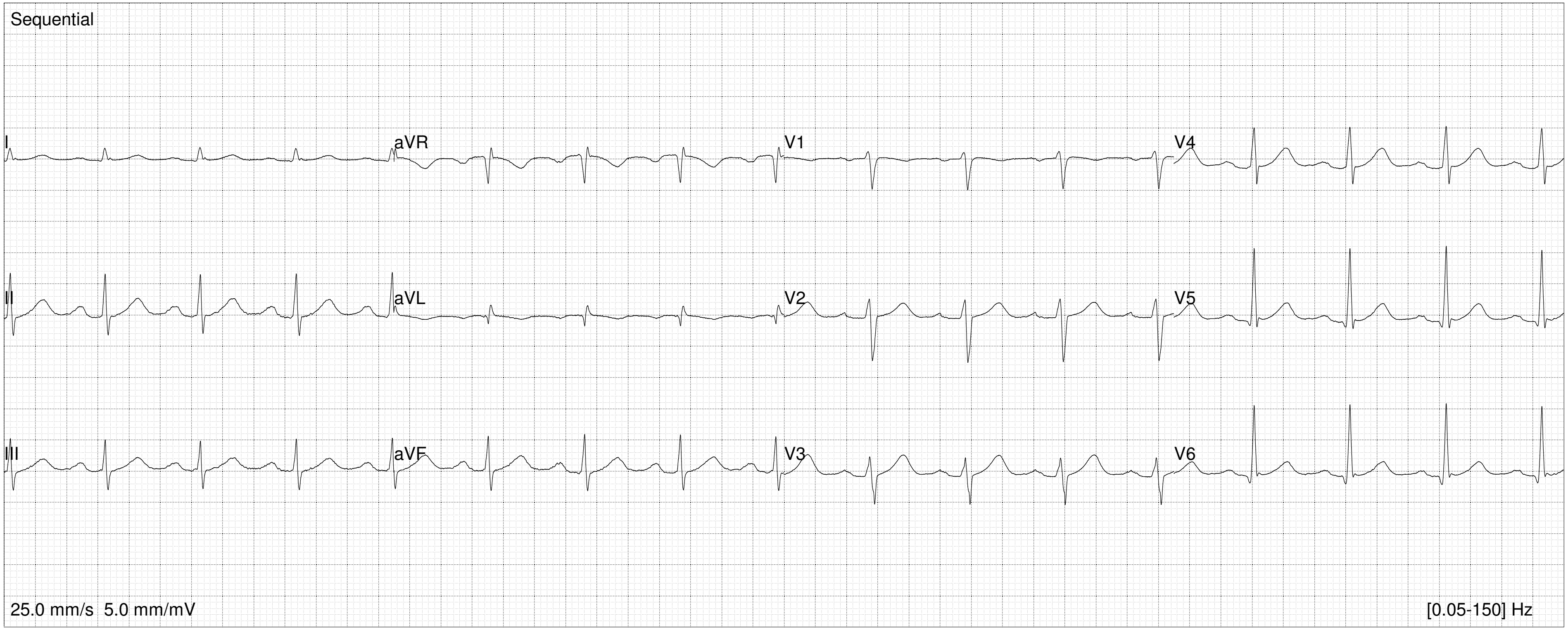

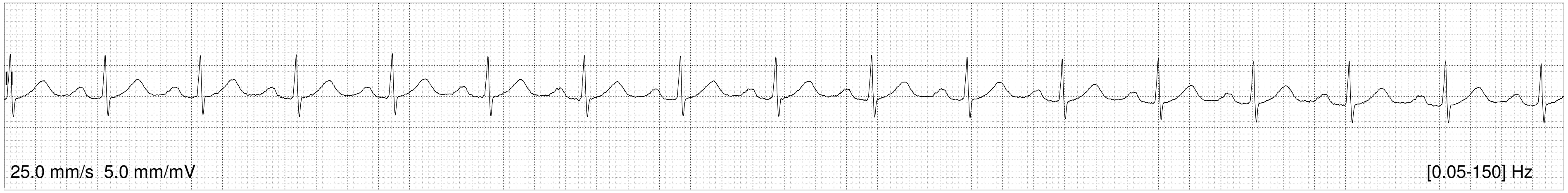

Anton Swart Biokinetic Rehabilitation Practice

Name:	002 002 002	Recorded:	05/05/2018 12:26:25	UNCONFIRMED INTERPRETATION - MD SHOULD REVIEW
Number:	002	Recorded by:	Mr. Anton Swart	
Gender:	Male	Referring physician:		
Birthdate:	04/02/1978 40 years	Location:	Anton Swart Biokinetic Rehabilitation Practice	
P / PQ:	120 ms / 177 ms	Ordering physician:		
QRS:	91 ms	Attending physician:		
QT / QTc / QTd:	353 ms / 418 ms / -	Comment:		
P/QRS/T axis:	75° / 47° / 73°			
Heartrate:	97 bpm			

**Beats**		**RR**	
Total:	484	Minimum	570 ms
Normal:	484	Maximum	660 ms
Other:	0	Mean:	618 ms
		SD:	21 ms

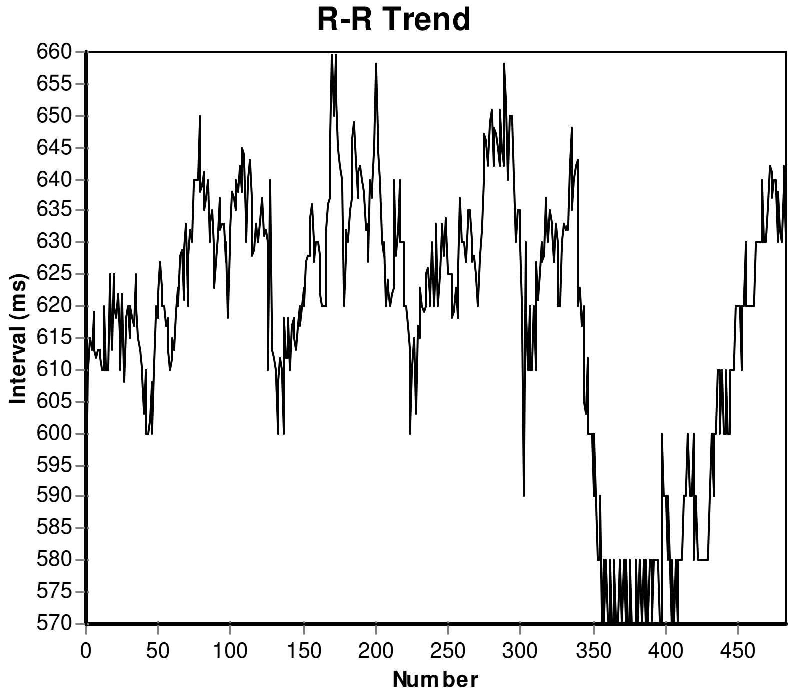

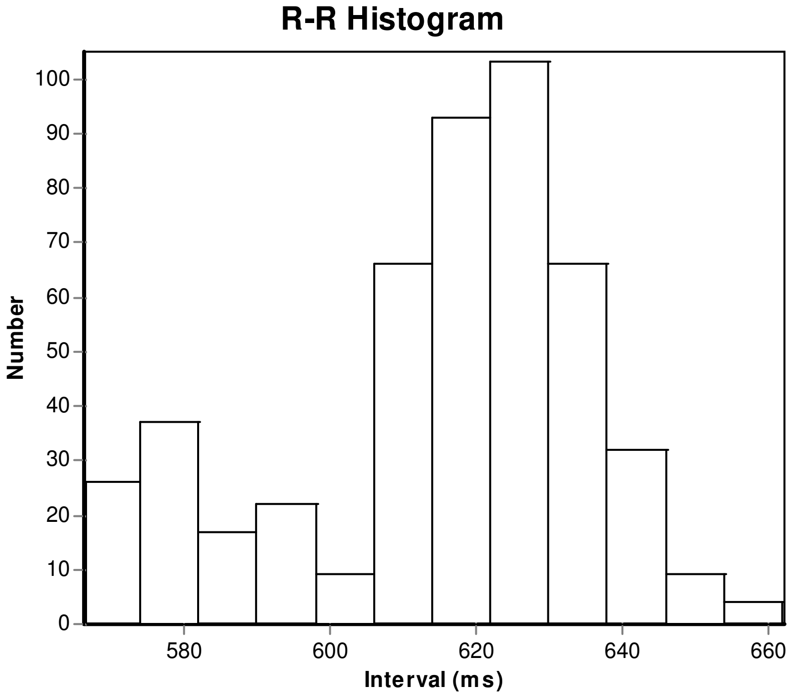

Heart Rate Variability: Time Domain Analysis

**Name:**	002, 002 002	**Birthdate:**	04/02/1978
**Number:**	002	**Recorded:**	05/05/2018 12:26:25
**Gender:**	Male		

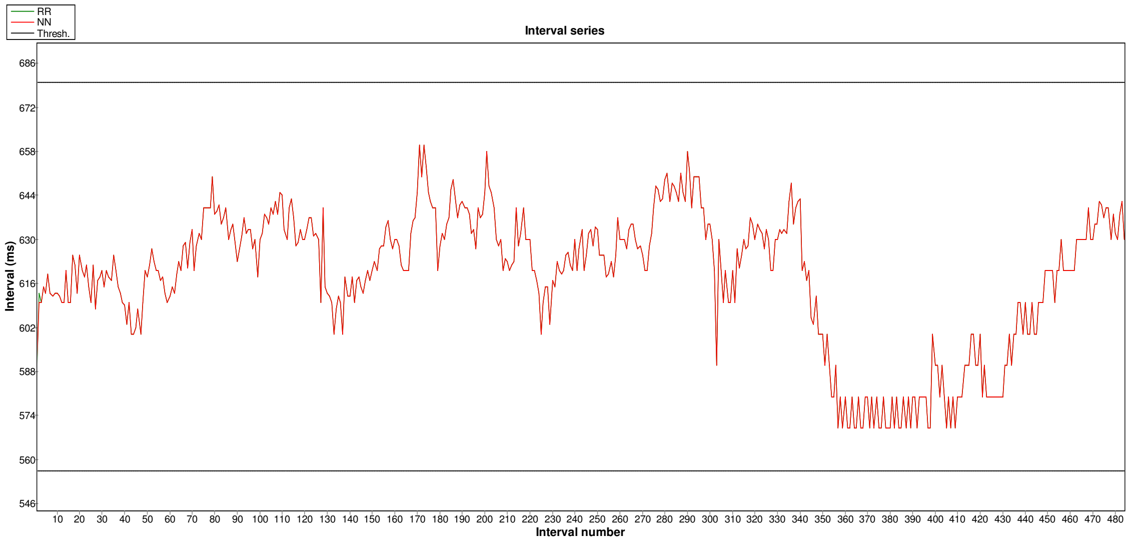

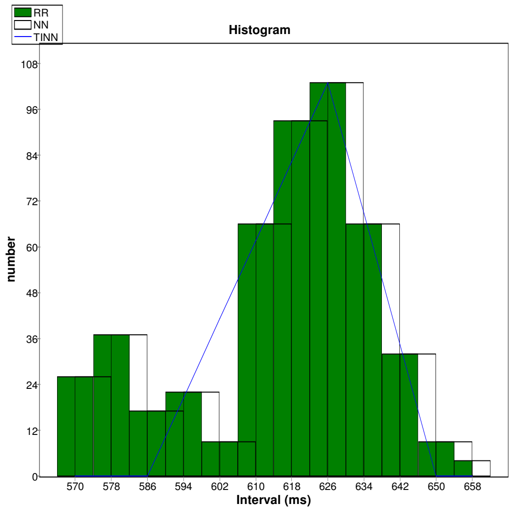

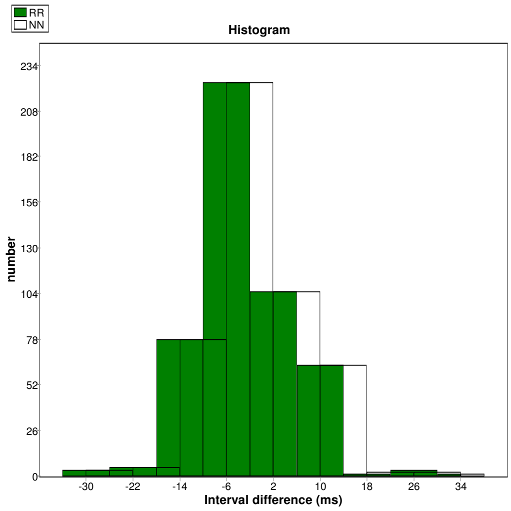

Binsize (ms) = 8

**HRV parameters**	**NN**	**RR**
SDNN (ms)	21	21
Triangular Interpolation (ms)	64	64
Triangular Index	4.70	4.70

**HRV parameters**	**NN**	**RR**
SDSD (ms)	8	8
RMSSD (ms)	8	8
NN50	0	0
NN50(1)	0	0
NN50(2)	0	0
pNN50	0.00	0.00
pNN50(1)	0.00	0.00
pNN50(2)	0.00	0.00
Logarithmic Index	1.51	1.49
SD(Logarithmic Index)	0.16	0.18

**Interval statistics**	**NN**	**RR**
Number	484	484
Minimum (ms)	570	570
Maximum (ms)	660	660
Range (ms)	90	90
Avg (ms)	618	618
SD (ms)	21	21
AvgDev (ms)	17	17
p5 (ms)	570	570
p50 (ms)	622	622
p95 (ms)	645	645
Skewness	-0.79	-0.79
Kurtosis	2.85	2.85

**Interval statistics**	**NN**	**RR**
Number	483	483
Minimum (ms)	-30	-30
Maximum (ms)	40	40
Range (ms)	70	70
Avg (ms)	0	0
SD (ms)	8	8
AvgDev (ms)	6	6
p5 (ms)	-10	-10
p50 (ms)	0	0
p95 (ms)	10	10
Skewness	0.23	0.27
Kurtosis	5.26	5.33

Heart Rate Variability: Frequency Domain Analysis

**Name:**	002, 002 002	**Birthdate:**	04/02/1978
**Number:**	002	**Recorded:**	05/05/2018 12:26:25
**Gender:**	Male		

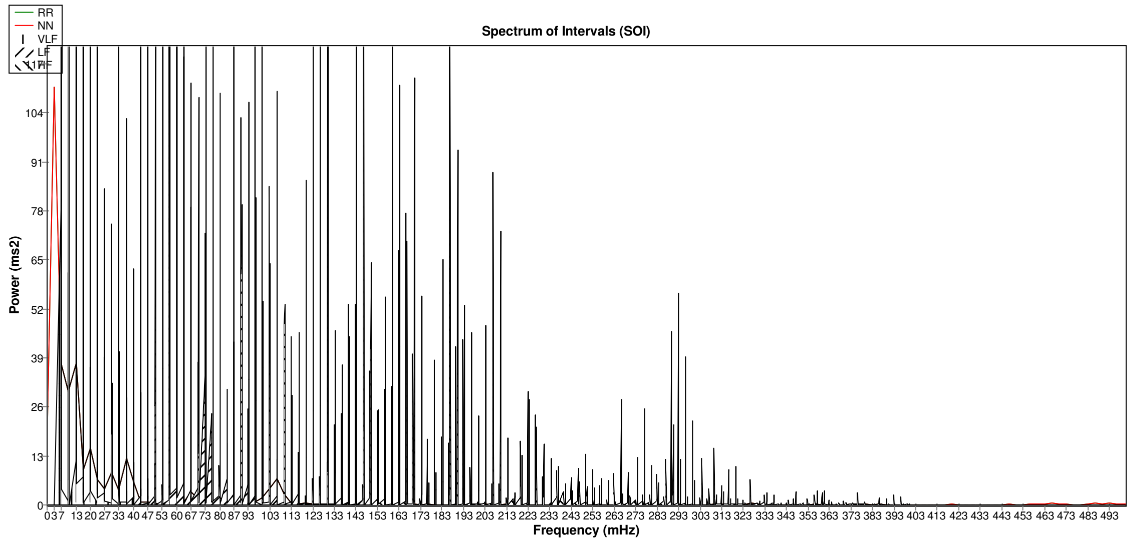

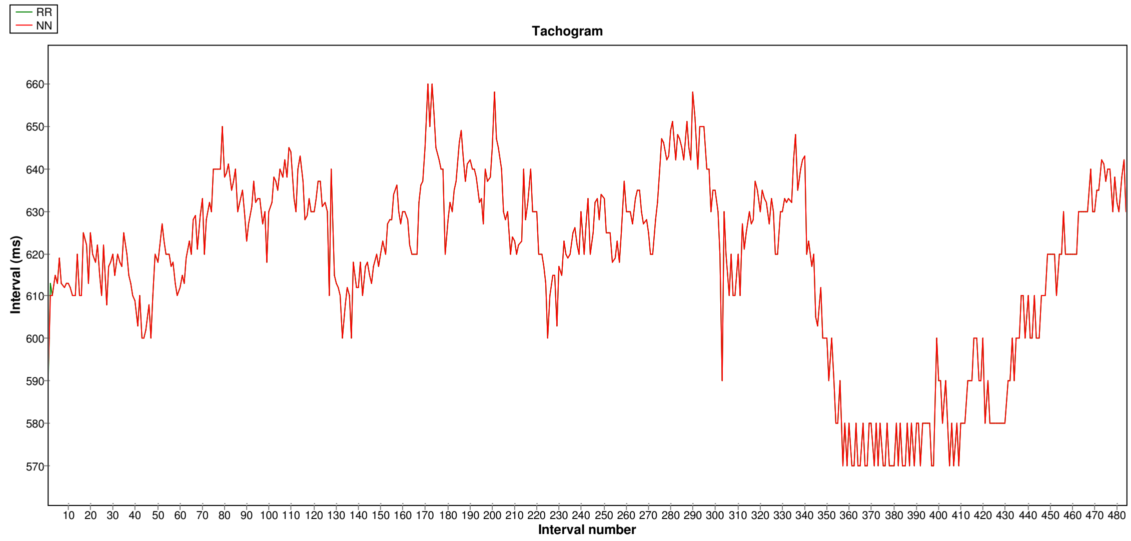

**HRV parameters**	**NN**	**RR**
TP (ms2)	214	214
VLF (ms2)	172	172
LF (ms2)	34	34
HF (ms2)	8	8
LF/HF	4.21	4.21
LF normalized	80.82	80.82
HF normalized	19.18	19.18
VLF peak (mHz)	13	13
LF peak (mHz)	107	107
HF peak (mHz)	326	326

**HRV spectral settings**	
Spectrum of Intervals (SOI)	
Frequency resolution (mHz)	3
VLF lower boundary (mHz)	3
VLF upper boundary (mHz)	40
LF upper boundary (mHz)	150
HF upper boundary (mHz)	400
Smoothing factor	1
Tapering	Hann
Fourier transform	DFT
Sample frequency (Hz)	1.62
Interval correction	Annotation
Interval threshold (%)	10
