# Supplementary material for: The effects of a 3-day mountain bike cycling race on the autonomic nervous system (ANS) and heart rate variability in amateur cyclists: a prospective quantitative research design
Source: BMC Sports Sci Med Rehabil. 2023 Jan 2;15:2. doi: 10.1186/s13102-022-00614-y (PMC9808932; doi:10.1186/s13102-022-00614-y)
Supplement: Supplementary file 1 — Additional file 1. Individual data of Participants. [file 13102_2022_614_MOESM1_ESM.zip › Individual data of Participants/HRV Data/002/ECG_002_20180506071050_.PDF]

Anton Swart Biokinetic Rehabilitation Practice

Name:	002 002 002	Recorded:	06/05/2018 07:10:50	UNCONFIRMED INTERPRETATION - MD SHOULD REVIEW
Number:	002	Recorded by:	Mr. Anton Swart	
Gender:	Male	Referring physician:		
Birthdate:	04/02/1978 40 years	Ordering physician:		
		Attending physician:		
		Location:	Anton Swart Biokinetic Rehabilitation Practi	
		Comment:		

P / PQ:	100 ms / 157 ms
QRS:	93 ms
QT / QTc / QTd:	393 ms / 418 ms / -
P/QRS/T axis:	53° / 40° / 71°
Heartrate:	74 bpm

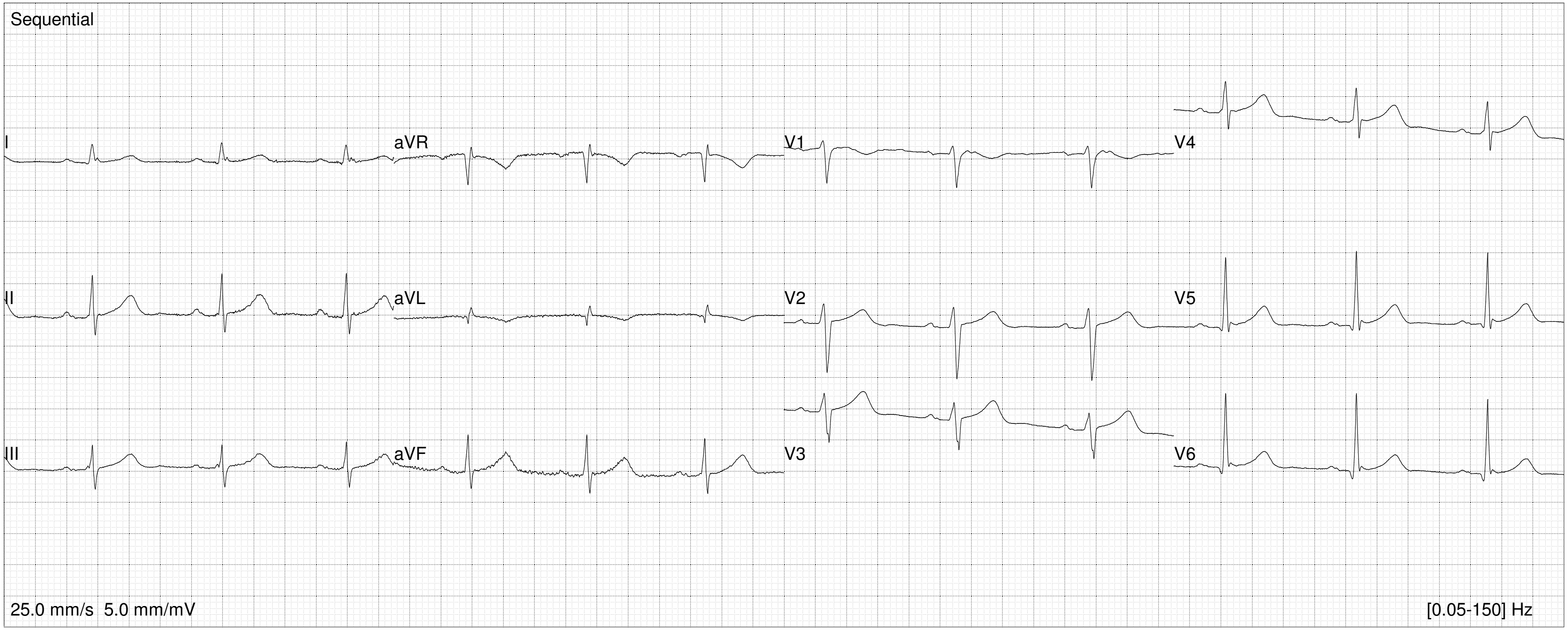

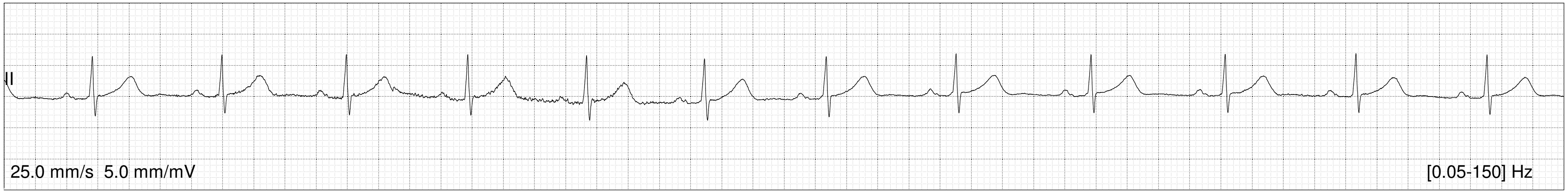

Anton Swart Biokinetic Rehabilitation Practice

Name:	002 002 002	Recorded:	06/05/2018 07:10:50	UNCONFIRMED INTERPRETATION - MD SHOULD REVIEW
Number:	002	Recorded by:	Mr. Anton Swart	
Gender:	Male	Referring physician:		
Birthdate:	04/02/1978 40 years	Location:	Anton Swart Biokinetic Rehabilitation Practice	
		Ordering physician:		
		Attending physician:		
		Comment:		

P / PQ:	100 ms / 157 ms
QRS:	93 ms
QT / QTc / QTd:	393 ms / 418 ms / -
P/QRS/T axis:	53° / 40° / 71°
Heartrate:	74 bpm

**Beats**		**RR**	
Total:	370	Minimum	690 ms
Normal:	370	Maximum	900 ms
Other:	0	Mean:	808 ms
		SD:	36 ms

R-R Trend

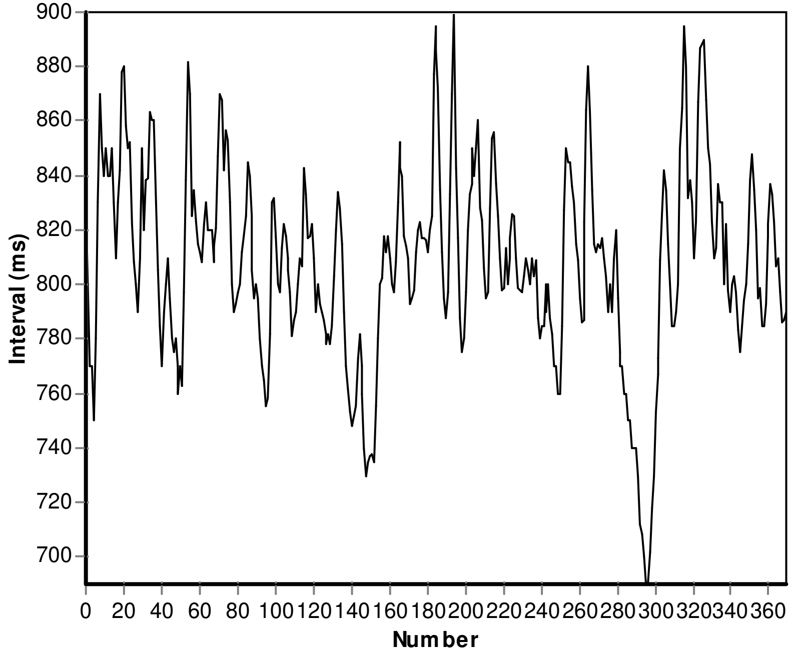

R-R Histogram

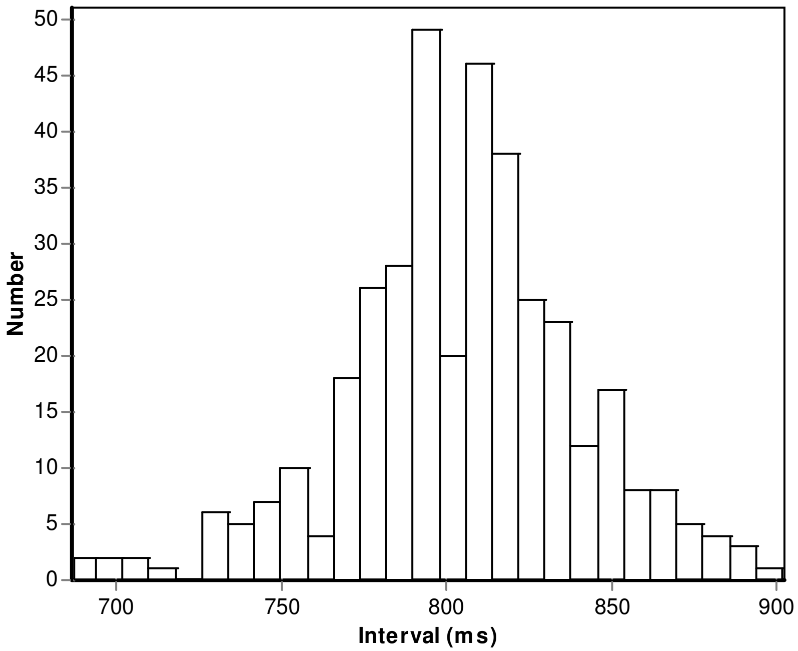

Cardio Control NV. User: Anton

Heart Rate Variability: Time Domain Analysis

**Name:**	002, 002 002	**Birthdate:**	04/02/1978
**Number:**	002	**Recorded:**	06/05/2018 07:10:50
**Gender:**	Male		

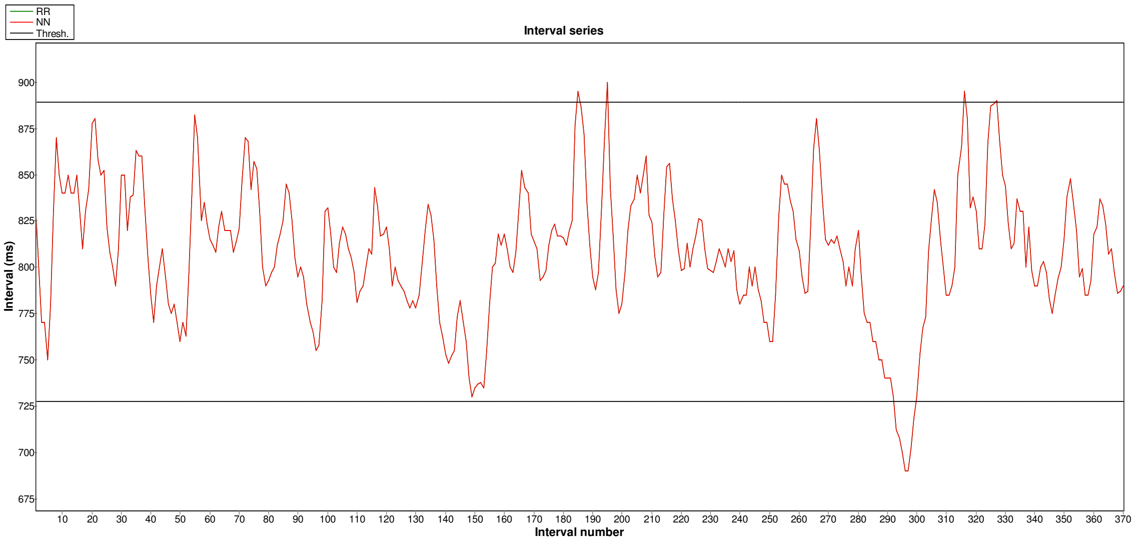

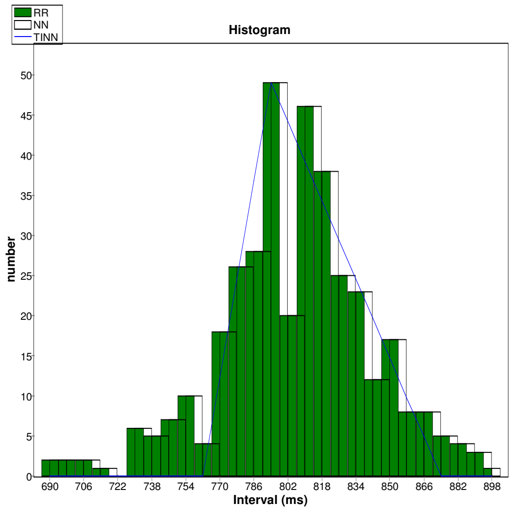

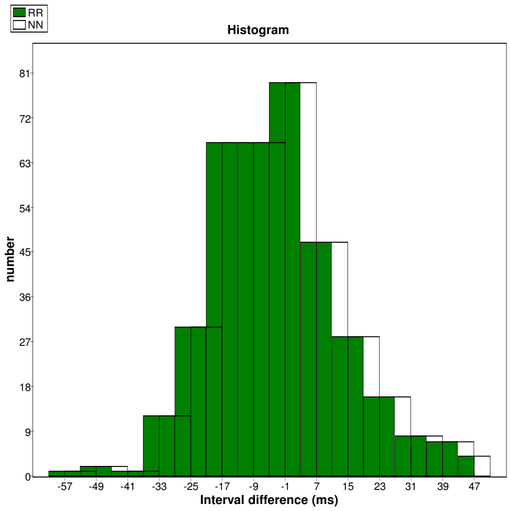

Binsize (ms) = 8

**HRV parameters**	**NN**	**RR**
SDNN (ms)	36	36
Triangular Interpolation (ms)	112	112
Triangular Index	7.55	7.55

**HRV parameters**	**NN**	**RR**
SDSD (ms)	17	17
RMSSD (ms)	17	17
NN50	2	2
NN50(1)	1	1
NN50(2)	1	1
pNN50	0.01	0.01
pNN50(1)	0.00	0.00
pNN50(2)	0.00	0.00
Logarithmic Index	0.68	0.68
SD(Logarithmic Index)	0.06	0.06

**Interval statistics**	**NN**	**RR**
Number	370	370
Minimum (ms)	690	690
Maximum (ms)	900	900
Range (ms)	210	210
Avg (ms)	808	808
SD (ms)	36	36
AvgDev (ms)	27	27
p5 (ms)	744	744
p50 (ms)	810	810
p95 (ms)	869	869
Skewness	-0.29	-0.29
Kurtosis	3.71	3.71

**Interval statistics**	**NN**	**RR**
Number	369	369
Minimum (ms)	-57	-57
Maximum (ms)	52	52
Range (ms)	109	109
Avg (ms)	-0	-0
SD (ms)	17	17
AvgDev (ms)	13	13
p5 (ms)	-25	-25
p50 (ms)	0	0
p95 (ms)	34	34
Skewness	0.41	0.41
Kurtosis	3.78	3.78

Cardio Control NV. User: Anton

Heart Rate Variability: Frequency Domain Analysis

**Name:**	002, 002 002	**Birthdate:**	04/02/1978
**Number:**	002	**Recorded:**	06/05/2018 07:10:50
**Gender:**	Male		

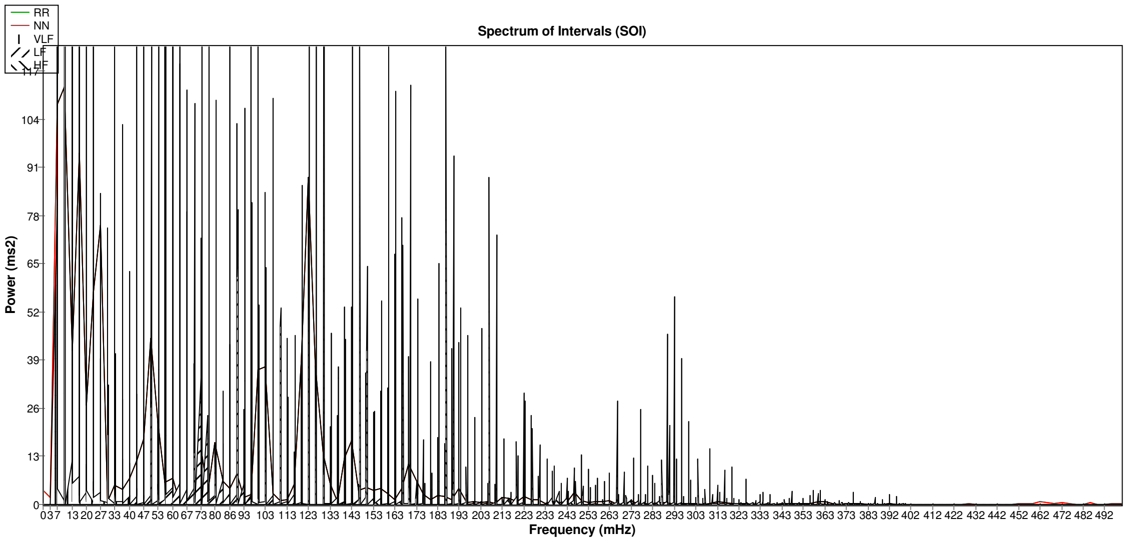

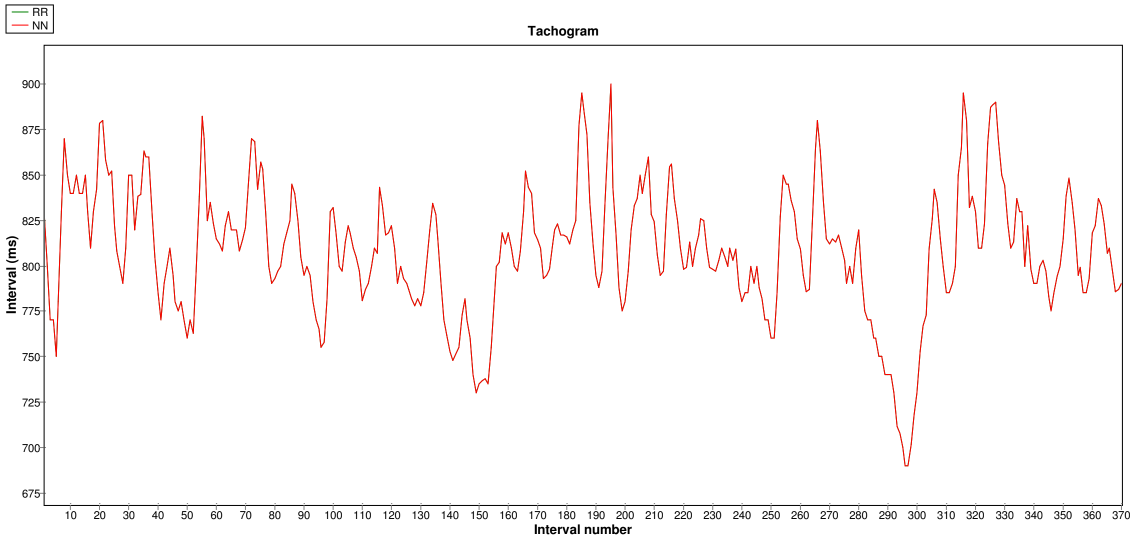

HRV parameters	NN	RR
TP (ms2)	1093	1093
VLF (ms2)	534	534
LF (ms2)	477	477
HF (ms2)	82	82
LF/HF	5.81	5.81
LF normalized	85.32	85.32
HF normalized	14.68	14.68
VLF peak (mHz)	10	10
LF peak (mHz)	123	123
HF peak (mHz)	170	170

HRV spectral settings	
Spectrum of Intervals (SOI)	
Frequency resolution (mHz)	3
VLF lower boundary (mHz)	3
VLF upper boundary (mHz)	40
LF upper boundary (mHz)	150
HF upper boundary (mHz)	400
Smoothing factor	1
Tapering	Hann
Fourier transform	DFT
Sample frequency (Hz)	1.24
Interval correction	Annotation
Interval threshold (%)	10
