# Supplementary material for: The effects of a 3-day mountain bike cycling race on the autonomic nervous system (ANS) and heart rate variability in amateur cyclists: a prospective quantitative research design
Source: BMC Sports Sci Med Rehabil. 2023 Jan 2;15:2. doi: 10.1186/s13102-022-00614-y (PMC9808932; doi:10.1186/s13102-022-00614-y)
Supplement: Supplementary file 1 — Additional file 1. Individual data of Participants. [file 13102_2022_614_MOESM1_ESM.zip › Individual data of Participants/HRV Data/003/ECG_003_20180501072219_.PDF]

Anton Swart Biokinetic Rehabilitation Practice

Name:	003 003 003	Recorded:	01/05/2018 07:22:19	UNCONFIRMED INTERPRETATION - MD SHOULD REVIEW
Number:	003	Recorded by:	Mr. Anton Swart	
Gender:	Male	Referring physician:		
Birthdate:	26/01/1958 60 years	Ordering physician:		
		Attending physician:		
P / PQ:	132 ms / 168 ms	Location:	Anton Swart Biokinetic Rehabilitation Practi	
QRS:	90 ms	Comment:		
QT / QTc / QTd:	422 ms / 427 ms / -			
P/QRS/T axis:	71° / 81° / 55°			
Heartrate:	63 bpm			

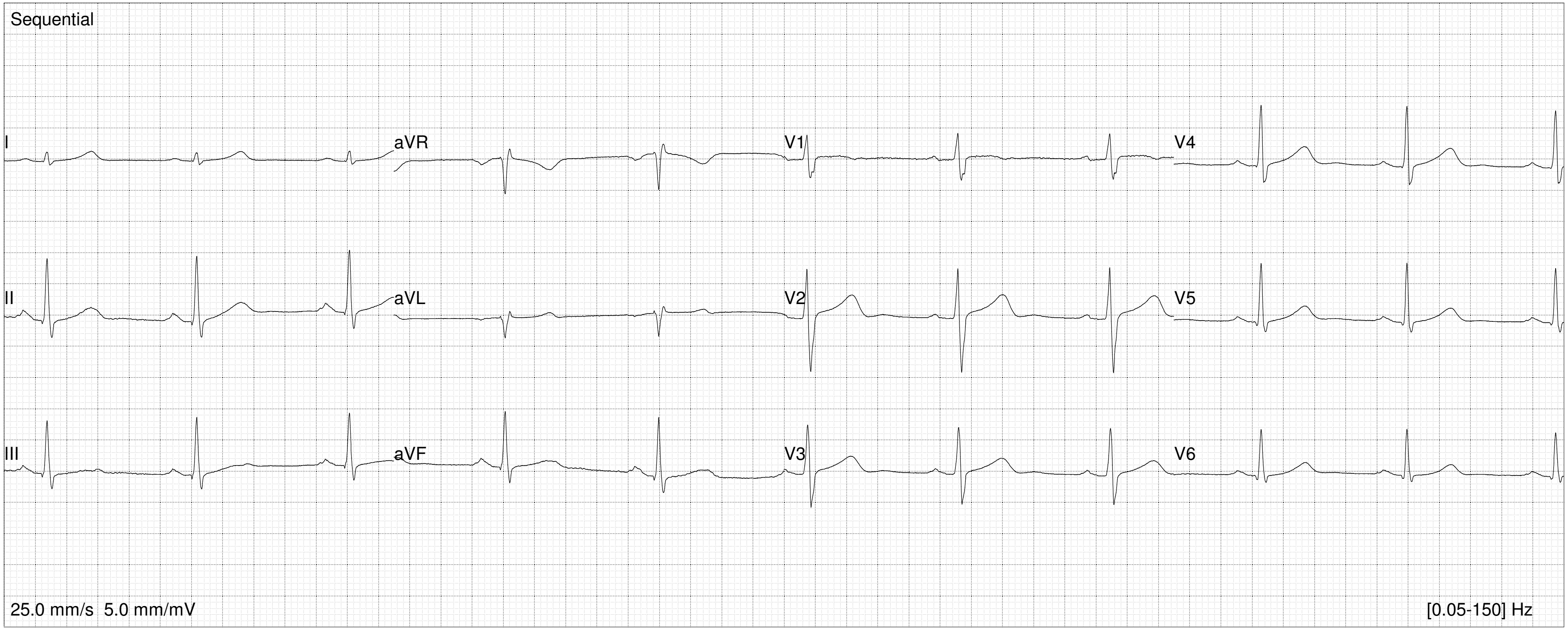

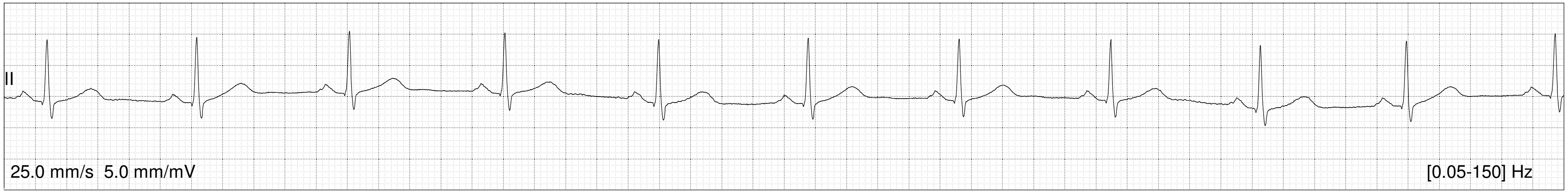

Anton Swart Biokinetic Rehabilitation Practice

Name:	003 003 003	Recorded:	01/05/2018 07:22:19	UNCONFIRMED INTERPRETATION - MD SHOULD REVIEW
Number:	003	Recorded by:	Mr. Anton Swart	
Gender:	Male	Referring physician:		
Birthdate:	26/01/1958 60 years	Location:	Anton Swart Biokinetic Rehabilitation Practice	
		Ordering physician:		
		Attending physician:		
		Comment:		

P / PQ:	132 ms / 168 ms
QRS:	90 ms
QT / QTc / QTd:	422 ms / 427 ms / -
P/QRS/T axis:	71° / 81° / 55°
Heartrate:	63 bpm

Beats

Total:	315
Normal:	315
Other:	0

RR

Minimum	860 ms
Maximum	1040 ms
Mean:	950 ms
SD:	26 ms

R-R Trend

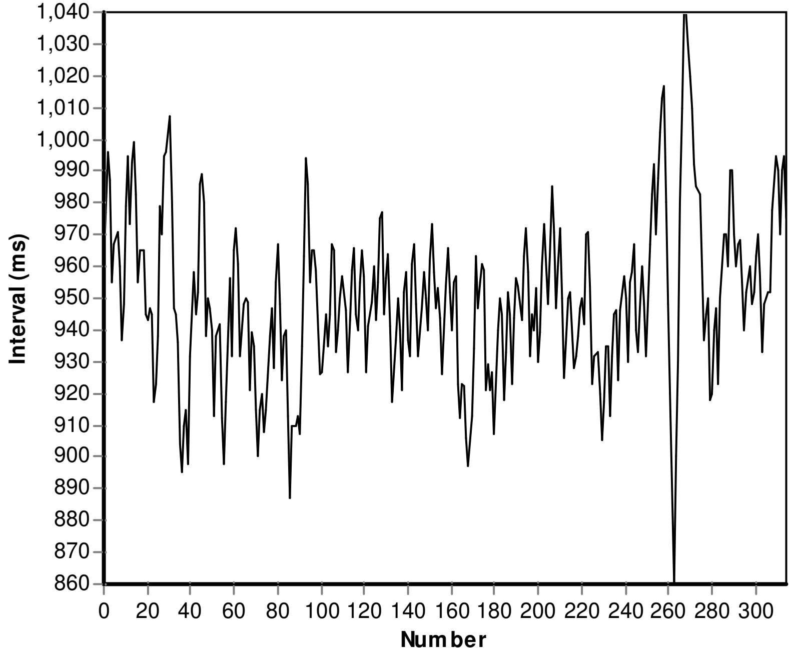

R-R Histogram

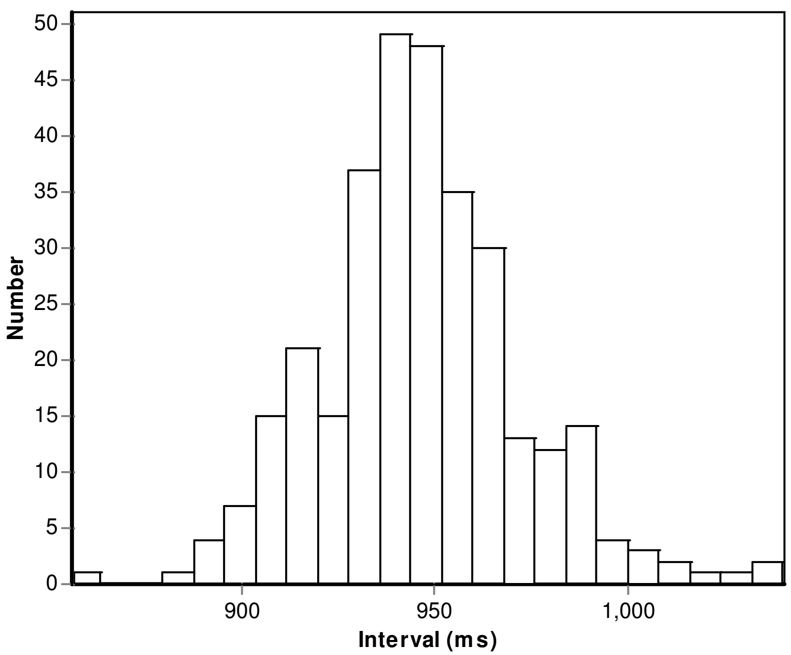

Heart Rate Variability: Time Domain Analysis

**Name:**	003, 003 003	**Birthdate:**	26/01/1958
**Number:**	003	**Recorded:**	01/05/2018 07:22:19
**Gender:**	Male		

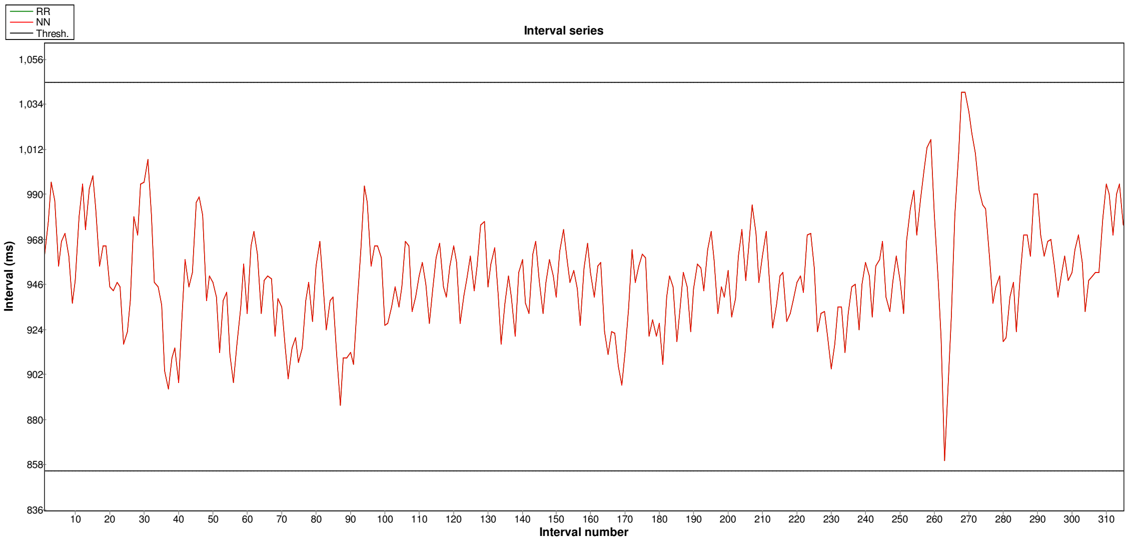

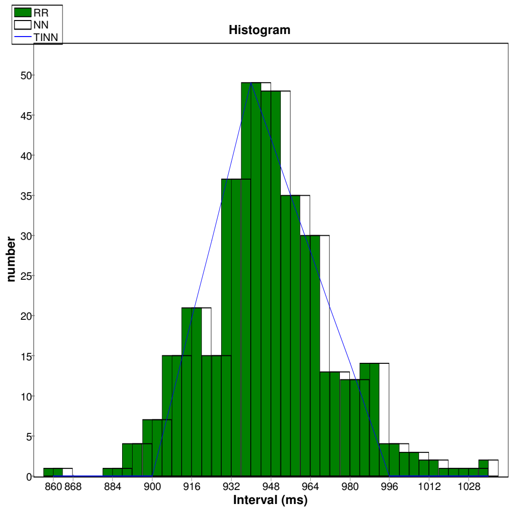

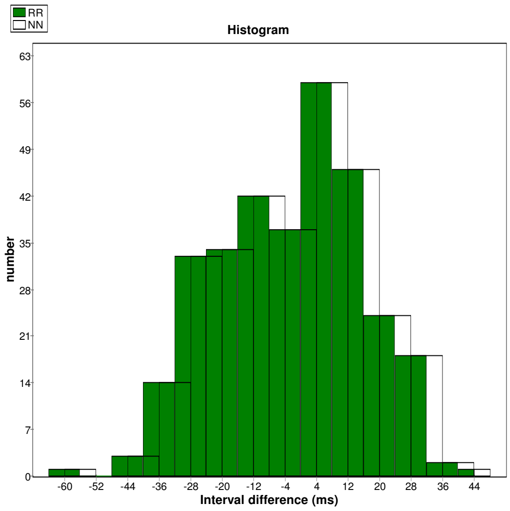

Binsize (ms) = 8

**HRV parameters**	**NN**	**RR**
SDNN (ms)	26	26
Triangular Interpolation (ms)	96	96
Triangular Index	6.43	6.43

**HRV parameters**	**NN**	**RR**
SDSD (ms)	18	18
RMSSD (ms)	18	18
NN50	1	1
NN50(1)	1	1
NN50(2)	0	0
pNN50	0.00	0.00
pNN50(1)	0.00	0.00
pNN50(2)	0.00	0.00
Logarithmic Index	0.76	0.76
SD(Logarithmic Index)	0.10	0.10

**Interval statistics**	**NN**	**RR**
Number	315	315
Minimum (ms)	860	860
Maximum (ms)	1040	1040
Range (ms)	180	180
Avg (ms)	950	950
SD (ms)	26	26
AvgDev (ms)	20	20
p5 (ms)	910	910
p50 (ms)	948	948
p95 (ms)	995	995
Skewness	0.35	0.35
Kurtosis	3.93	3.93

**Interval statistics**	**NN**	**RR**
Number	314	314
Minimum (ms)	-60	-60
Maximum (ms)	50	50
Range (ms)	110	110
Avg (ms)	0	0
SD (ms)	18	18
AvgDev (ms)	15	15
p5 (ms)	-31	-31
p50 (ms)	2	2
p95 (ms)	30	30
Skewness	-0.15	-0.15
Kurtosis	2.50	2.50

Cardio Control NV. User: Anton

Heart Rate Variability: Frequency Domain Analysis

**Name:**	003, 003 003	**Birthdate:**	26/01/1958
**Number:**	003	**Recorded:**	01/05/2018 07:22:19
**Gender:**	Male		

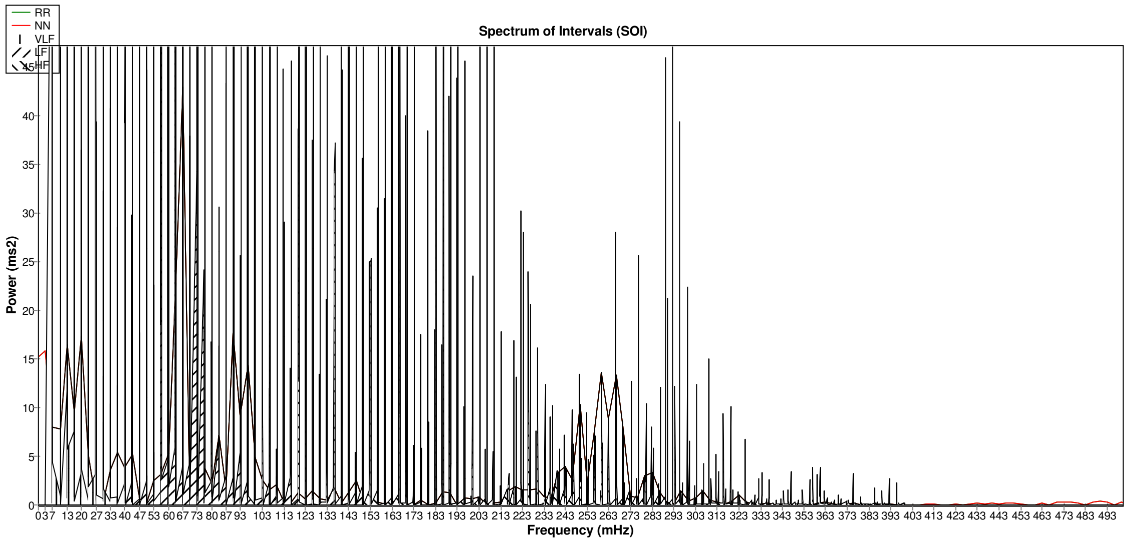

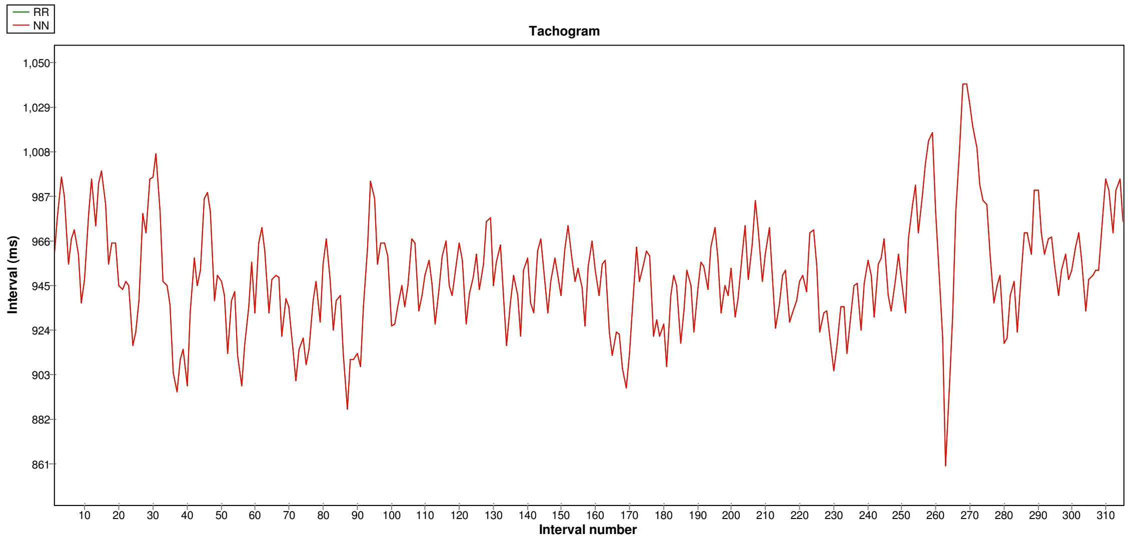

HRV parameters	NN	RR
TP (ms2)	347	347
VLF (ms2)	78	78
LF (ms2)	161	161
HF (ms2)	109	109
LF/HF	1.48	1.48
LF normalized	59.66	59.66
HF normalized	40.34	40.34
VLF peak (mHz)	20	20
LF peak (mHz)	67	67
HF peak (mHz)	260	260

HRV spectral settings	
Spectrum of Intervals (SOI)	
Frequency resolution (mHz)	3
VLF lower boundary (mHz)	3
VLF upper boundary (mHz)	40
LF upper boundary (mHz)	150
HF upper boundary (mHz)	400
Smoothing factor	1
Tapering	Hann
Fourier transform	DFT
Sample frequency (Hz)	1.05
Interval correction	Annotation
Interval threshold (%)	10
