# Supplementary material for: The effects of a 3-day mountain bike cycling race on the autonomic nervous system (ANS) and heart rate variability in amateur cyclists: a prospective quantitative research design
Source: BMC Sports Sci Med Rehabil. 2023 Jan 2;15:2. doi: 10.1186/s13102-022-00614-y (PMC9808932; doi:10.1186/s13102-022-00614-y)
Supplement: Supplementary file 1 — Additional file 1. Individual data of Participants. [file 13102_2022_614_MOESM1_ESM.zip › Individual data of Participants/HRV Data/003/ECG_003_20180503174516_.PDF]

Anton Swart Biokinetic Rehabilitation Practice

Name: 003 003 003
Number: 003
Gender: Male
Birthdate: 26/01/1958 60 years

P / PQ: 133 ms / 162 ms
QRS: 90 ms
QT / QTc / QTd: 398 ms / 426 ms / -
P/QRS/T axis: 80° / 84° / 63°
Heartrate: 76 bpm

Recorded: 03/05/2018 17:45:16
Recorded by: Mr. Anton Swart
Referring physician:
Ordering physician:
Attending physician:
Location: Anton Swart Biokinetic Rehabilitation Practi
Comment:

UNCONFIRMED INTERPRETATION - MD SHOULD REVIEW

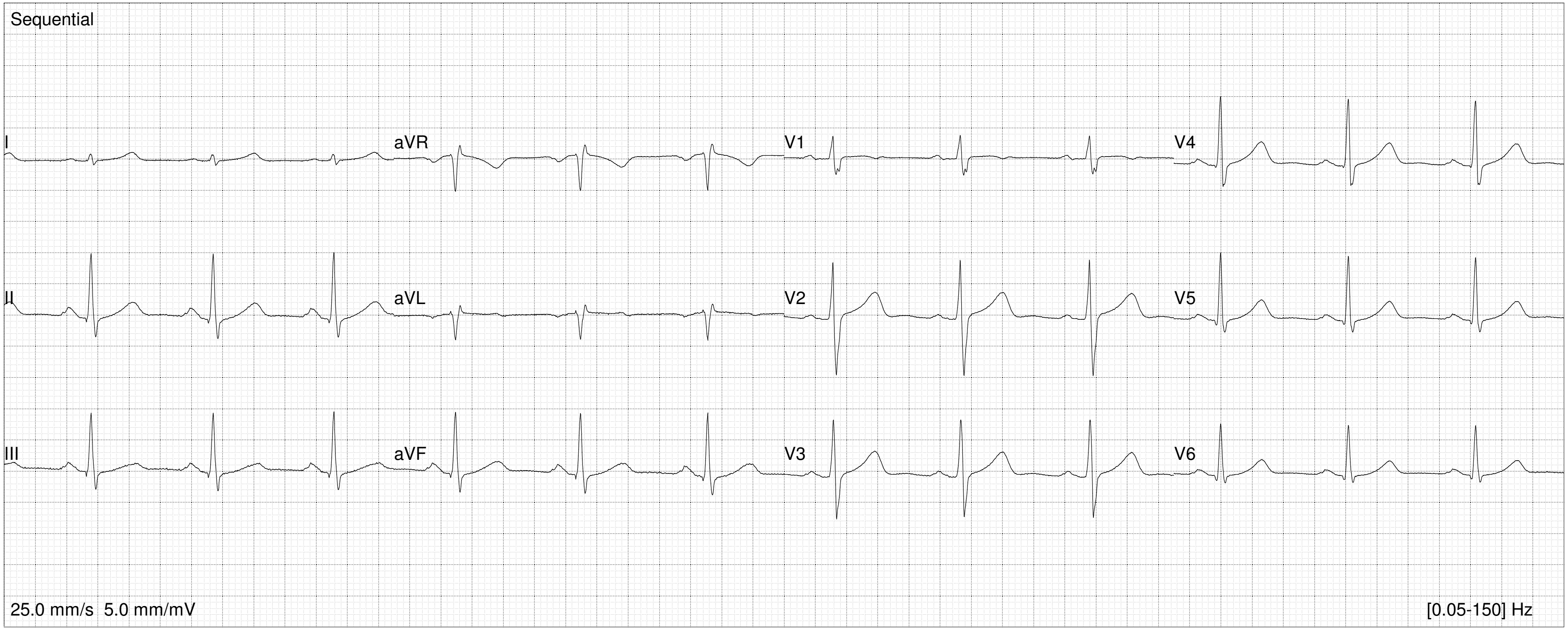

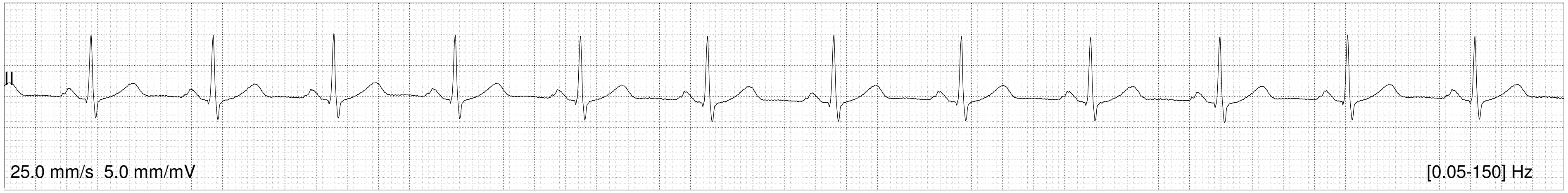

Anton Swart Biokinetic Rehabilitation Practice

Name:	003 003 003	Recorded:	03/05/2018 17:45:16	UNCONFIRMED INTERPRETATION - MD SHOULD REVIEW
Number:	003	Recorded by:	Mr. Anton Swart	
Gender:	Male	Referring physician:		
Birthdate:	26/01/1958 60 years	Location:	Anton Swart Biokinetic Rehabilitation Practice	
		Ordering physician:		
		Attending physician:		
		Comment:		

P / PQ:	133 ms / 162 ms
QRS:	90 ms
QT / QTc / QTd:	398 ms / 426 ms / -
P/QRS/T axis:	80° / 84° / 63°
Heartrate:	76 bpm

Beats

Total:	378
Normal:	378
Other:	0

RR

Minimum	737 ms
Maximum	858 ms
Mean:	791 ms
SD:	18 ms

R-R Trend

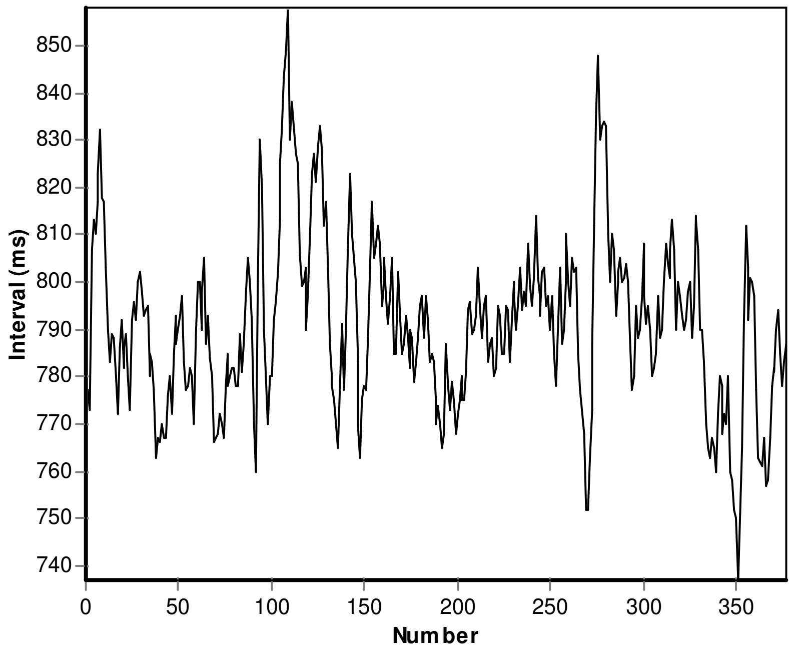

R-R Histogram

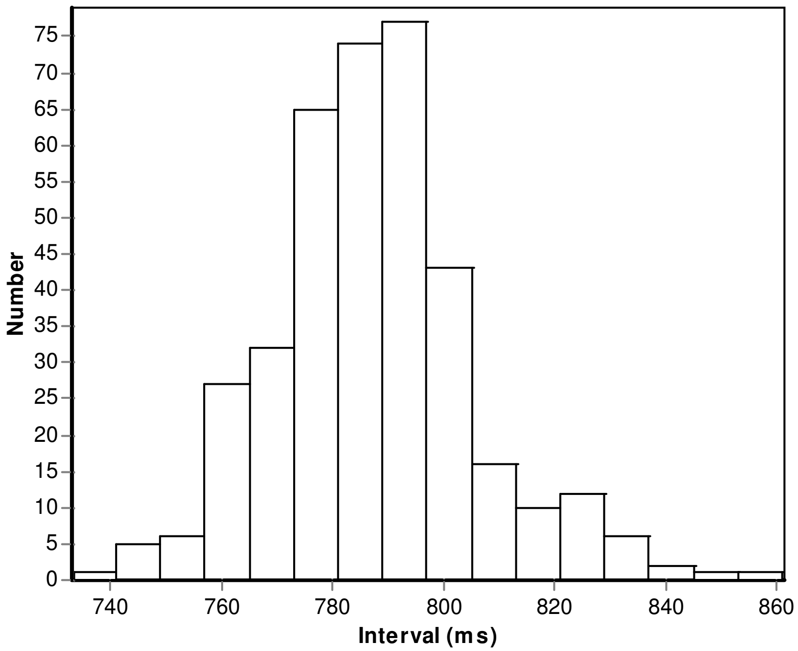

Heart Rate Variability: Time Domain Analysis

**Name:**	003, 003 003	**Birthdate:**	26/01/1958
**Number:**	003	**Recorded:**	03/05/2018 17:45:16
**Gender:**	Male		

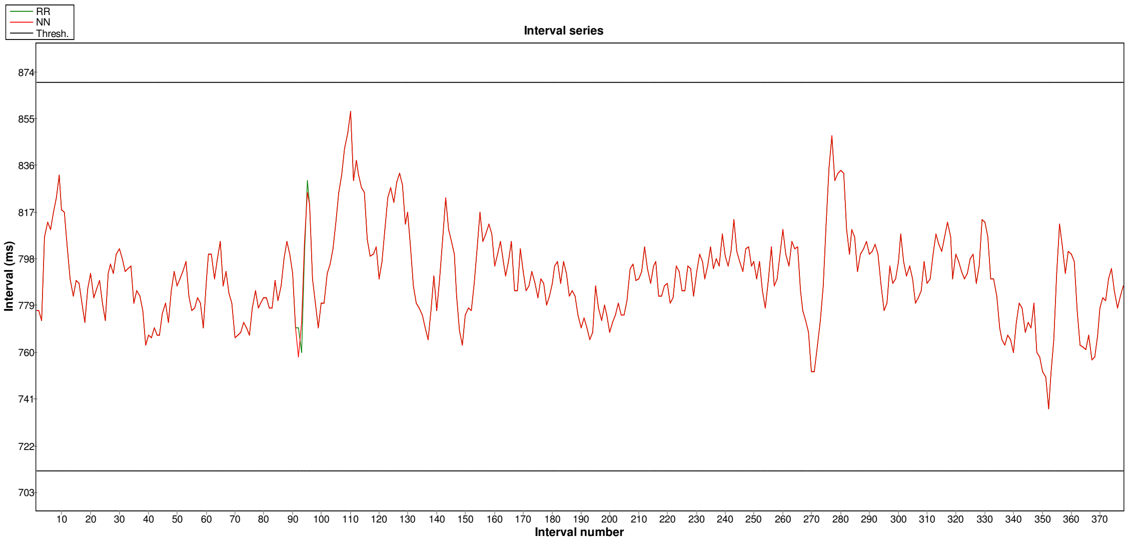

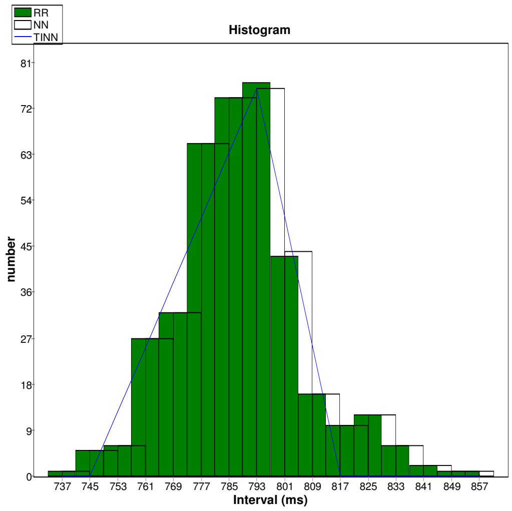

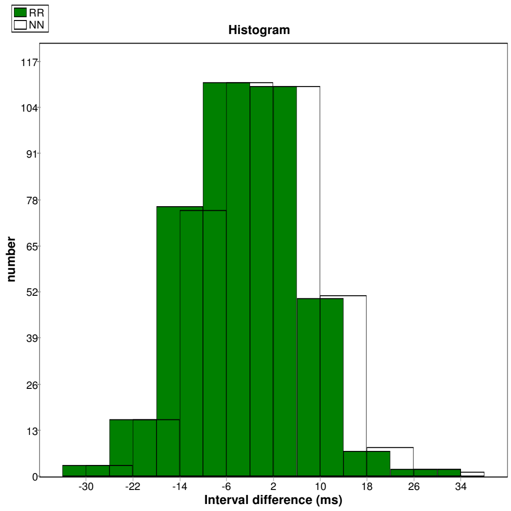

Binsize (ms) = 8

**HRV parameters**	**NN**	**RR**
SDNN (ms)	18	18
Triangular Interpolation (ms)	72	72
Triangular Index	4.97	4.91

**HRV parameters**	**NN**	**RR**
SDSD (ms)	10	10
RMSSD (ms)	10	10
NN50	0	0
NN50(1)	0	0
NN50(2)	0	0
pNN50	0.00	0.00
pNN50(1)	0.00	0.00
pNN50(2)	0.00	0.00
Logarithmic Index	1.28	1.23
SD(Logarithmic Index)	0.13	0.13

**Interval statistics**	**NN**	**RR**
Number	378	378
Minimum (ms)	737	737
Maximum (ms)	858	858
Range (ms)	121	121
Avg (ms)	791	791
SD (ms)	18	18
AvgDev (ms)	14	14
p5 (ms)	763	763
p50 (ms)	790	790
p95 (ms)	827	828
Skewness	0.49	0.50
Kurtosis	3.81	3.82

**Interval statistics**	**NN**	**RR**
Number	377	377
Minimum (ms)	-30	-30
Maximum (ms)	34	40
Range (ms)	64	70
Avg (ms)	0	0
SD (ms)	10	10
AvgDev (ms)	8	8
p5 (ms)	-15	-15
p50 (ms)	0	0
p95 (ms)	14	14
Skewness	0.11	0.23
Kurtosis	3.40	3.84

Cardio Control NV. User: Anton

Heart Rate Variability: Frequency Domain Analysis

**Name:**	003, 003 003	**Birthdate:**	26/01/1958
**Number:**	003	**Recorded:**	03/05/2018 17:45:16
**Gender:**	Male		

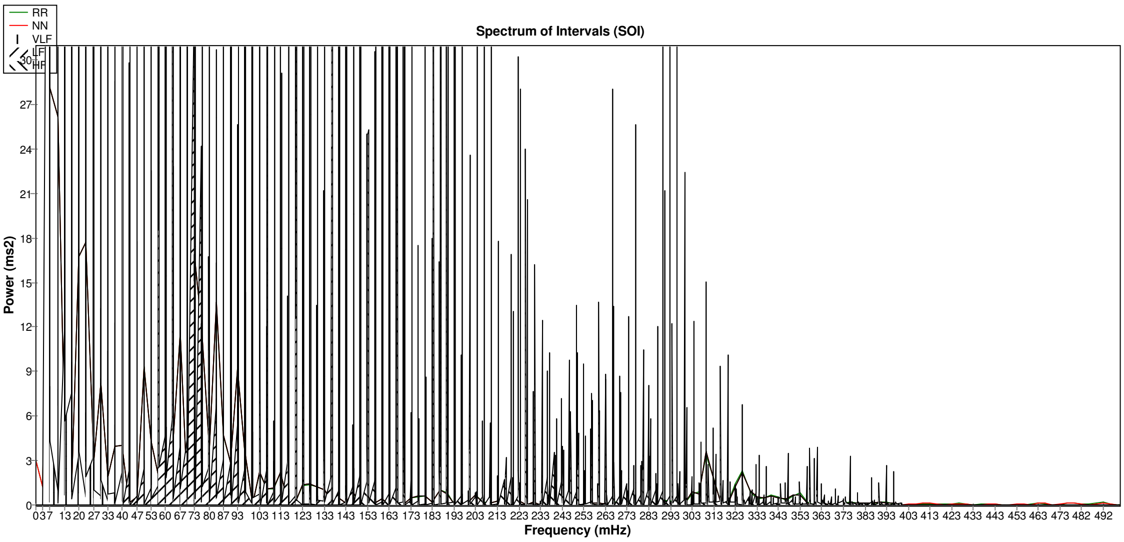

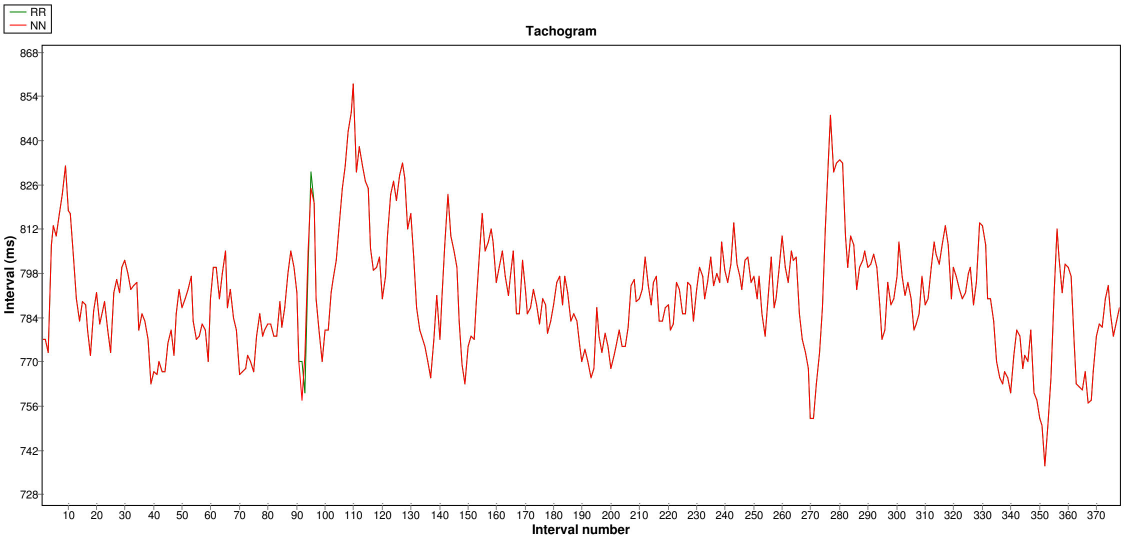

**HRV parameters**	**NN**	**RR**
TP (ms2)	259	259
VLF (ms2)	116	116
LF (ms2)	116	116
HF (ms2)	27	27
LF/HF	4.27	4.22
LF normalized	81.03	80.86
HF normalized	18.97	19.14
VLF peak (mHz)	7	7
LF peak (mHz)	73	73
HF peak (mHz)	309	309

**HRV spectral settings**	
Spectrum of Intervals (SOI)	
Frequency resolution (mHz)	3
VLF lower boundary (mHz)	3
VLF upper boundary (mHz)	40
LF upper boundary (mHz)	150
HF upper boundary (mHz)	400
Smoothing factor	1
Tapering	Hann
Fourier transform	DFT
Sample frequency (Hz)	1.26
Interval correction	Annotation
Interval threshold (%)	10
