# Supplementary material for: The effects of a 3-day mountain bike cycling race on the autonomic nervous system (ANS) and heart rate variability in amateur cyclists: a prospective quantitative research design
Source: BMC Sports Sci Med Rehabil. 2023 Jan 2;15:2. doi: 10.1186/s13102-022-00614-y (PMC9808932; doi:10.1186/s13102-022-00614-y)
Supplement: Supplementary file 1 — Additional file 1. Individual data of Participants. [file 13102_2022_614_MOESM1_ESM.zip › Individual data of Participants/HRV Data/003/ECG_003_20180504140631_.PDF]

Anton Swart Biokinetic Rehabilitation Practice

UNCONFIRMED INTERPRETATION - MD SHOULD REVIEW

Name:	003 003 003
Number:	003
Gender:	Male
Birthdate:	26/01/1958 60 years

Recorded:	04/05/2018 14:06:31
Recorded by:	Mr. Anton Swart
Referring physician:	
Ordering physician:	
Attending physician:	
Location:	Anton Swart Biokinetic Rehabilitation Practi
Comment:	

P / PQ:	127 ms / 150 ms
QRS:	90 ms
QT / QTc / QTd:	380 ms / 440 ms / -
P/QRS/T axis:	75° / 86° / 63°
Heartrate:	94 bpm

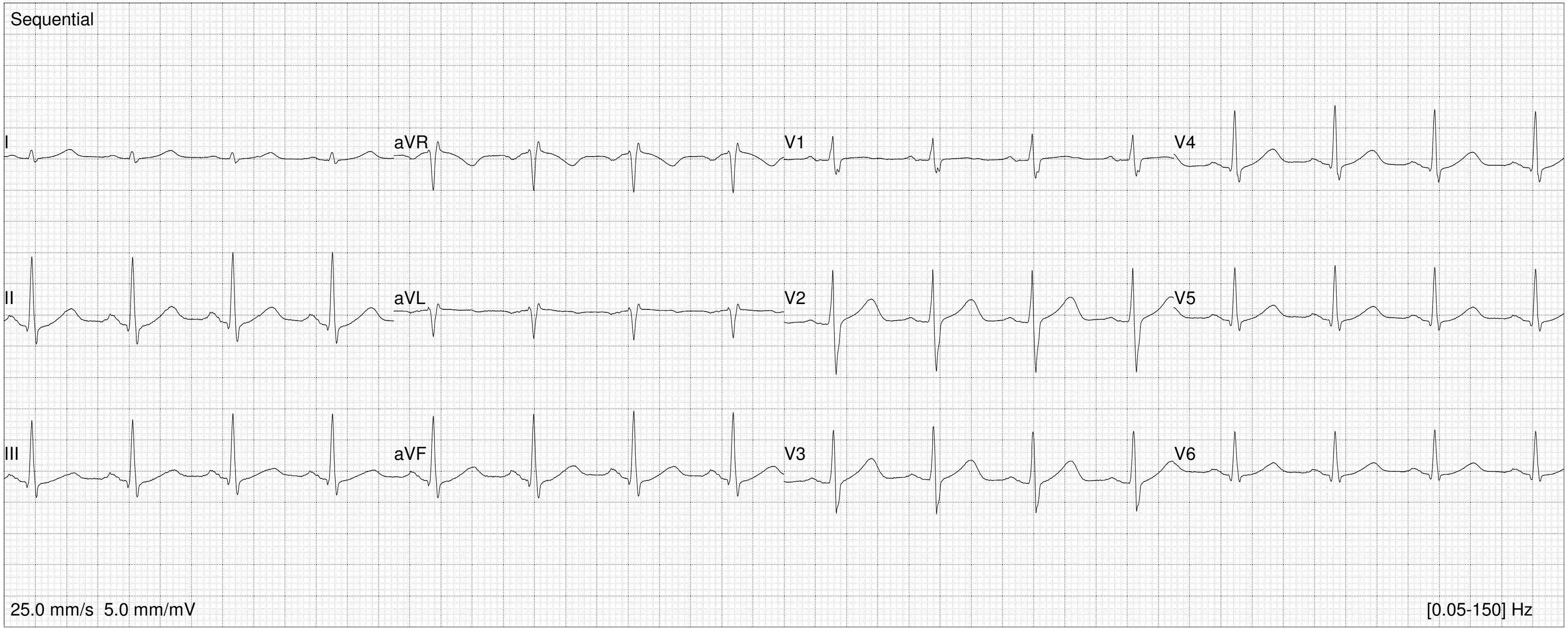

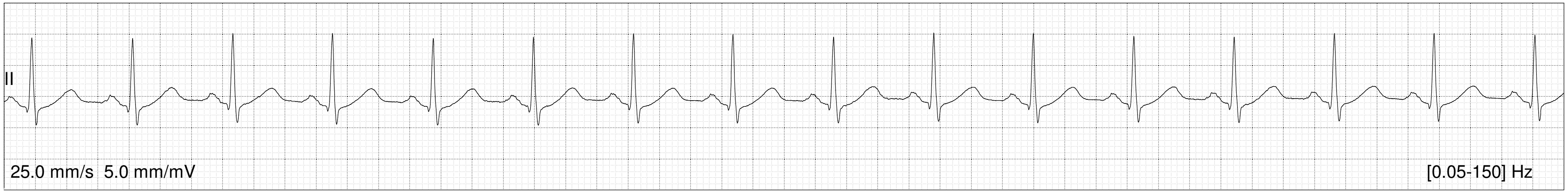

Anton Swart Biokinetic Rehabilitation Practice

Name:	003 003 003	Recorded:	04/05/2018 14:06:31	UNCONFIRMED INTERPRETATION - MD SHOULD REVIEW
Number:	003	Recorded by:	Mr. Anton Swart	
Gender:	Male	Referring physician:		
Birthdate:	26/01/1958 60 years	Location:	Anton Swart Biokinetic Rehabilitation Practice	
P / PQ:	127 ms / 150 ms	Ordering physician:		
QRS:	90 ms	Attending physician:		
QT / QTc / QTd:	380 ms / 440 ms / -	Comment:		
P/QRS/T axis:	75° / 86° / 63°			
Heartrate:	94 bpm			

**Beats**		**RR**	
Total:	469	Minimum	617 ms
Normal:	469	Maximum	657 ms
Other:	0	Mean:	639 ms
		SD:	7 ms

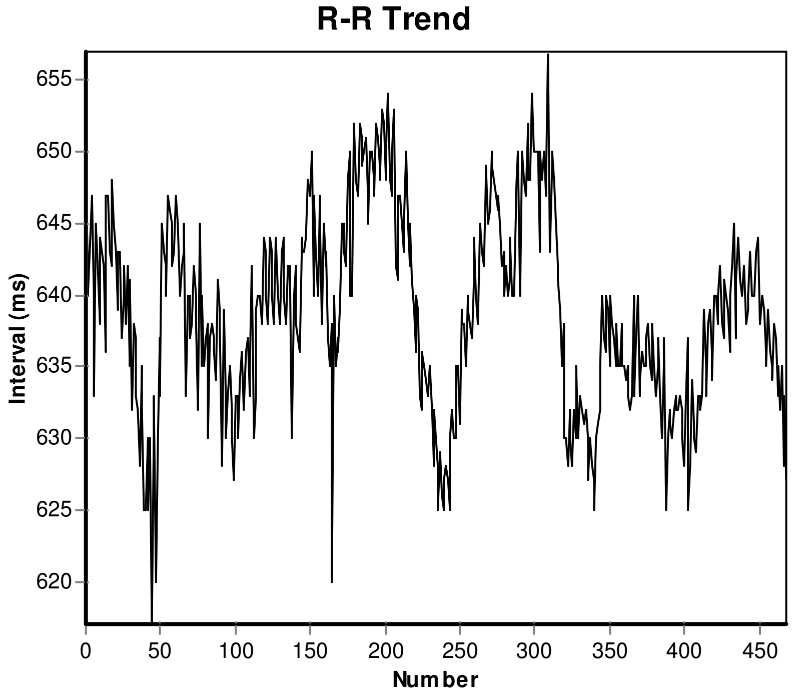

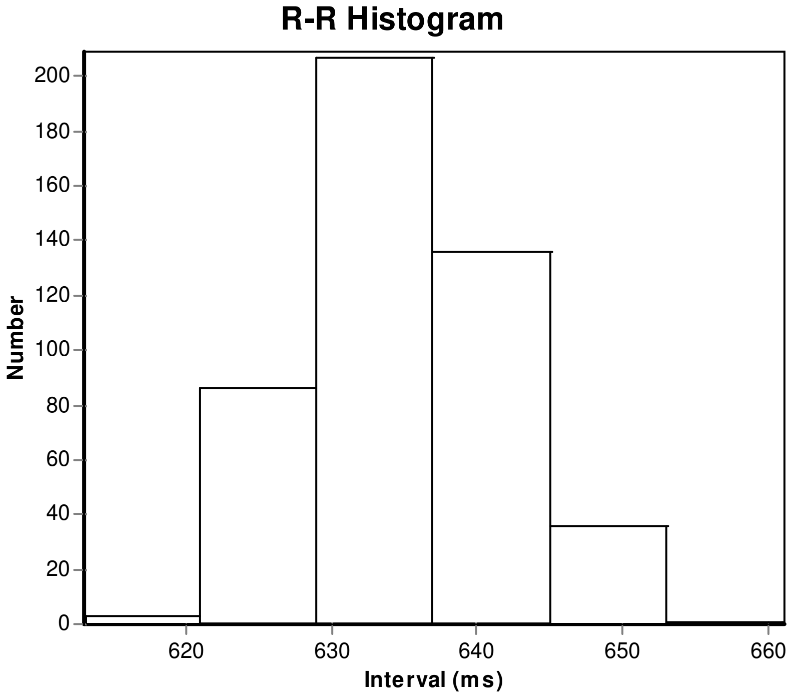

Heart Rate Variability: Time Domain Analysis

**Name:**	003, 003 003	**Birthdate:**	26/01/1958
**Number:**	003	**Recorded:**	04/05/2018 14:06:31
**Gender:**	Male		

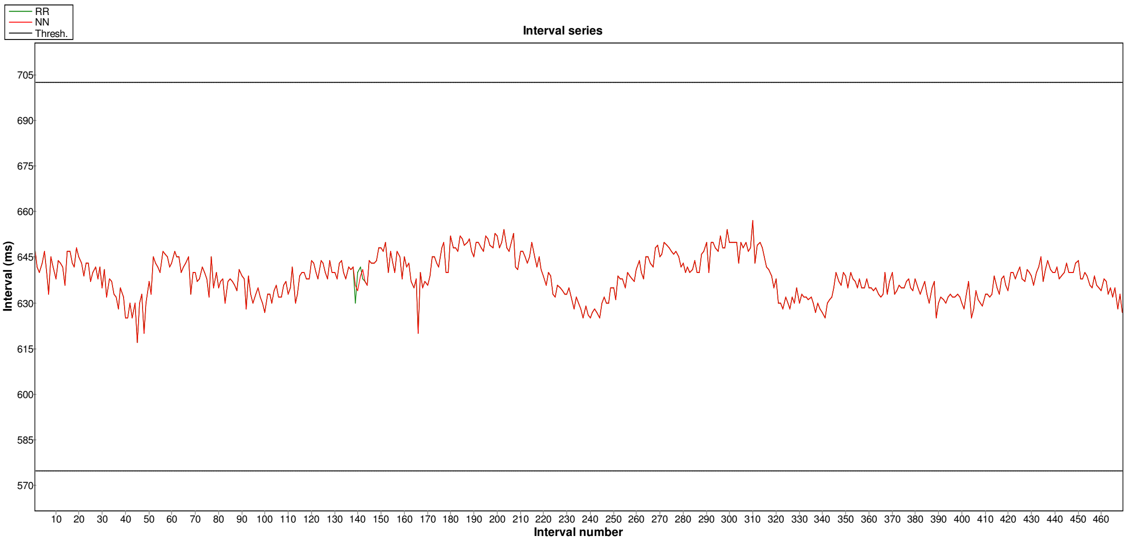

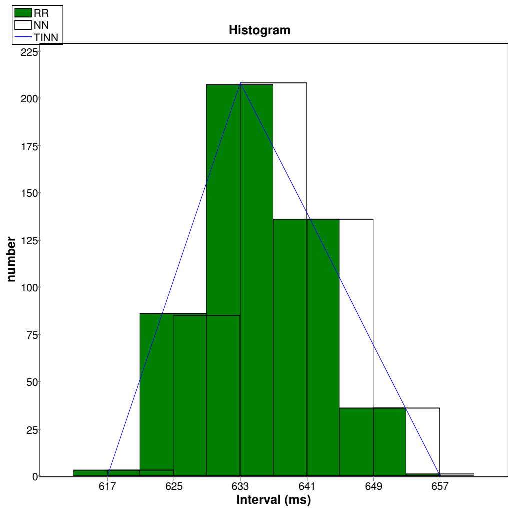

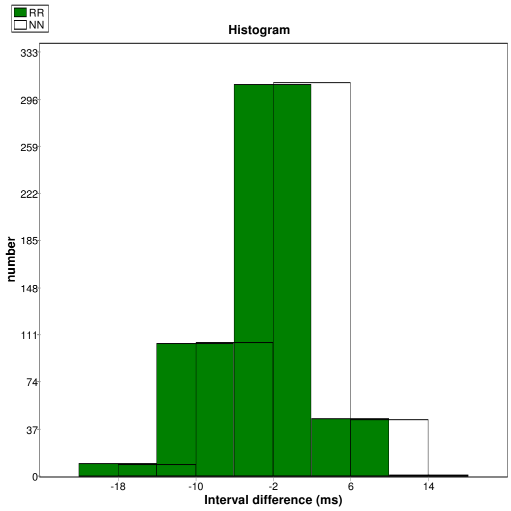

Binsize (ms) = 8

**HRV parameters**	**NN**	**RR**
SDNN (ms)	7	7
Triangular Interpolation (ms)	40	40
Triangular Index	2.25	2.27

**HRV parameters**	**NN**	**RR**
SDSD (ms)	4	4
RMSSD (ms)	4	4
NN50	0	0
NN50(1)	0	0
NN50(2)	0	0
pNN50	0.00	0.00
pNN50(1)	0.00	0.00
pNN50(2)	0.00	0.00
Logarithmic Index	2.69	2.69
SD(Logarithmic Index)	0.01	0.03

**Interval statistics**	**NN**	**RR**
Number	469	469
Minimum (ms)	617	617
Maximum (ms)	657	657
Range (ms)	40	40
Avg (ms)	639	639
SD (ms)	7	7
AvgDev (ms)	5	5
p5 (ms)	628	628
p50 (ms)	639	639
p95 (ms)	650	650
Skewness	-0.02	-0.02
Kurtosis	2.63	2.63

**Interval statistics**	**NN**	**RR**
Number	468	468
Minimum (ms)	-18	-18
Maximum (ms)	20	20
Range (ms)	38	38
Avg (ms)	-0	-0
SD (ms)	4	4
AvgDev (ms)	3	3
p5 (ms)	-7	-7
p50 (ms)	-1	-1
p95 (ms)	7	7
Skewness	0.03	0.01
Kurtosis	4.85	4.82

Heart Rate Variability: Frequency Domain Analysis

**Name:**	003, 003 003	**Birthdate:**	26/01/1958
**Number:**	003	**Recorded:**	04/05/2018 14:06:31
**Gender:**	Male		

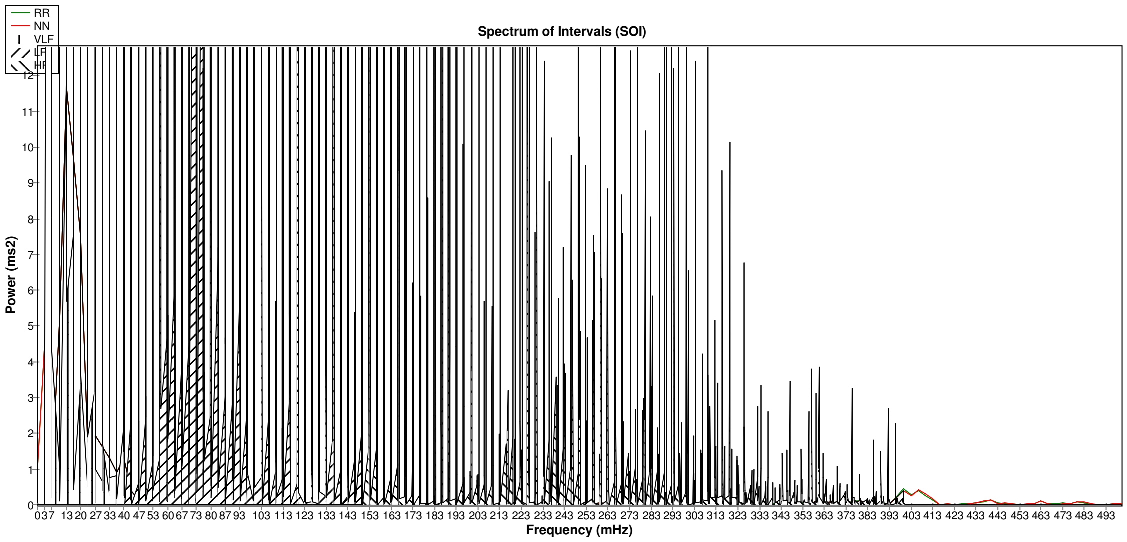

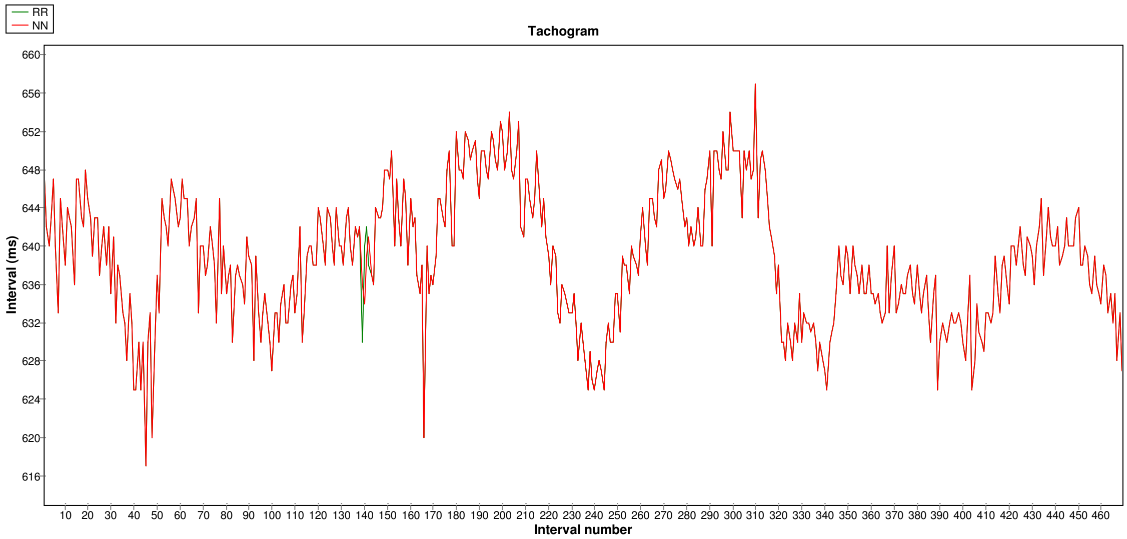

**HRV parameters**	**NN**	**RR**
TP (ms2)	50	50
VLF (ms2)	44	44
LF (ms2)	5	5
HF (ms2)	2	2
LF/HF	2.27	2.10
LF normalized	69.40	67.76
HF normalized	30.60	32.24
VLF peak (mHz)	13	13
LF peak (mHz)	80	80
HF peak (mHz)	400	400

**HRV spectral settings**	
Spectrum of Intervals (SOI)	
Frequency resolution (mHz)	3
VLF lower boundary (mHz)	3
VLF upper boundary (mHz)	40
LF upper boundary (mHz)	150
HF upper boundary (mHz)	400
Smoothing factor	1
Tapering	Hann
Fourier transform	DFT
Sample frequency (Hz)	1.57
Interval correction	Annotation
Interval threshold (%)	10
