# Supplementary material for: The effects of a 3-day mountain bike cycling race on the autonomic nervous system (ANS) and heart rate variability in amateur cyclists: a prospective quantitative research design
Source: BMC Sports Sci Med Rehabil. 2023 Jan 2;15:2. doi: 10.1186/s13102-022-00614-y (PMC9808932; doi:10.1186/s13102-022-00614-y)
Supplement: Supplementary file 1 — Additional file 1. Individual data of Participants. [file 13102_2022_614_MOESM1_ESM.zip › Individual data of Participants/HRV Data/003/ECG_003_20180505140153_.PDF]

Anton Swart Biokinetic Rehabilitation Practice

Name:	003 003 003	Recorded:	05/05/2018 14:01:53	UNCONFIRMED INTERPRETATION - MD SHOULD REVIEW
Number:	003	Recorded by:	Mr. Anton Swart	
Gender:	Male	Referring physician:		
Birthdate:	26/01/1958 60 years	Ordering physician:		
		Attending physician:		
		Location:	Anton Swart Biokinetic Rehabilitation Practi	
		Comment:		

P / PQ:	128 ms / 150 ms
QRS:	88 ms
QT / QTc / QTd:	392 ms / 436 ms / -
P/QRS/T axis:	76° / 83° / 62°
Heartrate:	85 bpm

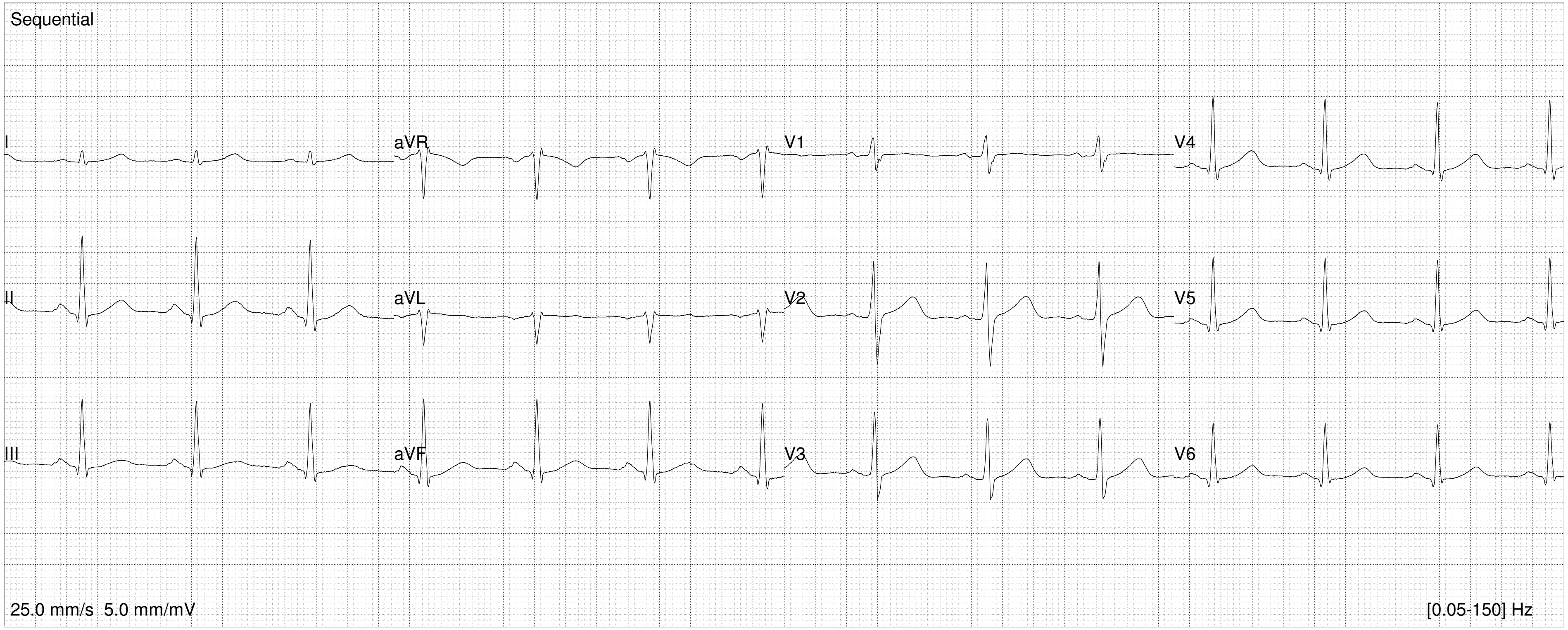

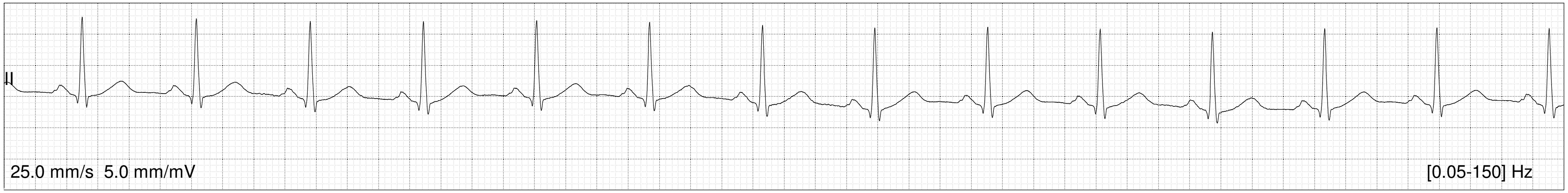

Anton Swart Biokinetic Rehabilitation Practice

Name:	003 003 003	Recorded:	05/05/2018 14:01:53	UNCONFIRMED INTERPRETATION - MD SHOULD REVIEW
Number:	003	Recorded by:	Mr. Anton Swart	
Gender:	Male	Referring physician:		
Birthdate:	26/01/1958 60 years	Location:	Anton Swart Biokinetic Rehabilitation Practice	
		Ordering physician:		
		Attending physician:		
		Comment:		

P / PQ:	128 ms / 150 ms
QRS:	88 ms
QT / QTc / QTd:	392 ms / 436 ms / -
P/QRS/T axis:	76° / 83° / 62°
Heartrate:	85 bpm

Beats

Total:	420
Normal:	420
Other:	0

RR

Minimum	690 ms
Maximum	737 ms
Mean:	711 ms
SD:	10 ms

R-R Trend

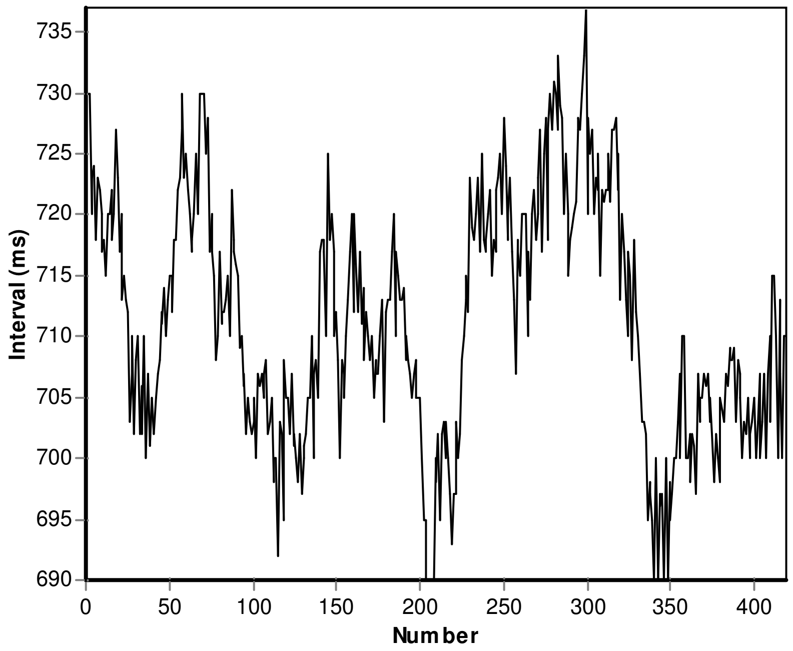

R-R Histogram

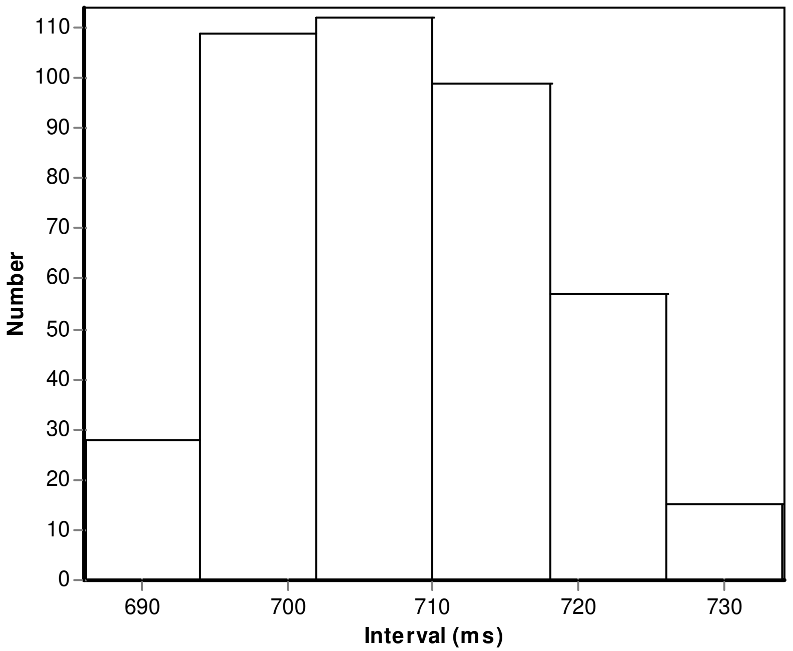

Cardio Control NV. User: Anton

Heart Rate Variability: Time Domain Analysis

**Name:**	003, 003 003	**Birthdate:**	26/01/1958
**Number:**	003	**Recorded:**	05/05/2018 14:01:53
**Gender:**	Male		

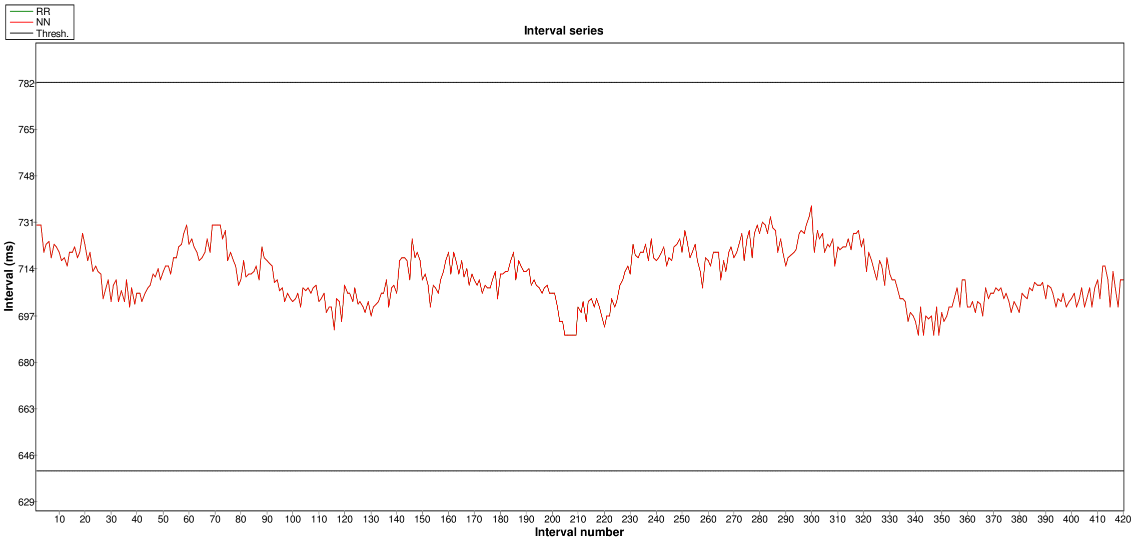

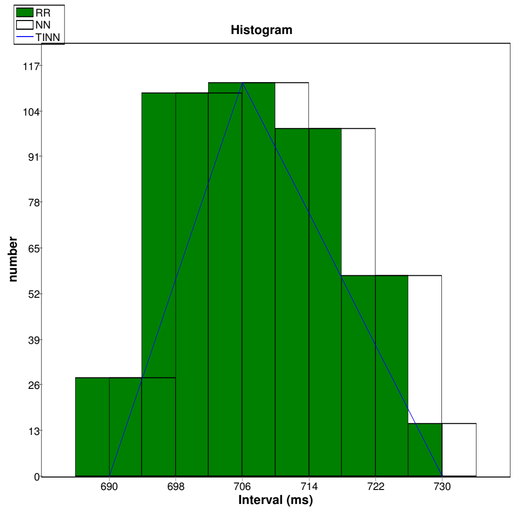

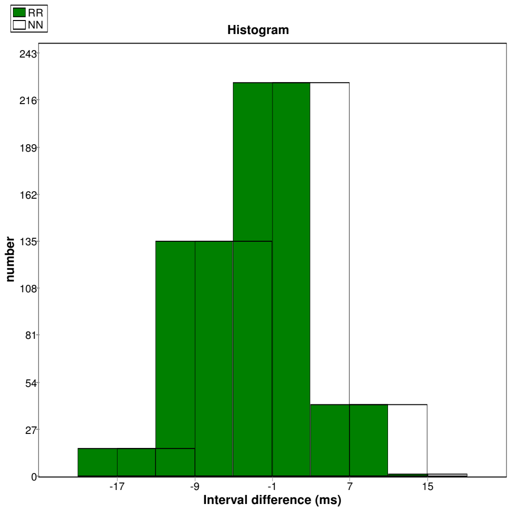

Binsize (ms) = 8

**HRV parameters**	**NN**	**RR**
SDNN (ms)	10	10
Triangular Interpolation (ms)	40	40
Triangular Index	3.75	3.75

**HRV parameters**	**NN**	**RR**
SDSD (ms)	5	5
RMSSD (ms)	5	5
NN50	0	0
NN50(1)	0	0
NN50(2)	0	0
pNN50	0.00	0.00
pNN50(1)	0.00	0.00
pNN50(2)	0.00	0.00
Logarithmic Index	2.96	2.96
SD(Logarithmic Index)	0.54	0.54

**Interval statistics**	**NN**	**RR**
Number	420	420
Minimum (ms)	690	690
Maximum (ms)	737	737
Range (ms)	47	47
Avg (ms)	711	711
SD (ms)	10	10
AvgDev (ms)	8	8
p5 (ms)	697	697
p50 (ms)	710	710
p95 (ms)	728	728
Skewness	0.09	0.09
Kurtosis	2.29	2.29

**Interval statistics**	**NN**	**RR**
Number	419	419
Minimum (ms)	-17	-17
Maximum (ms)	15	15
Range (ms)	32	32
Avg (ms)	-0	-0
SD (ms)	5	5
AvgDev (ms)	4	4
p5 (ms)	-8	-8
p50 (ms)	0	0
p95 (ms)	8	8
Skewness	0.05	0.05
Kurtosis	3.16	3.16

Heart Rate Variability: Frequency Domain Analysis

**Name:**	003, 003 003	**Birthdate:**	26/01/1958
**Number:**	003	**Recorded:**	05/05/2018 14:01:53
**Gender:**	Male		

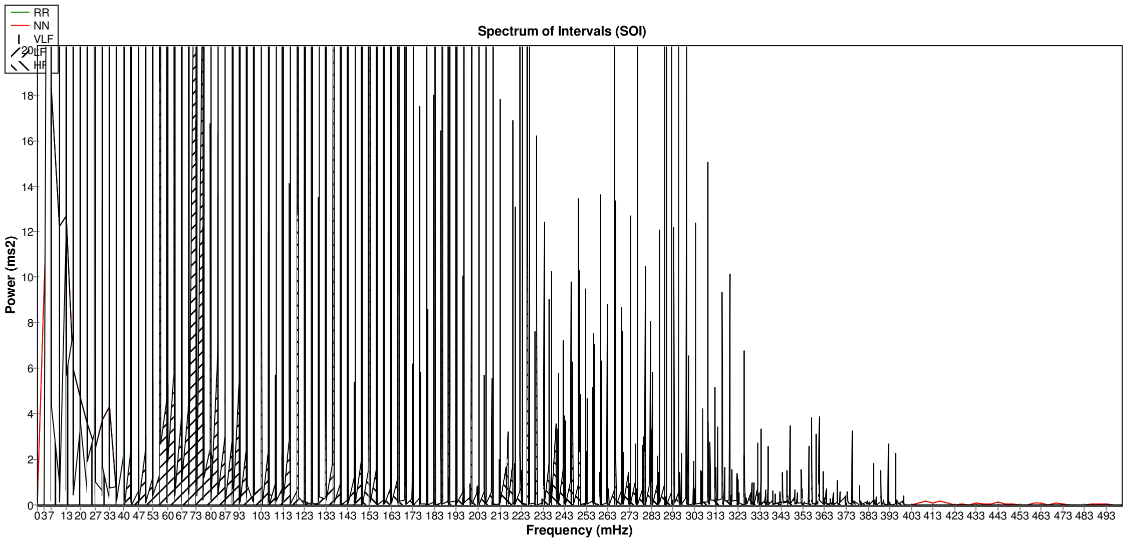

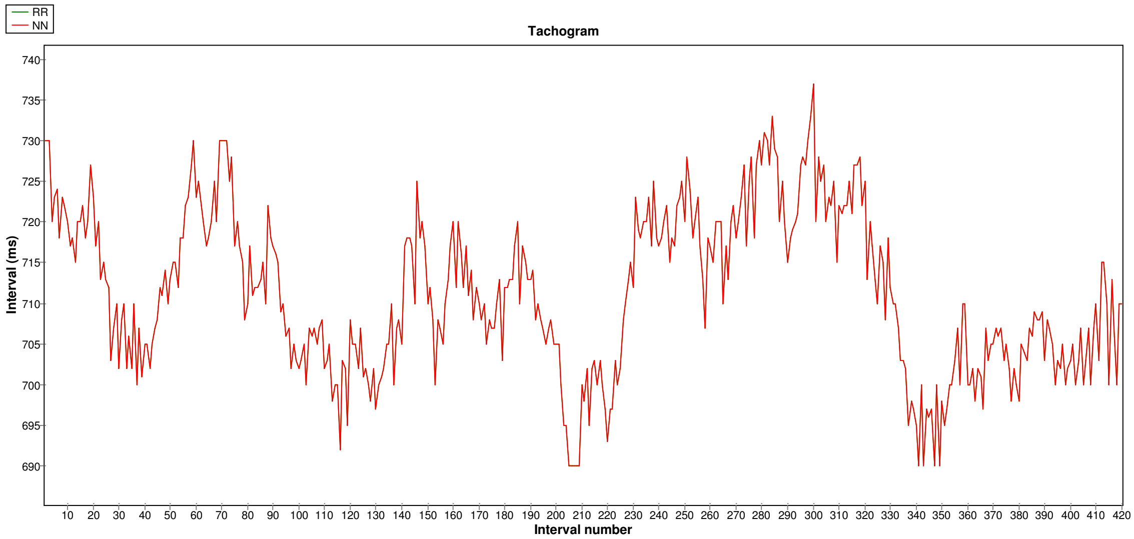

HRV parameters	NN	RR
TP (ms2)	83	83
VLF (ms2)	69	69
LF (ms2)	12	12
HF (ms2)	3	3
LF/HF	4.42	4.42
LF normalized	81.54	81.54
HF normalized	18.46	18.46
VLF peak (mHz)	7	7
LF peak (mHz)	80	80
HF peak (mHz)	346	346

HRV spectral settings	
Spectrum of Intervals (SOI)	
Frequency resolution (mHz)	3
VLF lower boundary (mHz)	3
VLF upper boundary (mHz)	40
LF upper boundary (mHz)	150
HF upper boundary (mHz)	400
Smoothing factor	1
Tapering	Hann
Fourier transform	DFT
Sample frequency (Hz)	1.41
Interval correction	Annotation
Interval threshold (%)	10
