# Supplementary material for: The effects of a 3-day mountain bike cycling race on the autonomic nervous system (ANS) and heart rate variability in amateur cyclists: a prospective quantitative research design
Source: BMC Sports Sci Med Rehabil. 2023 Jan 2;15:2. doi: 10.1186/s13102-022-00614-y (PMC9808932; doi:10.1186/s13102-022-00614-y)
Supplement: Supplementary file 1 — Additional file 1. Individual data of Participants. [file 13102_2022_614_MOESM1_ESM.zip › Individual data of Participants/HRV Data/003/ECG_003_20180506092910_.PDF]

Anton Swart Biokinetic Rehabilitation Practice

Name:	003 003 003	Recorded:	06/05/2018 09:29:10	UNCONFIRMED INTERPRETATION - MD SHOULD REVIEW
Number:	003	Recorded by:	Mr. Anton Swart	
Gender:	Male	Referring physician:		
Birthdate:	26/01/1958 60 years	Ordering physician:		
		Attending physician:		
P / PQ:	125 ms / 152 ms	Location:	Anton Swart Biokinetic Rehabilitation Practi	
QRS:	93 ms	Comment:		
QT / QTc / QTd:	428 ms / 442 ms / -			
P/QRS/T axis:	77° / 79° / 46°			
Heartrate:	68 bpm			

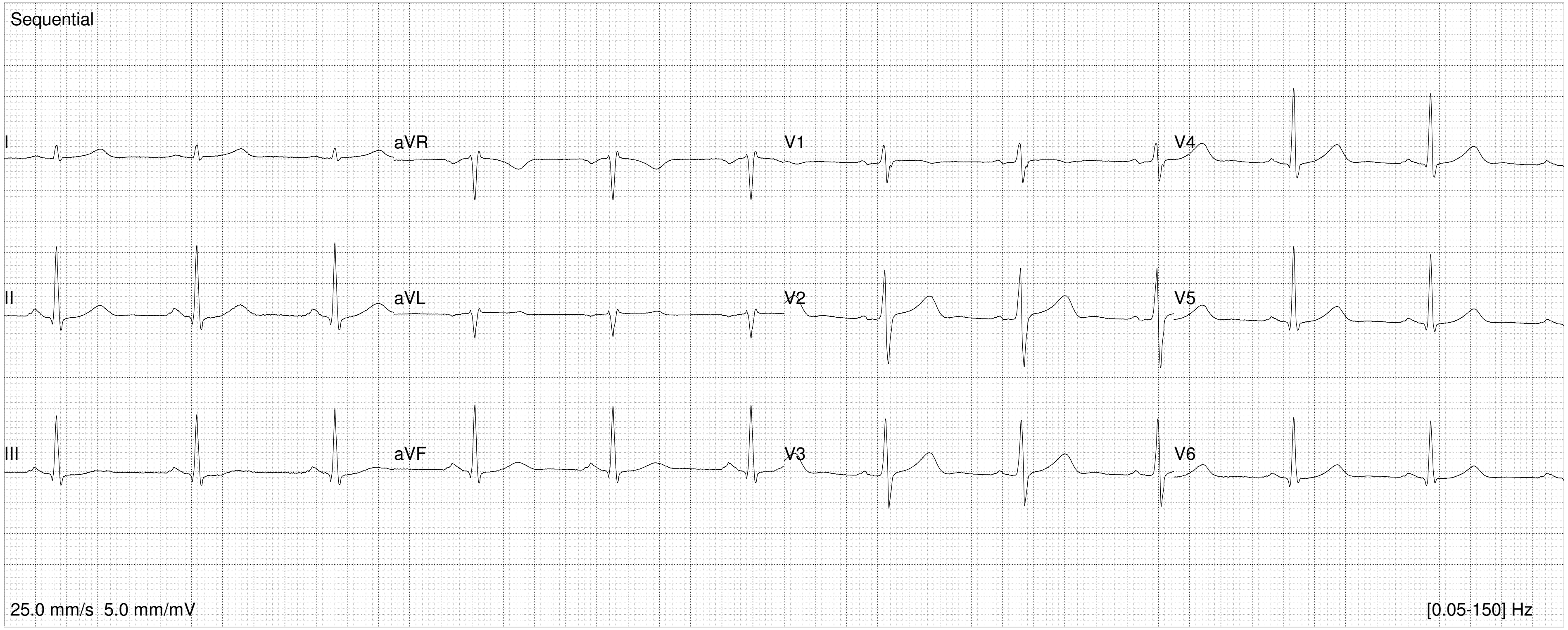

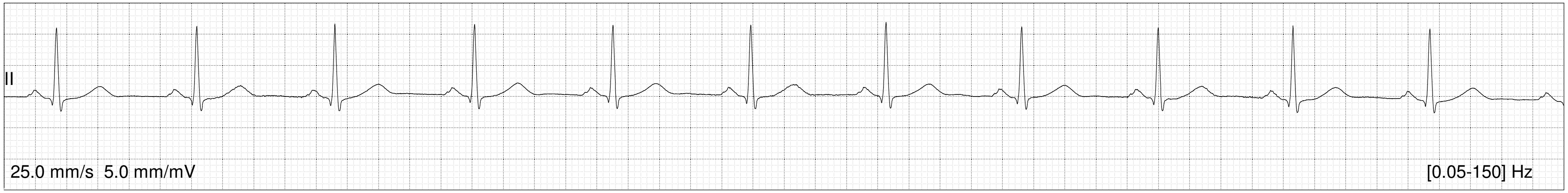

Anton Swart Biokinetic Rehabilitation Practice

Name:	003 003 003	Recorded:	06/05/2018 09:29:10	UNCONFIRMED INTERPRETATION - MD SHOULD REVIEW
Number:	003	Recorded by:	Mr. Anton Swart	
Gender:	Male	Referring physician:		
Birthdate:	26/01/1958 60 years	Location:	Anton Swart Biokinetic Rehabilitation Practice	
		Ordering physician:		
		Attending physician:		
		Comment:		

P / PQ:	125 ms / 152 ms
QRS:	93 ms
QT / QTc / QTd:	428 ms / 442 ms / -
P/QRS/T axis:	77° / 79° / 46°
Heartrate:	68 bpm

Beats

Total:	339
Normal:	339
Other:	0

RR

Minimum	848 ms
Maximum	945 ms
Mean:	882 ms
SD:	16 ms

R-R Trend

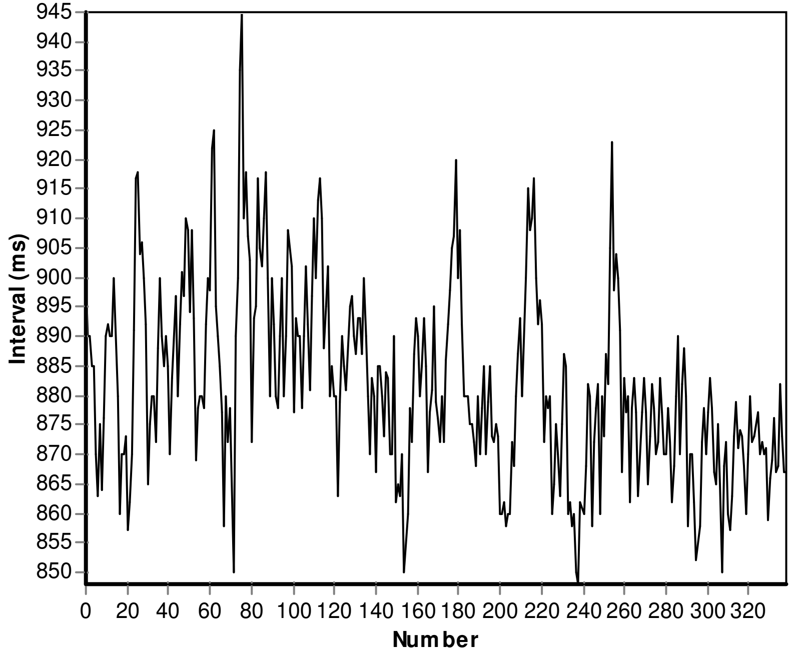

R-R Histogram

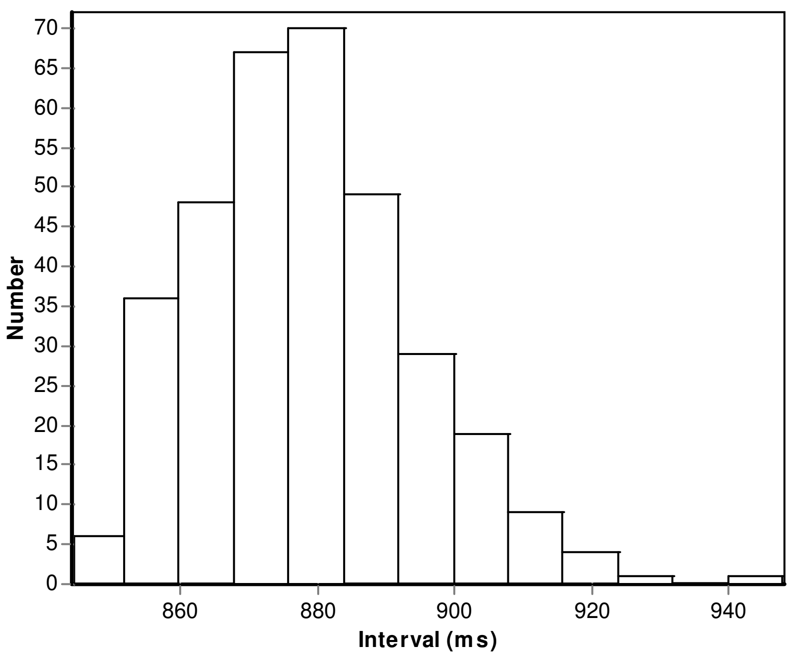

Cardio Control NV. User: Anton

Heart Rate Variability: Time Domain Analysis

**Name:**	003, 003 003	**Birthdate:**	26/01/1958
**Number:**	003	**Recorded:**	06/05/2018 09:29:10
**Gender:**	Male		

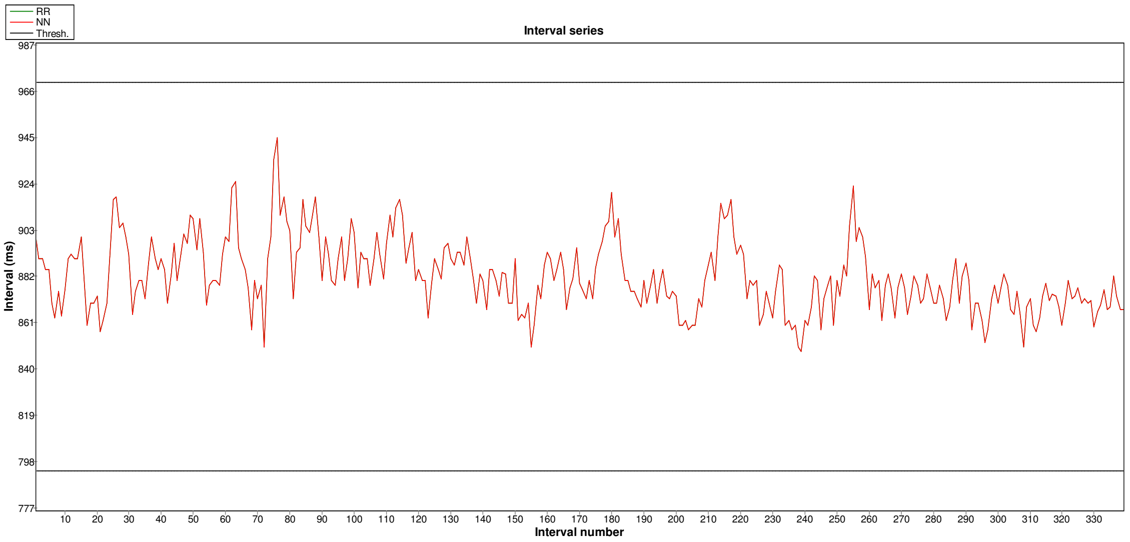

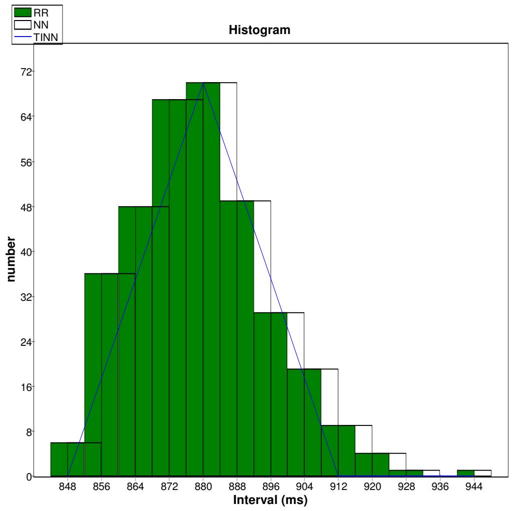

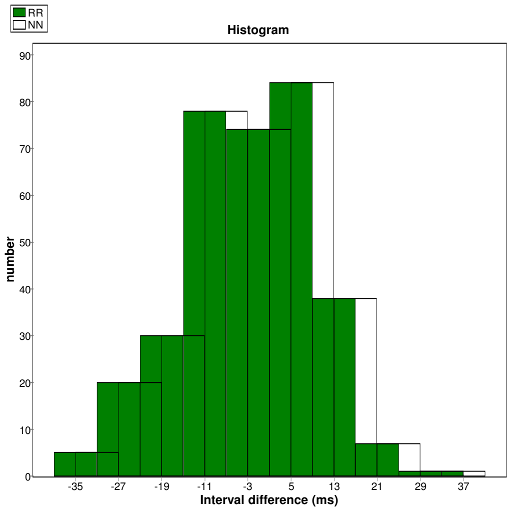

Binsize (ms) = 8

**HRV parameters**	**NN**	**RR**
SDNN (ms)	16	16
Triangular Interpolation (ms)	64	64
Triangular Index	4.84	4.84

**HRV parameters**	**NN**	**RR**
SDSD (ms)	12	12
RMSSD (ms)	12	12
NN50	0	0
NN50(1)	0	0
NN50(2)	0	0
pNN50	0.00	0.00
pNN50(1)	0.00	0.00
pNN50(2)	0.00	0.00
Logarithmic Index	1.11	1.11
SD(Logarithmic Index)	0.11	0.11

**Interval statistics**	**NN**	**RR**
Number	339	339
Minimum (ms)	848	848
Maximum (ms)	945	945
Range (ms)	97	97
Avg (ms)	882	882
SD (ms)	16	16
AvgDev (ms)	13	13
p5 (ms)	860	860
p50 (ms)	880	880
p95 (ms)	910	910
Skewness	0.62	0.62
Kurtosis	3.47	3.47

**Interval statistics**	**NN**	**RR**
Number	338	338
Minimum (ms)	-35	-35
Maximum (ms)	40	40
Range (ms)	75	75
Avg (ms)	-0	-0
SD (ms)	12	12
AvgDev (ms)	10	10
p5 (ms)	-21	-21
p50 (ms)	0	0
p95 (ms)	18	18
Skewness	-0.15	-0.15
Kurtosis	3.02	3.02

Cardio Control NV. User: Anton

Heart Rate Variability: Frequency Domain Analysis

**Name:**	003, 003 003	**Birthdate:**	26/01/1958
**Number:**	003	**Recorded:**	06/05/2018 09:29:10
**Gender:**	Male		

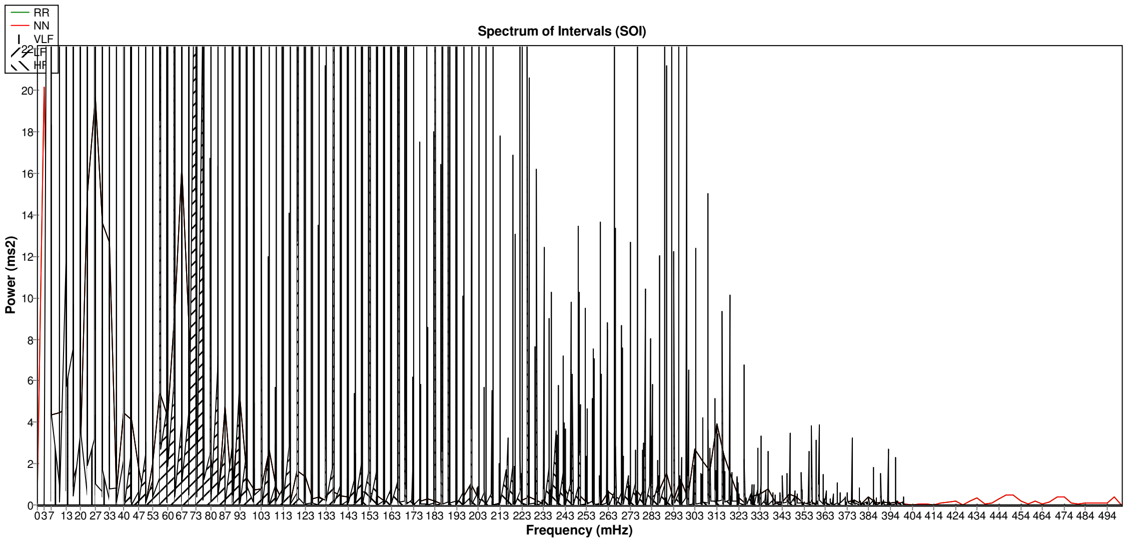

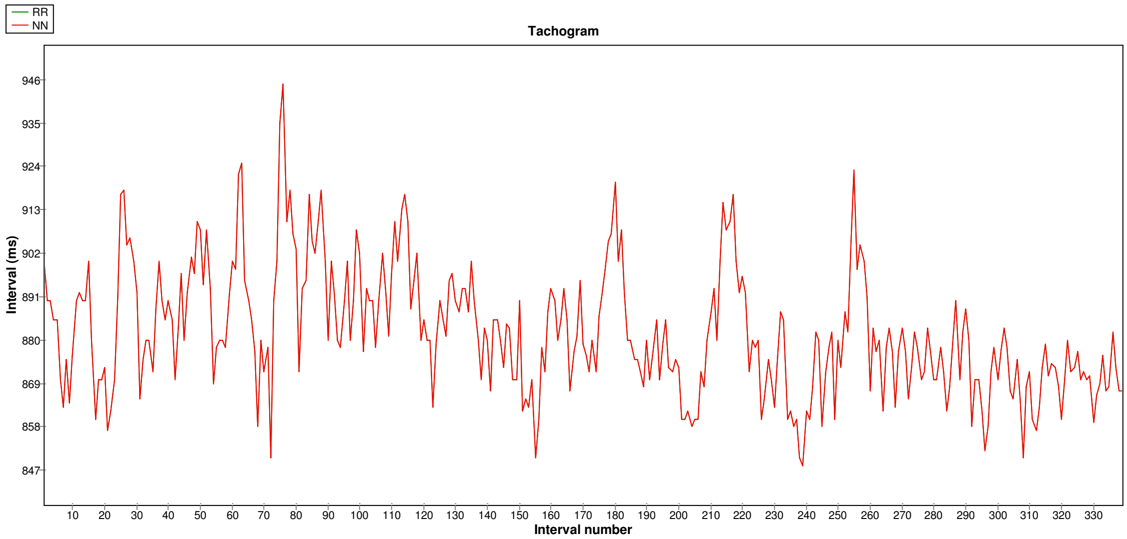

**HRV parameters**	**NN**	**RR**
TP (ms2)	197	197
VLF (ms2)	83	83
LF (ms2)	81	81
HF (ms2)	33	33
LF/HF	2.41	2.41
LF normalized	70.70	70.70
HF normalized	29.30	29.30
VLF peak (mHz)	27	27
LF peak (mHz)	67	67
HF peak (mHz)	313	313

**HRV spectral settings**	
Spectrum of Intervals (SOI)	
Frequency resolution (mHz)	3
VLF lower boundary (mHz)	3
VLF upper boundary (mHz)	40
LF upper boundary (mHz)	150
HF upper boundary (mHz)	400
Smoothing factor	1
Tapering	Hann
Fourier transform	DFT
Sample frequency (Hz)	1.13
Interval correction	Annotation
Interval threshold (%)	10
