# Supplementary material for: The effects of a 3-day mountain bike cycling race on the autonomic nervous system (ANS) and heart rate variability in amateur cyclists: a prospective quantitative research design
Source: BMC Sports Sci Med Rehabil. 2023 Jan 2;15:2. doi: 10.1186/s13102-022-00614-y (PMC9808932; doi:10.1186/s13102-022-00614-y)
Supplement: Supplementary file 1 — Additional file 1. Individual data of Participants. [file 13102_2022_614_MOESM1_ESM.zip › Individual data of Participants/HRV Data/004/ECG_004_20180501074614_.PDF]

Anton Swart Biokinetic Rehabilitation Practice

Name:	004 004 004	Recorded:	01/05/2018 07:46:14	UNCONFIRMED INTERPRETATION - MD SHOULD REVIEW
Number:	004	Recorded by:	Mr. Anton Swart	
Gender:	Male	Referring physician:		
Birthdate:	13/11/1964 53 years	Ordering physician:		
		Attending physician:		
		Location:	Anton Swart Biokinetic Rehabilitation Practi	
		Comment:		

P / PQ:	125 ms / 180 ms
QRS:	105 ms
QT / QTc / QTd:	475 ms / 458 ms / -
P/QRS/T axis:	71° / 80° / 69°
Heartrate:	50 bpm

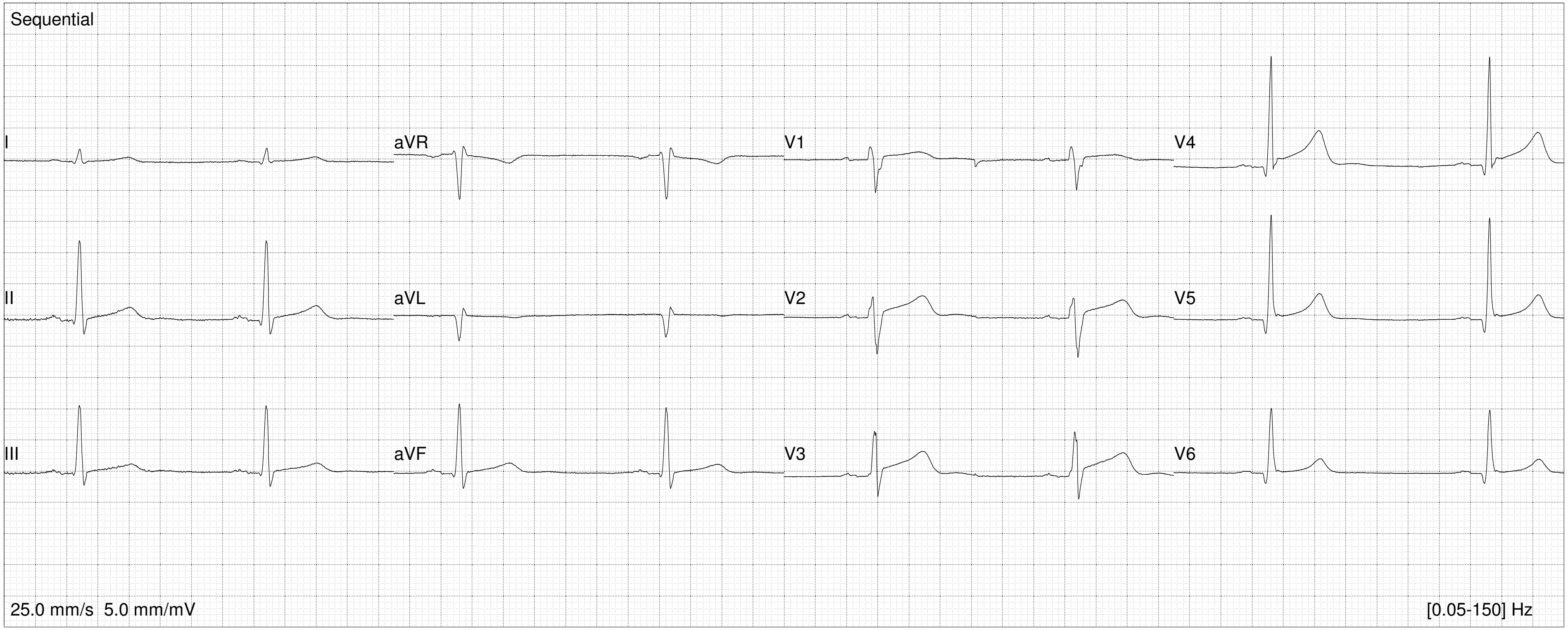

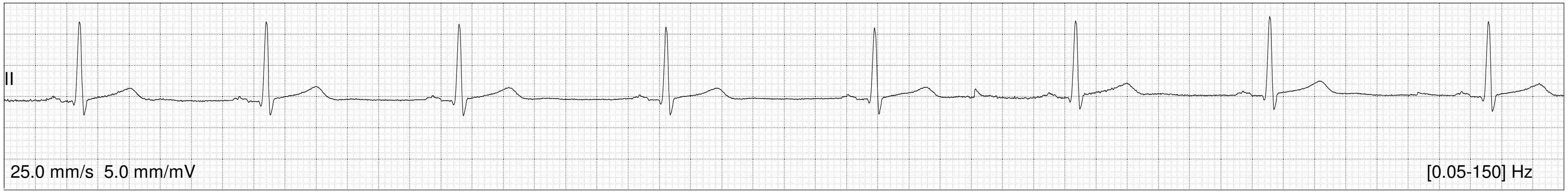

Anton Swart Biokinetic Rehabilitation Practice

Name:	004 004 004	Recorded:	01/05/2018 07:46:14	UNCONFIRMED INTERPRETATION - MD SHOULD REVIEW
Number:	004	Recorded by:	Mr. Anton Swart	
Gender:	Male	Referring physician:		
Birthdate:	13/11/1964 53 years	Location:	Anton Swart Biokinetic Rehabilitation Practice	
P / PQ:	125 ms / 180 ms	Ordering physician:		
QRS:	105 ms	Attending physician:		
QT / QTc / QTd:	475 ms / 458 ms / -	Comment:		
P/QRS/T axis:	71° / 80° / 69°			
Heartrate:	50 bpm			

Beats

Total:	247
Normal:	247
Other:	0

RR

Minimum	983 ms
Maximum	1405 ms
Mean:	1209 ms
SD:	73 ms

R-R Trend

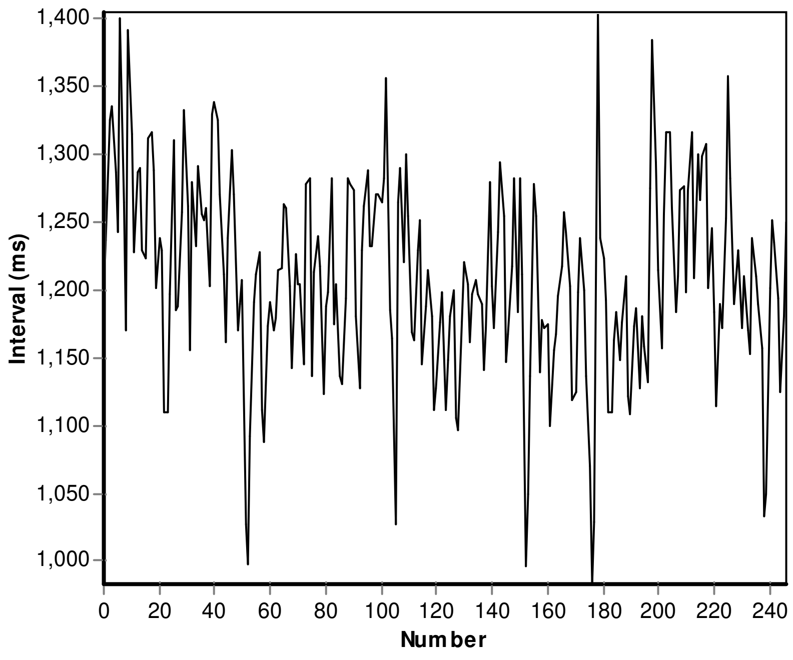

R-R Histogram

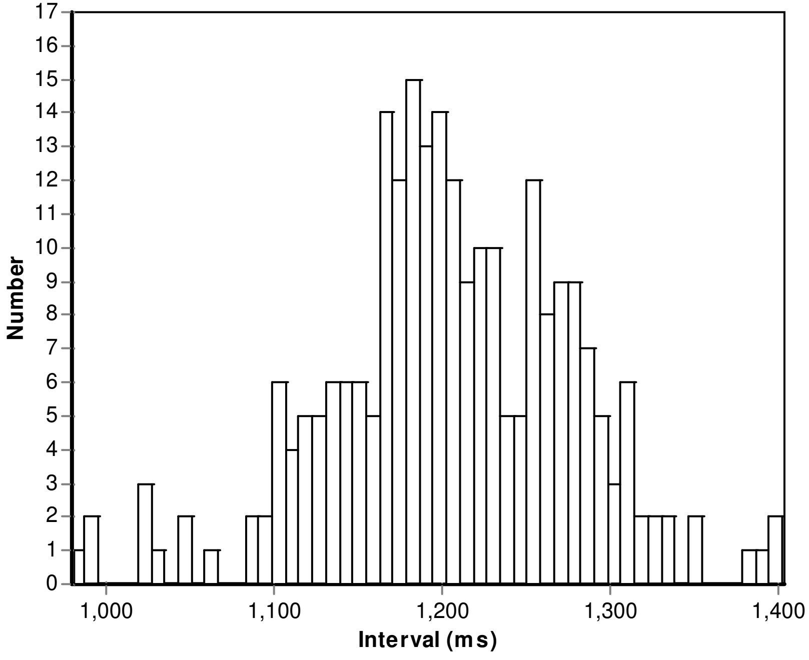

Cardio Control NV. User: Anton

Heart Rate Variability: Time Domain Analysis

**Name:**	004, 004 004	**Birthdate:**	13/11/1964
**Number:**	004	**Recorded:**	01/05/2018 07:46:14
**Gender:**	Male		

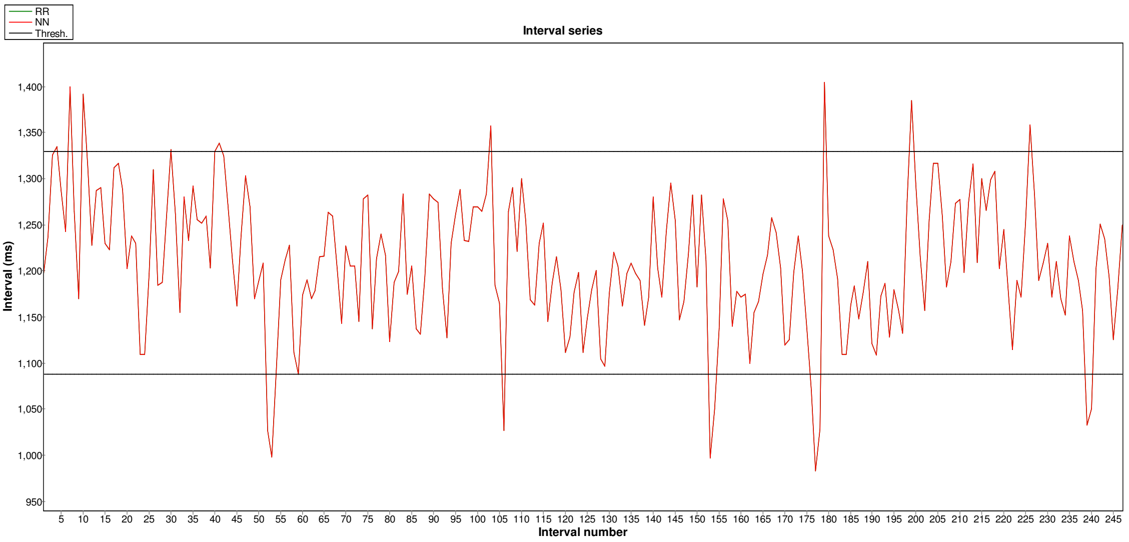

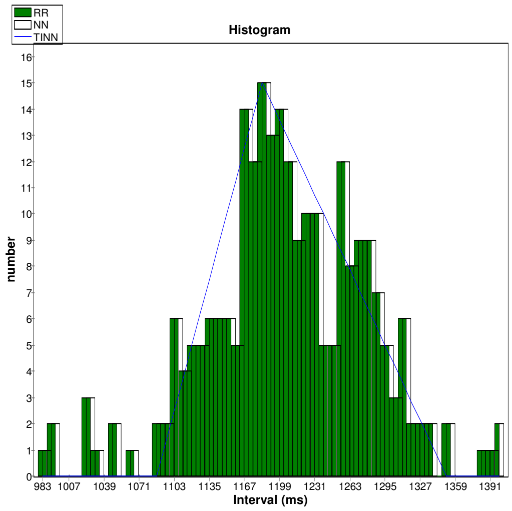

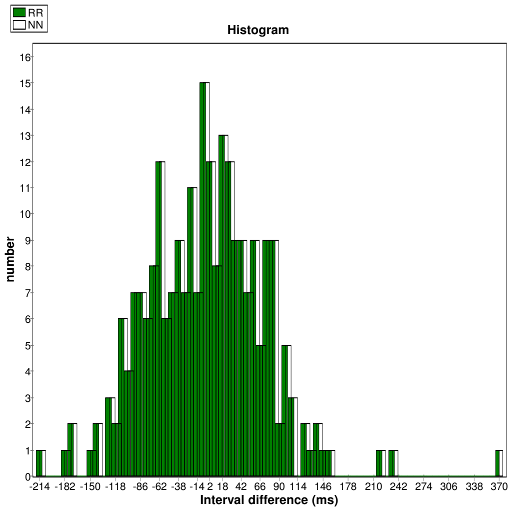

Binsize (ms) = 8

**HRV parameters**	**NN**	**RR**
SDNN (ms)	73	73
Triangular Interpolation (ms)	264	264
Triangular Index	16.47	16.47

**HRV parameters**	**NN**	**RR**
SDSD (ms)	75	75
RMSSD (ms)	75	75
NN50	123	123
NN50(1)	64	64
NN50(2)	59	59
pNN50	0.50	0.50
pNN50(1)	0.26	0.26
pNN50(2)	0.24	0.24
Logarithmic Index	0.15	0.15
SD(Logarithmic Index)	0.01	0.01

**Interval statistics**	**NN**	**RR**
Number	247	247
Minimum (ms)	983	983
Maximum (ms)	1405	1405
Range (ms)	422	422
Avg (ms)	1209	1209
SD (ms)	73	73
AvgDev (ms)	56	56
p5 (ms)	1097	1097
p50 (ms)	1205	1205
p95 (ms)	1325	1325
Skewness	-0.22	-0.22
Kurtosis	3.53	3.53

**Interval statistics**	**NN**	**RR**
Number	246	246
Minimum (ms)	-214	-214
Maximum (ms)	376	376
Range (ms)	590	590
Avg (ms)	0	0
SD (ms)	75	75
AvgDev (ms)	58	58
p5 (ms)	-112	-112
p50 (ms)	1	1
p95 (ms)	109	109
Skewness	0.50	0.50
Kurtosis	5.21	5.21

Cardio Control NV. User: Anton

Heart Rate Variability: Frequency Domain Analysis

**Name:**	004, 004 004	**Birthdate:**	13/11/1964
**Number:**	004	**Recorded:**	01/05/2018 07:46:14
**Gender:**	Male		

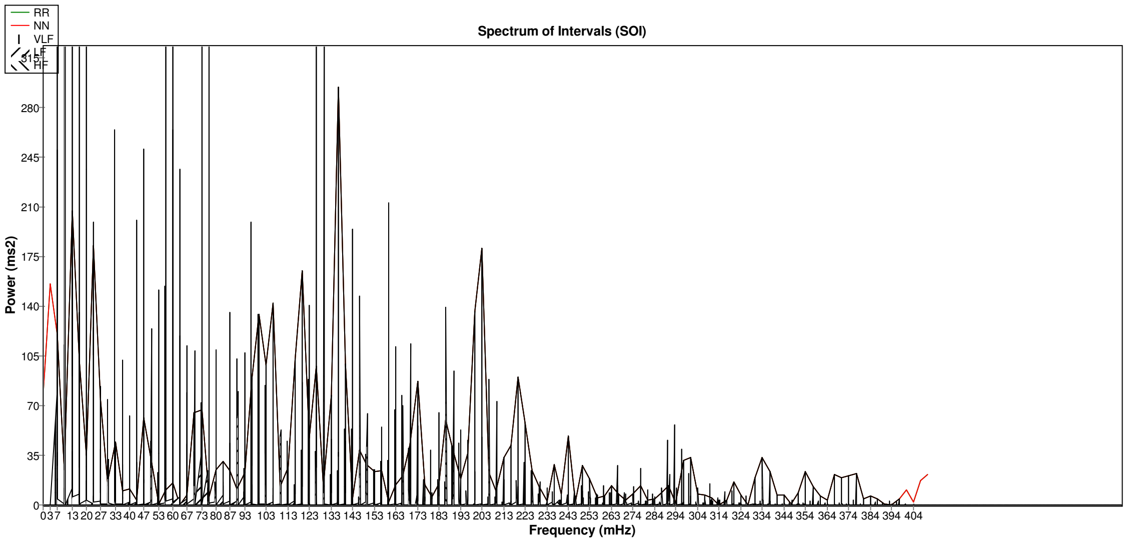

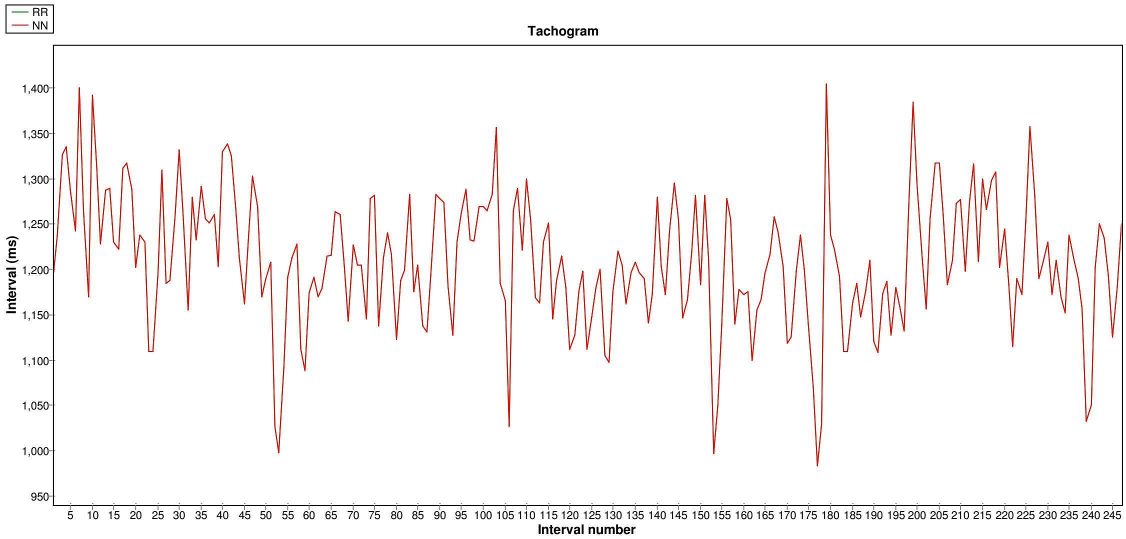

**HRV parameters**	**NN**	**RR**
TP (ms2)	4291	4291
VLF (ms2)	829	829
LF (ms2)	1859	1859
HF (ms2)	1603	1603
LF/HF	1.16	1.16
LF normalized	53.69	53.69
HF normalized	46.31	46.31
VLF peak (mHz)	13	13
LF peak (mHz)	137	137
HF peak (mHz)	203	203

**HRV spectral settings**	
Spectrum of Intervals (SOI)	
Frequency resolution (mHz)	3
VLF lower boundary (mHz)	3
VLF upper boundary (mHz)	40
LF upper boundary (mHz)	150
HF upper boundary (mHz)	400
Smoothing factor	1
Tapering	Hann
Fourier transform	DFT
Sample frequency (Hz)	0.83
Interval correction	Annotation
Interval threshold (%)	10
