# Supplementary material for: The effects of a 3-day mountain bike cycling race on the autonomic nervous system (ANS) and heart rate variability in amateur cyclists: a prospective quantitative research design
Source: BMC Sports Sci Med Rehabil. 2023 Jan 2;15:2. doi: 10.1186/s13102-022-00614-y (PMC9808932; doi:10.1186/s13102-022-00614-y)
Supplement: Supplementary file 1 — Additional file 1. Individual data of Participants. [file 13102_2022_614_MOESM1_ESM.zip › Individual data of Participants/HRV Data/004/ECG_004_20180503170007_.PDF]

Anton Swart Biokinetic Rehabilitation Practice

UNCONFIRMED INTERPRETATION - MD SHOULD REVIEW

Name:	004 004 004	Recorded:	03/05/2018 17:00:07
Number:	004	Recorded by:	Mr. Anton Swart
Gender:	Male	Referring physician:	
Birthdate:	13/11/1964 53 years	Ordering physician:	
		Attending physician:	
P / PQ:	115 ms / 157 ms	Location:	Anton Swart Biokinetic Rehabilitation Practi
QRS:	110 ms	Comment:	
QT / QTc / QTd:	405 ms / 438 ms / -		
P/QRS/T axis:	71° / 82° / 74°		
Heartrate:	79 bpm		

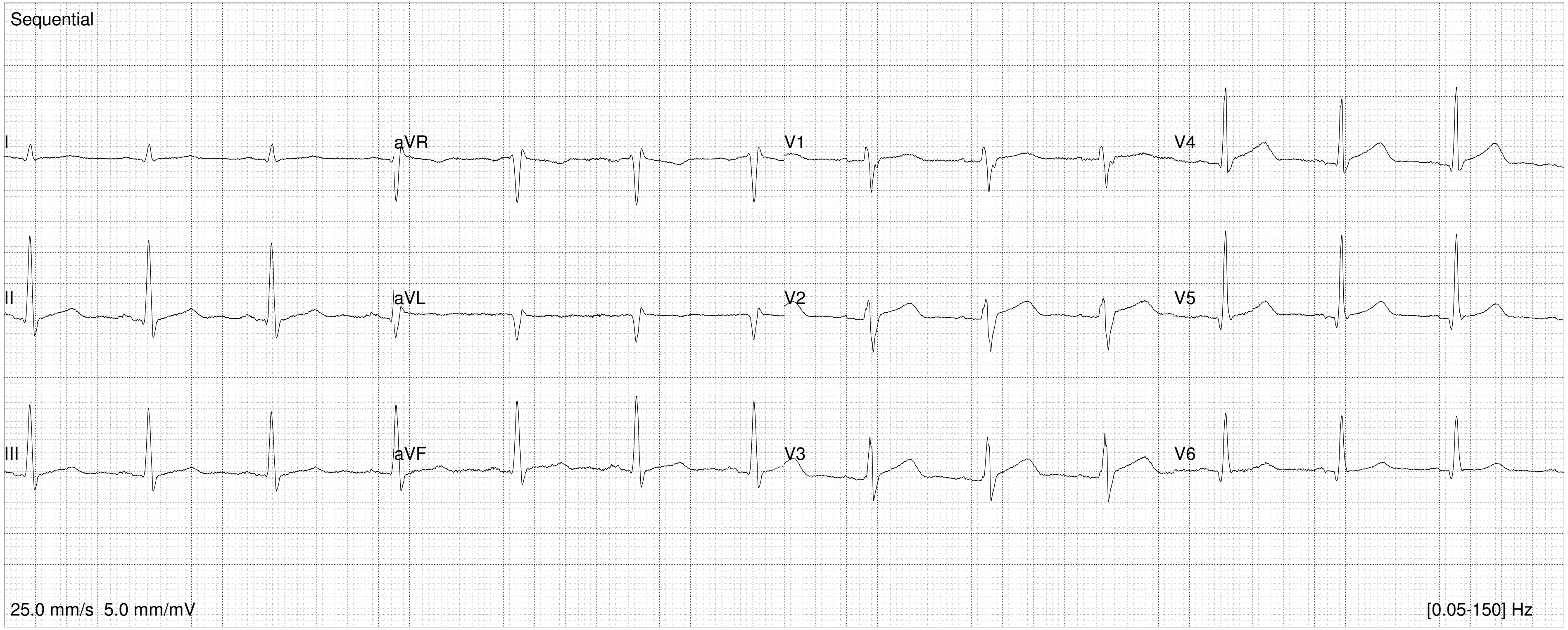

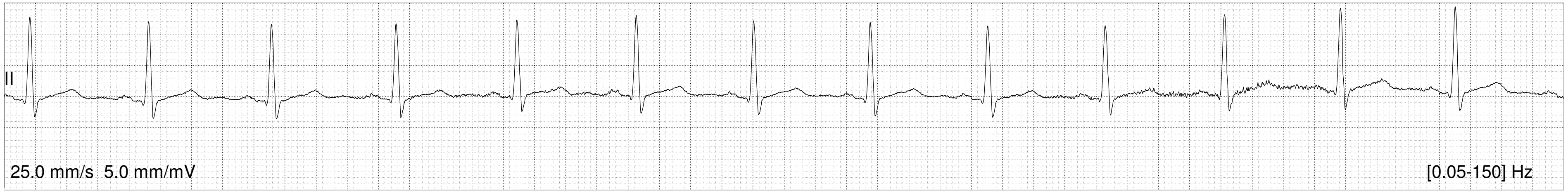

Anton Swart Biokinetic Rehabilitation Practice

Name:	004 004 004	Recorded:	03/05/2018 17:00:07	UNCONFIRMED INTERPRETATION - MD SHOULD REVIEW
Number:	004	Recorded by:	Mr. Anton Swart	
Gender:	Male	Referring physician:		
Birthdate:	13/11/1964 53 years	Location:	Anton Swart Biokinetic Rehabilitation Practice	
		Ordering physician:		
		Attending physician:		
		Comment:		

P / PQ:	115 ms / 157 ms
QRS:	110 ms
QT / QTc / QTd:	405 ms / 438 ms / -
P/QRS/T axis:	71° / 82° / 74°
Heartrate:	79 bpm

Beats

Total:	394
Normal:	394
Other:	0

RR

Minimum	699 ms
Maximum	814 ms
Mean:	760 ms
SD:	20 ms

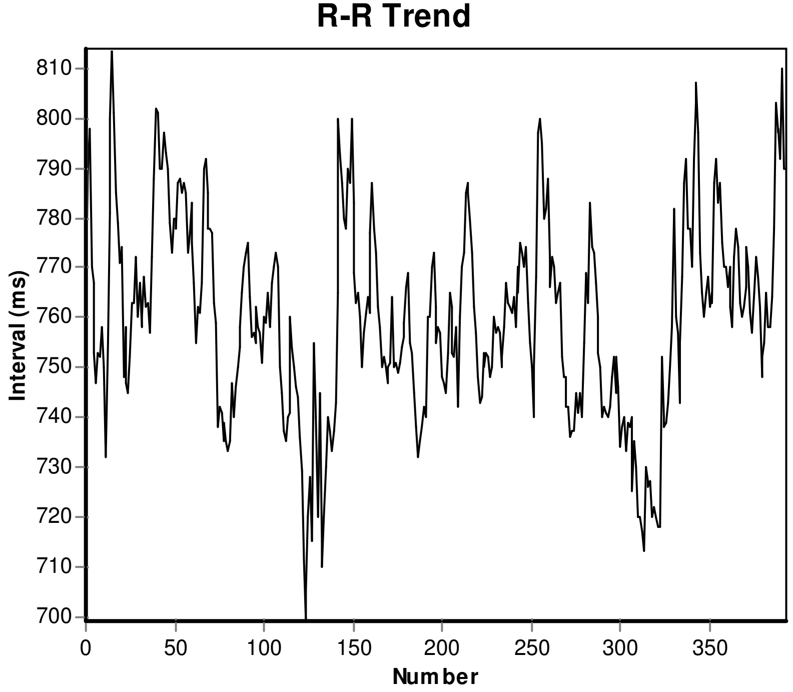

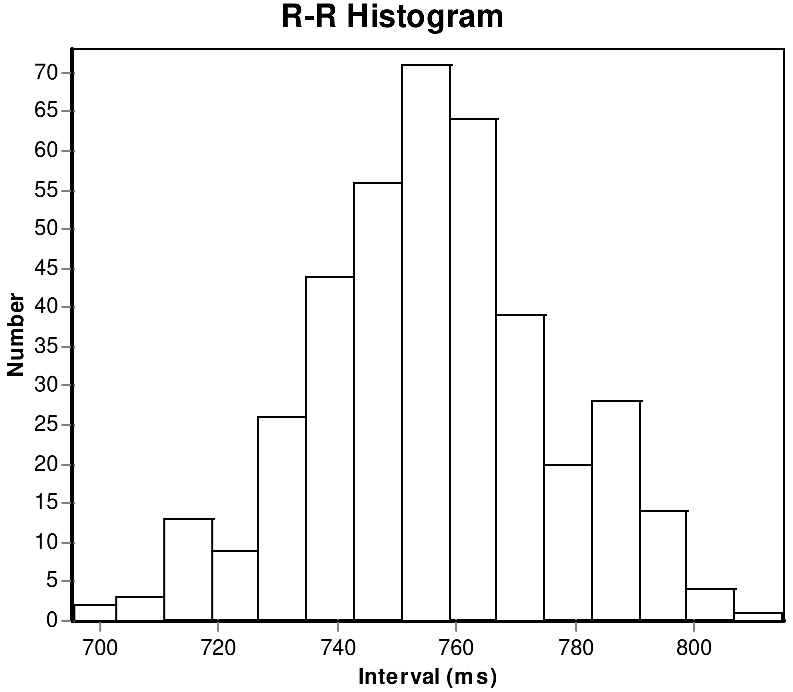

Cardio Control NV. User: Anton

Heart Rate Variability: Time Domain Analysis

**Name:**	004, 004 004	**Birthdate:**	13/11/1964
**Number:**	004	**Recorded:**	03/05/2018 17:00:07
**Gender:**	Male		

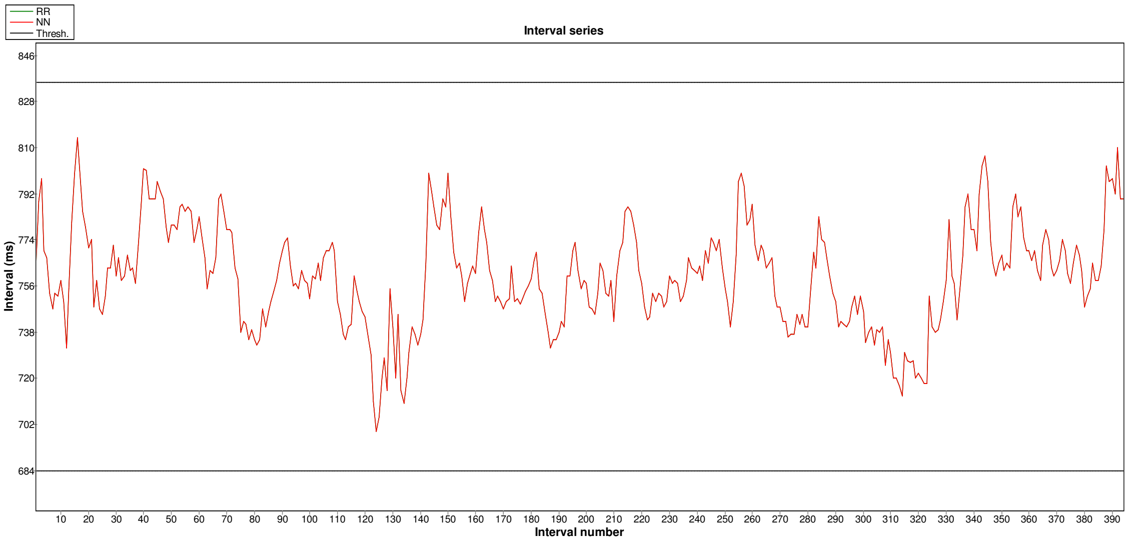

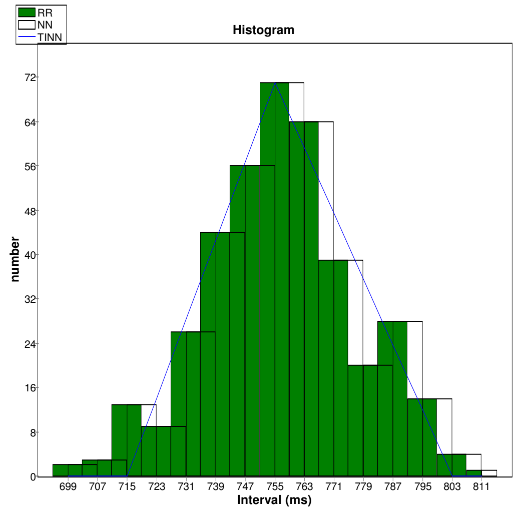

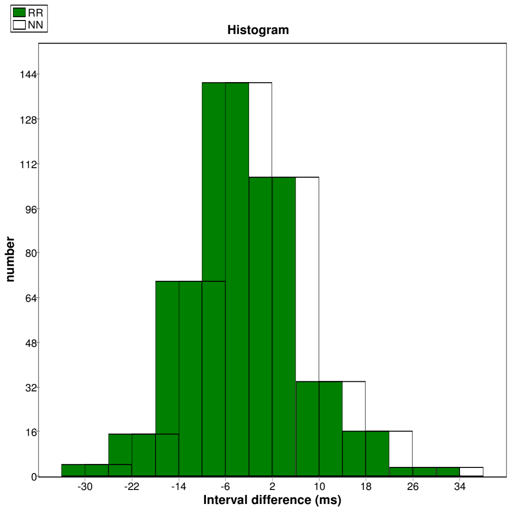

Binsize (ms) = 8

**HRV parameters**	**NN**	**RR**
SDNN (ms)	20	20
Triangular Interpolation (ms)	88	88
Triangular Index	5.55	5.55

**HRV parameters**	**NN**	**RR**
SDSD (ms)	10	10
RMSSD (ms)	10	10
NN50	0	0
NN50(1)	0	0
NN50(2)	0	0
pNN50	0.00	0.00
pNN50(1)	0.00	0.00
pNN50(2)	0.00	0.00
Logarithmic Index	1.15	1.15
SD(Logarithmic Index)	0.05	0.05

**Interval statistics**	**NN**	**RR**
Number	394	394
Minimum (ms)	699	699
Maximum (ms)	814	814
Range (ms)	115	115
Avg (ms)	760	760
SD (ms)	20	20
AvgDev (ms)	16	16
p5 (ms)	726	726
p50 (ms)	760	760
p95 (ms)	794	794
Skewness	-0.02	-0.02
Kurtosis	2.96	2.96

**Interval statistics**	**NN**	**RR**
Number	393	393
Minimum (ms)	-30	-30
Maximum (ms)	40	40
Range (ms)	70	70
Avg (ms)	0	0
SD (ms)	10	10
AvgDev (ms)	7	7
p5 (ms)	-14	-14
p50 (ms)	-1	-1
p95 (ms)	19	19
Skewness	0.52	0.52
Kurtosis	4.45	4.45

Cardio Control NV. User: Anton

Heart Rate Variability: Frequency Domain Analysis

**Name:**	004, 004 004	**Birthdate:**	13/11/1964
**Number:**	004	**Recorded:**	03/05/2018 17:00:07
**Gender:**	Male		

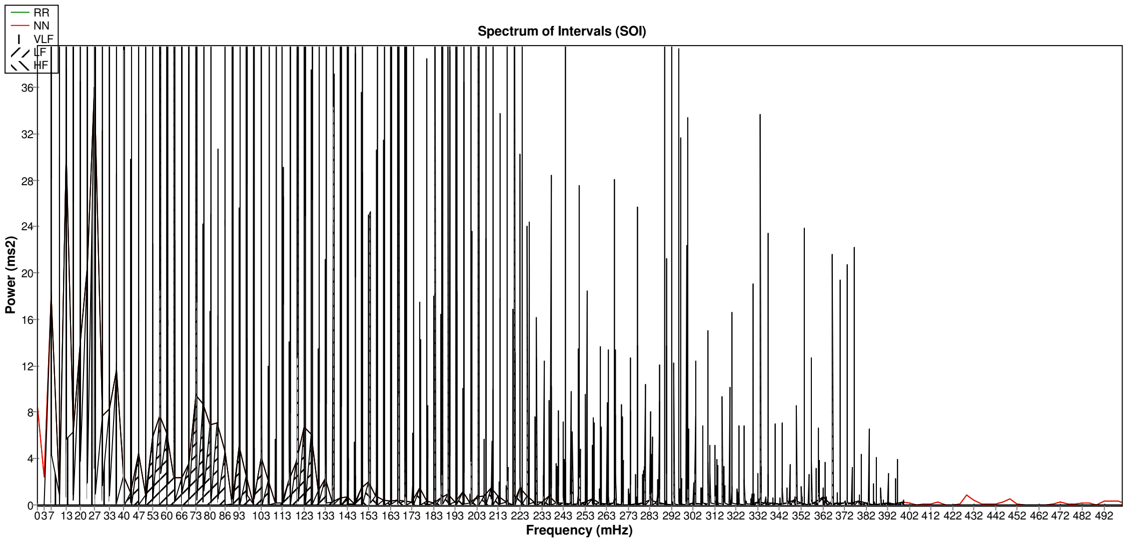

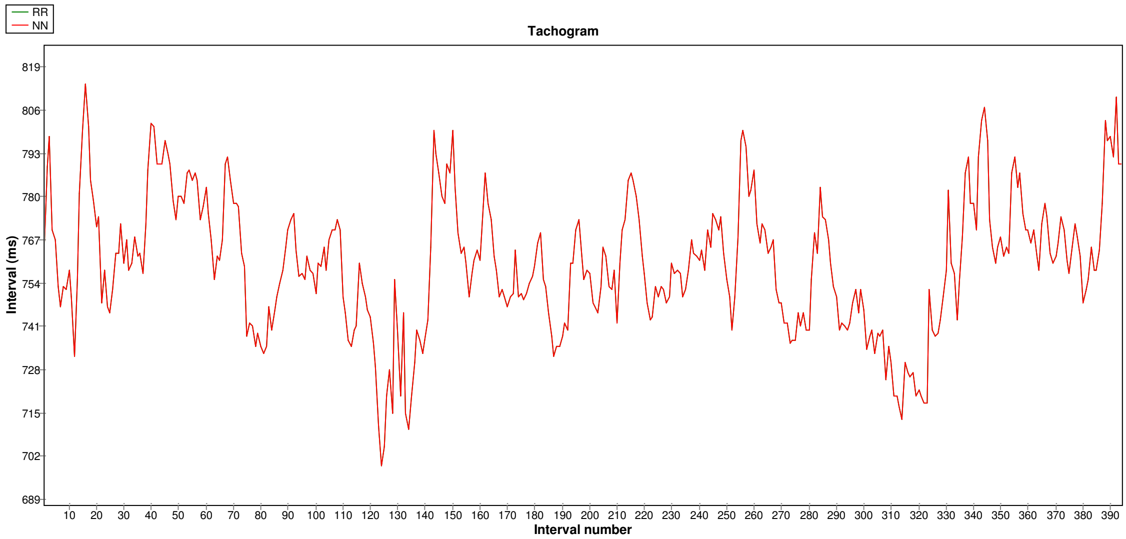

**HRV parameters**	**NN**	**RR**
TP (ms2)	292	292
VLF (ms2)	156	156
LF (ms2)	111	111
HF (ms2)	25	25
LF/HF	4.49	4.49
LF normalized	81.78	81.78
HF normalized	18.22	18.22
VLF peak (mHz)	27	27
LF peak (mHz)	73	73
HF peak (mHz)	153	153

**HRV spectral settings**	
Spectrum of Intervals (SOI)	
Frequency resolution (mHz)	3
VLF lower boundary (mHz)	3
VLF upper boundary (mHz)	40
LF upper boundary (mHz)	150
HF upper boundary (mHz)	400
Smoothing factor	1
Tapering	Hann
Fourier transform	DFT
Sample frequency (Hz)	1.32
Interval correction	Annotation
Interval threshold (%)	10
