# Supplementary material for: The effects of a 3-day mountain bike cycling race on the autonomic nervous system (ANS) and heart rate variability in amateur cyclists: a prospective quantitative research design
Source: BMC Sports Sci Med Rehabil. 2023 Jan 2;15:2. doi: 10.1186/s13102-022-00614-y (PMC9808932; doi:10.1186/s13102-022-00614-y)
Supplement: Supplementary file 1 — Additional file 1. Individual data of Participants. [file 13102_2022_614_MOESM1_ESM.zip › Individual data of Participants/HRV Data/004/ECG_004_20180504123828_.PDF]

Anton Swart Biokinetic Rehabilitation Practice

Name: 004 004 004
Number: 004
Gender: Male
Birthdate: 13/11/1964 53 years

Recorded: 04/05/2018 12:38:28
Recorded by: Mr. Anton Swart
Referring physician:
Ordering physician:
Attending physician:
Location: Anton Swart Biokinetic Rehabilitation Practi
Comment:

UNCONFIRMED INTERPRETATION - MD SHOULD REVIEW

P / PQ: 173 ms / 207 ms
QRS: 108 ms
QT / QTc / QTd: 401 ms / 445 ms / -
P/QRS/T axis: 77° / 82° / 78°
Heartrate: 85 bpm

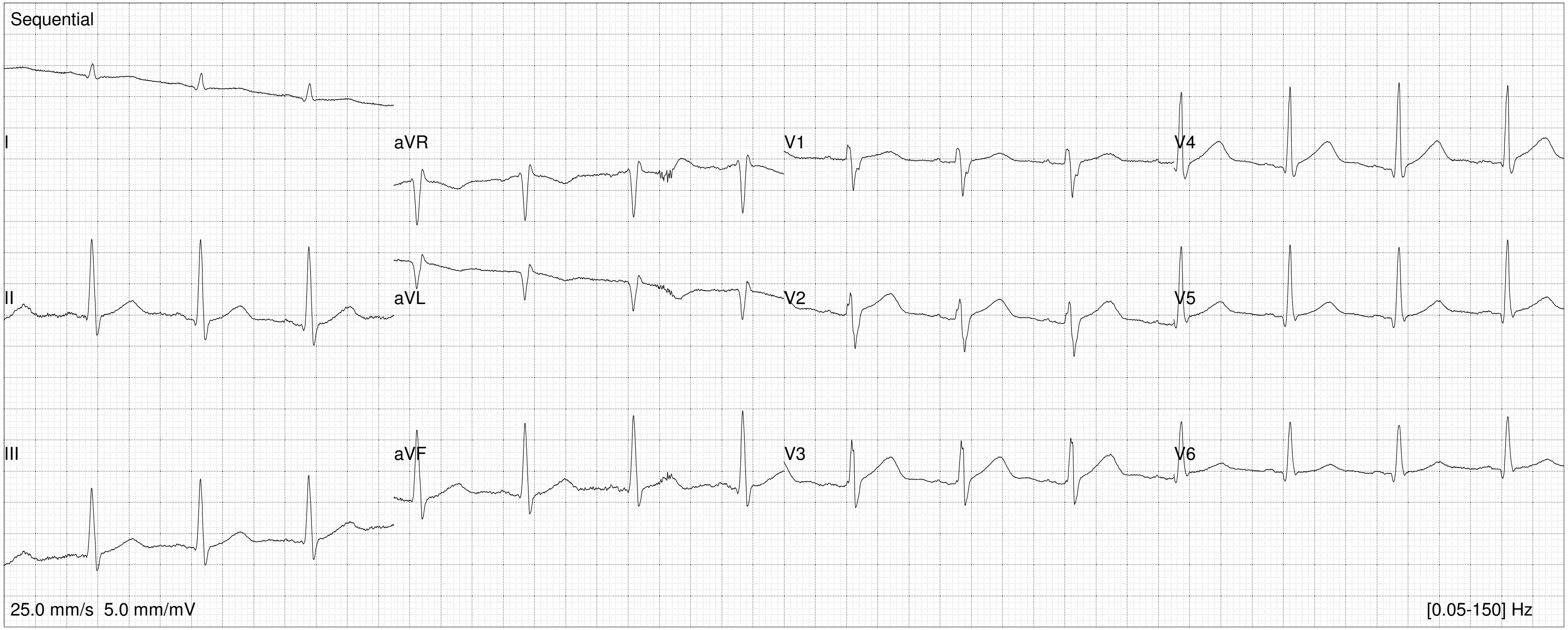

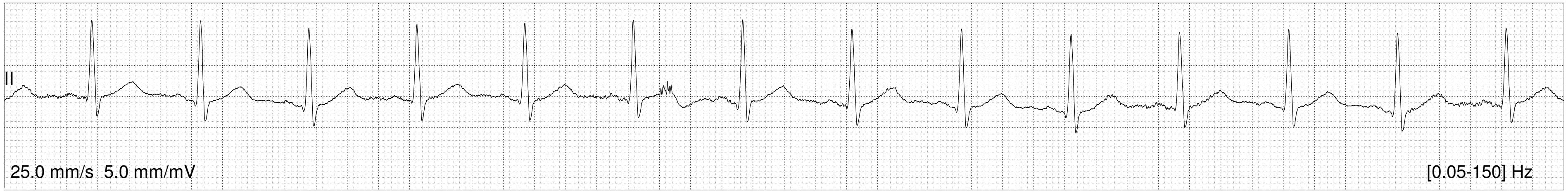

Anton Swart Biokinetic Rehabilitation Practice

Name:	004 004 004	Recorded:	04/05/2018 12:38:28	UNCONFIRMED INTERPRETATION - MD SHOULD REVIEW
Number:	004	Recorded by:	Mr. Anton Swart	
Gender:	Male	Referring physician:		
Birthdate:	13/11/1964 53 years	Location:	Anton Swart Biokinetic Rehabilitation Practice	
		Ordering physician:		
		Attending physician:		
		Comment:		

P / PQ:	173 ms / 207 ms
QRS:	108 ms
QT / QTc / QTd:	401 ms / 445 ms / -
P/QRS/T axis:	77° / 82° / 78°
Heartrate:	85 bpm

Beats

Total:	421
Normal:	421
Other:	0

RR

Minimum	650 ms
Maximum	763 ms
Mean:	709 ms
SD:	17 ms

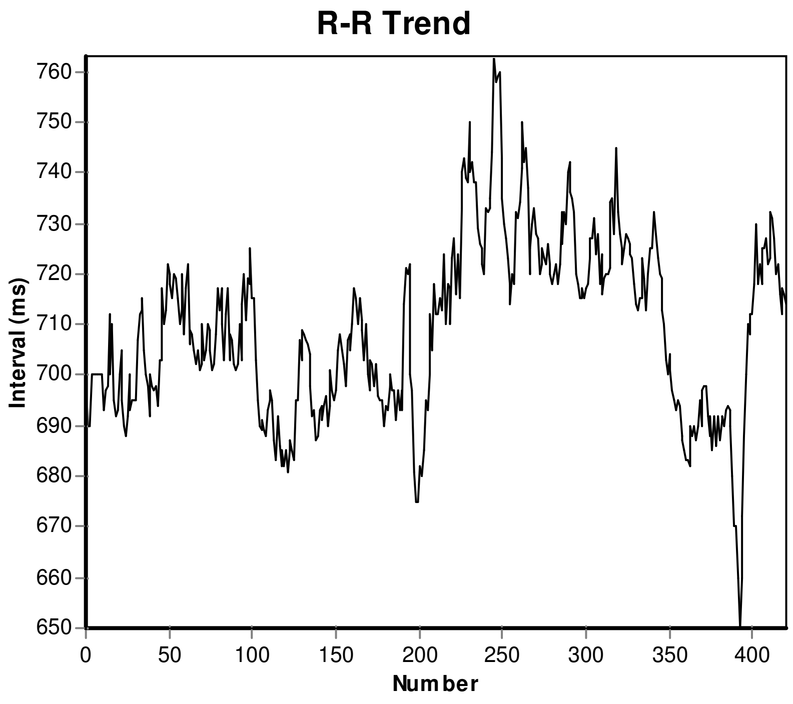

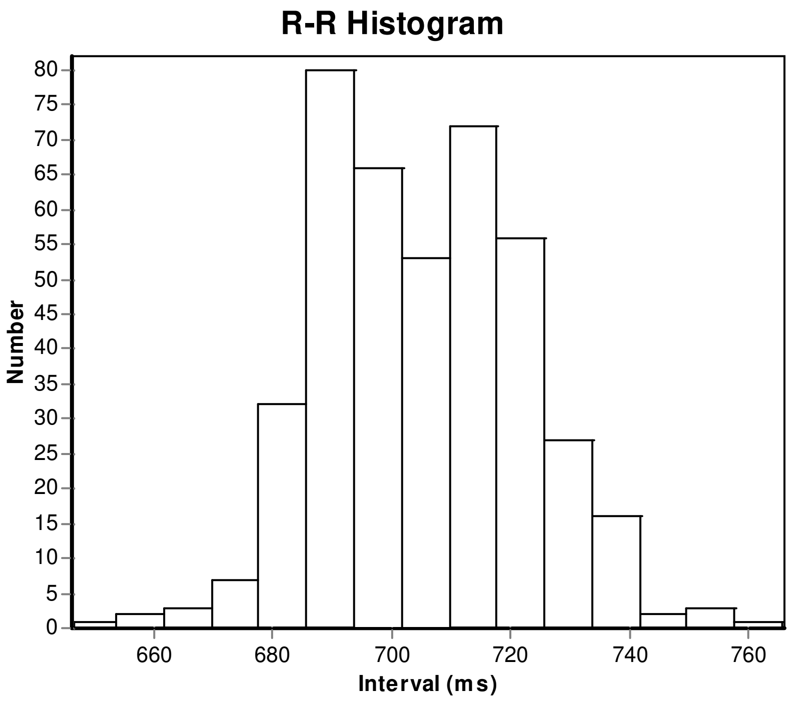

Cardio Control NV. User: Anton

Heart Rate Variability: Time Domain Analysis

**Name:**	004, 004 004	**Birthdate:**	13/11/1964
**Number:**	004	**Recorded:**	04/05/2018 12:38:28
**Gender:**	Male		

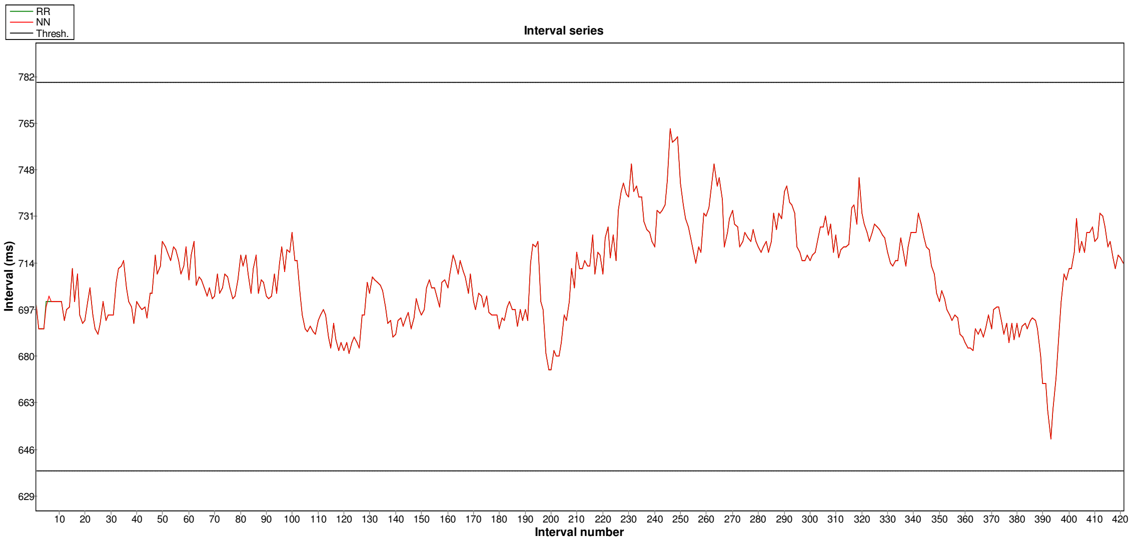

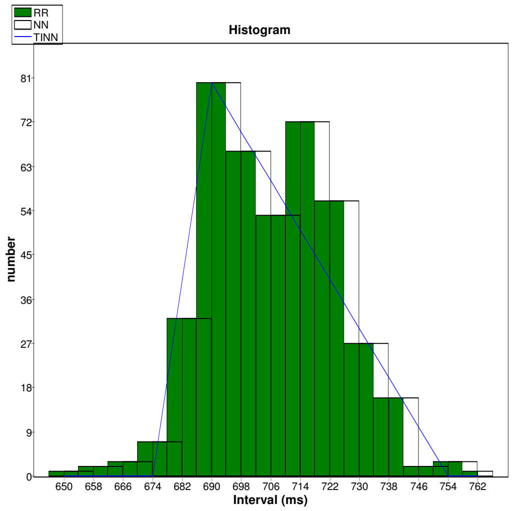

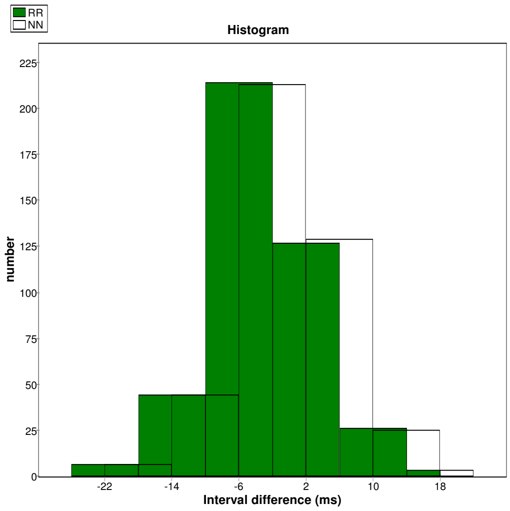

Binsize (ms) = 8

HRV parameters	NN	RR
SDNN (ms)	17	17
Triangular Interpolation (ms)	80	80
Triangular Index	5.26	5.26

HRV parameters	NN	RR
SDSD (ms)	6	6
RMSSD (ms)	6	6
NN50	0	0
NN50(1)	0	0
NN50(2)	0	0
pNN50	0.00	0.00
pNN50(1)	0.00	0.00
pNN50(2)	0.00	0.00
Logarithmic Index	1.76	1.76
SD(Logarithmic Index)	0.14	0.14

Interval statistics	NN	RR
Number	421	421
Minimum (ms)	650	650
Maximum (ms)	763	763
Range (ms)	113	113
Avg (ms)	709	709
SD (ms)	17	17
AvgDev (ms)	14	14
p5 (ms)	685	685
p50 (ms)	710	710
p95 (ms)	738	738
Skewness	0.12	0.12
Kurtosis	3.07	3.07

Interval statistics	NN	RR
Number	420	420
Minimum (ms)	-22	-22
Maximum (ms)	21	21
Range (ms)	43	43
Avg (ms)	0	0
SD (ms)	6	6
AvgDev (ms)	5	5
p5 (ms)	-10	-10
p50 (ms)	-1	0
p95 (ms)	11	11
Skewness	0.13	0.14
Kurtosis	3.61	3.61

Cardio Control NV. User: Anton

Heart Rate Variability: Frequency Domain Analysis

**Name:**	004, 004 004	**Birthdate:**	13/11/1964
**Number:**	004	**Recorded:**	04/05/2018 12:38:28
**Gender:**	Male		

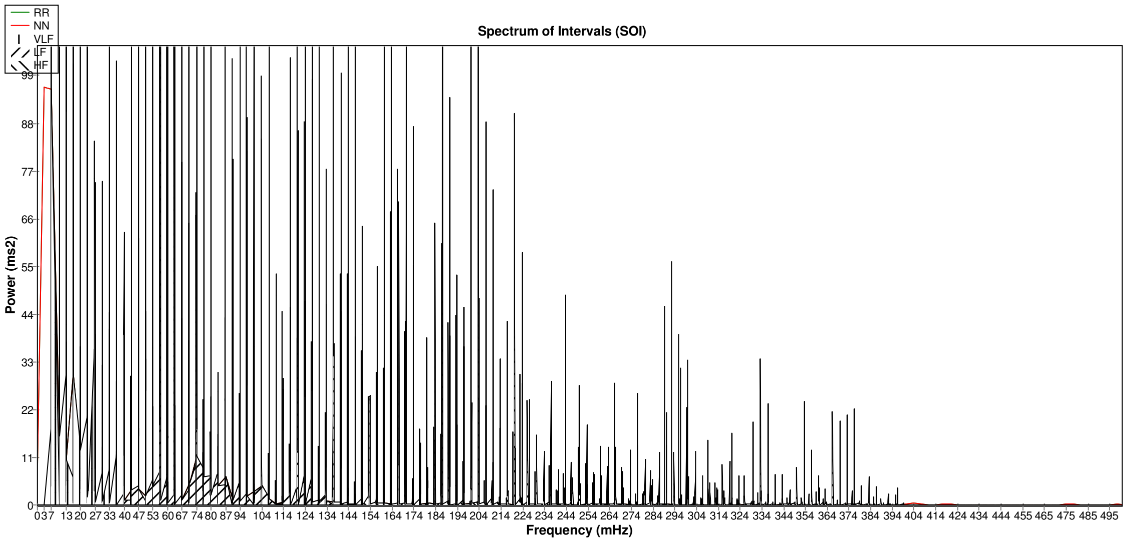

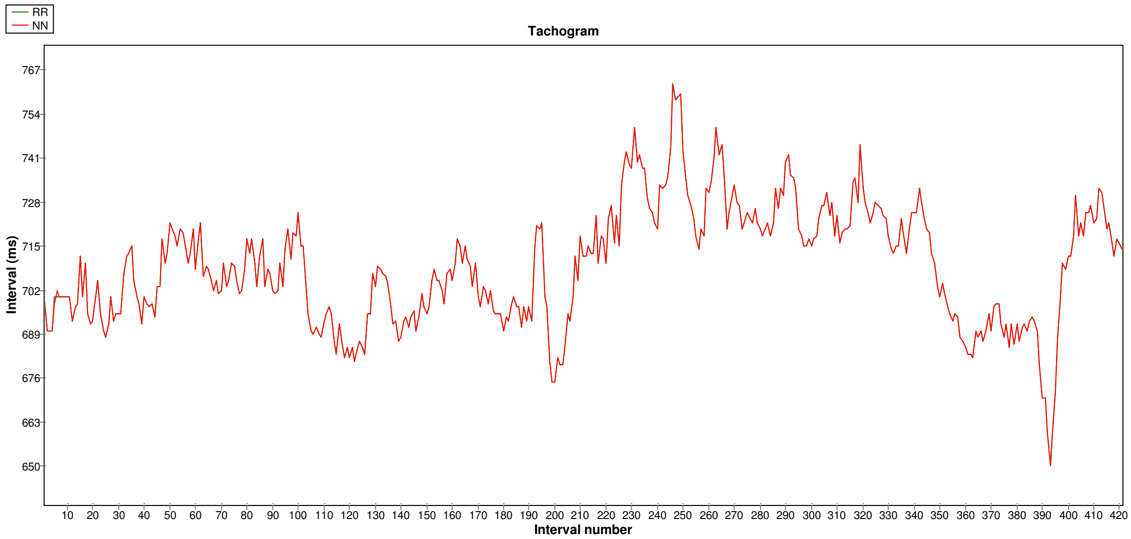

**HRV parameters**	**NN**	**RR**
TP (ms2)	253	253
VLF (ms2)	170	170
LF (ms2)	74	74
HF (ms2)	9	9
LF/HF	8.25	8.25
LF normalized	89.19	89.19
HF normalized	10.81	10.81
VLF peak (mHz)	7	7
LF peak (mHz)	74	74
HF peak (mHz)	160	160

**HRV spectral settings**	
Spectrum of Intervals (SOI)	
Frequency resolution (mHz)	3
VLF lower boundary (mHz)	3
VLF upper boundary (mHz)	40
LF upper boundary (mHz)	150
HF upper boundary (mHz)	400
Smoothing factor	1
Tapering	Hann
Fourier transform	DFT
Sample frequency (Hz)	1.41
Interval correction	Annotation
Interval threshold (%)	10
