# Supplementary material for: The effects of a 3-day mountain bike cycling race on the autonomic nervous system (ANS) and heart rate variability in amateur cyclists: a prospective quantitative research design
Source: BMC Sports Sci Med Rehabil. 2023 Jan 2;15:2. doi: 10.1186/s13102-022-00614-y (PMC9808932; doi:10.1186/s13102-022-00614-y)
Supplement: Supplementary file 1 — Additional file 1. Individual data of Participants. [file 13102_2022_614_MOESM1_ESM.zip › Individual data of Participants/HRV Data/004/ECG_004_20180505121848_.PDF]

Anton Swart Biokinetic Rehabilitation Practice

Name:	004 004 004	Recorded:	05/05/2018 12:18:48	UNCONFIRMED INTERPRETATION - MD SHOULD REVIEW
Number:	004	Recorded by:	Mr. Anton Swart	
Gender:	Male	Referring physician:		
Birthdate:	13/11/1964 53 years	Ordering physician:		
		Attending physician:		
P / PQ:	113 ms / 143 ms	Location:	Anton Swart Biokinetic Rehabilitation Practi	
QRS:	109 ms	Comment:		
QT / QTc / QTd:	404 ms / 453 ms / -			
P/QRS/T axis:	71° / 81° / 78°			
Heartrate:	88 bpm			

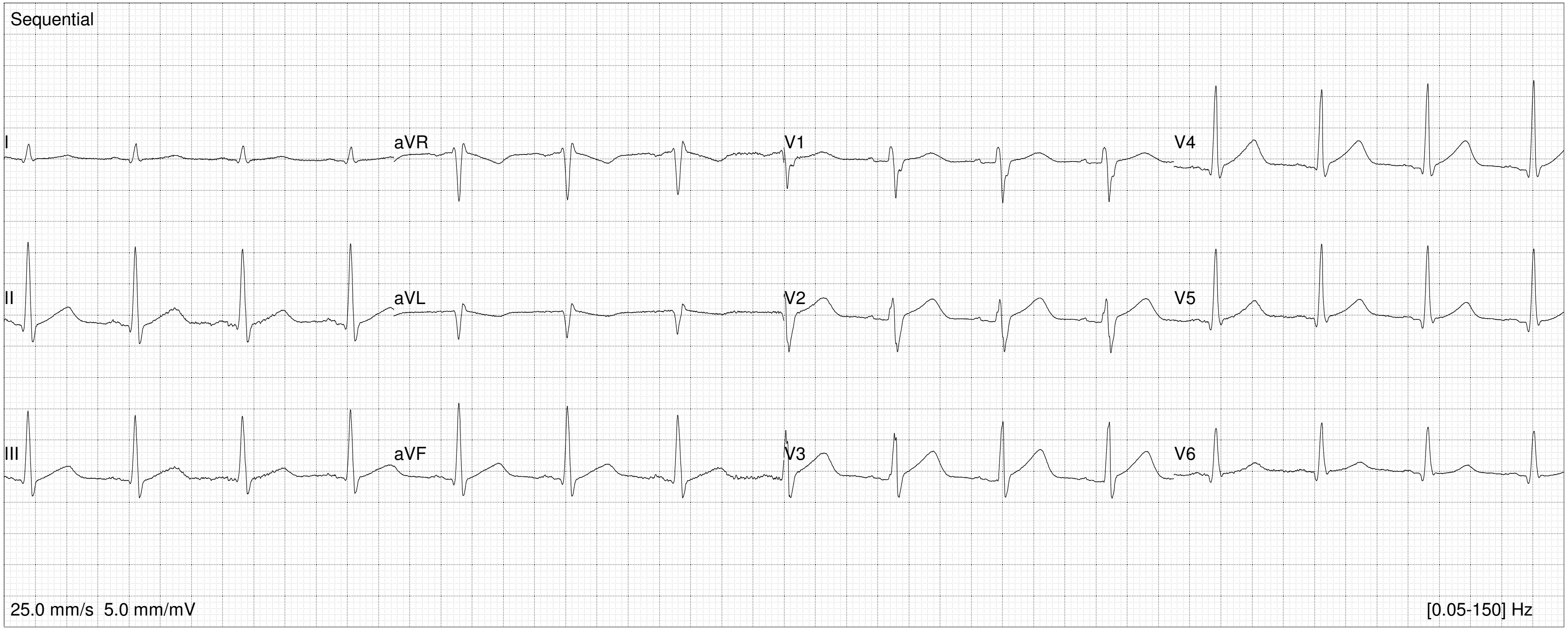

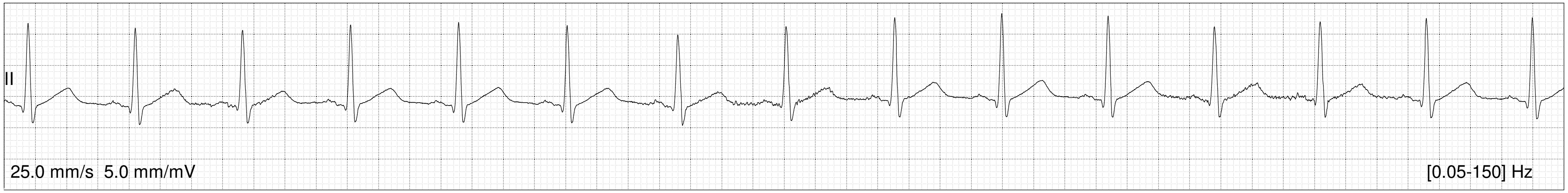

Anton Swart Biokinetic Rehabilitation Practice

Name:	004 004 004	Recorded:	05/05/2018 12:18:48	UNCONFIRMED INTERPRETATION - MD SHOULD REVIEW
Number:	004	Recorded by:	Mr. Anton Swart	
Gender:	Male	Referring physician:		
Birthdate:	13/11/1964 53 years	Location:	Anton Swart Biokinetic Rehabilitation Practice	
P / PQ:	113 ms / 143 ms	Ordering physician:		
QRS:	109 ms	Attending physician:		
QT / QTc / QTd:	404 ms / 453 ms / -	Comment:		
P/QRS/T axis:	71° / 81° / 78°			
Heartrate:	88 bpm			

Beats

Total:	443
Normal:	443
Other:	0

RR

Minimum	630 ms
Maximum	715 ms
Mean:	676 ms
SD:	16 ms

R-R Trend

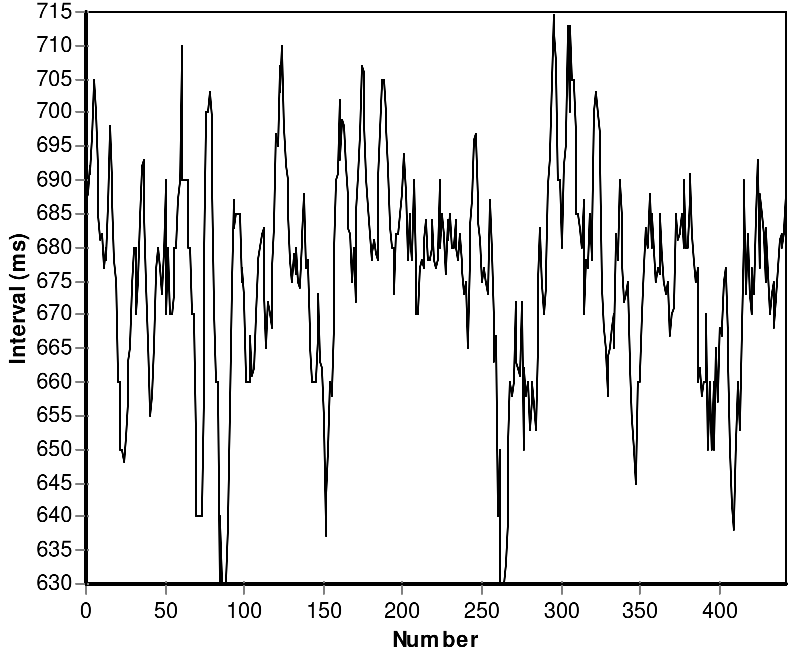

R-R Histogram

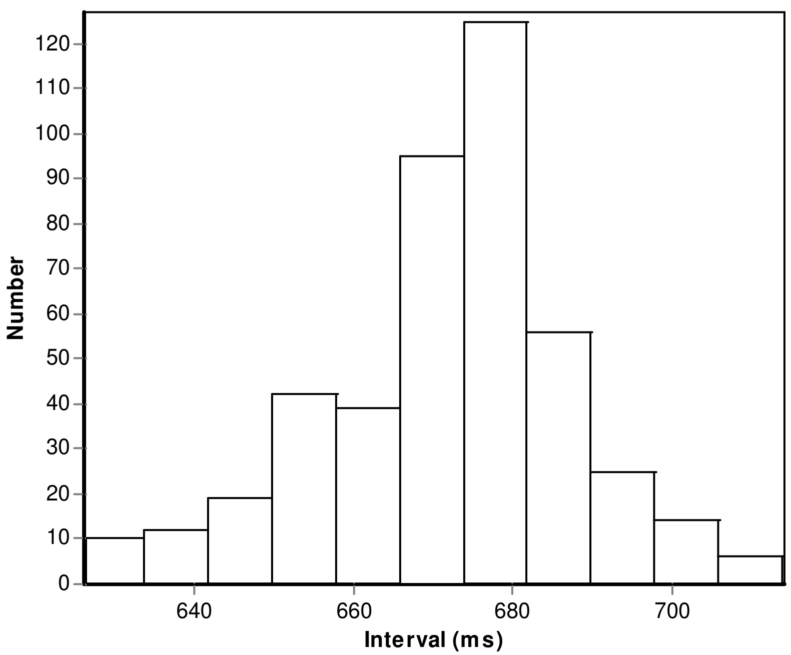

Heart Rate Variability: Time Domain Analysis

**Name:**	004, 004 004	**Birthdate:**	13/11/1964
**Number:**	004	**Recorded:**	05/05/2018 12:18:48
**Gender:**	Male		

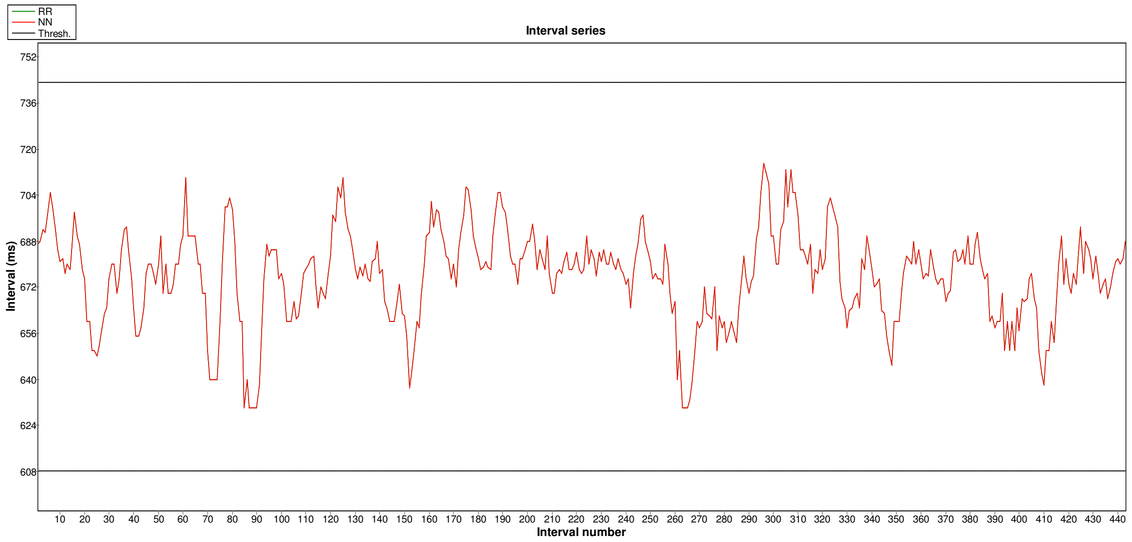

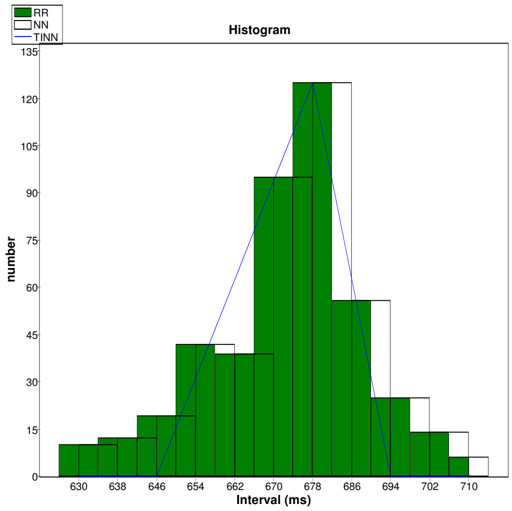

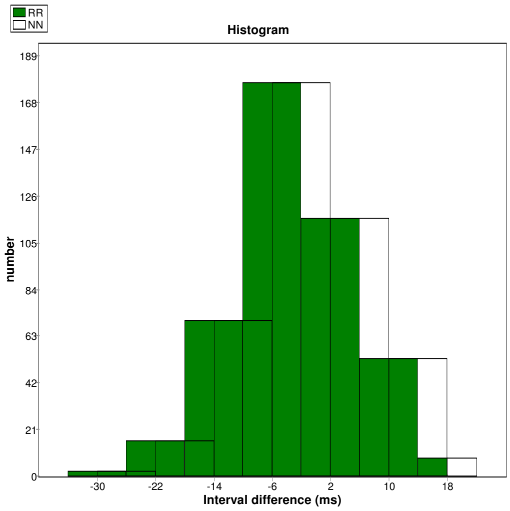

Binsize (ms) = 8

**HRV parameters**	**NN**	**RR**
SDNN (ms)	16	16
Triangular Interpolation (ms)	48	48
Triangular Index	3.54	3.54

**HRV parameters**	**NN**	**RR**
SDSD (ms)	8	8
RMSSD (ms)	8	8
NN50	0	0
NN50(1)	0	0
NN50(2)	0	0
pNN50	0.00	0.00
pNN50(1)	0.00	0.00
pNN50(2)	0.00	0.00
Logarithmic Index	1.66	1.66
SD(Logarithmic Index)	0.25	0.25

**Interval statistics**	**NN**	**RR**
Number	443	443
Minimum (ms)	630	630
Maximum (ms)	715	715
Range (ms)	85	85
Avg (ms)	676	676
SD (ms)	16	16
AvgDev (ms)	12	12
p5 (ms)	648	648
p50 (ms)	678	678
p95 (ms)	700	700
Skewness	-0.50	-0.50
Kurtosis	3.56	3.56

**Interval statistics**	**NN**	**RR**
Number	442	442
Minimum (ms)	-30	-30
Maximum (ms)	20	20
Range (ms)	50	50
Avg (ms)	0	0
SD (ms)	8	8
AvgDev (ms)	6	6
p5 (ms)	-12	-12
p50 (ms)	0	0
p95 (ms)	12	12
Skewness	-0.20	-0.20
Kurtosis	3.46	3.46

Heart Rate Variability: Frequency Domain Analysis

**Name:**	004, 004 004	**Birthdate:**	13/11/1964
**Number:**	004	**Recorded:**	05/05/2018 12:18:48
**Gender:**	Male		

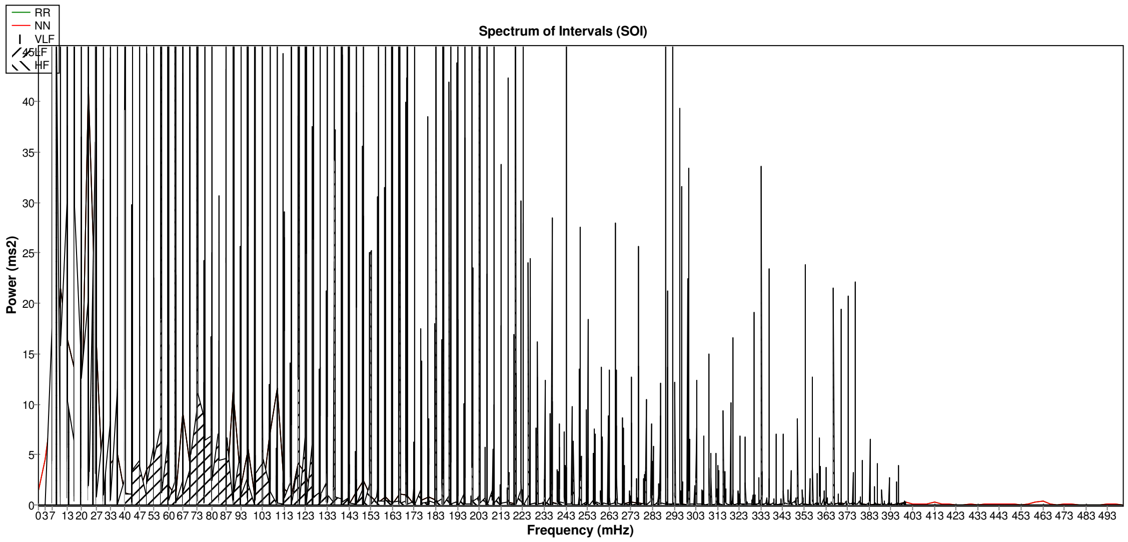

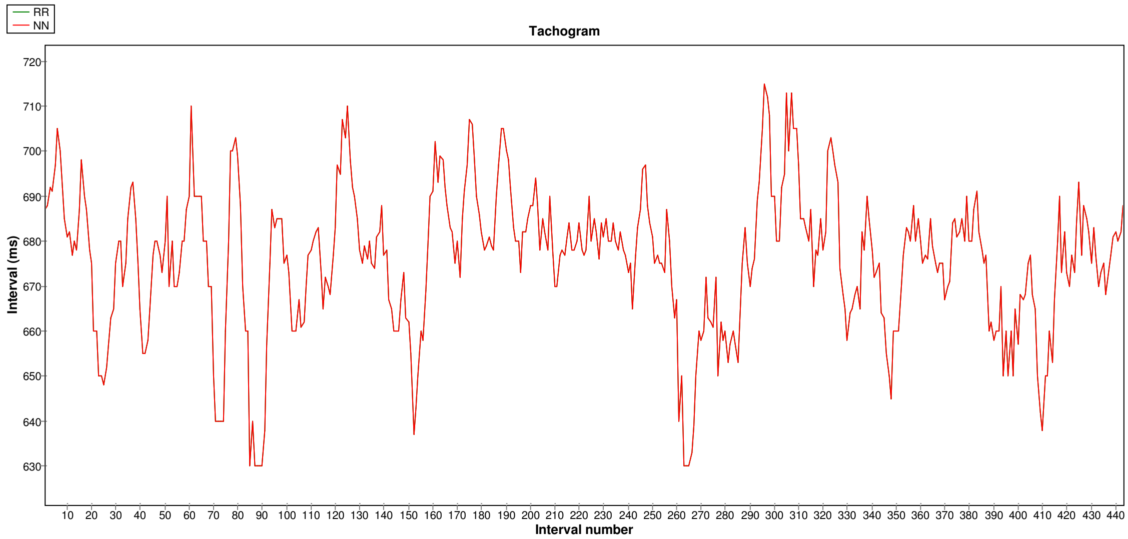

HRV parameters	NN	RR
TP (ms2)	237	237
VLF (ms2)	136	136
LF (ms2)	89	89
HF (ms2)	12	12
LF/HF	7.53	7.53
LF normalized	88.27	88.27
HF normalized	11.73	11.73
VLF peak (mHz)	23	23
LF peak (mHz)	110	110
HF peak (mHz)	167	167

HRV spectral settings	
Spectrum of Intervals (SOI)	
Frequency resolution (mHz)	3
VLF lower boundary (mHz)	3
VLF upper boundary (mHz)	40
LF upper boundary (mHz)	150
HF upper boundary (mHz)	400
Smoothing factor	1
Tapering	Hann
Fourier transform	DFT
Sample frequency (Hz)	1.48
Interval correction	Annotation
Interval threshold (%)	10
