# Supplementary material for: The effects of a 3-day mountain bike cycling race on the autonomic nervous system (ANS) and heart rate variability in amateur cyclists: a prospective quantitative research design
Source: BMC Sports Sci Med Rehabil. 2023 Jan 2;15:2. doi: 10.1186/s13102-022-00614-y (PMC9808932; doi:10.1186/s13102-022-00614-y)
Supplement: Supplementary file 1 — Additional file 1. Individual data of Participants. [file 13102_2022_614_MOESM1_ESM.zip › Individual data of Participants/HRV Data/004/ECG_004_20180506080101_.PDF]

Anton Swart Biokinetic Rehabilitation Practice

Name: 004 004 004
Number: 004
Gender: Male
Birthdate: 13/11/1964 53 years

P / PQ: 125 ms / 167 ms
QRS: 110 ms
QT / QTc / QTd: 475 ms / 484 ms / -
P/QRS/T axis: 75° / 81° / 64°
Heartrate: 65 bpm

Recorded: 06/05/2018 08:01:01
Recorded by: Mr. Anton Swart
Referring physician:
Ordering physician:
Attending physician:
Location: Anton Swart Biokinetic Rehabilitation Practi
Comment:

UNCONFIRMED INTERPRETATION - MD SHOULD REVIEW

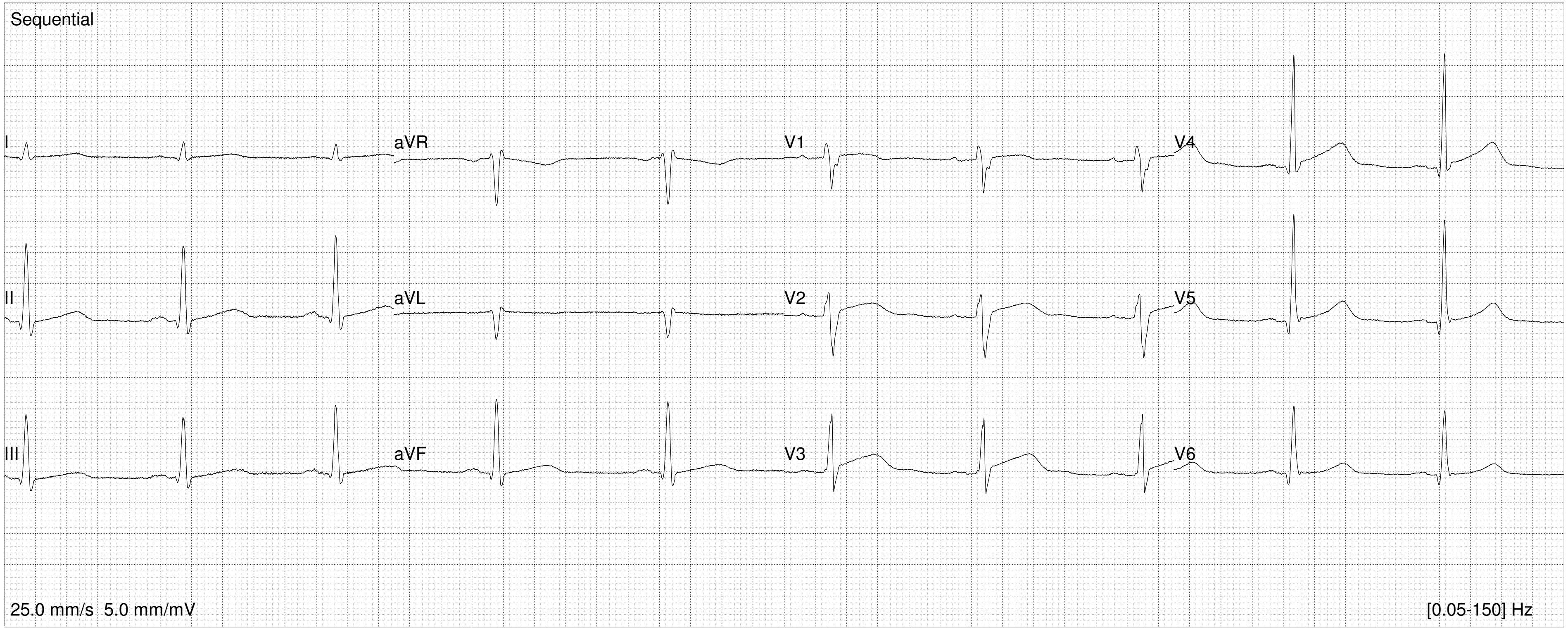

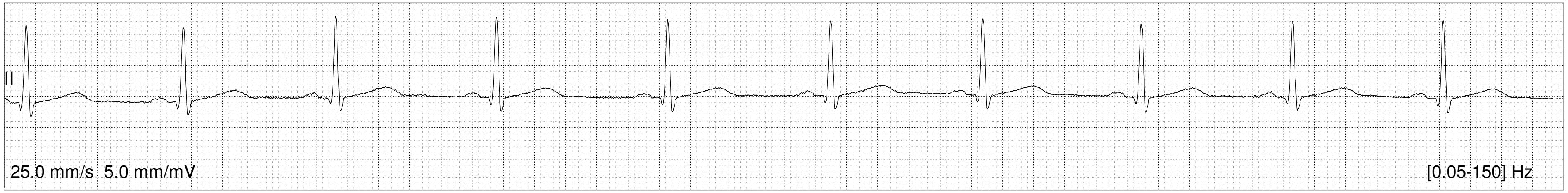

Anton Swart Biokinetic Rehabilitation Practice

Name:	004 004 004	Recorded:	06/05/2018 08:01:01	UNCONFIRMED INTERPRETATION - MD SHOULD REVIEW
Number:	004	Recorded by:	Mr. Anton Swart	
Gender:	Male	Referring physician:		
Birthdate:	13/11/1964 53 years	Location:	Anton Swart Biokinetic Rehabilitation Practice	
P / PQ:	125 ms / 167 ms	Ordering physician:		
QRS:	110 ms	Attending physician:		
QT / QTc / QTd:	475 ms / 484 ms / -	Comment:		
P/QRS/T axis:	75° / 81° / 64°			
Heartrate:	65 bpm			

**Beats**		**RR**	
Total:	319	Minimum	835 ms
Normal:	319	Maximum	1100 ms
Other:	0	Mean:	937 ms
		SD:	35 ms

R-R Trend

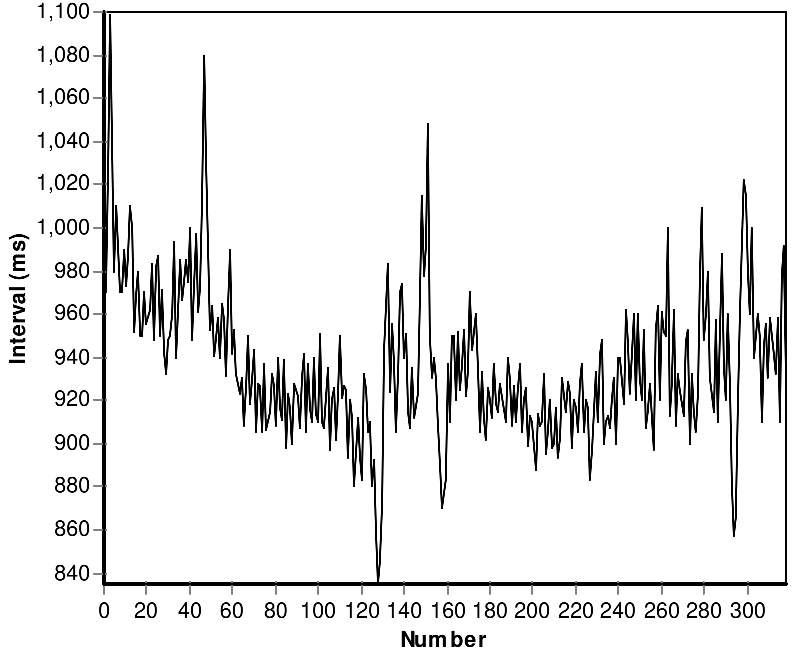

R-R Histogram

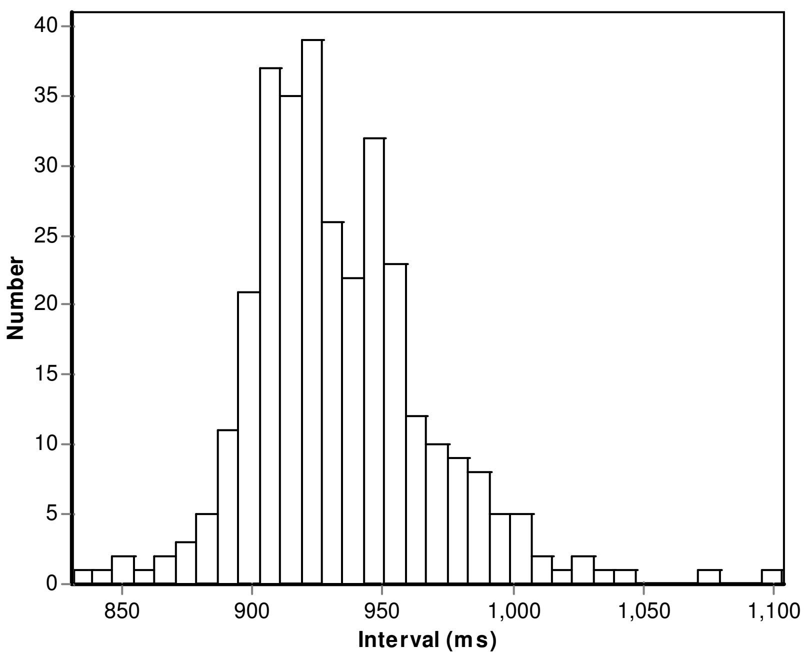

Cardio Control NV. User: Anton

Heart Rate Variability: Time Domain Analysis

**Name:**	004, 004 004	**Birthdate:**	13/11/1964
**Number:**	004	**Recorded:**	06/05/2018 08:01:01
**Gender:**	Male		

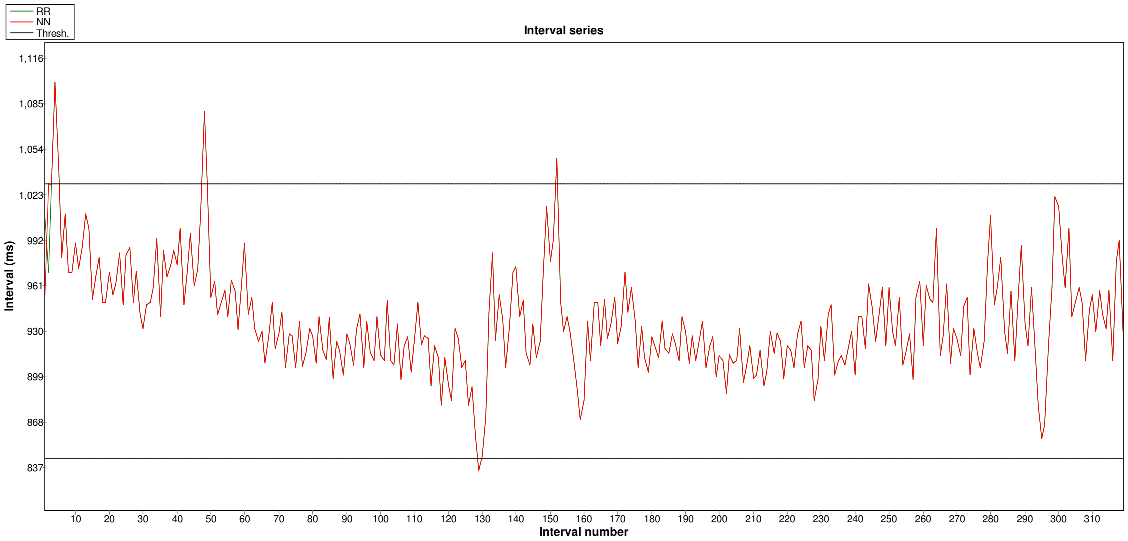

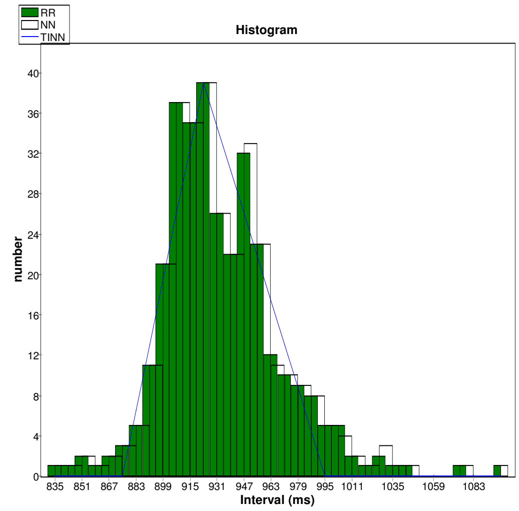

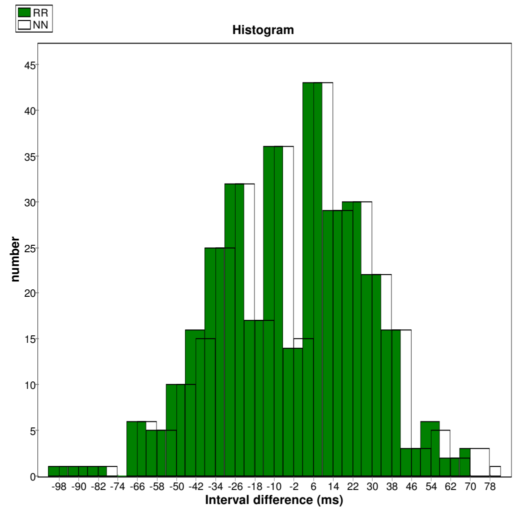

Binsize (ms) = 8

**HRV parameters**	**NN**	**RR**
SDNN (ms)	35	35
Triangular Interpolation (ms)	120	120
Triangular Index	8.18	8.18

**HRV parameters**	**NN**	**RR**
SDSD (ms)	30	30
RMSSD (ms)	30	30
NN50	25	25
NN50(1)	14	14
NN50(2)	11	11
pNN50	0.08	0.08
pNN50(1)	0.04	0.04
pNN50(2)	0.03	0.03
Logarithmic Index	0.42	0.44
SD(Logarithmic Index)	0.05	0.05

**Interval statistics**	**NN**	**RR**
Number	319	319
Minimum (ms)	835	835
Maximum (ms)	1100	1100
Range (ms)	265	265
Avg (ms)	937	937
SD (ms)	35	35
AvgDev (ms)	27	27
p5 (ms)	892	892
p50 (ms)	931	931
p95 (ms)	1000	1000
Skewness	0.88	0.86
Kurtosis	5.24	5.21

**Interval statistics**	**NN**	**RR**
Number	318	318
Minimum (ms)	-98	-98
Maximum (ms)	80	71
Range (ms)	178	169
Avg (ms)	-0	-0
SD (ms)	30	30
AvgDev (ms)	25	25
p5 (ms)	-50	-50
p50 (ms)	2	2
p95 (ms)	45	45
Skewness	-0.15	-0.17
Kurtosis	2.84	2.76

Heart Rate Variability: Frequency Domain Analysis

**Name:**	004, 004 004	**Birthdate:**	13/11/1964
**Number:**	004	**Recorded:**	06/05/2018 08:01:01
**Gender:**	Male		

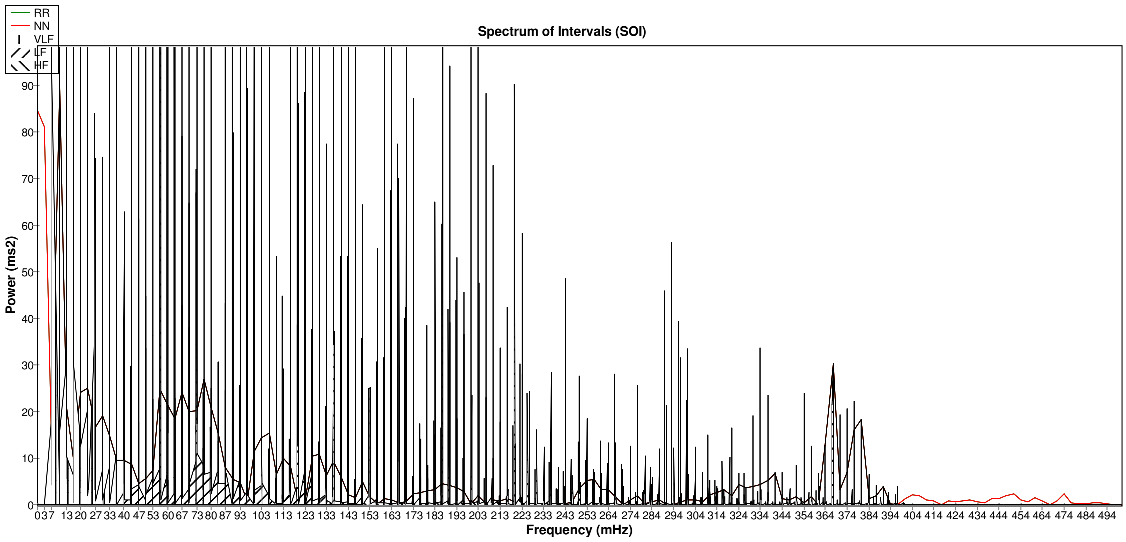

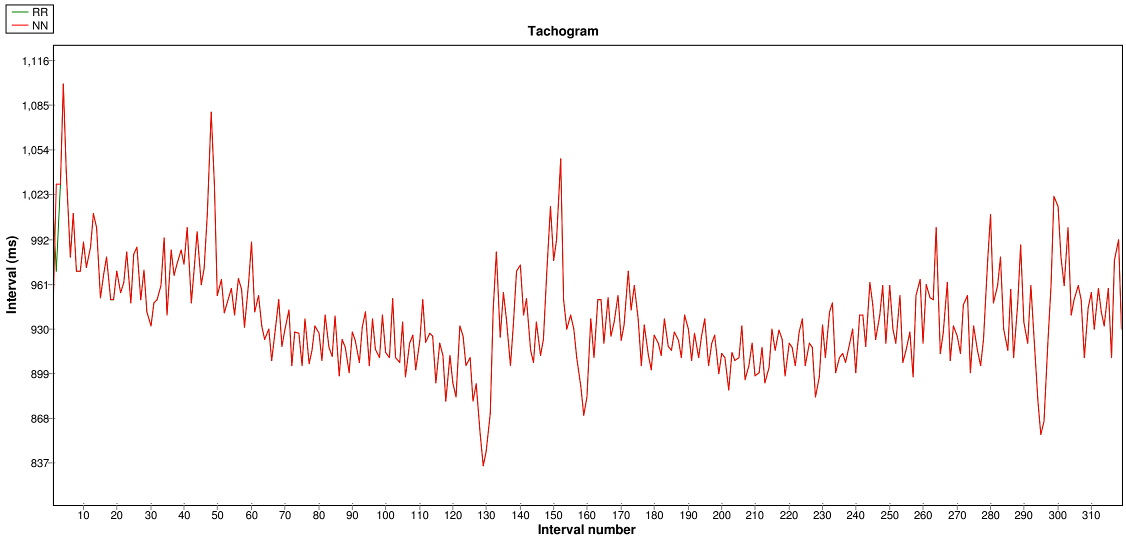

HRV parameters	NN	RR
TP (ms2)	822	822
VLF (ms2)	254	254
LF (ms2)	358	358
HF (ms2)	210	210
LF/HF	1.70	1.70
LF normalized	62.99	62.99
HF normalized	37.01	37.01
VLF peak (mHz)	10	10
LF peak (mHz)	77	77
HF peak (mHz)	367	367

HRV spectral settings	
Spectrum of Intervals (SOI)	
Frequency resolution (mHz)	3
VLF lower boundary (mHz)	3
VLF upper boundary (mHz)	40
LF upper boundary (mHz)	150
HF upper boundary (mHz)	400
Smoothing factor	1
Tapering	Hann
Fourier transform	DFT
Sample frequency (Hz)	1.07
Interval correction	Annotation
Interval threshold (%)	10
