# Supplementary material for: The effects of a 3-day mountain bike cycling race on the autonomic nervous system (ANS) and heart rate variability in amateur cyclists: a prospective quantitative research design
Source: BMC Sports Sci Med Rehabil. 2023 Jan 2;15:2. doi: 10.1186/s13102-022-00614-y (PMC9808932; doi:10.1186/s13102-022-00614-y)
Supplement: Supplementary file 1 — Additional file 1. Individual data of Participants. [file 13102_2022_614_MOESM1_ESM.zip › Individual data of Participants/HRV Data/005/ECG_005_20180501081120_.PDF]

Anton Swart Biokinetic Rehabilitation Practice

Name: 005 005 005
Number: 005
Gender: Male
Birthdate: 16/06/1977 40 years

Recorded: 01/05/2018 08:11:20
Recorded by: Mr. Anton Swart
Referring physician:
Ordering physician:
Attending physician:
Location: Anton Swart Biokinetic Rehabilitation Practi
Comment:

UNCONFIRMED INTERPRETATION - MD SHOULD REVIEW

P / PQ: 120 ms / 165 ms
QRS: 92 ms
QT / QTc / QTd: 493 ms / 472 ms / -
P/QRS/T axis: 73° / -51° / 60°
Heartrate: 48 bpm

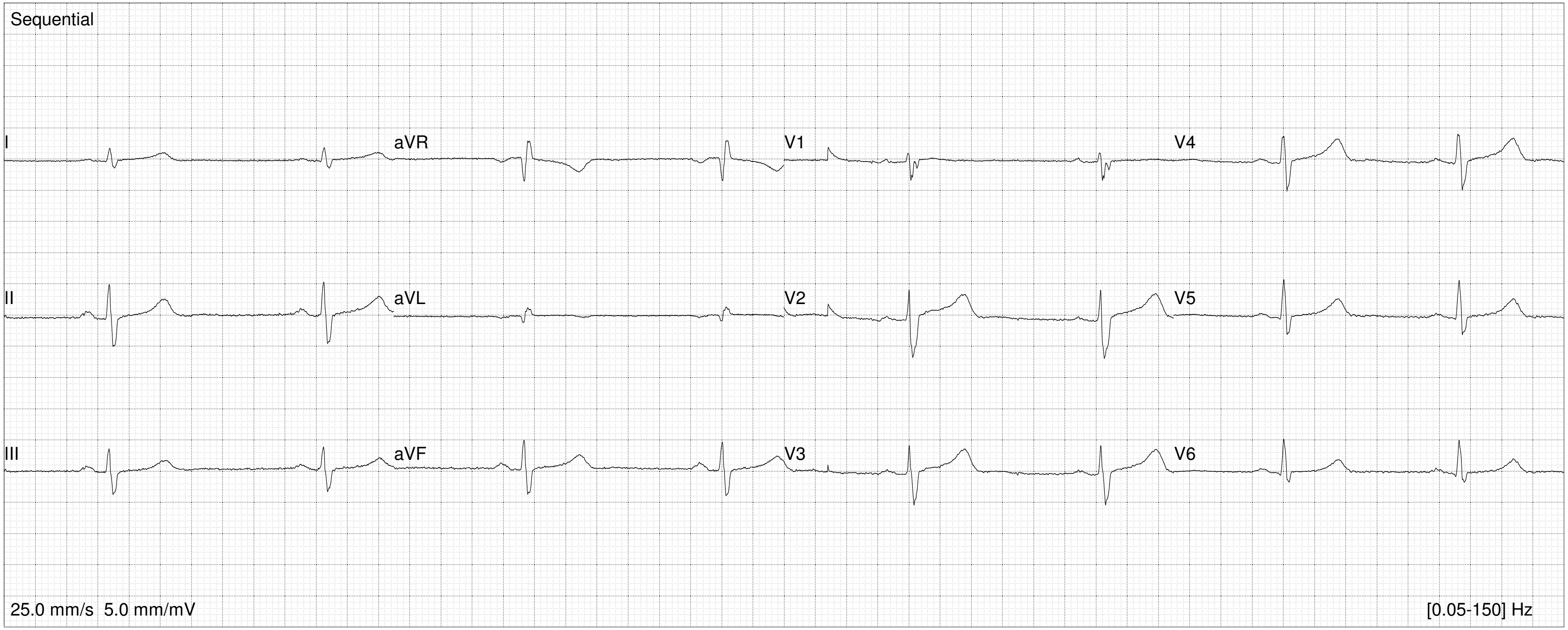

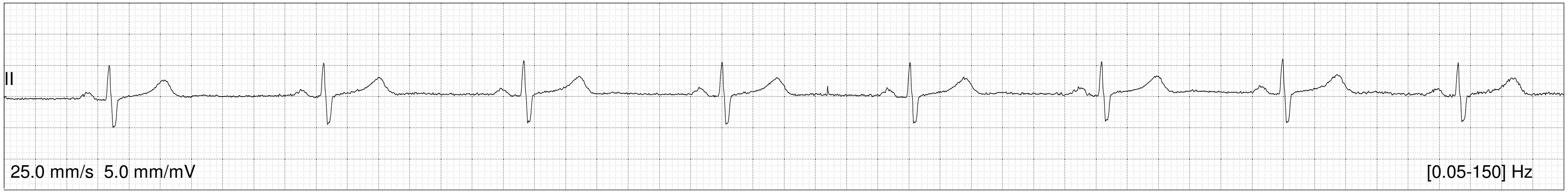

Anton Swart Biokinetic Rehabilitation Practice

Name:	005 005 005	Recorded:	01/05/2018 08:11:20	UNCONFIRMED INTERPRETATION - MD SHOULD REVIEW
Number:	005	Recorded by:	Mr. Anton Swart	
Gender:	Male	Referring physician:		
Birthdate:	16/06/1977 40 years	Location:	Anton Swart Biokinetic Rehabilitation Practice	
P / PQ:	120 ms / 165 ms	Ordering physician:		
QRS:	92 ms	Attending physician:		
QT / QTc / QTd:	493 ms / 472 ms / -	Comment:		
P/QRS/T axis:	73° / -51° / 60°			
Heartrate:	48 bpm			

Beats

Total:	242
Normal:	242
Other:	0

RR

Minimum	760 ms
Maximum	1420 ms
Mean:	1235 ms
SD:	70 ms

R-R Trend

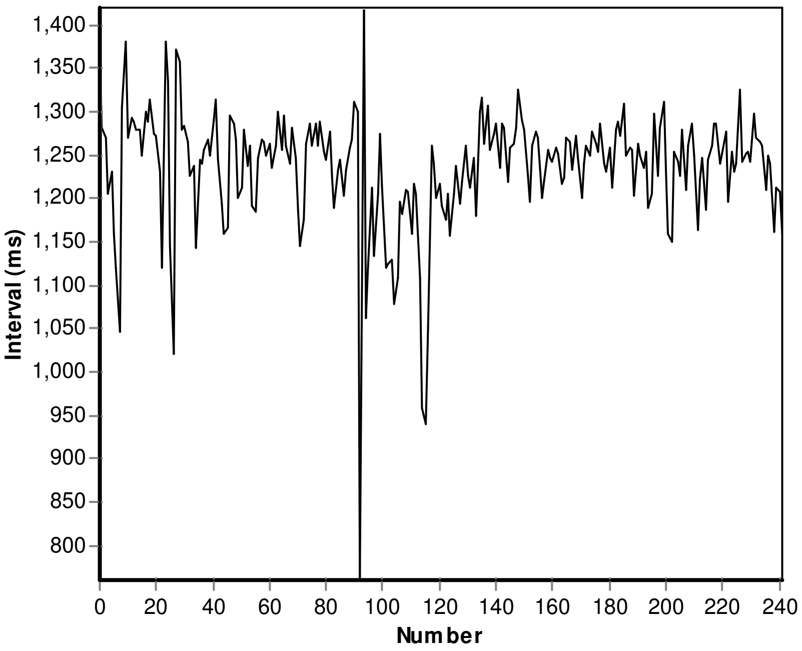

R-R Histogram

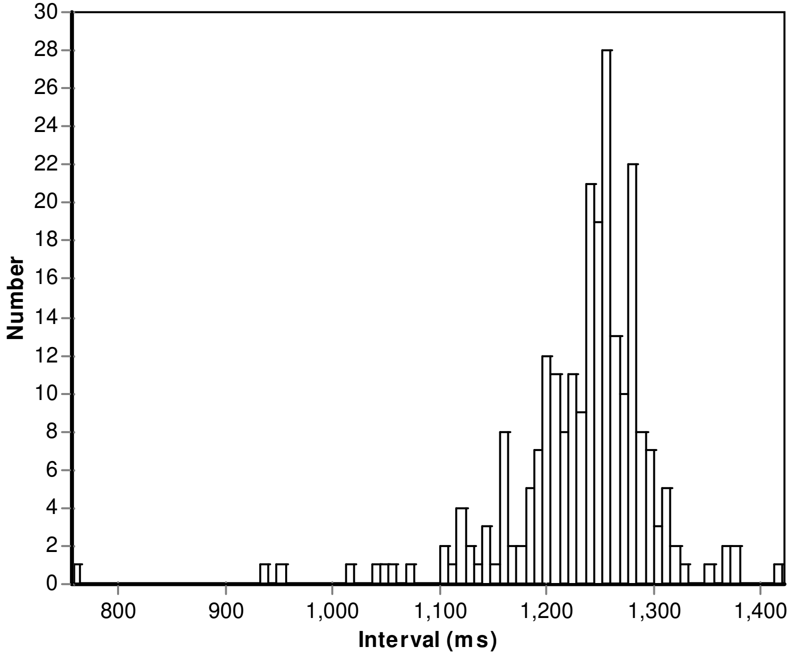

Cardio Control NV. User: Anton

Heart Rate Variability: Time Domain Analysis

**Name:**	005, 005 005	**Birthdate:**	16/06/1977
**Number:**	005	**Recorded:**	01/05/2018 08:11:20
**Gender:**	Male		

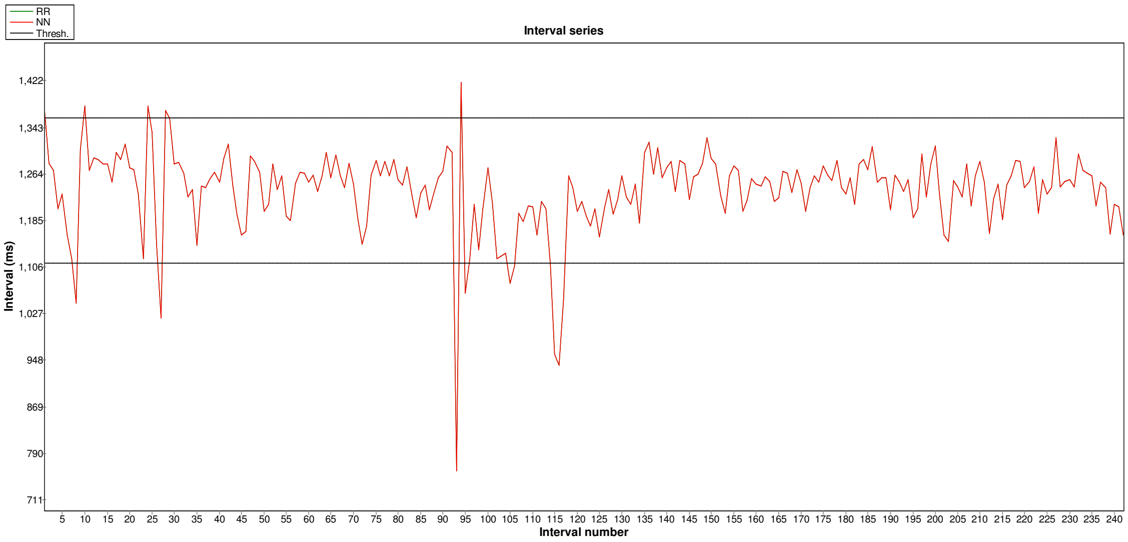

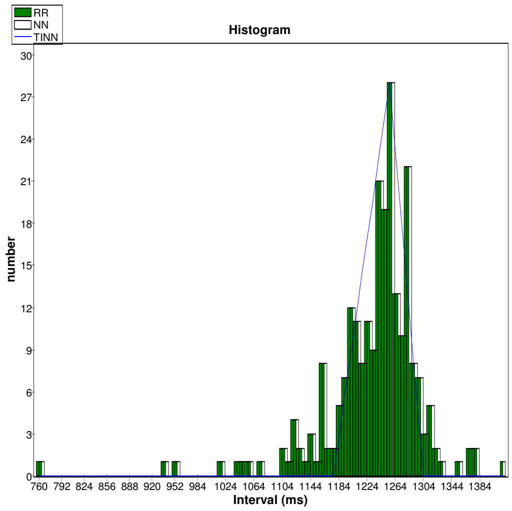

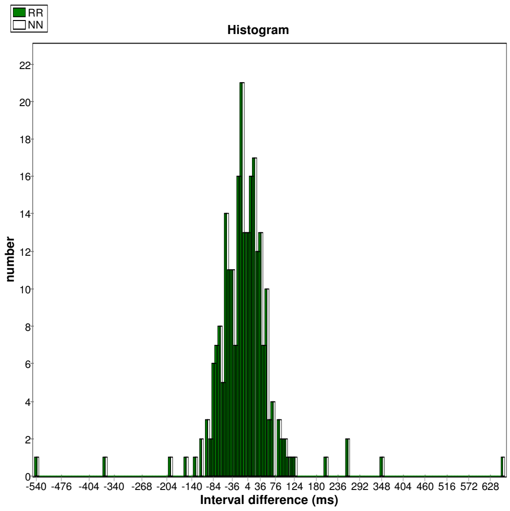

Binsize (ms) = 8

**HRV parameters**	**NN**	**RR**
SDNN (ms)	70	70
Triangular Interpolation (ms)	128	128
Triangular Index	8.64	8.64

**HRV parameters**	**NN**	**RR**
SDSD (ms)	85	85
RMSSD (ms)	85	85
NN50	73	73
NN50(1)	41	41
NN50(2)	32	32
pNN50	0.30	0.30
pNN50(1)	0.17	0.17
pNN50(2)	0.13	0.13
Logarithmic Index	0.19	0.19
SD(Logarithmic Index)	0.01	0.01

**Interval statistics**	**NN**	**RR**
Number	242	242
Minimum (ms)	760	760
Maximum (ms)	1420	1420
Range (ms)	660	660
Avg (ms)	1235	1235
SD (ms)	70	70
AvgDev (ms)	47	47
p5 (ms)	1120	1120
p50 (ms)	1250	1250
p95 (ms)	1314	1314
Skewness	-2.16	-2.16
Kurtosis	13.43	13.43

**Interval statistics**	**NN**	**RR**
Number	241	241
Minimum (ms)	-540	-540
Maximum (ms)	660	660
Range (ms)	1200	1200
Avg (ms)	-1	-1
SD (ms)	85	85
AvgDev (ms)	49	49
p5 (ms)	-83	-83
p50 (ms)	-4	-4
p95 (ms)	92	92
Skewness	1.12	1.12
Kurtosis	26.05	26.05

Heart Rate Variability: Frequency Domain Analysis

**Name:**	005, 005 005	**Birthdate:**	16/06/1977
**Number:**	005	**Recorded:**	01/05/2018 08:11:20
**Gender:**	Male		

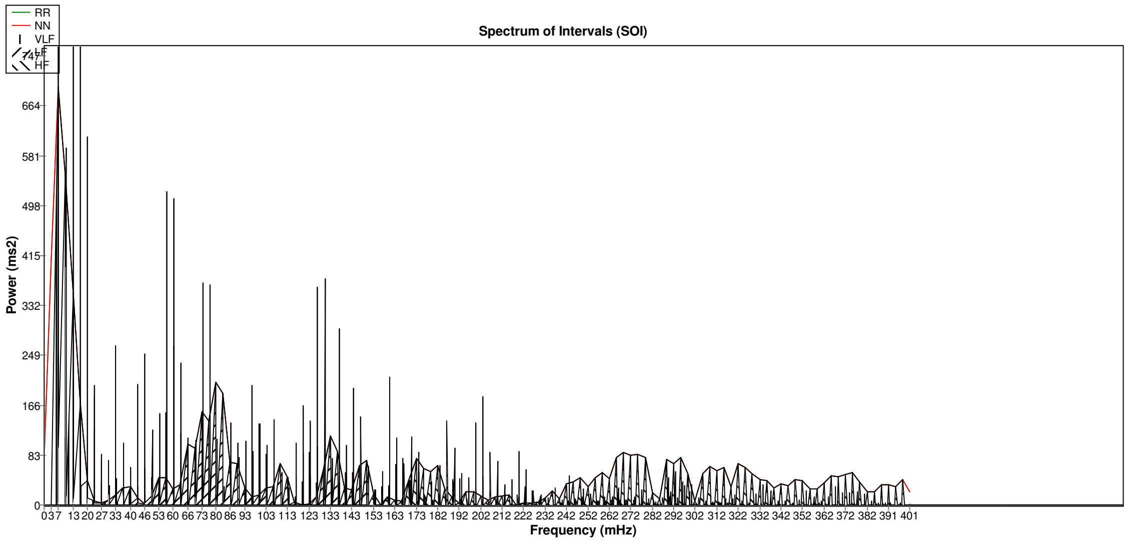

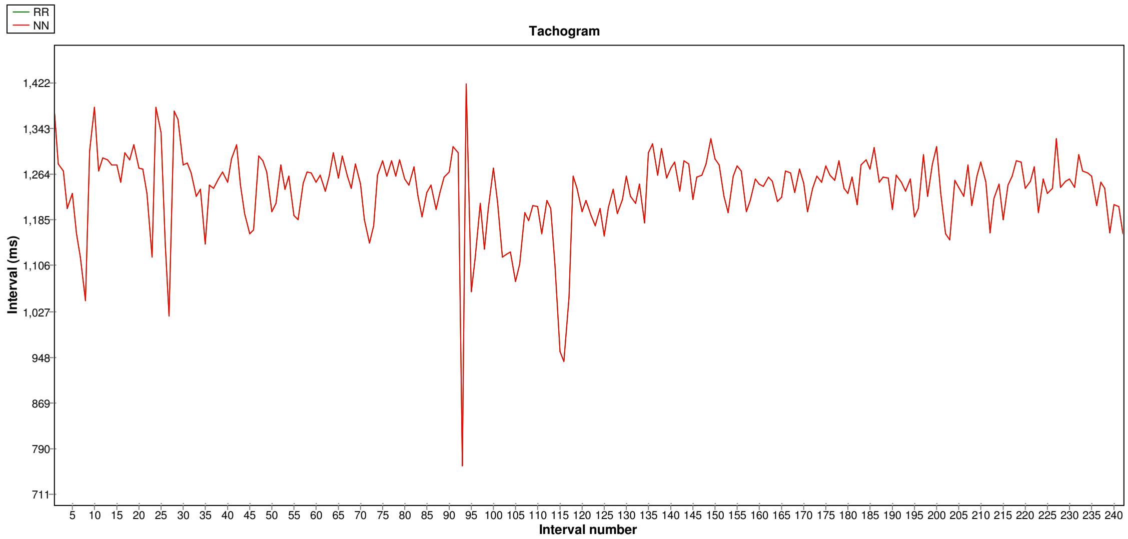

**HRV parameters**	**NN**	**RR**
TP (ms2)	6604	6604
VLF (ms2)	1895	1895
LF (ms2)	1912	1912
HF (ms2)	2797	2797
LF/HF	0.68	0.68
LF normalized	40.60	40.60
HF normalized	59.40	59.40
VLF peak (mHz)	7	7
LF peak (mHz)	80	80
HF peak (mHz)	269	269

**HRV spectral settings**	
Spectrum of Intervals (SOI)	
Frequency resolution (mHz)	3
VLF lower boundary (mHz)	3
VLF upper boundary (mHz)	40
LF upper boundary (mHz)	150
HF upper boundary (mHz)	400
Smoothing factor	1
Tapering	Hann
Fourier transform	DFT
Sample frequency (Hz)	0.81
Interval correction	Annotation
Interval threshold (%)	10
