# Supplementary material for: The effects of a 3-day mountain bike cycling race on the autonomic nervous system (ANS) and heart rate variability in amateur cyclists: a prospective quantitative research design
Source: BMC Sports Sci Med Rehabil. 2023 Jan 2;15:2. doi: 10.1186/s13102-022-00614-y (PMC9808932; doi:10.1186/s13102-022-00614-y)
Supplement: Supplementary file 1 — Additional file 1. Individual data of Participants. [file 13102_2022_614_MOESM1_ESM.zip › Individual data of Participants/HRV Data/005/ECG_005_20180503163233_.PDF]

Anton Swart Biokinetic Rehabilitation Practice

Name:	005 005 005	Recorded:	03/05/2018 16:32:33	UNCONFIRMED INTERPRETATION - MD SHOULD REVIEW
Number:	005	Recorded by:	Mr. Anton Swart	
Gender:	Male	Referring physician:		
Birthdate:	16/06/1977 40 years	Ordering physician:		
		Attending physician:		
		Location:	Anton Swart Biokinetic Rehabilitation Practi	
		Comment:		

P / PQ:	117 ms / 168 ms
QRS:	92 ms
QT / QTc / QTd:	407 ms / 444 ms / -
P/QRS/T axis:	76° / -65° / 61°
Heartrate:	81 bpm

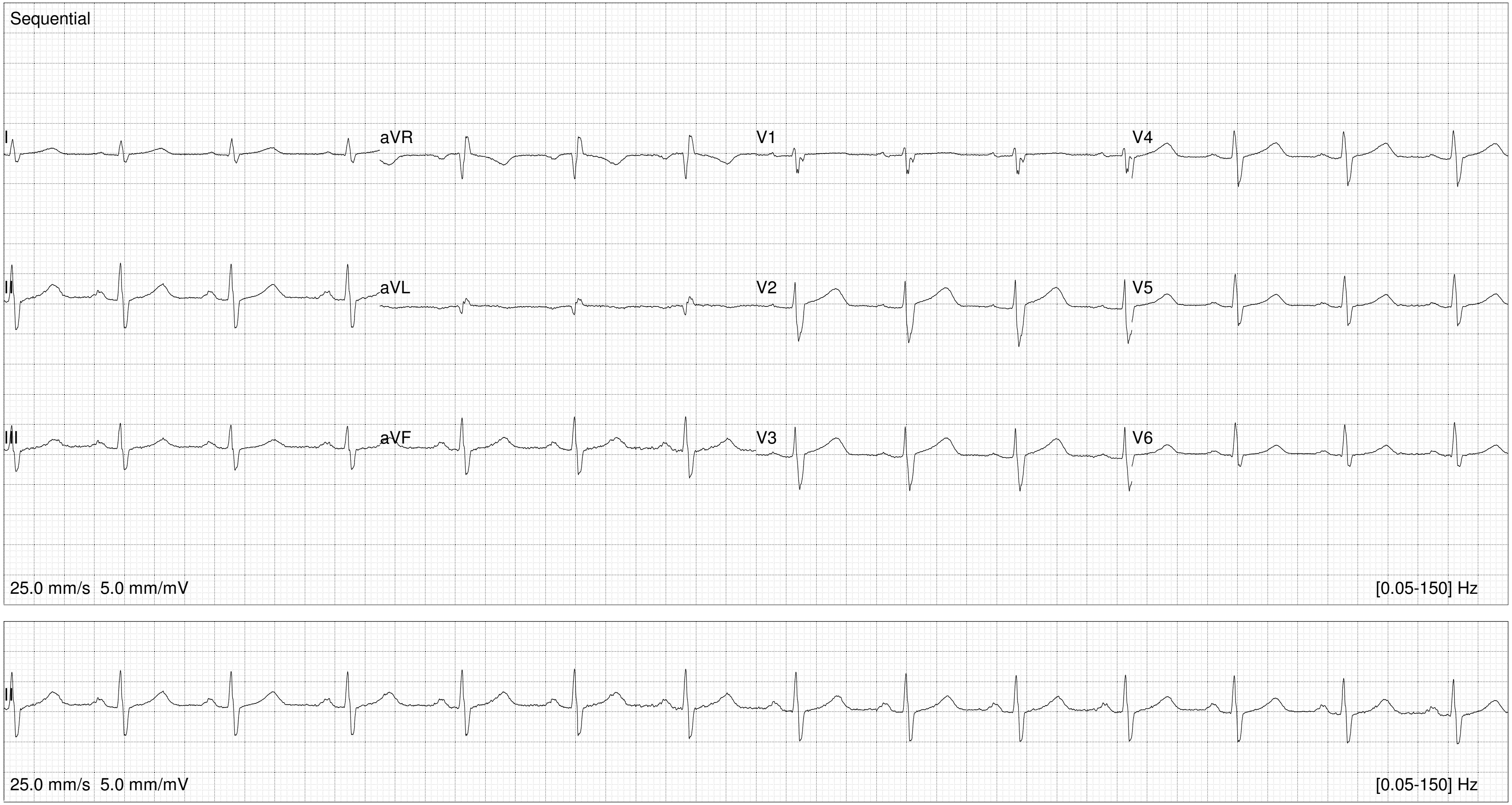

Anton Swart Biokinetic Rehabilitation Practice

Name:	005 005 005	Recorded:	03/05/2018 16:32:33	UNCONFIRMED INTERPRETATION - MD SHOULD REVIEW
Number:	005	Recorded by:	Mr. Anton Swart	
Gender:	Male	Referring physician:		
Birthdate:	16/06/1977 40 years	Location:	Anton Swart Biokinetic Rehabilitation Practice	
		Ordering physician:		
		Attending physician:		
		Comment:		

P / PQ:	117 ms / 168 ms
QRS:	92 ms
QT / QTc / QTd:	407 ms / 444 ms / -
P/QRS/T axis:	76° / -65° / 61°
Heartrate:	81 bpm

**Beats**		**RR**	
Total:	406	Minimum	667 ms
Normal:	406	Maximum	790 ms
Other:	0	Mean:	737 ms
		SD:	21 ms

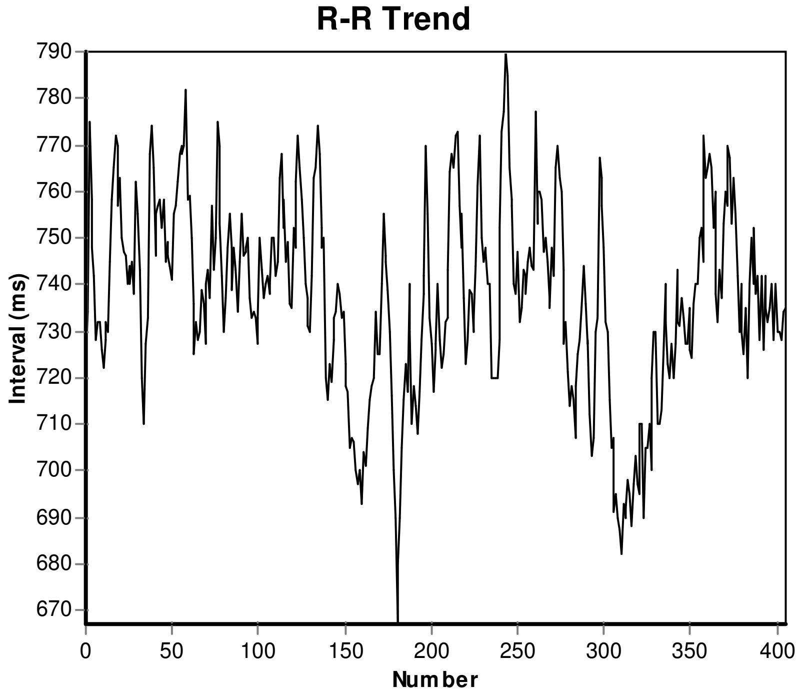

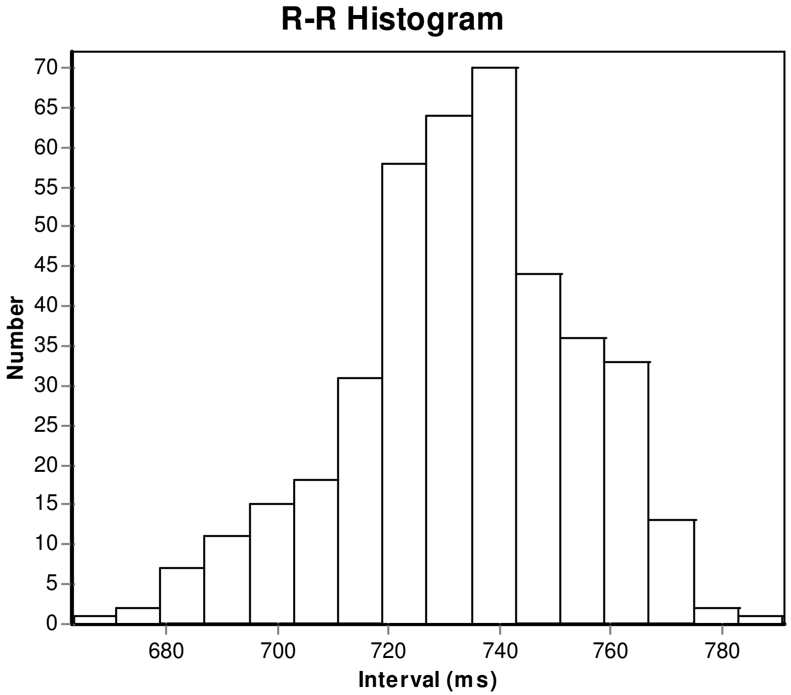

Heart Rate Variability: Time Domain Analysis

**Name:**	005, 005 005	**Birthdate:**	16/06/1977
**Number:**	005	**Recorded:**	03/05/2018 16:32:33
**Gender:**	Male		

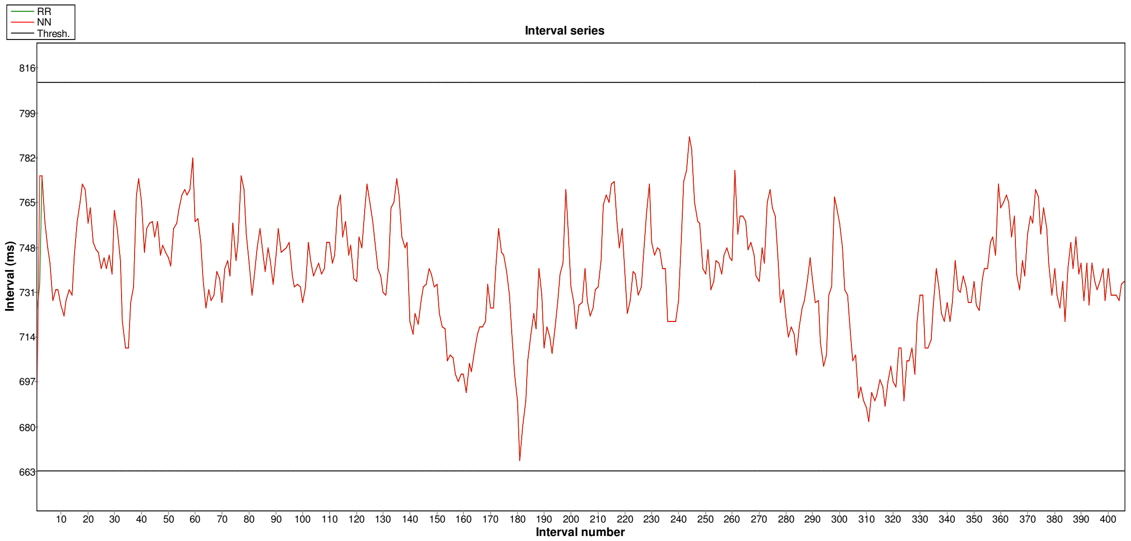

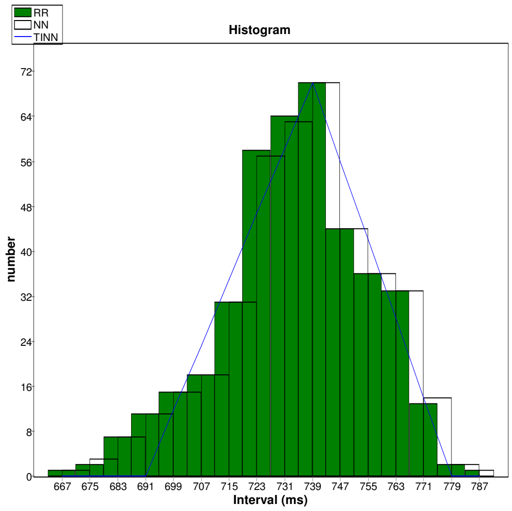

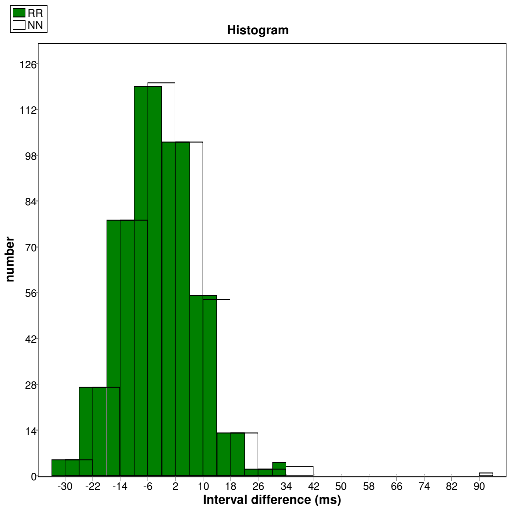

Binsize (ms) = 8

**HRV parameters**	**NN**	**RR**
SDNN (ms)	21	21
Triangular Interpolation (ms)	88	96
Triangular Index	5.80	5.80

**HRV parameters**	**NN**	**RR**
SDSD (ms)	11	11
RMSSD (ms)	11	11
NN50	1	0
NN50(1)	0	0
NN50(2)	1	0
pNN50	0.00	0.00
pNN50(1)	0.00	0.00
pNN50(2)	0.00	0.00
Logarithmic Index	1.14	1.14
SD(Logarithmic Index)	0.07	0.07

**Interval statistics**	**NN**	**RR**
Number	406	406
Minimum (ms)	667	667
Maximum (ms)	790	790
Range (ms)	123	123
Avg (ms)	737	737
SD (ms)	21	21
AvgDev (ms)	16	16
p5 (ms)	697	698
p50 (ms)	738	738
p95 (ms)	770	770
Skewness	-0.36	-0.34
Kurtosis	3.06	3.06

**Interval statistics**	**NN**	**RR**
Number	405	405
Minimum (ms)	-30	-30
Maximum (ms)	93	41
Range (ms)	123	71
Avg (ms)	0	0
SD (ms)	11	11
AvgDev (ms)	9	8
p5 (ms)	-16	-16
p50 (ms)	0	0
p95 (ms)	17	17
Skewness	1.47	0.36
Kurtosis	12.93	3.57

Cardio Control NV. User: Anton

Heart Rate Variability: Frequency Domain Analysis

**Name:**	005, 005 005	**Birthdate:**	16/06/1977
**Number:**	005	**Recorded:**	03/05/2018 16:32:33
**Gender:**	Male		

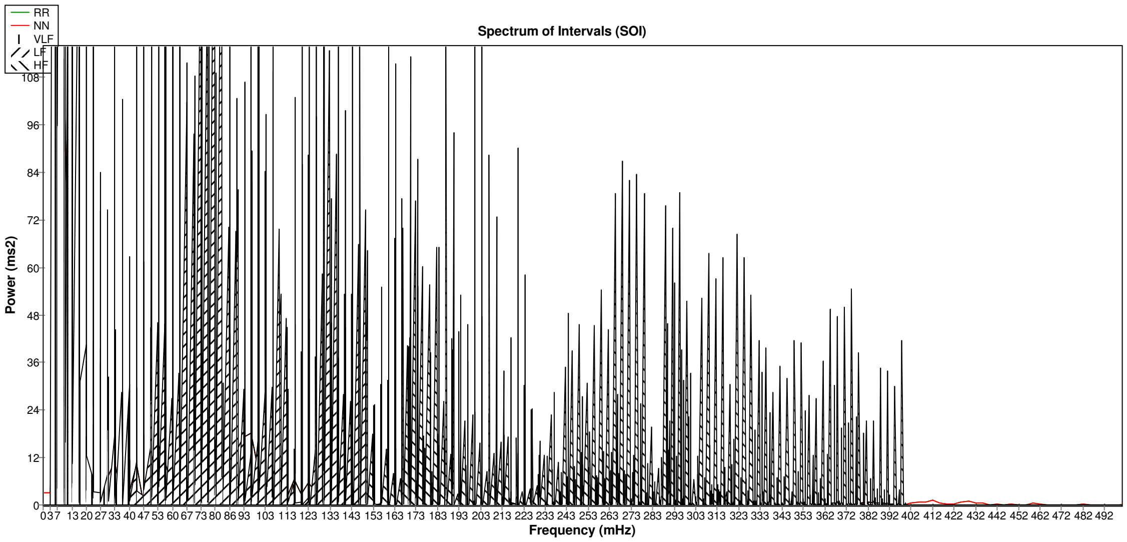

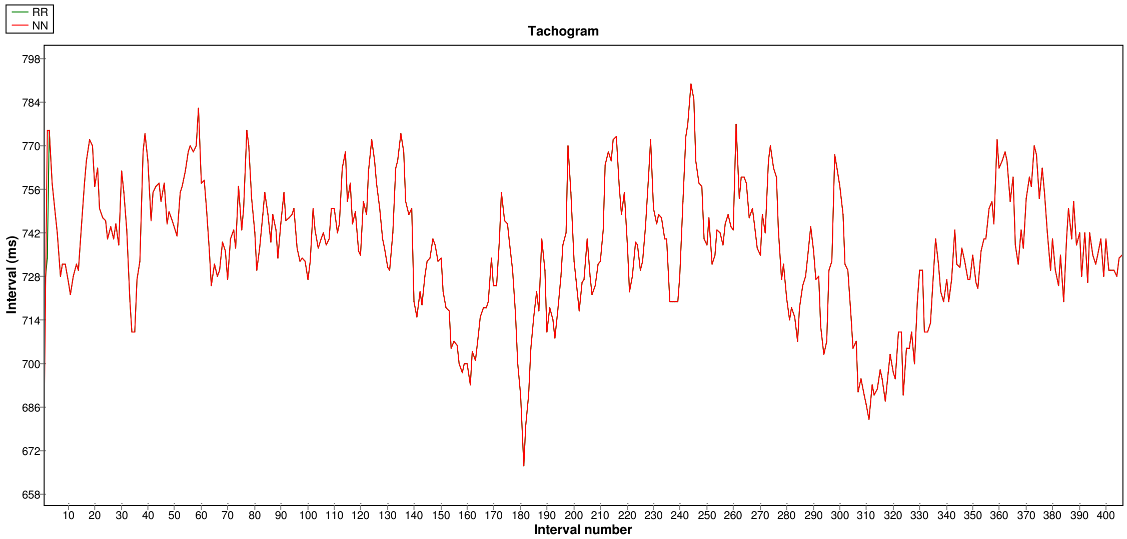

HRV parameters	NN	RR
TP (ms2)	467	467
VLF (ms2)	222	222
LF (ms2)	220	220
HF (ms2)	25	25
LF/HF	8.84	8.84
LF normalized	89.83	89.83
HF normalized	10.17	10.17
VLF peak (mHz)	10	10
LF peak (mHz)	96	96
HF peak (mHz)	176	176

HRV spectral settings	
Spectrum of Intervals (SOI)	
Frequency resolution (mHz)	3
VLF lower boundary (mHz)	3
VLF upper boundary (mHz)	40
LF upper boundary (mHz)	150
HF upper boundary (mHz)	400
Smoothing factor	1
Tapering	Hann
Fourier transform	DFT
Sample frequency (Hz)	1.36
Interval correction	Annotation
Interval threshold (%)	10
