# Supplementary material for: The effects of a 3-day mountain bike cycling race on the autonomic nervous system (ANS) and heart rate variability in amateur cyclists: a prospective quantitative research design
Source: BMC Sports Sci Med Rehabil. 2023 Jan 2;15:2. doi: 10.1186/s13102-022-00614-y (PMC9808932; doi:10.1186/s13102-022-00614-y)
Supplement: Supplementary file 1 — Additional file 1. Individual data of Participants. [file 13102_2022_614_MOESM1_ESM.zip › Individual data of Participants/HRV Data/005/ECG_005_20180504131148_.PDF]

Anton Swart Biokinetic Rehabilitation Practice

Name:	005 005 005	Recorded:	04/05/2018 13:11:48	UNCONFIRMED INTERPRETATION - MD SHOULD REVIEW
Number:	005	Recorded by:	Mr. Anton Swart	
Gender:	Male	Referring physician:		
Birthdate:	16/06/1977 40 years	Ordering physician:		
		Attending physician:		
P / PQ:	113 ms / 168 ms	Location:	Anton Swart Biokinetic Rehabilitation Practi	
QRS:	89 ms	Comment:		
QT / QTc / QTd:	414 ms / 446 ms / -			
P/QRS/T axis:	74° / -82° / 64°			
Heartrate:	78 bpm			

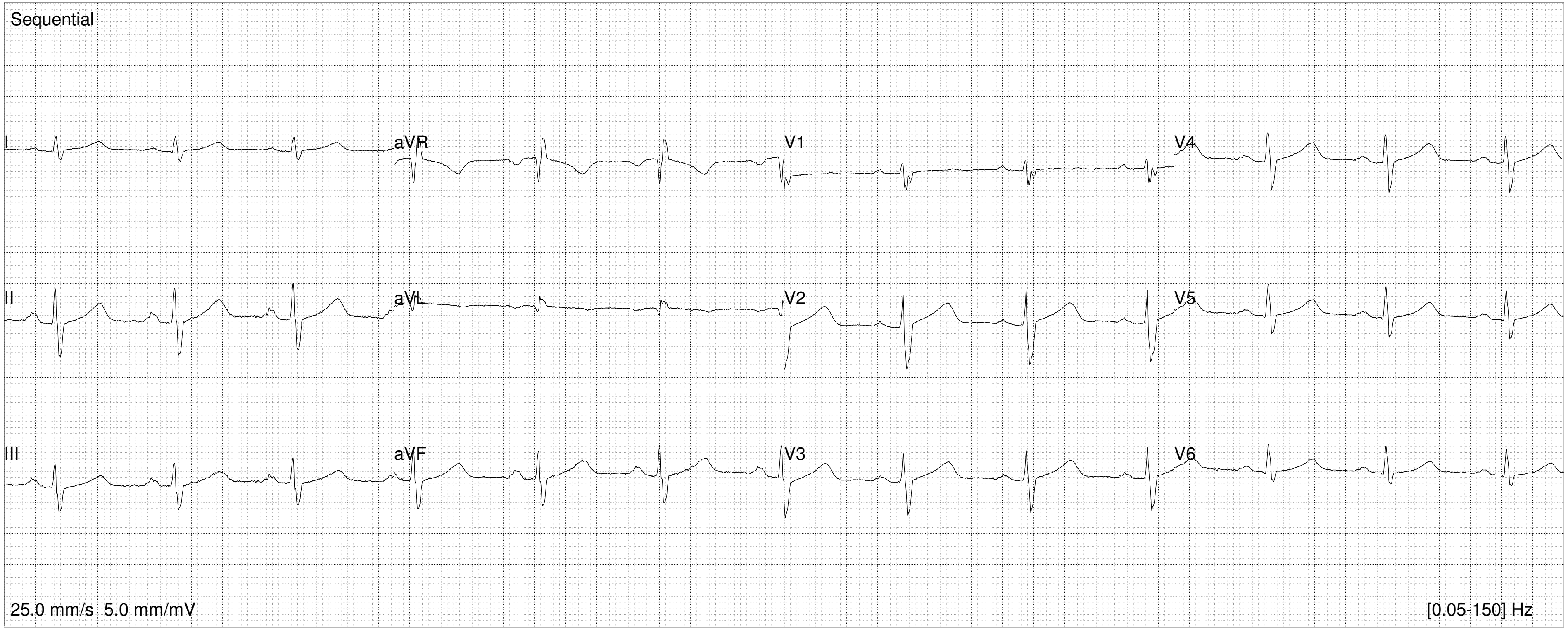

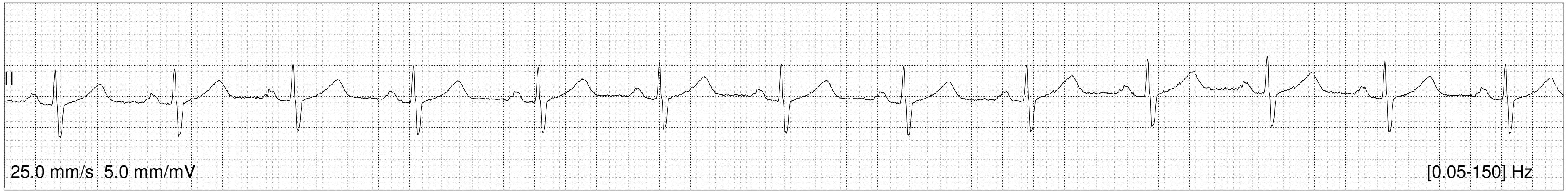

Anton Swart Biokinetic Rehabilitation Practice

Name:	005 005 005	Recorded:	04/05/2018 13:11:48	UNCONFIRMED INTERPRETATION - MD SHOULD REVIEW
Number:	005	Recorded by:	Mr. Anton Swart	
Gender:	Male	Referring physician:		
Birthdate:	16/06/1977 40 years	Location:	Anton Swart Biokinetic Rehabilitation Practice	
P / PQ:	113 ms / 168 ms	Ordering physician:		
QRS:	89 ms	Attending physician:		
QT / QTc / QTd:	414 ms / 446 ms / -	Comment:		
P/QRS/T axis:	74° / -82° / 64°			
Heartrate:	78 bpm			

**Beats**		**RR**	
Total:	389	Minimum	702 ms
Normal:	389	Maximum	905 ms
Other:	0	Mean:	769 ms
		SD:	30 ms

R-R Trend

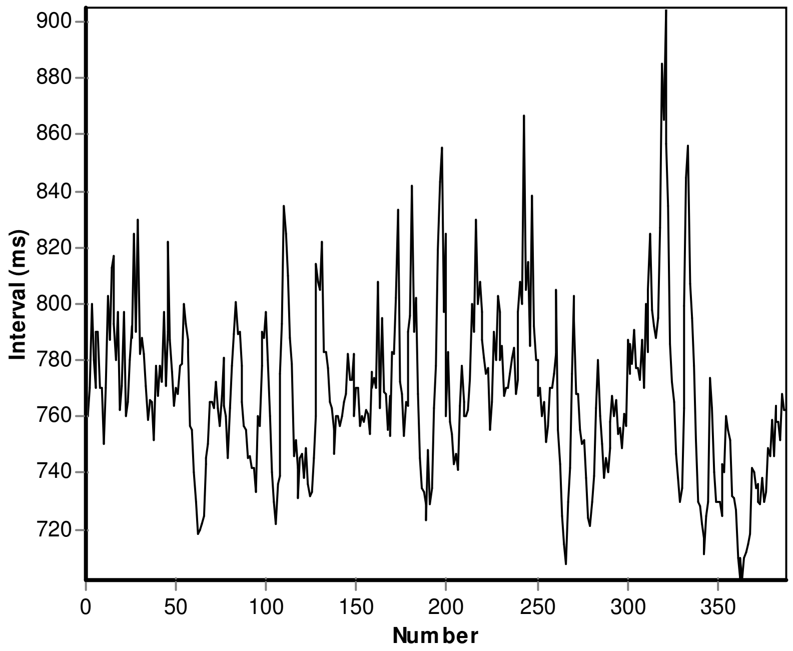

R-R Histogram

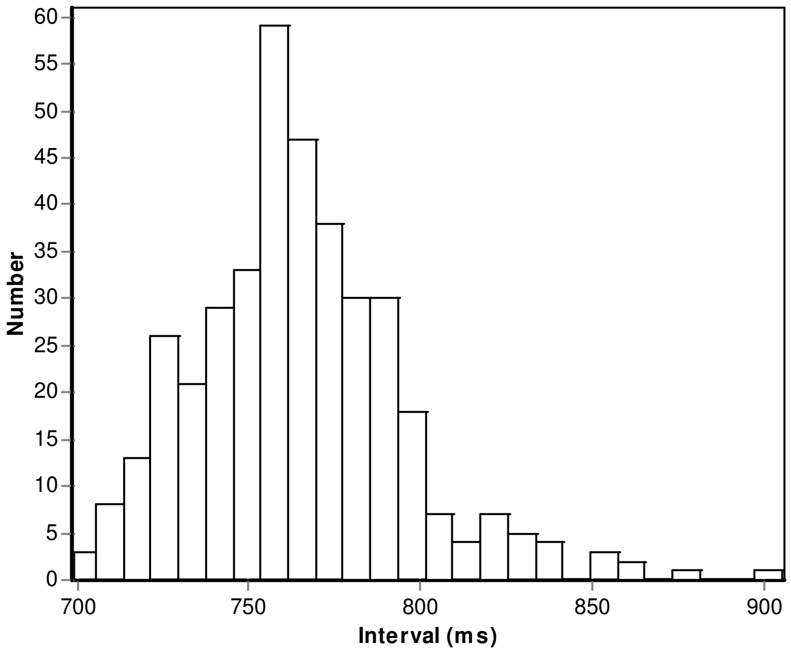

Cardio Control NV. User: Anton

Heart Rate Variability: Time Domain Analysis

**Name:**	005, 005 005	**Birthdate:**	16/06/1977
**Number:**	005	**Recorded:**	04/05/2018 13:11:48
**Gender:**	Male		

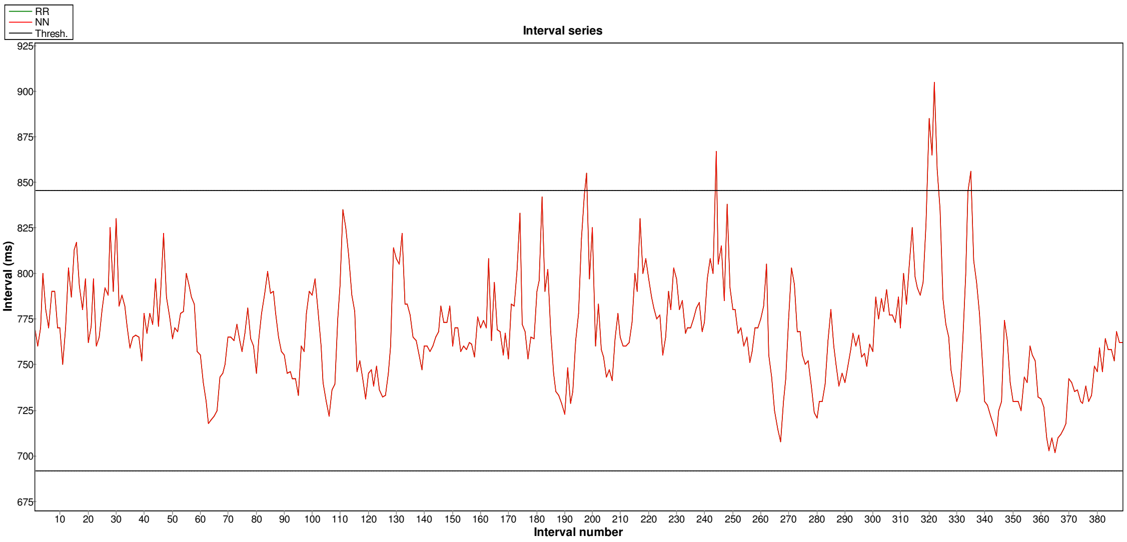

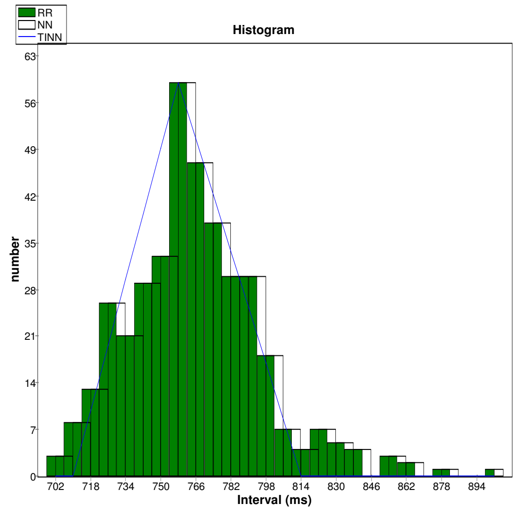

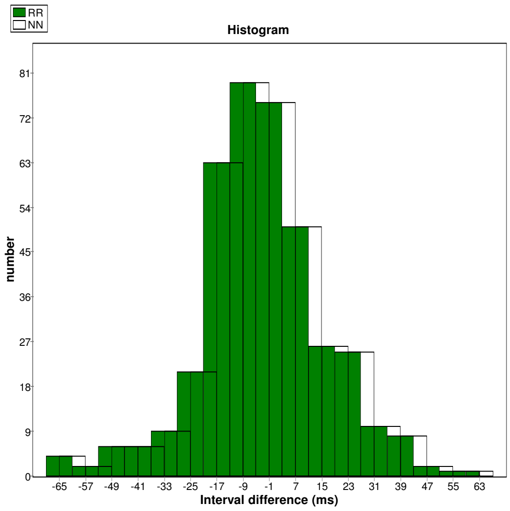

Binsize (ms) = 8

**HRV parameters**	**NN**	**RR**
SDNN (ms)	30	30
Triangular Interpolation (ms)	104	104
Triangular Index	6.59	6.59

**HRV parameters**	**NN**	**RR**
SDSD (ms)	19	19
RMSSD (ms)	19	19
NN50	9	9
NN50(1)	5	5
NN50(2)	4	4
pNN50	0.02	0.02
pNN50(1)	0.01	0.01
pNN50(2)	0.01	0.01
Logarithmic Index	0.54	0.54
SD(Logarithmic Index)	0.02	0.02

**Interval statistics**	**NN**	**RR**
Number	389	389
Minimum (ms)	702	702
Maximum (ms)	905	905
Range (ms)	203	203
Avg (ms)	769	769
SD (ms)	30	30
AvgDev (ms)	23	23
p5 (ms)	724	724
p50 (ms)	767	767
p95 (ms)	825	825
Skewness	0.76	0.76
Kurtosis	4.58	4.58

**Interval statistics**	**NN**	**RR**
Number	388	388
Minimum (ms)	-65	-65
Maximum (ms)	67	67
Range (ms)	132	132
Avg (ms)	-0	-0
SD (ms)	19	19
AvgDev (ms)	14	14
p5 (ms)	-32	-32
p50 (ms)	-1	-1
p95 (ms)	32	32
Skewness	-0.06	-0.06
Kurtosis	4.31	4.31

Cardio Control NV. User: Anton

Heart Rate Variability: Frequency Domain Analysis

**Name:**	005, 005 005	**Birthdate:**	16/06/1977
**Number:**	005	**Recorded:**	04/05/2018 13:11:48
**Gender:**	Male		

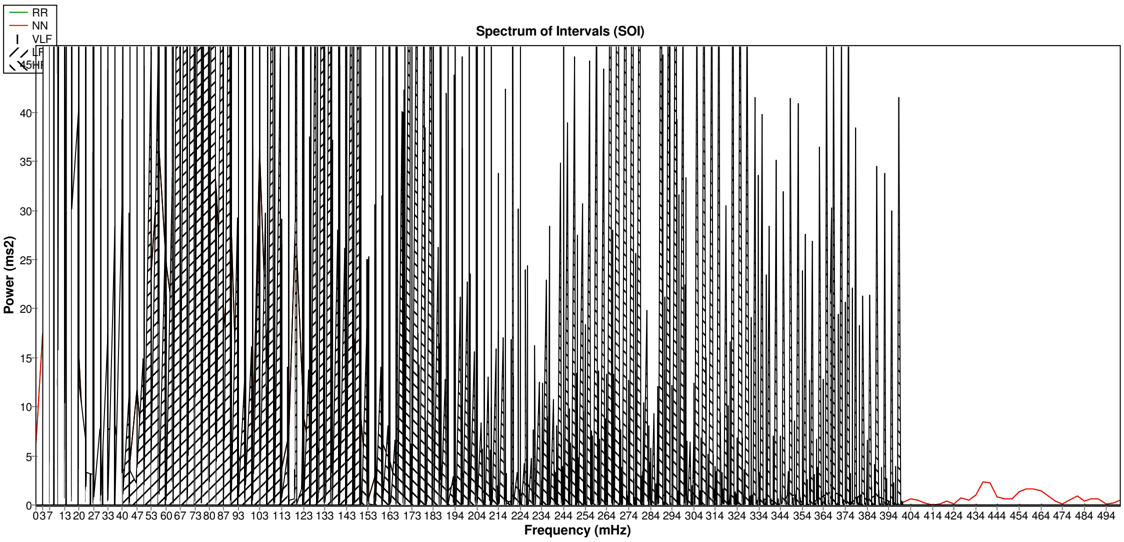

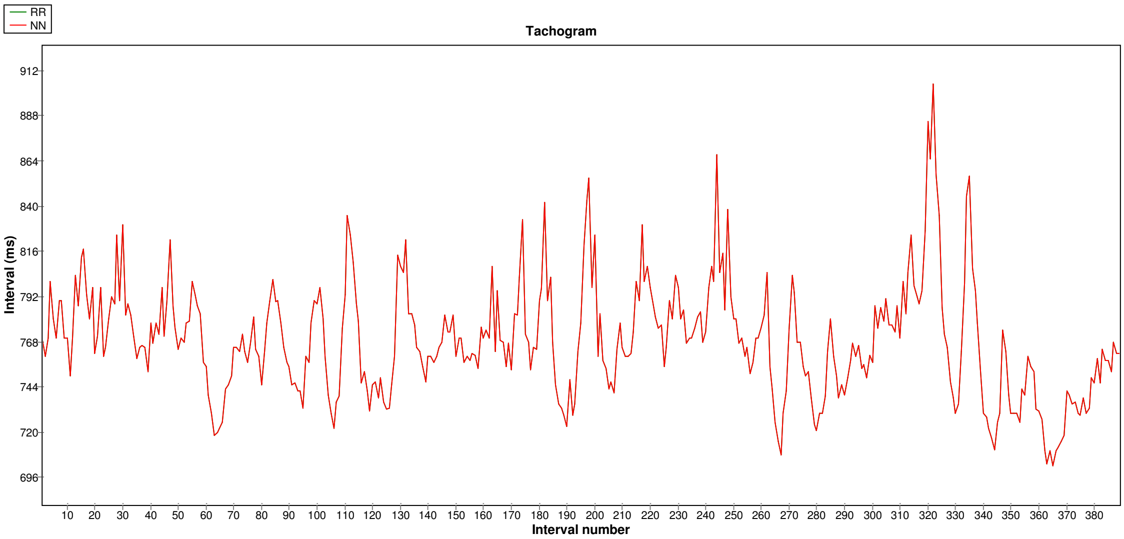

**HRV parameters**	**NN**	**RR**
TP (ms2)	685	685
VLF (ms2)	125	125
LF (ms2)	485	485
HF (ms2)	75	75
LF/HF	6.48	6.48
LF normalized	86.63	86.63
HF normalized	13.37	13.37
VLF peak (mHz)	17	17
LF peak (mHz)	77	77
HF peak (mHz)	150	150

**HRV spectral settings**	
Spectrum of Intervals (SOI)	
Frequency resolution (mHz)	3
VLF lower boundary (mHz)	3
VLF upper boundary (mHz)	40
LF upper boundary (mHz)	150
HF upper boundary (mHz)	400
Smoothing factor	1
Tapering	Hann
Fourier transform	DFT
Sample frequency (Hz)	1.30
Interval correction	Annotation
Interval threshold (%)	10
