# Supplementary material for: The effects of a 3-day mountain bike cycling race on the autonomic nervous system (ANS) and heart rate variability in amateur cyclists: a prospective quantitative research design
Source: BMC Sports Sci Med Rehabil. 2023 Jan 2;15:2. doi: 10.1186/s13102-022-00614-y (PMC9808932; doi:10.1186/s13102-022-00614-y)
Supplement: Supplementary file 1 — Additional file 1. Individual data of Participants. [file 13102_2022_614_MOESM1_ESM.zip › Individual data of Participants/HRV Data/005/ECG_005_20180505132217_.PDF]

Anton Swart Biokinetic Rehabilitation Practice

Name:	005 005 005	Recorded:	05/05/2018 13:22:17	UNCONFIRMED INTERPRETATION - MD SHOULD REVIEW
Number:	005	Recorded by:	Mr. Anton Swart	
Gender:	Male	Referring physician:		
Birthdate:	16/06/1977 40 years	Ordering physician:		
		Attending physician:		
P / PQ:	113 ms / 168 ms	Location:	Anton Swart Biokinetic Rehabilitation Practi	
QRS:	92 ms	Comment:		
QT / QTc / QTd:	419 ms / 447 ms / -			
P/QRS/T axis:	-17° / 131° / -5°			
Heartrate:	76 bpm			

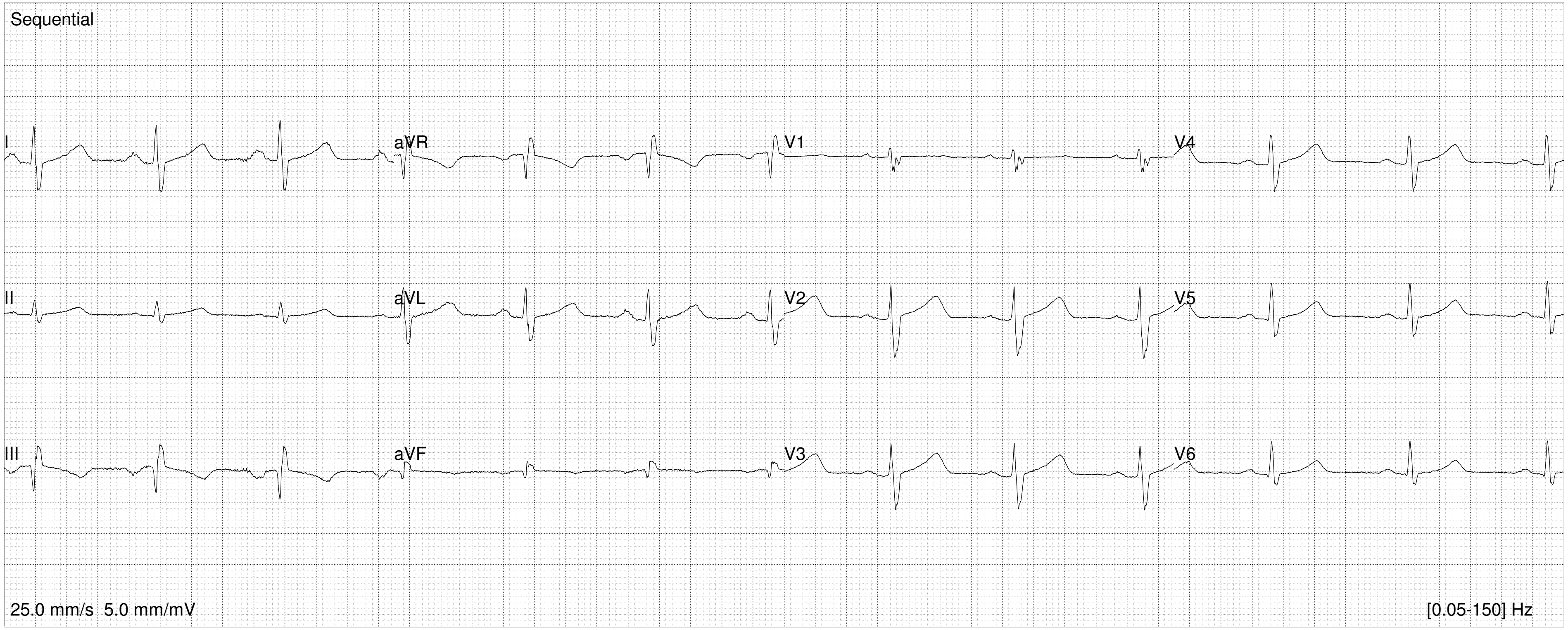

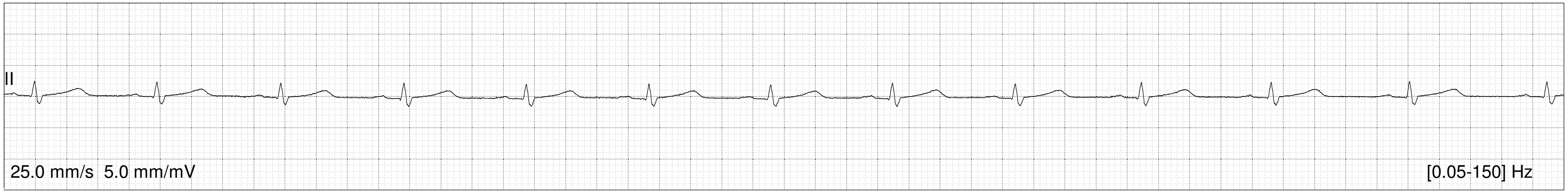

Anton Swart Biokinetic Rehabilitation Practice

Name:	005 005 005	Recorded:	05/05/2018 13:22:17	UNCONFIRMED INTERPRETATION - MD SHOULD REVIEW
Number:	005	Recorded by:	Mr. Anton Swart	
Gender:	Male	Referring physician:		
Birthdate:	16/06/1977 40 years	Location:	Anton Swart Biokinetic Rehabilitation Practice	
P / PQ:	113 ms / 168 ms	Ordering physician:		
QRS:	92 ms	Attending physician:		
QT / QTc / QTd:	419 ms / 447 ms / -	Comment:		
P/QRS/T axis:	-17° / 131° / -5°			
Heartrate:	76 bpm			

**Beats**		**RR**	
Total:	377	Minimum	733 ms
Normal:	377	Maximum	893 ms
Other:	0	Mean:	793 ms
		SD:	25 ms

R-R Trend

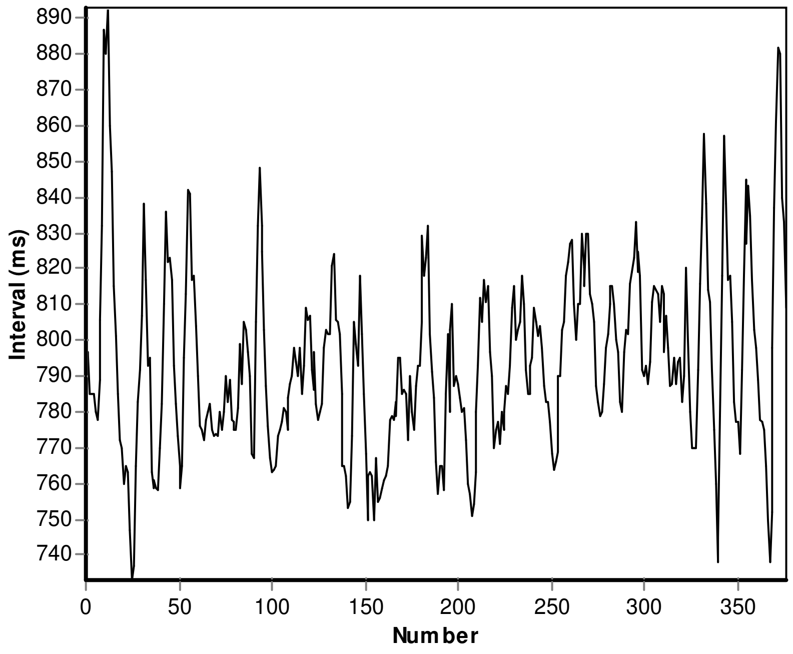

R-R Histogram

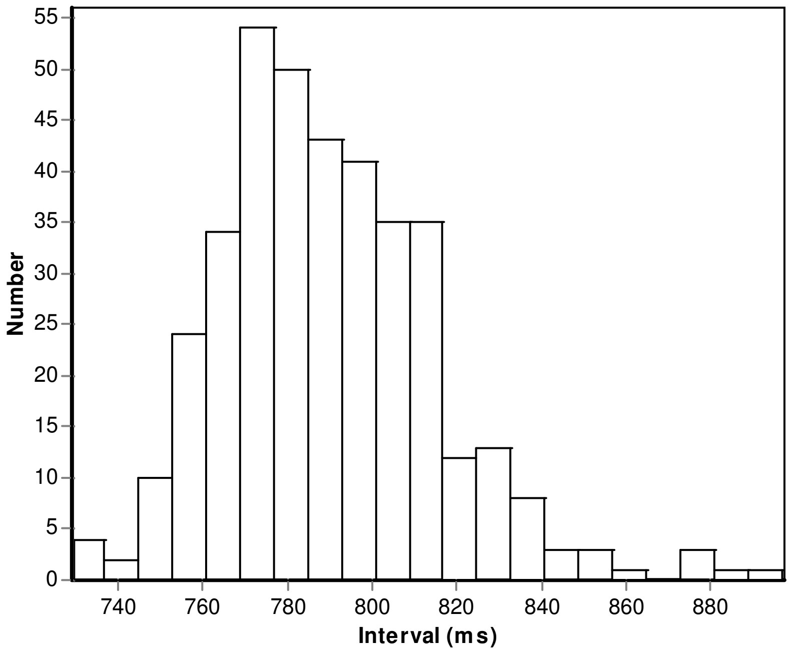

Heart Rate Variability: Time Domain Analysis

**Name:**	005, 005 005	**Birthdate:**	16/06/1977
**Number:**	005	**Recorded:**	05/05/2018 13:22:17
**Gender:**	Male		

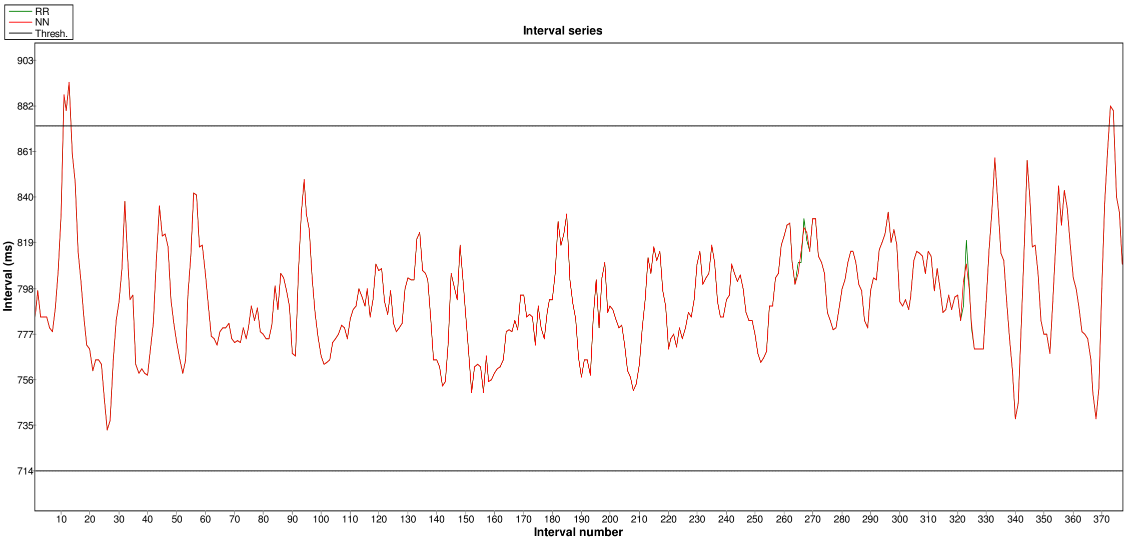

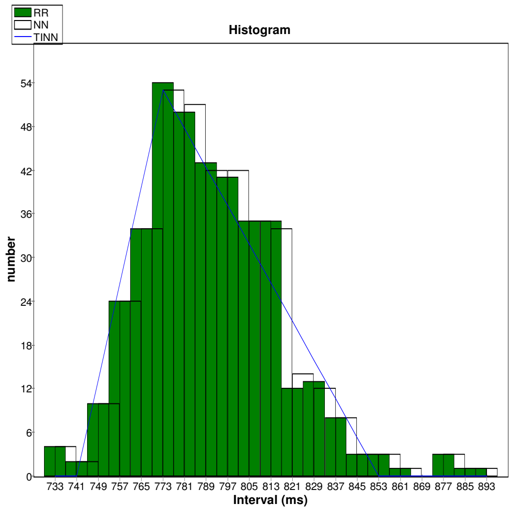

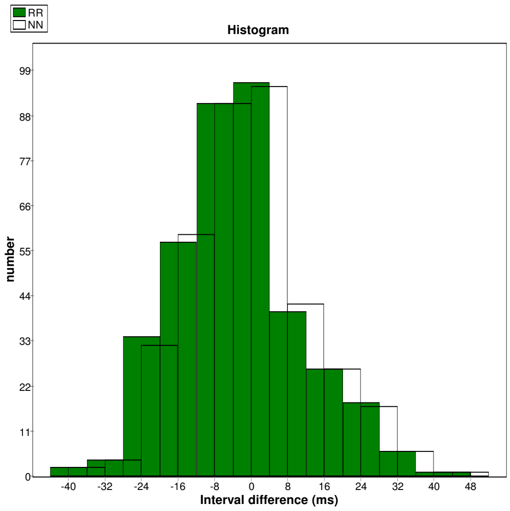

Binsize (ms) = 8

**HRV parameters**	**NN**	**RR**
SDNN (ms)	25	25
Triangular Interpolation (ms)	112	112
Triangular Index	7.11	6.98

**HRV parameters**	**NN**	**RR**
SDSD (ms)	14	14
RMSSD (ms)	14	14
NN50	1	1
NN50(1)	0	0
NN50(2)	1	1
pNN50	0.00	0.00
pNN50(1)	0.00	0.00
pNN50(2)	0.00	0.00
Logarithmic Index	0.90	0.90
SD(Logarithmic Index)	0.06	0.07

**Interval statistics**	**NN**	**RR**
Number	377	377
Minimum (ms)	733	733
Maximum (ms)	893	893
Range (ms)	160	160
Avg (ms)	793	793
SD (ms)	25	25
AvgDev (ms)	20	20
p5 (ms)	758	758
p50 (ms)	790	790
p95 (ms)	838	838
Skewness	0.74	0.74
Kurtosis	4.22	4.21

**Interval statistics**	**NN**	**RR**
Number	376	376
Minimum (ms)	-40	-40
Maximum (ms)	55	55
Range (ms)	95	95
Avg (ms)	0	0
SD (ms)	14	14
AvgDev (ms)	10	10
p5 (ms)	-21	-21
p50 (ms)	-1	-1
p95 (ms)	26	26
Skewness	0.51	0.51
Kurtosis	3.91	3.86

Heart Rate Variability: Frequency Domain Analysis

**Name:**	005, 005 005	**Birthdate:**	16/06/1977
**Number:**	005	**Recorded:**	05/05/2018 13:22:17
**Gender:**	Male		

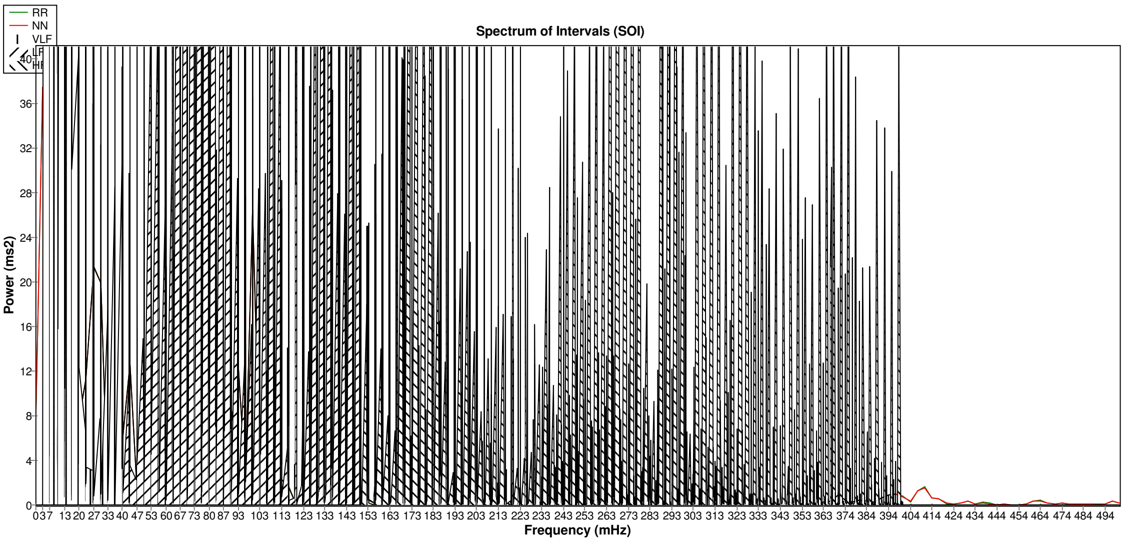

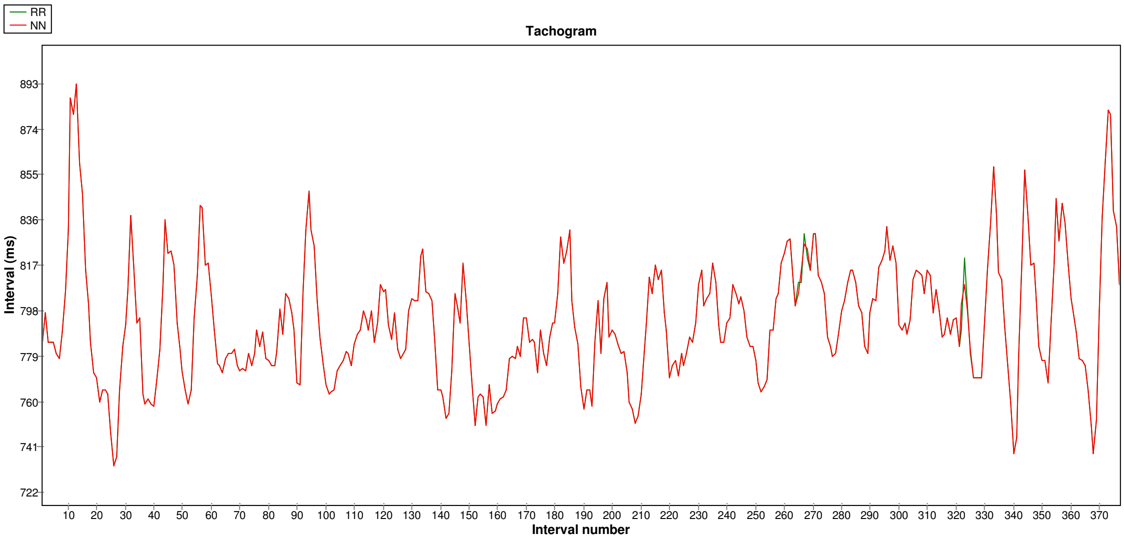

**HRV parameters**	**NN**	**RR**
TP (ms2)	360	360
VLF (ms2)	101	101
LF (ms2)	235	235
HF (ms2)	24	24
LF/HF	9.66	9.66
LF normalized	90.62	90.62
HF normalized	9.38	9.38
VLF peak (mHz)	7	7
LF peak (mHz)	77	77
HF peak (mHz)	310	310

**HRV spectral settings**	
Spectrum of Intervals (SOI)	
Frequency resolution (mHz)	3
VLF lower boundary (mHz)	3
VLF upper boundary (mHz)	40
LF upper boundary (mHz)	150
HF upper boundary (mHz)	400
Smoothing factor	1
Tapering	Hann
Fourier transform	DFT
Sample frequency (Hz)	1.26
Interval correction	Annotation
Interval threshold (%)	10
