# Supplementary material for: The effects of a 3-day mountain bike cycling race on the autonomic nervous system (ANS) and heart rate variability in amateur cyclists: a prospective quantitative research design
Source: BMC Sports Sci Med Rehabil. 2023 Jan 2;15:2. doi: 10.1186/s13102-022-00614-y (PMC9808932; doi:10.1186/s13102-022-00614-y)
Supplement: Supplementary file 1 — Additional file 1. Individual data of Participants. [file 13102_2022_614_MOESM1_ESM.zip › Individual data of Participants/HRV Data/005/ECG_005_20180506075040_.PDF]

Anton Swart Biokinetic Rehabilitation Practice

Name: 005 005 005
Number: 005
Gender: Male
Birthdate: 16/06/1977 40 years

P / PQ: 117 ms / 163 ms
QRS: 92 ms
QT / QTc / QTd: 492 ms / 483 ms / -
P/QRS/T axis: 76° / 6° / 57°
Heartrate: 55 bpm

Recorded: 06/05/2018 07:50:40
Recorded by: Mr. Anton Swart
Referring physician:
Ordering physician:
Attending physician:
Location: Anton Swart Biokinetic Rehabilitation Practi
Comment:

UNCONFIRMED INTERPRETATION - MD SHOULD REVIEW

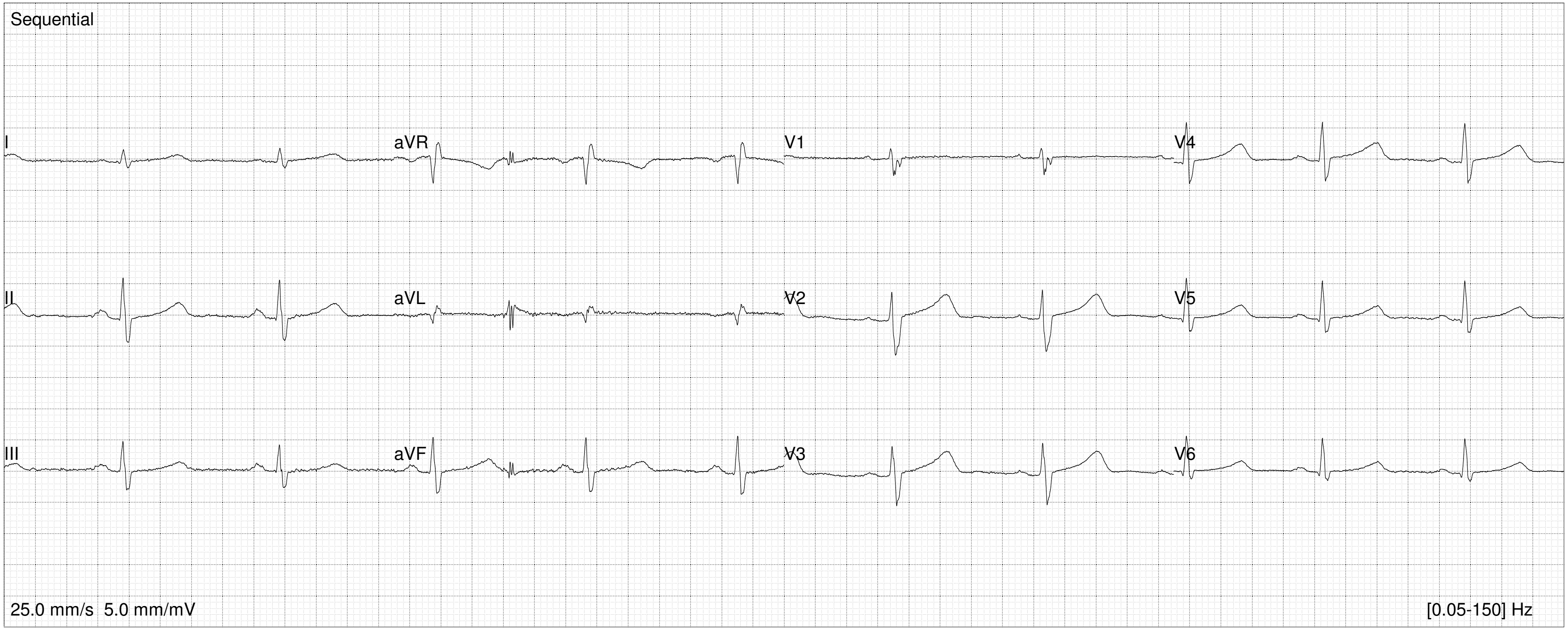

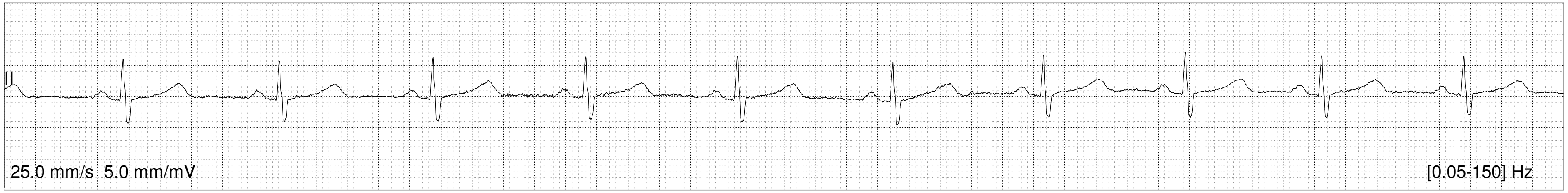

Anton Swart Biokinetic Rehabilitation Practice

Name:	005 005 005	Recorded:	06/05/2018 07:50:40	UNCONFIRMED INTERPRETATION - MD SHOULD REVIEW
Number:	005	Recorded by:	Mr. Anton Swart	
Gender:	Male	Referring physician:		
Birthdate:	16/06/1977 40 years	Location:	Anton Swart Biokinetic Rehabilitation Practice	
		Ordering physician:		
		Attending physician:		
		Comment:		

P / PQ:	117 ms / 163 ms
QRS:	92 ms
QT / QTc / QTd:	492 ms / 483 ms / -
P/QRS/T axis:	76° / 6° / 57°
Heartrate:	55 bpm

Beats

Total:	274
Normal:	274
Other:	0

RR

Minimum	872 ms
Maximum	1365 ms
Mean:	1091 ms
SD:	62 ms

R-R Trend

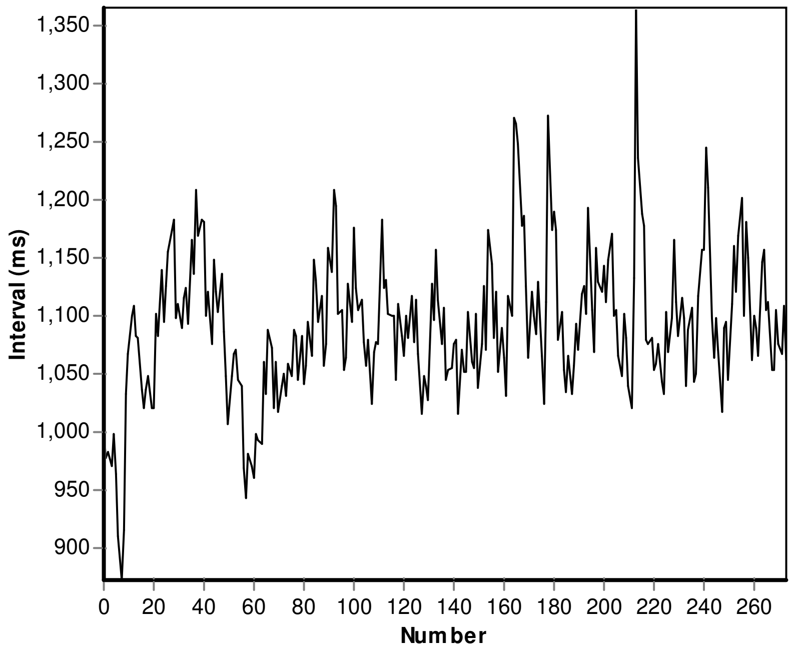

R-R Histogram

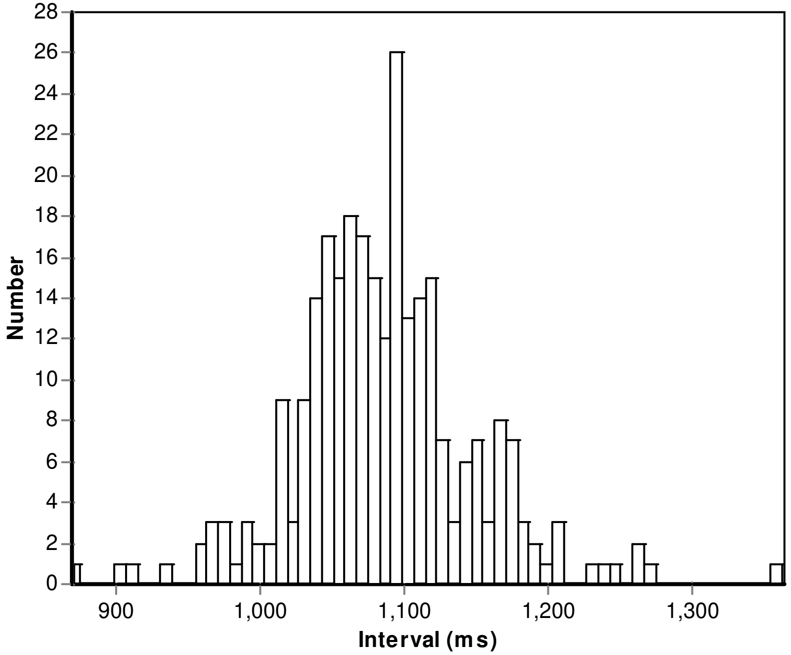

Cardio Control NV. User: Anton

Heart Rate Variability: Time Domain Analysis

**Name:**	005, 005 005	**Birthdate:**	16/06/1977
**Number:**	005	**Recorded:**	06/05/2018 07:50:40
**Gender:**	Male		

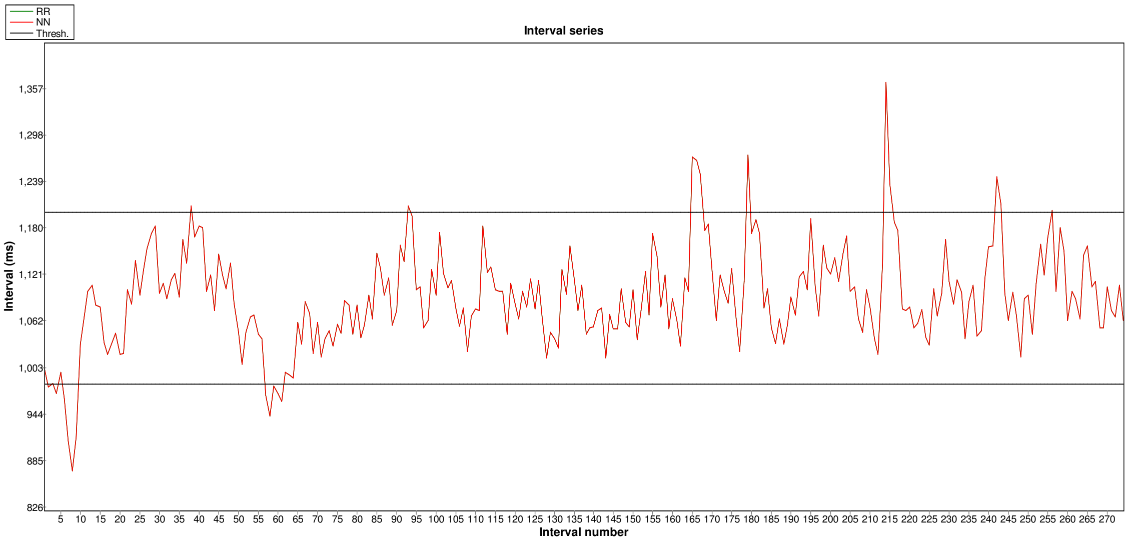

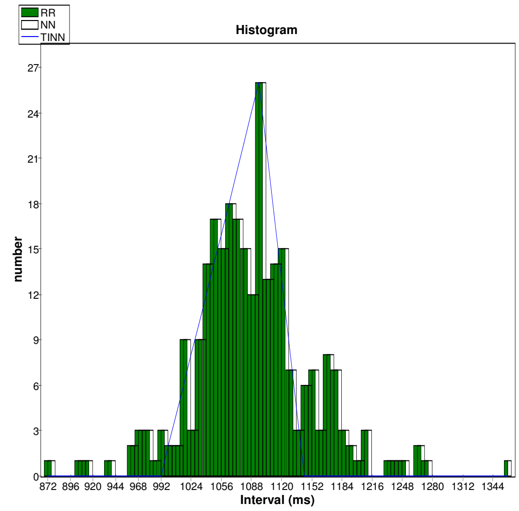

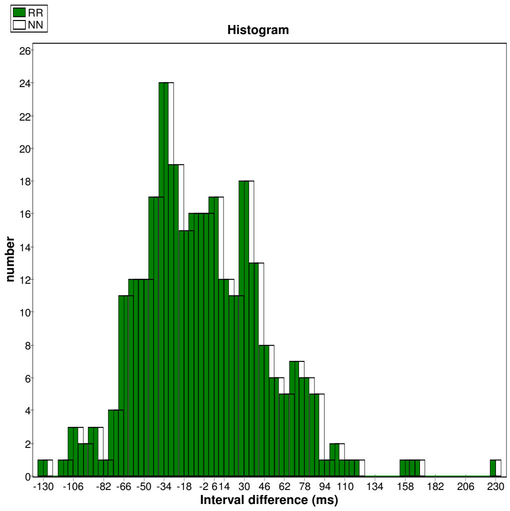

Binsize (ms) = 8

**HRV parameters**	**NN**	**RR**
SDNN (ms)	62	62
Triangular Interpolation (ms)	152	152
Triangular Index	10.54	10.54

**HRV parameters**	**NN**	**RR**
SDSD (ms)	50	50
RMSSD (ms)	50	50
NN50	77	77
NN50(1)	38	38
NN50(2)	39	39
pNN50	0.28	0.28
pNN50(1)	0.14	0.14
pNN50(2)	0.14	0.14
Logarithmic Index	0.24	0.24
SD(Logarithmic Index)	0.02	0.02

**Interval statistics**	**NN**	**RR**
Number	274	274
Minimum (ms)	872	872
Maximum (ms)	1365	1365
Range (ms)	493	493
Avg (ms)	1091	1091
SD (ms)	62	62
AvgDev (ms)	46	46
p5 (ms)	992	992
p50 (ms)	1088	1088
p95 (ms)	1191	1191
Skewness	0.34	0.34
Kurtosis	5.02	5.02

**Interval statistics**	**NN**	**RR**
Number	273	273
Minimum (ms)	-130	-130
Maximum (ms)	232	232
Range (ms)	362	362
Avg (ms)	0	0
SD (ms)	50	50
AvgDev (ms)	39	39
p5 (ms)	-70	-70
p50 (ms)	-5	-5
p95 (ms)	87	87
Skewness	0.65	0.65
Kurtosis	4.47	4.47

Heart Rate Variability: Frequency Domain Analysis

**Name:**	005, 005 005	**Birthdate:**	16/06/1977
**Number:**	005	**Recorded:**	06/05/2018 07:50:40
**Gender:**	Male		

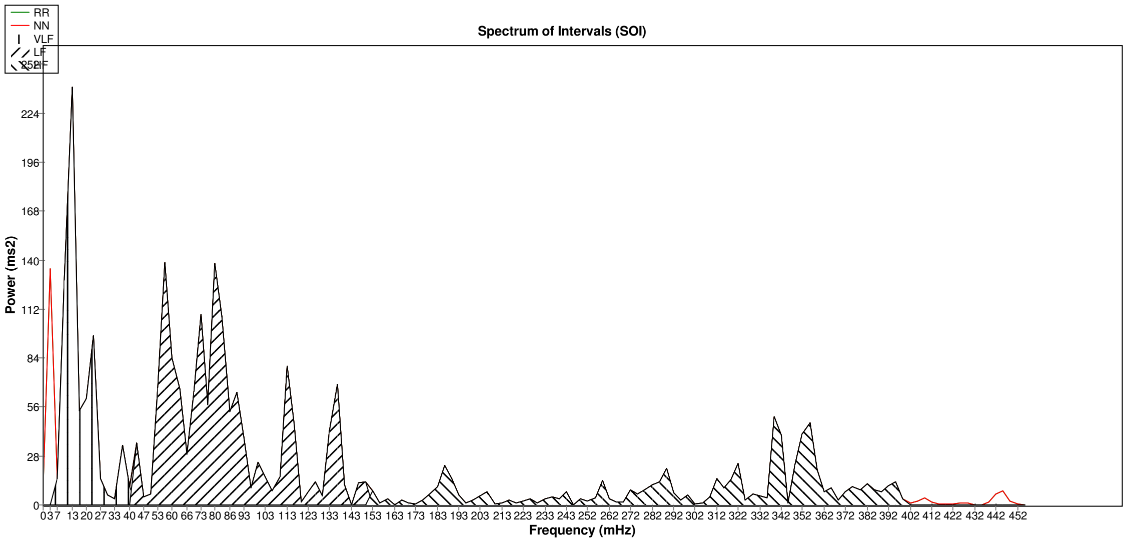

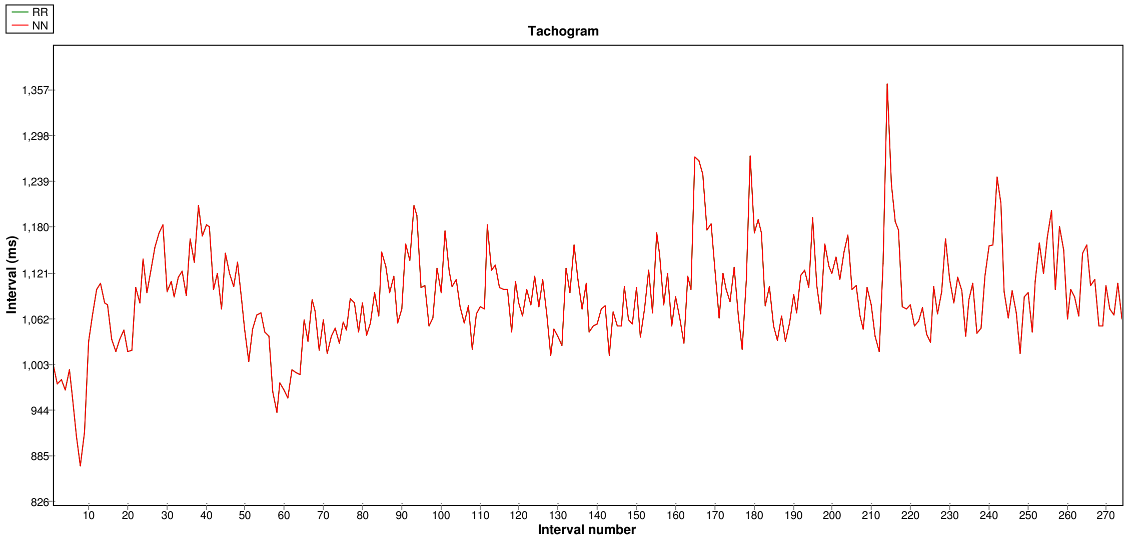

**HRV parameters**	**NN**	**RR**
TP (ms2)	2764	2764
VLF (ms2)	664	664
LF (ms2)	1441	1441
HF (ms2)	659	659
LF/HF	2.19	2.19
LF normalized	68.62	68.62
HF normalized	31.38	31.38
VLF peak (mHz)	13	13
LF peak (mHz)	56	56
HF peak (mHz)	339	339

**HRV spectral settings**	
Spectrum of Intervals (SOI)	
Frequency resolution (mHz)	3
VLF lower boundary (mHz)	3
VLF upper boundary (mHz)	40
LF upper boundary (mHz)	150
HF upper boundary (mHz)	400
Smoothing factor	1
Tapering	Hann
Fourier transform	DFT
Sample frequency (Hz)	0.92
Interval correction	Annotation
Interval threshold (%)	10
