# Supplementary material for: The effects of a 3-day mountain bike cycling race on the autonomic nervous system (ANS) and heart rate variability in amateur cyclists: a prospective quantitative research design
Source: BMC Sports Sci Med Rehabil. 2023 Jan 2;15:2. doi: 10.1186/s13102-022-00614-y (PMC9808932; doi:10.1186/s13102-022-00614-y)
Supplement: Supplementary file 1 — Additional file 1. Individual data of Participants. [file 13102_2022_614_MOESM1_ESM.zip › Individual data of Participants/HRV Data/006/ECG_006_20180501083440_.PDF]

Anton Swart Biokinetic Rehabilitation Practice

Name:	006 006 006	Recorded:	01/05/2018 08:34:40	UNCONFIRMED INTERPRETATION - MD SHOULD REVIEW
Number:	006	Recorded by:	Mr. Anton Swart	
Gender:	Female	Referring physician:		
Birthdate:	25/01/1979 39 years	Ordering physician:		
		Attending physician:		
		Location:	Anton Swart Biokinetic Rehabilitation Practi	
		Comment:		

P / PQ:	103 ms / 182 ms
QRS:	90 ms
QT / QTc / QTd:	441 ms / 458 ms / -
P/QRS/T axis:	72° / 86° / 71°
Heartrate:	70 bpm

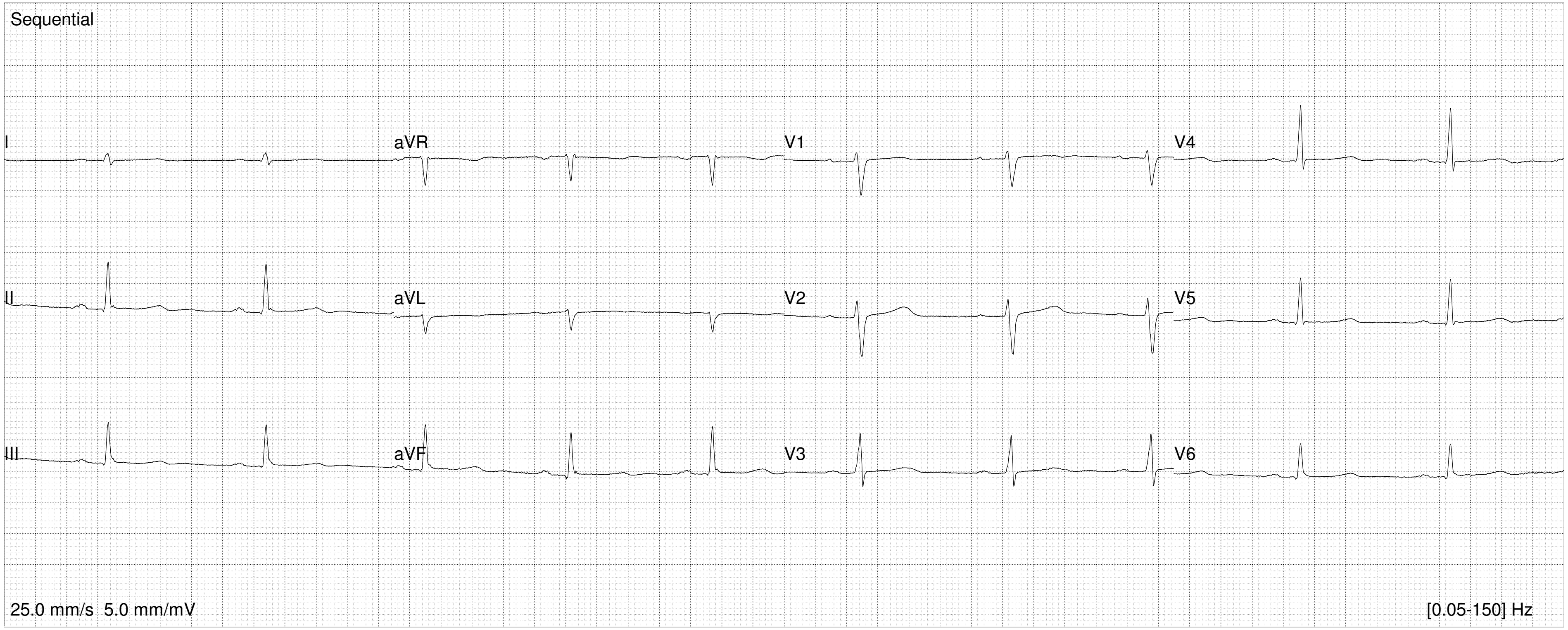

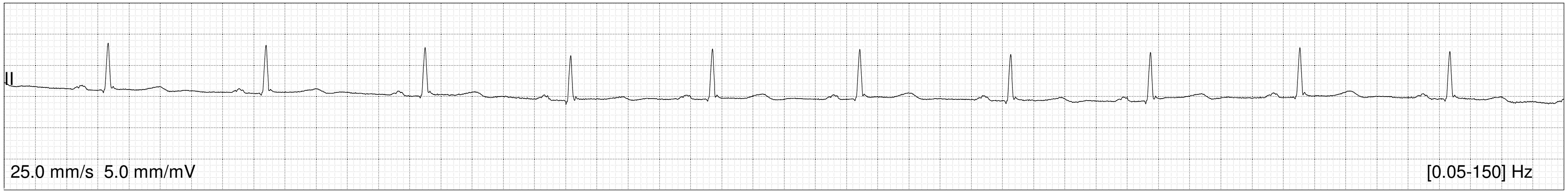

Anton Swart Biokinetic Rehabilitation Practice

Name:	006 006 006	Recorded:	01/05/2018 08:34:40	UNCONFIRMED INTERPRETATION - MD SHOULD REVIEW
Number:	006	Recorded by:	Mr. Anton Swart	
Gender:	Female	Referring physician:		
Birthdate:	25/01/1979 39 years	Location:	Anton Swart Biokinetic Rehabilitation Practice	
P / PQ:	103 ms / 182 ms	Ordering physician:		
QRS:	90 ms	Attending physician:		
QT / QTc / QTd:	441 ms / 458 ms / -	Comment:		
P/QRS/T axis:	72° / 86° / 71°			
Heartrate:	70 bpm			

**Beats**		**RR**	
Total:	344	Minimum	763 ms
Normal:	344	Maximum	1018 ms
Other:	0	Mean:	868 ms
		SD:	46 ms

R-R Trend

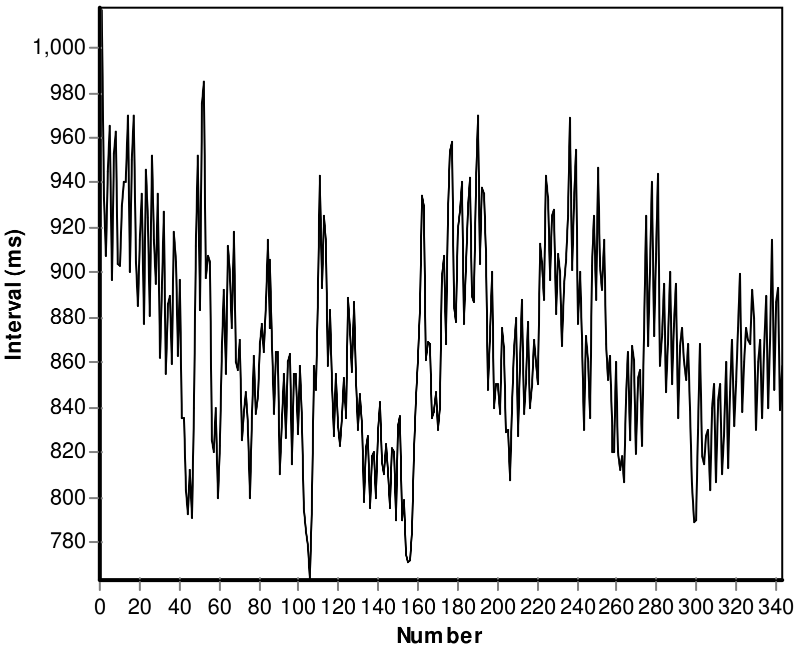

R-R Histogram

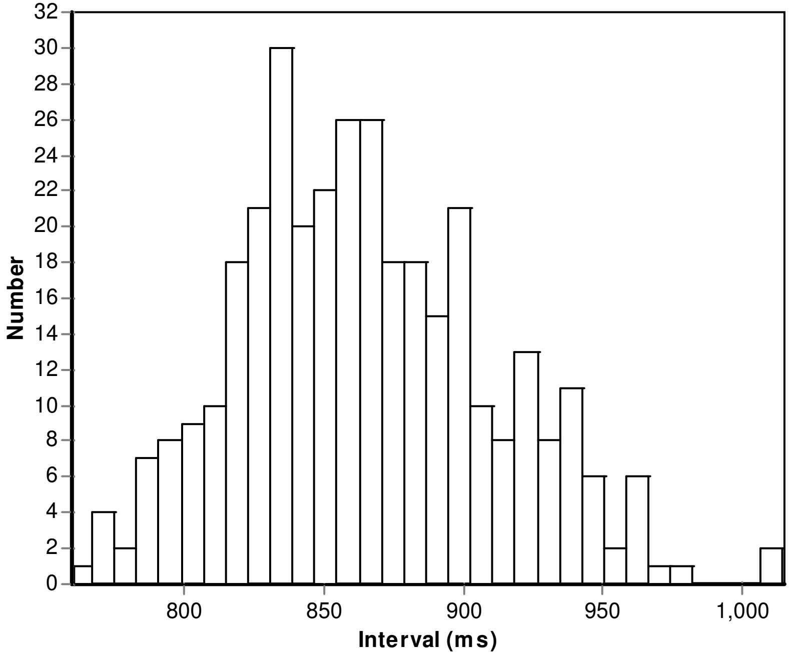

Cardio Control NV. User: Anton

Heart Rate Variability: Time Domain Analysis

**Name:**	006, 006 006	**Birthdate:**	25/01/1979
**Number:**	006	**Recorded:**	01/05/2018 08:34:40
**Gender:**	Female		

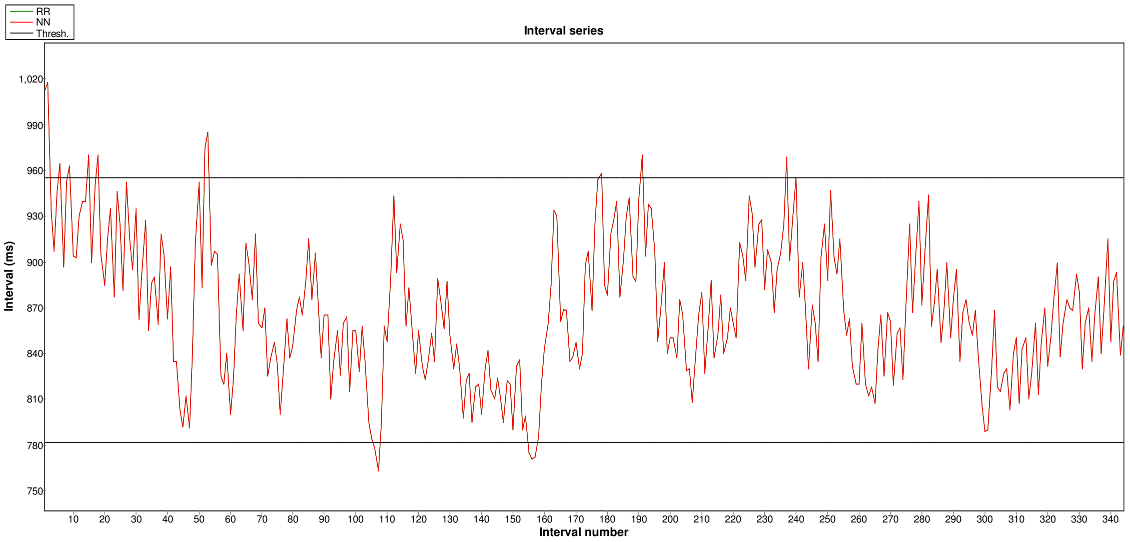

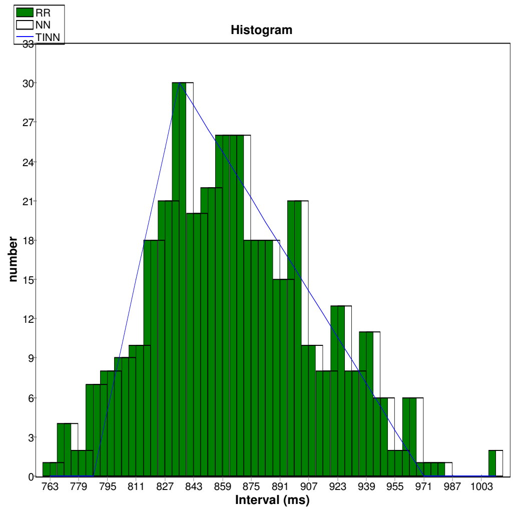

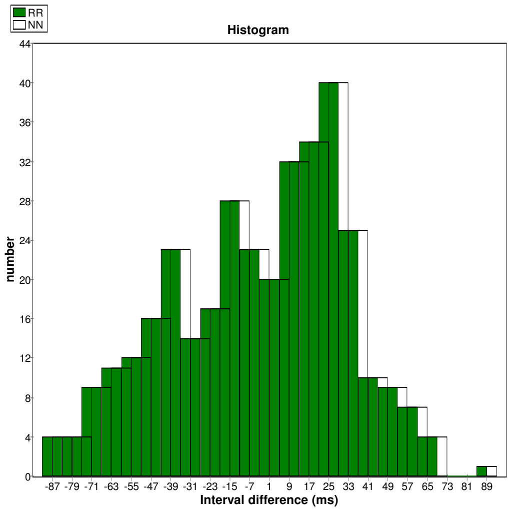

Binsize (ms) = 8

**HRV parameters**	**NN**	**RR**
SDNN (ms)	46	46
Triangular Interpolation (ms)	184	184
Triangular Index	11.47	11.47

**Interval statistics**	**NN**	**RR**
Number	344	344
Minimum (ms)	763	763
Maximum (ms)	1018	1018
Range (ms)	255	255
Avg (ms)	868	868
SD (ms)	46	46
AvgDev (ms)	37	37
p5 (ms)	797	797
p50 (ms)	865	865
p95 (ms)	949	949
Skewness	0.37	0.37
Kurtosis	2.89	2.89

**HRV parameters**	**NN**	**RR**
SDSD (ms)	35	35
RMSSD (ms)	35	35
NN50	51	51
NN50(1)	33	33
NN50(2)	18	18
pNN50	0.15	0.15
pNN50(1)	0.10	0.10
pNN50(2)	0.05	0.05
Logarithmic Index	0.32	0.32
SD(Logarithmic Index)	0.05	0.05

**Interval statistics**	**NN**	**RR**
Number	343	343
Minimum (ms)	-87	-87
Maximum (ms)	92	92
Range (ms)	179	179
Avg (ms)	-0	-0
SD (ms)	35	35
AvgDev (ms)	29	29
p5 (ms)	-63	-63
p50 (ms)	4	4
p95 (ms)	54	54
Skewness	-0.30	-0.30
Kurtosis	2.42	2.42

Cardio Control NV. User: Anton

Heart Rate Variability: Frequency Domain Analysis

**Name:**	006, 006 006	**Birthdate:**	25/01/1979
**Number:**	006	**Recorded:**	01/05/2018 08:34:40
**Gender:**	Female		

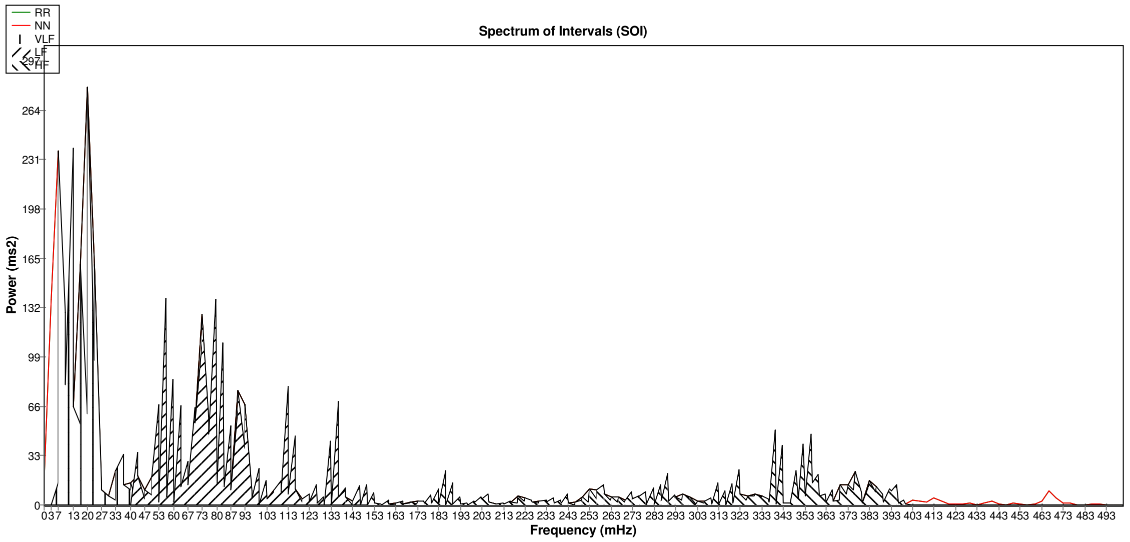

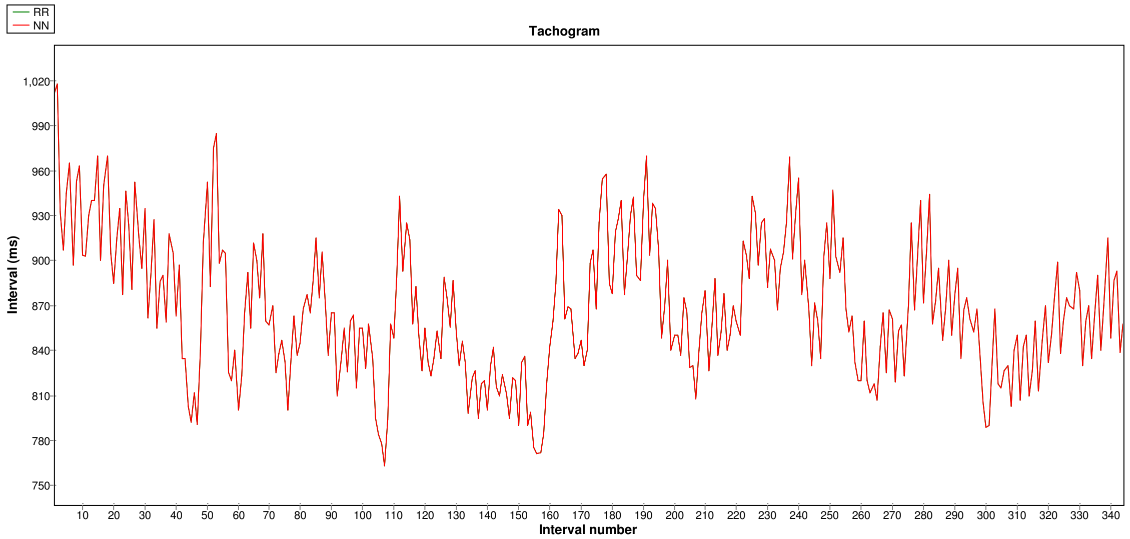

**HRV parameters**	**NN**	**RR**
TP (ms2)	1918	1918
VLF (ms2)	1052	1052
LF (ms2)	564	564
HF (ms2)	302	302
LF/HF	1.87	1.87
LF normalized	65.10	65.10
HF normalized	34.90	34.90
VLF peak (mHz)	20	20
LF peak (mHz)	73	73
HF peak (mHz)	376	376

**HRV spectral settings**	
Spectrum of Intervals (SOI)	
Frequency resolution (mHz)	3
VLF lower boundary (mHz)	3
VLF upper boundary (mHz)	40
LF upper boundary (mHz)	150
HF upper boundary (mHz)	400
Smoothing factor	1
Tapering	Hann
Fourier transform	DFT
Sample frequency (Hz)	1.15
Interval correction	Annotation
Interval threshold (%)	10
