# Supplementary material for: The effects of a 3-day mountain bike cycling race on the autonomic nervous system (ANS) and heart rate variability in amateur cyclists: a prospective quantitative research design
Source: BMC Sports Sci Med Rehabil. 2023 Jan 2;15:2. doi: 10.1186/s13102-022-00614-y (PMC9808932; doi:10.1186/s13102-022-00614-y)
Supplement: Supplementary file 1 — Additional file 1. Individual data of Participants. [file 13102_2022_614_MOESM1_ESM.zip › Individual data of Participants/HRV Data/006/ECG_006_20180503180827_.PDF]

Anton Swart Biokinetic Rehabilitation Practice

Name:	006 006 006	Recorded:	03/05/2018 18:08:27	UNCONFIRMED INTERPRETATION - MD SHOULD REVIEW
Number:	006	Recorded by:	Mr. Anton Swart	
Gender:	Female	Referring physician:		
Birthdate:	25/01/1979 39 years	Ordering physician:		
		Attending physician:		
P / PQ:	108 ms / 175 ms	Location:	Anton Swart Biokinetic Rehabilitation Practi	
QRS:	92 ms	Comment:		
QT / QTc / QTd:	392 ms / 432 ms / -			
P/QRS/T axis:	77° / 85° / 73°			
Heartrate:	83 bpm			

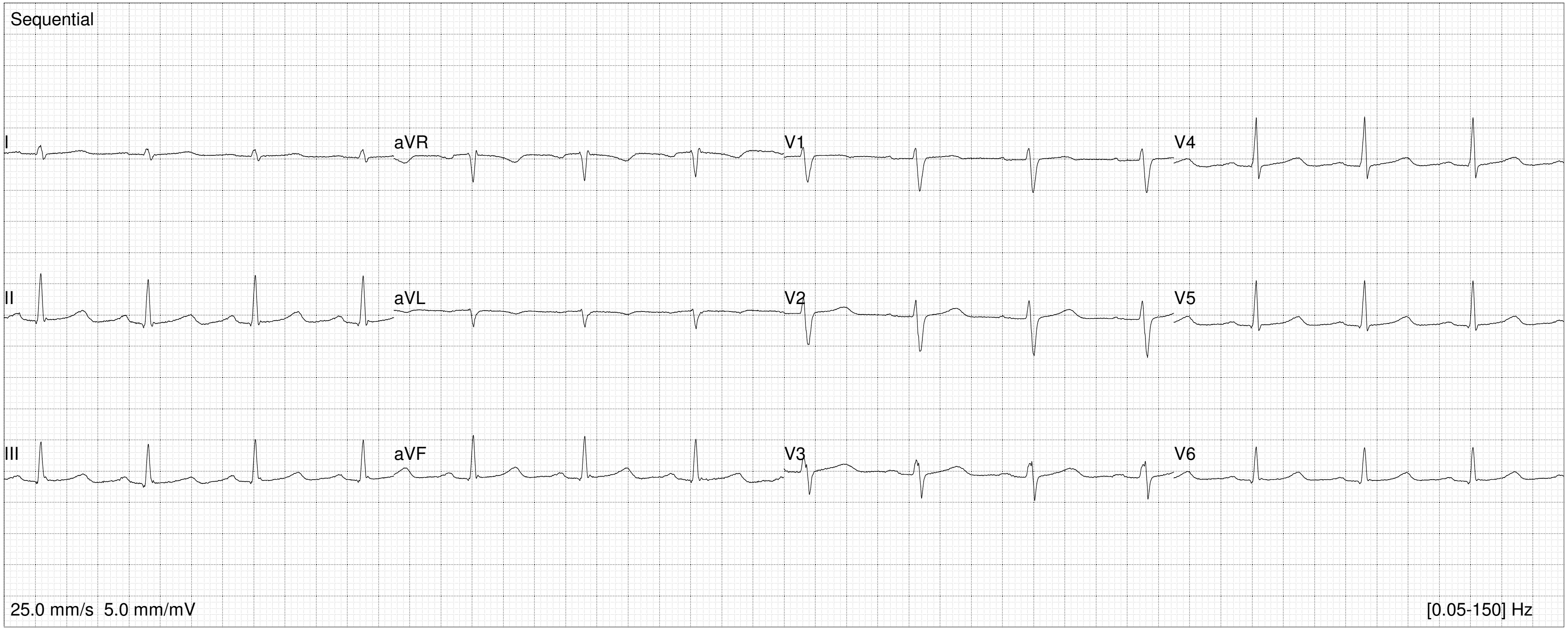

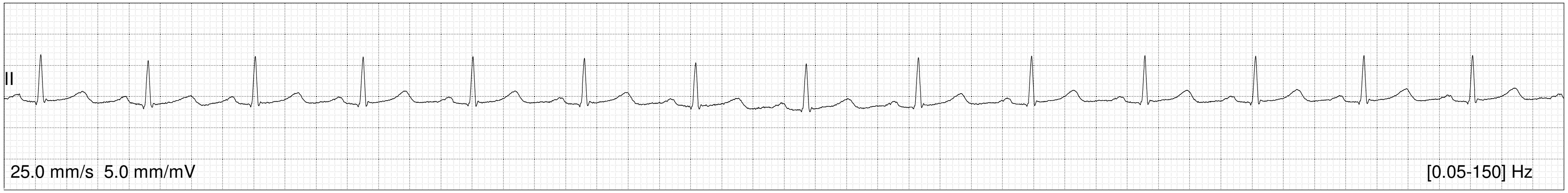

Anton Swart Biokinetic Rehabilitation Practice

Name:	006 006 006	Recorded:	03/05/2018 18:08:27	UNCONFIRMED INTERPRETATION - MD SHOULD REVIEW
Number:	006	Recorded by:	Mr. Anton Swart	
Gender:	Female	Referring physician:		
Birthdate:	25/01/1979 39 years	Location:	Anton Swart Biokinetic Rehabilitation Practice	
		Ordering physician:		
		Attending physician:		
		Comment:		

P / PQ:	108 ms / 175 ms
QRS:	92 ms
QT / QTc / QTd:	392 ms / 432 ms / -
P/QRS/T axis:	77° / 85° / 73°
Heartrate:	83 bpm

Beats

Total:	415
Normal:	415
Other:	0

RR

Minimum	610 ms
Maximum	860 ms
Mean:	722 ms
SD:	29 ms

R-R Trend

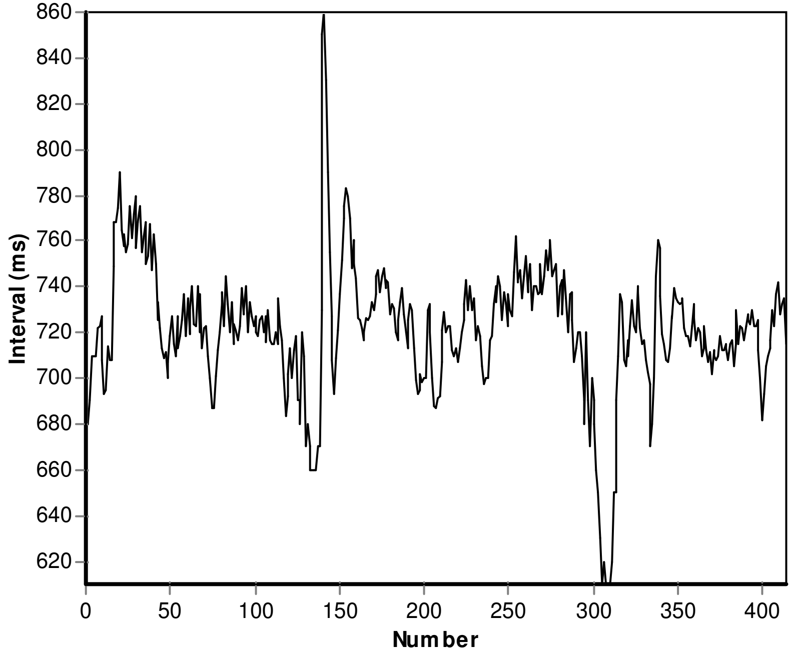

R-R Histogram

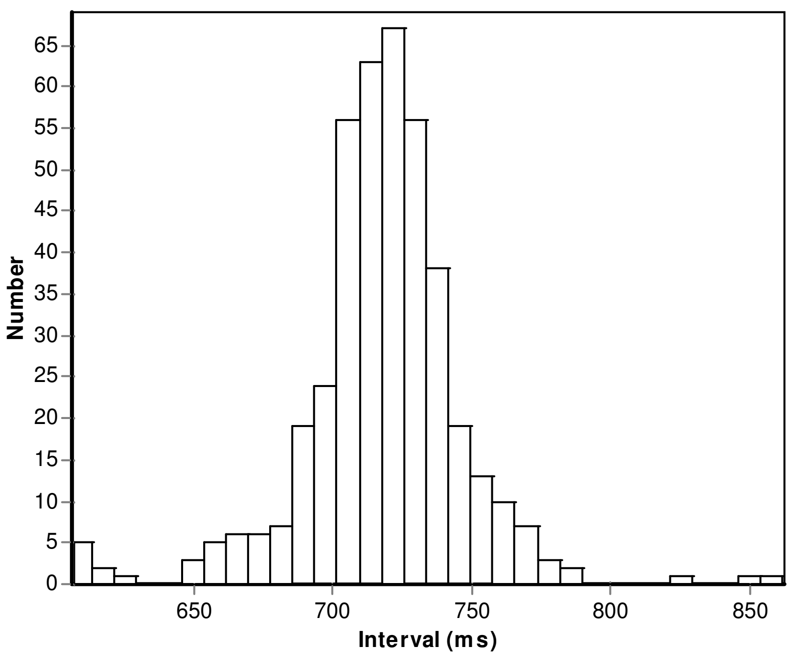

Cardio Control NV. User: Anton

Heart Rate Variability: Time Domain Analysis

**Name:**	006, 006 006	**Birthdate:**	25/01/1979
**Number:**	006	**Recorded:**	03/05/2018 18:08:27
**Gender:**	Female		

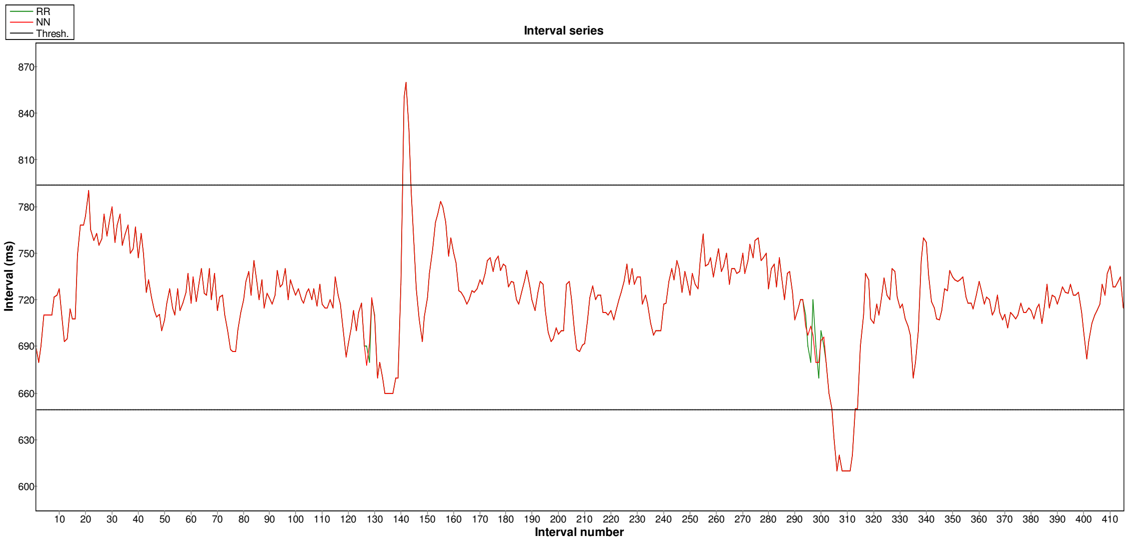

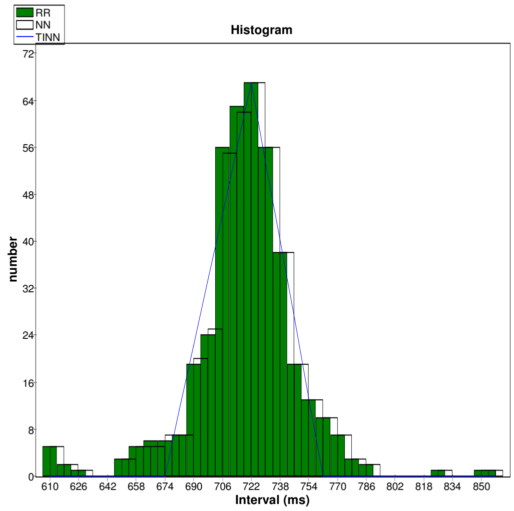

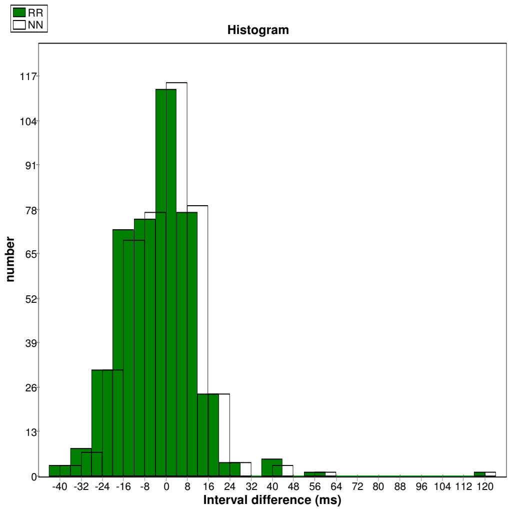

Binsize (ms) = 8

**HRV parameters**	**NN**	**RR**
SDNN (ms)	29	29
Triangular Interpolation (ms)	88	88
Triangular Index	6.19	6.19

**HRV parameters**	**NN**	**RR**
SDSD (ms)	14	14
RMSSD (ms)	14	14
NN50	2	2
NN50(1)	0	0
NN50(2)	2	2
pNN50	0.00	0.00
pNN50(1)	0.00	0.00
pNN50(2)	0.00	0.00
Logarithmic Index	0.85	0.84
SD(Logarithmic Index)	0.10	0.15

**Interval statistics**	**NN**	**RR**
Number	415	415
Minimum (ms)	610	610
Maximum (ms)	860	860
Range (ms)	250	250
Avg (ms)	722	722
SD (ms)	29	29
AvgDev (ms)	20	20
p5 (ms)	670	670
p50 (ms)	723	723
p95 (ms)	767	767
Skewness	-0.37	-0.37
Kurtosis	7.51	7.48

**Interval statistics**	**NN**	**RR**
Number	414	414
Minimum (ms)	-40	-40
Maximum (ms)	120	120
Range (ms)	160	160
Avg (ms)	0	0
SD (ms)	14	14
AvgDev (ms)	10	10
p5 (ms)	-20	-20
p50 (ms)	0	0
p95 (ms)	18	19
Skewness	1.67	1.63
Kurtosis	16.57	15.39

Heart Rate Variability: Frequency Domain Analysis

**Name:**	006, 006 006	**Birthdate:**	25/01/1979
**Number:**	006	**Recorded:**	03/05/2018 18:08:27
**Gender:**	Female		

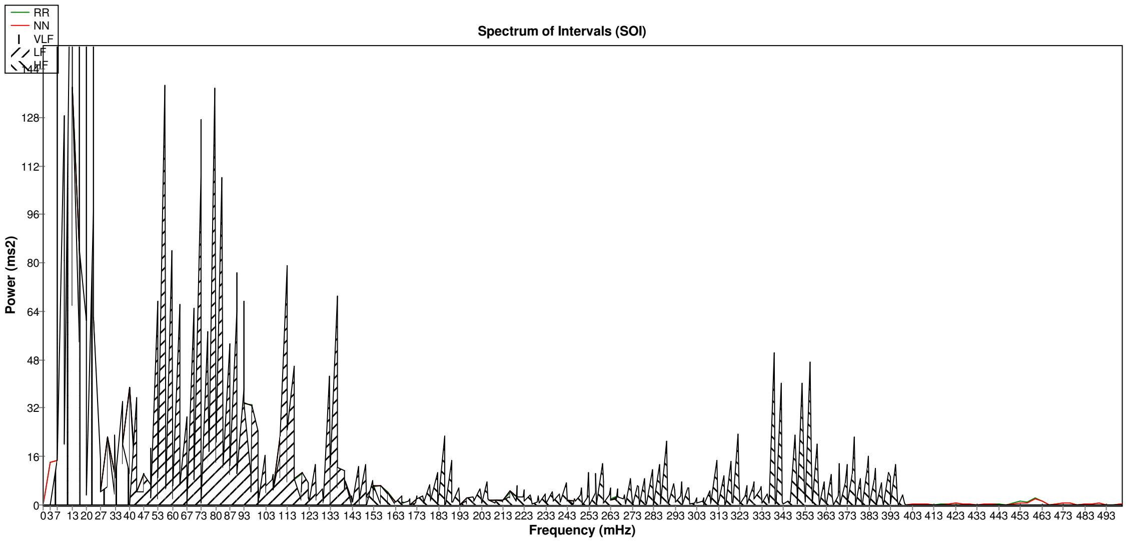

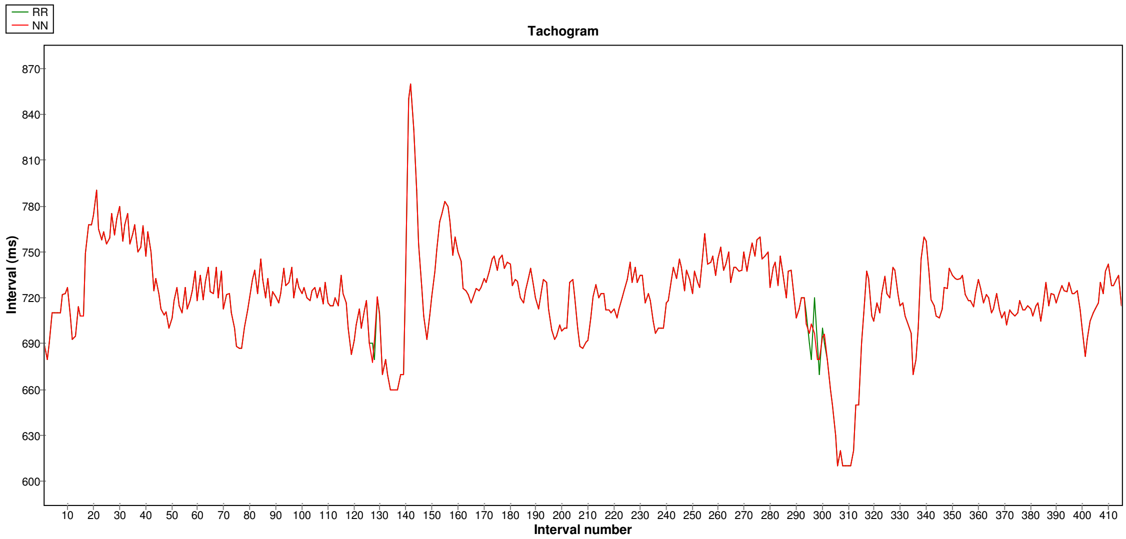

**HRV parameters**	**NN**	**RR**
TP (ms2)	816	815
VLF (ms2)	418	418
LF (ms2)	323	323
HF (ms2)	75	74
LF/HF	4.31	4.34
LF normalized	81.15	81.27
HF normalized	18.85	18.73
VLF peak (mHz)	13	13
LF peak (mHz)	93	93
HF peak (mHz)	157	157

**HRV spectral settings**	
Spectrum of Intervals (SOI)	
Frequency resolution (mHz)	3
VLF lower boundary (mHz)	3
VLF upper boundary (mHz)	40
LF upper boundary (mHz)	150
HF upper boundary (mHz)	400
Smoothing factor	1
Tapering	Hann
Fourier transform	DFT
Sample frequency (Hz)	1.39
Interval correction	Annotation
Interval threshold (%)	10
