# Supplementary material for: The effects of a 3-day mountain bike cycling race on the autonomic nervous system (ANS) and heart rate variability in amateur cyclists: a prospective quantitative research design
Source: BMC Sports Sci Med Rehabil. 2023 Jan 2;15:2. doi: 10.1186/s13102-022-00614-y (PMC9808932; doi:10.1186/s13102-022-00614-y)
Supplement: Supplementary file 1 — Additional file 1. Individual data of Participants. [file 13102_2022_614_MOESM1_ESM.zip › Individual data of Participants/HRV Data/006/ECG_006_20180504135855_.PDF]

Anton Swart Biokinetic Rehabilitation Practice

Name:	006 006 006	Recorded:	04/05/2018 13:58:55
Number:	006	Recorded by:	Mr. Anton Swart
Gender:	Female	Referring physician:	
Birthdate:	25/01/1979 39 years	Ordering physician:	
		Attending physician:	
		Location:	Anton Swart Biokinetic Rehabilitation Practi
		Comment:	

UNCONFIRMED INTERPRETATION - MD SHOULD REVIEW

P / PQ:	112 ms / 182 ms
QRS:	88 ms
QT / QTc / QTd:	366 ms / 418 ms / -
P/QRS/T axis:	69° / 85° / 71°
Heartrate:	90 bpm

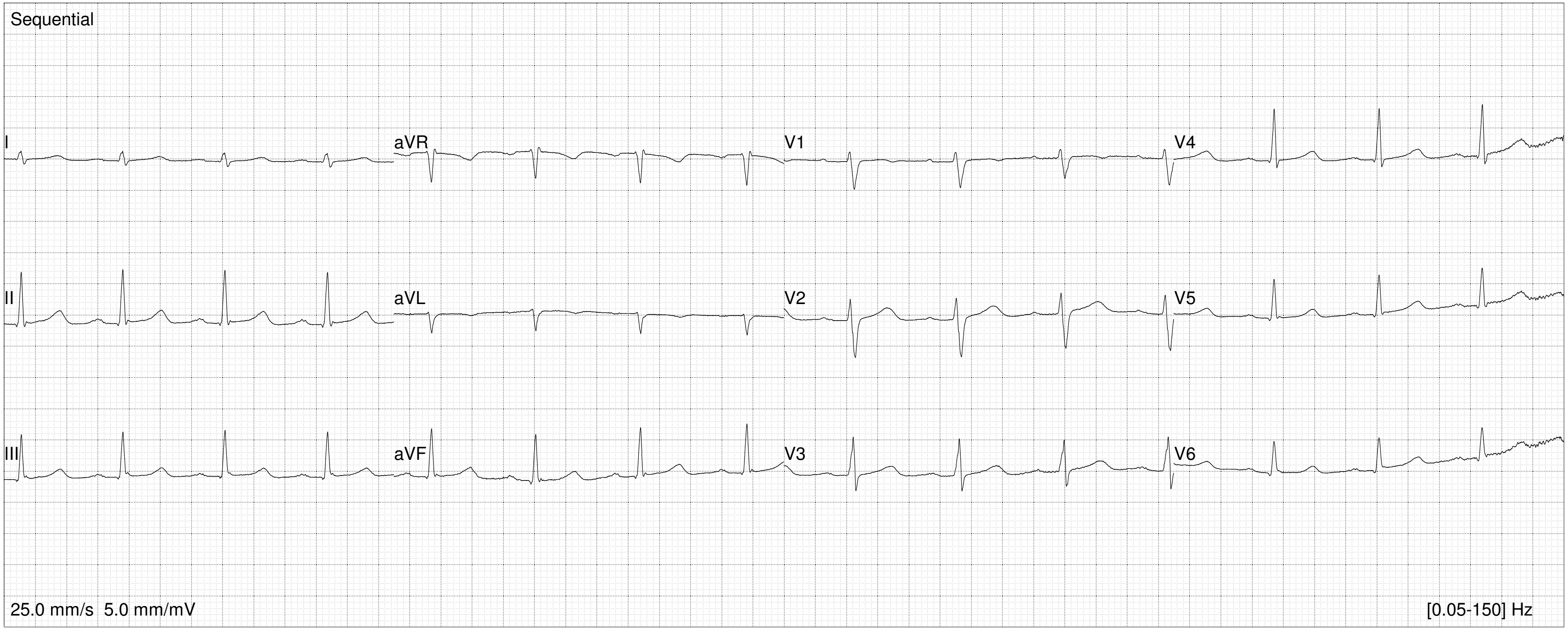

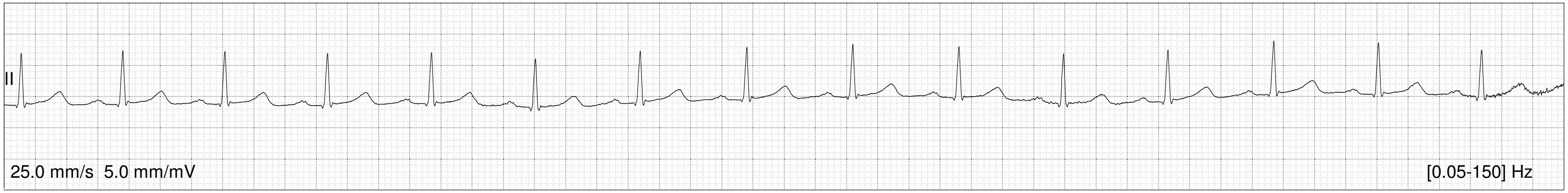

Anton Swart Biokinetic Rehabilitation Practice

Name:	006 006 006	Recorded:	04/05/2018 13:58:55	UNCONFIRMED INTERPRETATION - MD SHOULD REVIEW
Number:	006	Recorded by:	Mr. Anton Swart	
Gender:	Female	Referring physician:		
Birthdate:	25/01/1979 39 years	Location:	Anton Swart Biokinetic Rehabilitation Practice	
		Ordering physician:		
		Attending physician:		
		Comment:		

P / PQ:	112 ms / 182 ms
QRS:	88 ms
QT / QTc / QTd:	366 ms / 418 ms / -
P/QRS/T axis:	69° / 85° / 71°
Heartrate:	90 bpm

Beats		RR	
Total:	450	Minimum	610 ms
Normal:	450	Maximum	703 ms
Other:	0	Mean:	665 ms
		SD:	14 ms

R-R Trend

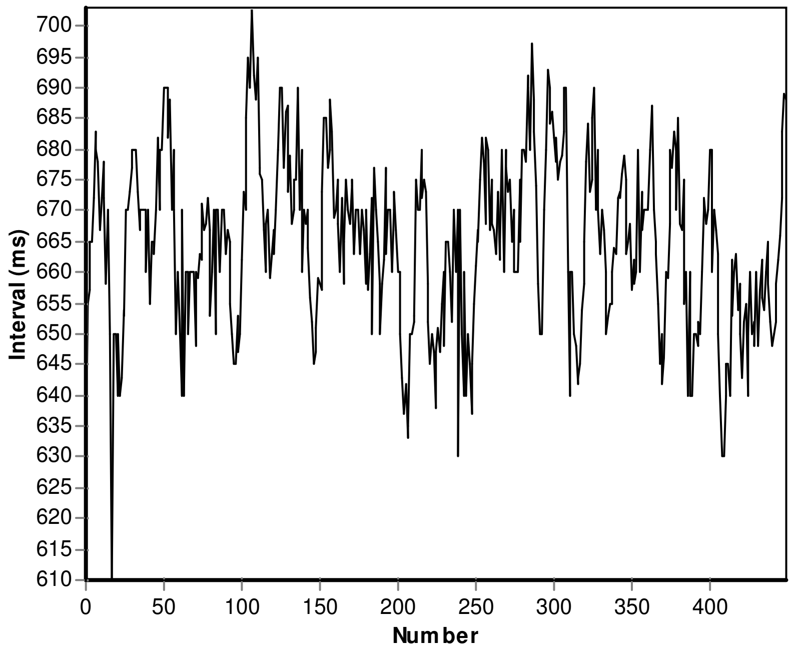

R-R Histogram

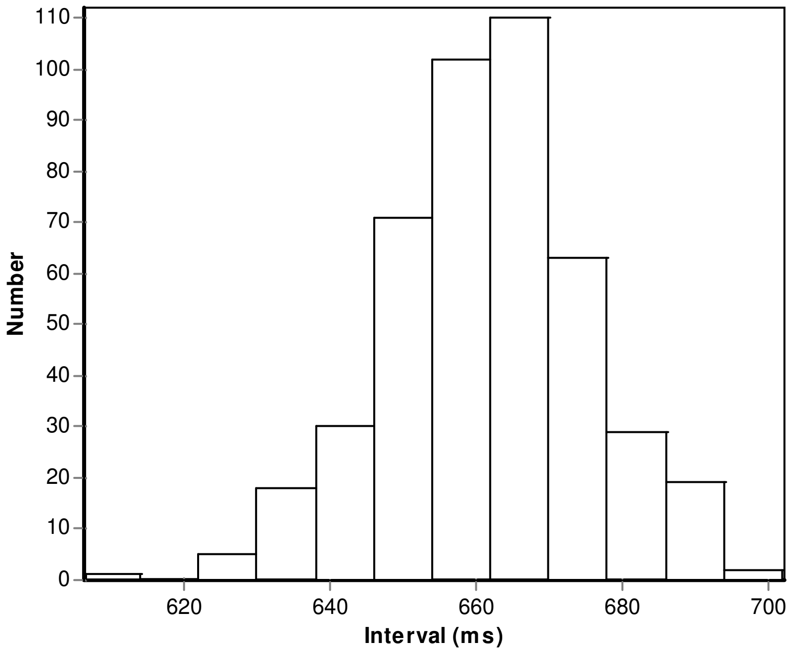

Heart Rate Variability: Time Domain Analysis

**Name:**	006, 006 006	**Birthdate:**	25/01/1979
**Number:**	006	**Recorded:**	04/05/2018 13:58:55
**Gender:**	Female		

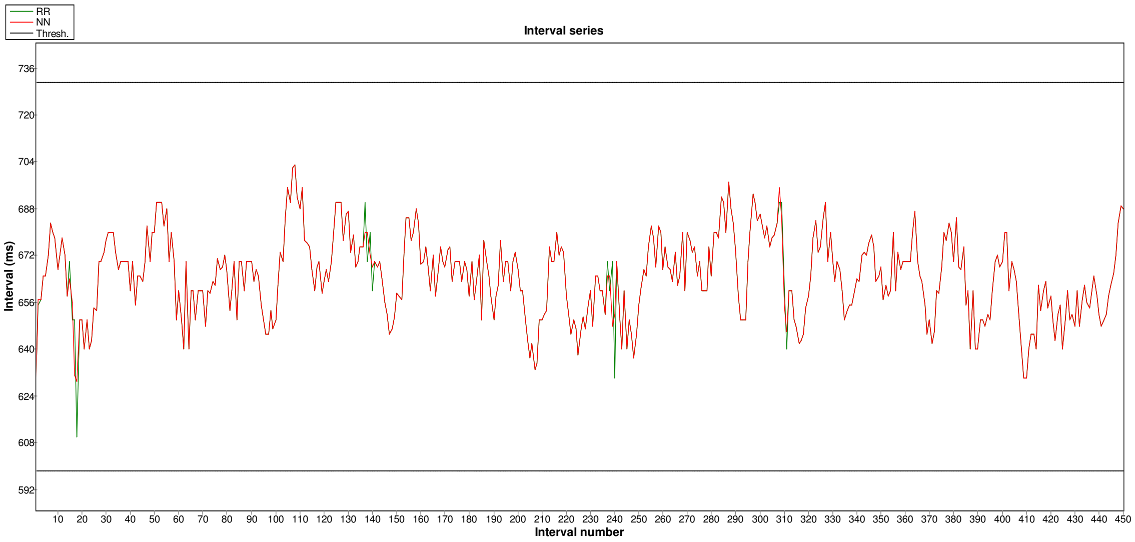

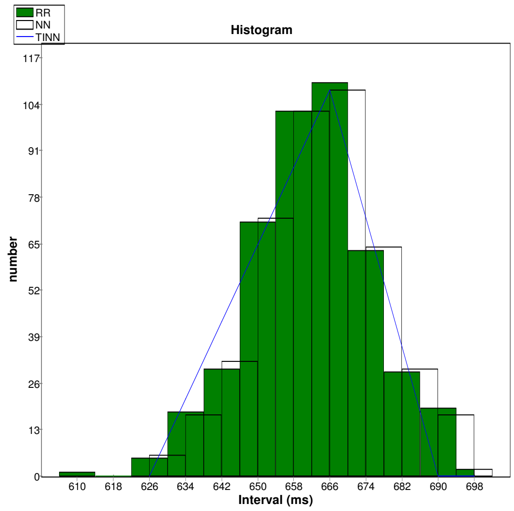

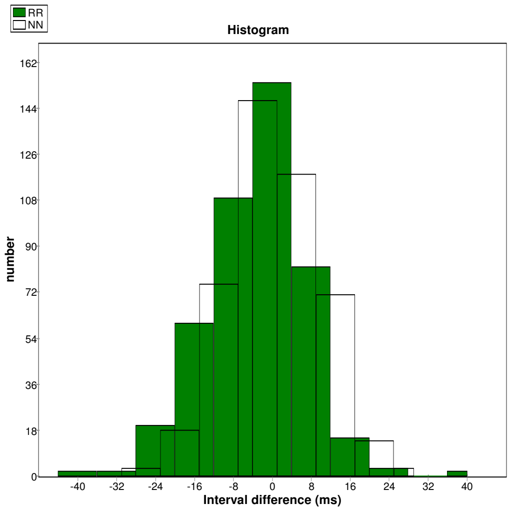

Binsize (ms) = 8

**HRV parameters**	**NN**	**RR**
SDNN (ms)	14	14
Triangular Interpolation (ms)	64	64
Triangular Index	4.17	4.09

**HRV parameters**	**NN**	**RR**
SDSD (ms)	9	10
RMSSD (ms)	9	10
NN50	0	0
NN50(1)	0	0
NN50(2)	0	0
pNN50	0.00	0.00
pNN50(1)	0.00	0.00
pNN50(2)	0.00	0.00
Logarithmic Index	1.28	1.17
SD(Logarithmic Index)	0.20	0.16

**Interval statistics**	**NN**	**RR**
Number	450	450
Minimum (ms)	626	610
Maximum (ms)	703	703
Range (ms)	77	93
Avg (ms)	665	665
SD (ms)	14	14
AvgDev (ms)	11	11
p5 (ms)	640	640
p50 (ms)	665	665
p95 (ms)	688	689
Skewness	-0.07	-0.16
Kurtosis	2.72	3.03

**Interval statistics**	**NN**	**RR**
Number	449	449
Minimum (ms)	-31	-40
Maximum (ms)	31	40
Range (ms)	62	80
Avg (ms)	0	0
SD (ms)	9	10
AvgDev (ms)	7	8
p5 (ms)	-15	-17
p50 (ms)	0	0
p95 (ms)	15	15
Skewness	-0.04	-0.07
Kurtosis	3.35	4.53

Cardio Control NV. User: Anton

Heart Rate Variability: Frequency Domain Analysis

**Name:**	006, 006 006	**Birthdate:**	25/01/1979
**Number:**	006	**Recorded:**	04/05/2018 13:58:55
**Gender:**	Female		

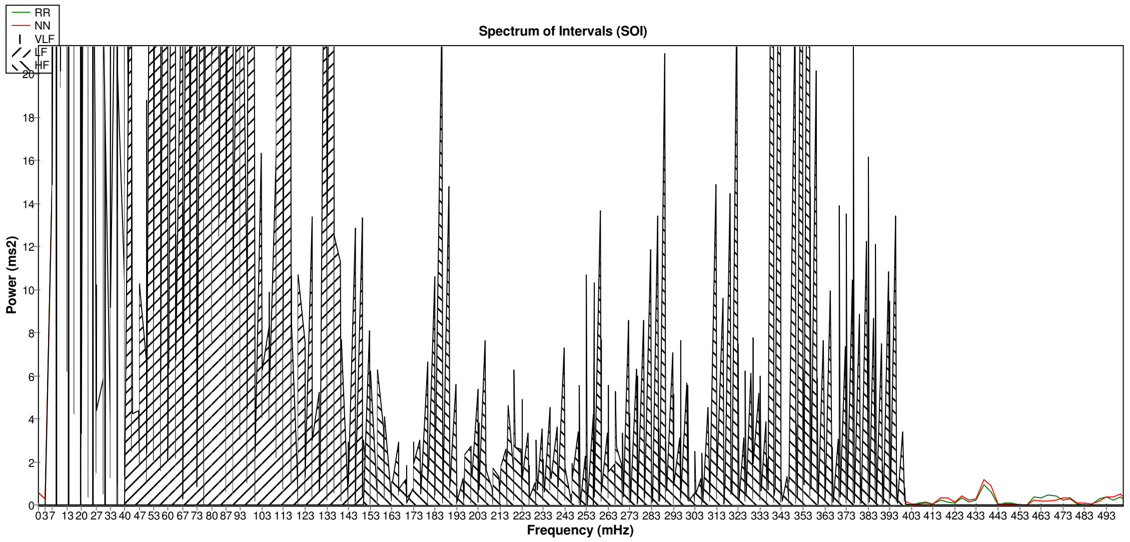

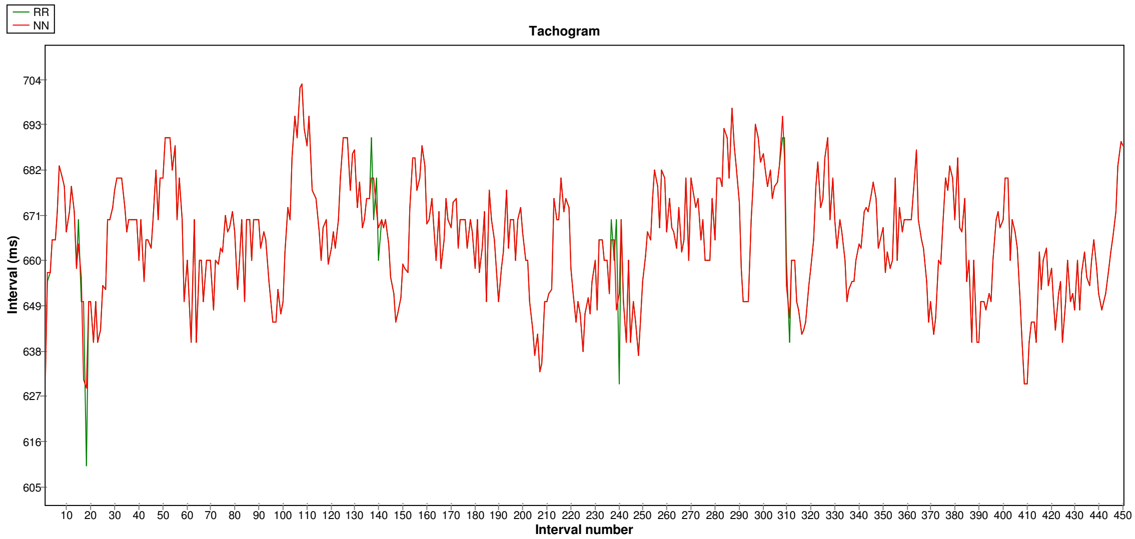

**HRV parameters**	**NN**	**RR**
TP (ms2)	160	161
VLF (ms2)	54	54
LF (ms2)	92	93
HF (ms2)	14	15
LF/HF	6.53	6.37
LF normalized	86.73	86.43
HF normalized	13.27	13.57
VLF peak (mHz)	10	10
LF peak (mHz)	70	70
HF peak (mHz)	176	176

**HRV spectral settings**	
Spectrum of Intervals (SOI)	
Frequency resolution (mHz)	3
VLF lower boundary (mHz)	3
VLF upper boundary (mHz)	40
LF upper boundary (mHz)	150
HF upper boundary (mHz)	400
Smoothing factor	1
Tapering	Hann
Fourier transform	DFT
Sample frequency (Hz)	1.50
Interval correction	Annotation
Interval threshold (%)	10
