# Supplementary material for: The effects of a 3-day mountain bike cycling race on the autonomic nervous system (ANS) and heart rate variability in amateur cyclists: a prospective quantitative research design
Source: BMC Sports Sci Med Rehabil. 2023 Jan 2;15:2. doi: 10.1186/s13102-022-00614-y (PMC9808932; doi:10.1186/s13102-022-00614-y)
Supplement: Supplementary file 1 — Additional file 1. Individual data of Participants. [file 13102_2022_614_MOESM1_ESM.zip › Individual data of Participants/HRV Data/006/ECG_006_20180505135425_.PDF]

Anton Swart Biokinetic Rehabilitation Practice

Name:	006 006 006
Number:	006
Gender:	Female
Birthdate:	25/01/1979 39 years

Recorded:	05/05/2018 13:54:25
Recorded by:	Mr. Anton Swart
Referring physician:	
Ordering physician:	
Attending physician:	
Location:	Anton Swart Biokinetic Rehabilitation Practi
Comment:	

UNCONFIRMED INTERPRETATION - MD SHOULD REVIEW

P / PQ:	108 ms / 172 ms
QRS:	86 ms
QT / QTc / QTd:	370 ms / 422 ms / -
P/QRS/T axis:	70° / 85° / 69°
Heartrate:	90 bpm

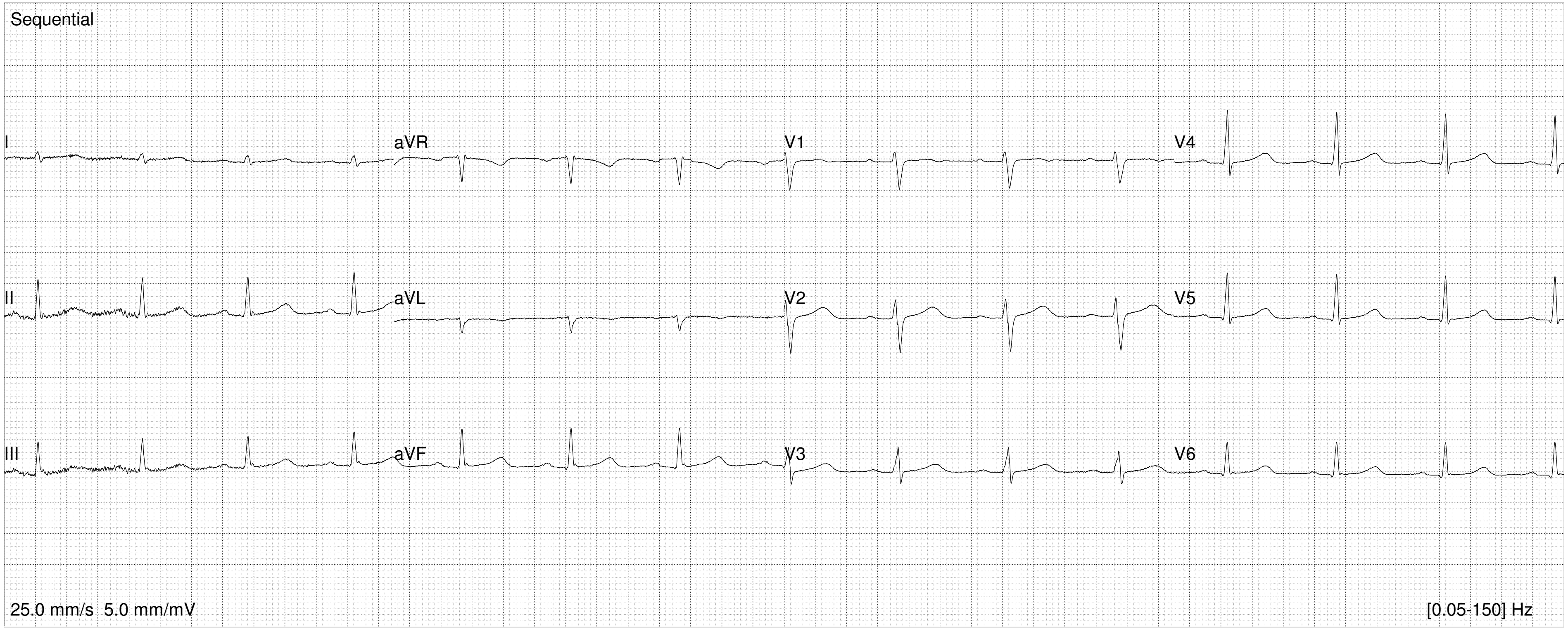

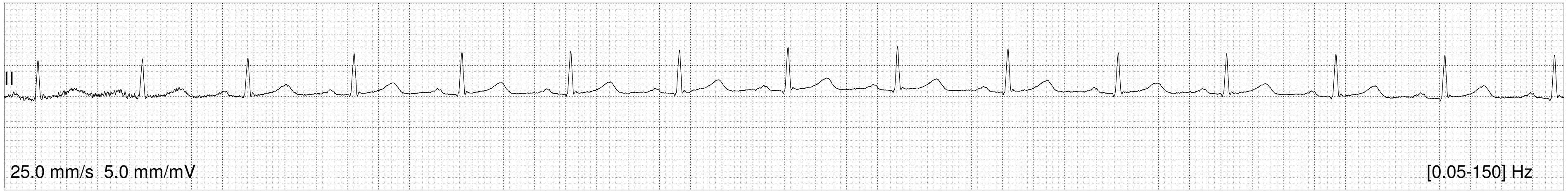

Anton Swart Biokinetic Rehabilitation Practice

Name:	006 006 006	Recorded:	05/05/2018 13:54:25	UNCONFIRMED INTERPRETATION - MD SHOULD REVIEW
Number:	006	Recorded by:	Mr. Anton Swart	
Gender:	Female	Referring physician:		
Birthdate:	25/01/1979 39 years	Location:	Anton Swart Biokinetic Rehabilitation Practice	
		Ordering physician:		
		Attending physician:		
		Comment:		

P / PQ:	108 ms / 172 ms
QRS:	86 ms
QT / QTc / QTd:	370 ms / 422 ms / -
P/QRS/T axis:	70° / 85° / 69°
Heartrate:	90 bpm

Beats

Total:	449
Normal:	449
Other:	0

RR

Minimum	630 ms
Maximum	710 ms
Mean:	667 ms
SD:	13 ms

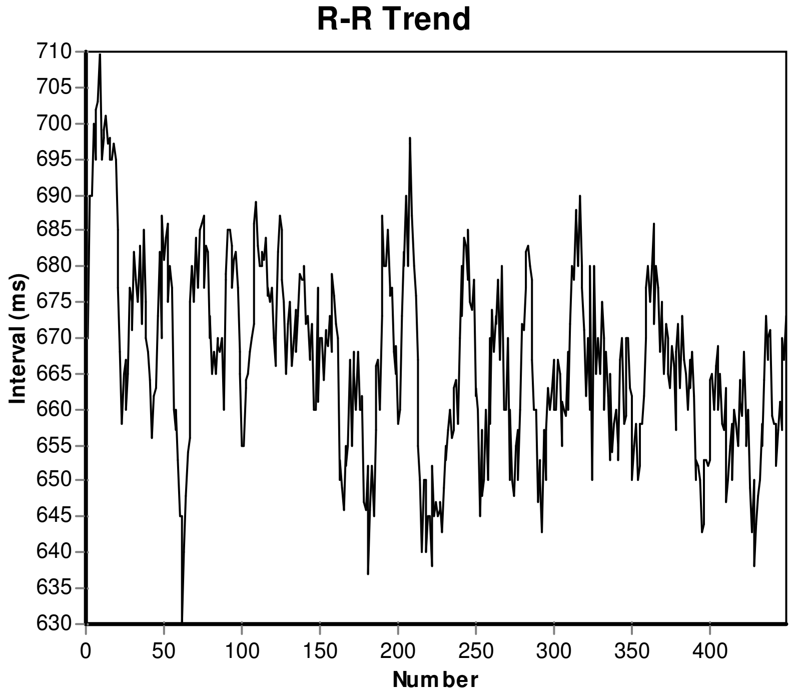

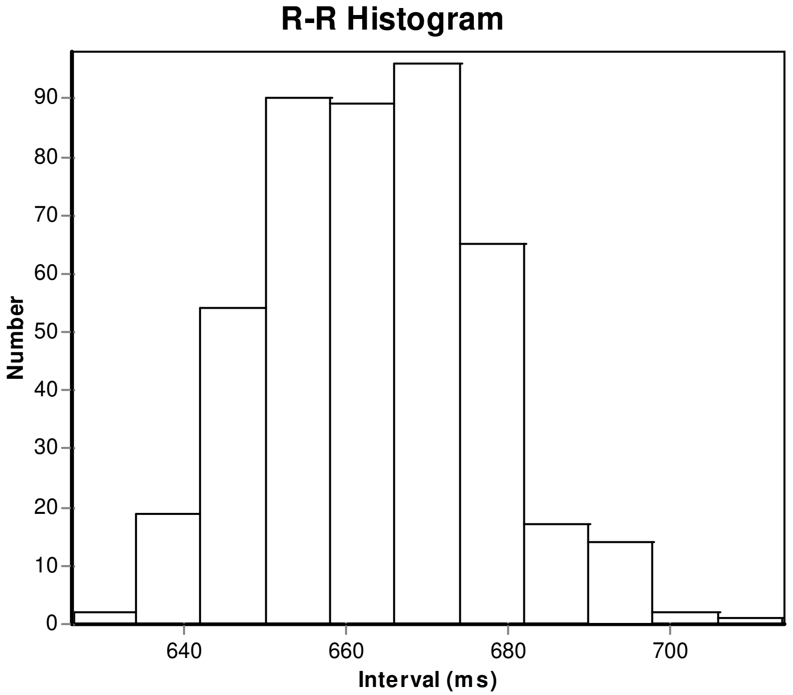

Heart Rate Variability: Time Domain Analysis

Name: 006, 006 006
Number: 006
Gender: Female
Birthdate: 25/01/1979
Recorded: 05/05/2018 13:54:25

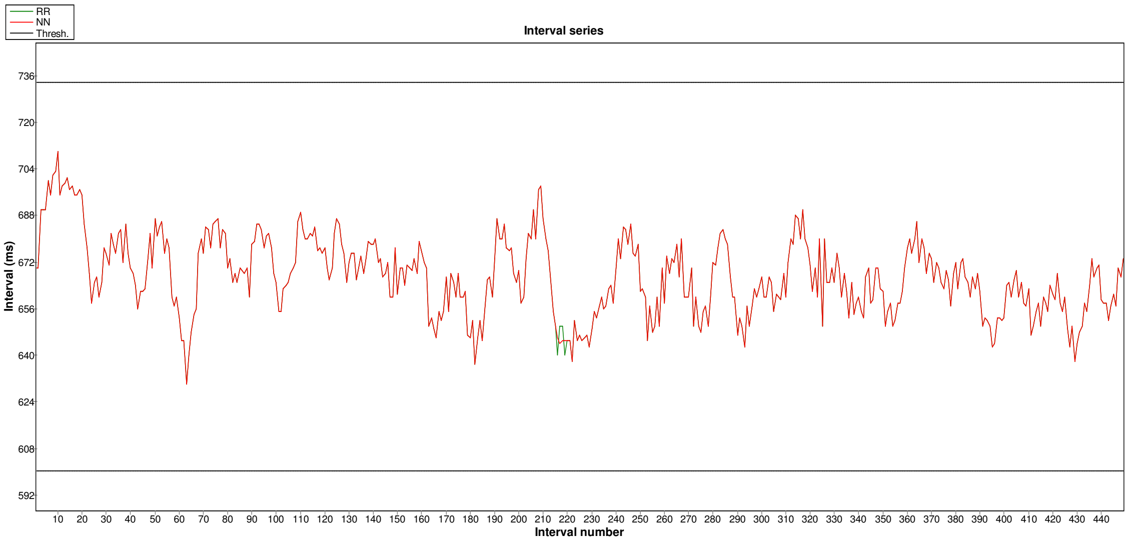

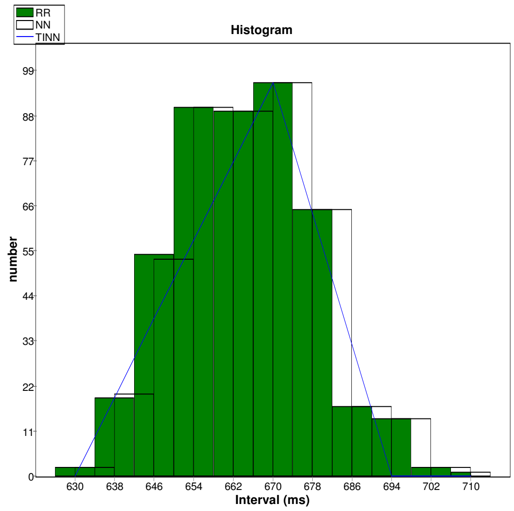

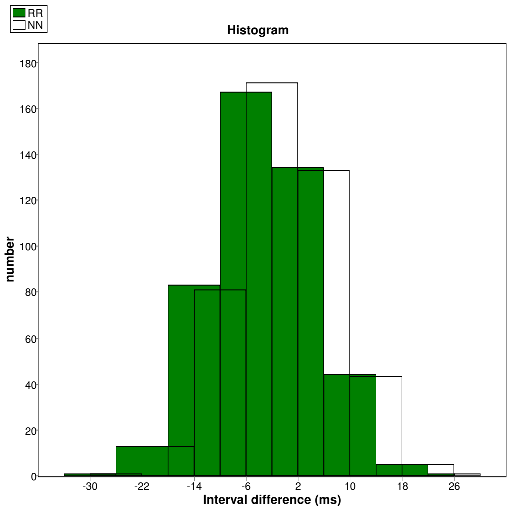

Binsize (ms) = 8

**HRV parameters**	**NN**	**RR**
SDNN (ms)	13	13
Triangular Interpolation (ms)	64	64
Triangular Index	4.68	4.68

**HRV parameters**	**NN**	**RR**
SDSD (ms)	8	8
RMSSD (ms)	8	8
NN50	0	0
NN50(1)	0	0
NN50(2)	0	0
pNN50	0.00	0.00
pNN50(1)	0.00	0.00
pNN50(2)	0.00	0.00
Logarithmic Index	1.71	1.71
SD(Logarithmic Index)	0.22	0.22

**Interval statistics**	**NN**	**RR**
Number	449	449
Minimum (ms)	630	630
Maximum (ms)	710	710
Range (ms)	80	80
Avg (ms)	667	667
SD (ms)	13	13
AvgDev (ms)	11	11
p5 (ms)	646	646
p50 (ms)	667	667
p95 (ms)	690	690
Skewness	0.23	0.22
Kurtosis	2.86	2.88

**Interval statistics**	**NN**	**RR**
Number	448	448
Minimum (ms)	-30	-30
Maximum (ms)	30	30
Range (ms)	60	60
Avg (ms)	0	0
SD (ms)	8	8
AvgDev (ms)	6	6
p5 (ms)	-12	-12
p50 (ms)	0	0
p95 (ms)	12	12
Skewness	0.03	0.03
Kurtosis	3.44	3.38

Cardio Control NV. User: Anton

Heart Rate Variability: Frequency Domain Analysis

**Name:**	006, 006 006	**Birthdate:**	25/01/1979
**Number:**	006	**Recorded:**	05/05/2018 13:54:25
**Gender:**	Female		

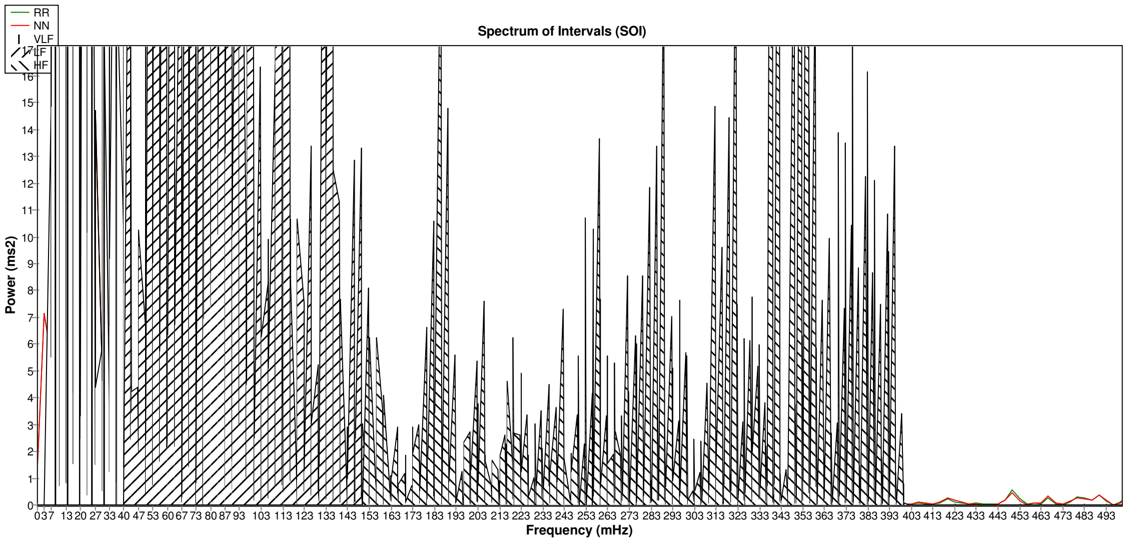

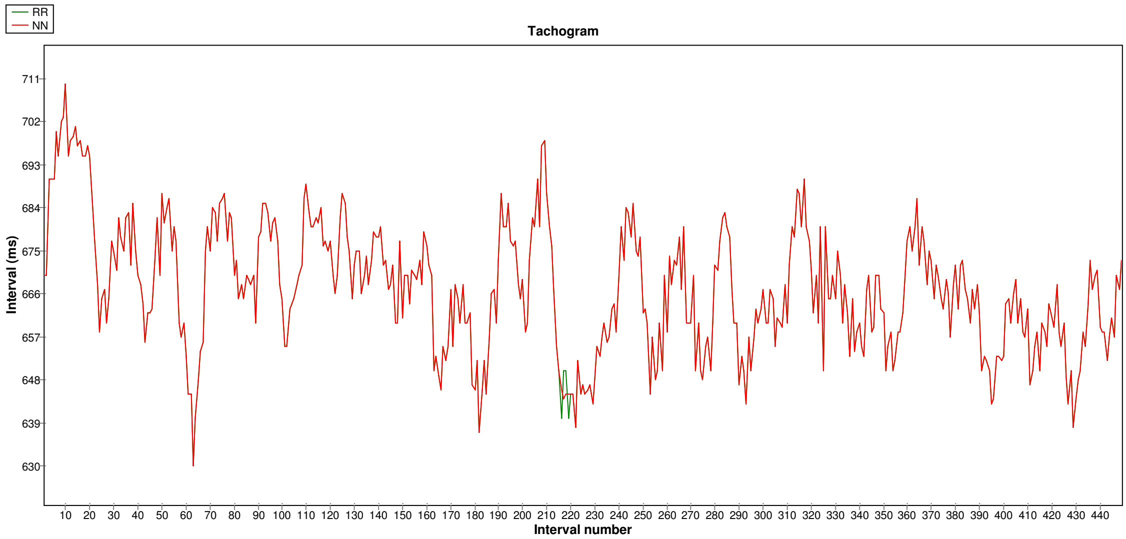

**HRV parameters**	**NN**	**RR**
TP (ms2)	146	147
VLF (ms2)	71	71
LF (ms2)	70	70
HF (ms2)	5	6
LF/HF	13.36	12.30
LF normalized	93.04	92.48
HF normalized	6.96	7.52
VLF peak (mHz)	37	37
LF peak (mHz)	87	87
HF peak (mHz)	333	333

**HRV spectral settings**	
Spectrum of Intervals (SOI)	
Frequency resolution (mHz)	3
VLF lower boundary (mHz)	3
VLF upper boundary (mHz)	40
LF upper boundary (mHz)	150
HF upper boundary (mHz)	400
Smoothing factor	1
Tapering	Hann
Fourier transform	DFT
Sample frequency (Hz)	1.50
Interval correction	Annotation
Interval threshold (%)	10
