# Supplementary material for: The effects of a 3-day mountain bike cycling race on the autonomic nervous system (ANS) and heart rate variability in amateur cyclists: a prospective quantitative research design
Source: BMC Sports Sci Med Rehabil. 2023 Jan 2;15:2. doi: 10.1186/s13102-022-00614-y (PMC9808932; doi:10.1186/s13102-022-00614-y)
Supplement: Supplementary file 1 — Additional file 1. Individual data of Participants. [file 13102_2022_614_MOESM1_ESM.zip › Individual data of Participants/HRV Data/006/ECG_006_20180506065014_.PDF]

Anton Swart Biokinetic Rehabilitation Practice

Name:	006 006 006	Recorded:	06/05/2018 06:50:14	UNCONFIRMED INTERPRETATION - MD SHOULD REVIEW
Number:	006	Recorded by:	Mr. Anton Swart	
Gender:	Female	Referring physician:		
Birthdate:	25/01/1979 39 years	Ordering physician:		
		Attending physician:		
P / PQ:	100 ms / 173 ms	Location:	Anton Swart Biokinetic Rehabilitation Practi	
QRS:	82 ms	Comment:		
QT / QTc / QTd:	420 ms / 444 ms / -			
P/QRS/T axis:	75° / 86° / 77°			
Heartrate:	74 bpm			

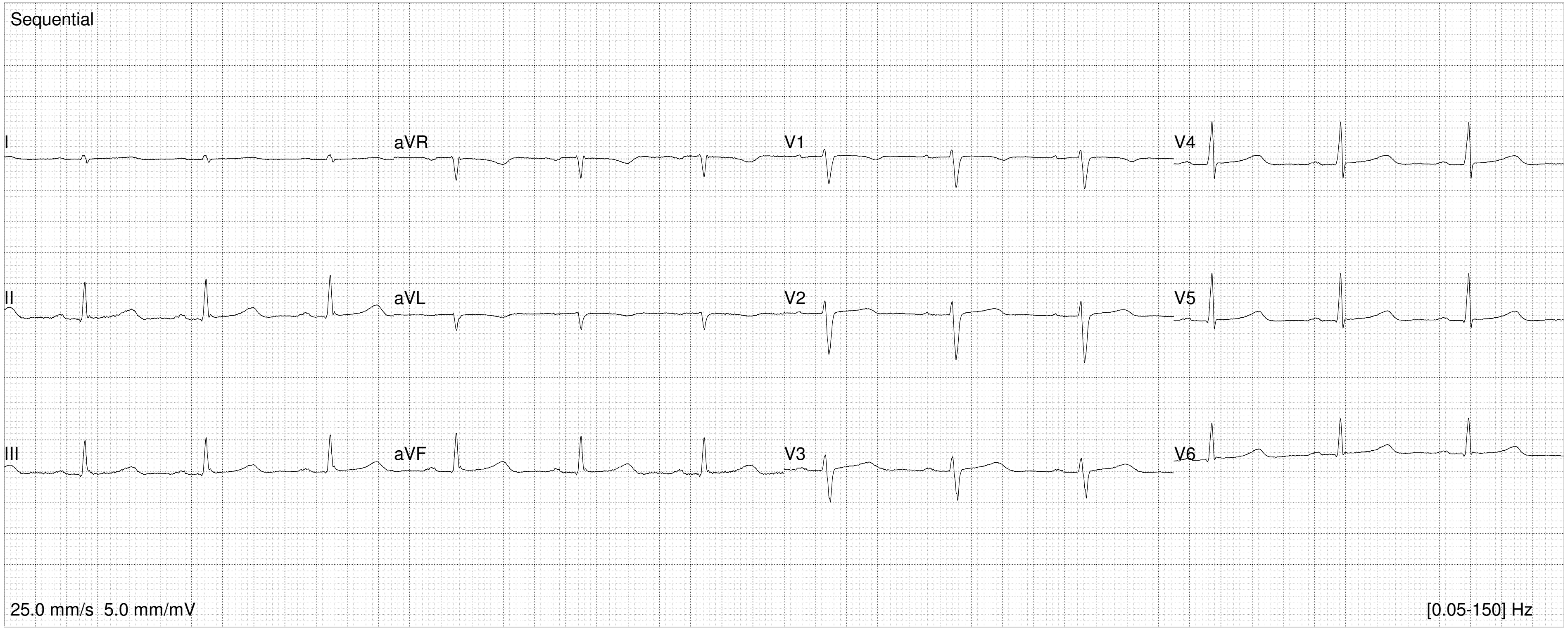

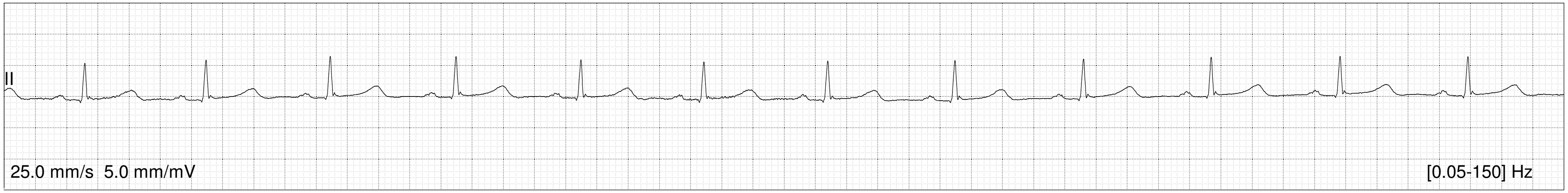

Anton Swart Biokinetic Rehabilitation Practice

Name:	006 006 006	Recorded:	06/05/2018 06:50:14	UNCONFIRMED INTERPRETATION - MD SHOULD REVIEW
Number:	006	Recorded by:	Mr. Anton Swart	
Gender:	Female	Referring physician:		
Birthdate:	25/01/1979 39 years	Location:	Anton Swart Biokinetic Rehabilitation Practice	
P / PQ:	100 ms / 173 ms	Ordering physician:		
QRS:	82 ms	Attending physician:		
QT / QTc / QTd:	420 ms / 444 ms / -	Comment:		
P/QRS/T axis:	75° / 86° / 77°			
Heartrate:	74 bpm			

Beats

Total:	369
Normal:	369
Other:	0

RR

Minimum	720 ms
Maximum	930 ms
Mean:	809 ms
SD:	34 ms

R-R Trend

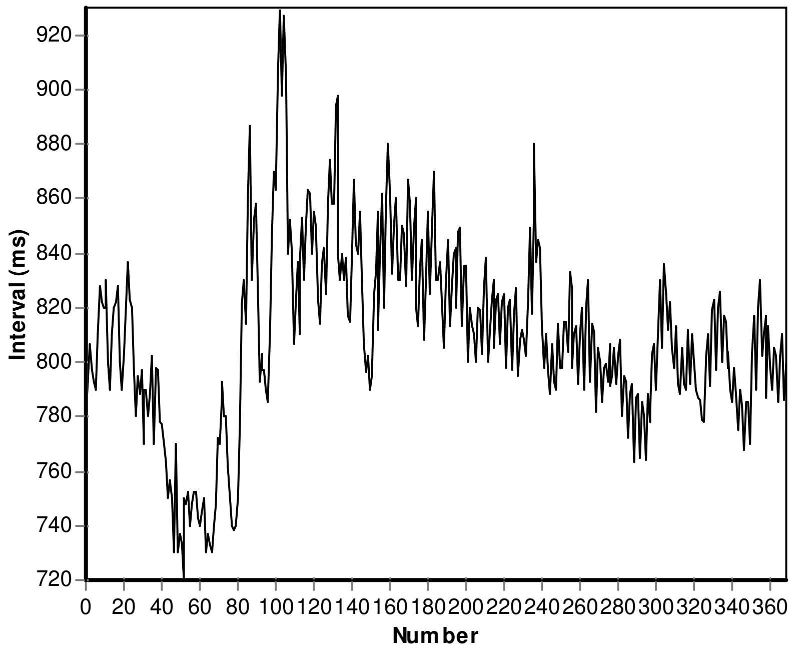

R-R Histogram

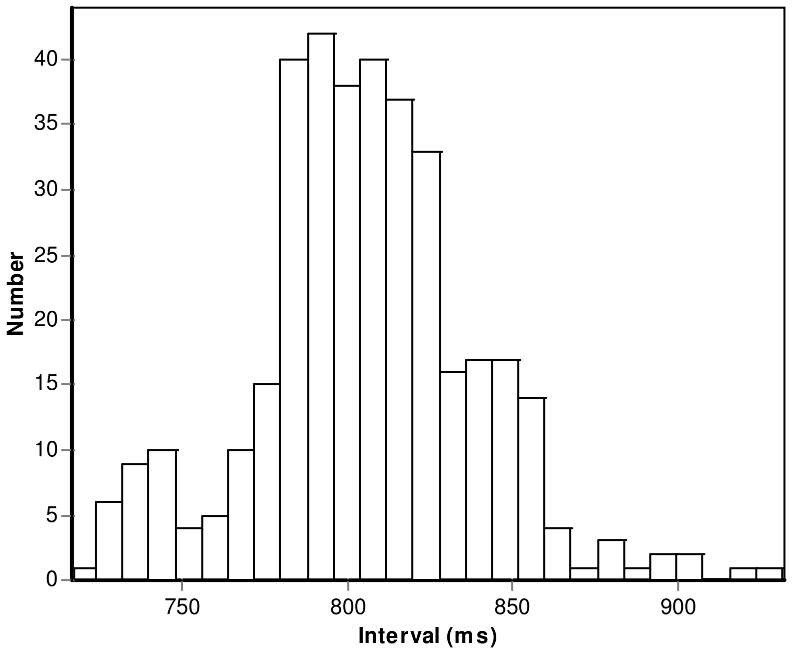

Heart Rate Variability: Time Domain Analysis

**Name:**	006, 006 006	**Birthdate:**	25/01/1979
**Number:**	006	**Recorded:**	06/05/2018 06:50:14
**Gender:**	Female		

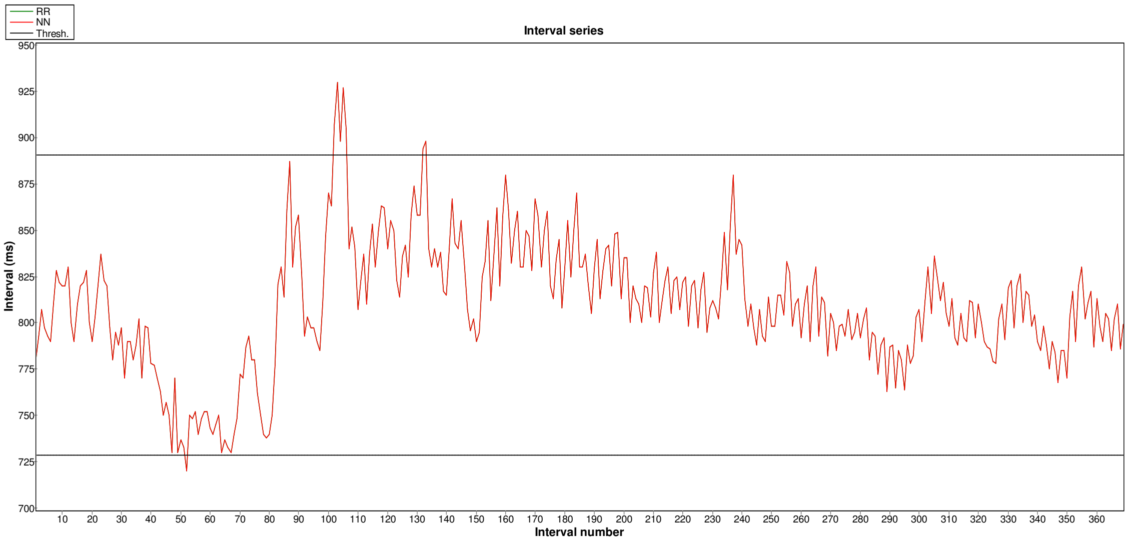

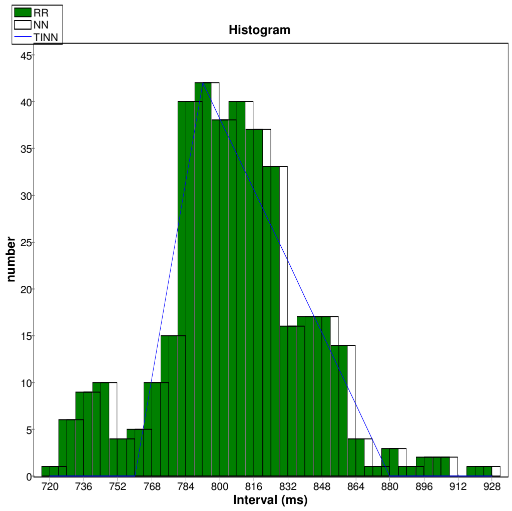

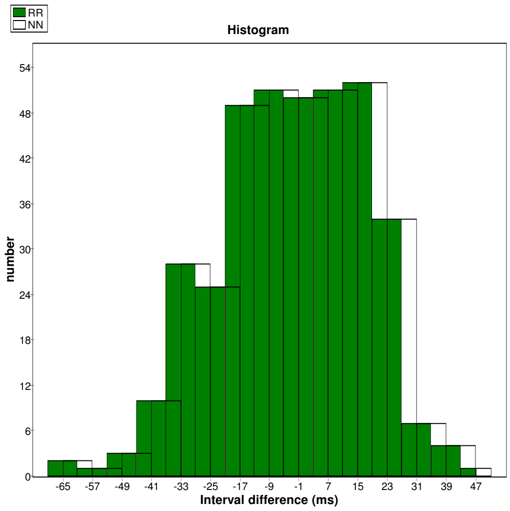

Binsize (ms) = 8

HRV parameters	NN	RR
SDNN (ms)	34	34
Triangular Interpolation (ms)	120	120
Triangular Index	8.79	8.79

HRV parameters	NN	RR
SDSD (ms)	20	20
RMSSD (ms)	20	20
NN50	3	3
NN50(1)	3	3
NN50(2)	0	0
pNN50	0.01	0.01
pNN50(1)	0.01	0.01
pNN50(2)	0.00	0.00
Logarithmic Index	0.70	0.70
SD(Logarithmic Index)	0.09	0.09

Interval statistics	NN	RR
Number	369	369
Minimum (ms)	720	720
Maximum (ms)	930	930
Range (ms)	210	210
Avg (ms)	809	809
SD (ms)	34	34
AvgDev (ms)	26	26
p5 (ms)	748	748
p50 (ms)	810	810
p95 (ms)	862	862
Skewness	0.15	0.15
Kurtosis	3.80	3.80

Interval statistics	NN	RR
Number	368	368
Minimum (ms)	-65	-65
Maximum (ms)	47	47
Range (ms)	112	112
Avg (ms)	0	0
SD (ms)	20	20
AvgDev (ms)	16	16
p5 (ms)	-32	-32
p50 (ms)	0	0
p95 (ms)	29	29
Skewness	-0.29	-0.29
Kurtosis	2.75	2.75

Cardio Control NV. User: Anton

Heart Rate Variability: Frequency Domain Analysis

**Name:**	006, 006 006	**Birthdate:**	25/01/1979
**Number:**	006	**Recorded:**	06/05/2018 06:50:14
**Gender:**	Female		

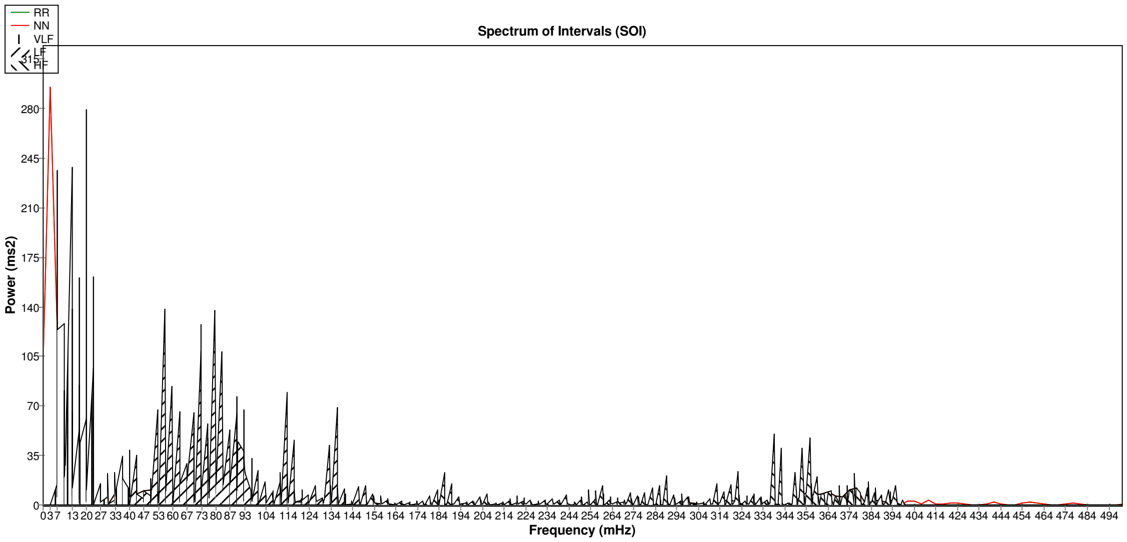

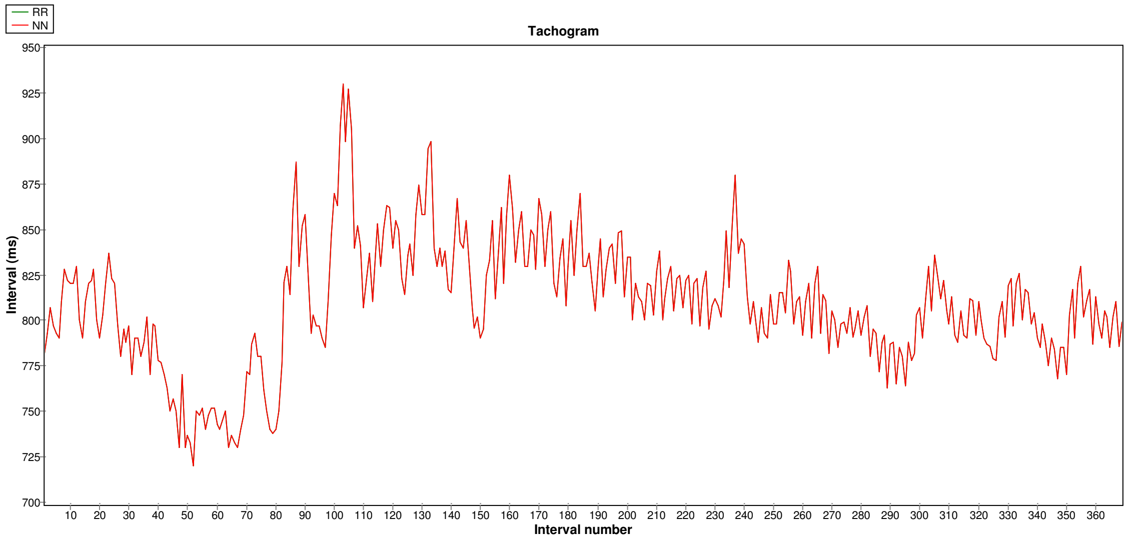

HRV parameters	NN	RR
TP (ms2)	609	609
VLF (ms2)	253	253
LF (ms2)	232	232
HF (ms2)	124	124
LF/HF	1.86	1.86
LF normalized	65.07	65.07
HF normalized	34.93	34.93
VLF peak (mHz)	7	7
LF peak (mHz)	90	90
HF peak (mHz)	377	377

HRV spectral settings	
Spectrum of Intervals (SOI)	
Frequency resolution (mHz)	3
VLF lower boundary (mHz)	3
VLF upper boundary (mHz)	40
LF upper boundary (mHz)	150
HF upper boundary (mHz)	400
Smoothing factor	1
Tapering	Hann
Fourier transform	DFT
Sample frequency (Hz)	1.24
Interval correction	Annotation
Interval threshold (%)	10
