# Supplementary material for: The effects of a 3-day mountain bike cycling race on the autonomic nervous system (ANS) and heart rate variability in amateur cyclists: a prospective quantitative research design
Source: BMC Sports Sci Med Rehabil. 2023 Jan 2;15:2. doi: 10.1186/s13102-022-00614-y (PMC9808932; doi:10.1186/s13102-022-00614-y)
Supplement: Supplementary file 1 — Additional file 1. Individual data of Participants. [file 13102_2022_614_MOESM1_ESM.zip › Individual data of Participants/HRV Data/007/ECG_007_20180501091051_.PDF]

Anton Swart Biokinetic Rehabilitation Practice

Name:	007 007 007	Recorded:	01/05/2018 09:10:51	UNCONFIRMED INTERPRETATION - MD SHOULD REVIEW
Number:	007	Recorded by:	Mr. Anton Swart	
Gender:	Male	Referring physician:		
Birthdate:	25/12/1976 41 years	Ordering physician:		
		Attending physician:		
P / PQ:	113 ms / 155 ms	Location:	Anton Swart Biokinetic Rehabilitation Practi	
QRS:	103 ms	Comment:		
QT / QTc / QTd:	418 ms / 414 ms / -			
P/QRS/T axis:	72° / 84° / 68°			
Heartrate:	58 bpm			

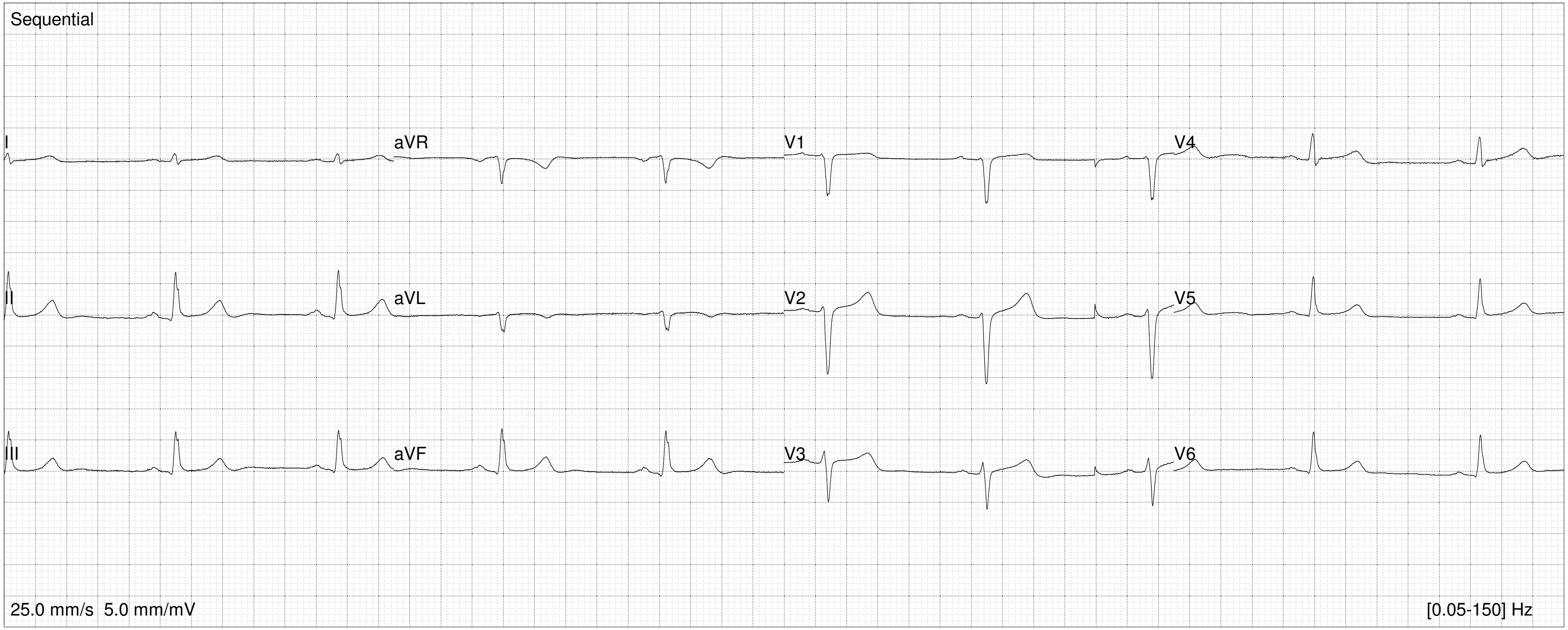

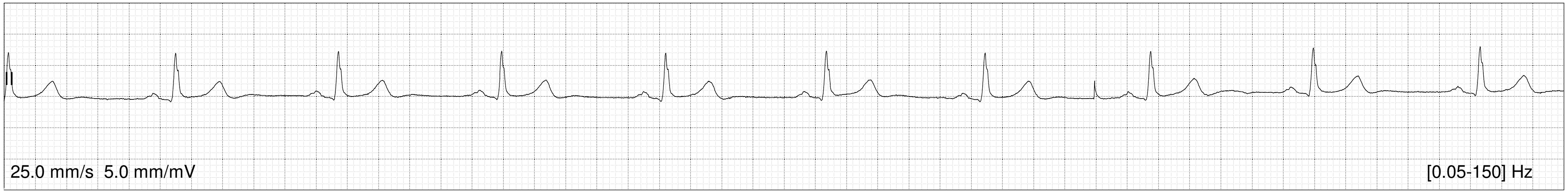

Anton Swart Biokinetic Rehabilitation Practice

Name:	007 007 007	Recorded:	01/05/2018 09:10:51	UNCONFIRMED INTERPRETATION - MD SHOULD REVIEW
Number:	007	Recorded by:	Mr. Anton Swart	
Gender:	Male	Referring physician:		
Birthdate:	25/12/1976 41 years	Location:	Anton Swart Biokinetic Rehabilitation Practice	
P / PQ:	113 ms / 155 ms	Ordering physician:		
QRS:	103 ms	Attending physician:		
QT / QTc / QTd:	418 ms / 414 ms / -	Comment:		
P/QRS/T axis:	72° / 84° / 68°			
Heartrate:	58 bpm			

**Beats**		**RR**	
Total:	292	Minimum	530 ms
Normal:	292	Maximum	1420 ms
Other:	0	Mean:	1025 ms
		SD:	63 ms

R-R Trend

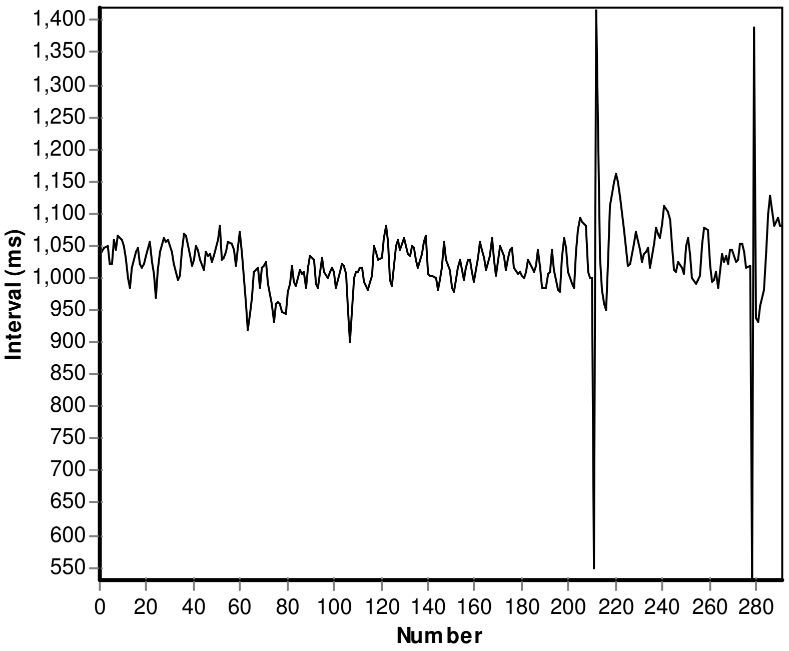

R-R Histogram

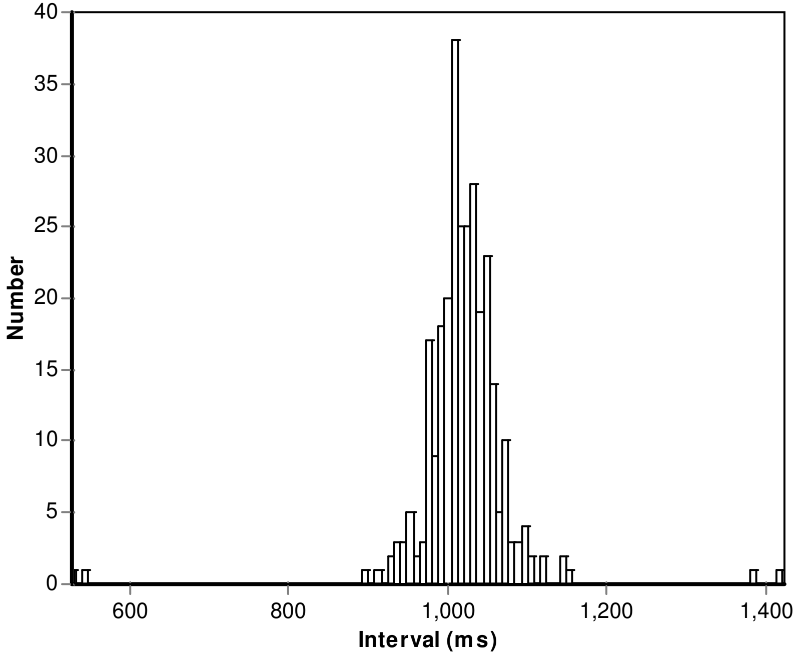

Heart Rate Variability: Time Domain Analysis

**Name:**	007, 007 007	**Birthdate:**	25/12/1976
**Number:**	007	**Recorded:**	01/05/2018 09:10:51
**Gender:**	Male		

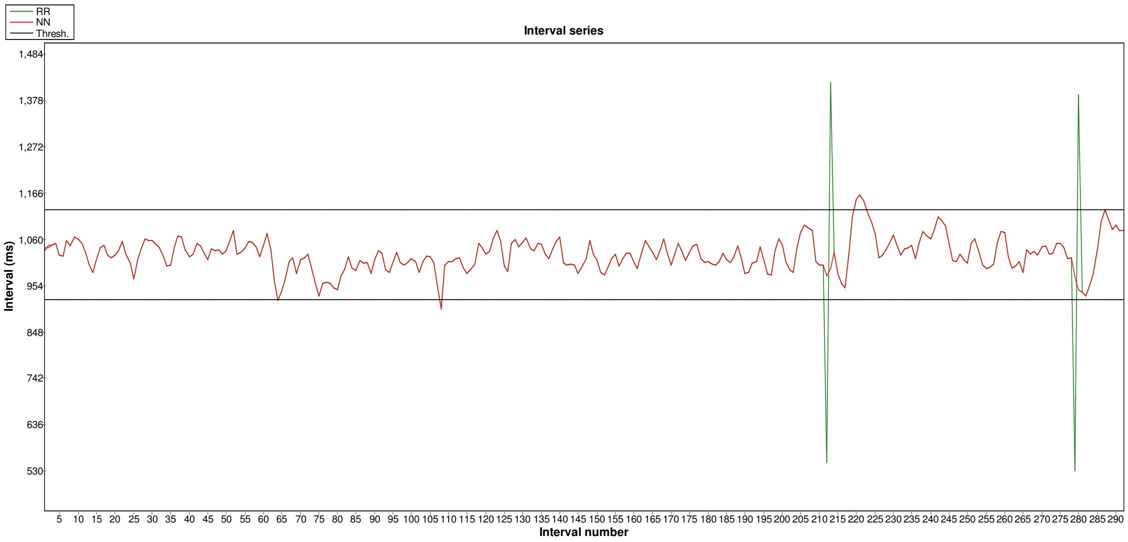

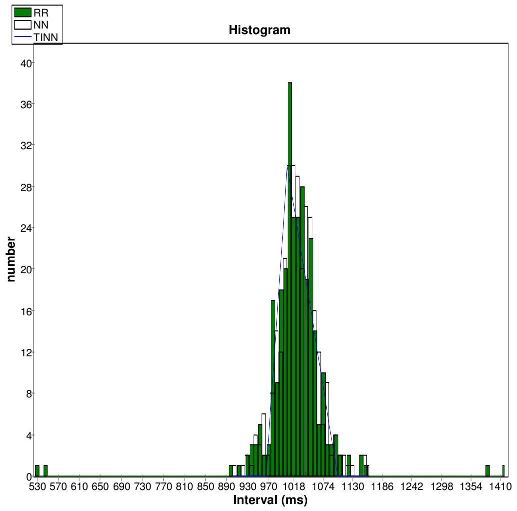

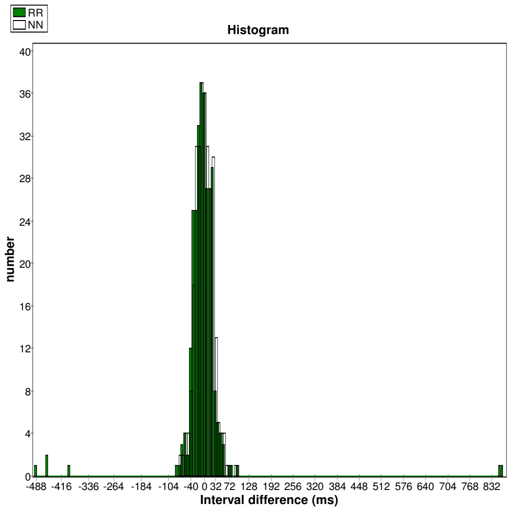

Binsize (ms) = 8

**HRV parameters**	**NN**	**RR**
SDNN (ms)	38	63
Triangular Interpolation (ms)	136	120
Triangular Index	9.73	7.68

**HRV parameters**	**NN**	**RR**
SDSD (ms)	26	92
RMSSD (ms)	26	92
NN50	17	23
NN50(1)	8	12
NN50(2)	9	11
pNN50	0.06	0.08
pNN50(1)	0.03	0.04
pNN50(2)	0.03	0.04
Logarithmic Index	0.46	0.31
SD(Logarithmic Index)	0.04	0.04

**Interval statistics**	**NN**	**RR**
Number	292	292
Minimum (ms)	901	530
Maximum (ms)	1161	1420
Range (ms)	260	890
Avg (ms)	1025	1025
SD (ms)	38	63
AvgDev (ms)	29	34
p5 (ms)	960	960
p50 (ms)	1025	1025
p95 (ms)	1089	1093
Skewness	0.19	-1.55
Kurtosis	4.23	33.28

**Interval statistics**	**NN**	**RR**
Number	291	291
Minimum (ms)	-74	-488
Maximum (ms)	99	870
Range (ms)	173	1358
Avg (ms)	0	0
SD (ms)	26	92
AvgDev (ms)	20	32
p5 (ms)	-38	-43
p50 (ms)	-1	-1
p95 (ms)	42	46
Skewness	0.25	4.08
Kurtosis	3.80	60.60

Heart Rate Variability: Frequency Domain Analysis

**Name:**	007, 007 007	**Birthdate:**	25/12/1976
**Number:**	007	**Recorded:**	01/05/2018 09:10:51
**Gender:**	Male		

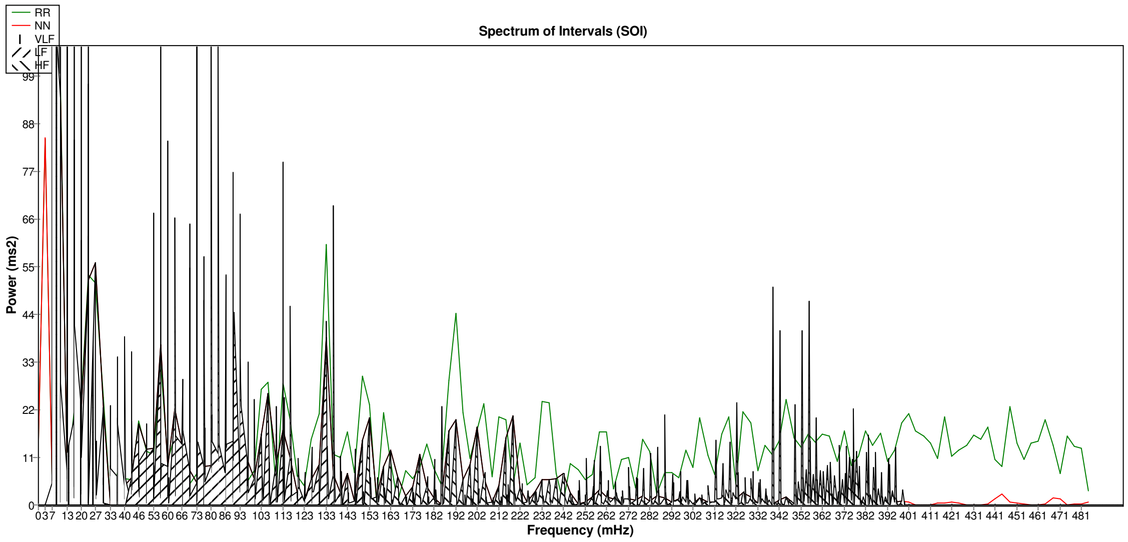

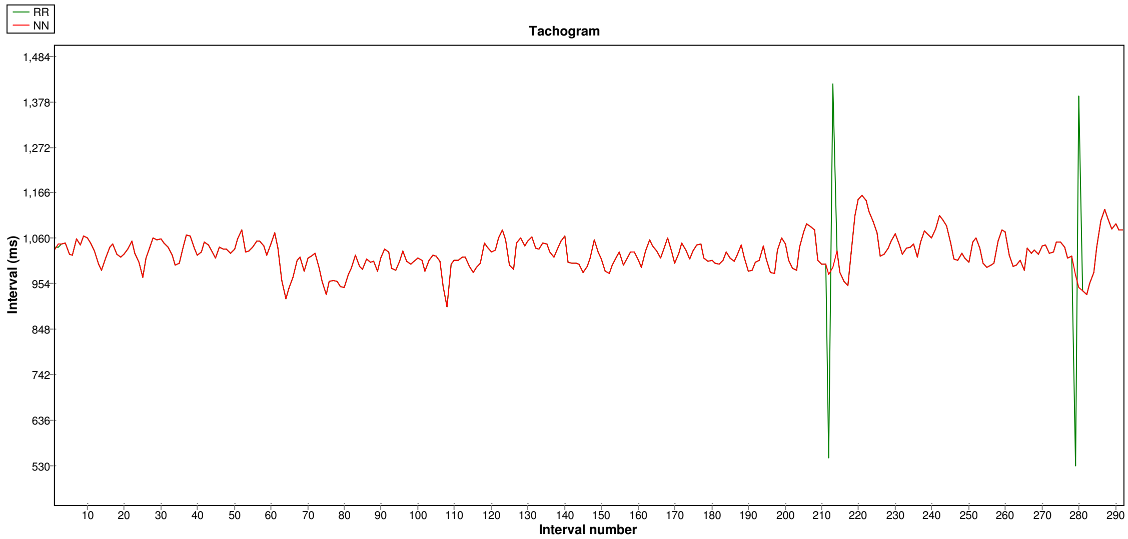

HRV parameters	NN	RR
TP (ms2)	955	1740
VLF (ms2)	290	297
LF (ms2)	391	471
HF (ms2)	273	972
LF/HF	1.43	0.48
LF normalized	58.83	32.63
HF normalized	41.17	67.37
VLF peak (mHz)	10	10
LF peak (mHz)	133	133
HF peak (mHz)	219	192

HRV spectral settings	
Spectrum of Intervals (SOI)	
Frequency resolution (mHz)	3
VLF lower boundary (mHz)	3
VLF upper boundary (mHz)	40
LF upper boundary (mHz)	150
HF upper boundary (mHz)	400
Smoothing factor	1
Tapering	Hann
Fourier transform	DFT
Sample frequency (Hz)	0.98
Interval correction	Annotation
Interval threshold (%)	10
