# Supplementary material for: The effects of a 3-day mountain bike cycling race on the autonomic nervous system (ANS) and heart rate variability in amateur cyclists: a prospective quantitative research design
Source: BMC Sports Sci Med Rehabil. 2023 Jan 2;15:2. doi: 10.1186/s13102-022-00614-y (PMC9808932; doi:10.1186/s13102-022-00614-y)
Supplement: Supplementary file 1 — Additional file 1. Individual data of Participants. [file 13102_2022_614_MOESM1_ESM.zip › Individual data of Participants/HRV Data/007/ECG_007_20180503160958_.PDF]

Anton Swart Biokinetic Rehabilitation Practice

Name:	007 007 007	Recorded:	03/05/2018 16:09:58	UNCONFIRMED INTERPRETATION - MD SHOULD REVIEW
Number:	007	Recorded by:	Mr. Anton Swart	
Gender:	Male	Referring physician:		
Birthdate:	25/12/1976 41 years	Ordering physician:		
		Attending physician:		
P / PQ:	118 ms / 165 ms	Location:	Anton Swart Biokinetic Rehabilitation Practi	
QRS:	100 ms	Comment:		
QT / QTc / QTd:	382 ms / 436 ms / -			
P/QRS/T axis:	77° / 84° / 73°			
Heartrate:	91 bpm			

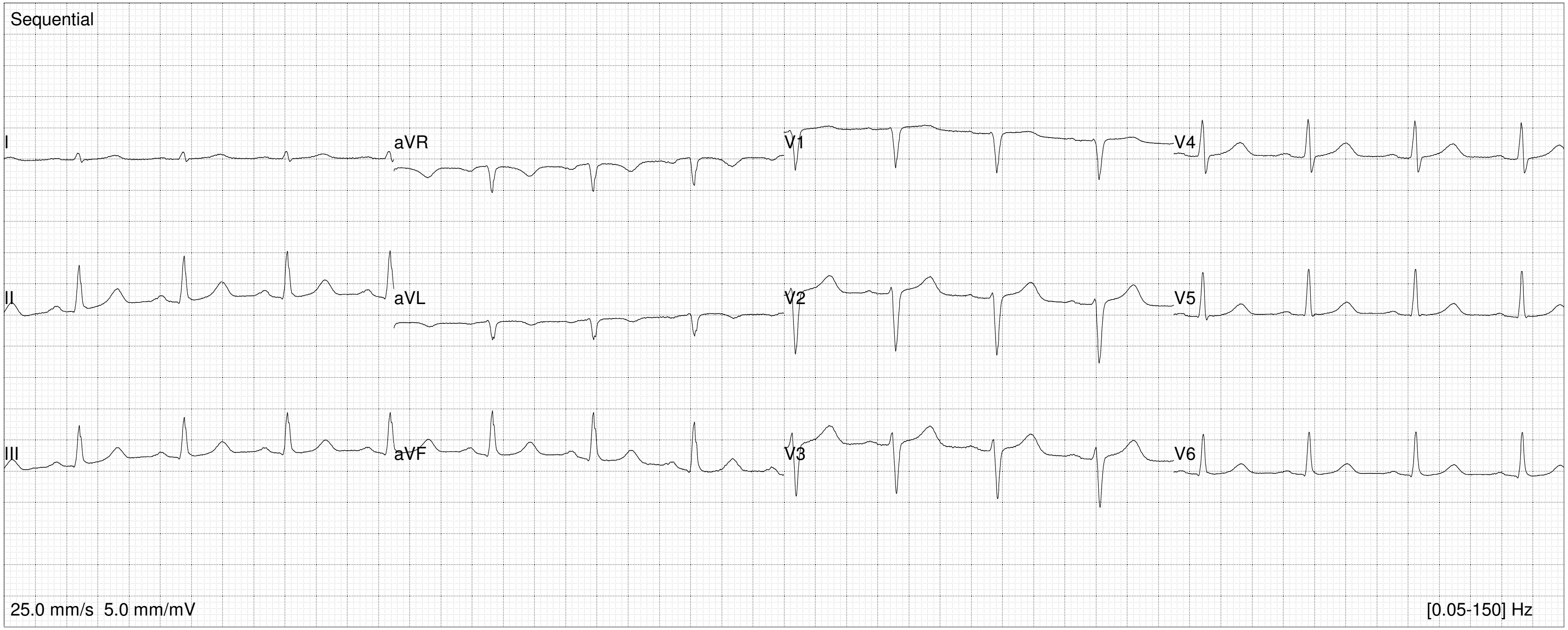

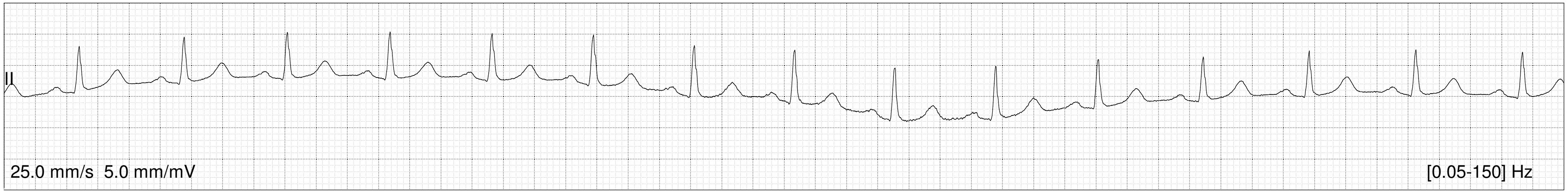

Anton Swart Biokinetic Rehabilitation Practice

Name:	007 007 007	Recorded:	03/05/2018 16:09:58	UNCONFIRMED INTERPRETATION - MD SHOULD REVIEW
Number:	007	Recorded by:	Mr. Anton Swart	
Gender:	Male	Referring physician:		
Birthdate:	25/12/1976 41 years	Location:	Anton Swart Biokinetic Rehabilitation Practice	
		Ordering physician:		
		Attending physician:		
		Comment:		

P / PQ:	118 ms / 165 ms
QRS:	100 ms
QT / QTc / QTd:	382 ms / 436 ms / -
P/QRS/T axis:	77° / 84° / 73°
Heartrate:	91 bpm

Beats

Total:	449
Normal:	449
Other:	0

RR

Minimum	620 ms
Maximum	730 ms
Mean:	665 ms
SD:	17 ms

R-R Trend

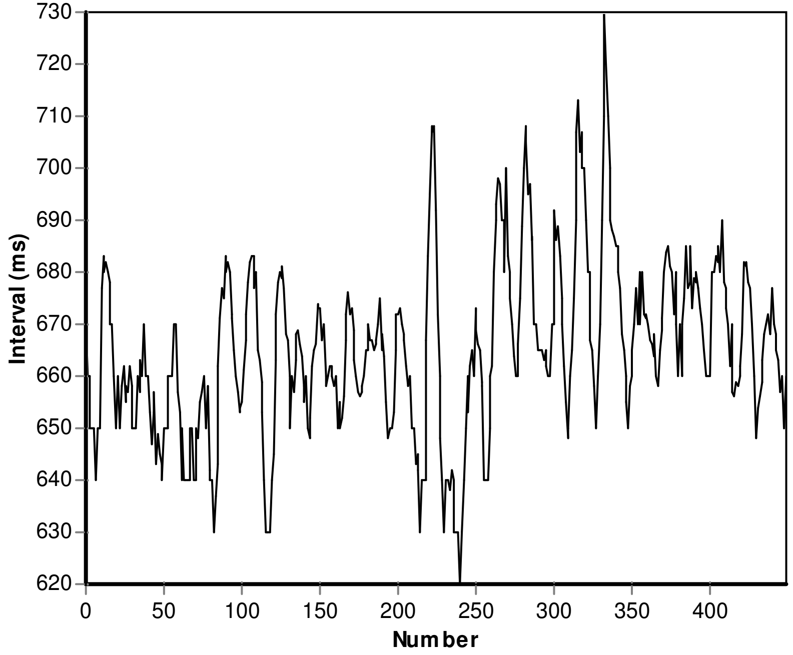

R-R Histogram

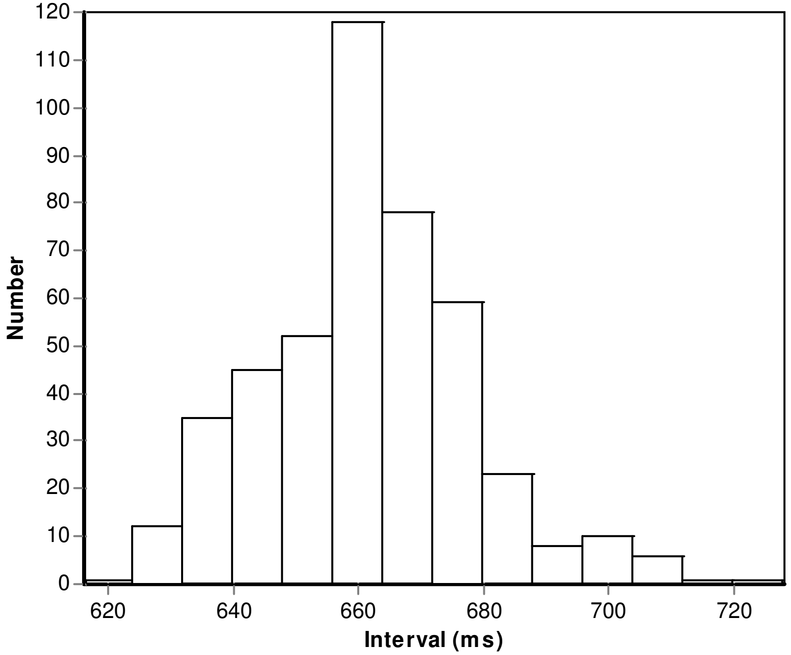

Heart Rate Variability: Time Domain Analysis

**Name:**	007, 007 007	**Birthdate:**	25/12/1976
**Number:**	007	**Recorded:**	03/05/2018 16:09:58
**Gender:**	Male		

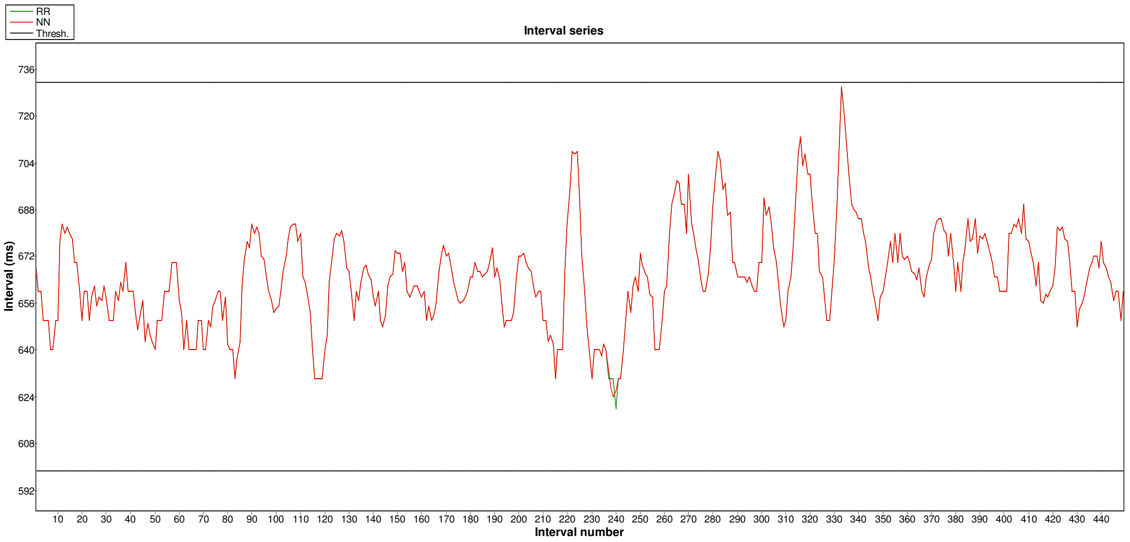

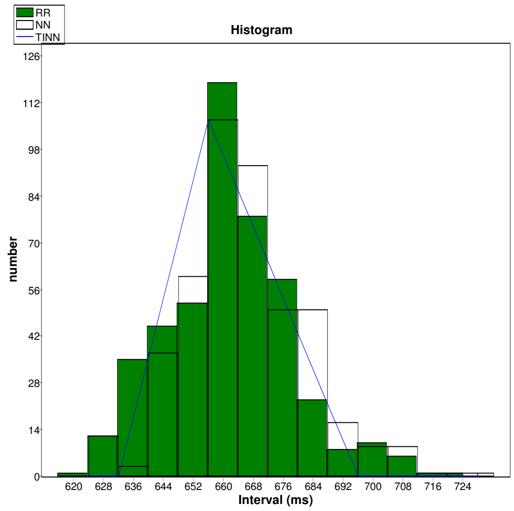

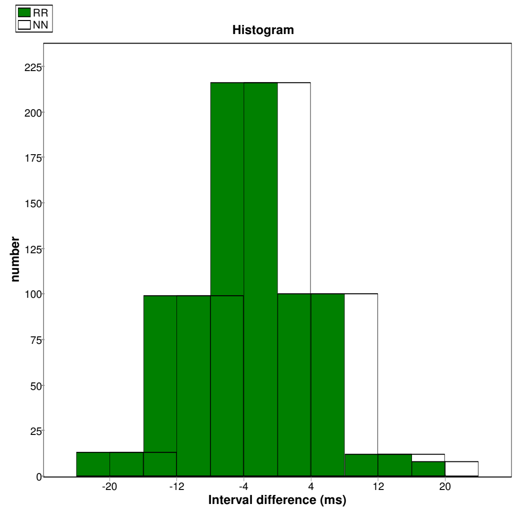

Binsize (ms) = 8

HRV parameters	NN	RR
SDNN (ms)	17	17
Triangular Interpolation (ms)	64	64
Triangular Index	4.20	3.81

HRV parameters	NN	RR
SDSD (ms)	7	7
RMSSD (ms)	7	7
NN50	0	0
NN50(1)	0	0
NN50(2)	0	0
pNN50	0.00	0.00
pNN50(1)	0.00	0.00
pNN50(2)	0.00	0.00
Logarithmic Index	1.71	1.71
SD(Logarithmic Index)	0.20	0.20

Interval statistics	NN	RR
Number	449	449
Minimum (ms)	624	620
Maximum (ms)	730	730
Range (ms)	106	110
Avg (ms)	665	665
SD (ms)	17	17
AvgDev (ms)	13	13
p5 (ms)	640	640
p50 (ms)	665	665
p95 (ms)	695	695
Skewness	0.40	0.40
Kurtosis	3.71	3.72

Interval statistics	NN	RR
Number	448	448
Minimum (ms)	-20	-20
Maximum (ms)	27	27
Range (ms)	47	47
Avg (ms)	-0	-0
SD (ms)	7	7
AvgDev (ms)	5	5
p5 (ms)	-10	-10
p50 (ms)	0	0
p95 (ms)	11	11
Skewness	0.43	0.42
Kurtosis	3.76	3.72

Cardio Control NV. User: Anton

Heart Rate Variability: Frequency Domain Analysis

**Name:**	007, 007 007	**Birthdate:**	25/12/1976
**Number:**	007	**Recorded:**	03/05/2018 16:09:58
**Gender:**	Male		

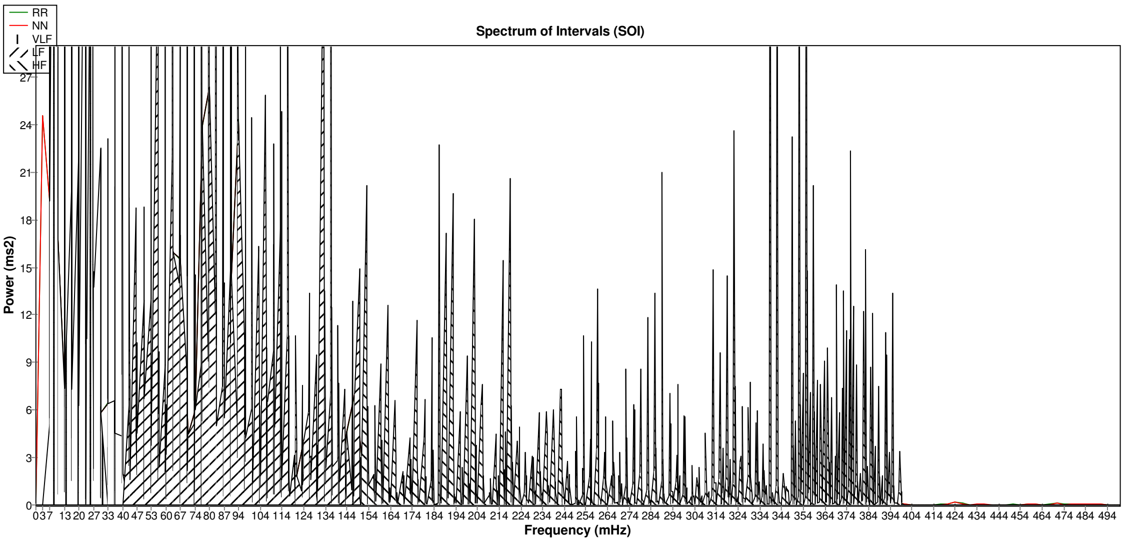

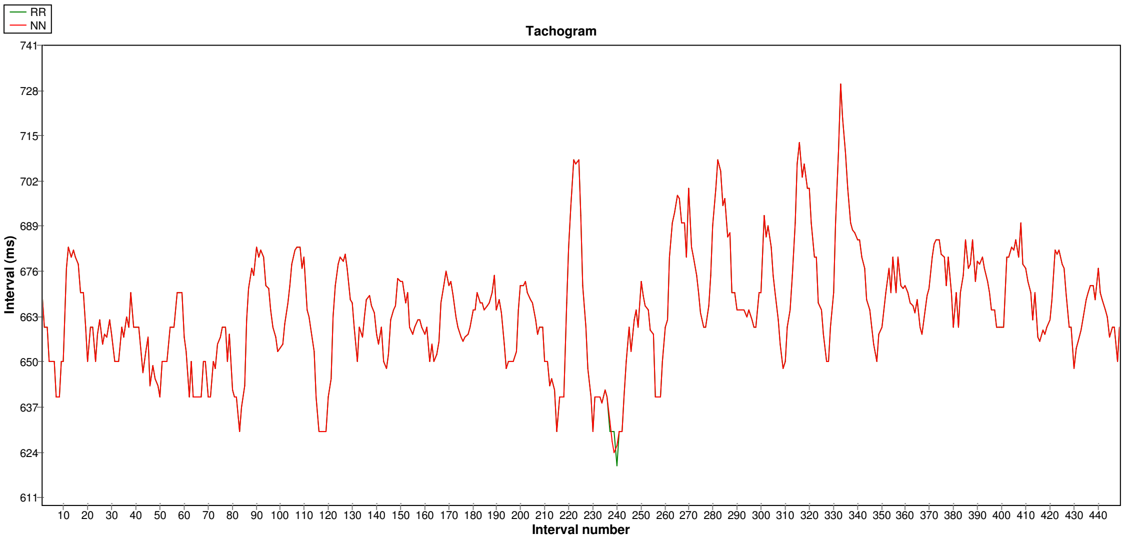

**HRV parameters**	**NN**	**RR**
TP (ms2)	322	322
VLF (ms2)	100	100
LF (ms2)	211	211
HF (ms2)	11	11
LF/HF	18.90	19.20
LF normalized	94.97	95.05
HF normalized	5.03	4.95
VLF peak (mHz)	7	7
LF peak (mHz)	80	80
HF peak (mHz)	150	150

**HRV spectral settings**	
Spectrum of Intervals (SOI)	
Frequency resolution (mHz)	3
VLF lower boundary (mHz)	3
VLF upper boundary (mHz)	40
LF upper boundary (mHz)	150
HF upper boundary (mHz)	400
Smoothing factor	1
Tapering	Hann
Fourier transform	DFT
Sample frequency (Hz)	1.50
Interval correction	Annotation
Interval threshold (%)	10
