# Supplementary material for: The effects of a 3-day mountain bike cycling race on the autonomic nervous system (ANS) and heart rate variability in amateur cyclists: a prospective quantitative research design
Source: BMC Sports Sci Med Rehabil. 2023 Jan 2;15:2. doi: 10.1186/s13102-022-00614-y (PMC9808932; doi:10.1186/s13102-022-00614-y)
Supplement: Supplementary file 1 — Additional file 1. Individual data of Participants. [file 13102_2022_614_MOESM1_ESM.zip › Individual data of Participants/HRV Data/007/ECG_007_20180504121649_.PDF]

Anton Swart Biokinetic Rehabilitation Practice

Name:	007 007 007	Recorded:	04/05/2018 12:16:49	UNCONFIRMED INTERPRETATION - MD SHOULD REVIEW
Number:	007	Recorded by:	Mr. Anton Swart	
Gender:	Male	Referring physician:		
Birthdate:	25/12/1976 41 years	Ordering physician:		
		Attending physician:		
P / PQ:	113 ms / 158 ms	Location:	Anton Swart Biokinetic Rehabilitation Practi	
QRS:	109 ms	Comment:		
QT / QTc / QTd:	389 ms / 433 ms / -			
P/QRS/T axis:	74° / 79° / 68°			
Heartrate:	85 bpm			

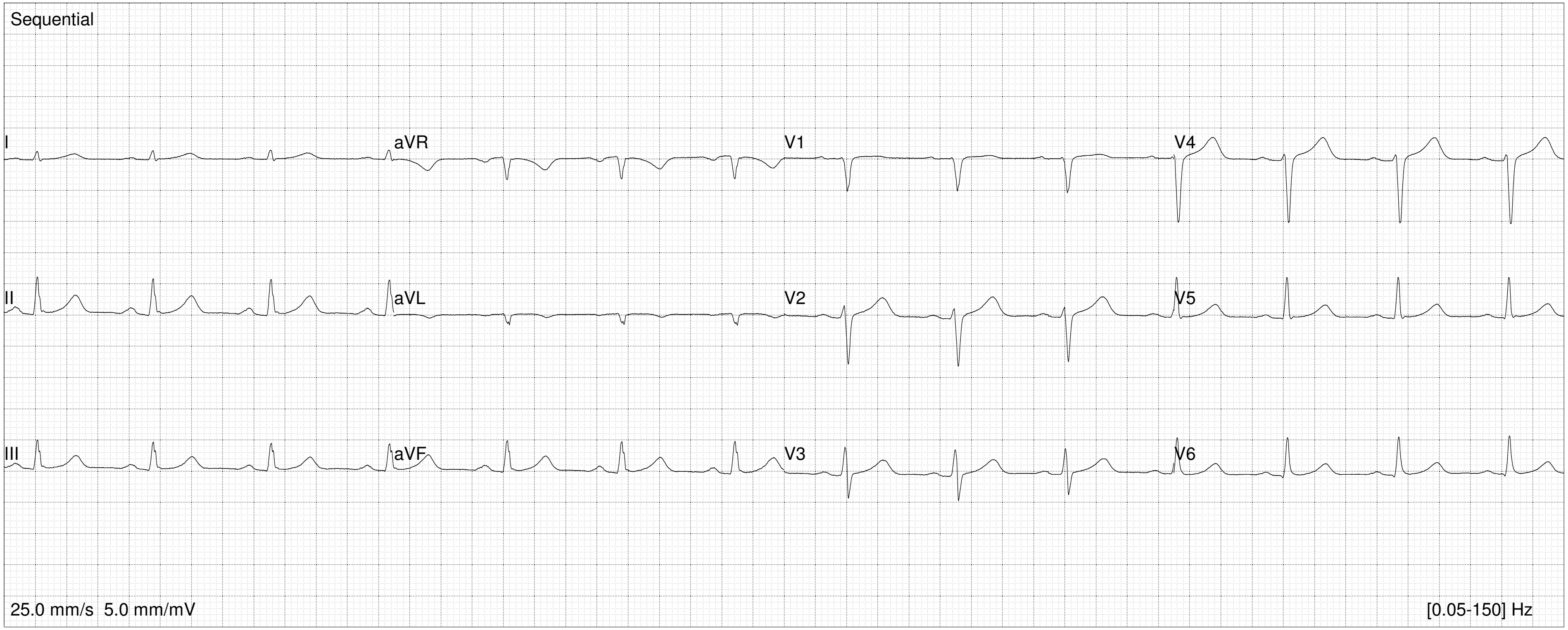

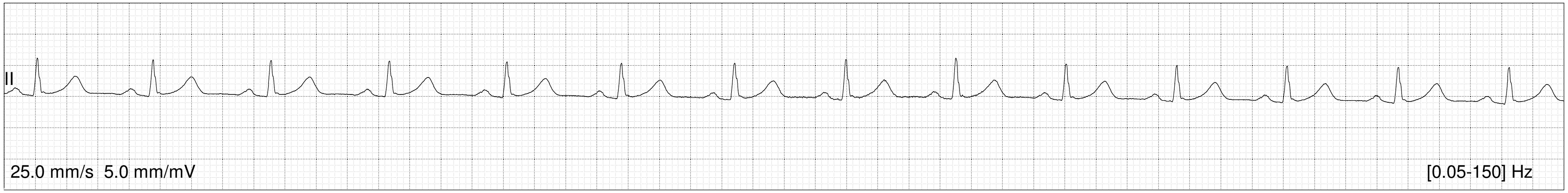

Anton Swart Biokinetic Rehabilitation Practice

Name:	007 007 007	Recorded:	04/05/2018 12:16:49	UNCONFIRMED INTERPRETATION - MD SHOULD REVIEW
Number:	007	Recorded by:	Mr. Anton Swart	
Gender:	Male	Referring physician:		
Birthdate:	25/12/1976 41 years	Location:	Anton Swart Biokinetic Rehabilitation Practice	
P / PQ:	113 ms / 158 ms	Ordering physician:		
QRS:	109 ms	Attending physician:		
QT / QTc / QTd:	389 ms / 433 ms / -	Comment:		
P/QRS/T axis:	74° / 79° / 68°			
Heartrate:	85 bpm			

Beats

Total:	419
Normal:	419
Other:	0

RR

Minimum	450 ms
Maximum	930 ms
Mean:	714 ms
SD:	39 ms

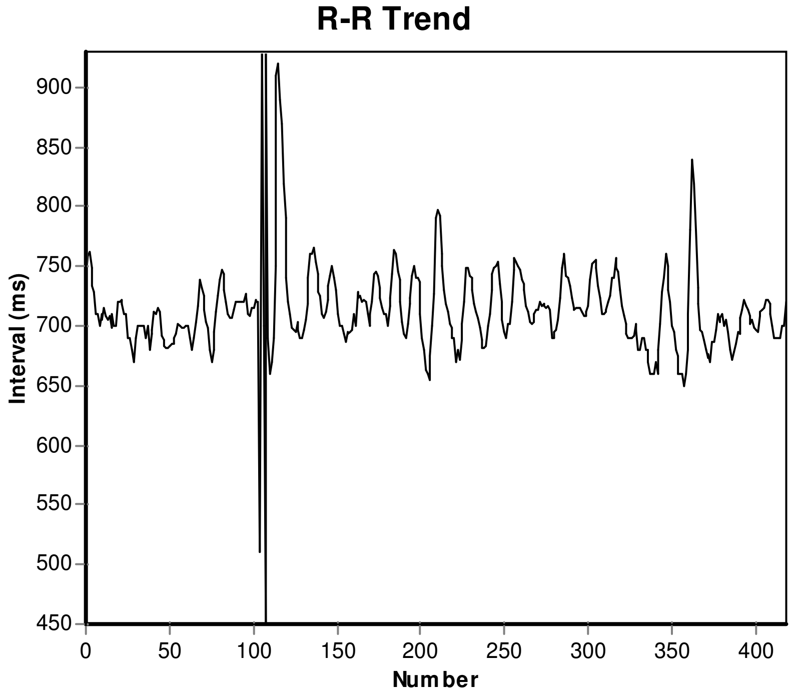

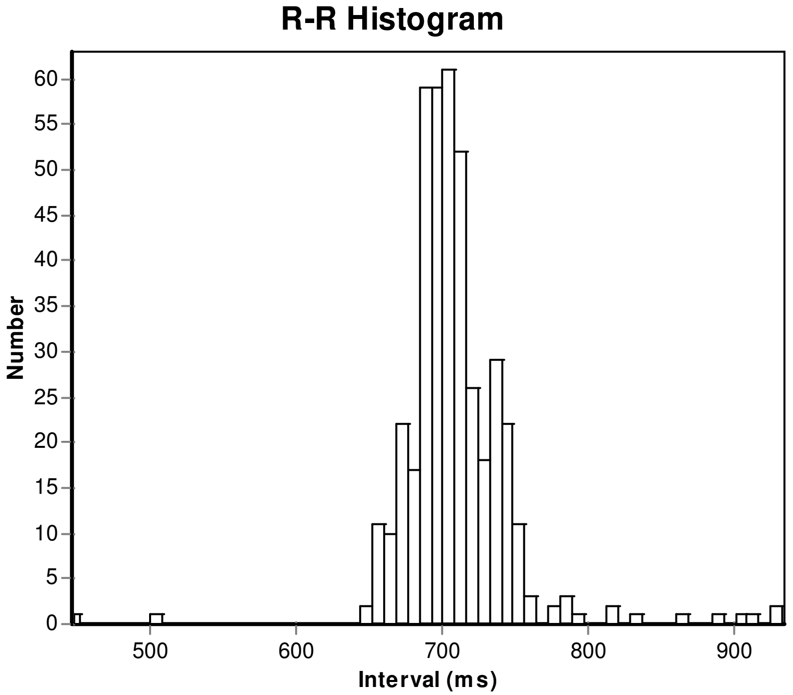

Cardio Control NV. User: Anton

Heart Rate Variability: Time Domain Analysis

**Name:**	007, 007 007	**Birthdate:**	25/12/1976
**Number:**	007	**Recorded:**	04/05/2018 12:16:49
**Gender:**	Male		

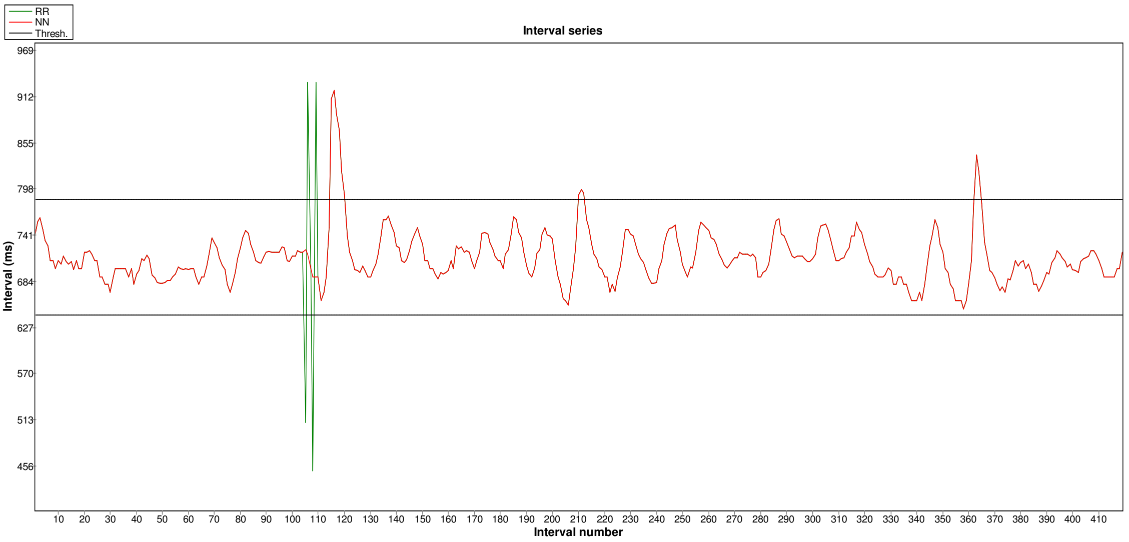

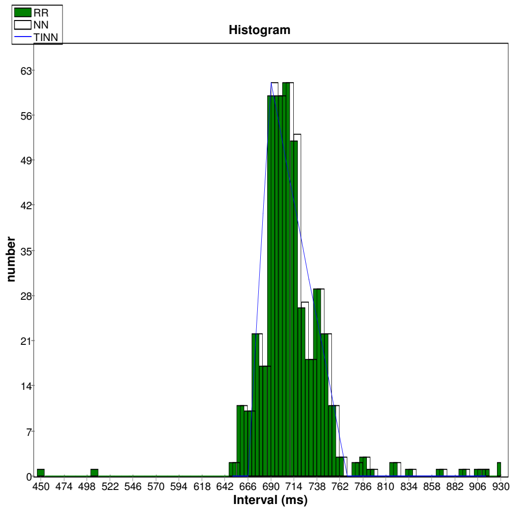

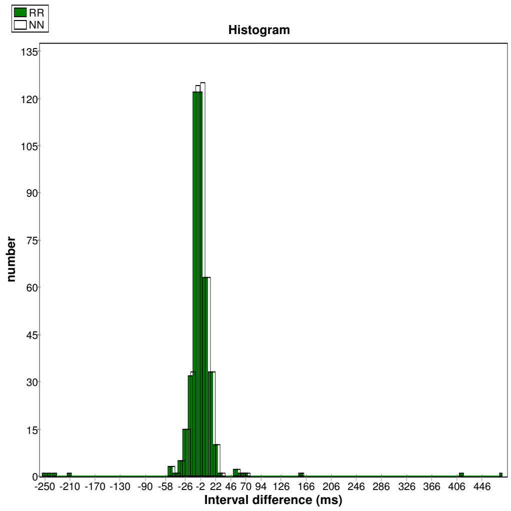

Binsize (ms) = 8

**HRV parameters**	**NN**	**RR**
SDNN (ms)	32	39
Triangular Interpolation (ms)	104	112
Triangular Index	6.87	6.87

**HRV parameters**	**NN**	**RR**
SDSD (ms)	15	42
RMSSD (ms)	15	42
NN50	5	11
NN50(1)	0	4
NN50(2)	5	7
pNN50	0.01	0.03
pNN50(1)	0.00	0.01
pNN50(2)	0.01	0.02
Logarithmic Index	0.61	0.31
SD(Logarithmic Index)	0.13	0.07

**Interval statistics**	**NN**	**RR**
Number	419	419
Minimum (ms)	650	450
Maximum (ms)	920	930
Range (ms)	270	480
Avg (ms)	714	714
SD (ms)	32	39
AvgDev (ms)	22	24
p5 (ms)	672	670
p50 (ms)	710	710
p95 (ms)	760	760
Skewness	2.20	0.98
Kurtosis	13.02	17.26

**Interval statistics**	**NN**	**RR**
Number	418	418
Minimum (ms)	-50	-250
Maximum (ms)	160	480
Range (ms)	210	730
Avg (ms)	-0	-0
SD (ms)	15	42
AvgDev (ms)	10	14
p5 (ms)	-20	-20
p50 (ms)	0	0
p95 (ms)	20	21
Skewness	3.07	4.61
Kurtosis	31.84	77.44

Heart Rate Variability: Frequency Domain Analysis

**Name:**	007, 007 007	**Birthdate:**	25/12/1976
**Number:**	007	**Recorded:**	04/05/2018 12:16:49
**Gender:**	Male		

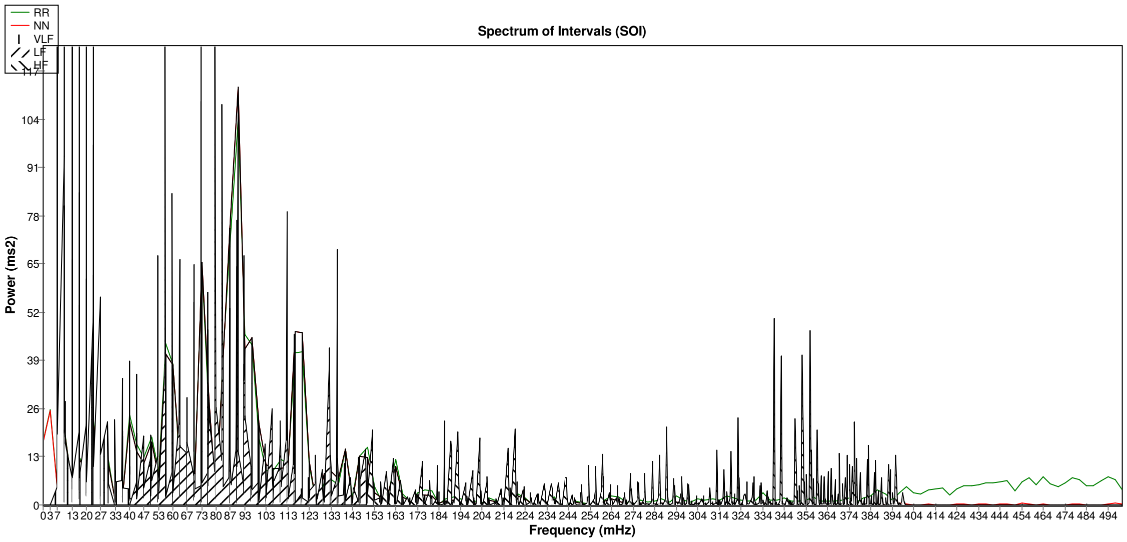

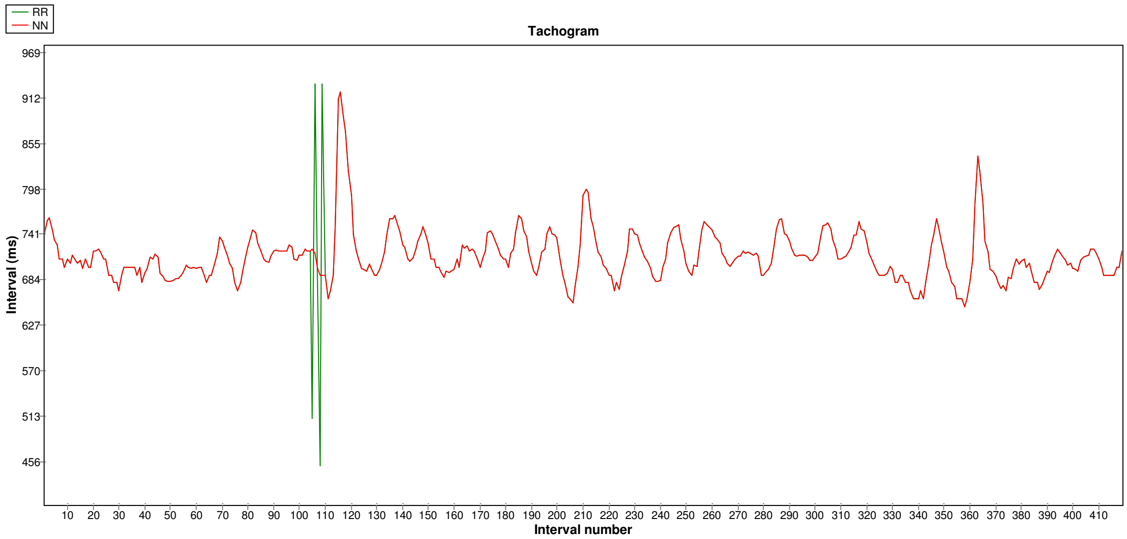

HRV parameters	NN	RR
TP (ms2)	967	1016
VLF (ms2)	107	113
LF (ms2)	783	757
HF (ms2)	76	146
LF/HF	10.34	5.18
LF normalized	91.18	83.83
HF normalized	8.82	16.17
VLF peak (mHz)	40	40
LF peak (mHz)	90	90
HF peak (mHz)	150	150

HRV spectral settings	
Spectrum of Intervals (SOI)	
Frequency resolution (mHz)	3
VLF lower boundary (mHz)	3
VLF upper boundary (mHz)	40
LF upper boundary (mHz)	150
HF upper boundary (mHz)	400
Smoothing factor	1
Tapering	Hann
Fourier transform	DFT
Sample frequency (Hz)	1.40
Interval correction	Annotation
Interval threshold (%)	10
