# Supplementary material for: The effects of a 3-day mountain bike cycling race on the autonomic nervous system (ANS) and heart rate variability in amateur cyclists: a prospective quantitative research design
Source: BMC Sports Sci Med Rehabil. 2023 Jan 2;15:2. doi: 10.1186/s13102-022-00614-y (PMC9808932; doi:10.1186/s13102-022-00614-y)
Supplement: Supplementary file 1 — Additional file 1. Individual data of Participants. [file 13102_2022_614_MOESM1_ESM.zip › Individual data of Participants/HRV Data/007/ECG_007_20180505115714_.PDF]

Anton Swart Biokinetic Rehabilitation Practice

Name: 007 007 007
Number: 007
Gender: Male
Birthdate: 25/12/1976 41 years

P / PQ: 110 ms / 148 ms
QRS: 100 ms
QT / QTc / QTd: 394 ms / 441 ms / -
P/QRS/T axis: 78° / 82° / 72°
Heartrate: 87 bpm

Recorded: 05/05/2018 11:57:14
Recorded by: Mr. Anton Swart
Referring physician:
Ordering physician:
Attending physician:
Location: Anton Swart Biokinetic Rehabilitation Practi
Comment:

UNCONFIRMED INTERPRETATION - MD SHOULD REVIEW

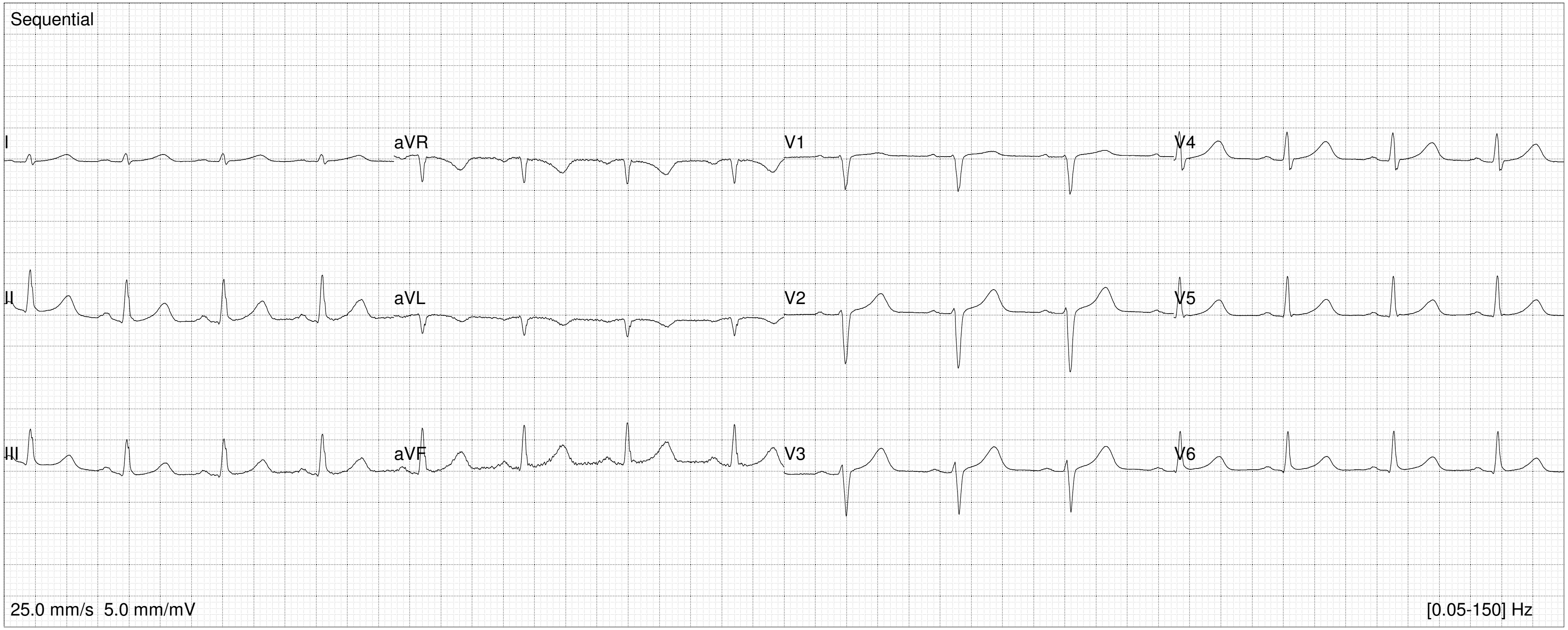

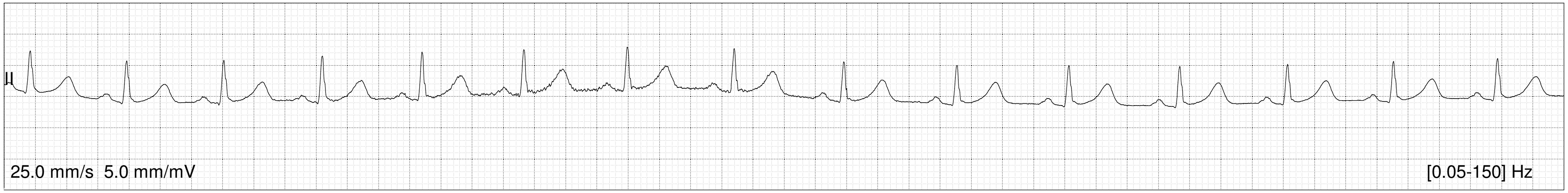

Anton Swart Biokinetic Rehabilitation Practice

Name:	007 007 007	Recorded:	05/05/2018 11:57:14	UNCONFIRMED INTERPRETATION - MD SHOULD REVIEW
Number:	007	Recorded by:	Mr. Anton Swart	
Gender:	Male	Referring physician:		
Birthdate:	25/12/1976 41 years	Location:	Anton Swart Biokinetic Rehabilitation Practice	
		Ordering physician:		
		Attending physician:		
		Comment:		

P / PQ:	110 ms / 148 ms
QRS:	100 ms
QT / QTc / QTd:	394 ms / 441 ms / -
P/QRS/T axis:	78° / 82° / 72°
Heartrate:	87 bpm

**Beats**		**RR**	
Total:	427	Minimum	460 ms
Normal:	427	Maximum	930 ms
Other:	0	Mean:	700 ms
		SD:	43 ms

R-R Trend

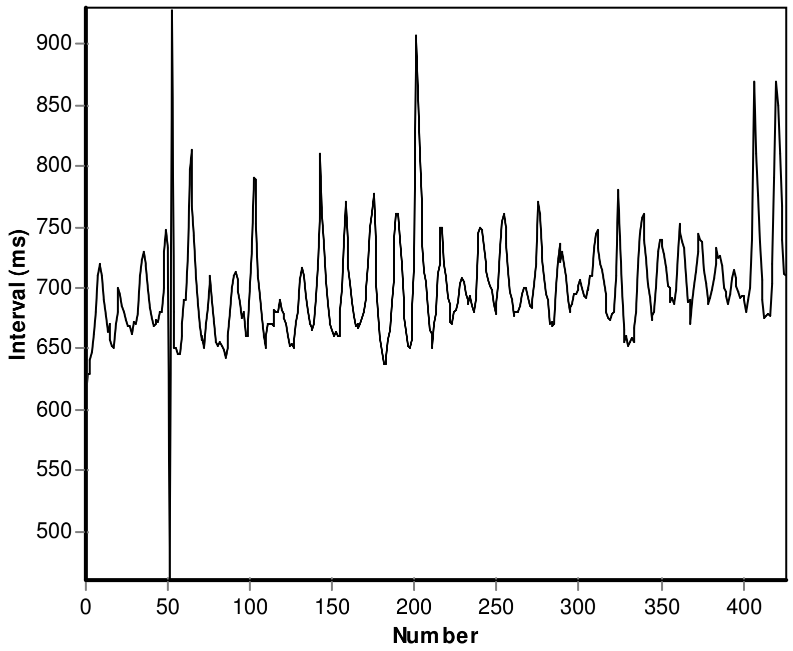

R-R Histogram

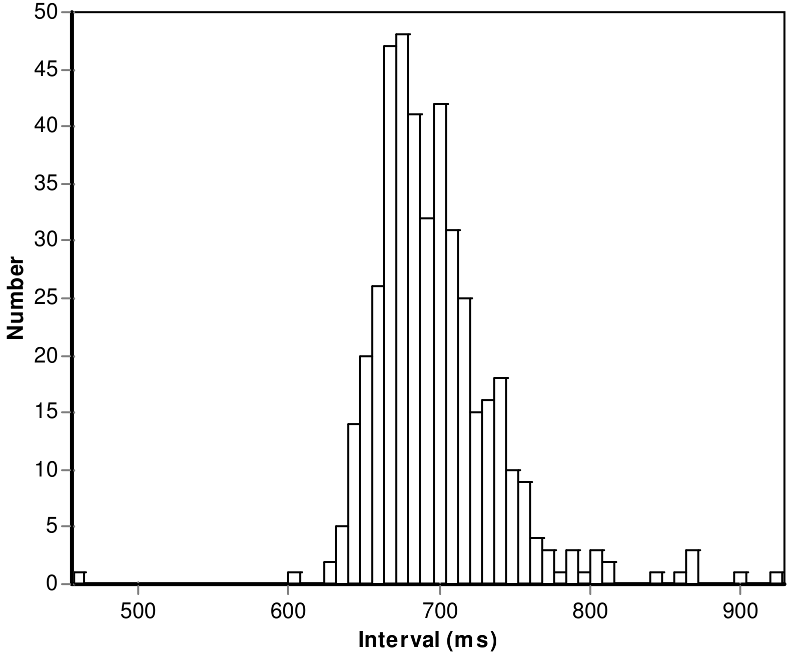

Heart Rate Variability: Time Domain Analysis

**Name:**	007, 007 007	**Birthdate:**	25/12/1976
**Number:**	007	**Recorded:**	05/05/2018 11:57:14
**Gender:**	Male		

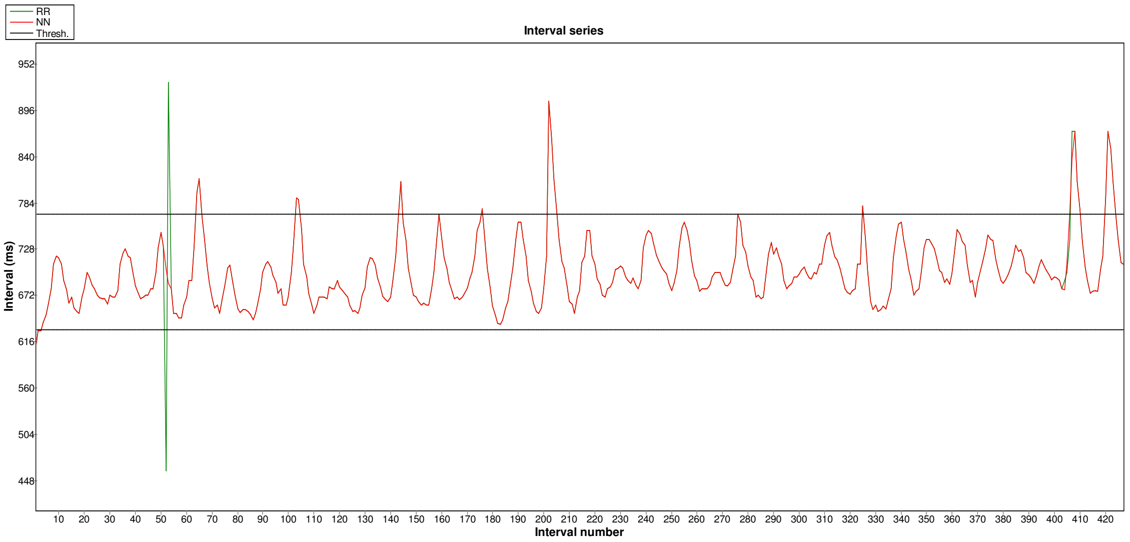

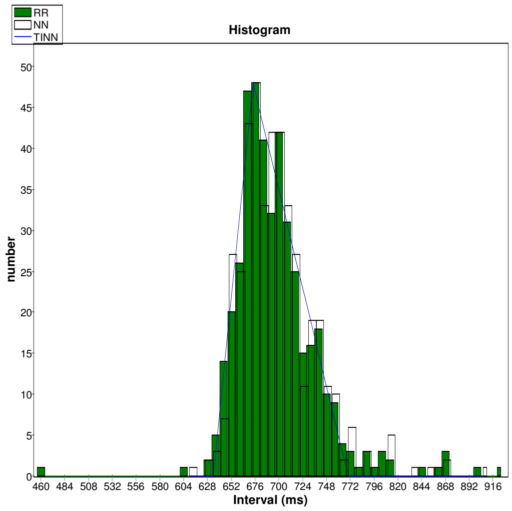

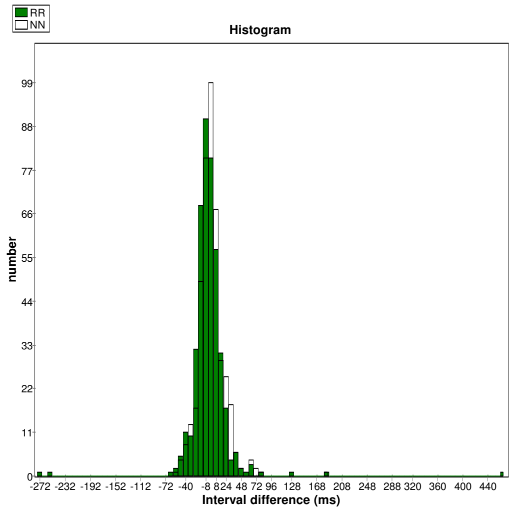

Binsize (ms) = 8

**HRV parameters**	**NN**	**RR**
SDNN (ms)	39	43
Triangular Interpolation (ms)	136	136
Triangular Index	8.90	8.90

**HRV parameters**	**NN**	**RR**
SDSD (ms)	21	36
RMSSD (ms)	21	36
NN50	9	11
NN50(1)	1	3
NN50(2)	8	8
pNN50	0.02	0.03
pNN50(1)	0.00	0.01
pNN50(2)	0.02	0.02
Logarithmic Index	0.54	0.40
SD(Logarithmic Index)	0.03	0.04

**Interval statistics**	**NN**	**RR**
Number	427	427
Minimum (ms)	610	460
Maximum (ms)	907	930
Range (ms)	297	470
Avg (ms)	700	700
SD (ms)	39	43
AvgDev (ms)	29	30
p5 (ms)	651	650
p50 (ms)	693	693
p95 (ms)	770	770
Skewness	1.47	1.16
Kurtosis	6.86	9.61

**Interval statistics**	**NN**	**RR**
Number	426	426
Minimum (ms)	-60	-272
Maximum (ms)	187	470
Range (ms)	247	742
Avg (ms)	0	0
SD (ms)	21	36
AvgDev (ms)	14	16
p5 (ms)	-31	-33
p50 (ms)	-2	-2
p95 (ms)	31	30
Skewness	2.01	3.88
Kurtosis	17.77	82.94

Heart Rate Variability: Frequency Domain Analysis

**Name:**	007, 007 007	**Birthdate:**	25/12/1976
**Number:**	007	**Recorded:**	05/05/2018 11:57:14
**Gender:**	Male		

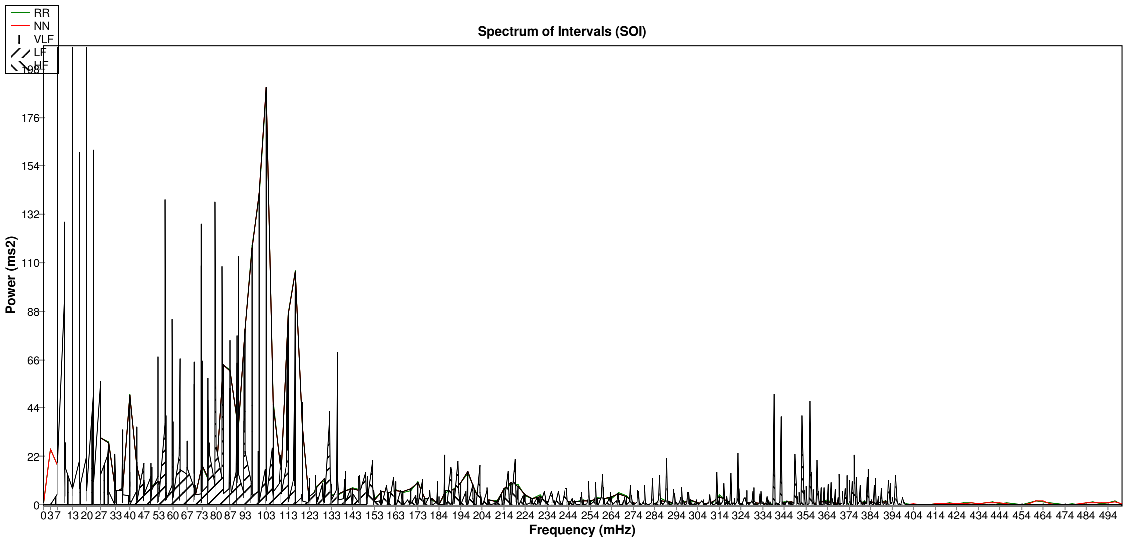

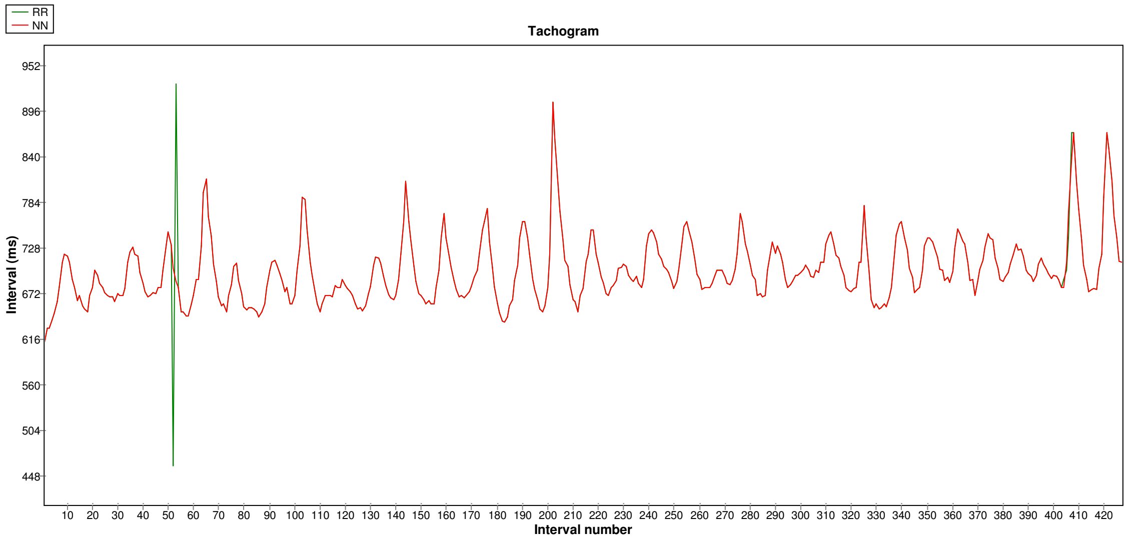

**HRV parameters**	**NN**	**RR**
TP (ms2)	1468	1471
VLF (ms2)	151	152
LF (ms2)	1121	1120
HF (ms2)	197	199
LF/HF	5.70	5.62
LF normalized	85.07	84.89
HF normalized	14.93	15.11
VLF peak (mHz)	40	40
LF peak (mHz)	103	103
HF peak (mHz)	197	197

**HRV spectral settings**	
Spectrum of Intervals (SOI)	
Frequency resolution (mHz)	3
VLF lower boundary (mHz)	3
VLF upper boundary (mHz)	40
LF upper boundary (mHz)	150
HF upper boundary (mHz)	400
Smoothing factor	1
Tapering	Hann
Fourier transform	DFT
Sample frequency (Hz)	1.43
Interval correction	Annotation
Interval threshold (%)	10
