# Supplementary material for: The effects of a 3-day mountain bike cycling race on the autonomic nervous system (ANS) and heart rate variability in amateur cyclists: a prospective quantitative research design
Source: BMC Sports Sci Med Rehabil. 2023 Jan 2;15:2. doi: 10.1186/s13102-022-00614-y (PMC9808932; doi:10.1186/s13102-022-00614-y)
Supplement: Supplementary file 1 — Additional file 1. Individual data of Participants. [file 13102_2022_614_MOESM1_ESM.zip › Individual data of Participants/HRV Data/007/ECG_007_20180506072750_.PDF]

Anton Swart Biokinetic Rehabilitation Practice

Name:	007 007 007	Recorded:	06/05/2018 07:27:50	UNCONFIRMED INTERPRETATION - MD SHOULD REVIEW
Number:	007	Recorded by:	Mr. Anton Swart	
Gender:	Male	Referring physician:		
Birthdate:	25/12/1976 41 years	Ordering physician:		
		Attending physician:		
P / PQ:	107 ms / 160 ms	Location:	Anton Swart Biokinetic Rehabilitation Practi	
QRS:	100 ms	Comment:		
QT / QTc / QTd:	488 ms / 469 ms / -			
P/QRS/T axis:	78° / 85° / 76°			
Heartrate:	49 bpm			

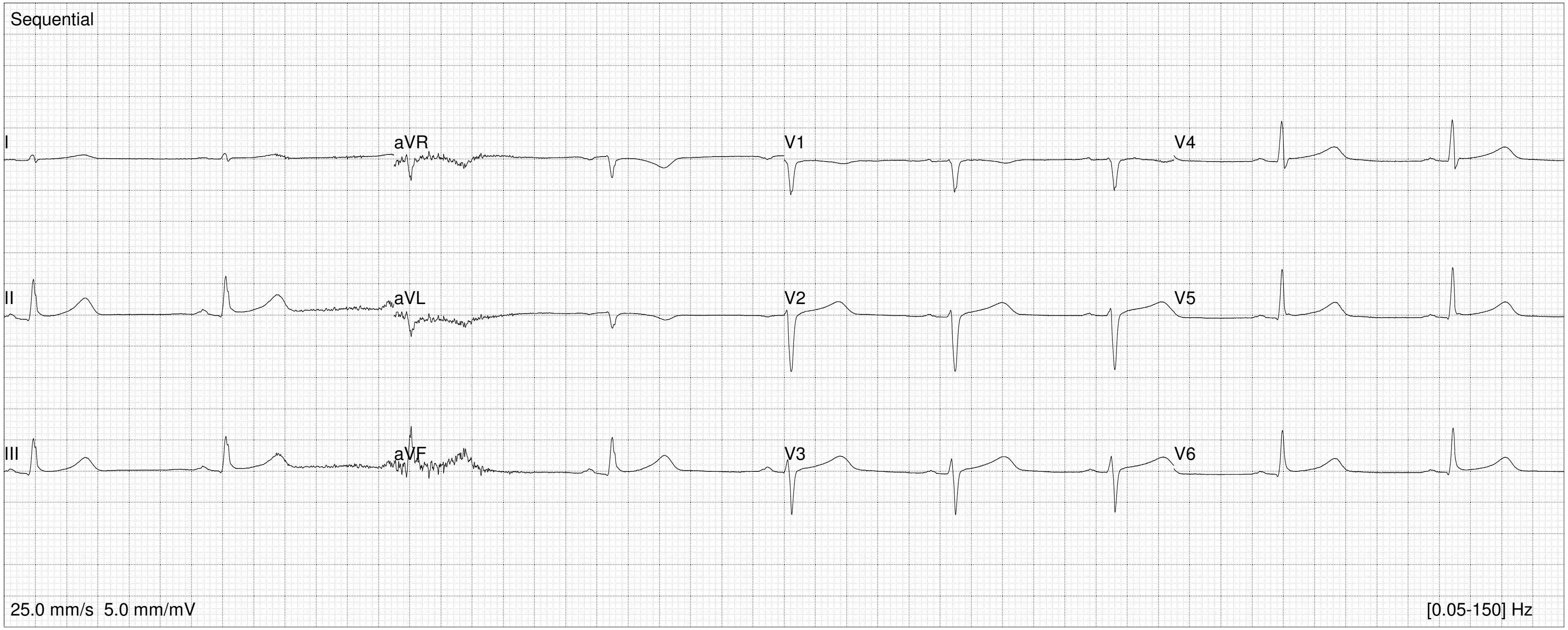

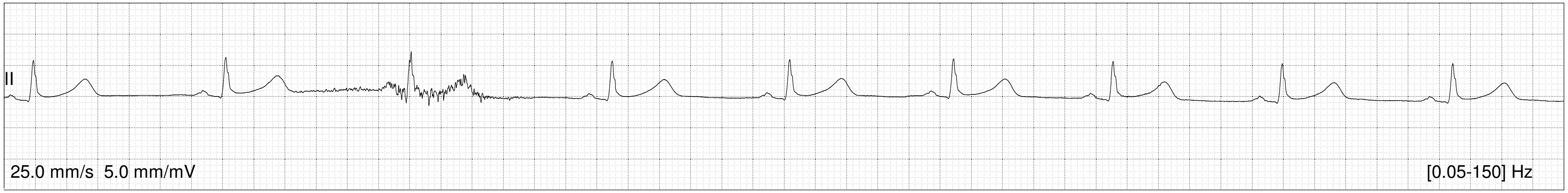

Anton Swart Biokinetic Rehabilitation Practice

Name:	007 007 007	Recorded:	06/05/2018 07:27:50
Number:	007	Recorded by:	Mr. Anton Swart
Gender:	Male	Referring physician:	
Birthdate:	25/12/1976 41 years	Location:	Anton Swart Biokinetic Rehabilitation Practice
		Ordering physician:	
		Attending physician:	
		Comment:	

UNCONFIRMED INTERPRETATION - MD SHOULD REVIEW

P / PQ:	107 ms / 160 ms
QRS:	100 ms
QT / QTc / QTd:	488 ms / 469 ms / -
P/QRS/T axis:	78° / 85° / 76°
Heartrate:	49 bpm

Beats

Total:	247
Normal:	247
Other:	0

RR

Minimum	550 ms
Maximum	1680 ms
Mean:	1213 ms
SD:	102 ms

R-R Trend

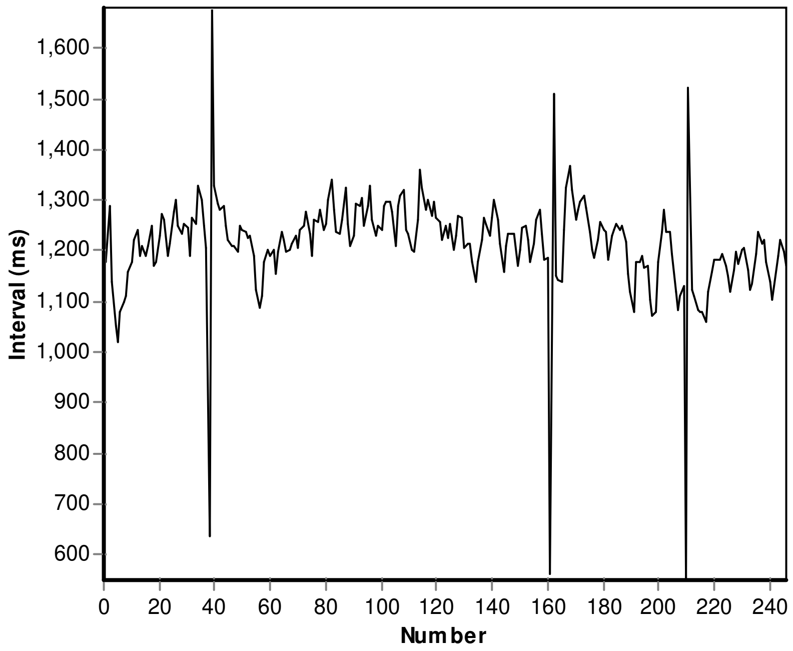

R-R Histogram

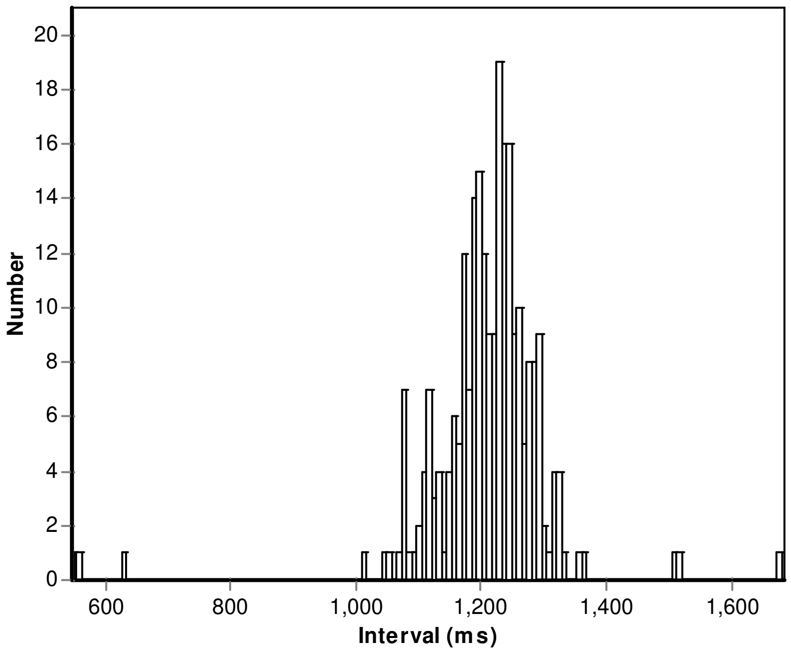

Heart Rate Variability: Time Domain Analysis

**Name:**	007, 007 007	**Birthdate:**	25/12/1976
**Number:**	007	**Recorded:**	06/05/2018 07:27:50
**Gender:**	Male		

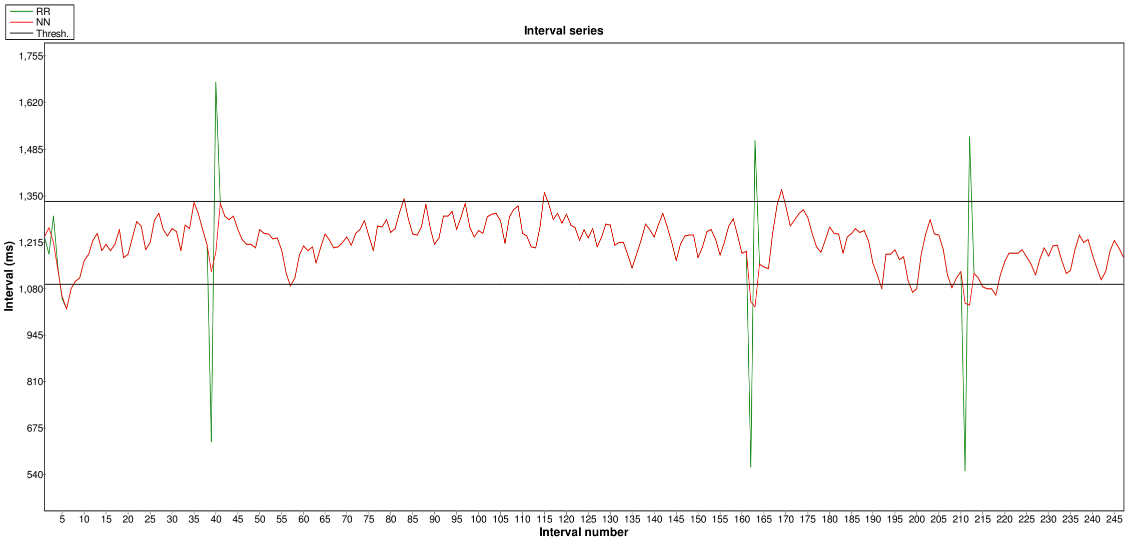

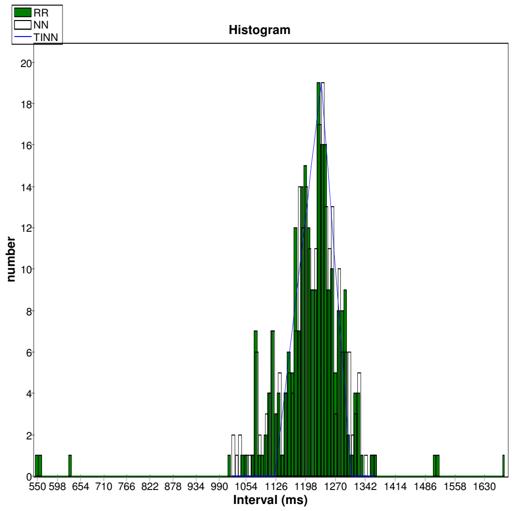

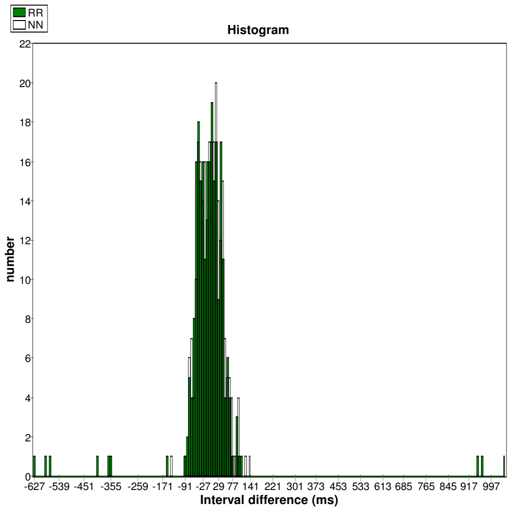

Binsize (ms) = 8

**HRV parameters**	**NN**	**RR**
SDNN (ms)	66	102
Triangular Interpolation (ms)	184	184
Triangular Index	13.00	13.00

**HRV parameters**	**NN**	**RR**
SDSD (ms)	43	140
RMSSD (ms)	43	140
NN50	48	51
NN50(1)	24	27
NN50(2)	24	24
pNN50	0.19	0.21
pNN50(1)	0.10	0.11
pNN50(2)	0.10	0.10
Logarithmic Index	0.27	0.19
SD(Logarithmic Index)	0.02	0.02

**Interval statistics**	**NN**	**RR**
Number	247	247
Minimum (ms)	1020	550
Maximum (ms)	1367	1680
Range (ms)	347	1130
Avg (ms)	1213	1213
SD (ms)	66	102
AvgDev (ms)	51	60
p5 (ms)	1080	1083
p50 (ms)	1221	1222
p95 (ms)	1310	1323
Skewness	-0.56	-2.40
Kurtosis	3.23	21.38

**Interval statistics**	**NN**	**RR**
Number	246	246
Minimum (ms)	-144	-627
Maximum (ms)	143	1045
Range (ms)	287	1672
Avg (ms)	-0	-0
SD (ms)	43	140
AvgDev (ms)	35	56
p5 (ms)	-68	-72
p50 (ms)	0	-1
p95 (ms)	70	74
Skewness	0.19	3.18
Kurtosis	3.23	35.51

Cardio Control NV. User: Anton

Heart Rate Variability: Frequency Domain Analysis

**Name:**	007, 007 007	**Birthdate:**	25/12/1976
**Number:**	007	**Recorded:**	06/05/2018 07:27:50
**Gender:**	Male		

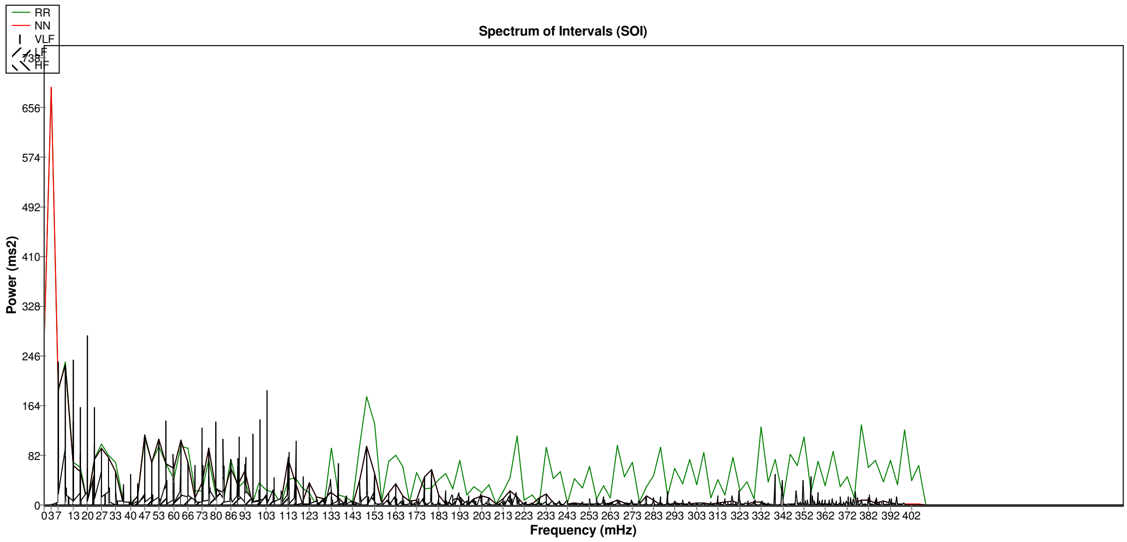

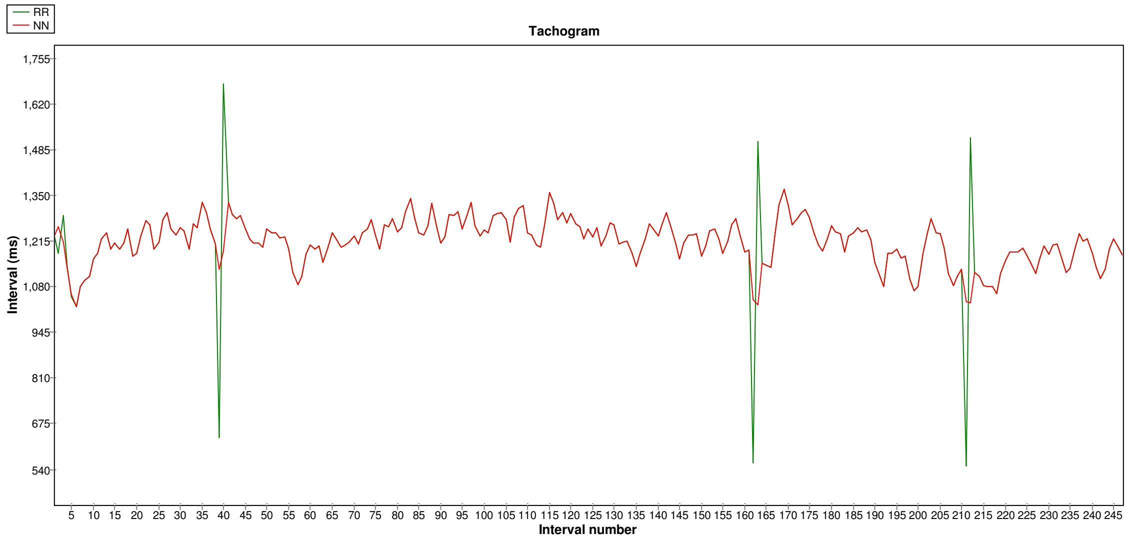

**HRV parameters**	**NN**	**RR**
TP (ms2)	2744	6062
VLF (ms2)	851	898
LF (ms2)	1339	1519
HF (ms2)	554	3645
LF/HF	2.42	0.42
LF normalized	70.73	29.42
HF normalized	29.27	70.58
VLF peak (mHz)	10	10
LF peak (mHz)	47	150
HF peak (mHz)	180	153

**HRV spectral settings**	
Spectrum of Intervals (SOI)	
Frequency resolution (mHz)	3
VLF lower boundary (mHz)	3
VLF upper boundary (mHz)	40
LF upper boundary (mHz)	150
HF upper boundary (mHz)	400
Smoothing factor	1
Tapering	Hann
Fourier transform	DFT
Sample frequency (Hz)	0.82
Interval correction	Annotation
Interval threshold (%)	10
