# Supplementary material for: The effects of a 3-day mountain bike cycling race on the autonomic nervous system (ANS) and heart rate variability in amateur cyclists: a prospective quantitative research design
Source: BMC Sports Sci Med Rehabil. 2023 Jan 2;15:2. doi: 10.1186/s13102-022-00614-y (PMC9808932; doi:10.1186/s13102-022-00614-y)
Supplement: Supplementary file 1 — Additional file 1. Individual data of Participants. [file 13102_2022_614_MOESM1_ESM.zip › Individual data of Participants/HRV Data/008/ECG_008_20180501100104_.PDF]

Anton Swart Biokinetic Rehabilitation Practice

Name:	008 008 008	Recorded:	01/05/2018 10:01:04	UNCONFIRMED INTERPRETATION - MD SHOULD REVIEW
Number:	008	Recorded by:	Mr. Anton Swart	
Gender:	Male	Referring physician:		
Birthdate:	13/12/1957 60 years	Ordering physician:		
		Attending physician:		
P / PQ:	115 ms / 168 ms	Location:	Anton Swart Biokinetic Rehabilitation Practi	
QRS:	89 ms	Comment:		
QT / QTc / QTd:	439 ms / 436 ms / -			
P/QRS/T axis:	75° / 68° / 70°			
Heartrate:	58 bpm			

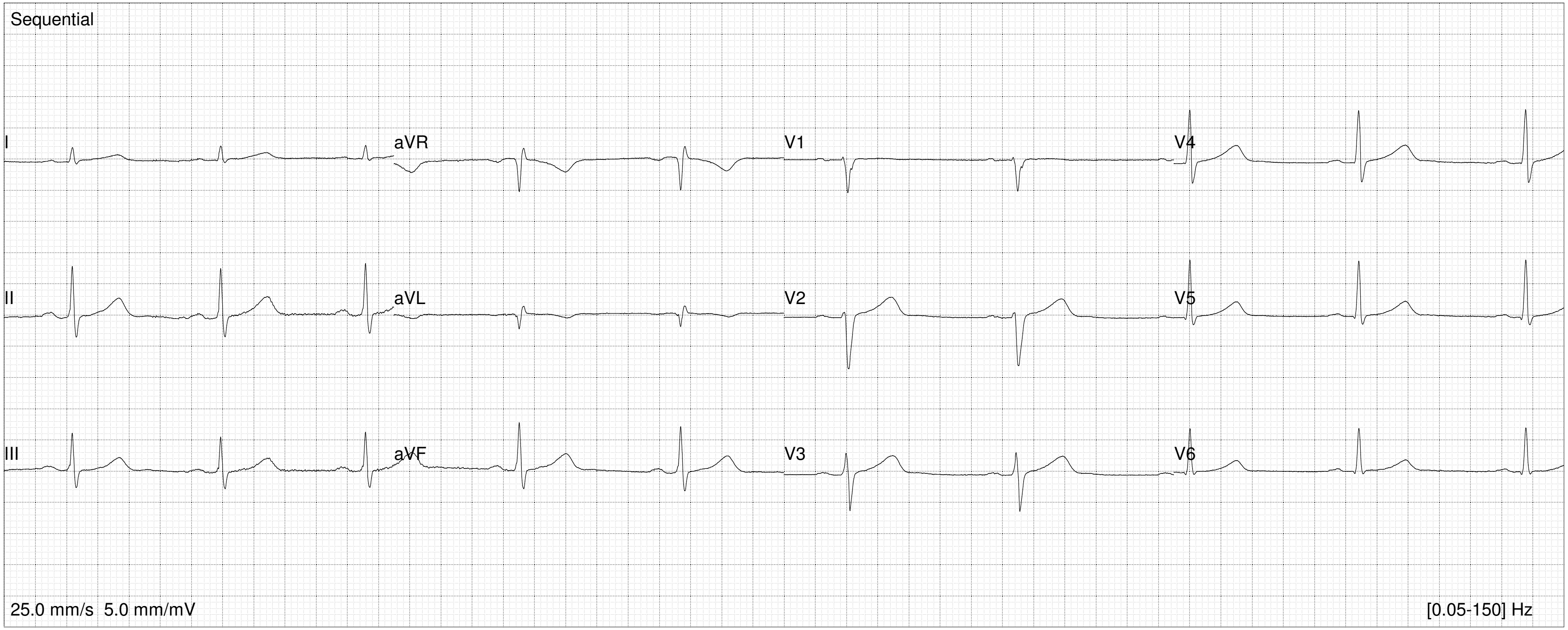

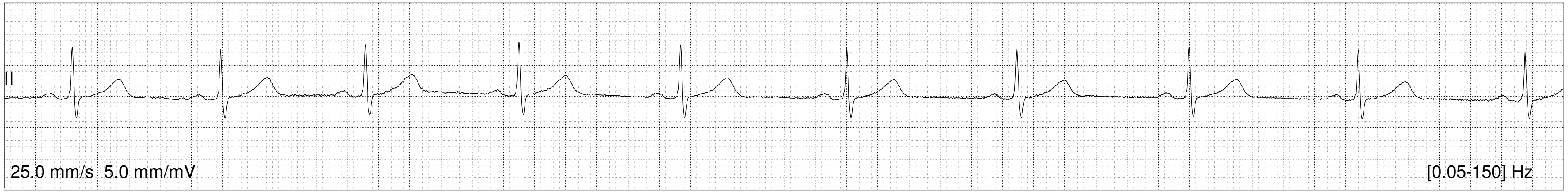

Anton Swart Biokinetic Rehabilitation Practice

Name:	008 008 008	Recorded:	01/05/2018 10:01:04	UNCONFIRMED INTERPRETATION - MD SHOULD REVIEW
Number:	008	Recorded by:	Mr. Anton Swart	
Gender:	Male	Referring physician:		
Birthdate:	13/12/1957 60 years	Location:	Anton Swart Biokinetic Rehabilitation Practice	
		Ordering physician:		
		Attending physician:		
		Comment:		

P / PQ:	115 ms / 168 ms
QRS:	89 ms
QT / QTc / QTd:	439 ms / 436 ms / -
P/QRS/T axis:	75° / 68° / 70°
Heartrate:	58 bpm

**Beats**		**RR**	
Total:	287	Minimum	702 ms
Normal:	287	Maximum	1250 ms
Other:	0	Mean:	1040 ms
		SD:	38 ms

R-R Trend

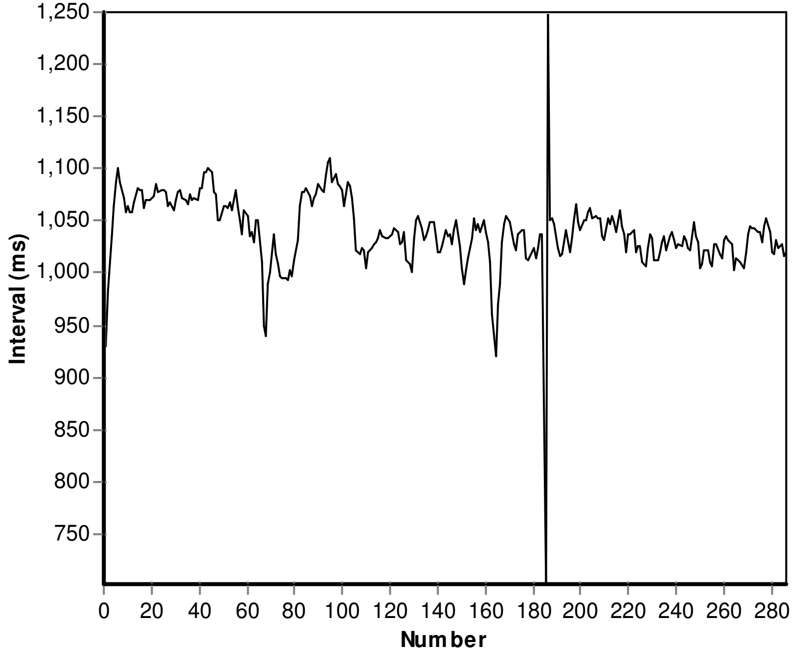

R-R Histogram

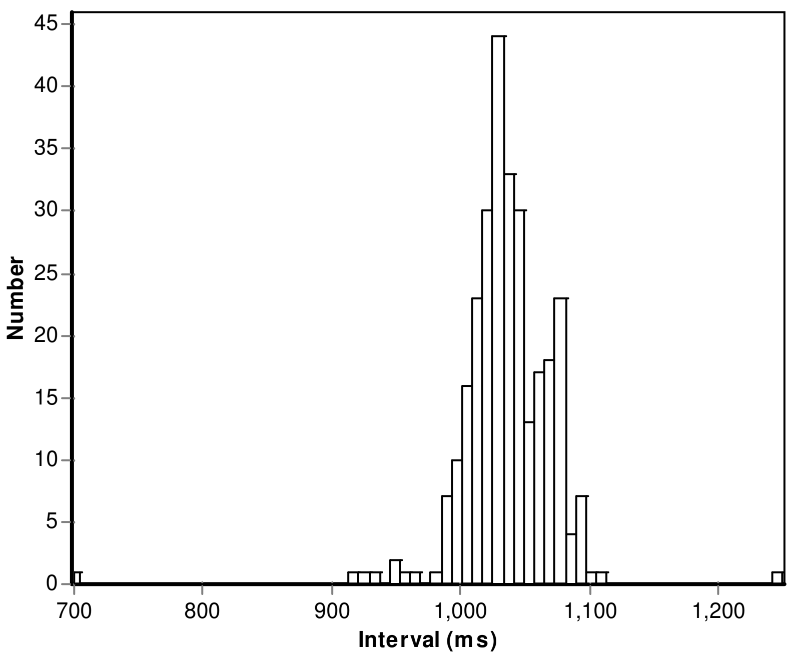

Cardio Control NV. User: Anton

Heart Rate Variability: Time Domain Analysis

**Name:**	008, 008 008	**Birthdate:**	13/12/1957
**Number:**	008	**Recorded:**	01/05/2018 10:01:04
**Gender:**	Male		

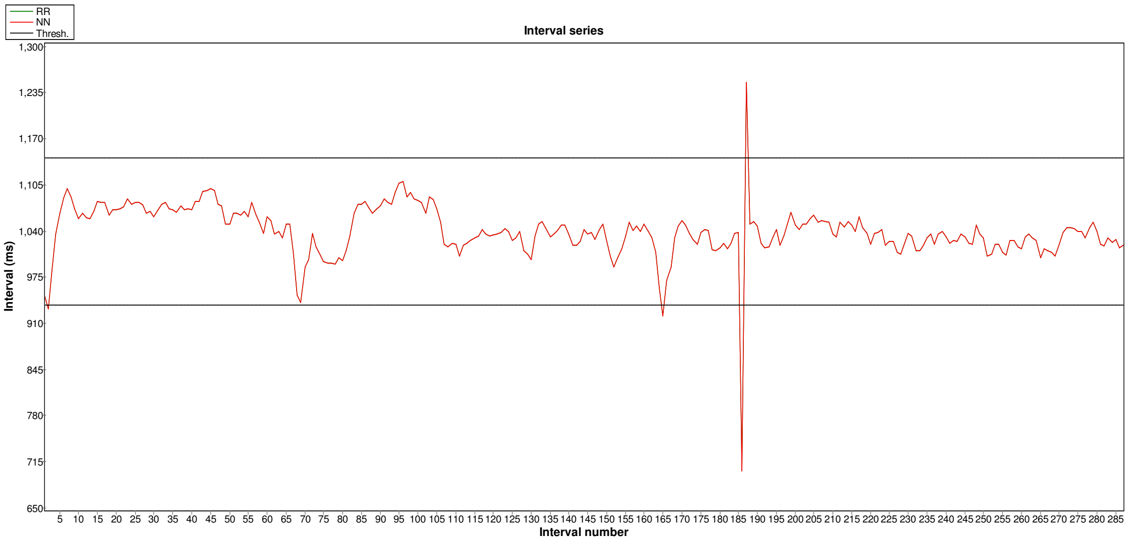

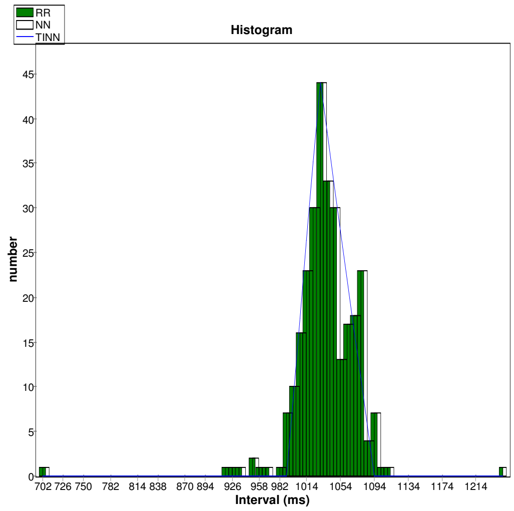

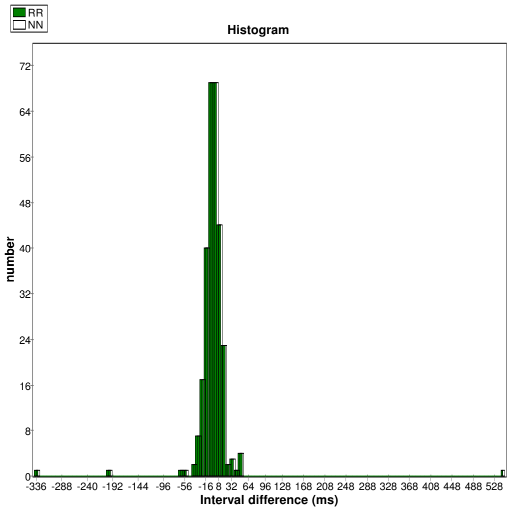

Binsize (ms) = 8

**HRV parameters**	**NN**	**RR**
SDNN (ms)	38	38
Triangular Interpolation (ms)	104	104
Triangular Index	6.52	6.52

**HRV parameters**	**NN**	**RR**
SDSD (ms)	42	42
RMSSD (ms)	42	42
NN50	6	6
NN50(1)	3	3
NN50(2)	3	3
pNN50	0.02	0.02
pNN50(1)	0.01	0.01
pNN50(2)	0.01	0.01
Logarithmic Index	0.52	0.52
SD(Logarithmic Index)	0.07	0.07

**Interval statistics**	**NN**	**RR**
Number	287	287
Minimum (ms)	702	702
Maximum (ms)	1250	1250
Range (ms)	548	548
Avg (ms)	1040	1040
SD (ms)	38	38
AvgDev (ms)	24	24
p5 (ms)	995	995
p50 (ms)	1038	1038
p95 (ms)	1087	1087
Skewness	-2.14	-2.14
Kurtosis	27.67	27.67

**Interval statistics**	**NN**	**RR**
Number	286	286
Minimum (ms)	-336	-336
Maximum (ms)	548	548
Range (ms)	884	884
Avg (ms)	0	0
SD (ms)	42	42
AvgDev (ms)	14	14
p5 (ms)	-23	-23
p50 (ms)	0	0
p95 (ms)	22	22
Skewness	5.44	5.44
Kurtosis	113.18	113.18

Cardio Control NV. User: Anton

Heart Rate Variability: Frequency Domain Analysis

**Name:**	008, 008 008	**Birthdate:**	13/12/1957
**Number:**	008	**Recorded:**	01/05/2018 10:01:04
**Gender:**	Male		

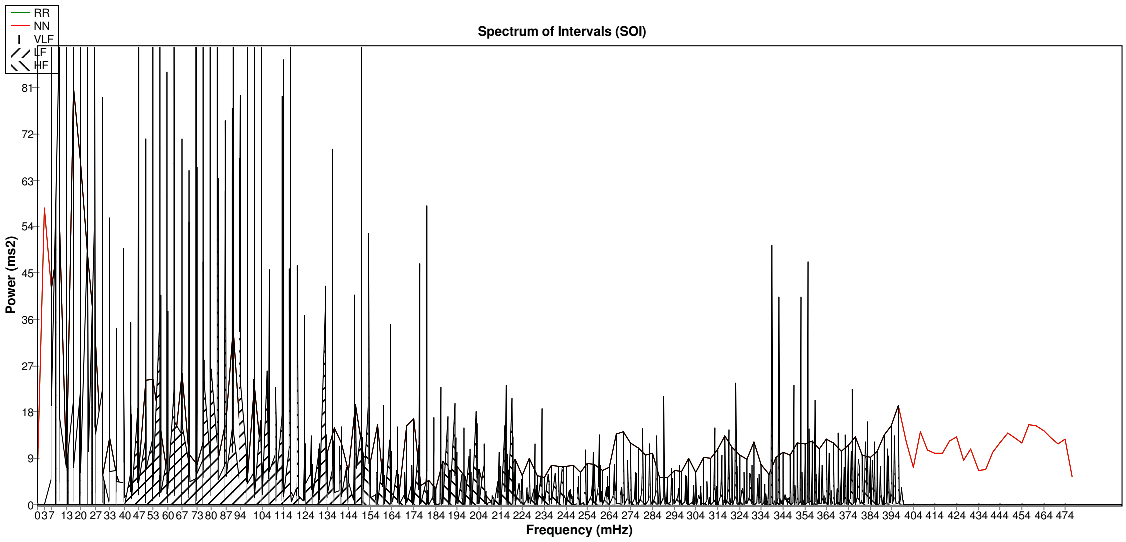

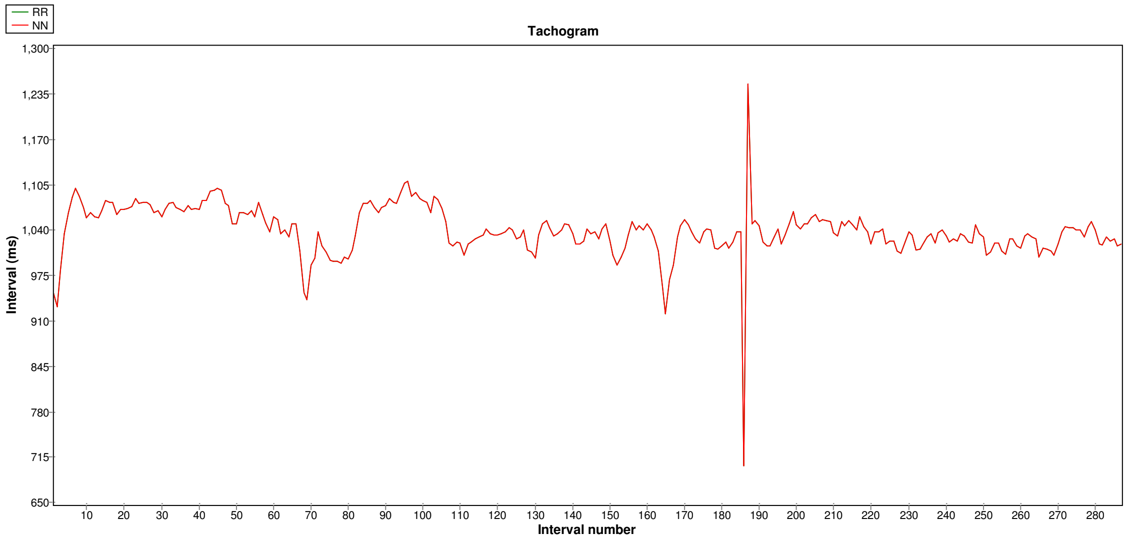

**HRV parameters**	**NN**	**RR**
TP (ms2)	1358	1358
VLF (ms2)	355	355
LF (ms2)	352	352
HF (ms2)	651	651
LF/HF	0.54	0.54
LF normalized	35.08	35.08
HF normalized	64.92	64.92
VLF peak (mHz)	17	17
LF peak (mHz)	90	90
HF peak (mHz)	397	397

**HRV spectral settings**	
Spectrum of Intervals (SOI)	
Frequency resolution (mHz)	3
VLF lower boundary (mHz)	3
VLF upper boundary (mHz)	40
LF upper boundary (mHz)	150
HF upper boundary (mHz)	400
Smoothing factor	1
Tapering	Hann
Fourier transform	DFT
Sample frequency (Hz)	0.96
Interval correction	Annotation
Interval threshold (%)	10
