# Supplementary material for: The effects of a 3-day mountain bike cycling race on the autonomic nervous system (ANS) and heart rate variability in amateur cyclists: a prospective quantitative research design
Source: BMC Sports Sci Med Rehabil. 2023 Jan 2;15:2. doi: 10.1186/s13102-022-00614-y (PMC9808932; doi:10.1186/s13102-022-00614-y)
Supplement: Supplementary file 1 — Additional file 1. Individual data of Participants. [file 13102_2022_614_MOESM1_ESM.zip › Individual data of Participants/HRV Data/008/ECG_008_20180503175329_.PDF]

Anton Swart Biokinetic Rehabilitation Practice

Name: 008 008 008
Number: 008
Gender: Male
Birthdate: 13/12/1957 60 years

P / PQ: 117 ms / 175 ms
QRS: 98 ms
QT / QTc / QTd: 397 ms / 423 ms / -
P/QRS/T axis: 76° / 60° / 75°
Heartrate: 75 bpm

Recorded: 03/05/2018 17:53:29
Recorded by: Mr. Anton Swart
Referring physician:
Ordering physician:
Attending physician:
Location: Anton Swart Biokinetic Rehabilitation Practi
Comment:

UNCONFIRMED INTERPRETATION - MD SHOULD REVIEW

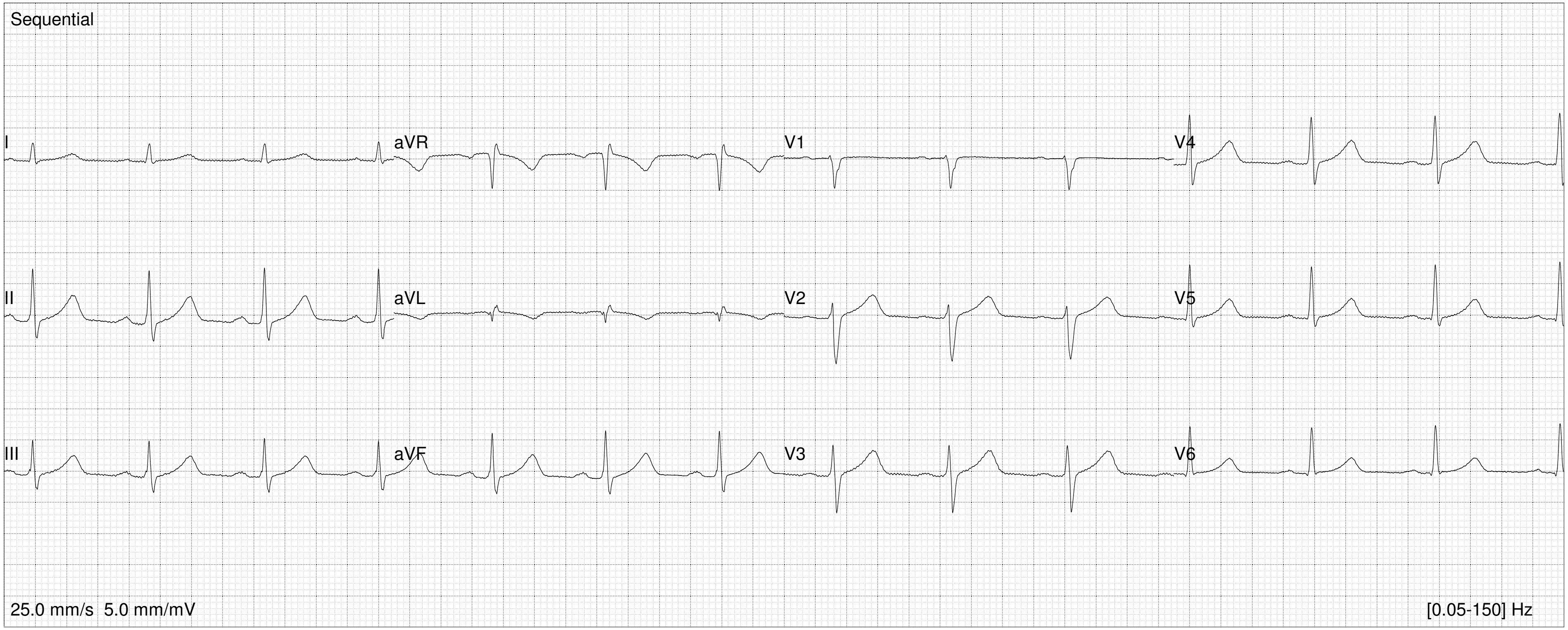

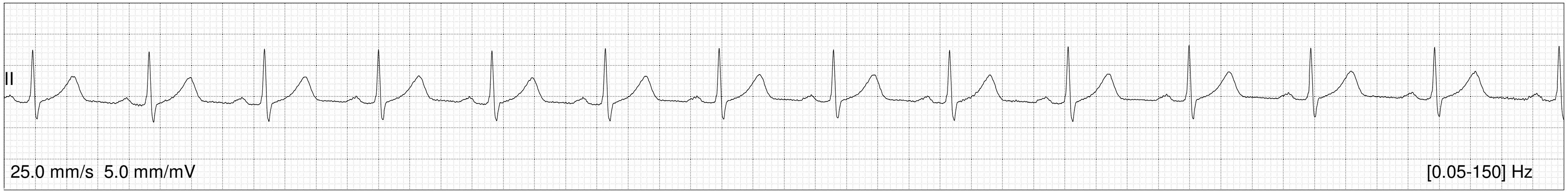

Anton Swart Biokinetic Rehabilitation Practice

Name:	008 008 008	Recorded:	03/05/2018 17:53:29	UNCONFIRMED INTERPRETATION - MD SHOULD REVIEW
Number:	008	Recorded by:	Mr. Anton Swart	
Gender:	Male	Referring physician:		
Birthdate:	13/12/1957 60 years	Location:	Anton Swart Biokinetic Rehabilitation Practice	
		Ordering physician:		
		Attending physician:		
		Comment:		

P / PQ:	117 ms / 175 ms
QRS:	98 ms
QT / QTc / QTd:	397 ms / 423 ms / -
P/QRS/T axis:	76° / 60° / 75°
Heartrate:	75 bpm

Beats

Total:	376
Normal:	376
Other:	0

RR

Minimum	727 ms
Maximum	880 ms
Mean:	796 ms
SD:	25 ms

R-R Trend

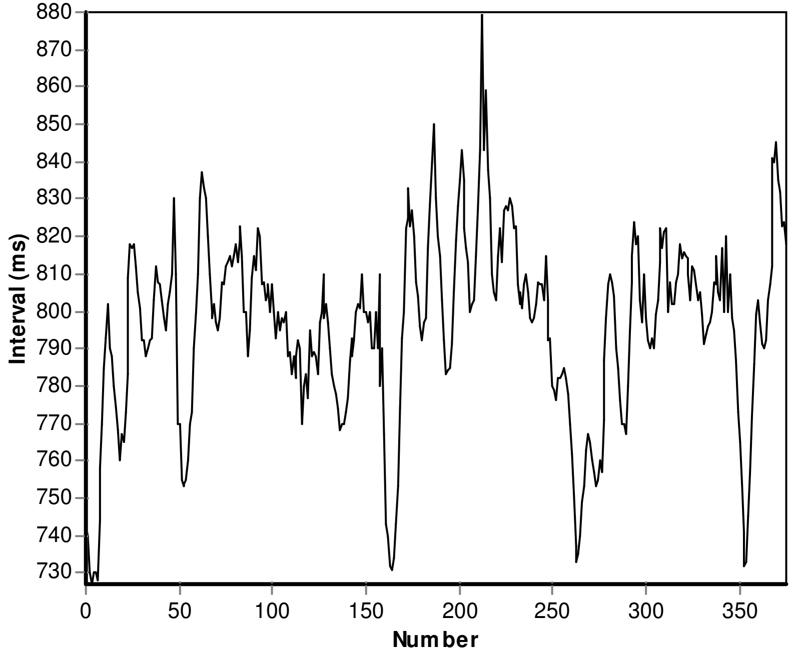

R-R Histogram

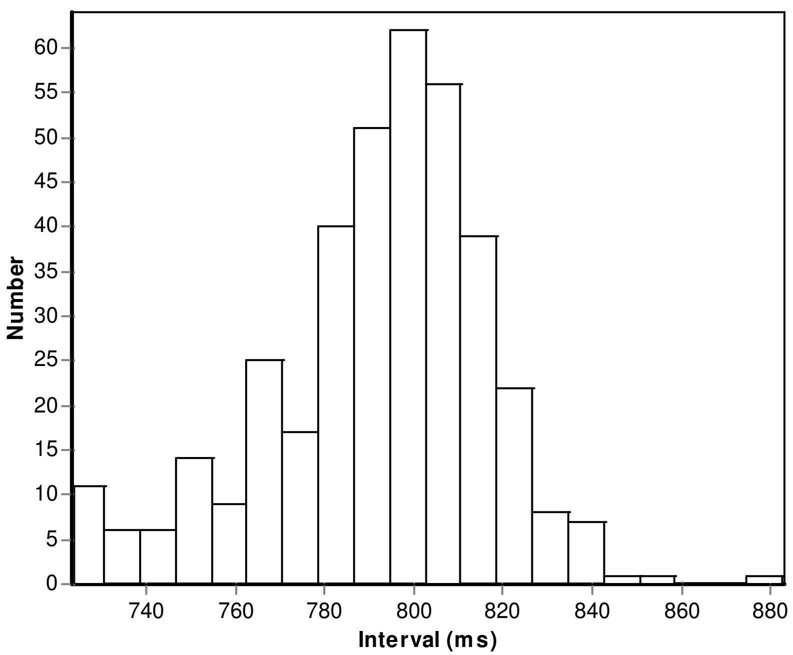

Cardio Control NV. User: Anton

Heart Rate Variability: Time Domain Analysis

**Name:**	008, 008 008	**Birthdate:**	13/12/1957
**Number:**	008	**Recorded:**	03/05/2018 17:53:29
**Gender:**	Male		

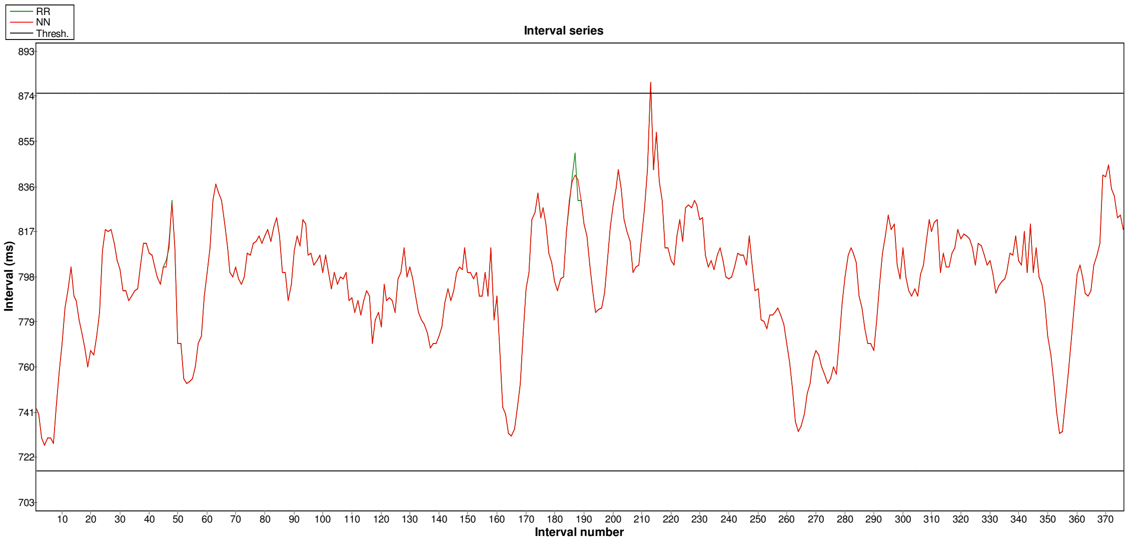

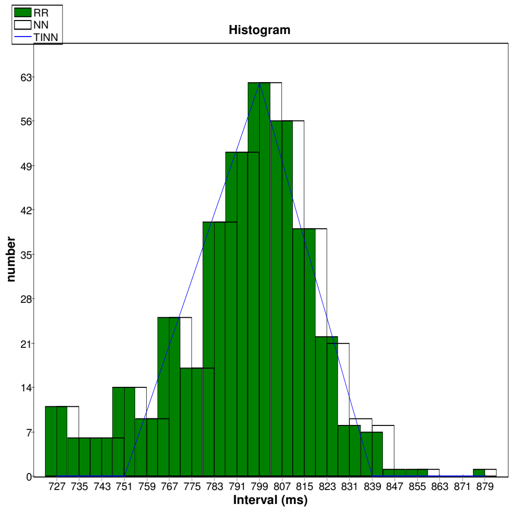

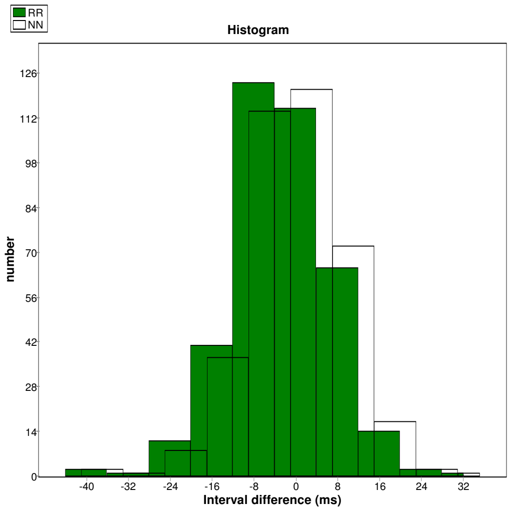

Binsize (ms) = 8

**HRV parameters**	**NN**	**RR**
SDNN (ms)	25	25
Triangular Interpolation (ms)	88	88
Triangular Index	6.06	6.06

**HRV parameters**	**NN**	**RR**
SDSD (ms)	9	10
RMSSD (ms)	9	10
NN50	0	0
NN50(1)	0	0
NN50(2)	0	0
pNN50	0.00	0.00
pNN50(1)	0.00	0.00
pNN50(2)	0.00	0.00
Logarithmic Index	1.18	1.18
SD(Logarithmic Index)	0.10	0.10

**Interval statistics**	**NN**	**RR**
Number	376	376
Minimum (ms)	727	727
Maximum (ms)	880	880
Range (ms)	153	153
Avg (ms)	796	796
SD (ms)	25	25
AvgDev (ms)	19	19
p5 (ms)	743	743
p50 (ms)	800	800
p95 (ms)	833	831
Skewness	-0.57	-0.56
Kurtosis	3.55	3.56

**Interval statistics**	**NN**	**RR**
Number	375	375
Minimum (ms)	-41	-40
Maximum (ms)	37	37
Range (ms)	78	77
Avg (ms)	0	0
SD (ms)	9	10
AvgDev (ms)	7	7
p5 (ms)	-14	-14
p50 (ms)	0	0
p95 (ms)	15	15
Skewness	-0.17	-0.17
Kurtosis	4.73	4.61

Heart Rate Variability: Frequency Domain Analysis

Name: 008, 008 008
Number: 008
Gender: Male
Birthdate: 13/12/1957
Recorded: 03/05/2018 17:53:29

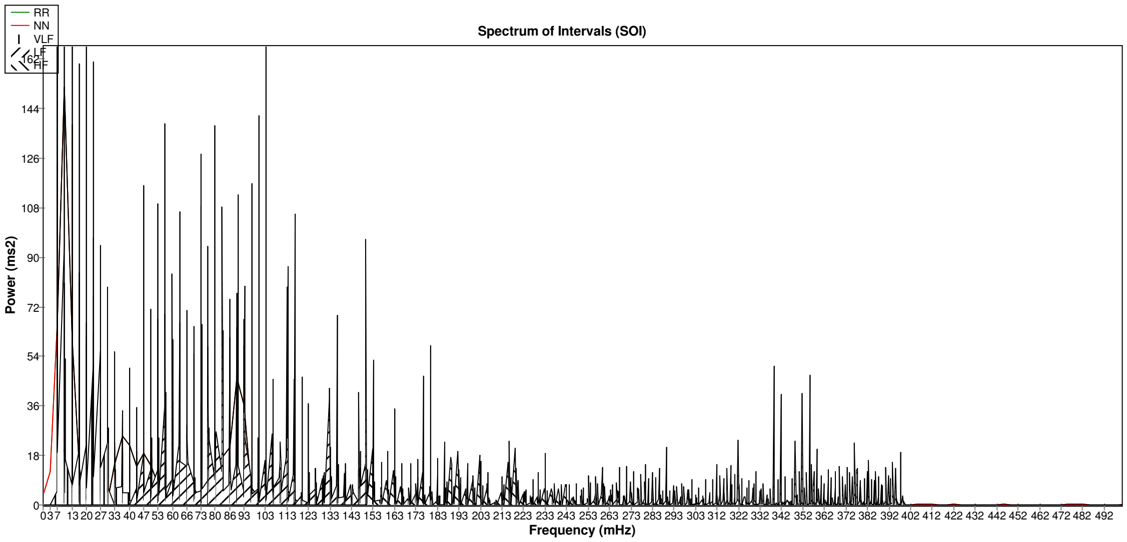

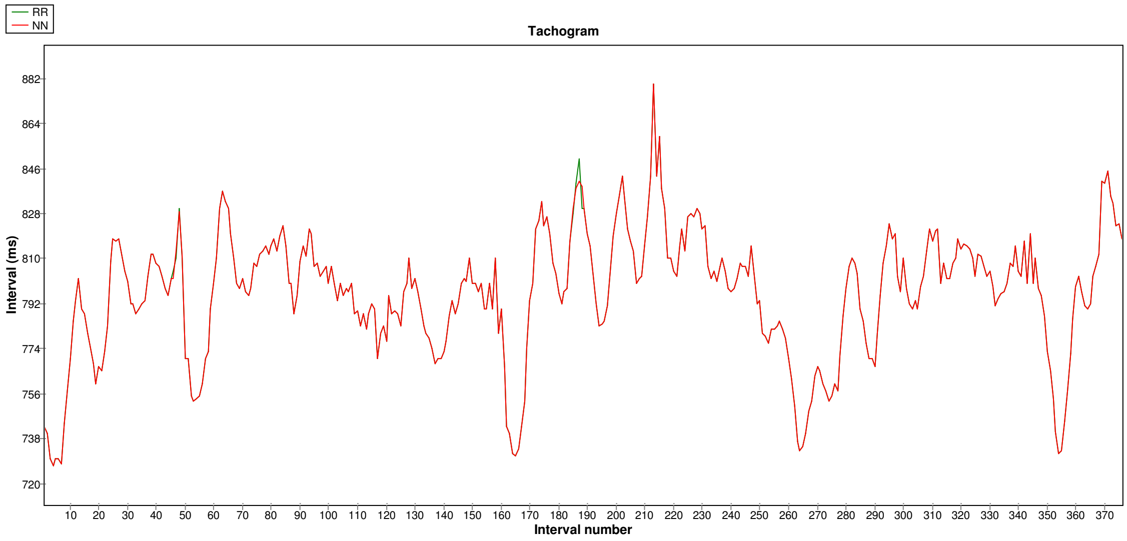

HRV parameters	NN	RR
TP (ms2)	648	649
VLF (ms2)	377	378
LF (ms2)	260	260
HF (ms2)	10	11
LF/HF	25.89	24.08
LF normalized	96.28	96.01
HF normalized	3.72	3.99
VLF peak (mHz)	10	10
LF peak (mHz)	90	90
HF peak (mHz)	173	173

HRV spectral settings	
Spectrum of Intervals (SOI)	
Frequency resolution (mHz)	3
VLF lower boundary (mHz)	3
VLF upper boundary (mHz)	40
LF upper boundary (mHz)	150
HF upper boundary (mHz)	400
Smoothing factor	1
Tapering	Hann
Fourier transform	DFT
Sample frequency (Hz)	1.26
Interval correction	Annotation
Interval threshold (%)	10
