# Supplementary material for: The effects of a 3-day mountain bike cycling race on the autonomic nervous system (ANS) and heart rate variability in amateur cyclists: a prospective quantitative research design
Source: BMC Sports Sci Med Rehabil. 2023 Jan 2;15:2. doi: 10.1186/s13102-022-00614-y (PMC9808932; doi:10.1186/s13102-022-00614-y)
Supplement: Supplementary file 1 — Additional file 1. Individual data of Participants. [file 13102_2022_614_MOESM1_ESM.zip › Individual data of Participants/HRV Data/008/ECG_008_20180504131844_.PDF]

Anton Swart Biokinetic Rehabilitation Practice

Name:	008 008 008	Recorded:	04/05/2018 13:18:44	UNCONFIRMED INTERPRETATION - MD SHOULD REVIEW
Number:	008	Recorded by:	Mr. Anton Swart	
Gender:	Male	Referring physician:		
Birthdate:	13/12/1957 60 years	Ordering physician:		
		Attending physician:		
P / PQ:	113 ms / 162 ms	Location:	Anton Swart Biokinetic Rehabilitation Practi	
QRS:	88 ms	Comment:		
QT / QTc / QTd:	396 ms / 445 ms / -			
P/QRS/T axis:	75° / 65° / 74°			
Heartrate:	88 bpm			

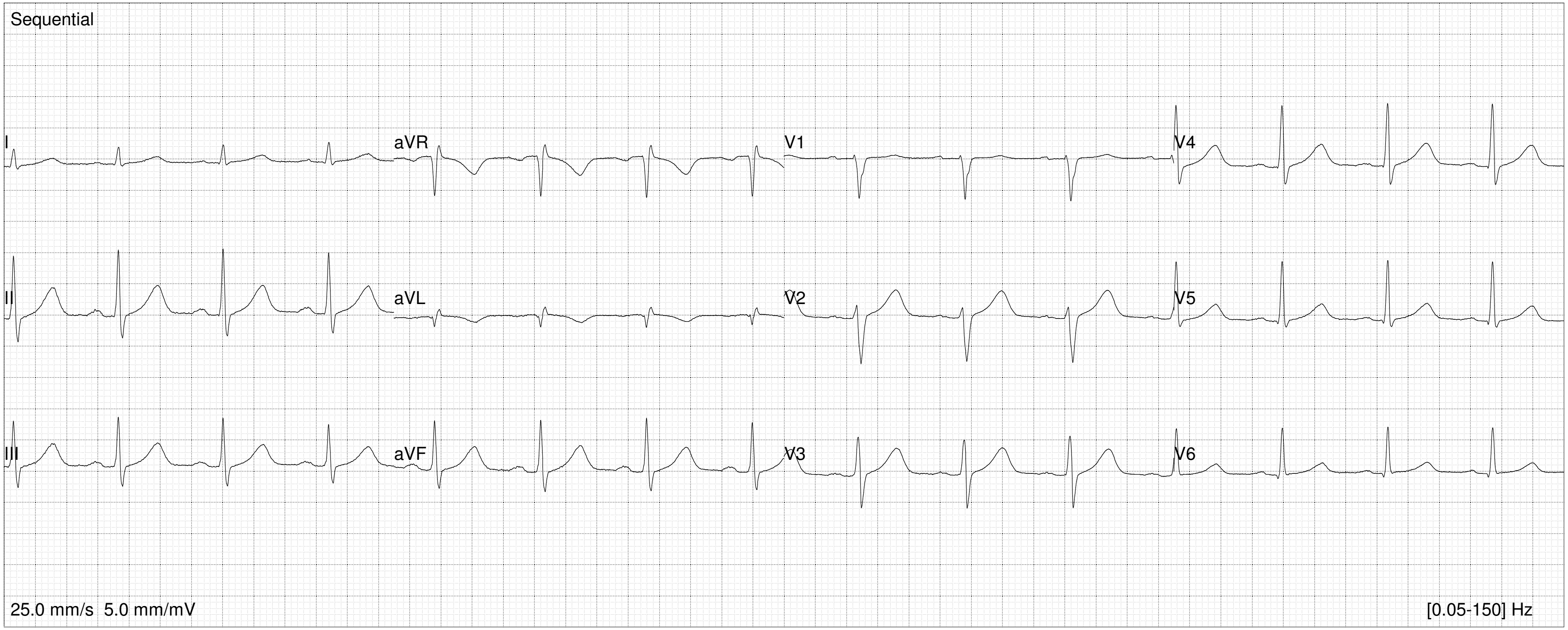

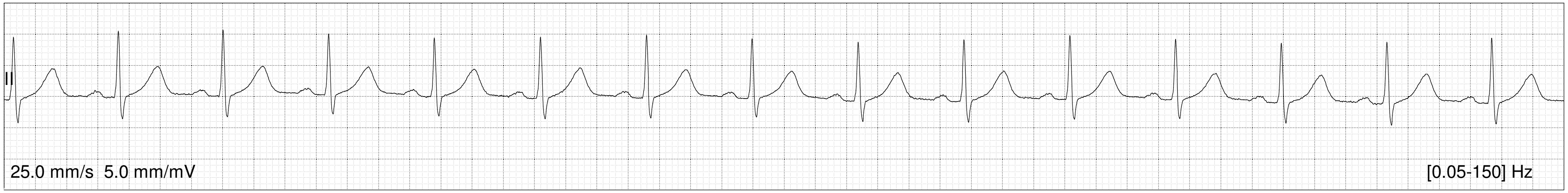

Anton Swart Biokinetic Rehabilitation Practice

Name:	008 008 008	Recorded:	04/05/2018 13:18:44	UNCONFIRMED INTERPRETATION - MD SHOULD REVIEW
Number:	008	Recorded by:	Mr. Anton Swart	
Gender:	Male	Referring physician:		
Birthdate:	13/12/1957 60 years	Location:	Anton Swart Biokinetic Rehabilitation Practice	
P / PQ:	113 ms / 162 ms	Ordering physician:		
QRS:	88 ms	Attending physician:		
QT / QTc / QTd:	396 ms / 445 ms / -	Comment:		
P/QRS/T axis:	75° / 65° / 74°			
Heartrate:	88 bpm			

Beats

Total:	442
Normal:	442
Other:	0

RR

Minimum	550 ms
Maximum	768 ms
Mean:	677 ms
SD:	14 ms

R-R Trend

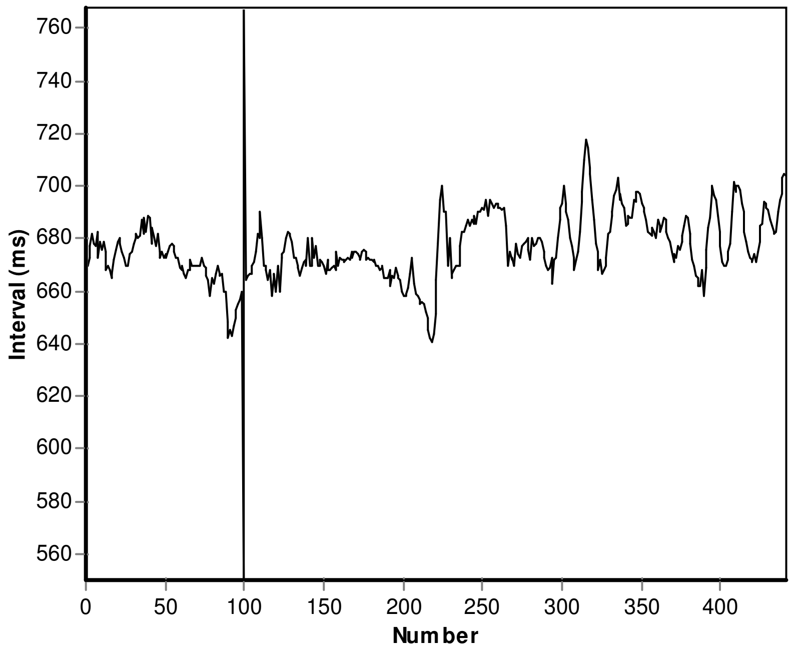

R-R Histogram

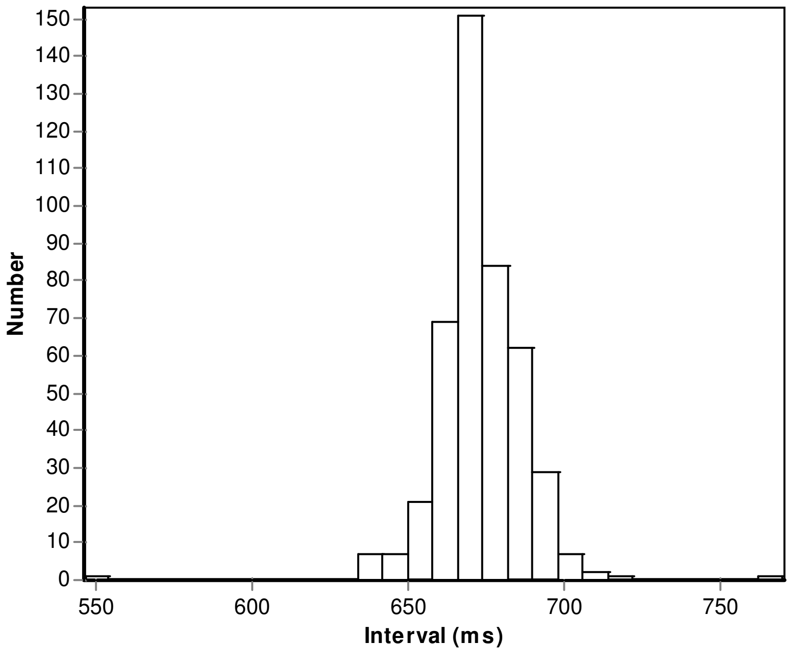

Heart Rate Variability: Time Domain Analysis

**Name:**	008, 008 008	**Birthdate:**	13/12/1957
**Number:**	008	**Recorded:**	04/05/2018 13:18:44
**Gender:**	Male		

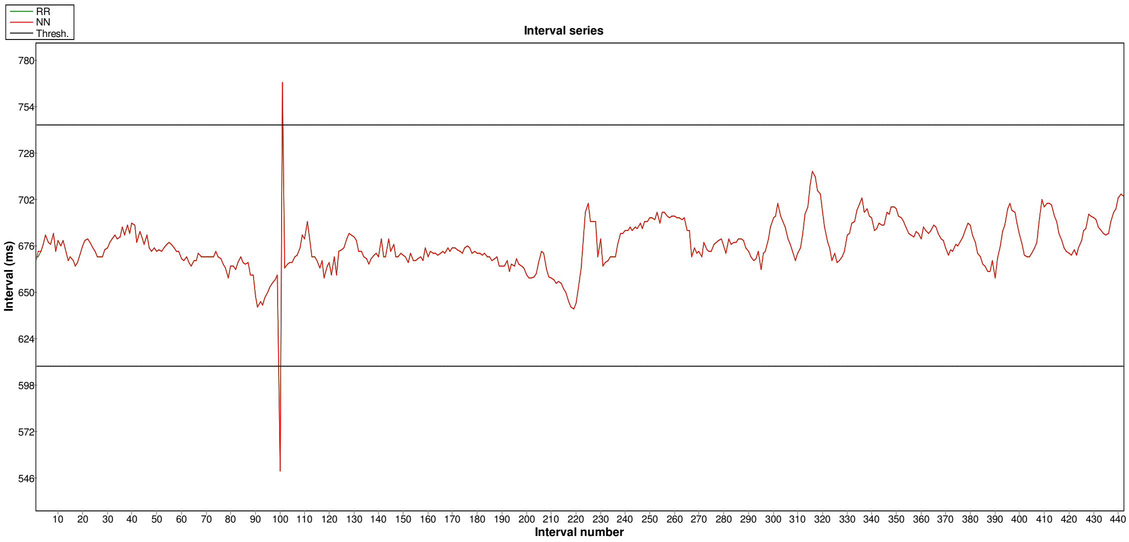

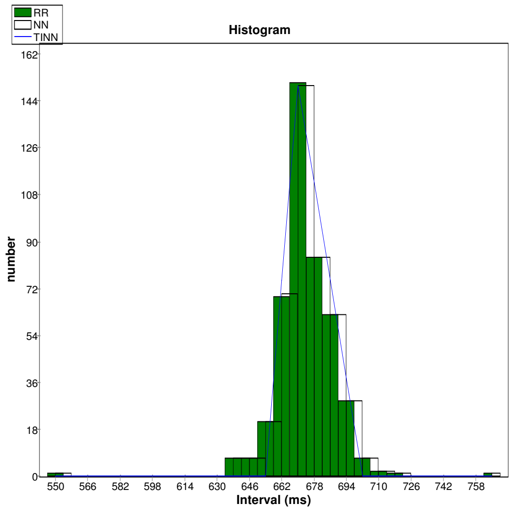

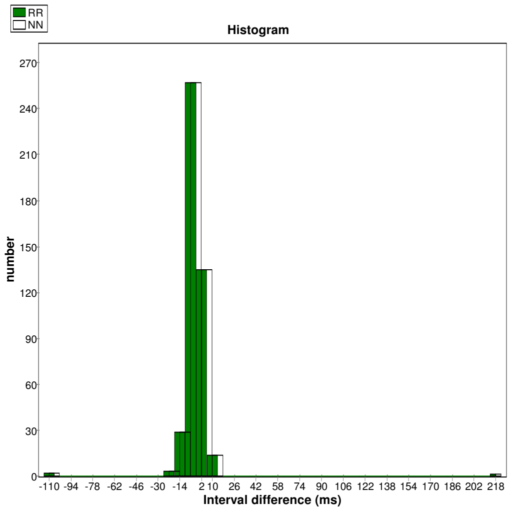

Binsize (ms) = 8

**HRV parameters**	**NN**	**RR**
SDNN (ms)	14	14
Triangular Interpolation (ms)	48	48
Triangular Index	2.95	2.93

**HRV parameters**	**NN**	**RR**
SDSD (ms)	13	13
RMSSD (ms)	13	13
NN50	3	3
NN50(1)	2	2
NN50(2)	1	1
pNN50	0.01	0.01
pNN50(1)	0.00	0.00
pNN50(2)	0.00	0.00
Logarithmic Index	1.50	1.50
SD(Logarithmic Index)	0.34	0.34

**Interval statistics**	**NN**	**RR**
Number	442	442
Minimum (ms)	550	550
Maximum (ms)	768	768
Range (ms)	218	218
Avg (ms)	677	677
SD (ms)	14	14
AvgDev (ms)	10	10
p5 (ms)	658	658
p50 (ms)	675	675
p95 (ms)	698	698
Skewness	-0.90	-0.90
Kurtosis	19.83	19.84

**Interval statistics**	**NN**	**RR**
Number	441	441
Minimum (ms)	-110	-110
Maximum (ms)	218	218
Range (ms)	328	328
Avg (ms)	0	0
SD (ms)	13	13
AvgDev (ms)	4	4
p5 (ms)	-7	-7
p50 (ms)	0	0
p95 (ms)	8	8
Skewness	7.33	7.34
Kurtosis	174.06	174.19

Heart Rate Variability: Frequency Domain Analysis

**Name:**	008, 008 008	**Birthdate:**	13/12/1957
**Number:**	008	**Recorded:**	04/05/2018 13:18:44
**Gender:**	Male		

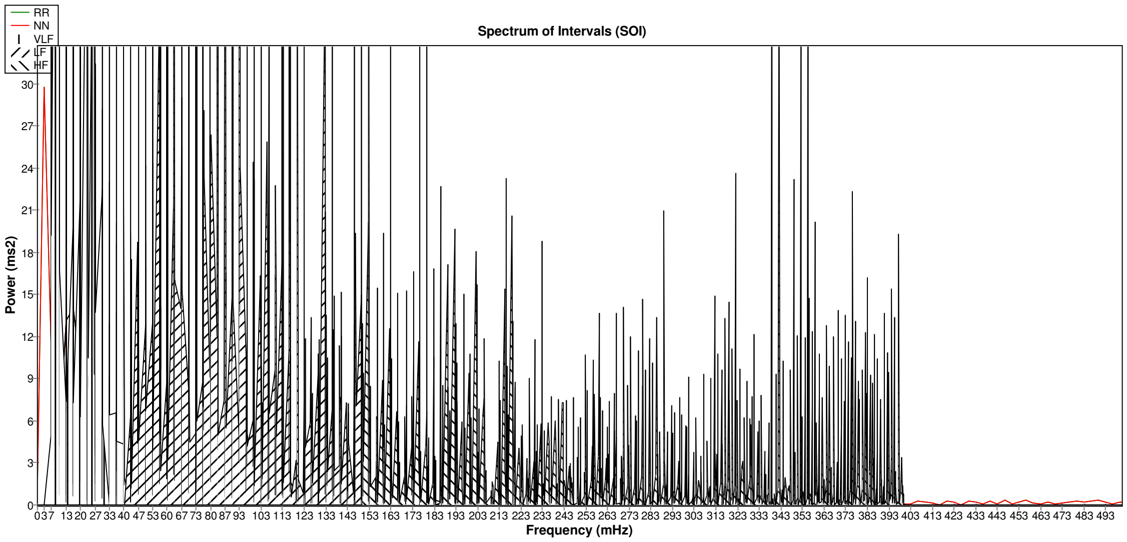

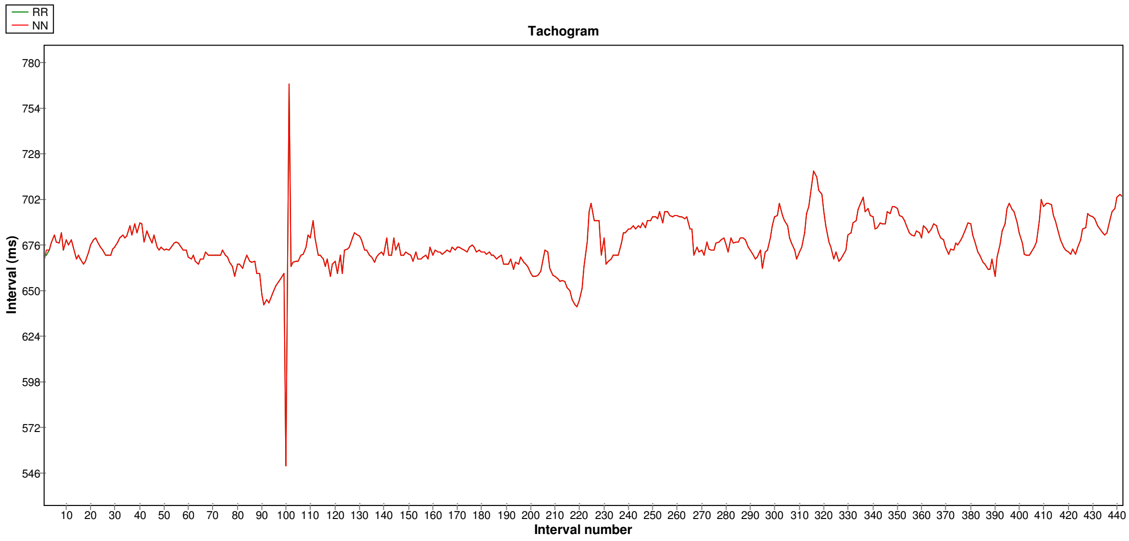

**HRV parameters**	**NN**	**RR**
TP (ms2)	136	136
VLF (ms2)	68	68
LF (ms2)	58	58
HF (ms2)	11	11
LF/HF	5.49	5.49
LF normalized	84.60	84.60
HF normalized	15.40	15.40
VLF peak (mHz)	17	17
LF peak (mHz)	77	77
HF peak (mHz)	156	156

**HRV spectral settings**	
Spectrum of Intervals (SOI)	
Frequency resolution (mHz)	3
VLF lower boundary (mHz)	3
VLF upper boundary (mHz)	40
LF upper boundary (mHz)	150
HF upper boundary (mHz)	400
Smoothing factor	1
Tapering	Hann
Fourier transform	DFT
Sample frequency (Hz)	1.48
Interval correction	Annotation
Interval threshold (%)	10
