# Supplementary material for: The effects of a 3-day mountain bike cycling race on the autonomic nervous system (ANS) and heart rate variability in amateur cyclists: a prospective quantitative research design
Source: BMC Sports Sci Med Rehabil. 2023 Jan 2;15:2. doi: 10.1186/s13102-022-00614-y (PMC9808932; doi:10.1186/s13102-022-00614-y)
Supplement: Supplementary file 1 — Additional file 1. Individual data of Participants. [file 13102_2022_614_MOESM1_ESM.zip › Individual data of Participants/HRV Data/008/ECG_008_20180505133144_.PDF]

# Anton Swart Biokinetic Rehabilitation Practice

UNCONFIRMED INTERPRETATION - MD SHOULD REVIEW

| | | | |
|---|---|---|---|
| Name: | 008 008 008 | Recorded: | 05/05/2018 13:31:44 |
| Number: | 008 | Recorded by: | Mr. Anton Swart |
| Gender: | Male | Referring physician: | |
| Birthdate: | 13/12/1957 60 years | Ordering physician: | |
| | | Attending physician: | |
| P / PQ: | 113 ms / 163 ms | Location: | Anton Swart Biokinetic Rehabilitation Practi |
| QRS: | 87 ms | Comment: | |
| QT / QTc / QTd: | 407 ms / 451 ms / - | | |
| P/QRS/T axis: | 77° / 67° / 74° | | |
| Heartrate: | 85 bpm | | |

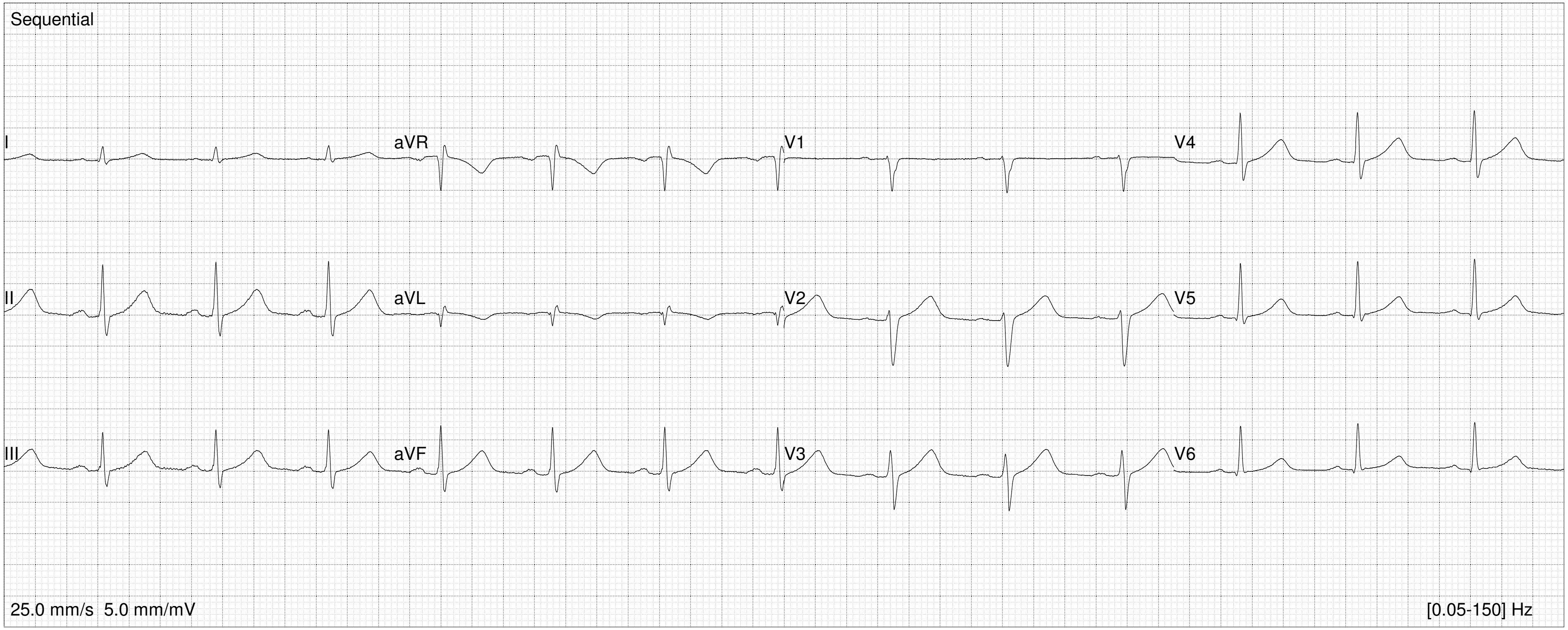


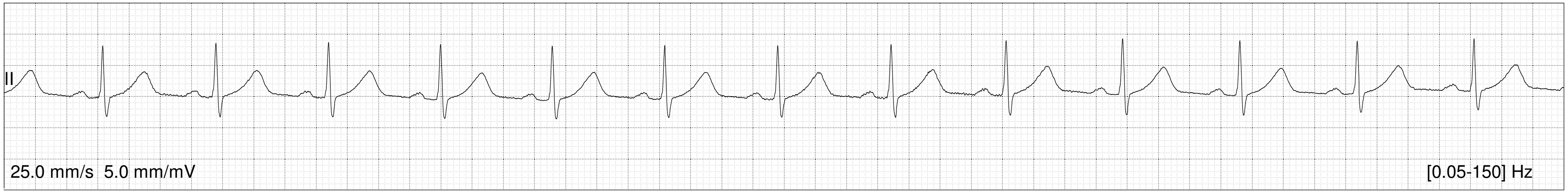

# Anton Swart Biokinetic Rehabilitation Practice

| | | | | |
|---|---|---|---|---|
| Name: | 008 008 008 | Recorded: | 05/05/2018 13:31:44 | UNCONFIRMED INTERPRETATION - MD SHOULD REVIEW |
| Number: | 008 | Recorded by: | Mr. Anton Swart | |
| Gender: | Male | Referring physician: | | |
| Birthdate: | 13/12/1957 60 years | Location: | Anton Swart Biokinetic Rehabilitation Practice | |
| P / PQ: | 113 ms / 163 ms | Ordering physician: | | |
| QRS: | 87 ms | Attending physician: | | |
| QT / QTc / QTd: | 407 ms / 451 ms / - | Comment: | | |
| P/QRS/T axis: | 77° / 67° / 74° | | | |
| Heartrate: | 85 bpm | | | |

| **Beats** | | **RR** | |
|---|---|---|---|
| Total: | 418 | Minimum | 570 ms |
| Normal: | 418 | Maximum | 970 ms |
| Other: | 0 | Mean: | 715 ms |
| | | SD: | 25 ms |

## R-R Trend

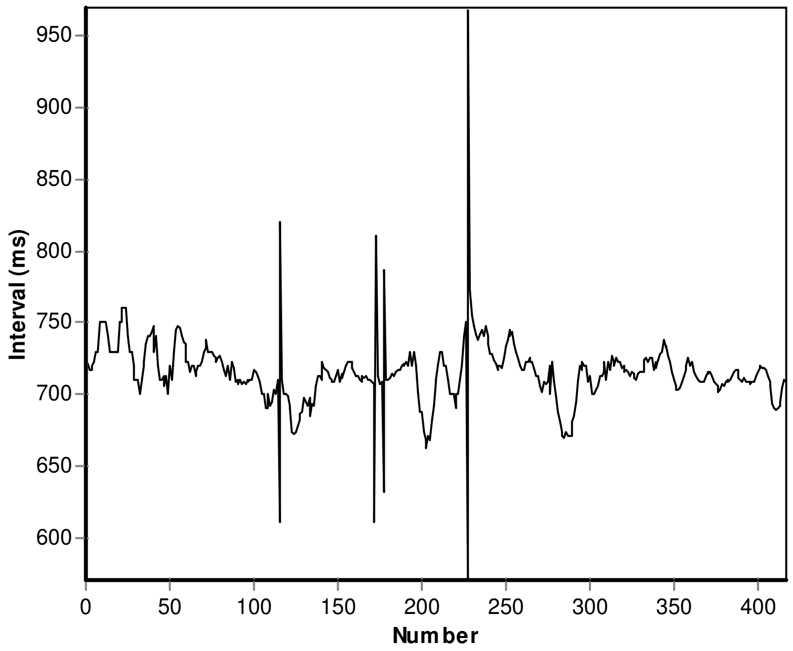


## R-R Histogram

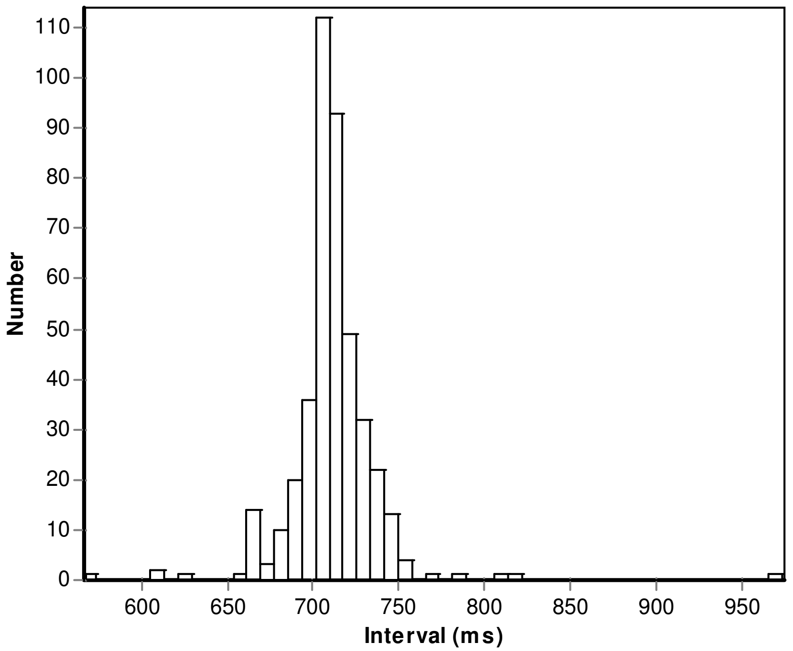

Cardio Control NV. User: Anton

## Heart Rate Variability: Time Domain Analysis

| | | | |
|---|---|---|---|
| **Name:** | 008, 008 008 | **Birthdate:** | 13/12/1957 |
| **Number:** | 008 | **Recorded:** | 05/05/2018 13:31:44 |
| **Gender:** | Male | | |

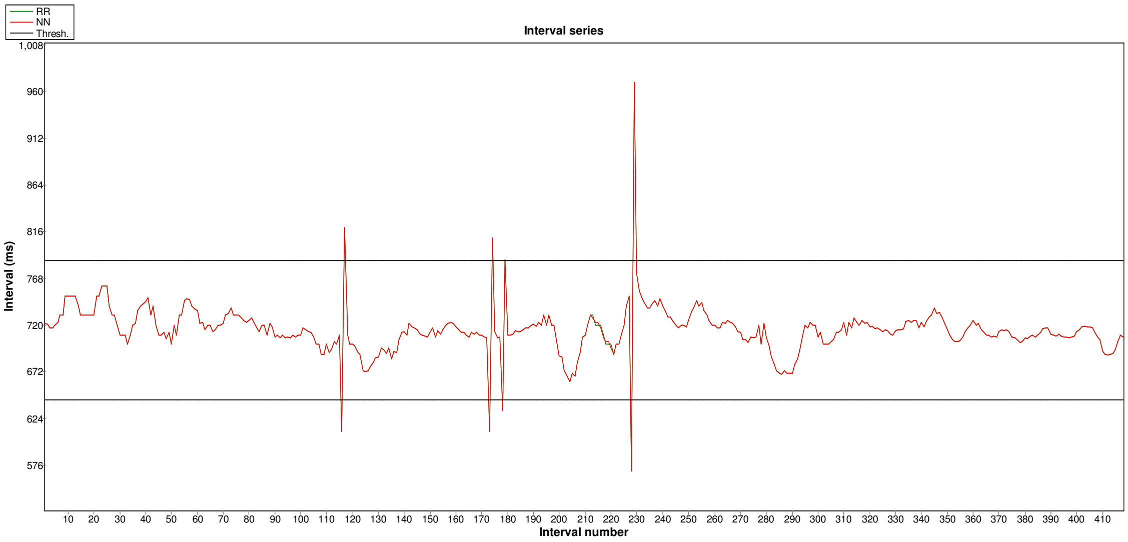


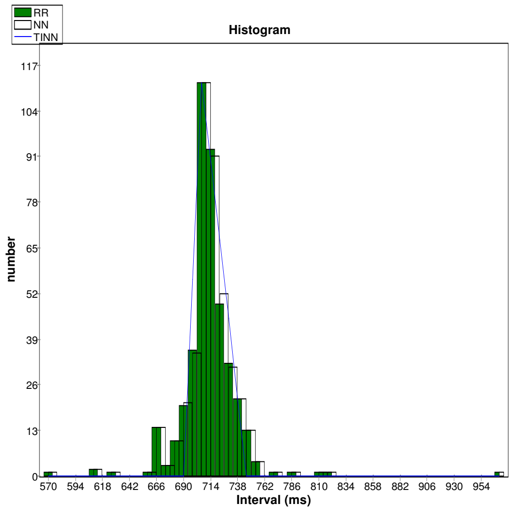


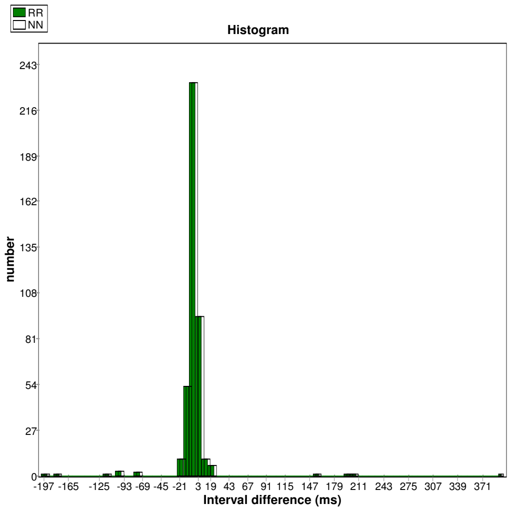


Binsize (ms) = 8

| **HRV parameters** | **NN** | **RR** |
|---|---|---|
| SDNN (ms) | 25 | 25 |
| Triangular Interpolation (ms) | 56 | 56 |
| Triangular Index | 3.73 | 3.73 |

| **HRV parameters** | **NN** | **RR** |
|---|---|---|
| SDSD (ms) | 31 | 31 |
| RMSSD (ms) | 31 | 31 |
| NN50 | 12 | 12 |
| NN50(1) | 8 | 8 |
| NN50(2) | 4 | 4 |
| pNN50 | 0.03 | 0.03 |
| pNN50(1) | 0.02 | 0.02 |
| pNN50(2) | 0.01 | 0.01 |
| Logarithmic Index | 0.44 | 0.45 |
| SD(Logarithmic Index) | 0.10 | 0.10 |

| **Interval statistics** | **NN** | **RR** |
|---|---|---|
| Number | 418 | 418 |
| Minimum (ms) | 570 | 570 |
| Maximum (ms) | 970 | 970 |
| Range (ms) | 400 | 400 |
| Avg (ms) | 715 | 715 |
| SD (ms) | 25 | 25 |
| AvgDev (ms) | 14 | 14 |
| p5 (ms) | 679 | 679 |
| p50 (ms) | 715 | 715 |
| p95 (ms) | 747 | 747 |
| Skewness | 1.97 | 1.97 |
| Kurtosis | 33.68 | 33.67 |

| **Interval statistics** | **NN** | **RR** |
|---|---|---|
| Number | 417 | 417 |
| Minimum (ms) | -197 | -197 |
| Maximum (ms) | 400 | 400 |
| Range (ms) | 597 | 597 |
| Avg (ms) | -0 | -0 |
| SD (ms) | 31 | 31 |
| AvgDev (ms) | 9 | 9 |
| p5 (ms) | -12 | -12 |
| p50 (ms) | 0 | 0 |
| p95 (ms) | 12 | 12 |
| Skewness | 5.20 | 5.19 |
| Kurtosis | 81.79 | 81.74 |

Cardio Control NV. User: Anton

## Heart Rate Variability: Frequency Domain Analysis

| | | | |
|---|---|---|---|
| **Name:** | 008, 008 008 | **Birthdate:** | 13/12/1957 |
| **Number:** | 008 | **Recorded:** | 05/05/2018 13:31:44 |
| **Gender:** | Male | | |

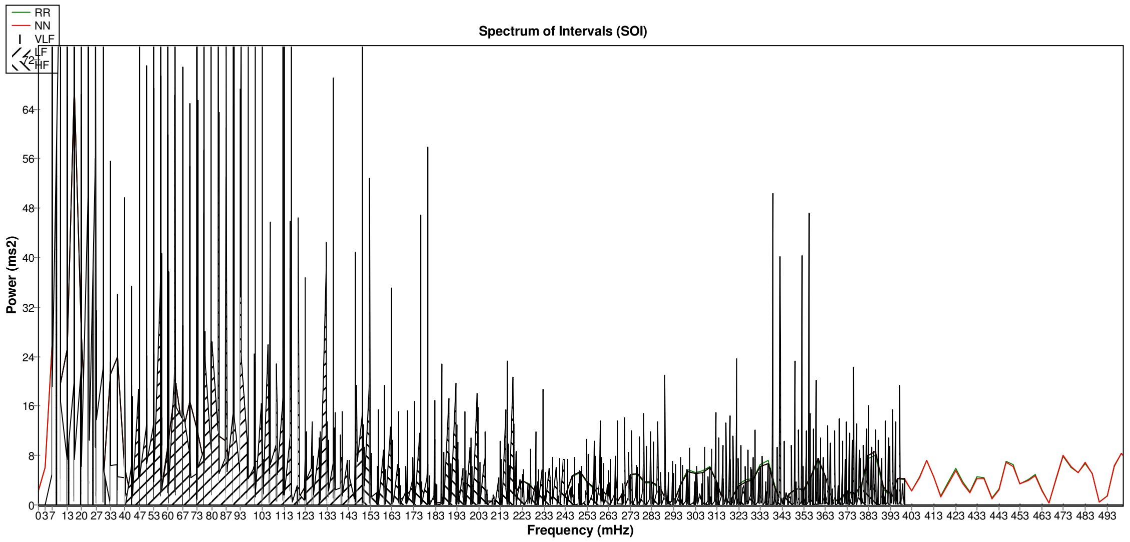


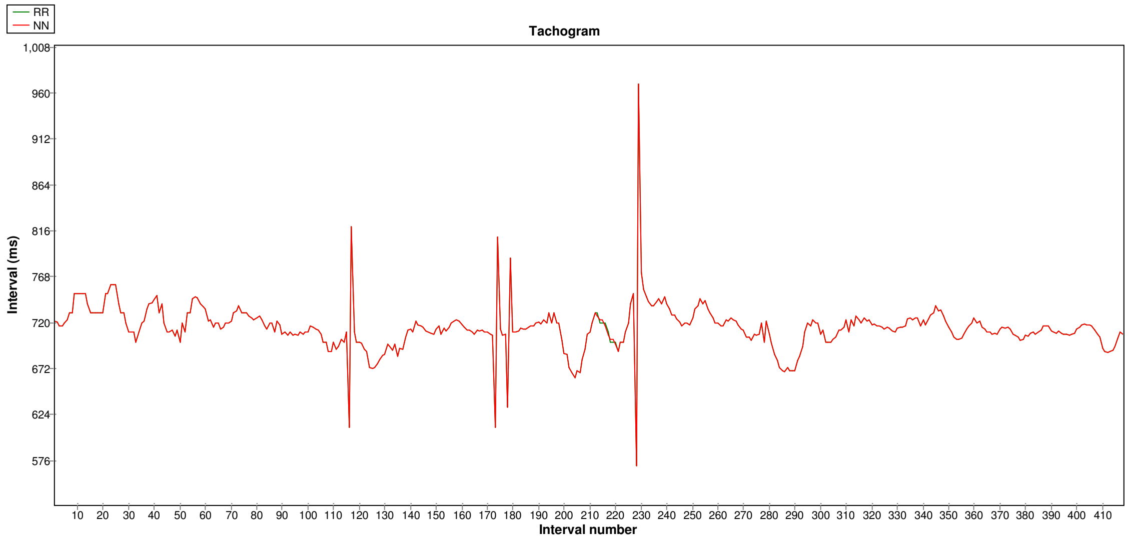


| HRV parameters | NN | RR |
|---|---|---|
| TP (ms2) | 582 | 581 |
| VLF (ms2) | 227 | 227 |
| LF (ms2) | 153 | 153 |
| HF (ms2) | 203 | 201 |
| LF/HF | 0.75 | 0.76 |
| LF normalized | 42.99 | 43.15 |
| HF normalized | 57.01 | 56.85 |
| VLF peak (mHz) | 17 | 17 |
| LF peak (mHz) | 63 | 63 |
| HF peak (mHz) | 386 | 386 |

| HRV spectral settings | |
|---|---|
| Spectrum of Intervals (SOI) | |
| Frequency resolution (mHz) | 3 |
| VLF lower boundary (mHz) | 3 |
| VLF upper boundary (mHz) | 40 |
| LF upper boundary (mHz) | 150 |
| HF upper boundary (mHz) | 400 |
| Smoothing factor | 1 |
| Tapering | Hann |
| Fourier transform | DFT |
| Sample frequency (Hz) | 1.40 |
| Interval correction | Annotation |
| Interval threshold (%) | 10 |
