# Supplementary material for: The effects of a 3-day mountain bike cycling race on the autonomic nervous system (ANS) and heart rate variability in amateur cyclists: a prospective quantitative research design
Source: BMC Sports Sci Med Rehabil. 2023 Jan 2;15:2. doi: 10.1186/s13102-022-00614-y (PMC9808932; doi:10.1186/s13102-022-00614-y)
Supplement: Supplementary file 1 — Additional file 1. Individual data of Participants. [file 13102_2022_614_MOESM1_ESM.zip › Individual data of Participants/HRV Data/008/ECG_008_20180506082447_.PDF]

Anton Swart Biokinetic Rehabilitation Practice

Name:	008 008 008	Recorded:	06/05/2018 08:24:47	UNCONFIRMED INTERPRETATION - MD SHOULD REVIEW
Number:	008	Recorded by:	Mr. Anton Swart	
Gender:	Male	Referring physician:		
Birthdate:	13/12/1957 60 years	Ordering physician:		
		Attending physician:		
P / PQ:	105 ms / 160 ms	Location:	Anton Swart Biokinetic Rehabilitation Practi	
QRS:	87 ms	Comment:		
QT / QTc / QTd:	453 ms / 458 ms / -			
P/QRS/T axis:	70° / 71° / 73°			
Heartrate:	63 bpm			

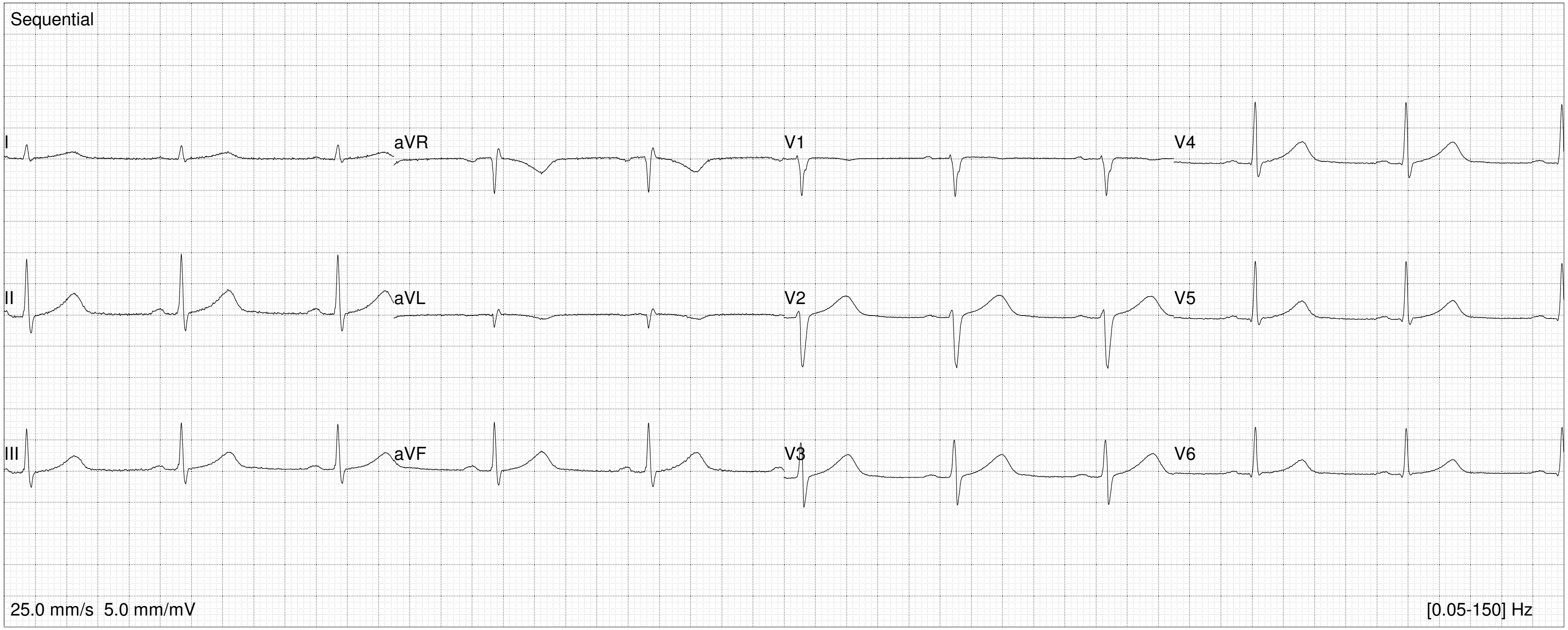

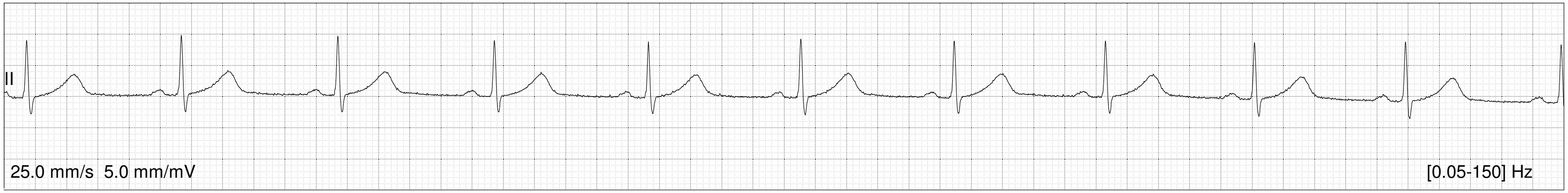

Anton Swart Biokinetic Rehabilitation Practice

Name:	008 008 008	Recorded:	06/05/2018 08:24:47	UNCONFIRMED INTERPRETATION - MD SHOULD REVIEW
Number:	008	Recorded by:	Mr. Anton Swart	
Gender:	Male	Referring physician:		
Birthdate:	13/12/1957 60 years	Location:	Anton Swart Biokinetic Rehabilitation Practice	
P / PQ:	105 ms / 160 ms	Ordering physician:		
QRS:	87 ms	Attending physician:		
QT / QTc / QTd:	453 ms / 458 ms / -	Comment:		
P/QRS/T axis:	70° / 71° / 73°			
Heartrate:	63 bpm			

**Beats**		**RR**	
Total:	312	Minimum	470 ms
Normal:	312	Maximum	1200 ms
Other:	0	Mean:	957 ms
		SD:	73 ms

R-R Trend

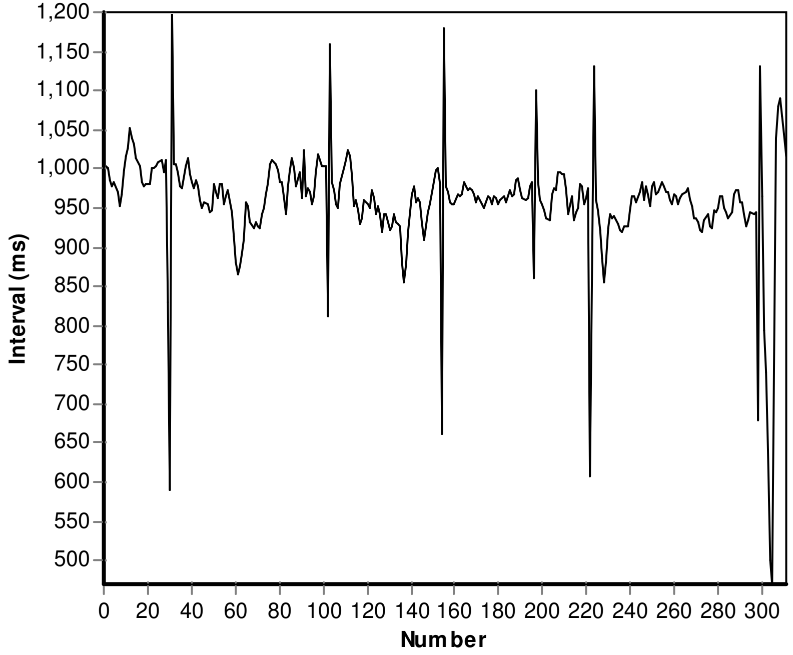

R-R Histogram

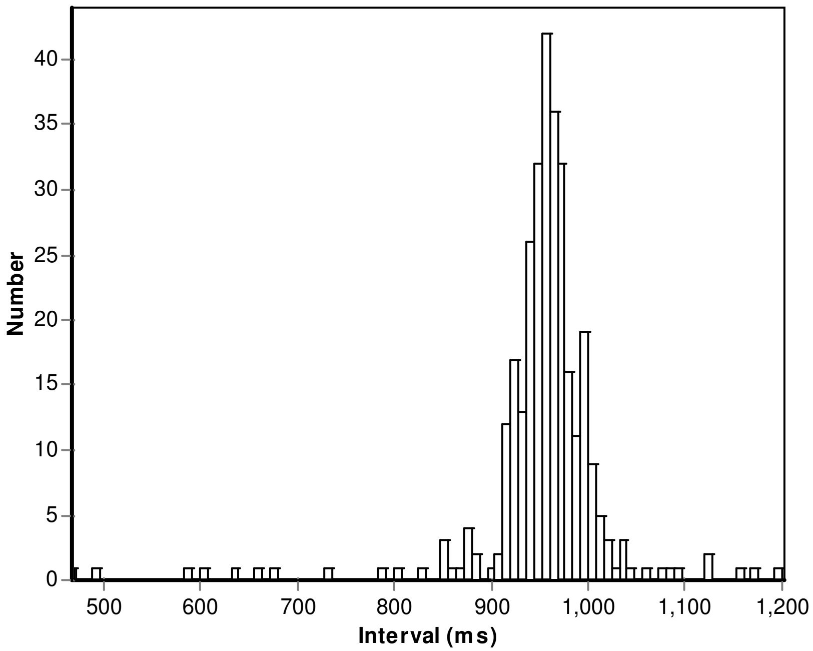

Heart Rate Variability: Time Domain Analysis

**Name:**	008, 008 008	**Birthdate:**	13/12/1957
**Number:**	008	**Recorded:**	06/05/2018 08:24:47
**Gender:**	Male		

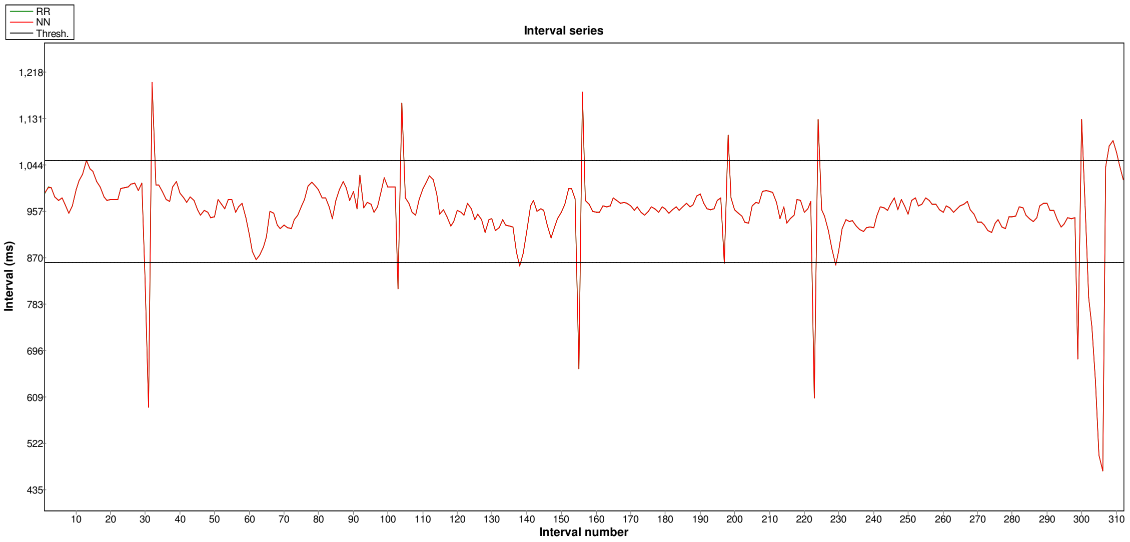

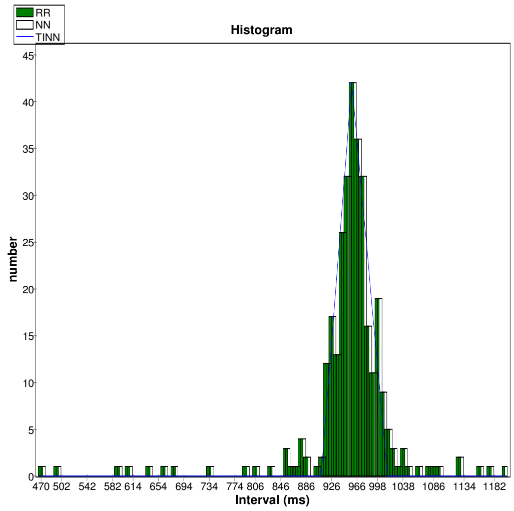

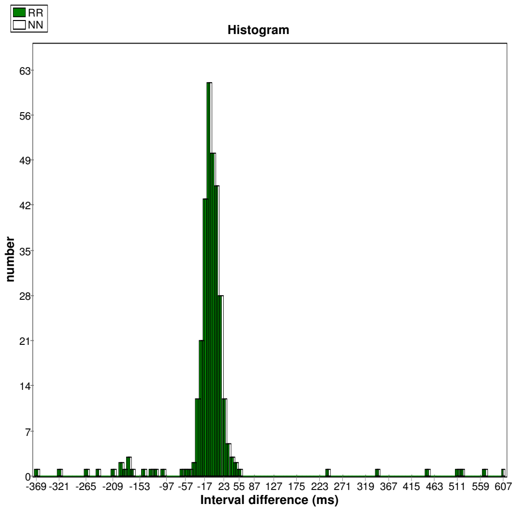

Binsize (ms) = 8

**HRV parameters**	**NN**	**RR**
SDNN (ms)	73	73
Triangular Interpolation (ms)	104	104
Triangular Index	7.43	7.43

**HRV parameters**	**NN**	**RR**
SDSD (ms)	88	88
RMSSD (ms)	88	88
NN50	26	26
NN50(1)	18	18
NN50(2)	8	8
pNN50	0.08	0.08
pNN50(1)	0.06	0.06
pNN50(2)	0.03	0.03
Logarithmic Index	0.13	0.13
SD(Logarithmic Index)	0.02	0.02

**Interval statistics**	**NN**	**RR**
Number	312	312
Minimum (ms)	470	470
Maximum (ms)	1200	1200
Range (ms)	730	730
Avg (ms)	957	957
SD (ms)	73	73
AvgDev (ms)	38	38
p5 (ms)	871	871
p50 (ms)	965	965
p95 (ms)	1026	1026
Skewness	-2.90	-2.90
Kurtosis	19.67	19.67

**Interval statistics**	**NN**	**RR**
Number	311	311
Minimum (ms)	-369	-369
Maximum (ms)	610	610
Range (ms)	979	979
Avg (ms)	0	0
SD (ms)	88	88
AvgDev (ms)	33	33
p5 (ms)	-100	-100
p50 (ms)	-2	-2
p95 (ms)	33	33
Skewness	3.20	3.20
Kurtosis	27.02	27.02

Cardio Control NV. User: Anton

Heart Rate Variability: Frequency Domain Analysis

**Name:**	008, 008 008	**Birthdate:**	13/12/1957
**Number:**	008	**Recorded:**	06/05/2018 08:24:47
**Gender:**	Male		

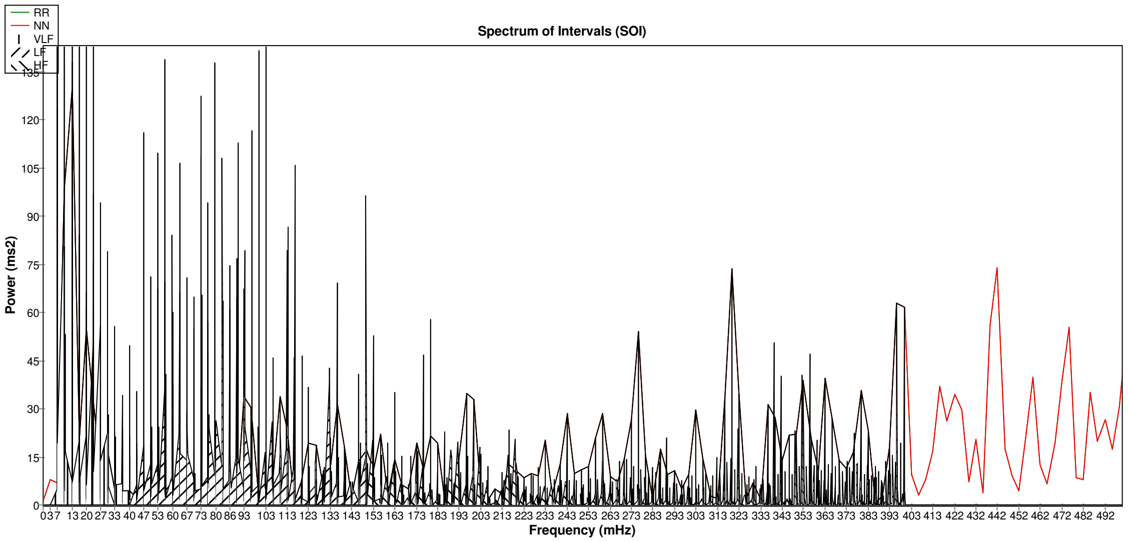

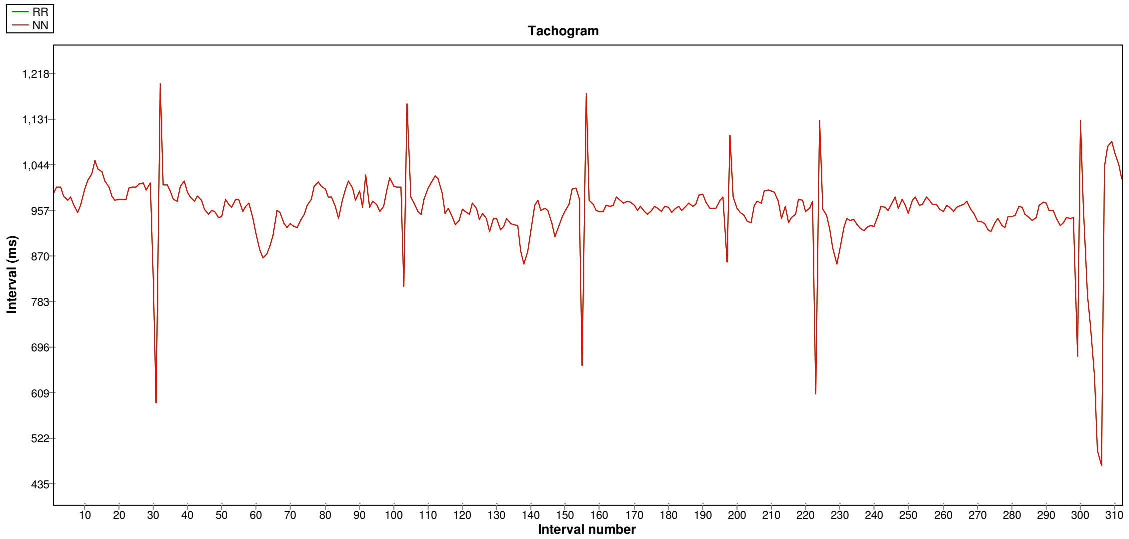

**HRV parameters**	**NN**	**RR**
TP (ms2)	2031	2031
VLF (ms2)	386	386
LF (ms2)	336	336
HF (ms2)	1309	1309
LF/HF	0.26	0.26
LF normalized	20.44	20.44
HF normalized	79.56	79.56
VLF peak (mHz)	13	13
LF peak (mHz)	93	93
HF peak (mHz)	319	319

**HRV spectral settings**	
Spectrum of Intervals (SOI)	
Frequency resolution (mHz)	3
VLF lower boundary (mHz)	3
VLF upper boundary (mHz)	40
LF upper boundary (mHz)	150
HF upper boundary (mHz)	400
Smoothing factor	1
Tapering	Hann
Fourier transform	DFT
Sample frequency (Hz)	1.04
Interval correction	Annotation
Interval threshold (%)	10
