# Supplementary material for: The effects of a 3-day mountain bike cycling race on the autonomic nervous system (ANS) and heart rate variability in amateur cyclists: a prospective quantitative research design
Source: BMC Sports Sci Med Rehabil. 2023 Jan 2;15:2. doi: 10.1186/s13102-022-00614-y (PMC9808932; doi:10.1186/s13102-022-00614-y)
Supplement: Supplementary file 1 — Additional file 1. Individual data of Participants. [file 13102_2022_614_MOESM1_ESM.zip › Individual data of Participants/HRV Data/009/ECG_009_20180501102045_.PDF]

Anton Swart Biokinetic Rehabilitation Practice

Name:	009 009 009	Recorded:	01/05/2018 10:20:45	UNCONFIRMED INTERPRETATION - MD SHOULD REVIEW
Number:	009	Recorded by:	Mr. Anton Swart	
Gender:	Female	Referring physician:		
Birthdate:	21/01/1958 60 years	Ordering physician:		
		Attending physician:		
		Location:	Anton Swart Biokinetic Rehabilitation Practi	
		Comment:		

P / PQ:	107 ms / 133 ms
QRS:	84 ms
QT / QTc / QTd:	467 ms / 446 ms / -
P/QRS/T axis:	59° / 83° / 69°
Heartrate:	48 bpm

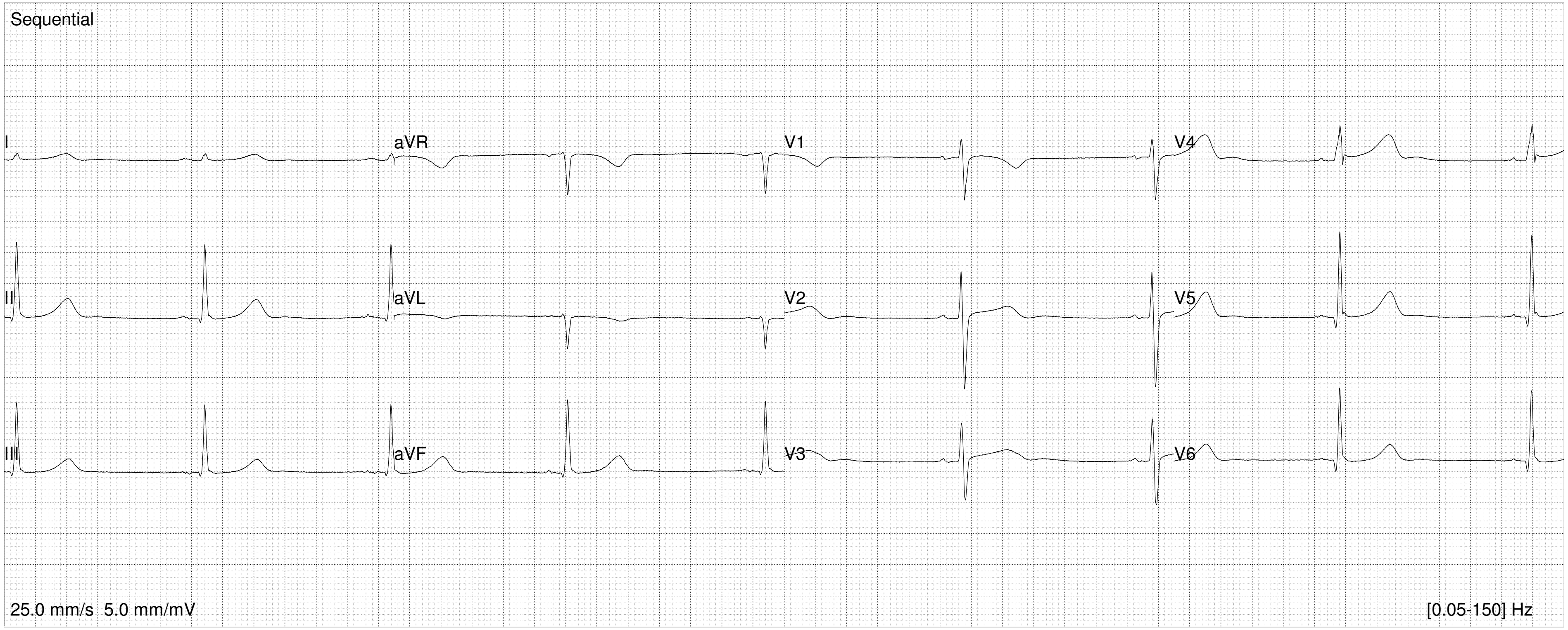

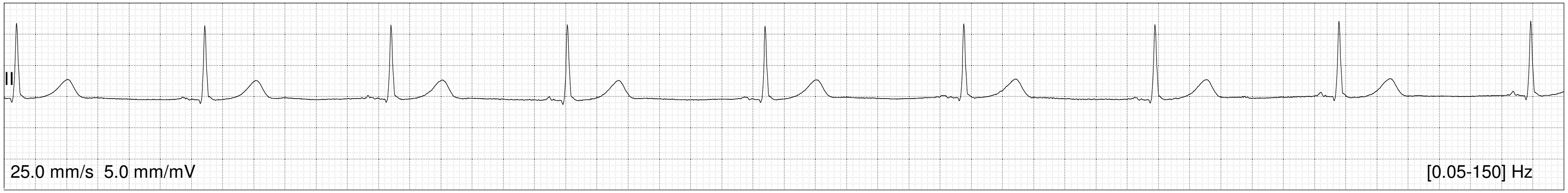

Anton Swart Biokinetic Rehabilitation Practice

Name:	009 009 009	Recorded:	01/05/2018 10:20:45	UNCONFIRMED INTERPRETATION - MD SHOULD REVIEW
Number:	009	Recorded by:	Mr. Anton Swart	
Gender:	Female	Referring physician:		
Birthdate:	21/01/1958 60 years	Location:	Anton Swart Biokinetic Rehabilitation Practice	
		Ordering physician:		
		Attending physician:		
		Comment:		

P / PQ:	107 ms / 133 ms
QRS:	84 ms
QT / QTc / QTd:	467 ms / 446 ms / -
P/QRS/T axis:	59° / 83° / 69°
Heartrate:	48 bpm

**Beats**		**RR**	
Total:	236	Minimum	1113 ms
Normal:	236	Maximum	1458 ms
Other:	0	Mean:	1268 ms
		SD:	75 ms

R-R Trend

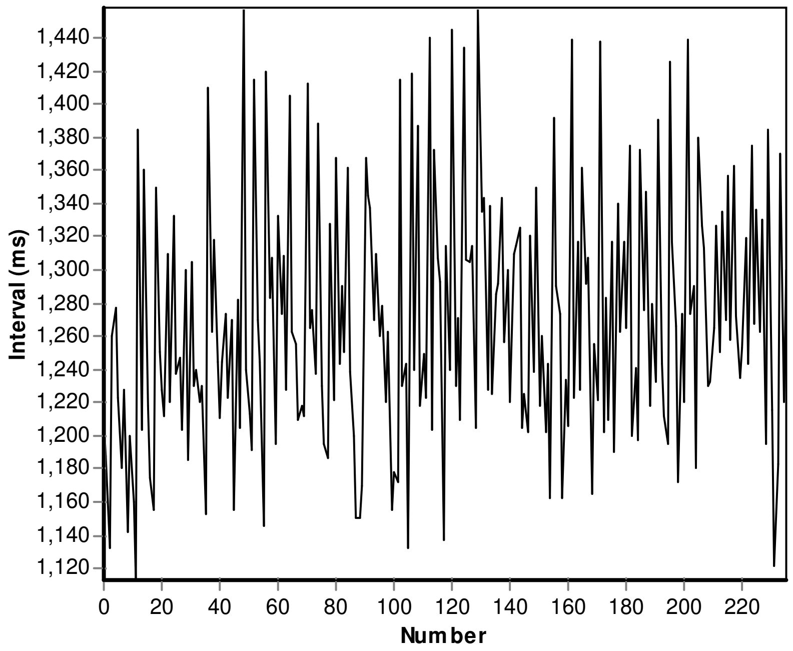

R-R Histogram

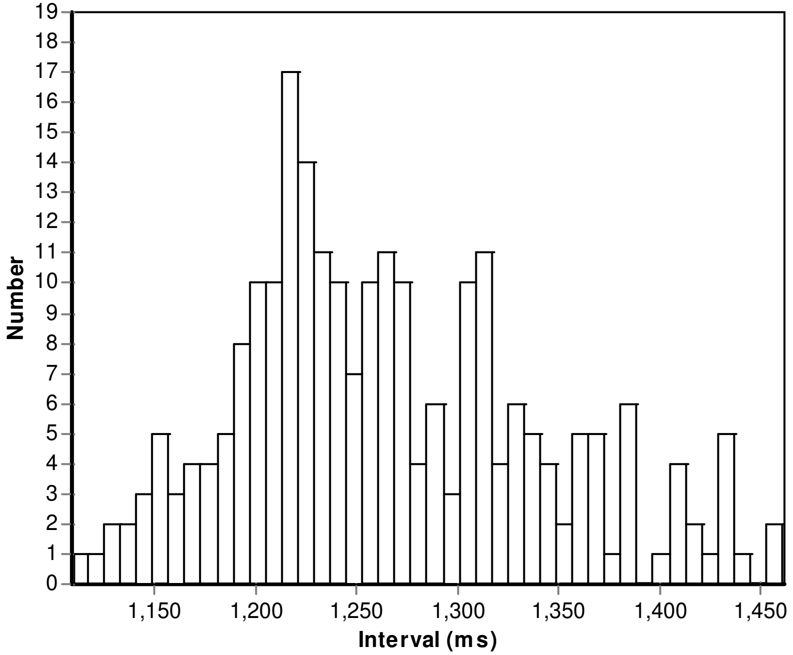

Cardio Control NV. User: Anton

Heart Rate Variability: Time Domain Analysis

**Name:**	009, 009 009	**Birthdate:**	21/01/1958
**Number:**	009	**Recorded:**	01/05/2018 10:20:45
**Gender:**	Female		

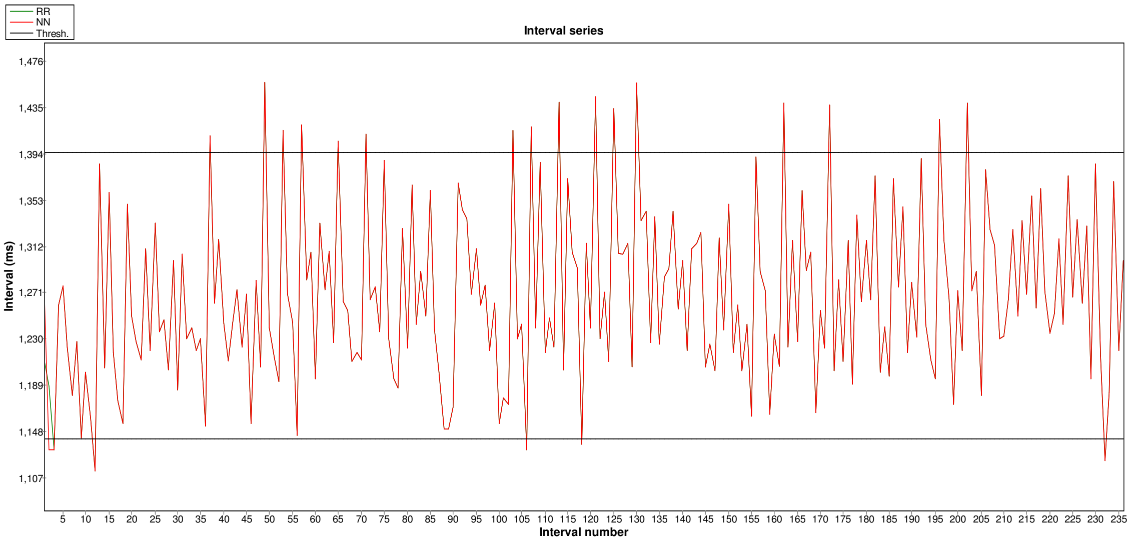

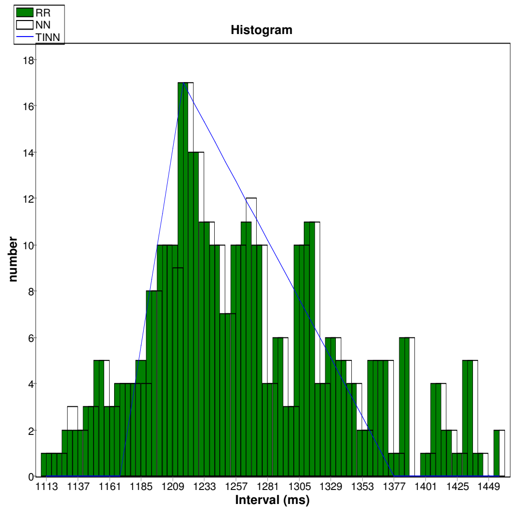

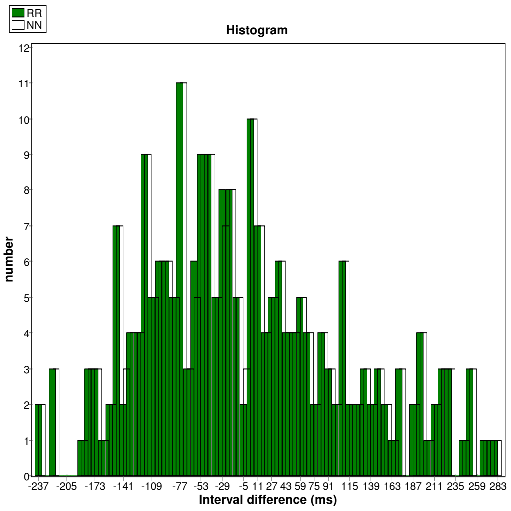

Binsize (ms) = 8

**HRV parameters**	**NN**	**RR**
SDNN (ms)	75	75
Triangular Interpolation (ms)	208	216
Triangular Index	13.88	13.88

**Interval statistics**	**NN**	**RR**
Number	236	236
Minimum (ms)	1113	1113
Maximum (ms)	1458	1458
Range (ms)	345	345
Avg (ms)	1268	1268
SD (ms)	75	75
AvgDev (ms)	61	61
p5 (ms)	1154	1155
p50 (ms)	1259	1258
p95 (ms)	1417	1417
Skewness	0.46	0.49
Kurtosis	2.69	2.69

**HRV parameters**	**NN**	**RR**
SDSD (ms)	115	115
RMSSD (ms)	115	115
NN50	158	158
NN50(1)	88	88
NN50(2)	70	70
pNN50	0.67	0.67
pNN50(1)	0.37	0.37
pNN50(2)	0.30	0.30
Logarithmic Index	0.07	0.07
SD(Logarithmic Index)	0.01	0.01

**Interval statistics**	**NN**	**RR**
Number	235	235
Minimum (ms)	-237	-237
Maximum (ms)	286	286
Range (ms)	523	523
Avg (ms)	0	0
SD (ms)	115	115
AvgDev (ms)	94	93
p5 (ms)	-166	-166
p50 (ms)	-17	-17
p95 (ms)	224	224
Skewness	0.45	0.45
Kurtosis	2.58	2.60

Cardio Control NV. User: Anton

Heart Rate Variability: Frequency Domain Analysis

**Name:**	009, 009 009	**Birthdate:**	21/01/1958
**Number:**	009	**Recorded:**	01/05/2018 10:20:45
**Gender:**	Female		

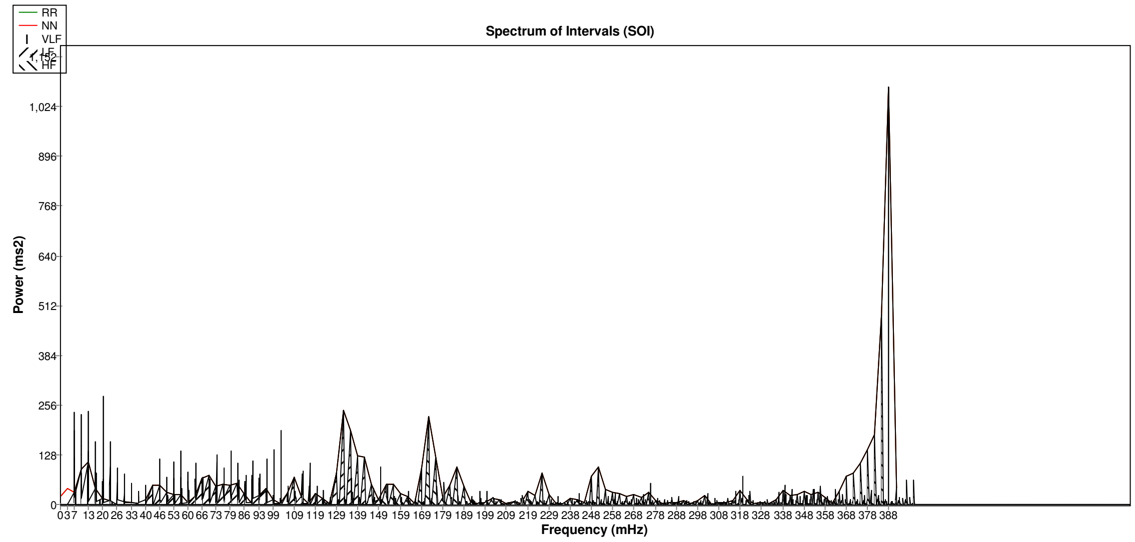

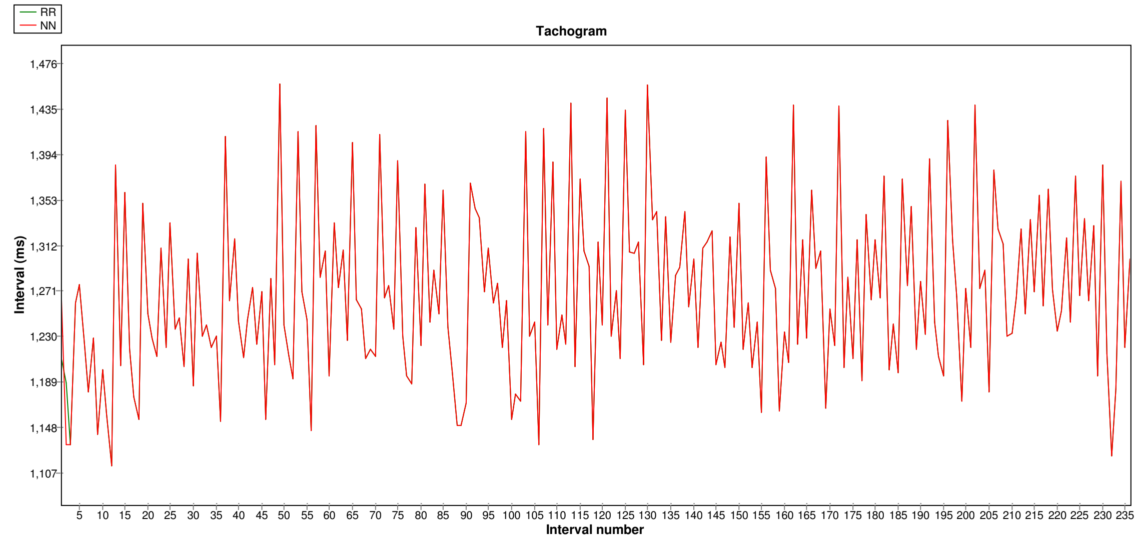

**HRV parameters**	**NN**	**RR**
TP (ms2)	6013	6013
VLF (ms2)	327	327
LF (ms2)	1667	1667
HF (ms2)	4019	4019
LF/HF	0.41	0.41
LF normalized	29.32	29.32
HF normalized	70.68	70.68
VLF peak (mHz)	13	13
LF peak (mHz)	132	132
HF peak (mHz)	388	388

**HRV spectral settings**	
Spectrum of Intervals (SOI)	
Frequency resolution (mHz)	3
VLF lower boundary (mHz)	3
VLF upper boundary (mHz)	40
LF upper boundary (mHz)	150
HF upper boundary (mHz)	400
Smoothing factor	1
Tapering	Hann
Fourier transform	DFT
Sample frequency (Hz)	0.79
Interval correction	Annotation
Interval threshold (%)	10
