# Supplementary material for: The effects of a 3-day mountain bike cycling race on the autonomic nervous system (ANS) and heart rate variability in amateur cyclists: a prospective quantitative research design
Source: BMC Sports Sci Med Rehabil. 2023 Jan 2;15:2. doi: 10.1186/s13102-022-00614-y (PMC9808932; doi:10.1186/s13102-022-00614-y)
Supplement: Supplementary file 1 — Additional file 1. Individual data of Participants. [file 13102_2022_614_MOESM1_ESM.zip › Individual data of Participants/HRV Data/009/ECG_009_20180503181558_.PDF]

Anton Swart Biokinetic Rehabilitation Practice

Name:	009 009 009	Recorded:	03/05/2018 18:15:58
Number:	009	Recorded by:	Mr. Anton Swart
Gender:	Female	Referring physician:	
Birthdate:	21/01/1958 60 years	Ordering physician:	
		Attending physician:	
P / PQ:	117 ms / 153 ms	Location:	Anton Swart Biokinetic Rehabilitation Practi
QRS:	82 ms	Comment:	
QT / QTc / QTd:	409 ms / 432 ms / -		
P/QRS/T axis:	73° / 82° / 62°		
Heartrate:	73 bpm		

UNCONFIRMED INTERPRETATION - MD SHOULD REVIEW

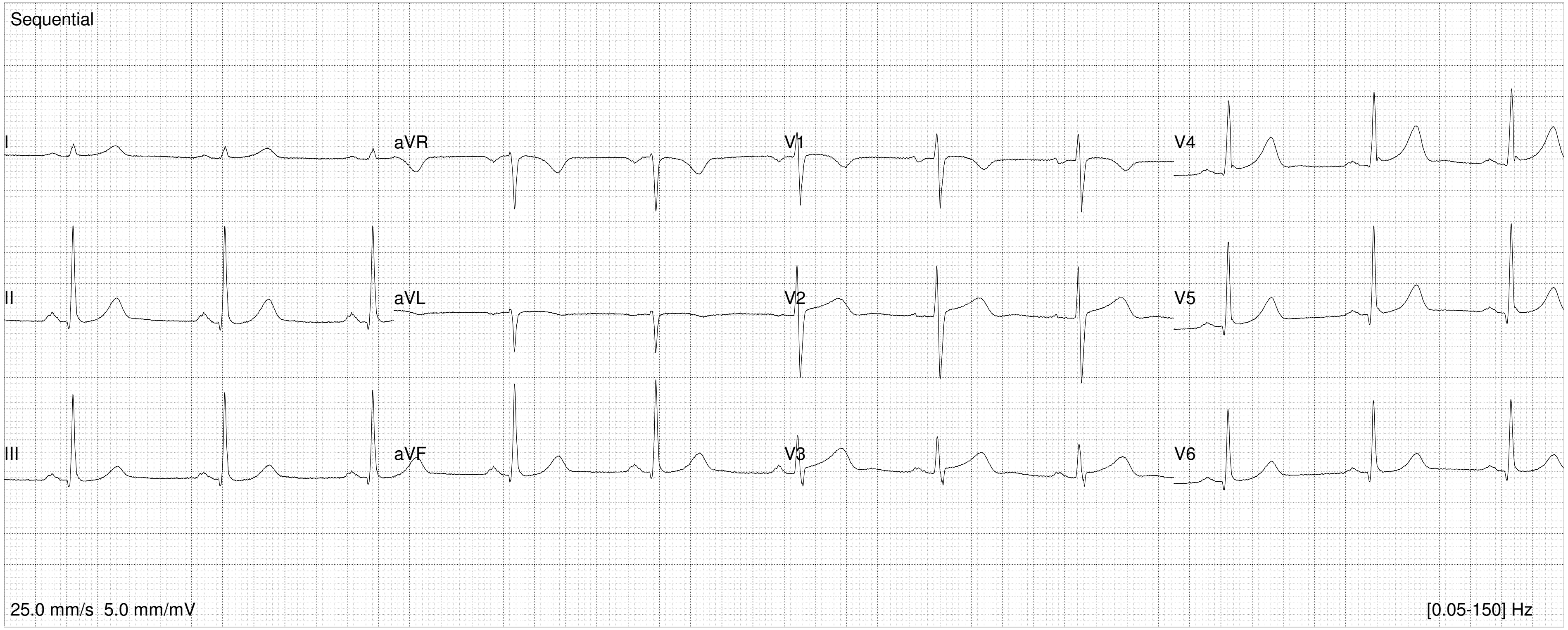

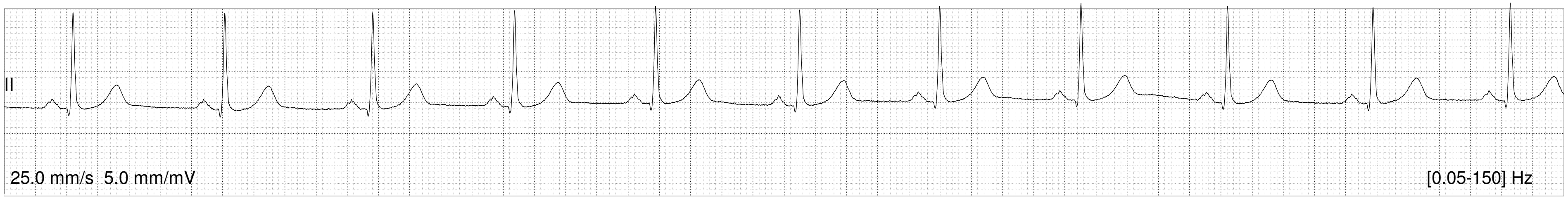

Anton Swart Biokinetic Rehabilitation Practice

Name:	009 009 009	Recorded:	03/05/2018 18:15:58	UNCONFIRMED INTERPRETATION - MD SHOULD REVIEW
Number:	009	Recorded by:	Mr. Anton Swart	
Gender:	Female	Referring physician:		
Birthdate:	21/01/1958 60 years	Location:	Anton Swart Biokinetic Rehabilitation Practice	
		Ordering physician:		
		Attending physician:		
		Comment:		

P / PQ:	117 ms / 153 ms
QRS:	82 ms
QT / QTc / QTd:	409 ms / 432 ms / -
P/QRS/T axis:	73° / 82° / 62°
Heartrate:	73 bpm

Beats

Total:	361
Normal:	361
Other:	0

RR

Minimum	760 ms
Maximum	970 ms
Mean:	829 ms
SD:	34 ms

R-R Trend

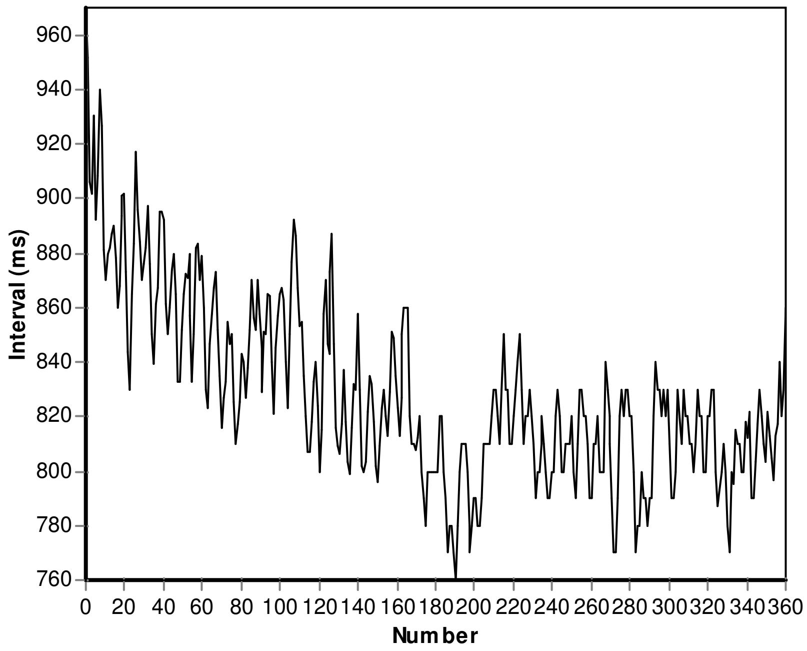

R-R Histogram

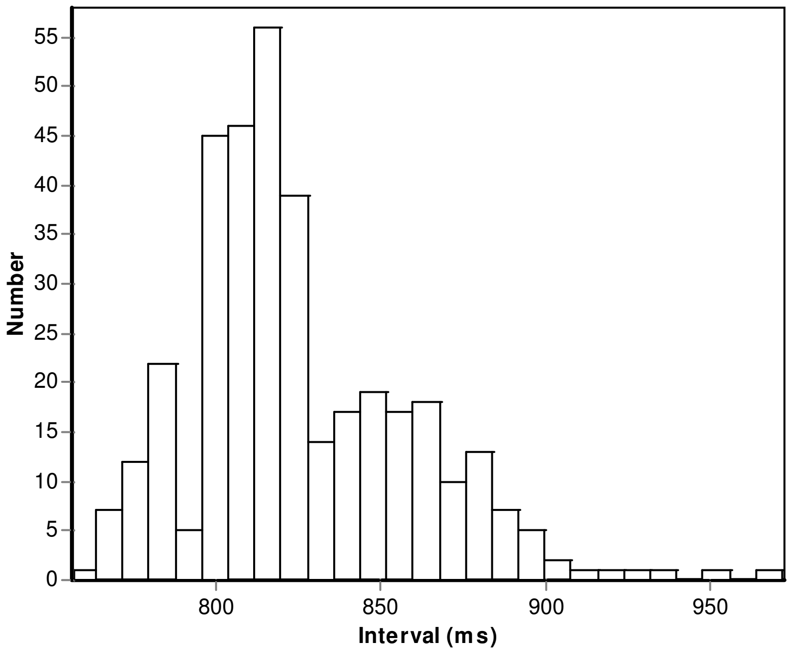

Cardio Control NV. User: Anton

Heart Rate Variability: Time Domain Analysis

**Name:**	009, 009 009	**Birthdate:**	21/01/1958
**Number:**	009	**Recorded:**	03/05/2018 18:15:58
**Gender:**	Female		

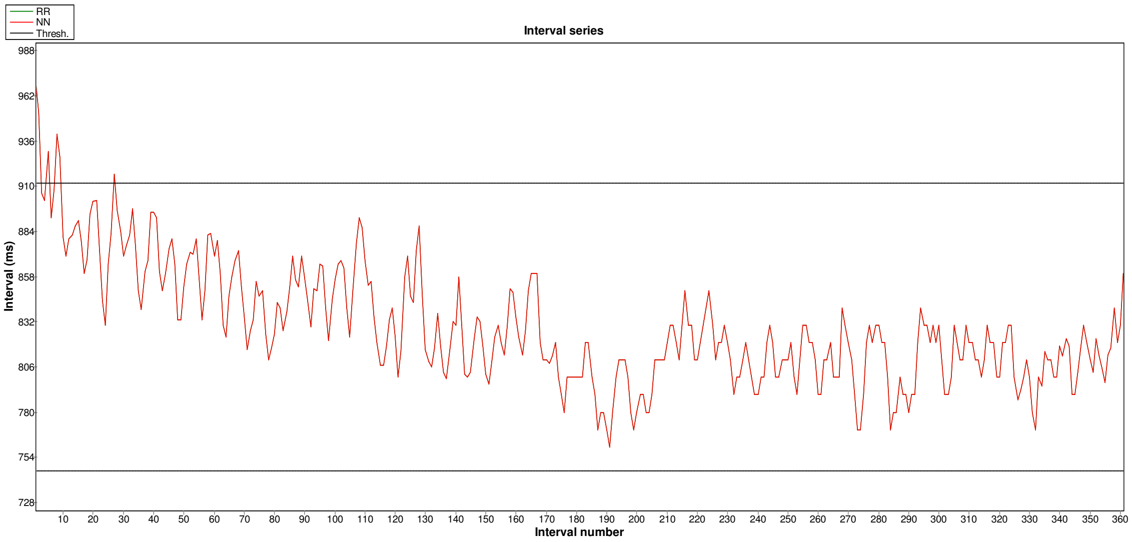

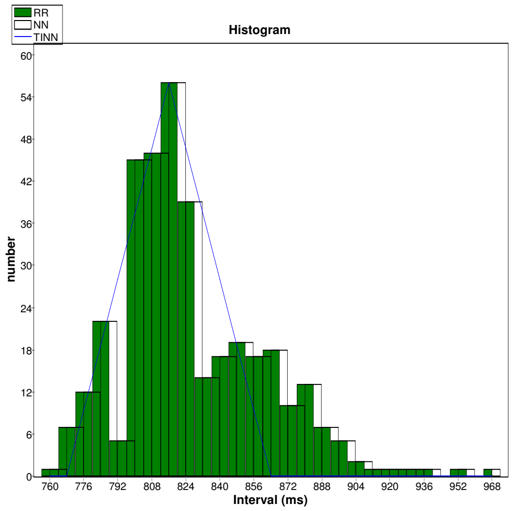

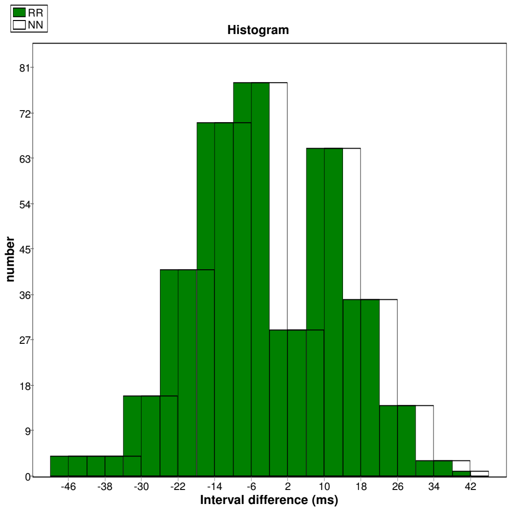

Binsize (ms) = 8

**HRV parameters**	**NN**	**RR**
SDNN (ms)	34	34
Triangular Interpolation (ms)	96	96
Triangular Index	6.45	6.45

**HRV parameters**	**NN**	**RR**
SDSD (ms)	16	16
RMSSD (ms)	16	16
NN50	0	0
NN50(1)	0	0
NN50(2)	0	0
pNN50	0.00	0.00
pNN50(1)	0.00	0.00
pNN50(2)	0.00	0.00
Logarithmic Index	0.70	0.70
SD(Logarithmic Index)	0.13	0.13

**Interval statistics**	**NN**	**RR**
Number	361	361
Minimum (ms)	760	760
Maximum (ms)	970	970
Range (ms)	210	210
Avg (ms)	829	829
SD (ms)	34	34
AvgDev (ms)	26	26
p5 (ms)	780	780
p50 (ms)	820	820
p95 (ms)	892	892
Skewness	0.87	0.87
Kurtosis	3.96	3.96

**Interval statistics**	**NN**	**RR**
Number	360	360
Minimum (ms)	-46	-46
Maximum (ms)	43	43
Range (ms)	89	89
Avg (ms)	-0	-0
SD (ms)	16	16
AvgDev (ms)	13	13
p5 (ms)	-25	-25
p50 (ms)	0	0
p95 (ms)	25	25
Skewness	-0.05	-0.05
Kurtosis	2.77	2.77

Cardio Control NV. User: Anton

Heart Rate Variability: Frequency Domain Analysis

**Name:**	009, 009 009	**Birthdate:**	21/01/1958
**Number:**	009	**Recorded:**	03/05/2018 18:15:58
**Gender:**	Female		

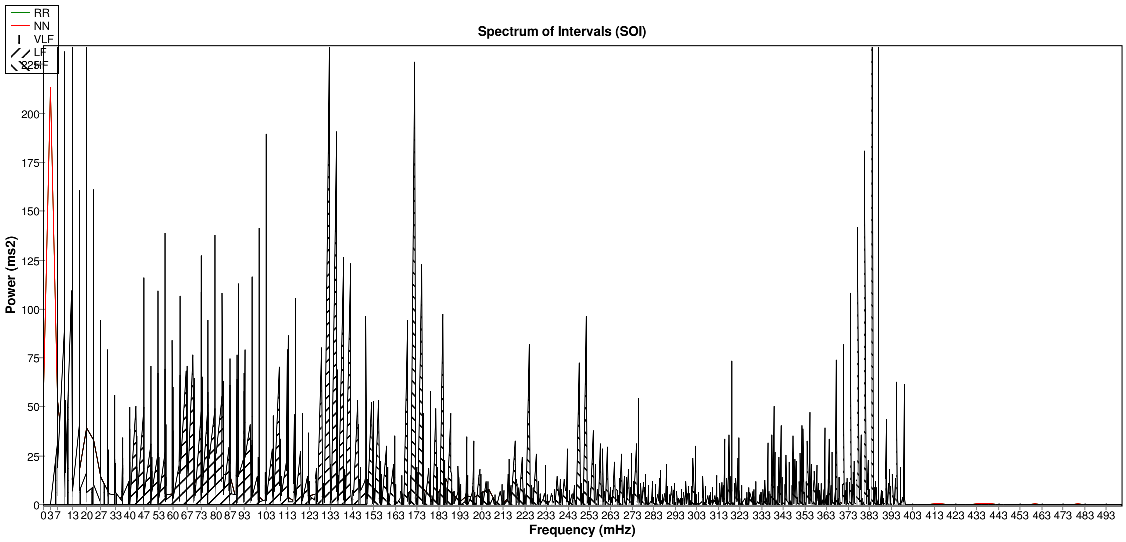

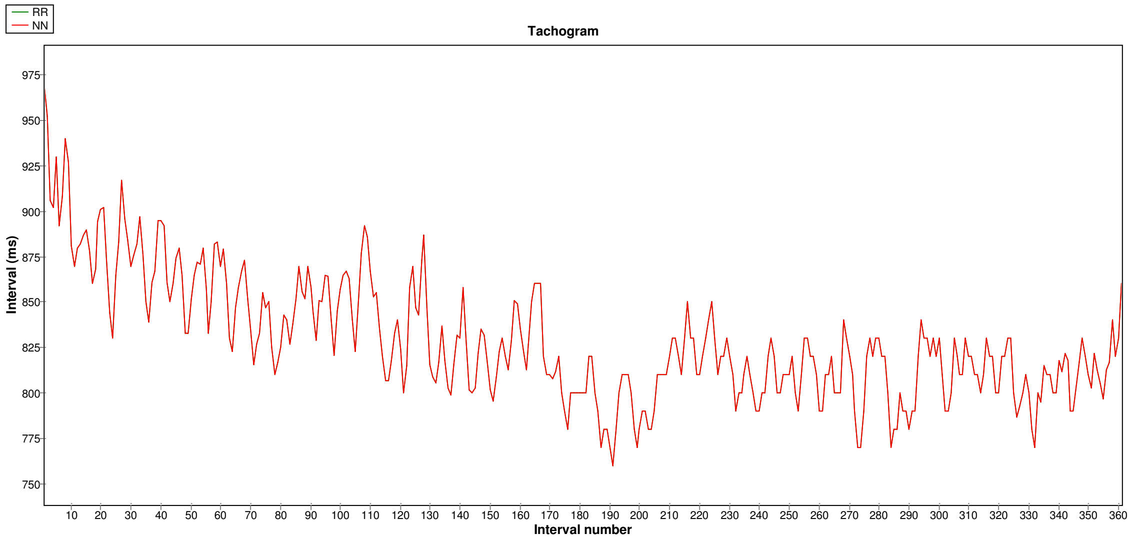

**HRV parameters**	**NN**	**RR**
TP (ms2)	477	477
VLF (ms2)	211	211
LF (ms2)	143	143
HF (ms2)	123	123
LF/HF	1.16	1.16
LF normalized	53.79	53.79
HF normalized	46.21	46.21
VLF peak (mHz)	7	7
LF peak (mHz)	83	83
HF peak (mHz)	187	187

**HRV spectral settings**	
Spectrum of Intervals (SOI)	
Frequency resolution (mHz)	3
VLF lower boundary (mHz)	3
VLF upper boundary (mHz)	40
LF upper boundary (mHz)	150
HF upper boundary (mHz)	400
Smoothing factor	1
Tapering	Hann
Fourier transform	DFT
Sample frequency (Hz)	1.21
Interval correction	Annotation
Interval threshold (%)	10
