# Supplementary material for: The effects of a 3-day mountain bike cycling race on the autonomic nervous system (ANS) and heart rate variability in amateur cyclists: a prospective quantitative research design
Source: BMC Sports Sci Med Rehabil. 2023 Jan 2;15:2. doi: 10.1186/s13102-022-00614-y (PMC9808932; doi:10.1186/s13102-022-00614-y)
Supplement: Supplementary file 1 — Additional file 1. Individual data of Participants. [file 13102_2022_614_MOESM1_ESM.zip › Individual data of Participants/HRV Data/009/ECG_009_20180504142711_.PDF]

Anton Swart Biokinetic Rehabilitation Practice

Name:	009 009 009	Recorded:	04/05/2018 14:27:11	UNCONFIRMED INTERPRETATION - MD SHOULD REVIEW
Number:	009	Recorded by:	Mr. Anton Swart	
Gender:	Female	Referring physician:		
Birthdate:	21/01/1958 60 years	Ordering physician:		
		Attending physician:		
		Location:	Anton Swart Biokinetic Rehabilitation Practi	
		Comment:		

P / PQ:	108 ms / 140 ms
QRS:	83 ms
QT / QTc / QTd:	383 ms / 430 ms / -
P/QRS/T axis:	78° / 84° / 71°
Heartrate:	87 bpm

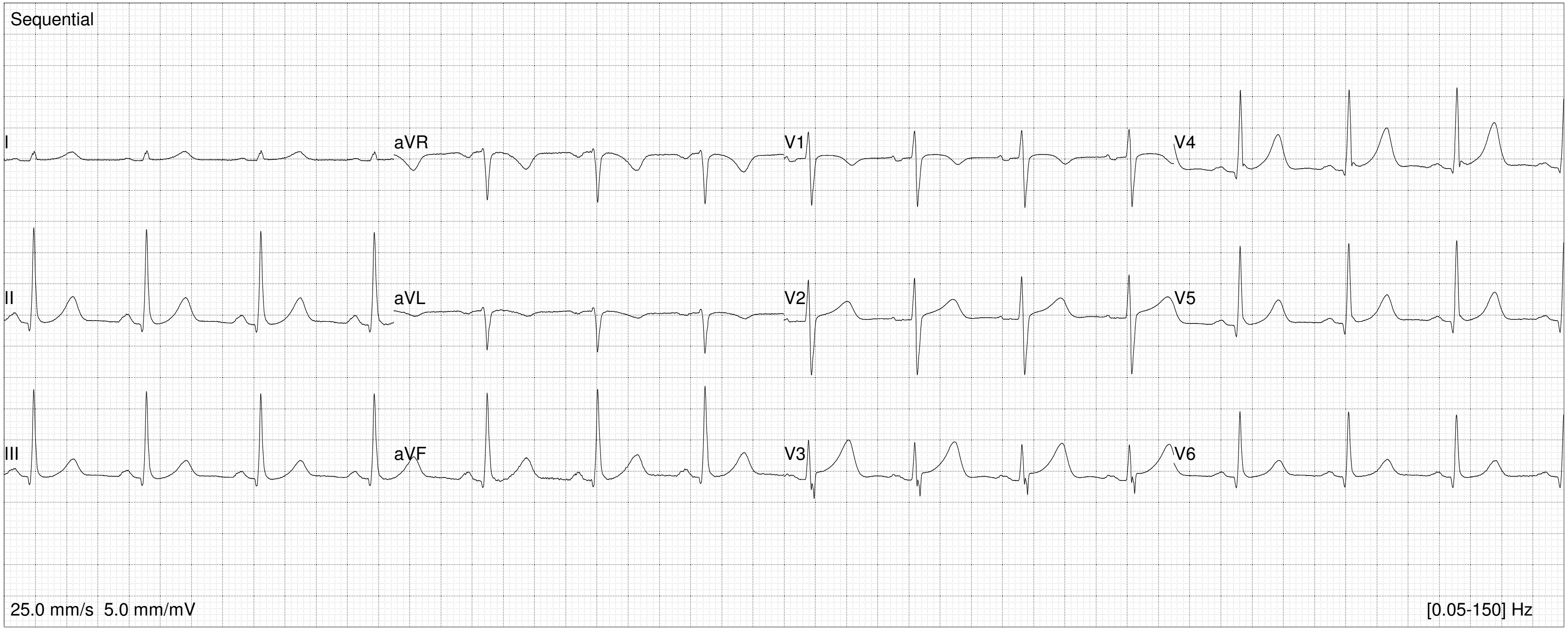

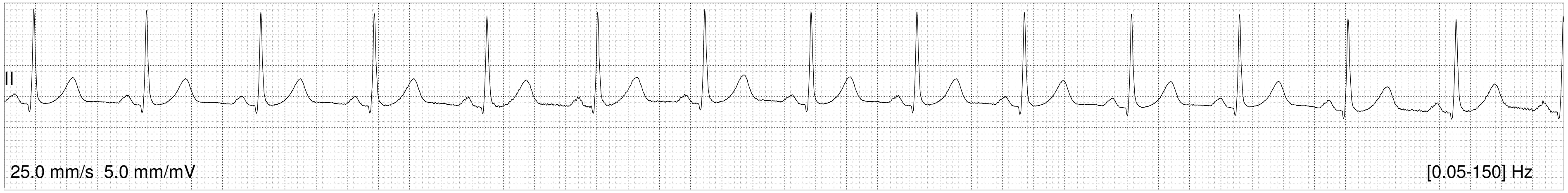

Anton Swart Biokinetic Rehabilitation Practice

Name:	009 009 009	Recorded:	04/05/2018 14:27:11	UNCONFIRMED INTERPRETATION - MD SHOULD REVIEW
Number:	009	Recorded by:	Mr. Anton Swart	
Gender:	Female	Referring physician:		
Birthdate:	21/01/1958 60 years	Location:	Anton Swart Biokinetic Rehabilitation Practice	
P / PQ:	108 ms / 140 ms	Ordering physician:		
QRS:	83 ms	Attending physician:		
QT / QTc / QTd:	383 ms / 430 ms / -	Comment:		
P/QRS/T axis:	78° / 84° / 71°			
Heartrate:	87 bpm			

Beats

Total:	430
Normal:	430
Other:	0

RR

Minimum	640 ms
Maximum	778 ms
Mean:	696 ms
SD:	22 ms

R-R Trend

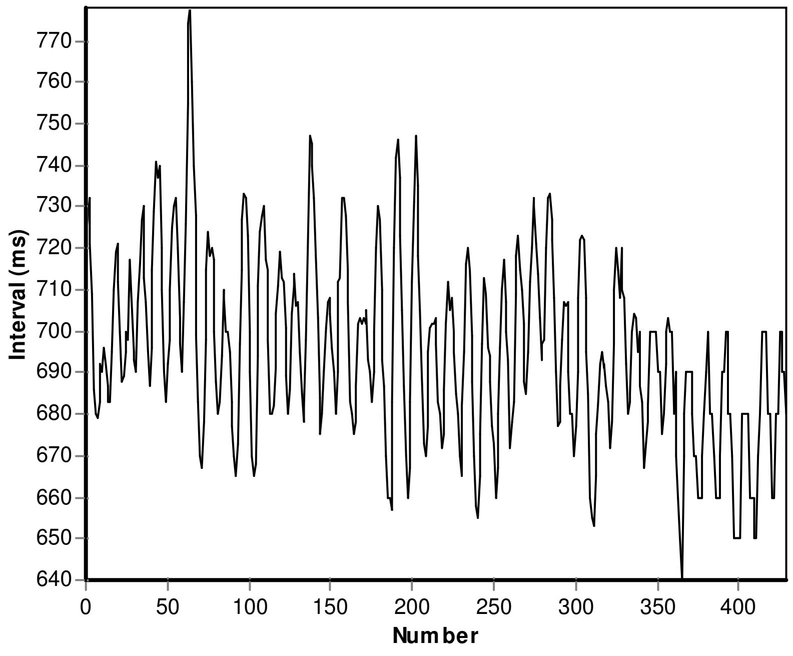

R-R Histogram

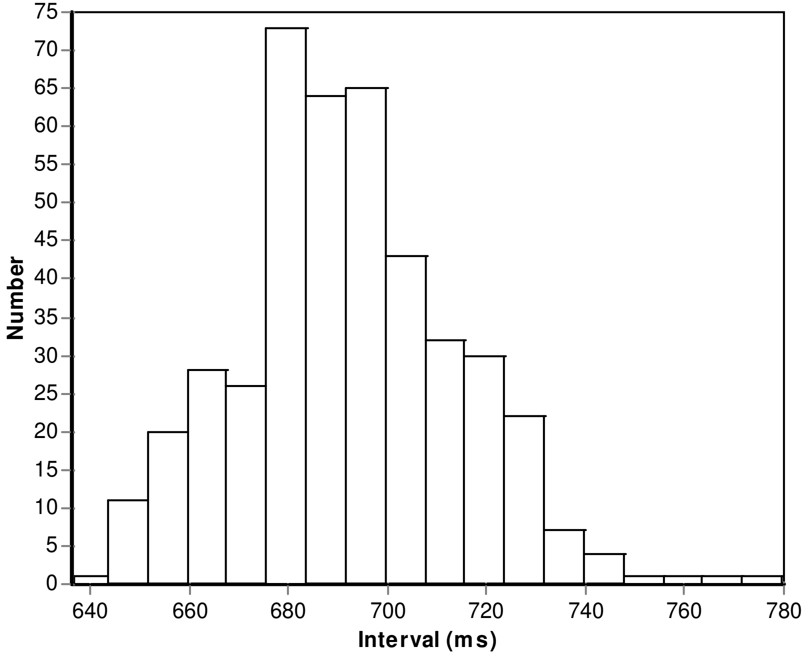

Heart Rate Variability: Time Domain Analysis

**Name:**	009, 009 009	**Birthdate:**	21/01/1958
**Number:**	009	**Recorded:**	04/05/2018 14:27:11
**Gender:**	Female		

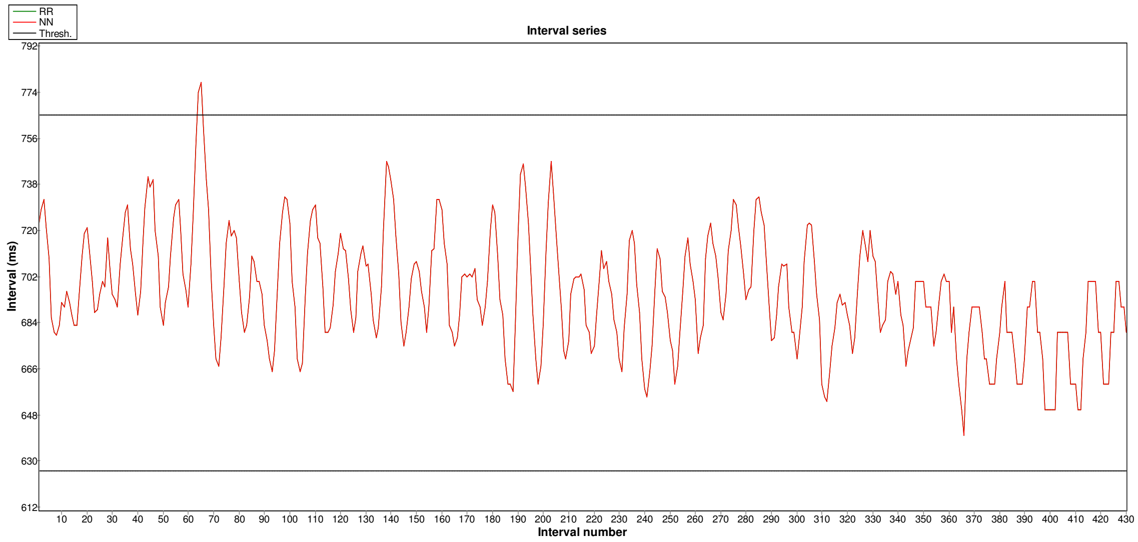

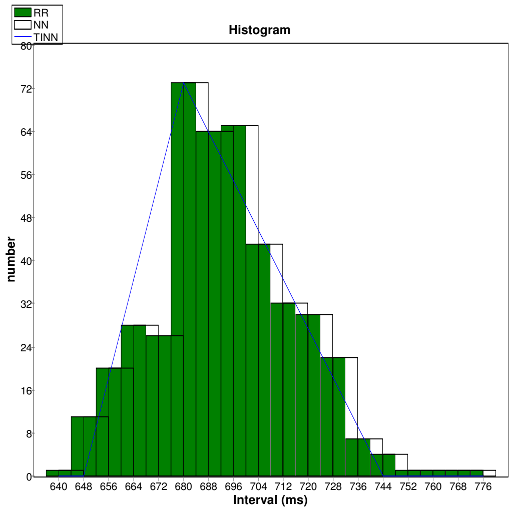

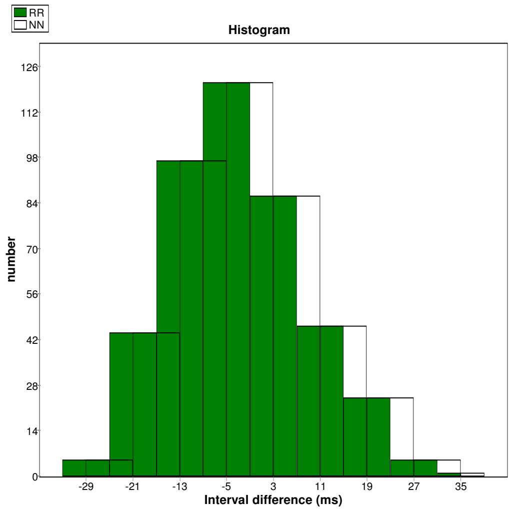

Binsize (ms) = 8

**HRV parameters**	**NN**	**RR**
SDNN (ms)	22	22
Triangular Interpolation (ms)	96	96
Triangular Index	5.89	5.89

**HRV parameters**	**NN**	**RR**
SDSD (ms)	12	12
RMSSD (ms)	12	12
NN50	0	0
NN50(1)	0	0
NN50(2)	0	0
pNN50	0.00	0.00
pNN50(1)	0.00	0.00
pNN50(2)	0.00	0.00
Logarithmic Index	1.30	1.30
SD(Logarithmic Index)	0.28	0.28

**Interval statistics**	**NN**	**RR**
Number	430	430
Minimum (ms)	640	640
Maximum (ms)	778	778
Range (ms)	138	138
Avg (ms)	696	696
SD (ms)	22	22
AvgDev (ms)	18	18
p5 (ms)	660	660
p50 (ms)	695	695
p95 (ms)	732	732
Skewness	0.32	0.32
Kurtosis	3.23	3.23

**Interval statistics**	**NN**	**RR**
Number	429	429
Minimum (ms)	-29	-29
Maximum (ms)	37	37
Range (ms)	66	66
Avg (ms)	-0	-0
SD (ms)	12	12
AvgDev (ms)	9	9
p5 (ms)	-18	-18
p50 (ms)	0	0
p95 (ms)	20	20
Skewness	0.28	0.28
Kurtosis	2.70	2.70

Heart Rate Variability: Frequency Domain Analysis

**Name:**	009, 009 009	**Birthdate:**	21/01/1958
**Number:**	009	**Recorded:**	04/05/2018 14:27:11
**Gender:**	Female		

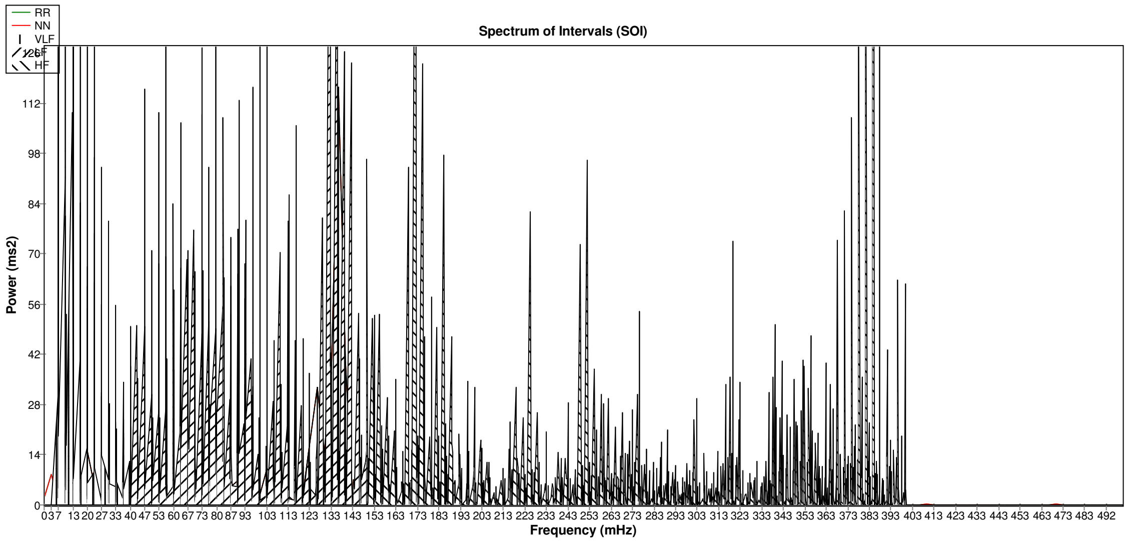

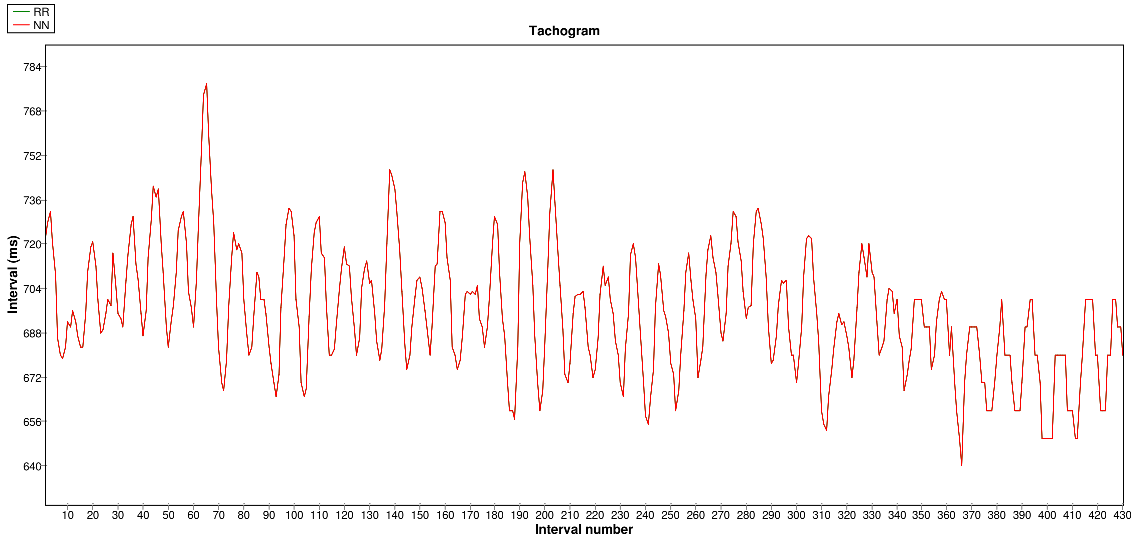

**HRV parameters**	**NN**	**RR**
TP (ms2)	425	425
VLF (ms2)	46	46
LF (ms2)	349	349
HF (ms2)	30	30
LF/HF	11.83	11.83
LF normalized	92.21	92.21
HF normalized	7.79	7.79
VLF peak (mHz)	20	20
LF peak (mHz)	136	136
HF peak (mHz)	156	156

**HRV spectral settings**	
Spectrum of Intervals (SOI)	
Frequency resolution (mHz)	3
VLF lower boundary (mHz)	3
VLF upper boundary (mHz)	40
LF upper boundary (mHz)	150
HF upper boundary (mHz)	400
Smoothing factor	1
Tapering	Hann
Fourier transform	DFT
Sample frequency (Hz)	1.44
Interval correction	Annotation
Interval threshold (%)	10
