# Supplementary material for: The effects of a 3-day mountain bike cycling race on the autonomic nervous system (ANS) and heart rate variability in amateur cyclists: a prospective quantitative research design
Source: BMC Sports Sci Med Rehabil. 2023 Jan 2;15:2. doi: 10.1186/s13102-022-00614-y (PMC9808932; doi:10.1186/s13102-022-00614-y)
Supplement: Supplementary file 1 — Additional file 1. Individual data of Participants. [file 13102_2022_614_MOESM1_ESM.zip › Individual data of Participants/HRV Data/009/ECG_009_20180505142424_.PDF]

Anton Swart Biokinetic Rehabilitation Practice

Name:	009 009 009	Recorded:	05/05/2018 14:24:24	UNCONFIRMED INTERPRETATION - MD SHOULD REVIEW
Number:	009	Recorded by:	Mr. Anton Swart	
Gender:	Female	Referring physician:		
Birthdate:	21/01/1958 60 years	Ordering physician:		
		Attending physician:		
P / PQ:	113 ms / 147 ms	Location:	Anton Swart Biokinetic Rehabilitation Practi	
QRS:	81 ms	Comment:		
QT / QTc / QTd:	393 ms / 428 ms / -			
P/QRS/T axis:	78° / 83° / 71°			
Heartrate:	80 bpm			

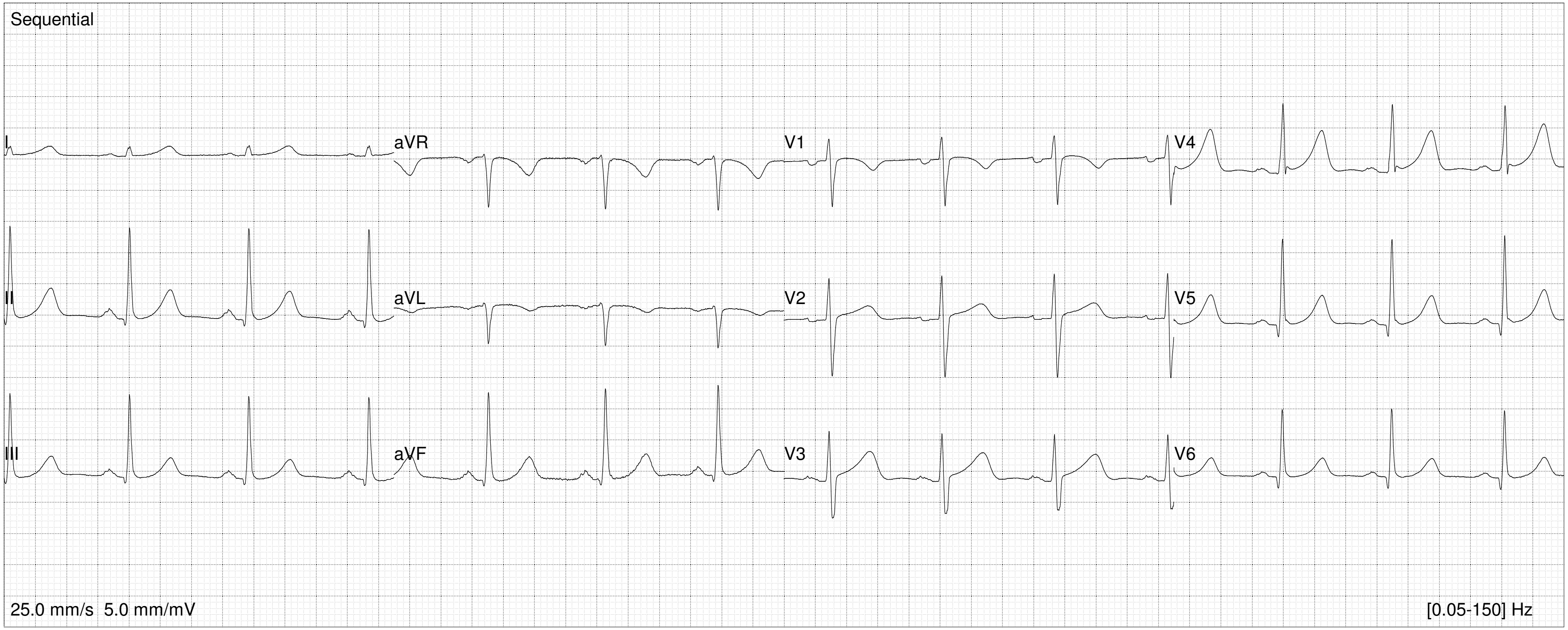

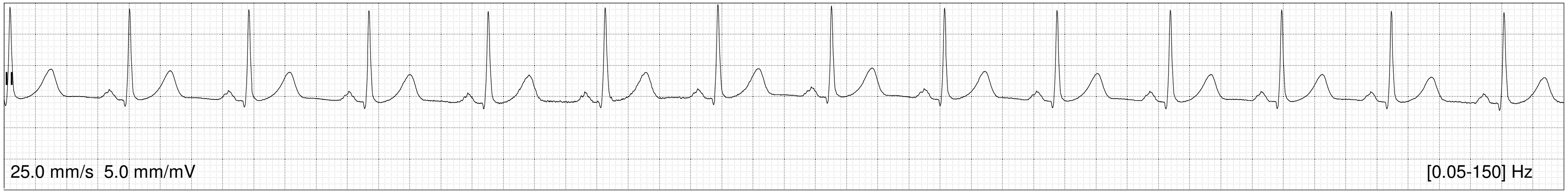

Anton Swart Biokinetic Rehabilitation Practice

Name:	009 009 009	Recorded:	05/05/2018 14:24:24	UNCONFIRMED INTERPRETATION - MD SHOULD REVIEW
Number:	009	Recorded by:	Mr. Anton Swart	
Gender:	Female	Referring physician:		
Birthdate:	21/01/1958 60 years	Location:	Anton Swart Biokinetic Rehabilitation Practice	
		Ordering physician:		
		Attending physician:		
		Comment:		

P / PQ:	113 ms / 147 ms
QRS:	81 ms
QT / QTc / QTd:	393 ms / 428 ms / -
P/QRS/T axis:	78° / 83° / 71°
Heartrate:	80 bpm

**Beats**		**RR**	
Total:	400	Minimum	697 ms
Normal:	400	Maximum	792 ms
Other:	0	Mean:	749 ms
		SD:	15 ms

R-R Trend

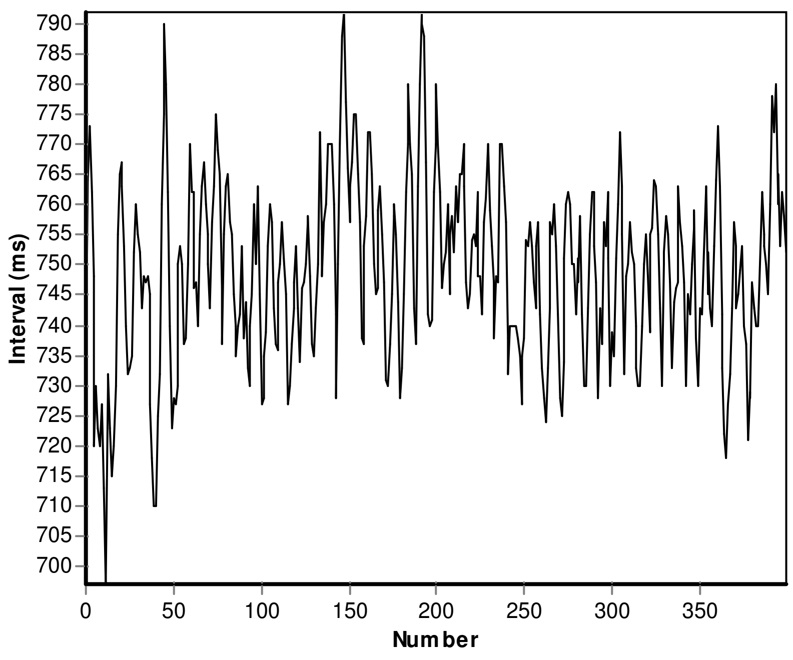

R-R Histogram

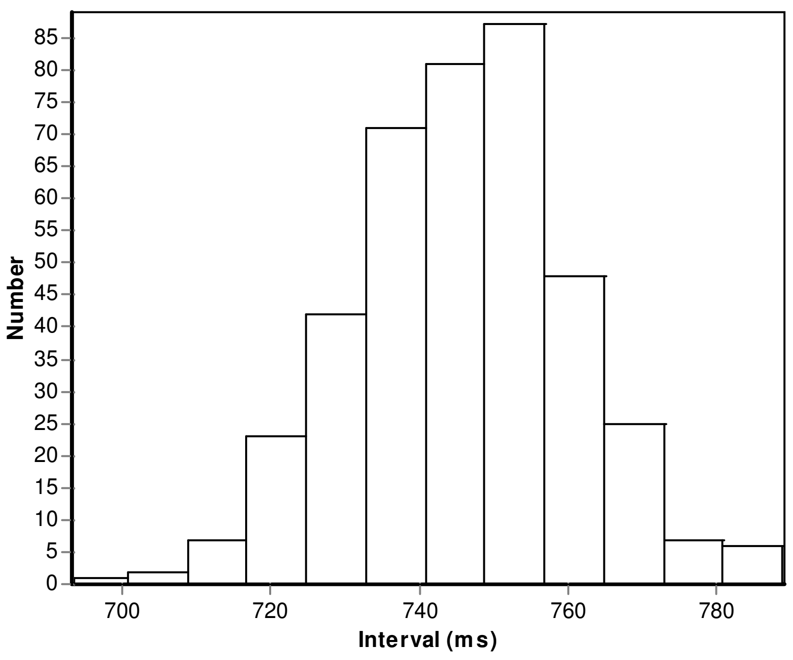

Cardio Control NV. User: Anton

Heart Rate Variability: Time Domain Analysis

**Name:**	009, 009 009	**Birthdate:**	21/01/1958
**Number:**	009	**Recorded:**	05/05/2018 14:24:24
**Gender:**	Female		

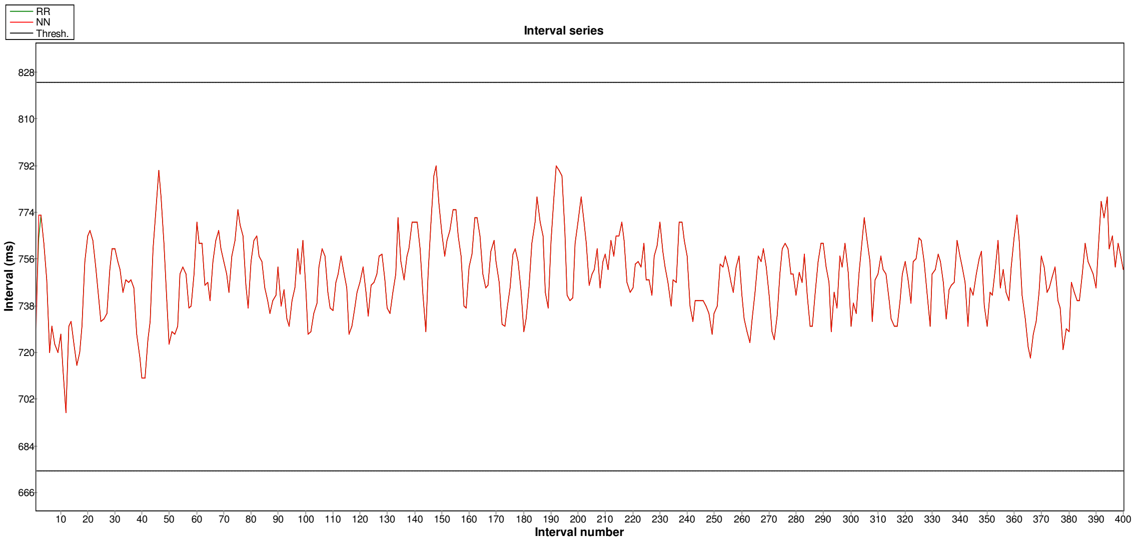

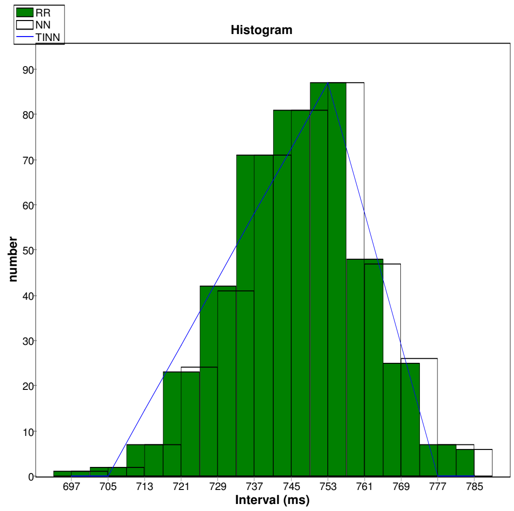

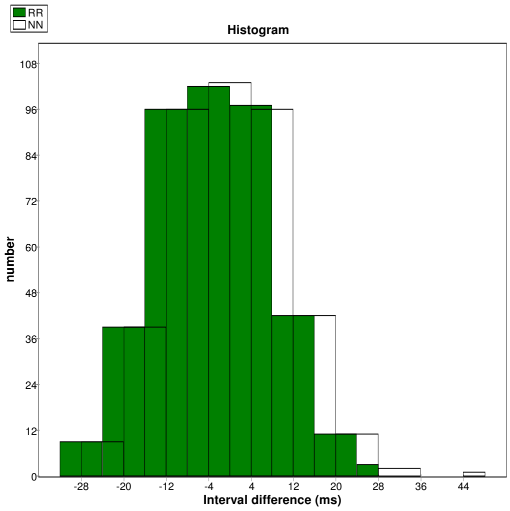

Binsize (ms) = 8

HRV parameters	NN	RR
SDNN (ms)	15	15
Triangular Interpolation (ms)	72	72
Triangular Index	4.60	4.60

HRV parameters	NN	RR
SDSD (ms)	11	11
RMSSD (ms)	11	11
NN50	0	0
NN50(1)	0	0
NN50(2)	0	0
pNN50	0.00	0.00
pNN50(1)	0.00	0.00
pNN50(2)	0.00	0.00
Logarithmic Index	1.25	1.37
SD(Logarithmic Index)	0.17	0.22

Interval statistics	NN	RR
Number	400	400
Minimum (ms)	697	697
Maximum (ms)	792	792
Range (ms)	95	95
Avg (ms)	749	749
SD (ms)	15	15
AvgDev (ms)	12	12
p5 (ms)	727	727
p50 (ms)	750	750
p95 (ms)	773	772
Skewness	-0.00	0.00
Kurtosis	3.24	3.26

Interval statistics	NN	RR
Number	399	399
Minimum (ms)	-28	-28
Maximum (ms)	49	33
Range (ms)	77	61
Avg (ms)	0	0
SD (ms)	11	11
AvgDev (ms)	9	9
p5 (ms)	-18	-18
p50 (ms)	0	0
p95 (ms)	17	17
Skewness	0.27	0.11
Kurtosis	3.43	2.70

Heart Rate Variability: Frequency Domain Analysis

**Name:**	009, 009 009	**Birthdate:**	21/01/1958
**Number:**	009	**Recorded:**	05/05/2018 14:24:24
**Gender:**	Female		

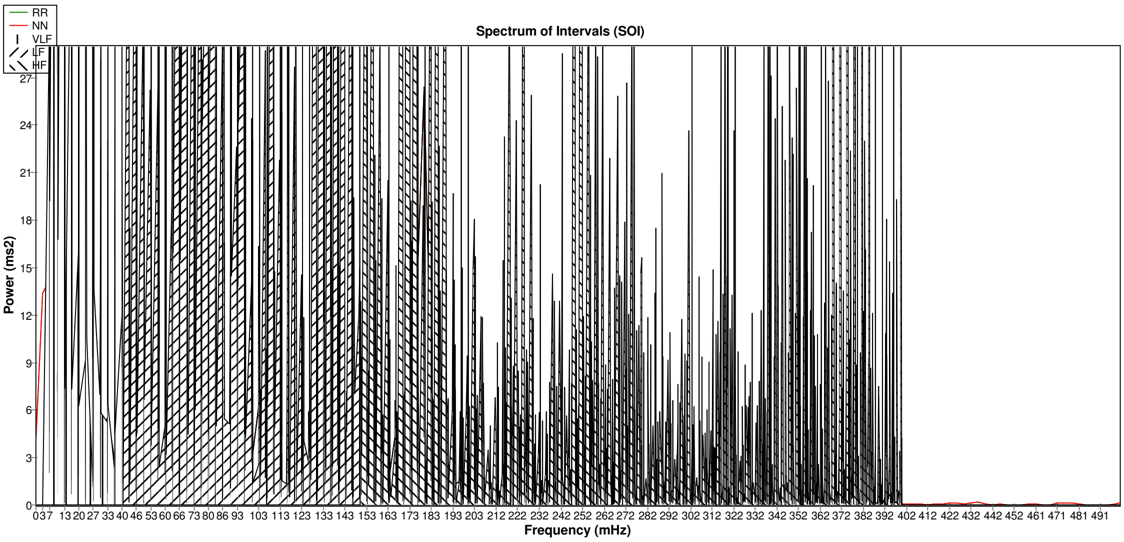

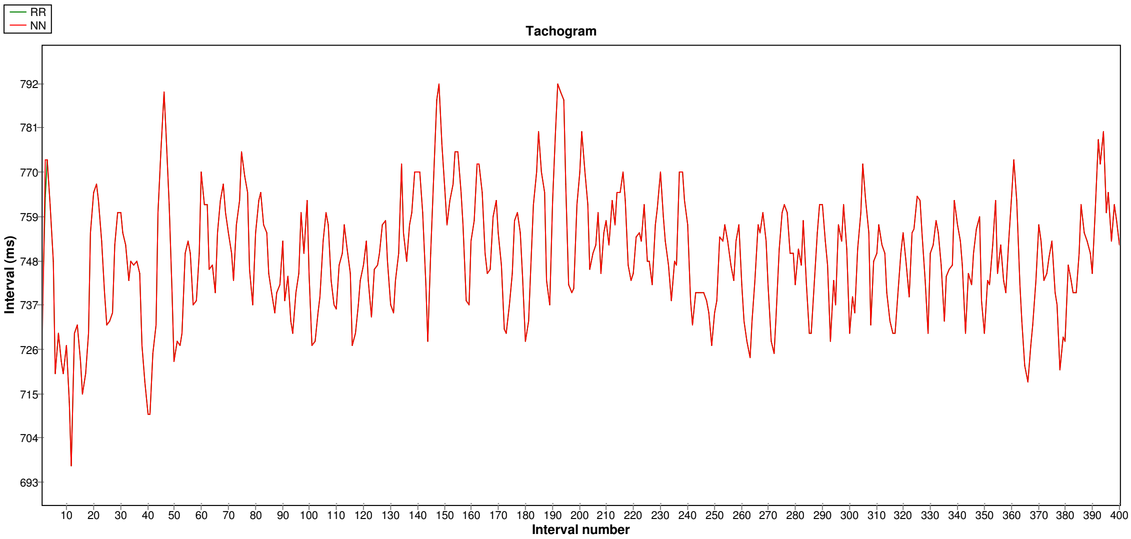

**HRV parameters**	**NN**	**RR**
TP (ms2)	190	190
VLF (ms2)	52	52
LF (ms2)	48	48
HF (ms2)	90	90
LF/HF	0.53	0.53
LF normalized	34.72	34.72
HF normalized	65.28	65.28
VLF peak (mHz)	7	7
LF peak (mHz)	103	103
HF peak (mHz)	179	179

**HRV spectral settings**	
Spectrum of Intervals (SOI)	
Frequency resolution (mHz)	3
VLF lower boundary (mHz)	3
VLF upper boundary (mHz)	40
LF upper boundary (mHz)	150
HF upper boundary (mHz)	400
Smoothing factor	1
Tapering	Hann
Fourier transform	DFT
Sample frequency (Hz)	1.33
Interval correction	Annotation
Interval threshold (%)	10
