# Supplementary material for: The effects of a 3-day mountain bike cycling race on the autonomic nervous system (ANS) and heart rate variability in amateur cyclists: a prospective quantitative research design
Source: BMC Sports Sci Med Rehabil. 2023 Jan 2;15:2. doi: 10.1186/s13102-022-00614-y (PMC9808932; doi:10.1186/s13102-022-00614-y)
Supplement: Supplementary file 1 — Additional file 1. Individual data of Participants. [file 13102_2022_614_MOESM1_ESM.zip › Individual data of Participants/HRV Data/009/ECG_009_20180506083755_.PDF]

Anton Swart Biokinetic Rehabilitation Practice

Name: 009 009 009
Number: 009
Gender: Female
Birthdate: 21/01/1958 60 years

Recorded: 06/05/2018 08:37:55
Recorded by: Mr. Anton Swart
Referring physician:
Ordering physician:
Attending physician:
Location: Anton Swart Biokinetic Rehabilitation Practi
Comment:

UNCONFIRMED INTERPRETATION - MD SHOULD REVIEW

P / PQ: 112 ms / 150 ms
QRS: 83 ms
QT / QTc / QTd: 475 ms / 466 ms / -
P/QRS/T axis: 78° / 83° / 72°
Heartrate: 55 bpm

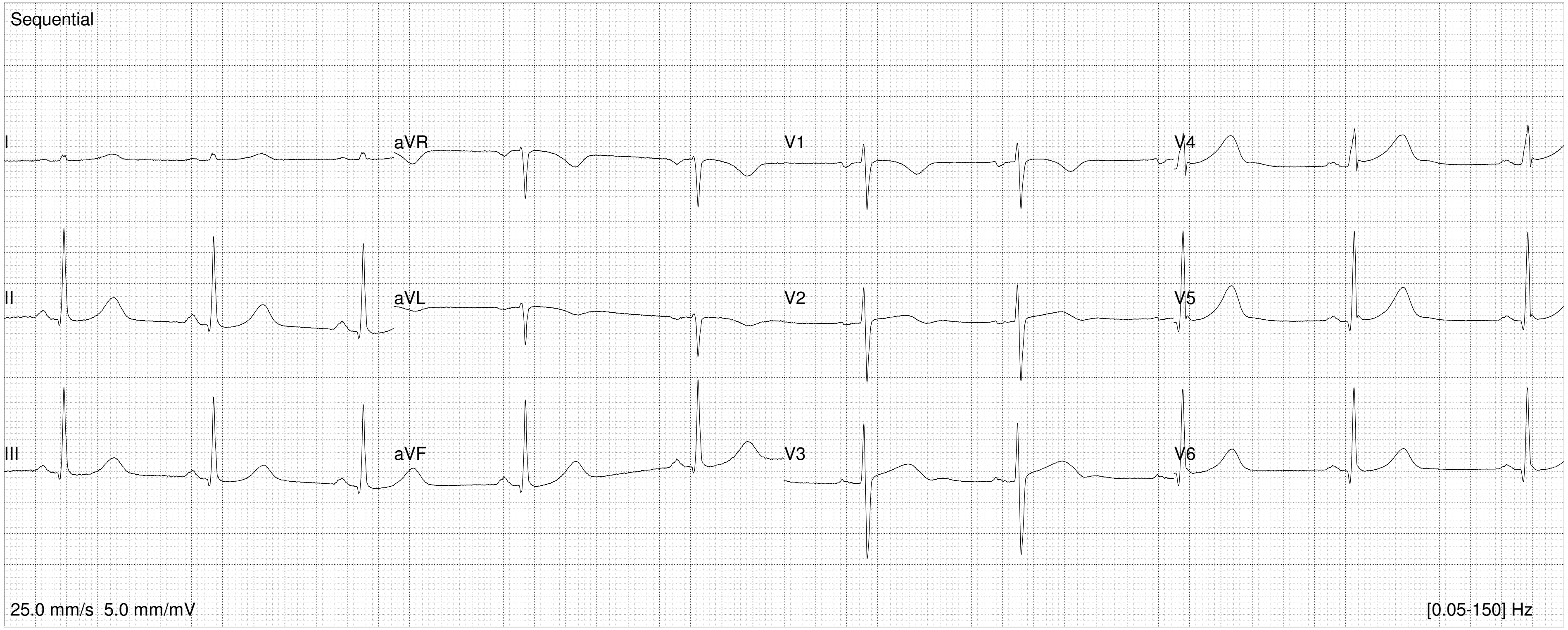

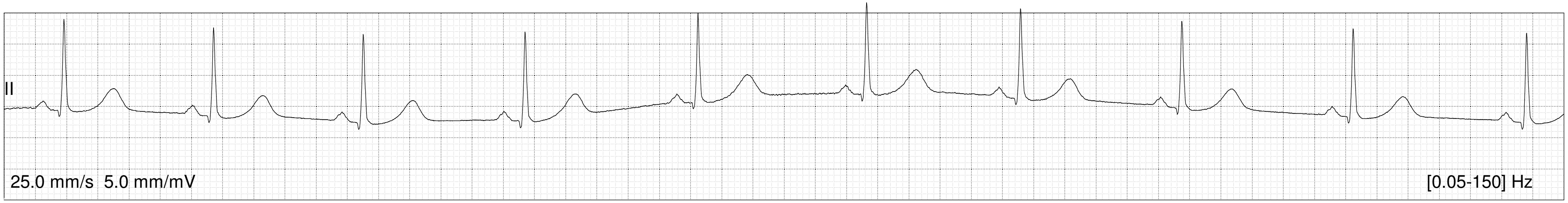

Anton Swart Biokinetic Rehabilitation Practice

Name:	009 009 009	Recorded:	06/05/2018 08:37:55	UNCONFIRMED INTERPRETATION - MD SHOULD REVIEW
Number:	009	Recorded by:	Mr. Anton Swart	
Gender:	Female	Referring physician:		
Birthdate:	21/01/1958 60 years	Location:	Anton Swart Biokinetic Rehabilitation Practice	
P / PQ:	112 ms / 150 ms	Ordering physician:		
QRS:	83 ms	Attending physician:		
QT / QTc / QTd:	475 ms / 466 ms / -	Comment:		
P/QRS/T axis:	78° / 83° / 72°			
Heartrate:	55 bpm			

**Beats**		**RR**	
Total:	275	Minimum	897 ms
Normal:	275	Maximum	1288 ms
Other:	0	Mean:	1088 ms
		SD:	90 ms

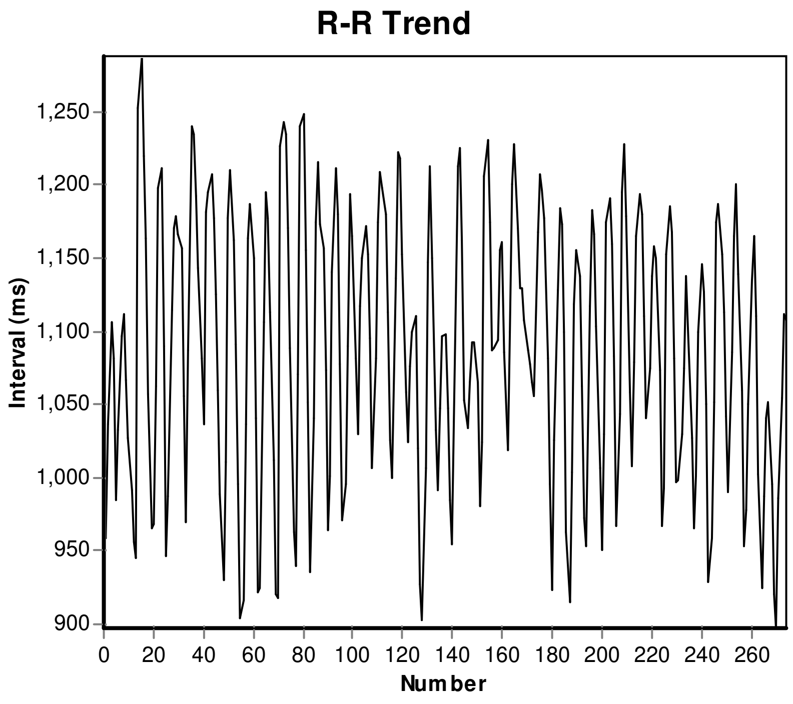

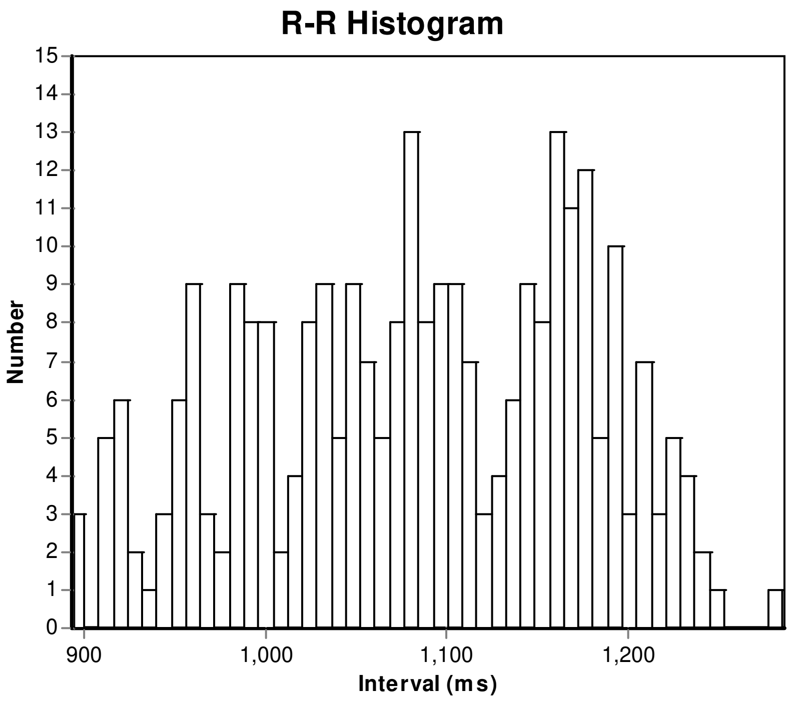

Heart Rate Variability: Time Domain Analysis

**Name:**	009, 009 009	**Birthdate:**	21/01/1958
**Number:**	009	**Recorded:**	06/05/2018 08:37:55
**Gender:**	Female		

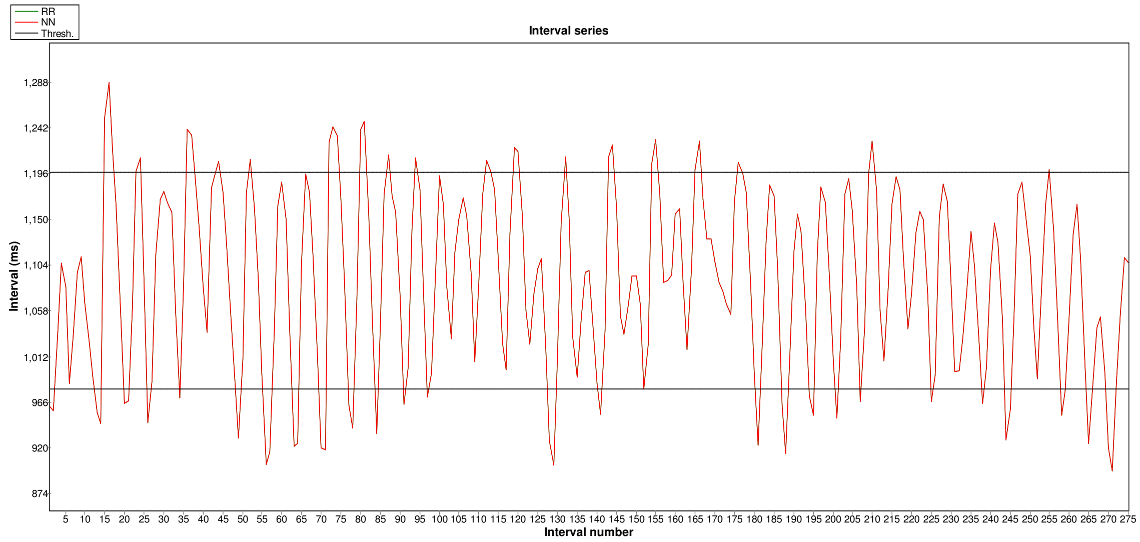

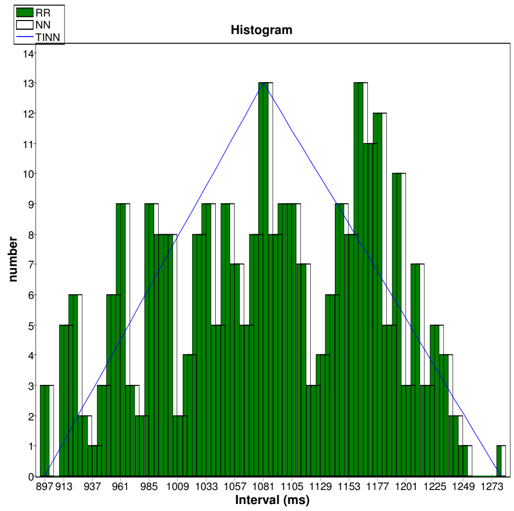

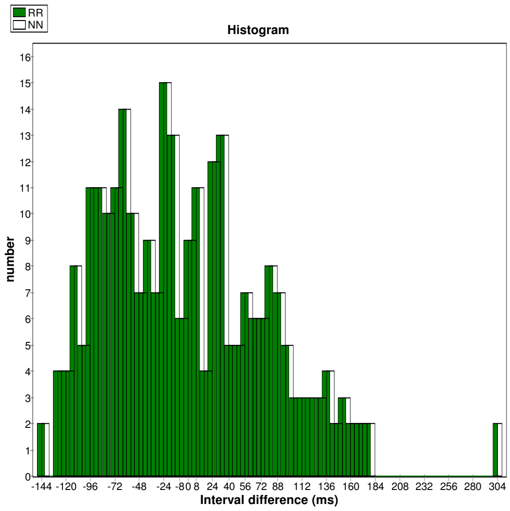

Binsize (ms) = 8

**HRV parameters**	**NN**	**RR**
SDNN (ms)	90	90
Triangular Interpolation (ms)	384	384
Triangular Index	21.15	21.15

**HRV parameters**	**NN**	**RR**
SDSD (ms)	80	80
RMSSD (ms)	80	80
NN50	157	157
NN50(1)	86	86
NN50(2)	71	71
pNN50	0.57	0.57
pNN50(1)	0.31	0.31
pNN50(2)	0.26	0.26
Logarithmic Index	0.12	0.12
SD(Logarithmic Index)	0.01	0.01

**Interval statistics**	**NN**	**RR**
Number	275	275
Minimum (ms)	897	897
Maximum (ms)	1288	1288
Range (ms)	391	391
Avg (ms)	1088	1088
SD (ms)	90	90
AvgDev (ms)	76	76
p5 (ms)	928	928
p50 (ms)	1090	1090
p95 (ms)	1225	1225
Skewness	-0.18	-0.18
Kurtosis	2.06	2.06

**Interval statistics**	**NN**	**RR**
Number	274	274
Minimum (ms)	-144	-144
Maximum (ms)	309	309
Range (ms)	453	453
Avg (ms)	1	1
SD (ms)	80	80
AvgDev (ms)	65	65
p5 (ms)	-109	-109
p50 (ms)	-11	-11
p95 (ms)	143	143
Skewness	0.68	0.68
Kurtosis	3.46	3.46

Heart Rate Variability: Frequency Domain Analysis

**Name:**	009, 009 009	**Birthdate:**	21/01/1958
**Number:**	009	**Recorded:**	06/05/2018 08:37:55
**Gender:**	Female		

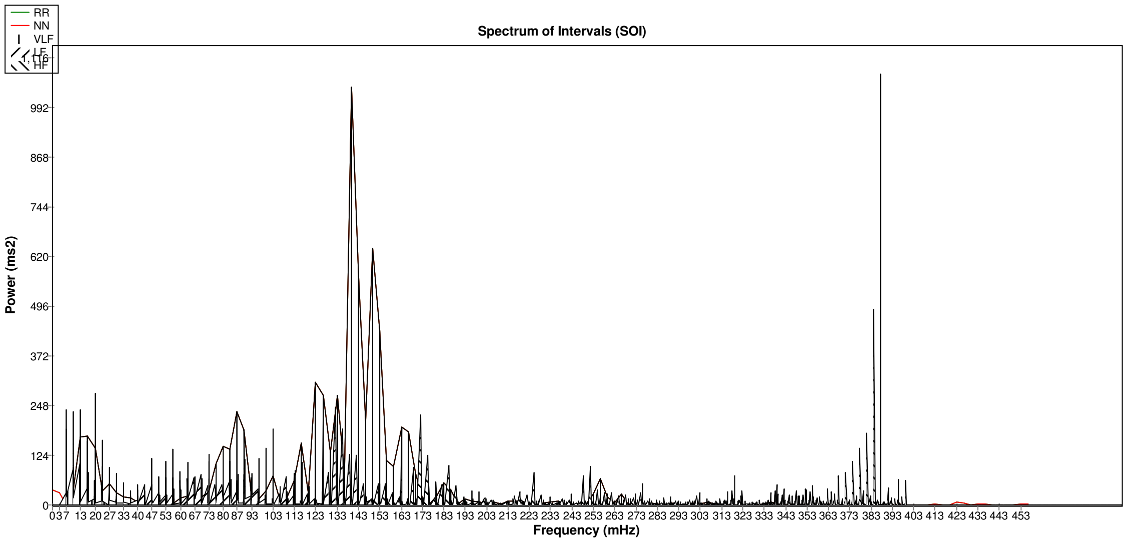

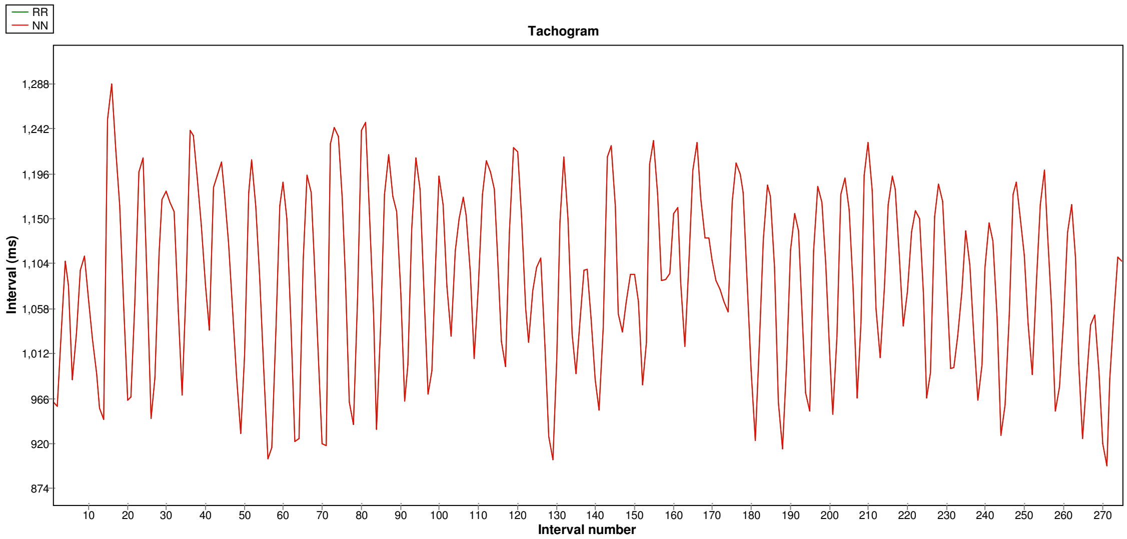

**HRV parameters**	**NN**	**RR**
TP (ms2)	7117	7117
VLF (ms2)	685	685
LF (ms2)	4877	4877
HF (ms2)	1554	1554
LF/HF	3.14	3.14
LF normalized	75.83	75.83
HF normalized	24.17	24.17
VLF peak (mHz)	17	17
LF peak (mHz)	140	140
HF peak (mHz)	153	153

**HRV spectral settings**	
Spectrum of Intervals (SOI)	
Frequency resolution (mHz)	3
VLF lower boundary (mHz)	3
VLF upper boundary (mHz)	40
LF upper boundary (mHz)	150
HF upper boundary (mHz)	400
Smoothing factor	1
Tapering	Hann
Fourier transform	DFT
Sample frequency (Hz)	0.92
Interval correction	Annotation
Interval threshold (%)	10
