# Supplementary material for: The effects of a 3-day mountain bike cycling race on the autonomic nervous system (ANS) and heart rate variability in amateur cyclists: a prospective quantitative research design
Source: BMC Sports Sci Med Rehabil. 2023 Jan 2;15:2. doi: 10.1186/s13102-022-00614-y (PMC9808932; doi:10.1186/s13102-022-00614-y)
Supplement: Supplementary file 1 — Additional file 1. Individual data of Participants. [file 13102_2022_614_MOESM1_ESM.zip › Individual data of Participants/HRV Data/010/ECG_010_20180501111307_.PDF]

Anton Swart Biokinetic Rehabilitation Practice

Name:	011 011 011	Recorded:	01/05/2018 11:13:07	UNCONFIRMED INTERPRETATION - MD SHOULD REVIEW
Number:	011	Recorded by:	Mr. Anton Swart	
Gender:	Male	Referring physician:		
Birthdate:	18/01/1976 42 years	Ordering physician:		
		Attending physician:		
P / PQ:	115 ms / 192 ms	Location:	Anton Swart Biokinetic Rehabilitation Practi	
QRS:	85 ms	Comment:		
QT / QTc / QTd:	386 ms / 398 ms / -			
P/QRS/T axis:	78° / 72° / 64°			
Heartrate:	67 bpm			

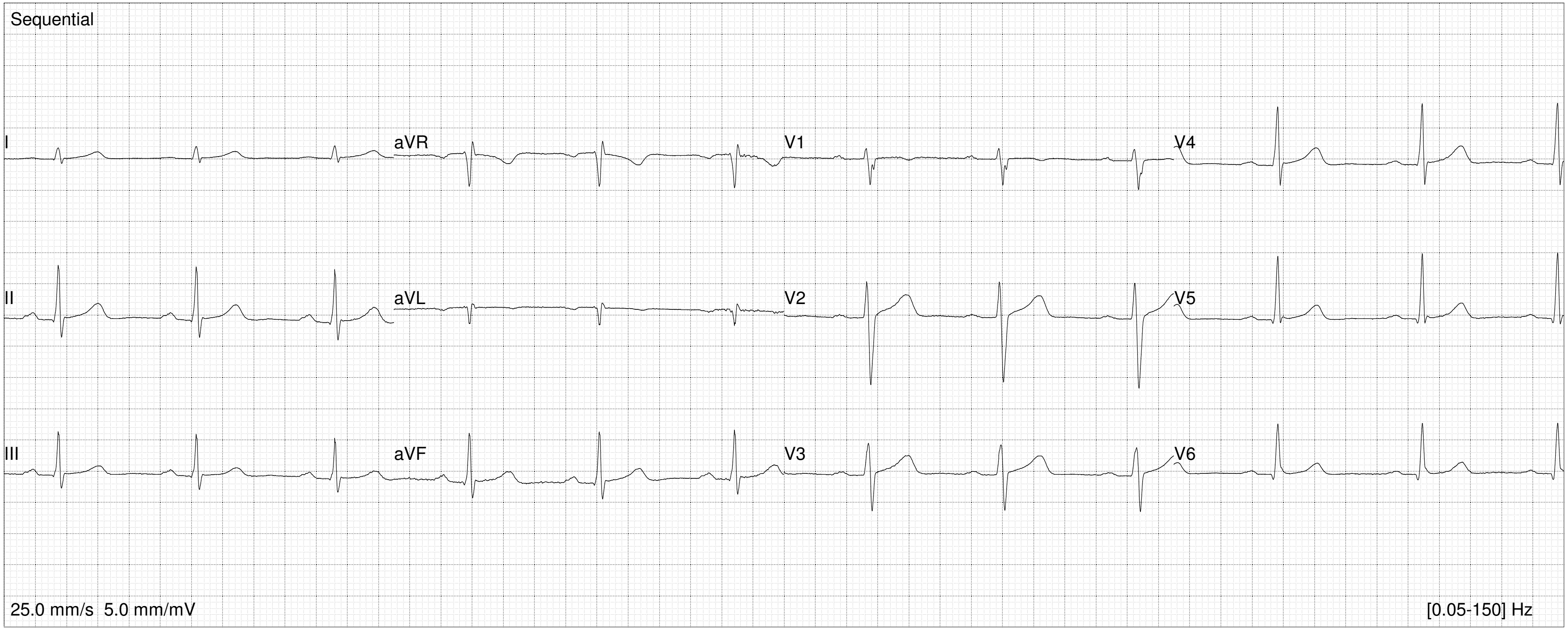

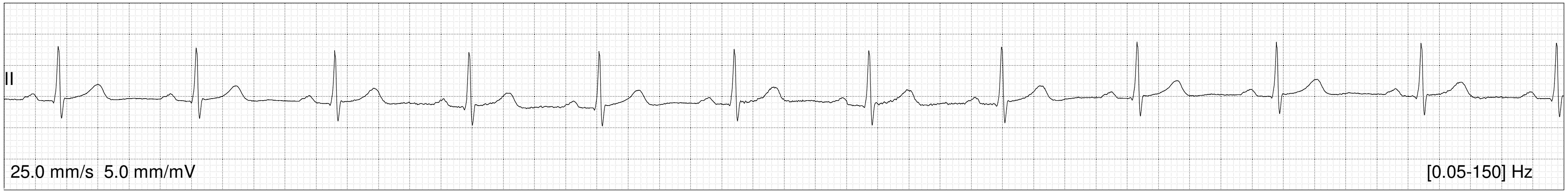

Anton Swart Biokinetic Rehabilitation Practice

Name:	011 011 011	Recorded:	01/05/2018 11:13:07	UNCONFIRMED INTERPRETATION - MD SHOULD REVIEW
Number:	011	Recorded by:	Mr. Anton Swart	
Gender:	Male	Referring physician:		
Birthdate:	18/01/1976 42 years	Location:	Anton Swart Biokinetic Rehabilitation Practice	
		Ordering physician:		
		Attending physician:		
		Comment:		

P / PQ:	115 ms / 192 ms
QRS:	85 ms
QT / QTc / QTd:	386 ms / 398 ms / -
P/QRS/T axis:	78° / 72° / 64°
Heartrate:	67 bpm

**Beats**		**RR**	
Total:	330	Minimum	730 ms
Normal:	330	Maximum	1031 ms
Other:	0	Mean:	905 ms
		SD:	57 ms

R-R Trend

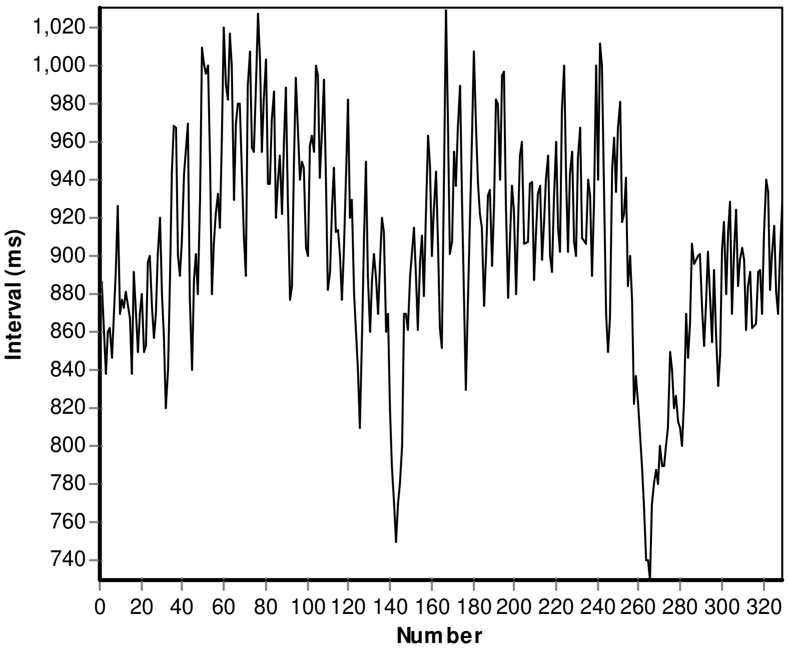

R-R Histogram

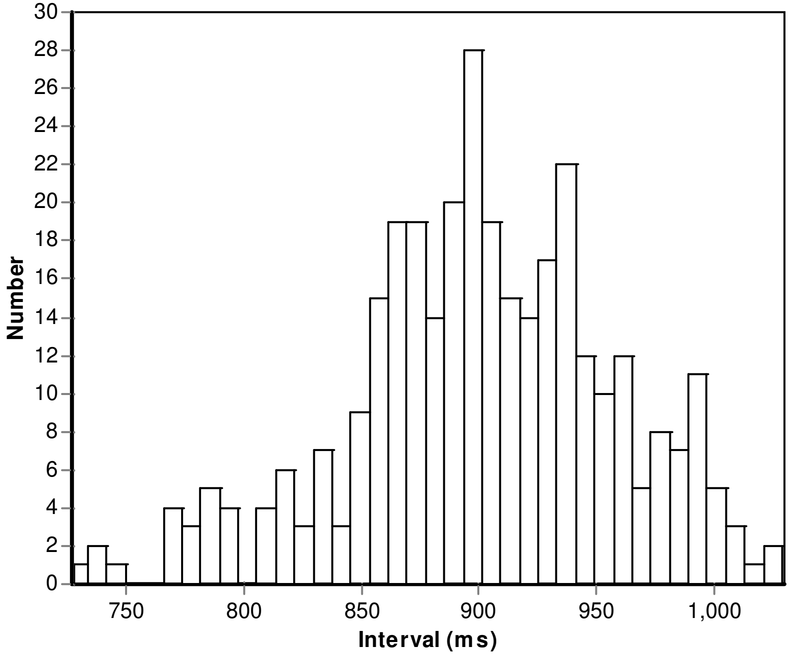

Cardio Control NV. User: Anton

Heart Rate Variability: Time Domain Analysis

**Name:**	011, 011 011	**Birthdate:**	18/01/1976
**Number:**	011	**Recorded:**	01/05/2018 11:13:07
**Gender:**	Male		

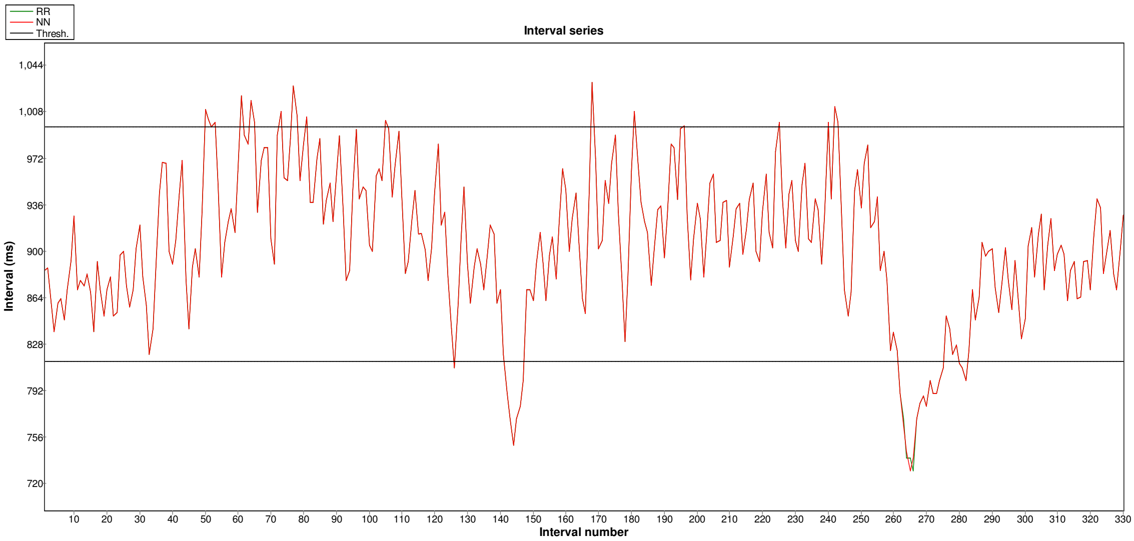

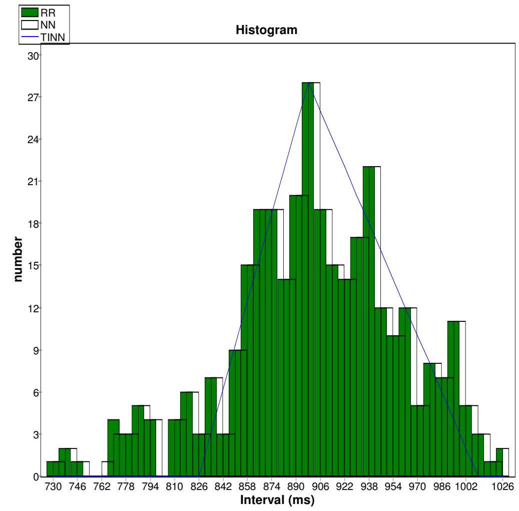

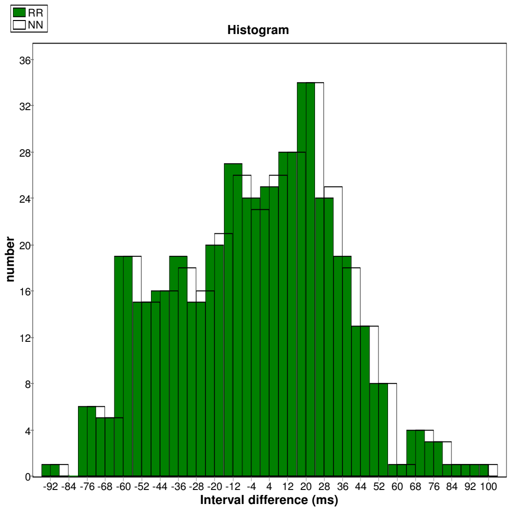

Binsize (ms) = 8

**HRV parameters**	**NN**	**RR**
SDNN (ms)	57	57
Triangular Interpolation (ms)	184	184
Triangular Index	11.79	11.79

**HRV parameters**	**NN**	**RR**
SDSD (ms)	36	36
RMSSD (ms)	36	36
NN50	54	54
NN50(1)	35	35
NN50(2)	19	19
pNN50	0.16	0.16
pNN50(1)	0.11	0.11
pNN50(2)	0.06	0.06
Logarithmic Index	0.33	0.33
SD(Logarithmic Index)	0.04	0.04

**Interval statistics**	**NN**	**RR**
Number	330	330
Minimum (ms)	730	730
Maximum (ms)	1031	1031
Range (ms)	301	301
Avg (ms)	905	905
SD (ms)	57	57
AvgDev (ms)	44	44
p5 (ms)	795	795
p50 (ms)	905	905
p95 (ms)	1000	1000
Skewness	-0.37	-0.38
Kurtosis	3.19	3.19

**Interval statistics**	**NN**	**RR**
Number	329	329
Minimum (ms)	-92	-92
Maximum (ms)	100	100
Range (ms)	192	192
Avg (ms)	0	0
SD (ms)	36	36
AvgDev (ms)	30	30
p5 (ms)	-59	-59
p50 (ms)	3	3
p95 (ms)	56	56
Skewness	-0.04	-0.04
Kurtosis	2.52	2.51

Cardio Control NV. User: Anton

Heart Rate Variability: Frequency Domain Analysis

**Name:**	011, 011 011	**Birthdate:**	18/01/1976
**Number:**	011	**Recorded:**	01/05/2018 11:13:07
**Gender:**	Male		

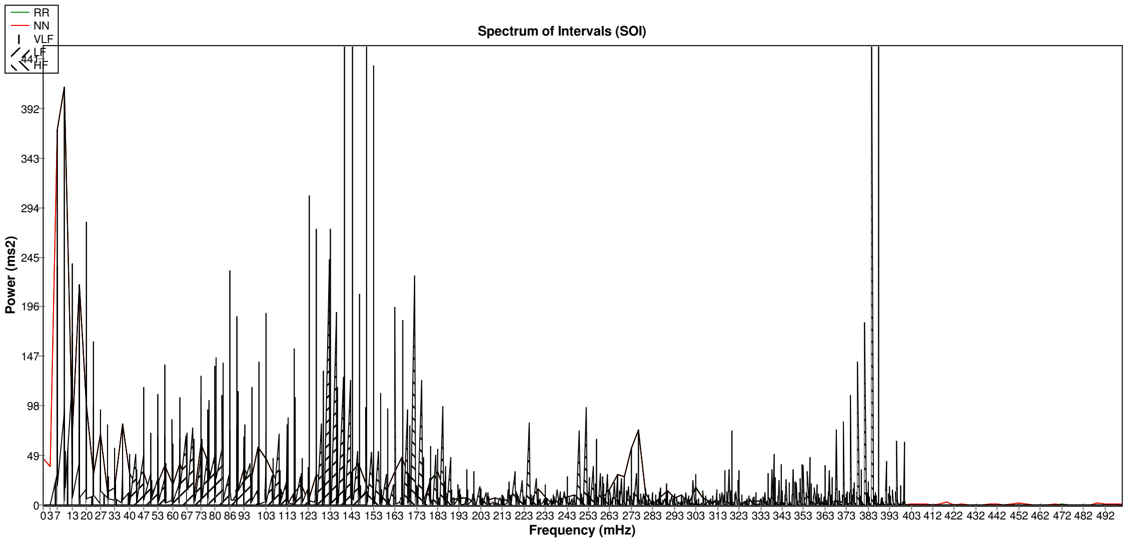

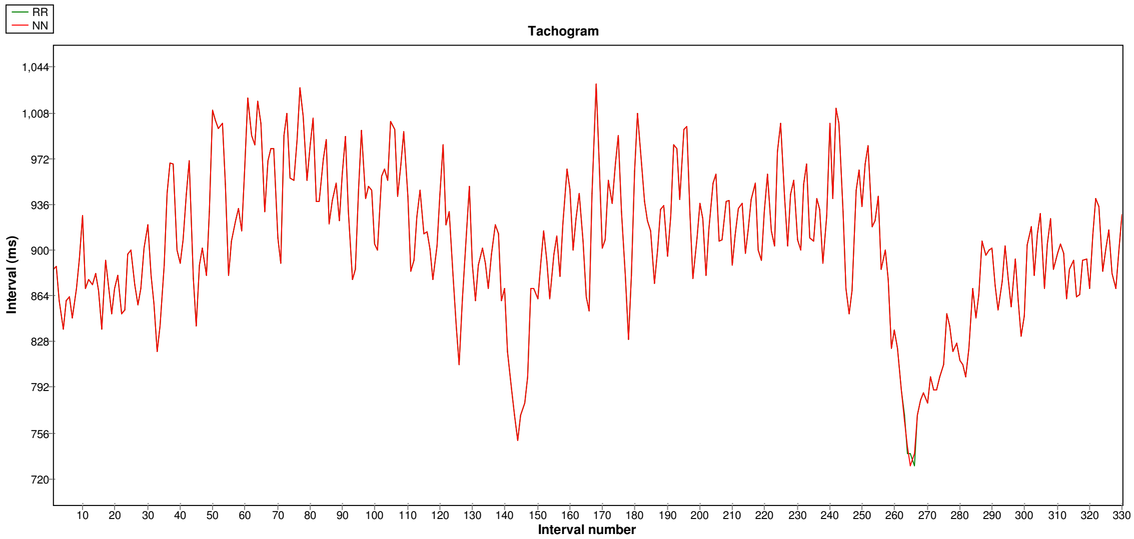

**HRV parameters**	**NN**	**RR**
TP (ms2)	2855	2855
VLF (ms2)	1413	1413
LF (ms2)	745	745
HF (ms2)	697	697
LF/HF	1.07	1.07
LF normalized	51.67	51.67
HF normalized	48.33	48.33
VLF peak (mHz)	10	10
LF peak (mHz)	73	73
HF peak (mHz)	276	276

**HRV spectral settings**	
Spectrum of Intervals (SOI)	
Frequency resolution (mHz)	3
VLF lower boundary (mHz)	3
VLF upper boundary (mHz)	40
LF upper boundary (mHz)	150
HF upper boundary (mHz)	400
Smoothing factor	1
Tapering	Hann
Fourier transform	DFT
Sample frequency (Hz)	1.10
Interval correction	Annotation
Interval threshold (%)	10
