# Supplementary material for: The effects of a 3-day mountain bike cycling race on the autonomic nervous system (ANS) and heart rate variability in amateur cyclists: a prospective quantitative research design
Source: BMC Sports Sci Med Rehabil. 2023 Jan 2;15:2. doi: 10.1186/s13102-022-00614-y (PMC9808932; doi:10.1186/s13102-022-00614-y)
Supplement: Supplementary file 1 — Additional file 1. Individual data of Participants. [file 13102_2022_614_MOESM1_ESM.zip › Individual data of Participants/HRV Data/010/ECG_010_20180503161744_.PDF]

Anton Swart Biokinetic Rehabilitation Practice

Name:	011 011 011	Recorded:	03/05/2018 16:17:44	UNCONFIRMED INTERPRETATION - MD SHOULD REVIEW
Number:	011	Recorded by:	Mr. Anton Swart	
Gender:	Male	Referring physician:		
Birthdate:	18/01/1976 42 years	Ordering physician:		
		Attending physician:		
P / PQ:	122 ms / 187 ms	Location:	Anton Swart Biokinetic Rehabilitation Practi	
QRS:	83 ms	Comment:		
QT / QTc / QTd:	360 ms / 420 ms / -			
P/QRS/T axis:	83° / 80° / 68°			
Heartrate:	94 bpm			

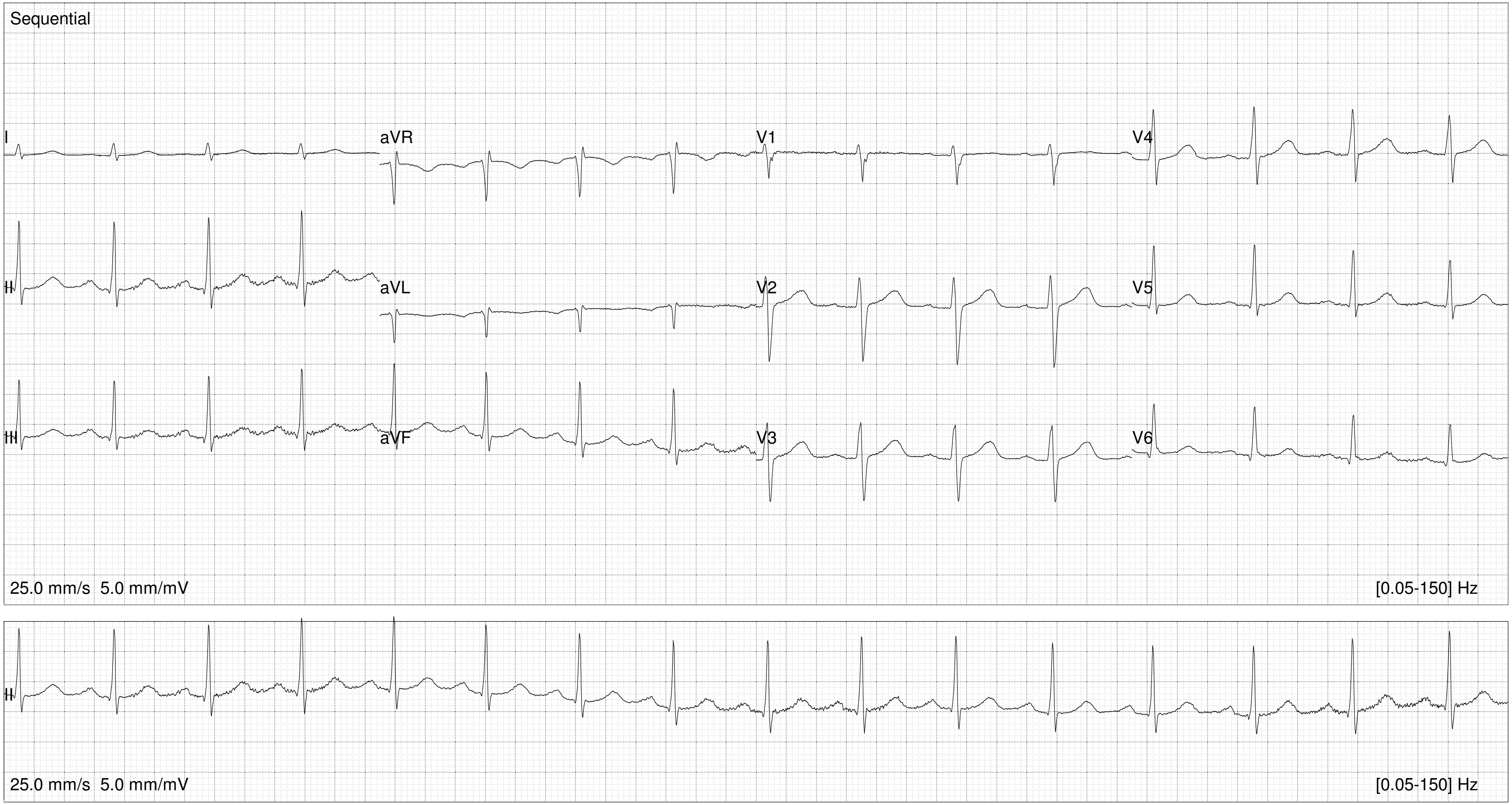

Anton Swart Biokinetic Rehabilitation Practice

Name:	011 011 011	Recorded:	03/05/2018 16:17:44	UNCONFIRMED INTERPRETATION - MD SHOULD REVIEW
Number:	011	Recorded by:	Mr. Anton Swart	
Gender:	Male	Referring physician:		
Birthdate:	18/01/1976 42 years	Location:	Anton Swart Biokinetic Rehabilitation Practice	
P / PQ:	122 ms / 187 ms	Ordering physician:		
QRS:	83 ms	Attending physician:		
QT / QTc / QTd:	360 ms / 420 ms / -	Comment:		
P/QRS/T axis:	83° / 80° / 68°			
Heartrate:	94 bpm			

**Beats**		**RR**	
Total:	464	Minimum	600 ms
Normal:	464	Maximum	746 ms
Other:	0	Mean:	645 ms
		SD:	23 ms

R-R Trend

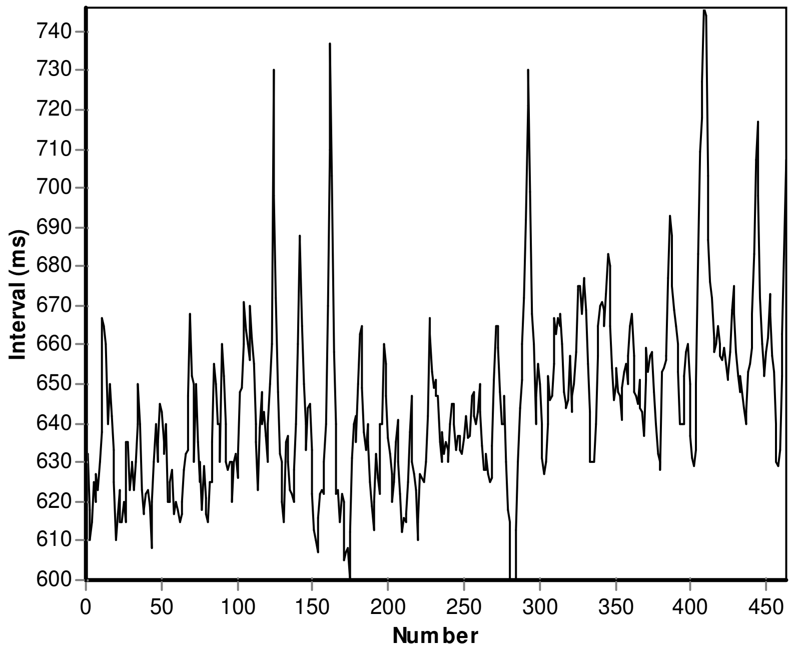

R-R Histogram

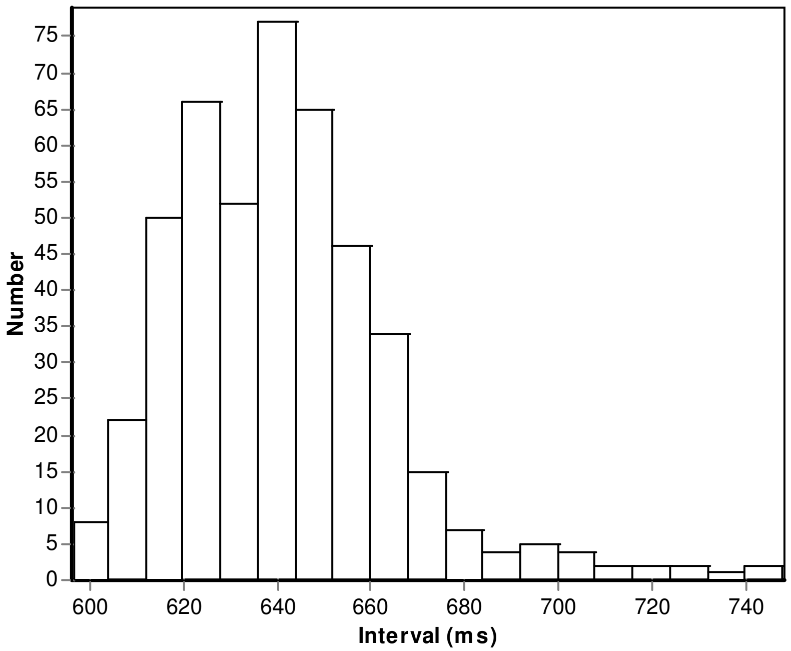

Cardio Control NV. User: Anton

Heart Rate Variability: Time Domain Analysis

**Name:**	011, 011 011	**Birthdate:**	18/01/1976
**Number:**	011	**Recorded:**	03/05/2018 16:17:44
**Gender:**	Male		

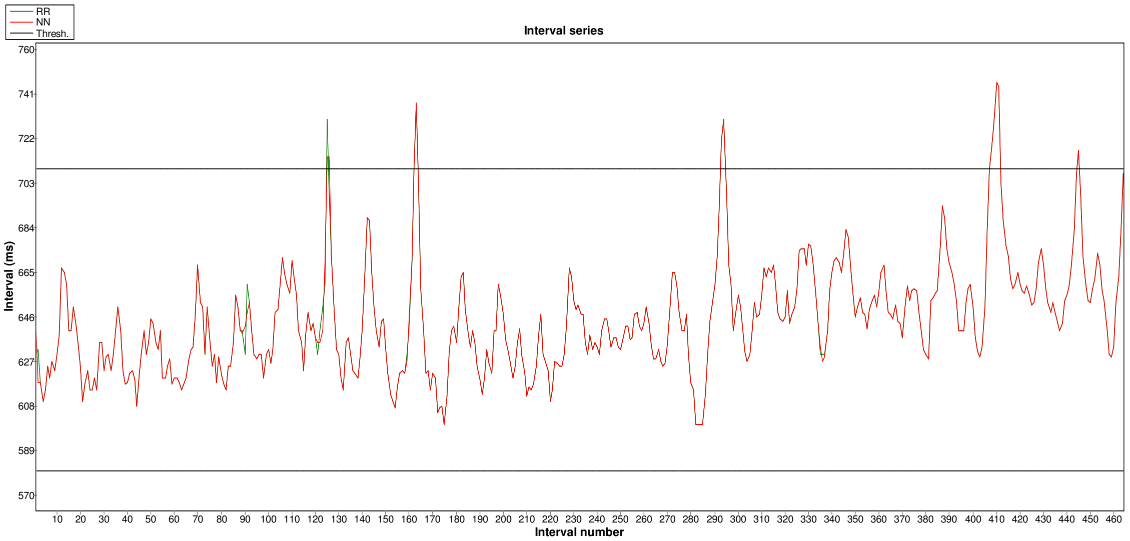

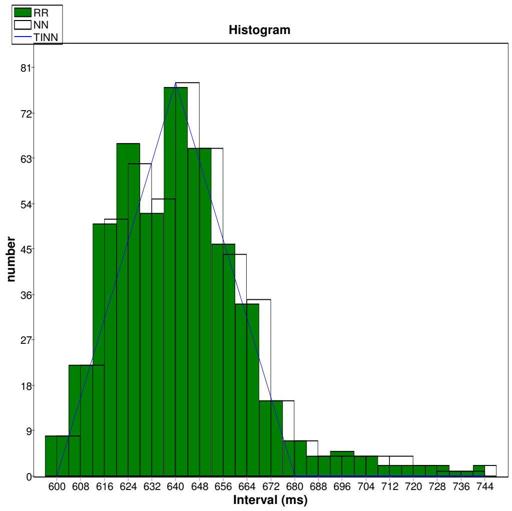

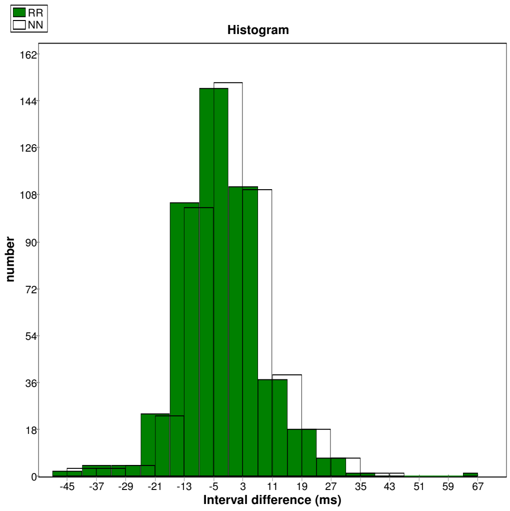

Binsize (ms) = 8

**HRV parameters**	**NN**	**RR**
SDNN (ms)	23	23
Triangular Interpolation (ms)	80	80
Triangular Index	5.95	6.03

**HRV parameters**	**NN**	**RR**
SDSD (ms)	11	11
RMSSD (ms)	11	11
NN50	0	1
NN50(1)	0	0
NN50(2)	0	1
pNN50	0.00	0.00
pNN50(1)	0.00	0.00
pNN50(2)	0.00	0.00
Logarithmic Index	0.95	0.96
SD(Logarithmic Index)	0.11	0.07

**Interval statistics**	**NN**	**RR**
Number	464	464
Minimum (ms)	600	600
Maximum (ms)	746	746
Range (ms)	146	146
Avg (ms)	645	645
SD (ms)	23	23
AvgDev (ms)	18	18
p5 (ms)	615	615
p50 (ms)	641	640
p95 (ms)	687	687
Skewness	1.18	1.20
Kurtosis	5.48	5.58

**Interval statistics**	**NN**	**RR**
Number	463	463
Minimum (ms)	-45	-45
Maximum (ms)	44	70
Range (ms)	89	115
Avg (ms)	0	0
SD (ms)	11	11
AvgDev (ms)	8	8
p5 (ms)	-16	-16
p50 (ms)	0	0
p95 (ms)	20	20
Skewness	0.04	0.47
Kurtosis	4.88	6.81

Cardio Control NV. User: Anton

Heart Rate Variability: Frequency Domain Analysis

**Name:**	011, 011 011	**Birthdate:**	18/01/1976
**Number:**	011	**Recorded:**	03/05/2018 16:17:44
**Gender:**	Male		

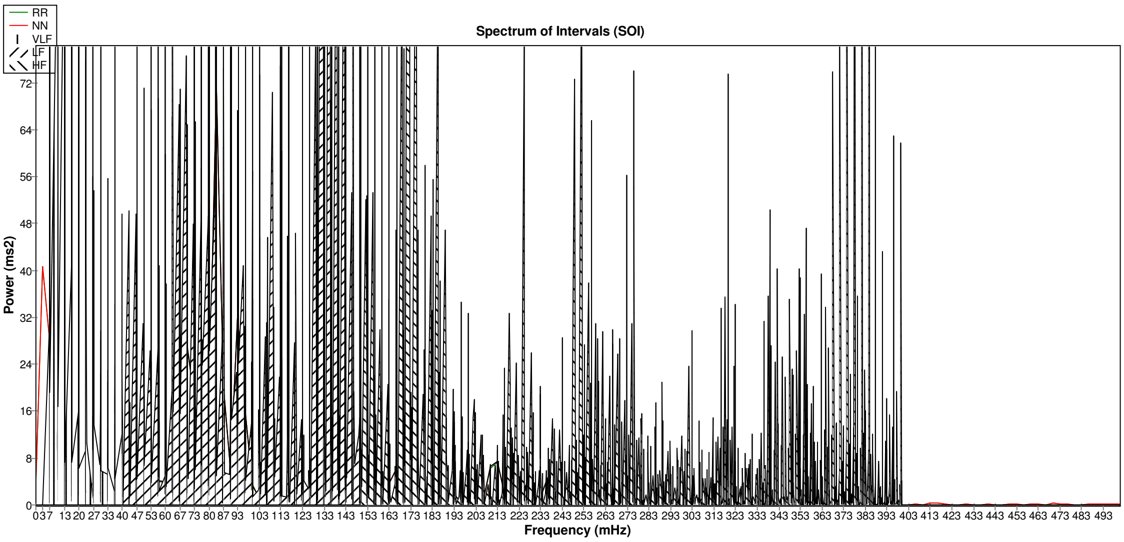

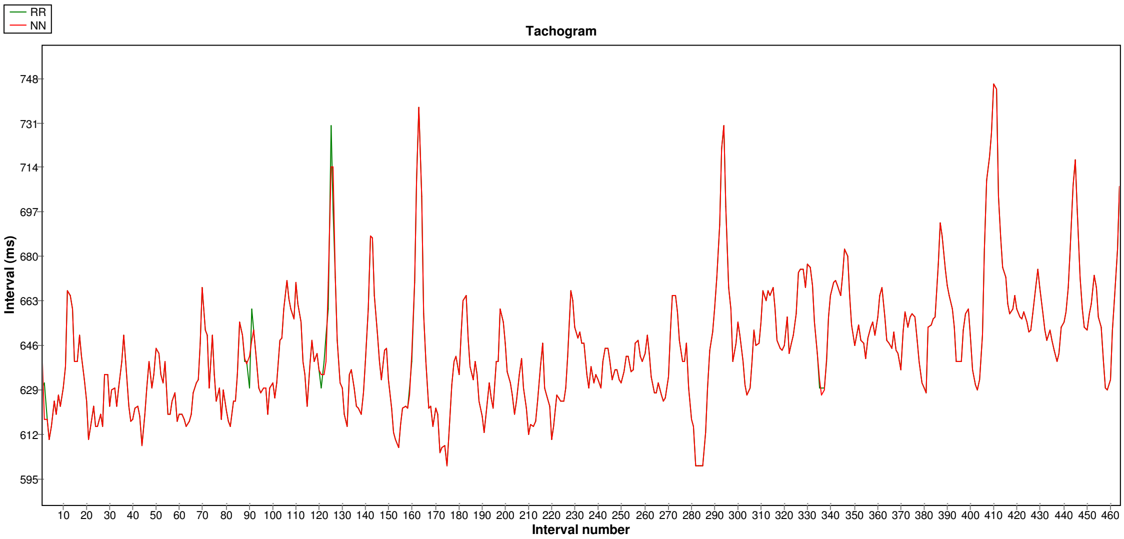

HRV parameters	NN	RR
TP (ms2)	425	424
VLF (ms2)	54	55
LF (ms2)	292	291
HF (ms2)	79	78
LF/HF	3.69	3.72
LF normalized	78.70	78.82
HF normalized	21.30	21.18
VLF peak (mHz)	7	7
LF peak (mHz)	83	83
HF peak (mHz)	213	213

HRV spectral settings	
Spectrum of Intervals (SOI)	
Frequency resolution (mHz)	3
VLF lower boundary (mHz)	3
VLF upper boundary (mHz)	40
LF upper boundary (mHz)	150
HF upper boundary (mHz)	400
Smoothing factor	1
Tapering	Hann
Fourier transform	DFT
Sample frequency (Hz)	1.55
Interval correction	Annotation
Interval threshold (%)	10
