# Supplementary material for: The effects of a 3-day mountain bike cycling race on the autonomic nervous system (ANS) and heart rate variability in amateur cyclists: a prospective quantitative research design
Source: BMC Sports Sci Med Rehabil. 2023 Jan 2;15:2. doi: 10.1186/s13102-022-00614-y (PMC9808932; doi:10.1186/s13102-022-00614-y)
Supplement: Supplementary file 1 — Additional file 1. Individual data of Participants. [file 13102_2022_614_MOESM1_ESM.zip › Individual data of Participants/HRV Data/010/ECG_010_20180504122400_.PDF]

Anton Swart Biokinetic Rehabilitation Practice

Name:	011 011 011	Recorded:	04/05/2018 12:24:00	UNCONFIRMED INTERPRETATION - MD SHOULD REVIEW
Number:	011	Recorded by:	Mr. Anton Swart	
Gender:	Male	Referring physician:		
Birthdate:	18/01/1976 42 years	Ordering physician:		
		Attending physician:		
P / PQ:	108 ms / 173 ms	Location:	Anton Swart Biokinetic Rehabilitation Practi	
QRS:	82 ms	Comment:		
QT / QTc / QTd:	369 ms / 413 ms / -			
P/QRS/T axis:	83° / 81° / 74°			
Heartrate:	85 bpm			

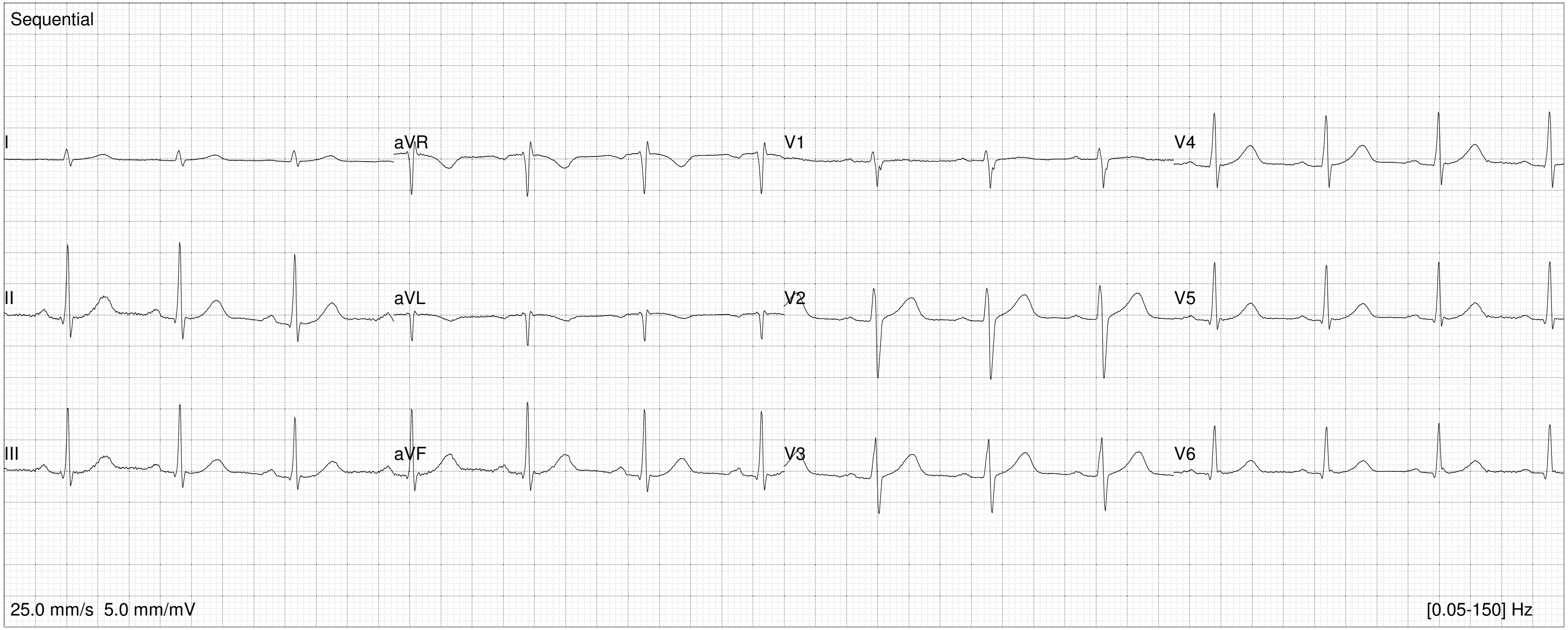

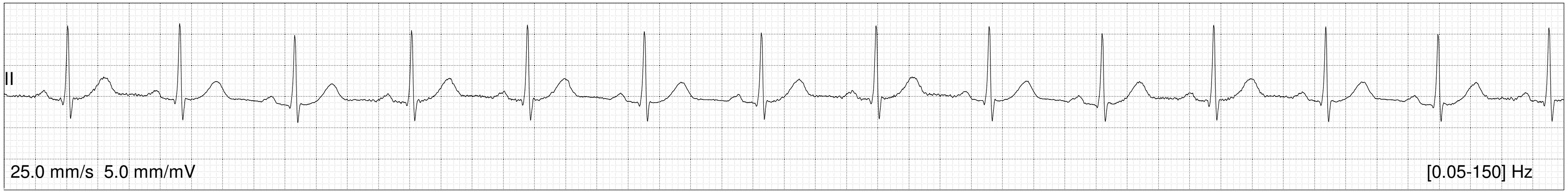

Anton Swart Biokinetic Rehabilitation Practice

Name:	011 011 011	Recorded:	04/05/2018 12:24:00	UNCONFIRMED INTERPRETATION - MD SHOULD REVIEW
Number:	011	Recorded by:	Mr. Anton Swart	
Gender:	Male	Referring physician:		
Birthdate:	18/01/1976 42 years	Location:	Anton Swart Biokinetic Rehabilitation Practice	
P / PQ:	108 ms / 173 ms	Ordering physician:		
QRS:	82 ms	Attending physician:		
QT / QTc / QTd:	369 ms / 413 ms / -	Comment:		
P/QRS/T axis:	83° / 81° / 74°			
Heartrate:	85 bpm			

**Beats**		**RR**	
Total:	421	Minimum	637 ms
Normal:	421	Maximum	774 ms
Other:	0	Mean:	710 ms
		SD:	23 ms

R-R Trend

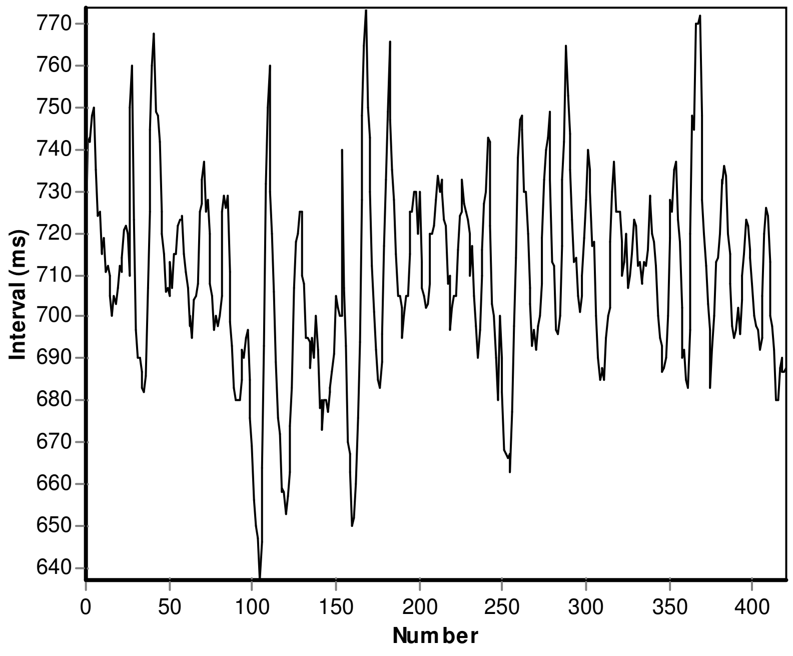

R-R Histogram

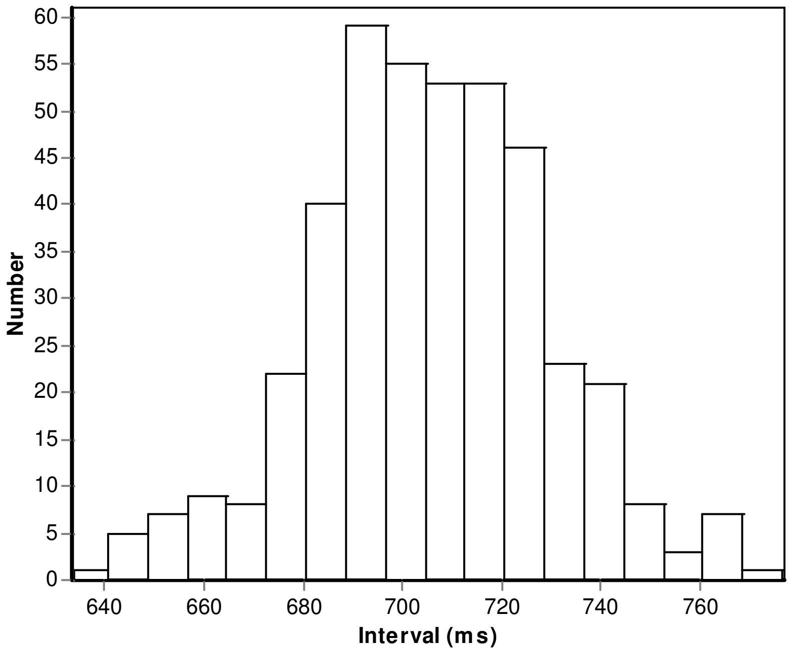

Cardio Control NV. User: Anton

Heart Rate Variability: Time Domain Analysis

**Name:**	011, 011 011	**Birthdate:**	18/01/1976
**Number:**	011	**Recorded:**	04/05/2018 12:24:00
**Gender:**	Male		

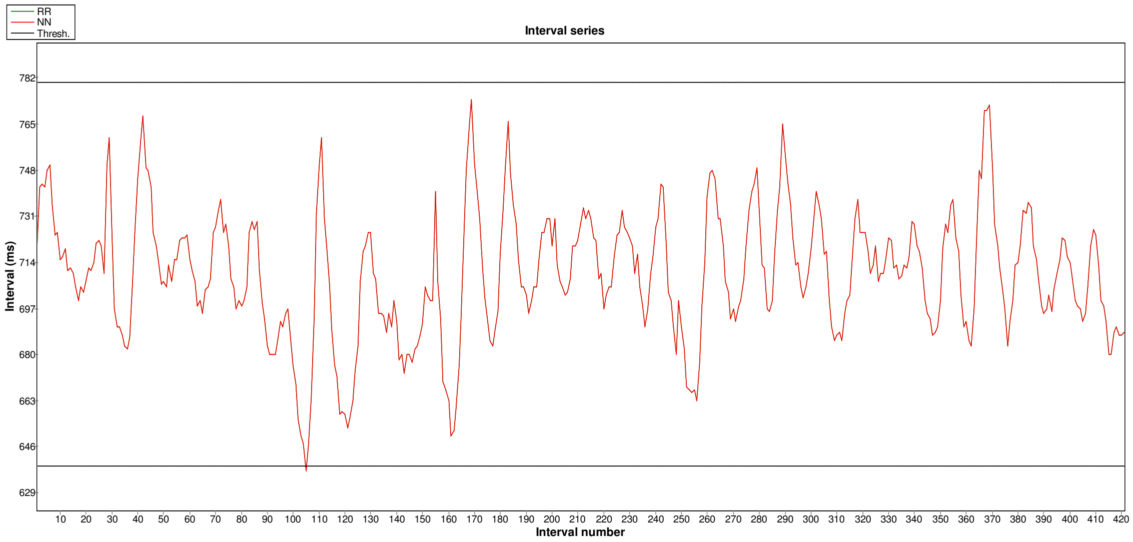

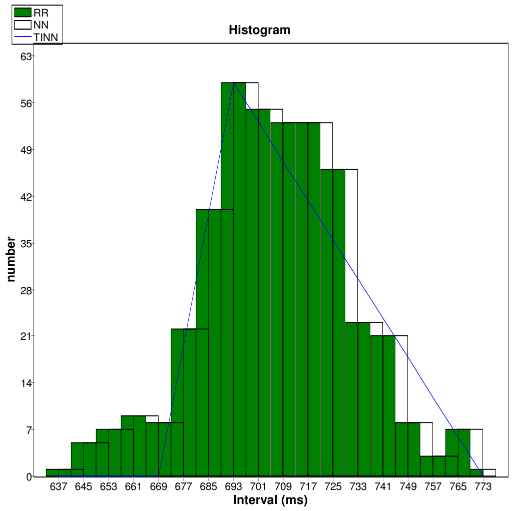

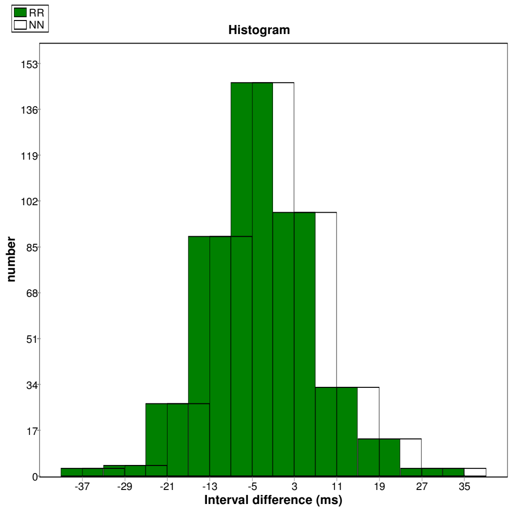

Binsize (ms) = 8

**HRV parameters**	**NN**	**RR**
SDNN (ms)	23	23
Triangular Interpolation (ms)	104	104
Triangular Index	7.14	7.14

**HRV parameters**	**NN**	**RR**
SDSD (ms)	11	11
RMSSD (ms)	11	11
NN50	0	0
NN50(1)	0	0
NN50(2)	0	0
pNN50	0.00	0.00
pNN50(1)	0.00	0.00
pNN50(2)	0.00	0.00
Logarithmic Index	1.02	1.02
SD(Logarithmic Index)	0.05	0.05

**Interval statistics**	**NN**	**RR**
Number	421	421
Minimum (ms)	637	637
Maximum (ms)	774	774
Range (ms)	137	137
Avg (ms)	710	710
SD (ms)	23	23
AvgDev (ms)	18	18
p5 (ms)	668	668
p50 (ms)	710	710
p95 (ms)	748	748
Skewness	-0.08	-0.08
Kurtosis	3.29	3.29

**Interval statistics**	**NN**	**RR**
Number	420	420
Minimum (ms)	-37	-37
Maximum (ms)	40	40
Range (ms)	77	77
Avg (ms)	-0	-0
SD (ms)	11	11
AvgDev (ms)	8	8
p5 (ms)	-16	-16
p50 (ms)	0	0
p95 (ms)	18	18
Skewness	0.37	0.37
Kurtosis	4.38	4.38

Heart Rate Variability: Frequency Domain Analysis

**Name:**	011, 011 011	**Birthdate:**	18/01/1976
**Number:**	011	**Recorded:**	04/05/2018 12:24:00
**Gender:**	Male		

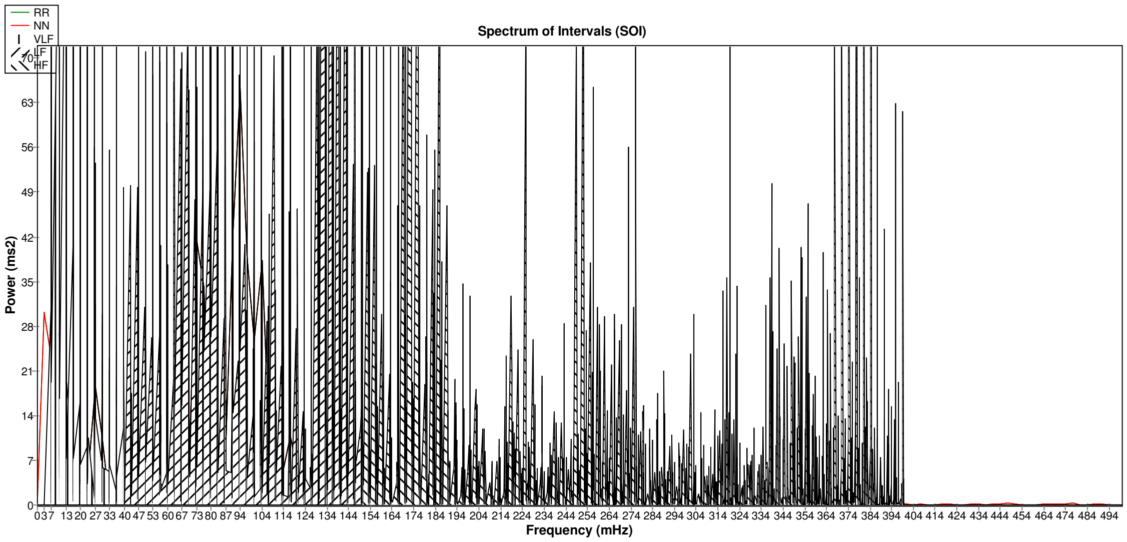

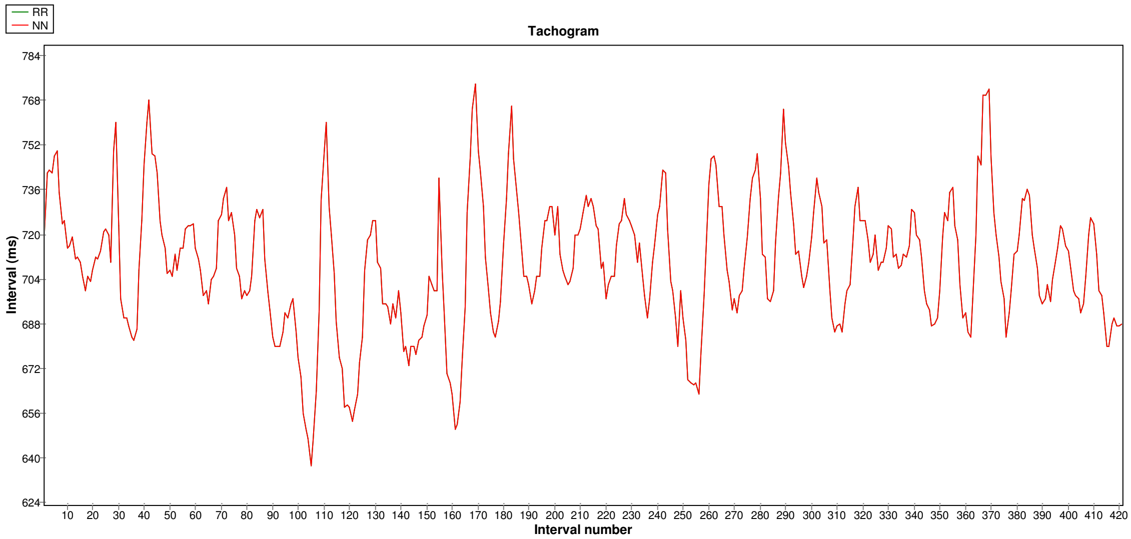

HRV parameters	NN	RR
TP (ms2)	549	549
VLF (ms2)	114	114
LF (ms2)	409	409
HF (ms2)	26	26
LF/HF	15.75	15.75
LF normalized	94.03	94.03
HF normalized	5.97	5.97
VLF peak (mHz)	10	10
LF peak (mHz)	94	94
HF peak (mHz)	160	160

HRV spectral settings	
Spectrum of Intervals (SOI)	
Frequency resolution (mHz)	3
VLF lower boundary (mHz)	3
VLF upper boundary (mHz)	40
LF upper boundary (mHz)	150
HF upper boundary (mHz)	400
Smoothing factor	1
Tapering	Hann
Fourier transform	DFT
Sample frequency (Hz)	1.41
Interval correction	Annotation
Interval threshold (%)	10
