# Supplementary material for: The effects of a 3-day mountain bike cycling race on the autonomic nervous system (ANS) and heart rate variability in amateur cyclists: a prospective quantitative research design
Source: BMC Sports Sci Med Rehabil. 2023 Jan 2;15:2. doi: 10.1186/s13102-022-00614-y (PMC9808932; doi:10.1186/s13102-022-00614-y)
Supplement: Supplementary file 1 — Additional file 1. Individual data of Participants. [file 13102_2022_614_MOESM1_ESM.zip › Individual data of Participants/HRV Data/010/ECG_010_20180505120508_.PDF]

Anton Swart Biokinetic Rehabilitation Practice

Name:	011 011 011	Recorded:	05/05/2018 12:05:08	UNCONFIRMED INTERPRETATION - MD SHOULD REVIEW
Number:	011	Recorded by:	Mr. Anton Swart	
Gender:	Male	Referring physician:		
Birthdate:	18/01/1976 42 years	Ordering physician:		
		Attending physician:		
P / PQ:	107 ms / 155 ms	Location:	Anton Swart Biokinetic Rehabilitation Practi	
QRS:	80 ms	Comment:		
QT / QTc / QTd:	387 ms / 420 ms / -			
P/QRS/T axis:	85° / 84° / 80°			
Heartrate:	79 bpm			

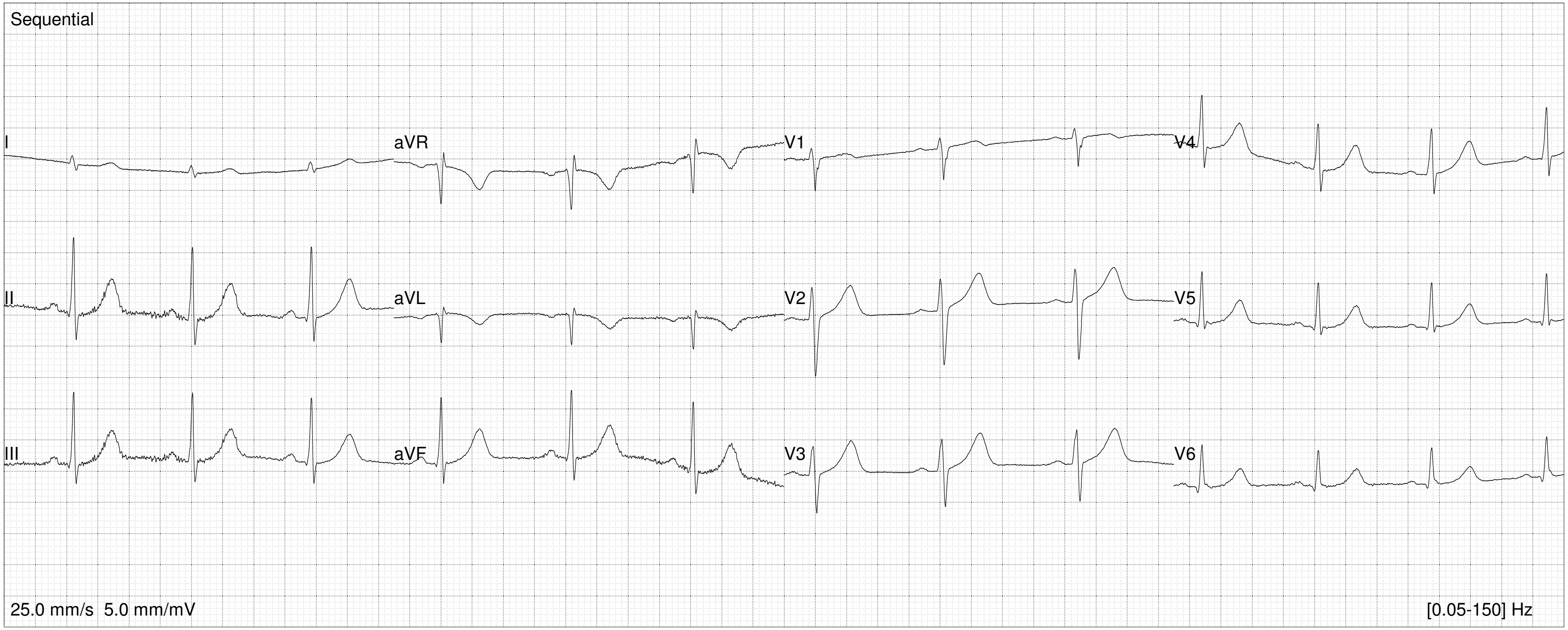

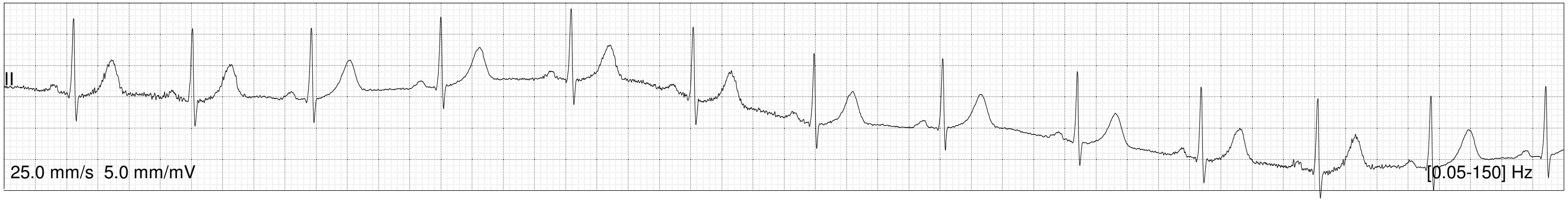

Anton Swart Biokinetic Rehabilitation Practice

Name:	011 011 011	Recorded:	05/05/2018 12:05:08	UNCONFIRMED INTERPRETATION - MD SHOULD REVIEW
Number:	011	Recorded by:	Mr. Anton Swart	
Gender:	Male	Referring physician:		
Birthdate:	18/01/1976 42 years	Location:	Anton Swart Biokinetic Rehabilitation Practice	
P / PQ:	107 ms / 155 ms	Ordering physician:		
QRS:	80 ms	Attending physician:		
QT / QTc / QTd:	387 ms / 420 ms / -	Comment:		
P/QRS/T axis:	85° / 84° / 80°			
Heartrate:	79 bpm			

Beats

Total:	387
Normal:	387
Other:	0

RR

Minimum	678 ms
Maximum	1230 ms
Mean:	772 ms
SD:	78 ms

R-R Trend

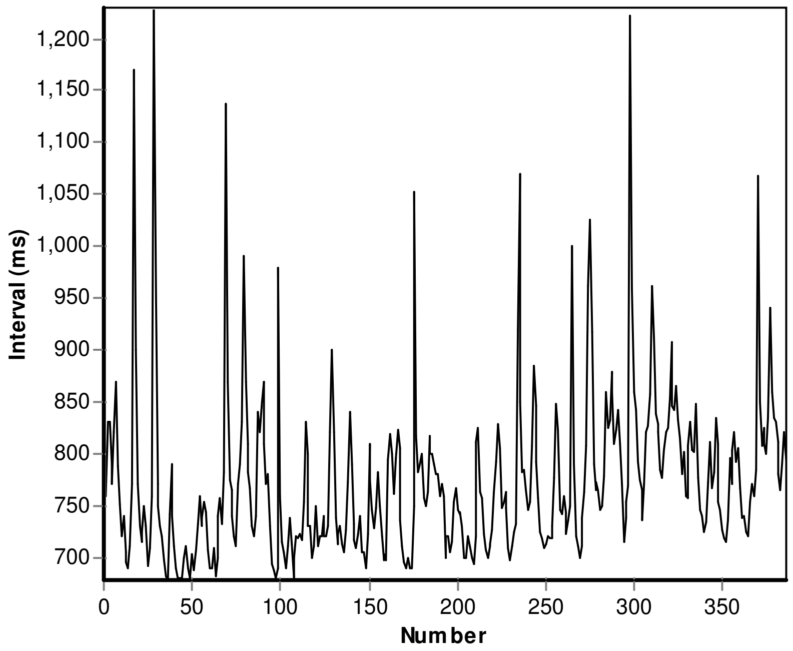

R-R Histogram

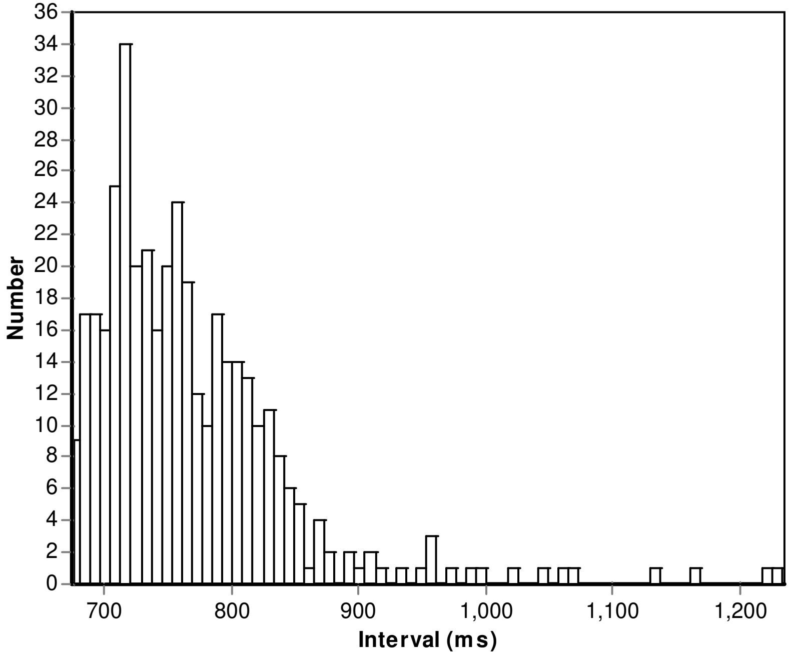

Cardio Control NV. User: Anton

Heart Rate Variability: Time Domain Analysis

**Name:**	011, 011 011	**Birthdate:**	18/01/1976
**Number:**	011	**Recorded:**	05/05/2018 12:05:08
**Gender:**	Male		

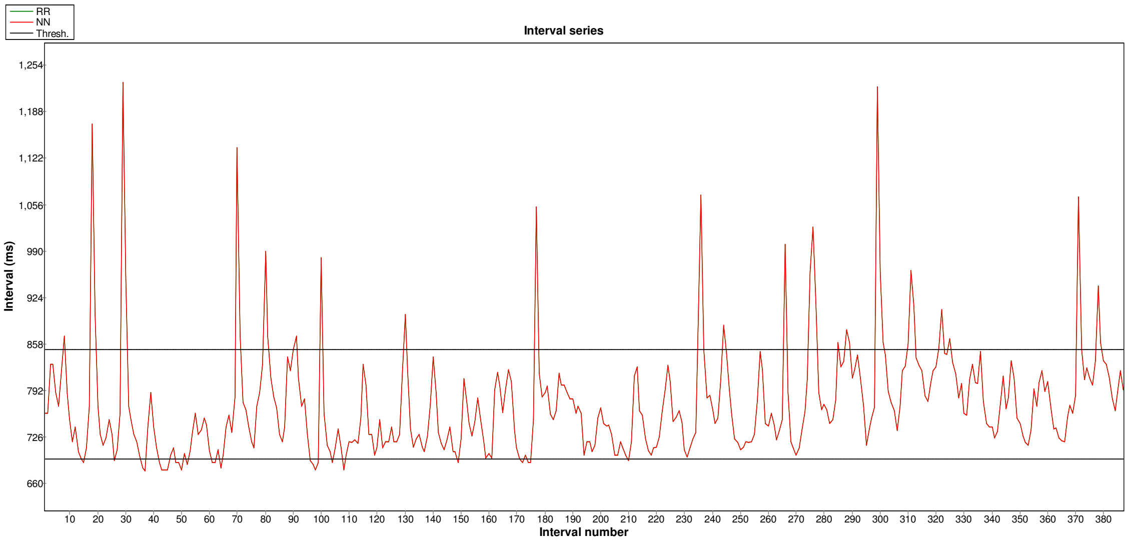

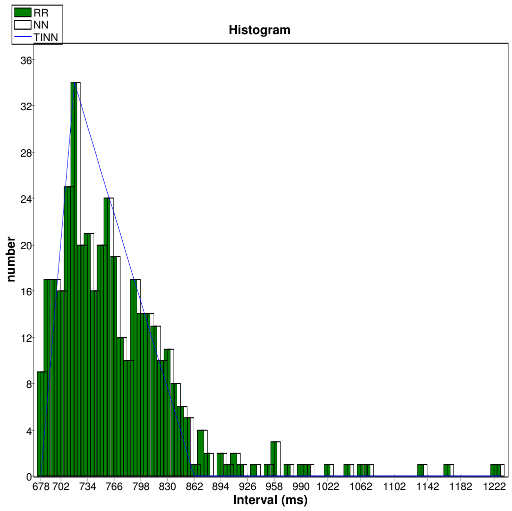

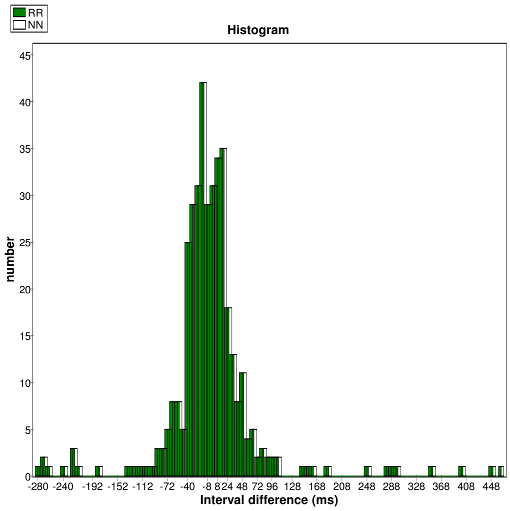

Binsize (ms) = 8

**HRV parameters**	**NN**	**RR**
SDNN (ms)	78	78
Triangular Interpolation (ms)	184	184
Triangular Index	11.38	11.38

**HRV parameters**	**NN**	**RR**
SDSD (ms)	75	75
RMSSD (ms)	75	75
NN50	77	77
NN50(1)	38	38
NN50(2)	39	39
pNN50	0.20	0.20
pNN50(1)	0.10	0.10
pNN50(2)	0.10	0.10
Logarithmic Index	0.13	0.13
SD(Logarithmic Index)	0.01	0.01

**Interval statistics**	**NN**	**RR**
Number	387	387
Minimum (ms)	678	678
Maximum (ms)	1230	1230
Range (ms)	552	552
Avg (ms)	772	772
SD (ms)	78	78
AvgDev (ms)	54	54
p5 (ms)	690	690
p50 (ms)	757	757
p95 (ms)	912	912
Skewness	2.39	2.39
Kurtosis	11.77	11.77

**Interval statistics**	**NN**	**RR**
Number	386	386
Minimum (ms)	-280	-280
Maximum (ms)	470	470
Range (ms)	750	750
Avg (ms)	0	0
SD (ms)	75	75
AvgDev (ms)	41	41
p5 (ms)	-81	-81
p50 (ms)	-4	-4
p95 (ms)	84	84
Skewness	1.62	1.62
Kurtosis	15.82	15.82

Cardio Control NV. User: Anton

Heart Rate Variability: Frequency Domain Analysis

**Name:**	011, 011 011	**Birthdate:**	18/01/1976
**Number:**	011	**Recorded:**	05/05/2018 12:05:08
**Gender:**	Male		

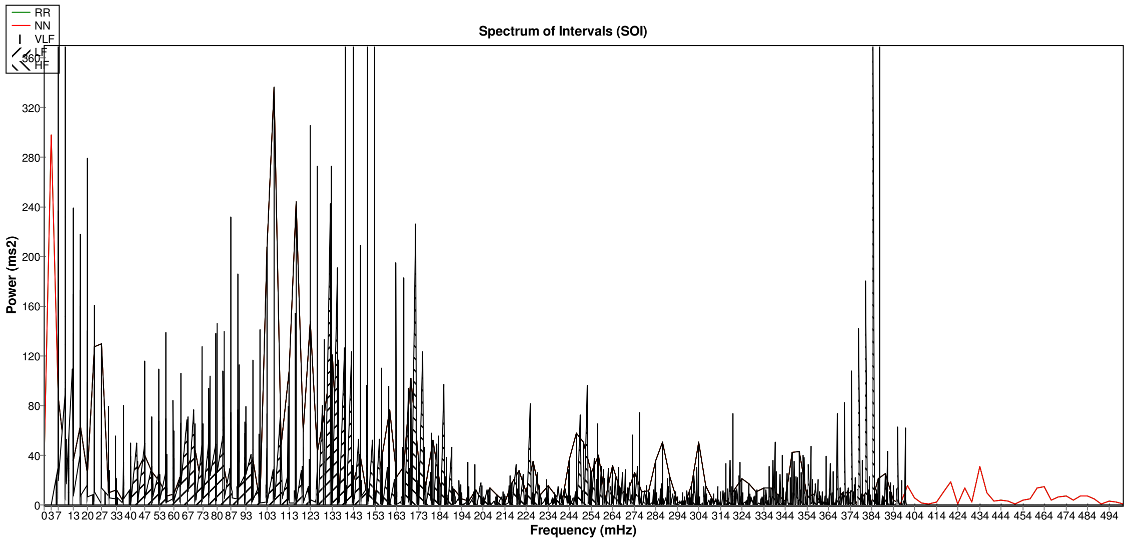

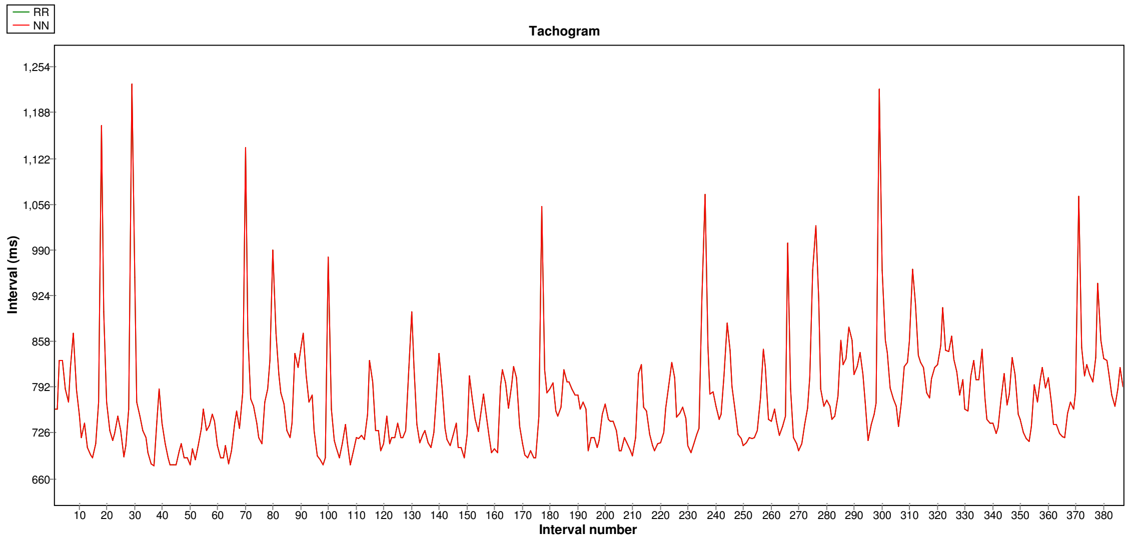

**HRV parameters**	**NN**	**RR**
TP (ms2)	3787	3787
VLF (ms2)	538	538
LF (ms2)	1832	1832
HF (ms2)	1417	1417
LF/HF	1.29	1.29
LF normalized	56.38	56.38
HF normalized	43.62	43.62
VLF peak (mHz)	27	27
LF peak (mHz)	107	107
HF peak (mHz)	170	170

**HRV spectral settings**	
Spectrum of Intervals (SOI)	
Frequency resolution (mHz)	3
VLF lower boundary (mHz)	3
VLF upper boundary (mHz)	40
LF upper boundary (mHz)	150
HF upper boundary (mHz)	400
Smoothing factor	1
Tapering	Hann
Fourier transform	DFT
Sample frequency (Hz)	1.29
Interval correction	Annotation
Interval threshold (%)	10
