# Supplementary material for: The effects of a 3-day mountain bike cycling race on the autonomic nervous system (ANS) and heart rate variability in amateur cyclists: a prospective quantitative research design
Source: BMC Sports Sci Med Rehabil. 2023 Jan 2;15:2. doi: 10.1186/s13102-022-00614-y (PMC9808932; doi:10.1186/s13102-022-00614-y)
Supplement: Supplementary file 1 — Additional file 1. Individual data of Participants. [file 13102_2022_614_MOESM1_ESM.zip › Individual data of Participants/HRV Data/010/ECG_010_20180506073807_.PDF]

Anton Swart Biokinetic Rehabilitation Practice

Name:	011 011 011	Recorded:	06/05/2018 07:38:07	UNCONFIRMED INTERPRETATION - MD SHOULD REVIEW
Number:	011	Recorded by:	Mr. Anton Swart	
Gender:	Male	Referring physician:		
Birthdate:	18/01/1976 42 years	Ordering physician:		
		Attending physician:		
P / PQ:	110 ms / 190 ms	Location:	Anton Swart Biokinetic Rehabilitation Practi	
QRS:	85 ms	Comment:		
QT / QTc / QTd:	408 ms / 427 ms / -			
P/QRS/T axis:	83° / 81° / 68°			
Heartrate:	71 bpm			

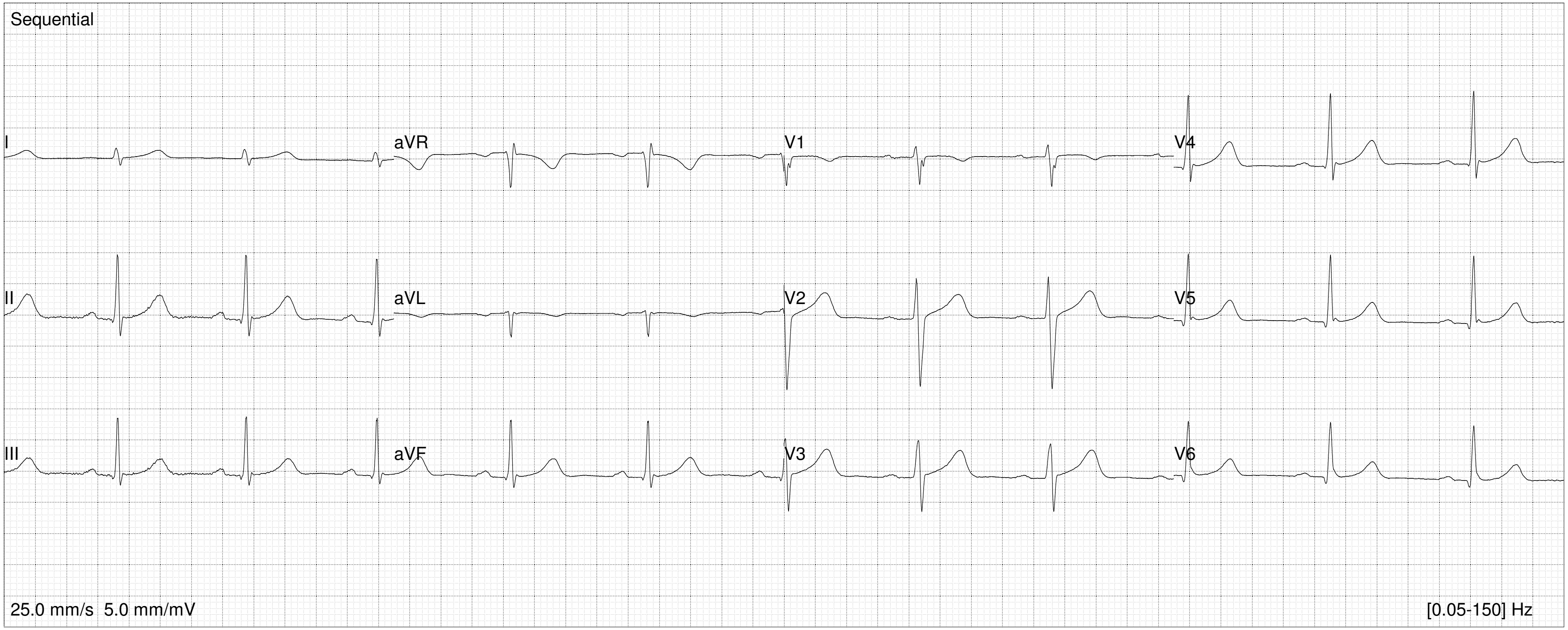

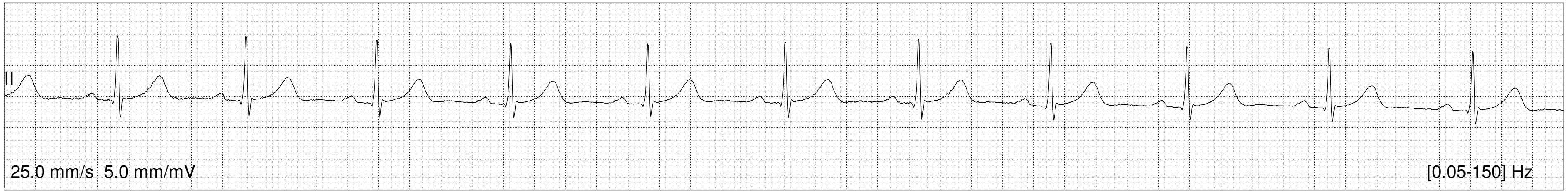

Anton Swart Biokinetic Rehabilitation Practice

Name:	011 011 011	Recorded:	06/05/2018 07:38:07	UNCONFIRMED INTERPRETATION - MD SHOULD REVIEW
Number:	011	Recorded by:	Mr. Anton Swart	
Gender:	Male	Referring physician:		
Birthdate:	18/01/1976 42 years	Location:	Anton Swart Biokinetic Rehabilitation Practice	
		Ordering physician:		
		Attending physician:		
		Comment:		

P / PQ:	110 ms / 190 ms
QRS:	85 ms
QT / QTc / QTd:	408 ms / 427 ms / -
P/QRS/T axis:	83° / 81° / 68°
Heartrate:	71 bpm

Beats

Total:	353
Normal:	353
Other:	0

RR

Minimum	740 ms
Maximum	950 ms
Mean:	847 ms
SD:	32 ms

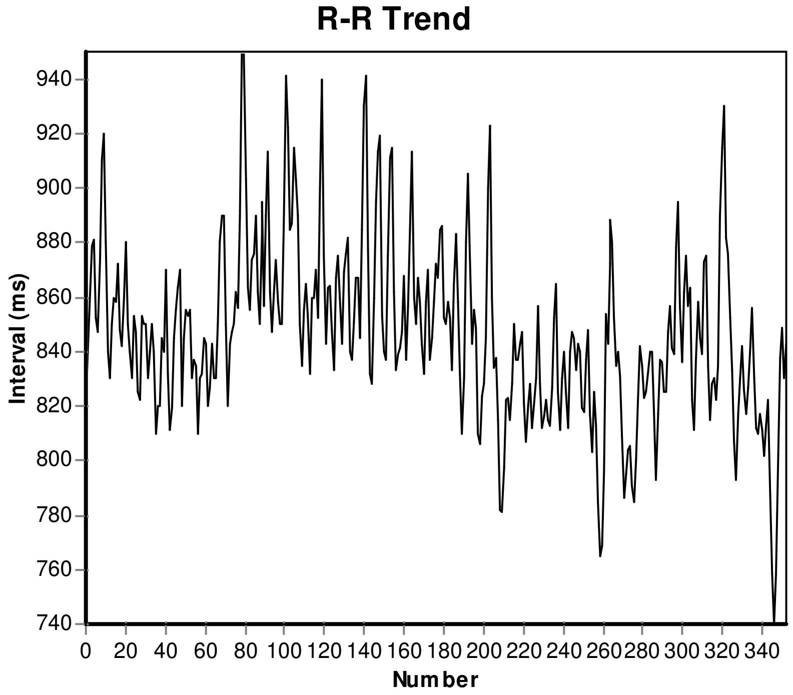

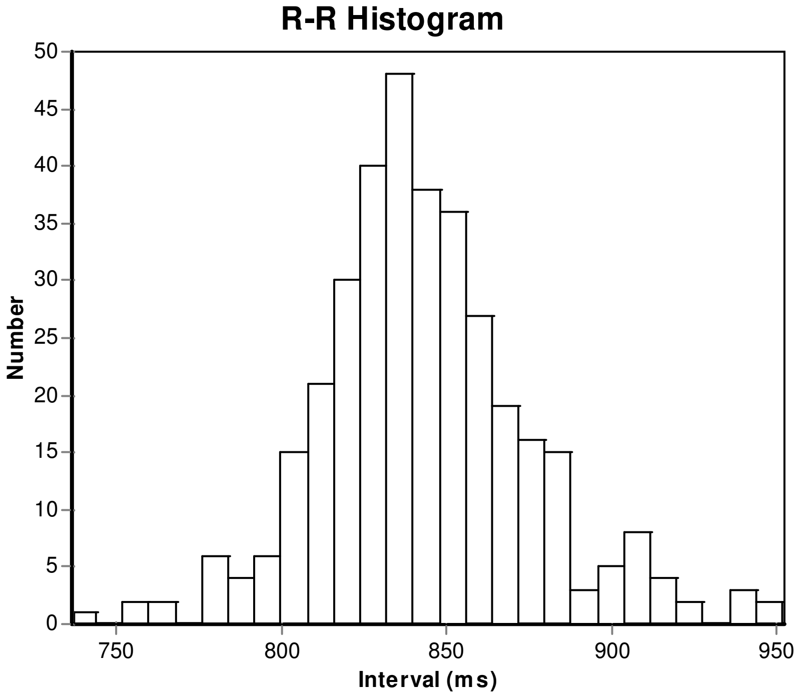

Cardio Control NV. User: Anton

Heart Rate Variability: Time Domain Analysis

**Name:**	011, 011 011	**Birthdate:**	18/01/1976
**Number:**	011	**Recorded:**	06/05/2018 07:38:07
**Gender:**	Male		

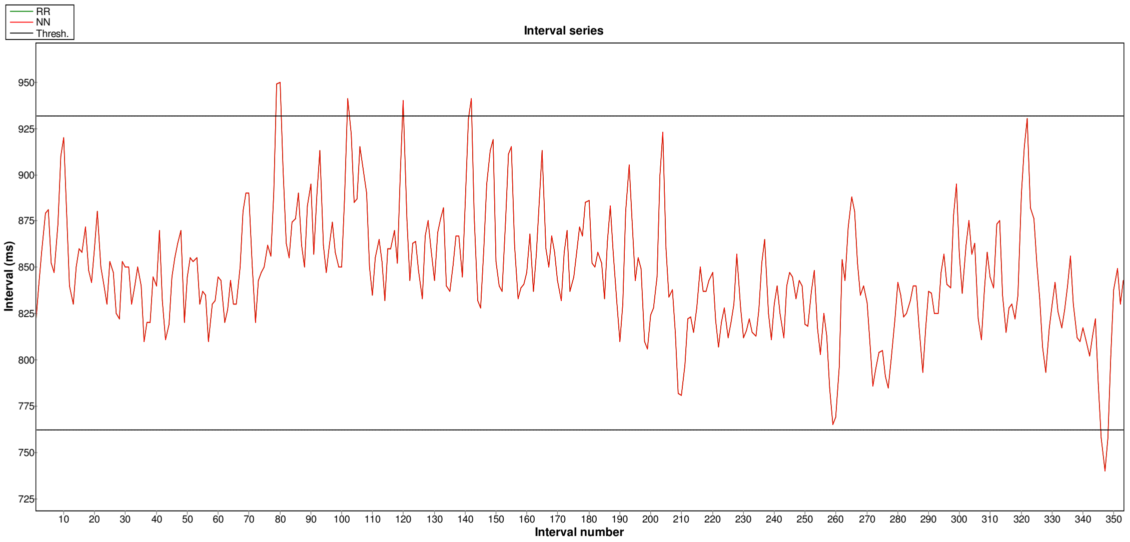

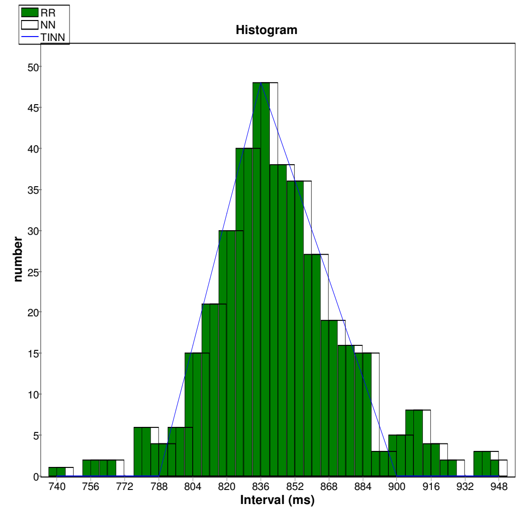

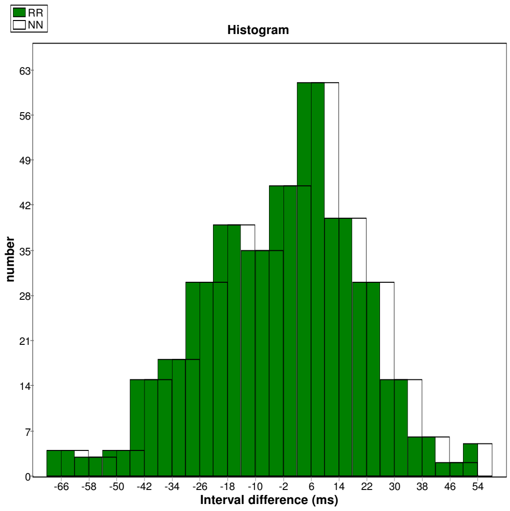

Binsize (ms) = 8

**HRV parameters**	**NN**	**RR**
SDNN (ms)	32	32
Triangular Interpolation (ms)	112	112
Triangular Index	7.35	7.35

**HRV parameters**	**NN**	**RR**
SDSD (ms)	23	23
RMSSD (ms)	23	23
NN50	12	12
NN50(1)	7	7
NN50(2)	5	5
pNN50	0.03	0.03
pNN50(1)	0.02	0.02
pNN50(2)	0.01	0.01
Logarithmic Index	0.48	0.48
SD(Logarithmic Index)	0.05	0.05

**Interval statistics**	**NN**	**RR**
Number	353	353
Minimum (ms)	740	740
Maximum (ms)	950	950
Range (ms)	210	210
Avg (ms)	847	847
SD (ms)	32	32
AvgDev (ms)	24	24
p5 (ms)	800	800
p50 (ms)	845	845
p95 (ms)	913	913
Skewness	0.36	0.36
Kurtosis	3.98	3.98

**Interval statistics**	**NN**	**RR**
Number	352	352
Minimum (ms)	-66	-66
Maximum (ms)	59	59
Range (ms)	125	125
Avg (ms)	0	0
SD (ms)	23	23
AvgDev (ms)	18	18
p5 (ms)	-40	-40
p50 (ms)	2	2
p95 (ms)	36	36
Skewness	-0.23	-0.23
Kurtosis	2.99	2.99

Cardio Control NV. User: Anton

Heart Rate Variability: Frequency Domain Analysis

**Name:**	011, 011 011	**Birthdate:**	18/01/1976
**Number:**	011	**Recorded:**	06/05/2018 07:38:07
**Gender:**	Male		

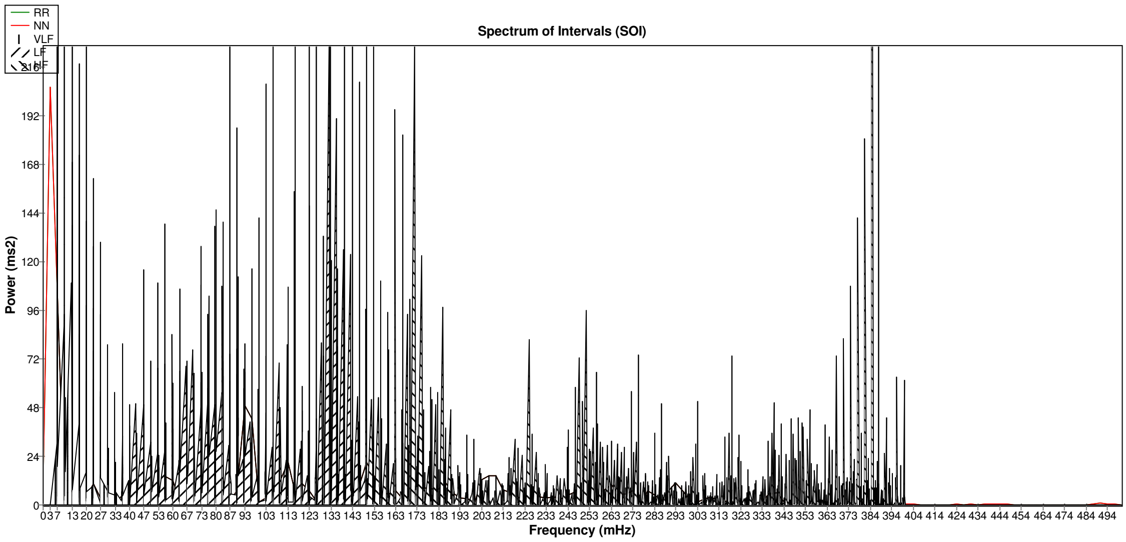

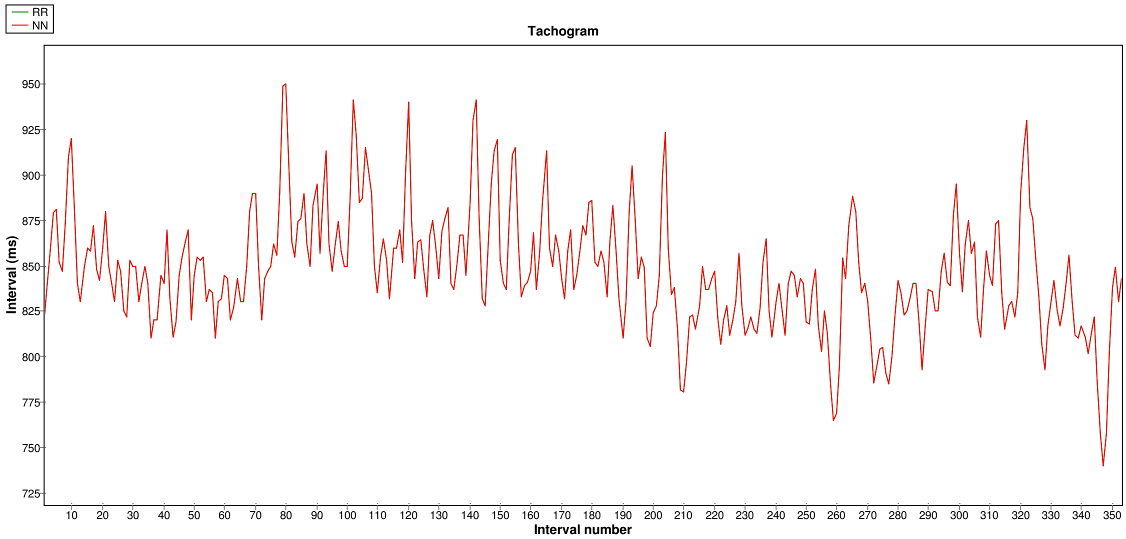

**HRV parameters**	**NN**	**RR**
TP (ms2)	804	804
VLF (ms2)	154	154
LF (ms2)	337	337
HF (ms2)	314	314
LF/HF	1.07	1.07
LF normalized	51.79	51.79
HF normalized	48.21	48.21
VLF peak (mHz)	7	7
LF peak (mHz)	93	93
HF peak (mHz)	207	207

**HRV spectral settings**	
Spectrum of Intervals (SOI)	
Frequency resolution (mHz)	3
VLF lower boundary (mHz)	3
VLF upper boundary (mHz)	40
LF upper boundary (mHz)	150
HF upper boundary (mHz)	400
Smoothing factor	1
Tapering	Hann
Fourier transform	DFT
Sample frequency (Hz)	1.18
Interval correction	Annotation
Interval threshold (%)	10
