# Supplementary material for: The effects of a 3-day mountain bike cycling race on the autonomic nervous system (ANS) and heart rate variability in amateur cyclists: a prospective quantitative research design
Source: BMC Sports Sci Med Rehabil. 2023 Jan 2;15:2. doi: 10.1186/s13102-022-00614-y (PMC9808932; doi:10.1186/s13102-022-00614-y)
Supplement: Supplementary file 1 — Additional file 1. Individual data of Participants. [file 13102_2022_614_MOESM1_ESM.zip › Individual data of Participants/HRV Data/011/ECG_011_20180501114421_.PDF]

Anton Swart Biokinetic Rehabilitation Practice

Name:	012 012 012	Recorded:	01/05/2018 11:44:21	UNCONFIRMED INTERPRETATION - MD SHOULD REVIEW
Number:	012	Recorded by:	Mr. Anton Swart	
Gender:	Male	Referring physician:		
Birthdate:	28/12/1963 54 years	Ordering physician:		
		Attending physician:		
P / PQ:	113 ms / 187 ms	Location:	Anton Swart Biokinetic Rehabilitation Practi	
QRS:	111 ms	Comment:		
QT / QTc / QTd:	415 ms / 424 ms / -			
P/QRS/T axis:	80° / 75° / 85°			
Heartrate:	65 bpm			

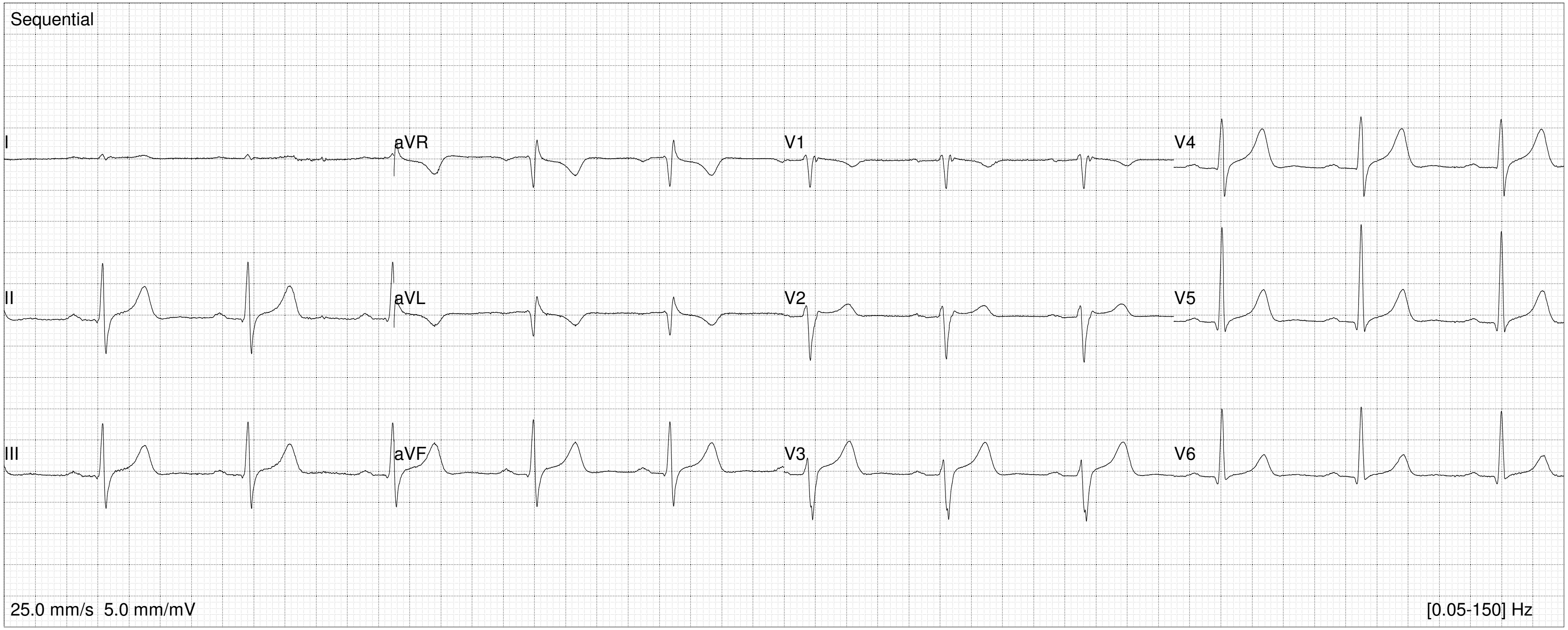

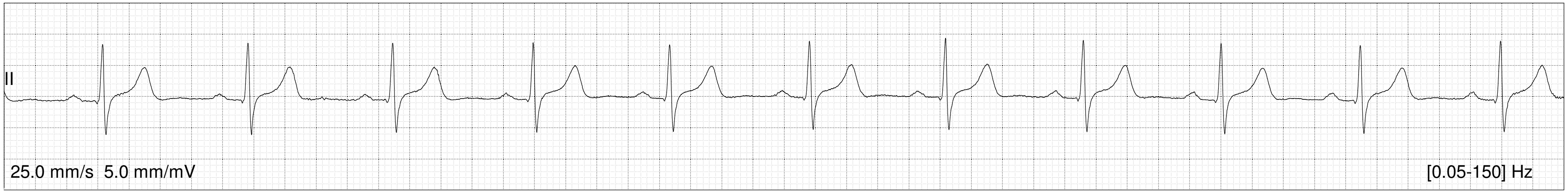

Anton Swart Biokinetic Rehabilitation Practice

Name:	012 012 012	Recorded:	01/05/2018 11:44:21	UNCONFIRMED INTERPRETATION - MD SHOULD REVIEW
Number:	012	Recorded by:	Mr. Anton Swart	
Gender:	Male	Referring physician:		
Birthdate:	28/12/1963 54 years	Location:	Anton Swart Biokinetic Rehabilitation Practice	
P / PQ:	113 ms / 187 ms	Ordering physician:		
QRS:	111 ms	Attending physician:		
QT / QTc / QTd:	415 ms / 424 ms / -	Comment:		
P/QRS/T axis:	80° / 75° / 85°			
Heartrate:	65 bpm			

**Beats**		**RR**	
Total:	324	Minimum	860 ms
Normal:	324	Maximum	976 ms
Other:	0	Mean:	921 ms
		SD:	20 ms

R-R Trend

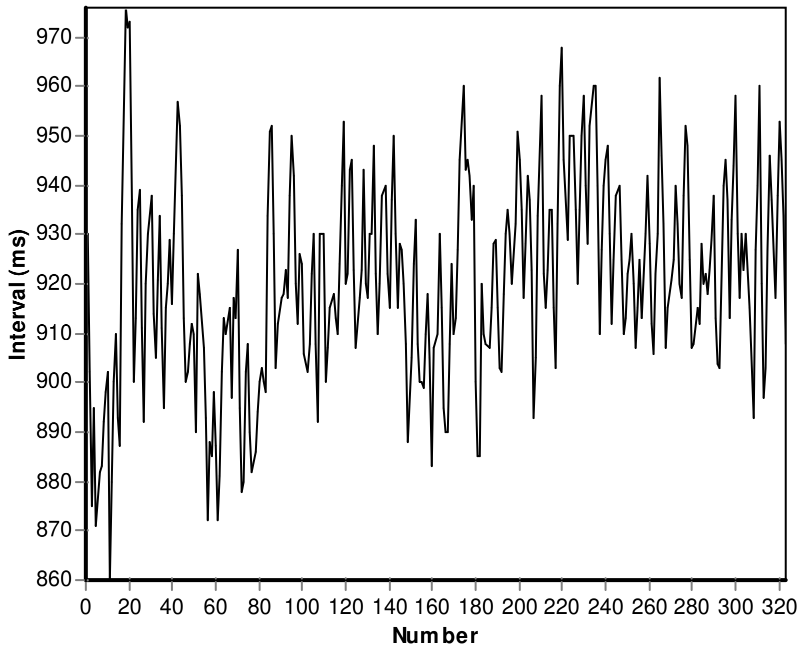

R-R Histogram

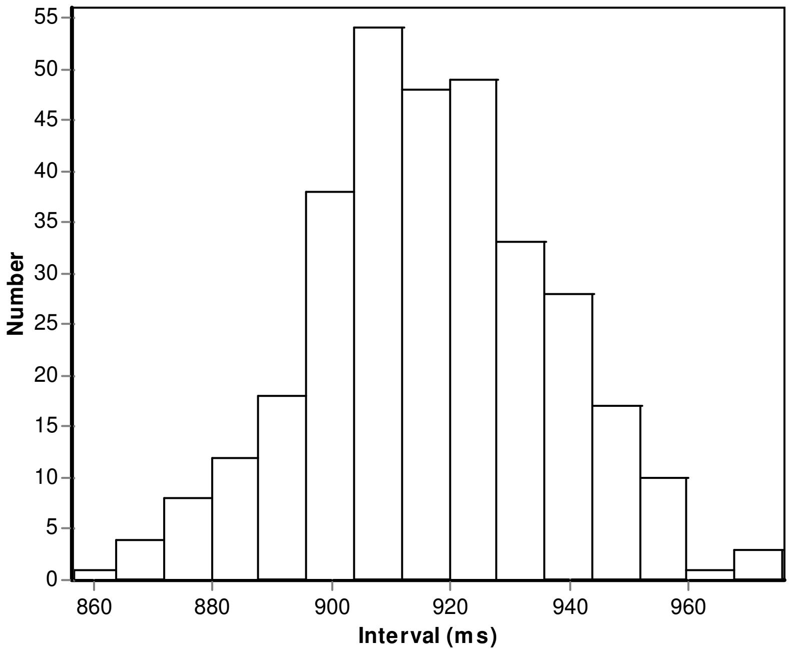

Cardio Control NV. User: Anton

Heart Rate Variability: Time Domain Analysis

**Name:**	012, 012 012	**Birthdate:**	28/12/1963
**Number:**	012	**Recorded:**	01/05/2018 11:44:21
**Gender:**	Male		

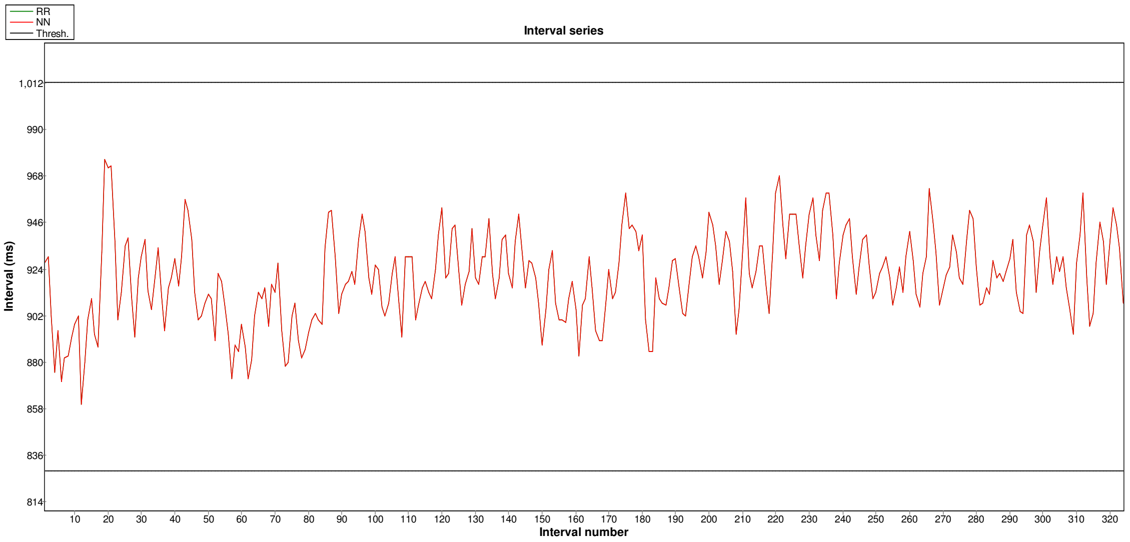

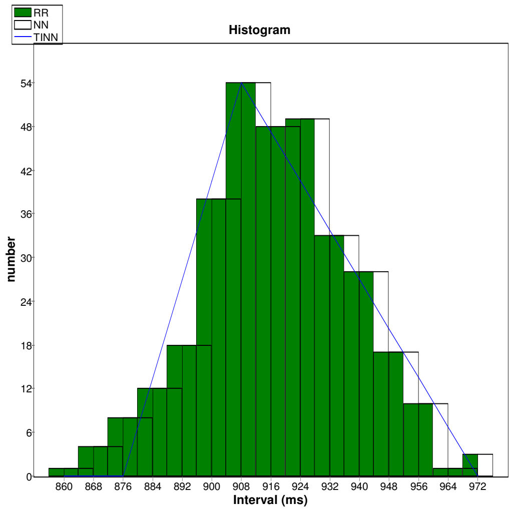

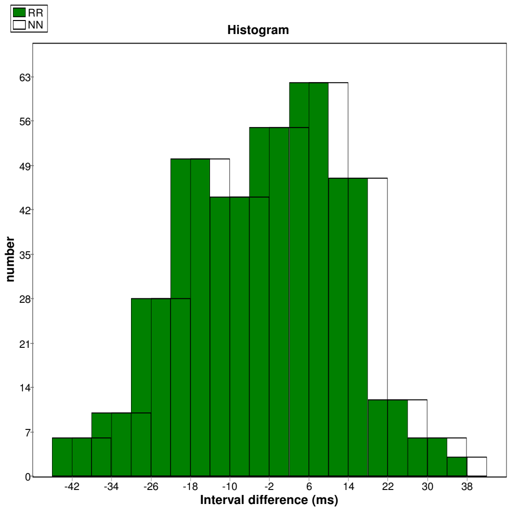

Binsize (ms) = 8

**HRV parameters**	**NN**	**RR**
SDNN (ms)	20	20
Triangular Interpolation (ms)	96	96
Triangular Index	6.00	6.00

**HRV parameters**	**NN**	**RR**
SDSD (ms)	16	16
RMSSD (ms)	16	16
NN50	0	0
NN50(1)	0	0
NN50(2)	0	0
pNN50	0.00	0.00
pNN50(1)	0.00	0.00
pNN50(2)	0.00	0.00
Logarithmic Index	0.64	0.64
SD(Logarithmic Index)	0.10	0.10

**Interval statistics**	**NN**	**RR**
Number	324	324
Minimum (ms)	860	860
Maximum (ms)	976	976
Range (ms)	116	116
Avg (ms)	921	921
SD (ms)	20	20
AvgDev (ms)	16	16
p5 (ms)	886	886
p50 (ms)	920	920
p95 (ms)	953	953
Skewness	-0.04	-0.04
Kurtosis	2.91	2.91

**Interval statistics**	**NN**	**RR**
Number	323	323
Minimum (ms)	-42	-42
Maximum (ms)	45	45
Range (ms)	87	87
Avg (ms)	-0	-0
SD (ms)	16	16
AvgDev (ms)	13	13
p5 (ms)	-26	-26
p50 (ms)	1	1
p95 (ms)	24	24
Skewness	-0.07	-0.07
Kurtosis	2.64	2.64

Heart Rate Variability: Frequency Domain Analysis

**Name:**	012, 012 012	**Birthdate:**	28/12/1963
**Number:**	012	**Recorded:**	01/05/2018 11:44:21
**Gender:**	Male		

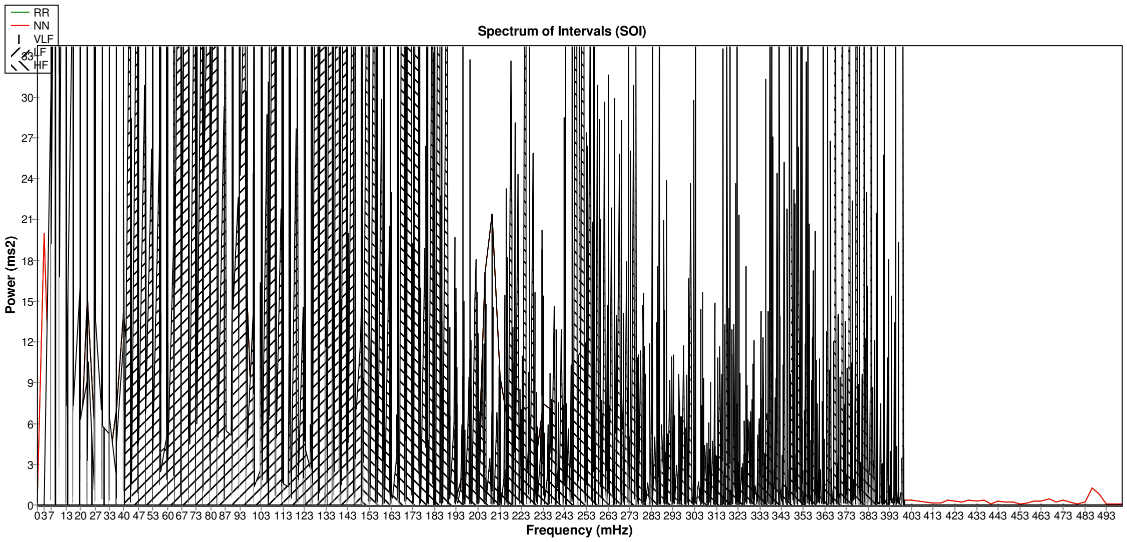

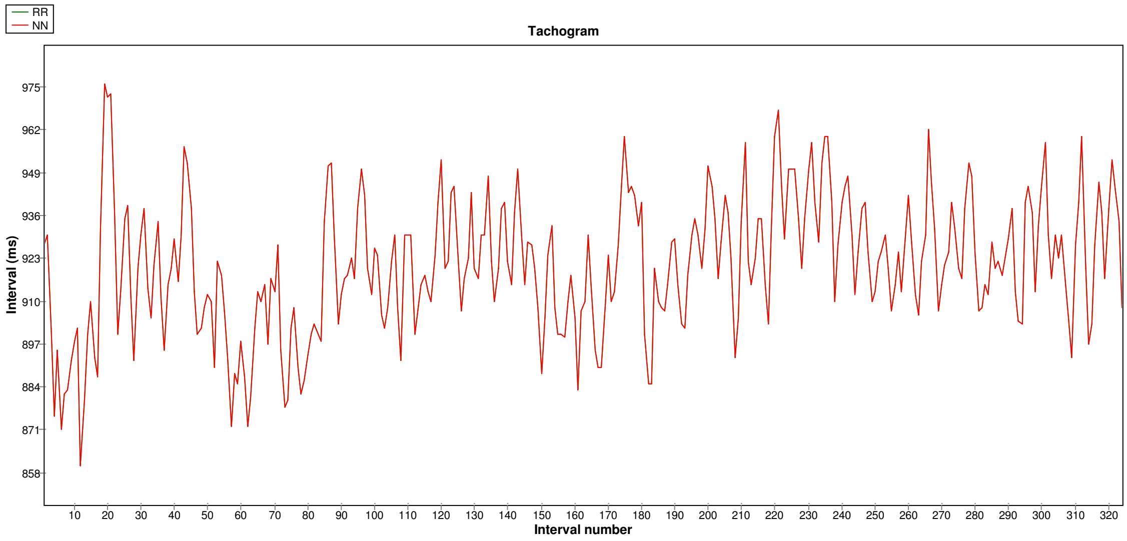

**HRV parameters**	**NN**	**RR**
TP (ms2)	297	297
VLF (ms2)	92	92
LF (ms2)	84	84
HF (ms2)	121	121
LF/HF	0.70	0.70
LF normalized	41.11	41.11
HF normalized	58.89	58.89
VLF peak (mHz)	10	10
LF peak (mHz)	97	97
HF peak (mHz)	210	210

**HRV spectral settings**	
Spectrum of Intervals (SOI)	
Frequency resolution (mHz)	3
VLF lower boundary (mHz)	3
VLF upper boundary (mHz)	40
LF upper boundary (mHz)	150
HF upper boundary (mHz)	400
Smoothing factor	1
Tapering	Hann
Fourier transform	DFT
Sample frequency (Hz)	1.09
Interval correction	Annotation
Interval threshold (%)	10
