# Supplementary material for: The effects of a 3-day mountain bike cycling race on the autonomic nervous system (ANS) and heart rate variability in amateur cyclists: a prospective quantitative research design
Source: BMC Sports Sci Med Rehabil. 2023 Jan 2;15:2. doi: 10.1186/s13102-022-00614-y (PMC9808932; doi:10.1186/s13102-022-00614-y)
Supplement: Supplementary file 1 — Additional file 1. Individual data of Participants. [file 13102_2022_614_MOESM1_ESM.zip › Individual data of Participants/HRV Data/011/ECG_011_20180503162515_.PDF]

Anton Swart Biokinetic Rehabilitation Practice

Name:	012 012 012	Recorded:	03/05/2018 16:25:15	UNCONFIRMED INTERPRETATION - MD SHOULD REVIEW
Number:	012	Recorded by:	Mr. Anton Swart	
Gender:	Male	Referring physician:		
Birthdate:	28/12/1963 54 years	Ordering physician:		
		Attending physician:		
P / PQ:	112 ms / 193 ms	Location:	Anton Swart Biokinetic Rehabilitation Practi	
QRS:	114 ms	Comment:		
QT / QTc / QTd:	395 ms / 425 ms / -			
P/QRS/T axis:	83° / 71° / 84°			
Heartrate:	77 bpm			

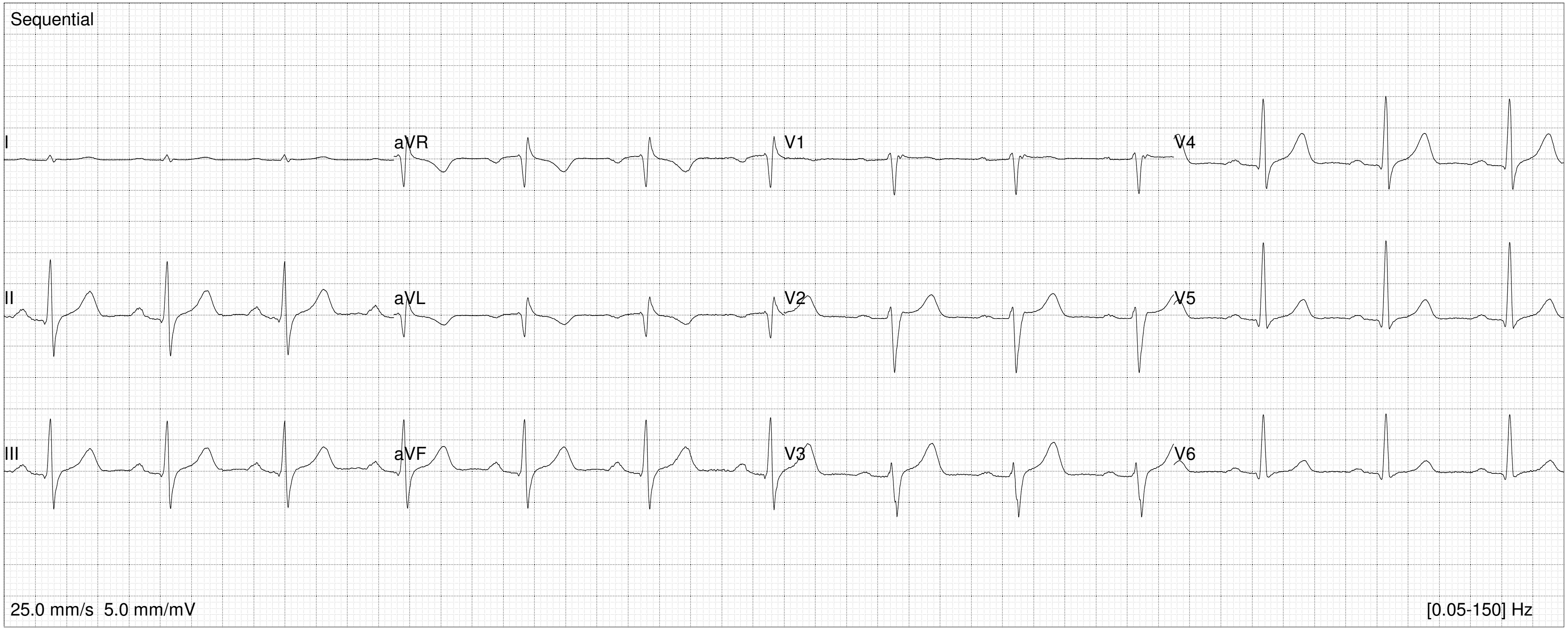

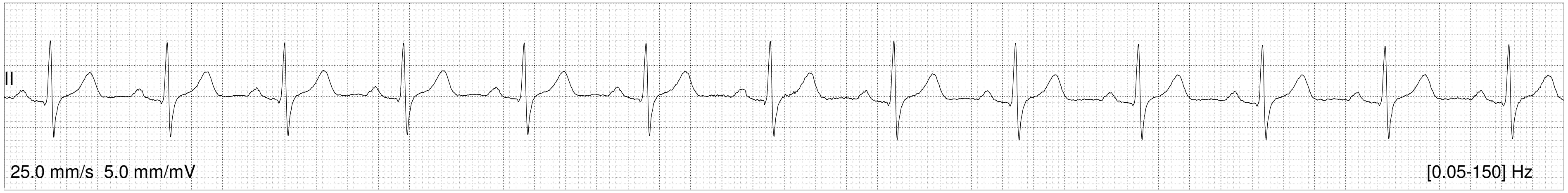

Anton Swart Biokinetic Rehabilitation Practice

Name:	012 012 012	Recorded:	03/05/2018 16:25:15	UNCONFIRMED INTERPRETATION - MD SHOULD REVIEW
Number:	012	Recorded by:	Mr. Anton Swart	
Gender:	Male	Referring physician:		
Birthdate:	28/12/1963 54 years	Location:	Anton Swart Biokinetic Rehabilitation Practice	
P / PQ:	112 ms / 193 ms	Ordering physician:		
QRS:	114 ms	Attending physician:		
QT / QTc / QTd:	395 ms / 425 ms / -	Comment:		
P/QRS/T axis:	83° / 71° / 84°			
Heartrate:	77 bpm			

Beats

Total:	383
Normal:	383
Other:	0

RR

Minimum	737 ms
Maximum	825 ms
Mean:	780 ms
SD:	16 ms

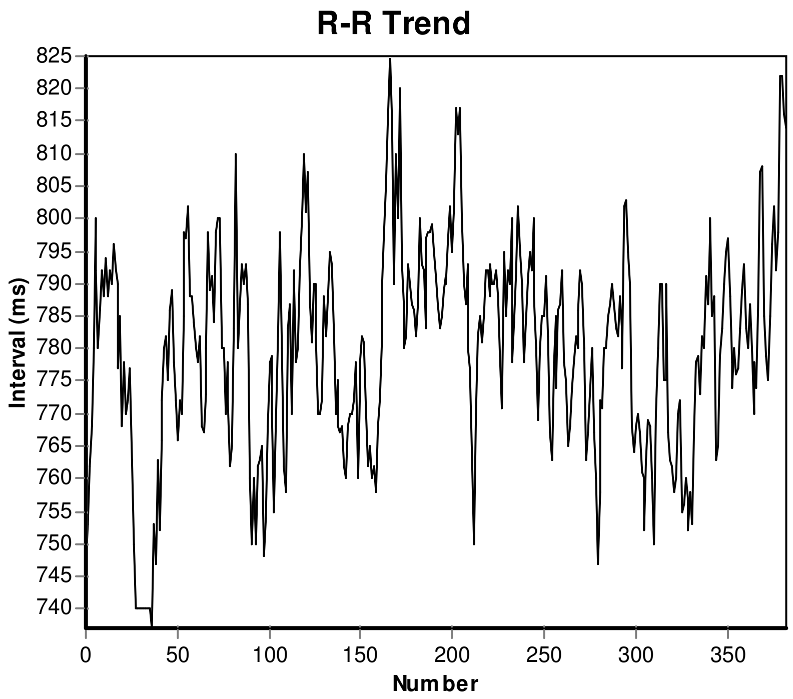

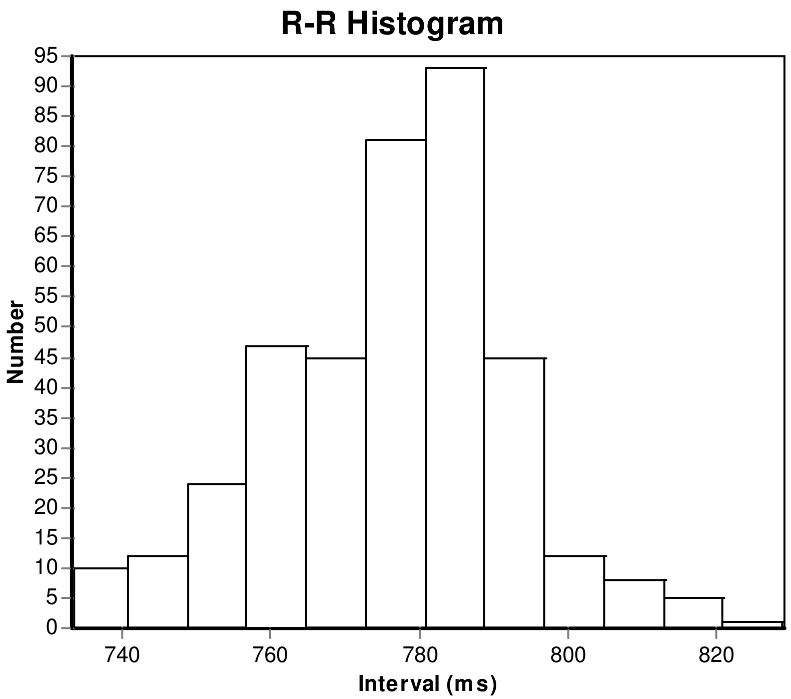

Heart Rate Variability: Time Domain Analysis

Name: 012, 012 012 **Birthdate:** 28/12/1963
Number: 012 **Recorded:** 03/05/2018 16:25:15
Gender: Male

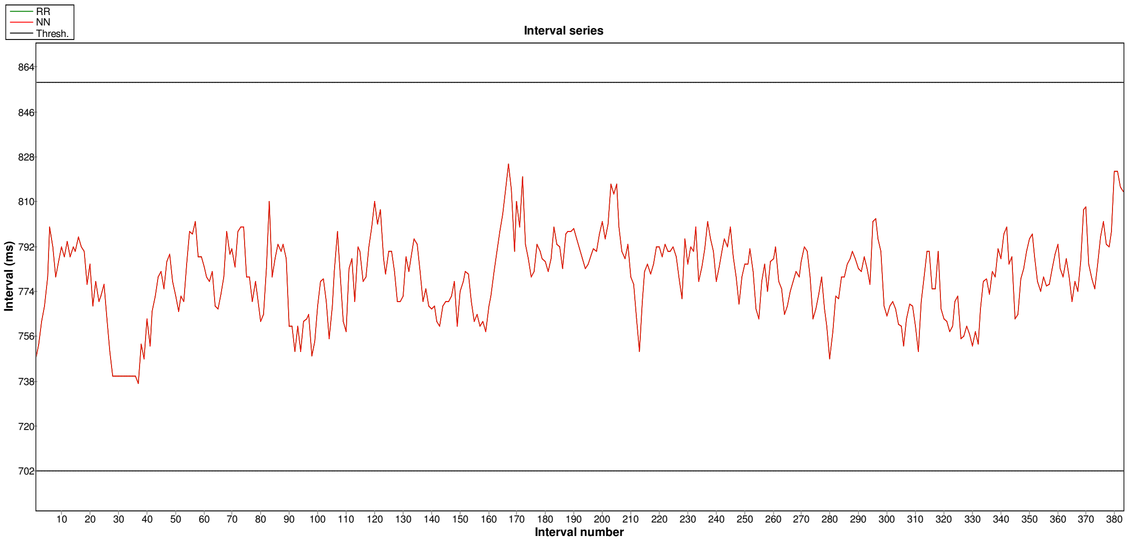

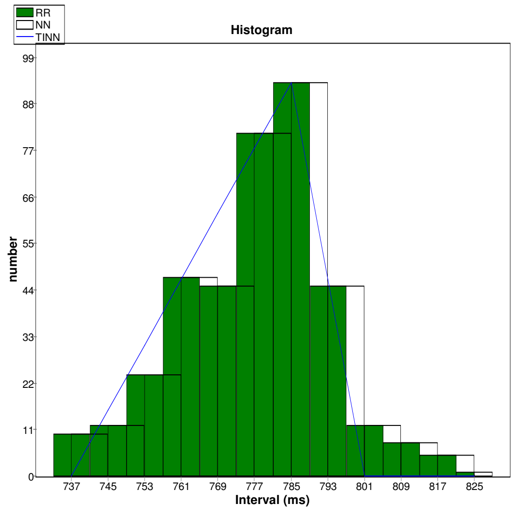

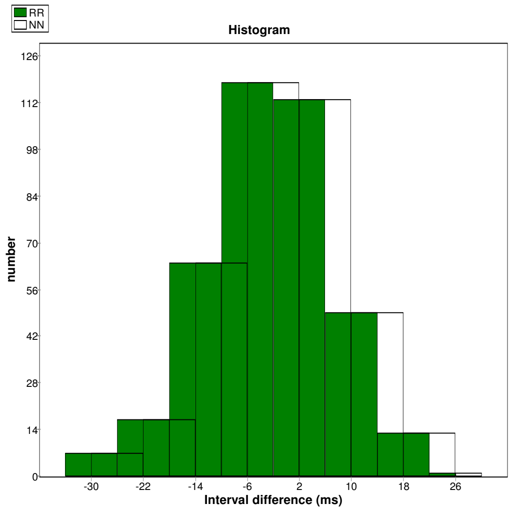

Binsize (ms) = 8

HRV parameters	NN	RR
SDNN (ms)	16	16
Triangular Interpolation (ms)	64	64
Triangular Index	4.12	4.12

HRV parameters	NN	RR
SDSD (ms)	10	10
RMSSD (ms)	10	10
NN50	0	0
NN50(1)	0	0
NN50(2)	0	0
pNN50	0.00	0.00
pNN50(1)	0.00	0.00
pNN50(2)	0.00	0.00
Logarithmic Index	1.08	1.08
SD(Logarithmic Index)	0.10	0.10

Interval statistics	NN	RR
Number	383	383
Minimum (ms)	737	737
Maximum (ms)	825	825
Range (ms)	88	88
Avg (ms)	780	780
SD (ms)	16	16
AvgDev (ms)	13	13
p5 (ms)	752	752
p50 (ms)	782	782
p95 (ms)	803	803
Skewness	-0.20	-0.20
Kurtosis	3.18	3.18

Interval statistics	NN	RR
Number	382	382
Minimum (ms)	-30	-30
Maximum (ms)	27	27
Range (ms)	57	57
Avg (ms)	0	0
SD (ms)	10	10
AvgDev (ms)	8	8
p5 (ms)	-17	-17
p50 (ms)	0	0
p95 (ms)	16	16
Skewness	-0.14	-0.14
Kurtosis	3.24	3.24

Cardio Control NV. User: Anton

Heart Rate Variability: Frequency Domain Analysis

**Name:**	012, 012 012	**Birthdate:**	28/12/1963
**Number:**	012	**Recorded:**	03/05/2018 16:25:15
**Gender:**	Male		

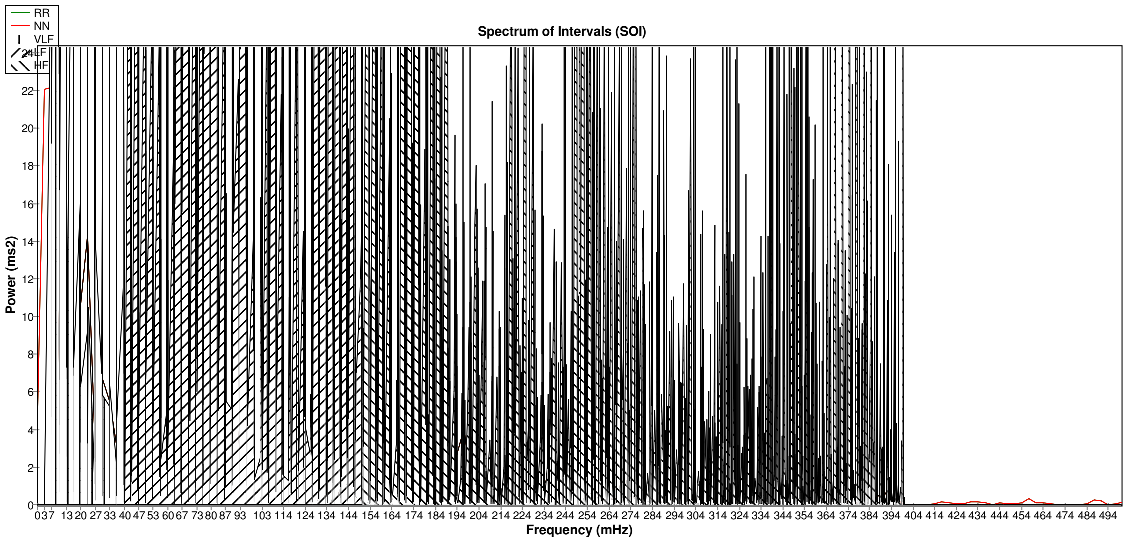

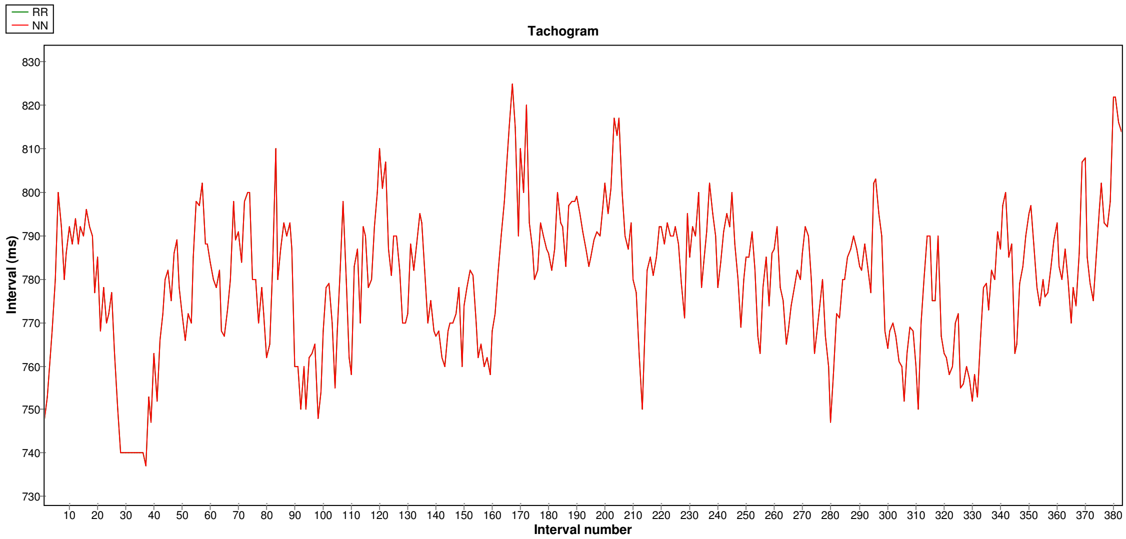

HRV parameters	NN	RR
TP (ms2)	191	191
VLF (ms2)	81	81
LF (ms2)	75	75
HF (ms2)	35	35
LF/HF	2.17	2.17
LF normalized	68.50	68.50
HF normalized	31.50	31.50
VLF peak (mHz)	7	7
LF peak (mHz)	77	77
HF peak (mHz)	197	197

HRV spectral settings	
Spectrum of Intervals (SOI)	
Frequency resolution (mHz)	3
VLF lower boundary (mHz)	3
VLF upper boundary (mHz)	40
LF upper boundary (mHz)	150
HF upper boundary (mHz)	400
Smoothing factor	1
Tapering	Hann
Fourier transform	DFT
Sample frequency (Hz)	1.28
Interval correction	Annotation
Interval threshold (%)	10
