# Supplementary material for: The effects of a 3-day mountain bike cycling race on the autonomic nervous system (ANS) and heart rate variability in amateur cyclists: a prospective quantitative research design
Source: BMC Sports Sci Med Rehabil. 2023 Jan 2;15:2. doi: 10.1186/s13102-022-00614-y (PMC9808932; doi:10.1186/s13102-022-00614-y)
Supplement: Supplementary file 1 — Additional file 1. Individual data of Participants. [file 13102_2022_614_MOESM1_ESM.zip › Individual data of Participants/HRV Data/011/ECG_011_20180504125835_.PDF]

Anton Swart Biokinetic Rehabilitation Practice

Name:	012 012 012	Recorded:	04/05/2018 12:58:35	UNCONFIRMED INTERPRETATION - MD SHOULD REVIEW
Number:	012	Recorded by:	Mr. Anton Swart	
Gender:	Male	Referring physician:		
Birthdate:	28/12/1963 54 years	Ordering physician:		
		Attending physician:		
P / PQ:	120 ms / 202 ms	Location:	Anton Swart Biokinetic Rehabilitation Practi	
QRS:	128 ms	Comment:		
QT / QTc / QTd:	383 ms / 413 ms / -			
P/QRS/T axis:	84° / 80° / 85°			
Heartrate:	77 bpm			

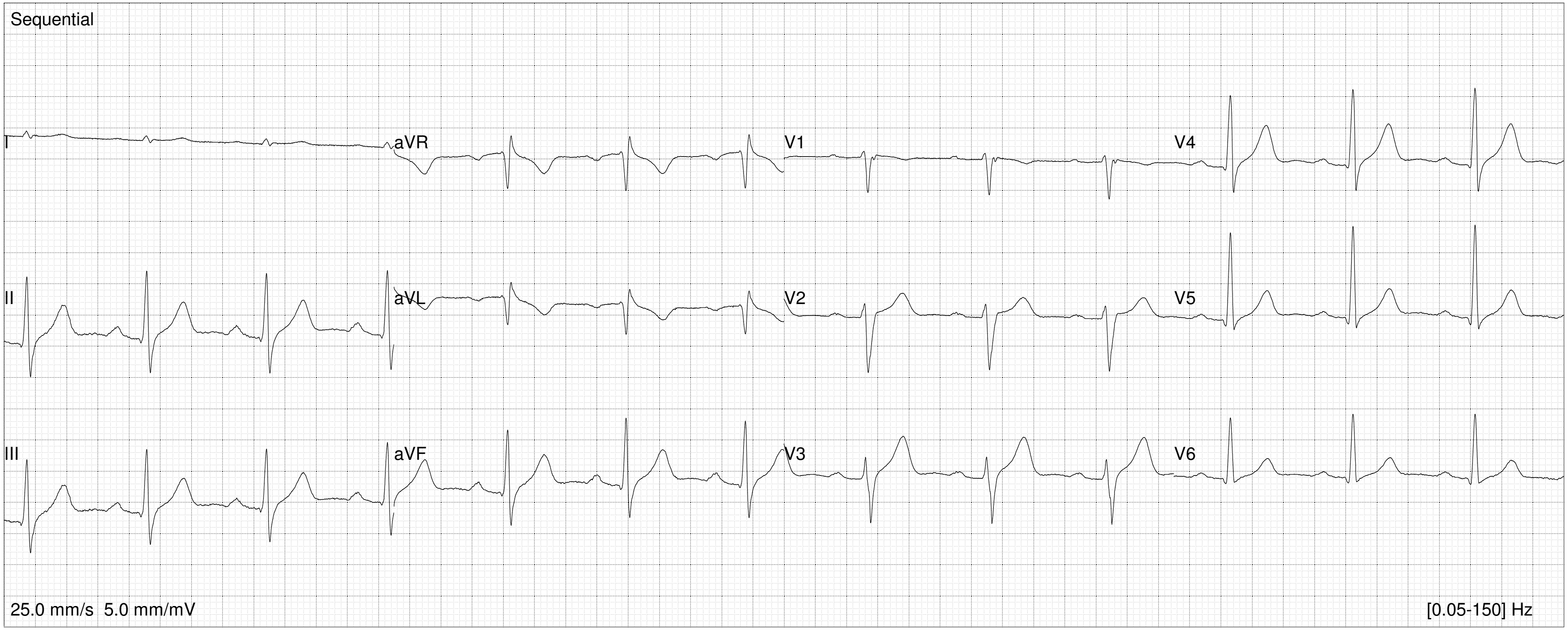

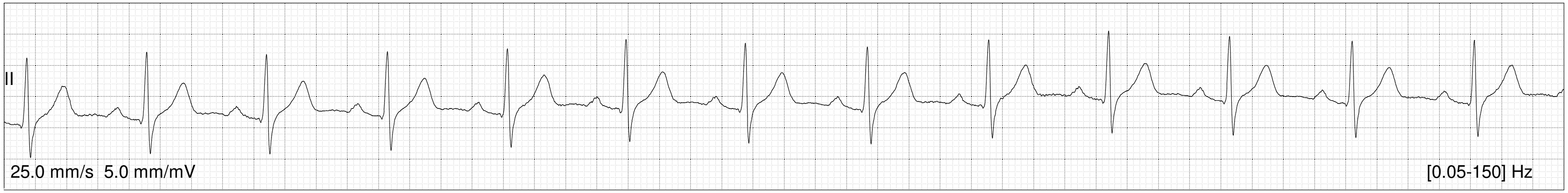

Anton Swart Biokinetic Rehabilitation Practice

Name:	012 012 012	Recorded:	04/05/2018 12:58:35	UNCONFIRMED INTERPRETATION - MD SHOULD REVIEW
Number:	012	Recorded by:	Mr. Anton Swart	
Gender:	Male	Referring physician:		
Birthdate:	28/12/1963 54 years	Location:	Anton Swart Biokinetic Rehabilitation Practice	
		Ordering physician:		
		Attending physician:		
		Comment:		

P / PQ:	120 ms / 202 ms
QRS:	128 ms
QT / QTc / QTd:	383 ms / 413 ms / -
P/QRS/T axis:	84° / 80° / 85°
Heartrate:	77 bpm

Beats

Total:	389
Normal:	389
Other:	0

RR

Minimum	690 ms
Maximum	857 ms
Mean:	769 ms
SD:	30 ms

R-R Trend

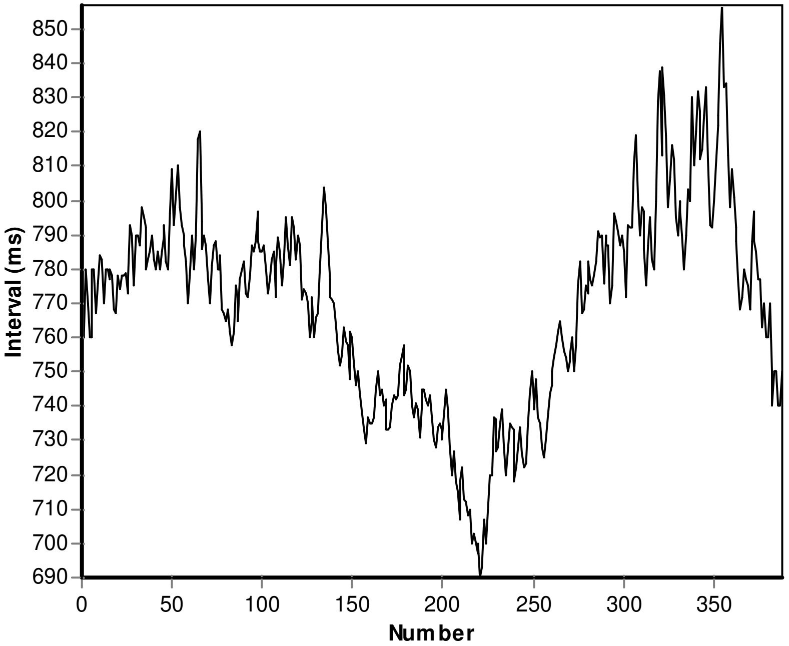

R-R Histogram

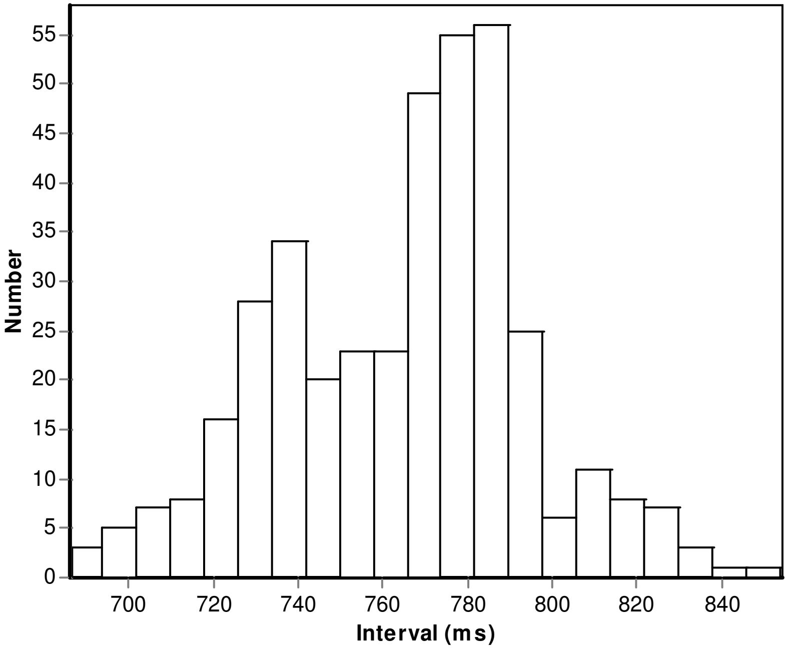

Cardio Control NV. User: Anton

Heart Rate Variability: Time Domain Analysis

**Name:**	012, 012 012	**Birthdate:**	28/12/1963
**Number:**	012	**Recorded:**	04/05/2018 12:58:35
**Gender:**	Male		

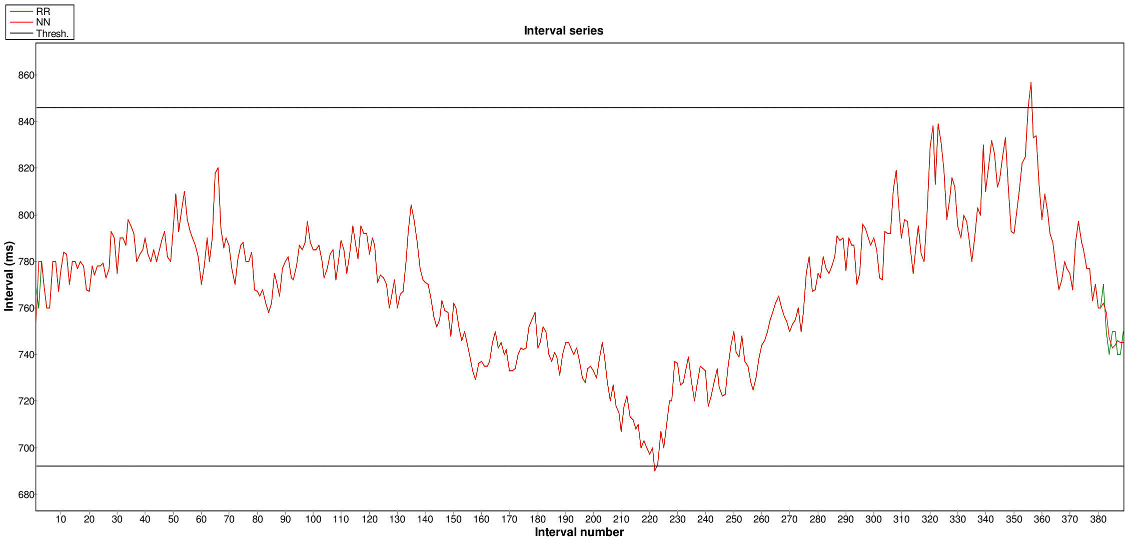

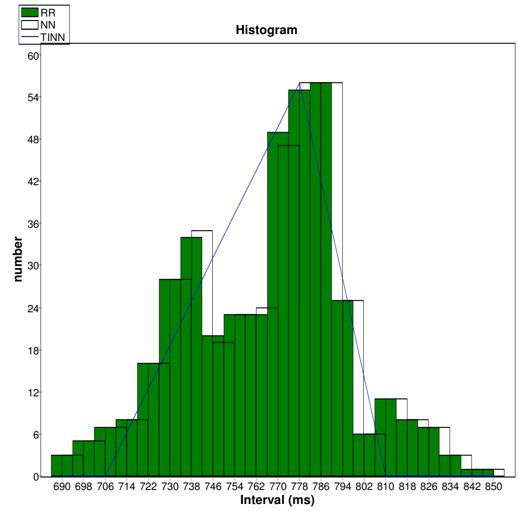

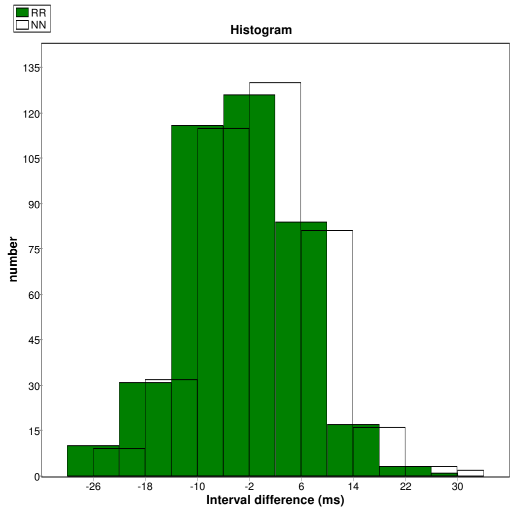

Binsize (ms) = 8

**HRV parameters**	**NN**	**RR**
SDNN (ms)	30	30
Triangular Interpolation (ms)	104	104
Triangular Index	6.95	6.95

**HRV parameters**	**NN**	**RR**
SDSD (ms)	9	9
RMSSD (ms)	9	9
NN50	0	0
NN50(1)	0	0
NN50(2)	0	0
pNN50	0.00	0.00
pNN50(1)	0.00	0.00
pNN50(2)	0.00	0.00
Logarithmic Index	1.18	1.21
SD(Logarithmic Index)	0.16	0.17

**Interval statistics**	**NN**	**RR**
Number	389	389
Minimum (ms)	690	690
Maximum (ms)	857	857
Range (ms)	167	167
Avg (ms)	769	769
SD (ms)	30	30
AvgDev (ms)	24	24
p5 (ms)	720	720
p50 (ms)	775	775
p95 (ms)	819	819
Skewness	-0.14	-0.14
Kurtosis	2.85	2.85

**Interval statistics**	**NN**	**RR**
Number	388	388
Minimum (ms)	-26	-26
Maximum (ms)	30	30
Range (ms)	56	56
Avg (ms)	-0	-0
SD (ms)	9	9
AvgDev (ms)	7	7
p5 (ms)	-15	-15
p50 (ms)	0	0
p95 (ms)	14	14
Skewness	0.23	0.15
Kurtosis	3.63	3.39

Cardio Control NV. User: Anton

Heart Rate Variability: Frequency Domain Analysis

**Name:**	012, 012 012	**Birthdate:**	28/12/1963
**Number:**	012	**Recorded:**	04/05/2018 12:58:35
**Gender:**	Male		

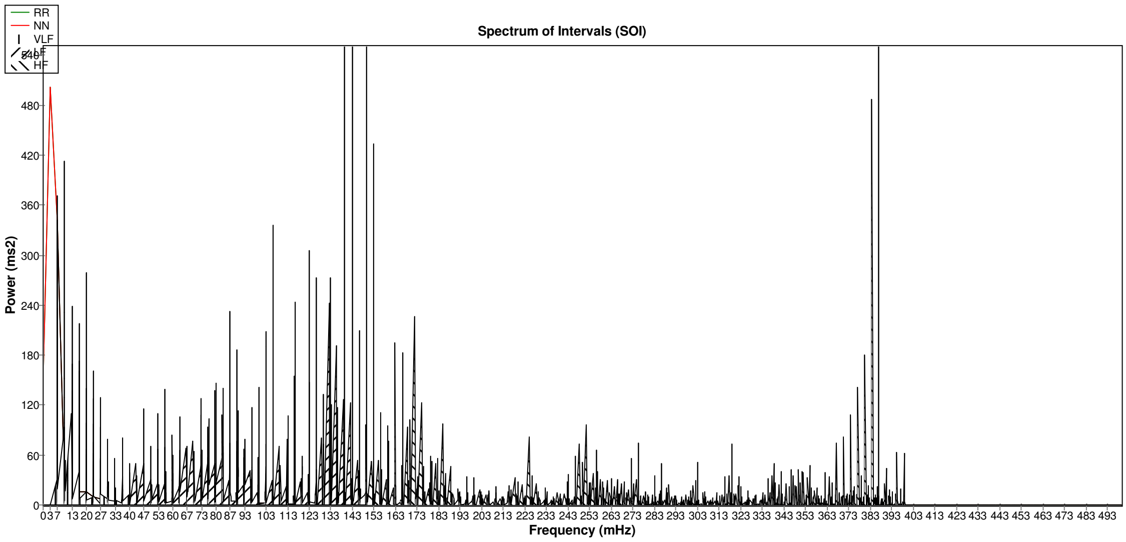

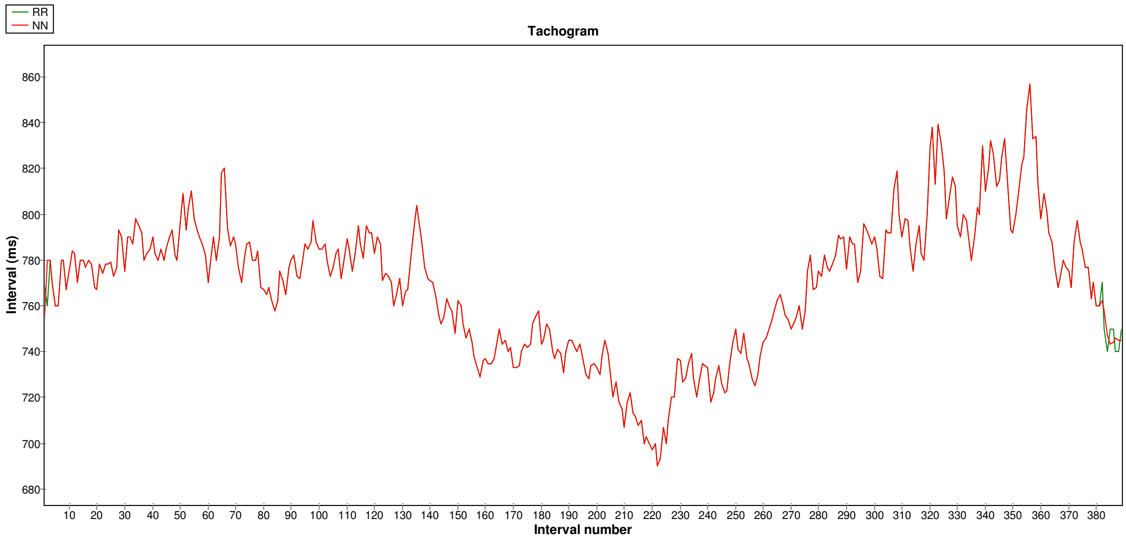

HRV parameters	NN	RR
TP (ms2)	507	507
VLF (ms2)	440	440
LF (ms2)	49	49
HF (ms2)	18	18
LF/HF	2.78	2.78
LF normalized	73.57	73.57
HF normalized	26.43	26.43
VLF peak (mHz)	7	7
LF peak (mHz)	80	80
HF peak (mHz)	320	320

HRV spectral settings	
Spectrum of Intervals (SOI)	
Frequency resolution (mHz)	3
VLF lower boundary (mHz)	3
VLF upper boundary (mHz)	40
LF upper boundary (mHz)	150
HF upper boundary (mHz)	400
Smoothing factor	1
Tapering	Hann
Fourier transform	DFT
Sample frequency (Hz)	1.30
Interval correction	Annotation
Interval threshold (%)	10
