# Supplementary material for: The effects of a 3-day mountain bike cycling race on the autonomic nervous system (ANS) and heart rate variability in amateur cyclists: a prospective quantitative research design
Source: BMC Sports Sci Med Rehabil. 2023 Jan 2;15:2. doi: 10.1186/s13102-022-00614-y (PMC9808932; doi:10.1186/s13102-022-00614-y)
Supplement: Supplementary file 1 — Additional file 1. Individual data of Participants. [file 13102_2022_614_MOESM1_ESM.zip › Individual data of Participants/HRV Data/011/ECG_011_20180505121140_.PDF]

Anton Swart Biokinetic Rehabilitation Practice

Name:	012 012 012	Recorded:	05/05/2018 12:11:40	UNCONFIRMED INTERPRETATION - MD SHOULD REVIEW
Number:	012	Recorded by:	Mr. Anton Swart	
Gender:	Male	Referring physician:		
Birthdate:	28/12/1963 54 years	Ordering physician:		
		Attending physician:		
P / PQ:	113 ms / 175 ms	Location:	Anton Swart Biokinetic Rehabilitation Practi	
QRS:	118 ms	Comment:		
QT / QTc / QTd:	375 ms / 436 ms / -			
P/QRS/T axis:	85° / 77° / 85°			
Heartrate:	95 bpm			

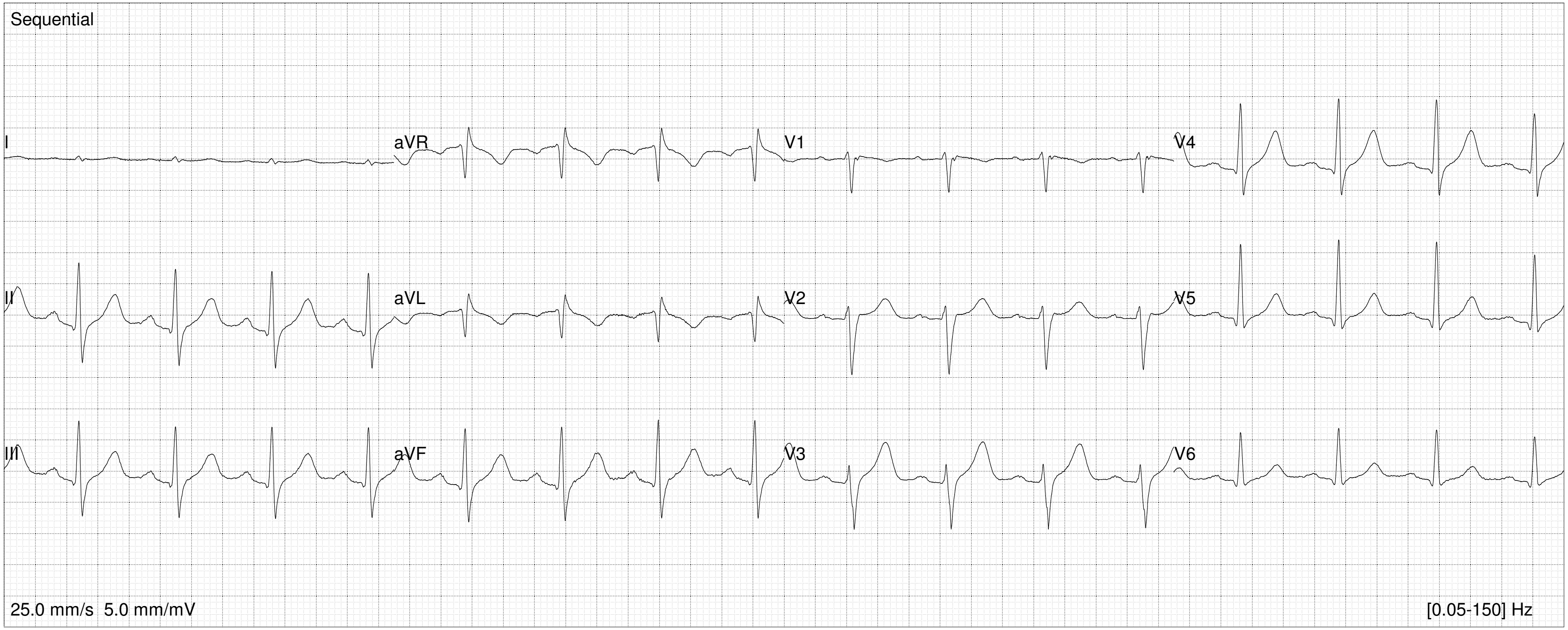

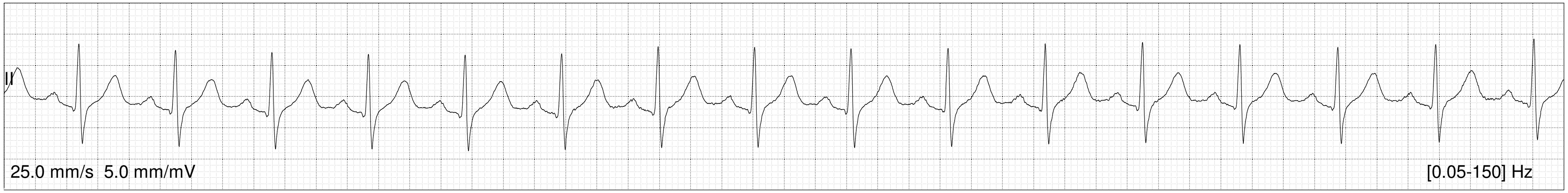

Anton Swart Biokinetic Rehabilitation Practice

Name:	012 012 012	Recorded:	05/05/2018 12:11:40	UNCONFIRMED INTERPRETATION - MD SHOULD REVIEW
Number:	012	Recorded by:	Mr. Anton Swart	
Gender:	Male	Referring physician:		
Birthdate:	28/12/1963 54 years	Location:	Anton Swart Biokinetic Rehabilitation Practice	
		Ordering physician:		
		Attending physician:		
		Comment:		

P / PQ:	113 ms / 175 ms
QRS:	118 ms
QT / QTc / QTd:	375 ms / 436 ms / -
P/QRS/T axis:	85° / 77° / 85°
Heartrate:	95 bpm

Beats

Total:	477
Normal:	477
Other:	0

RR

Minimum	600 ms
Maximum	647 ms
Mean:	626 ms
SD:	9 ms

R-R Trend

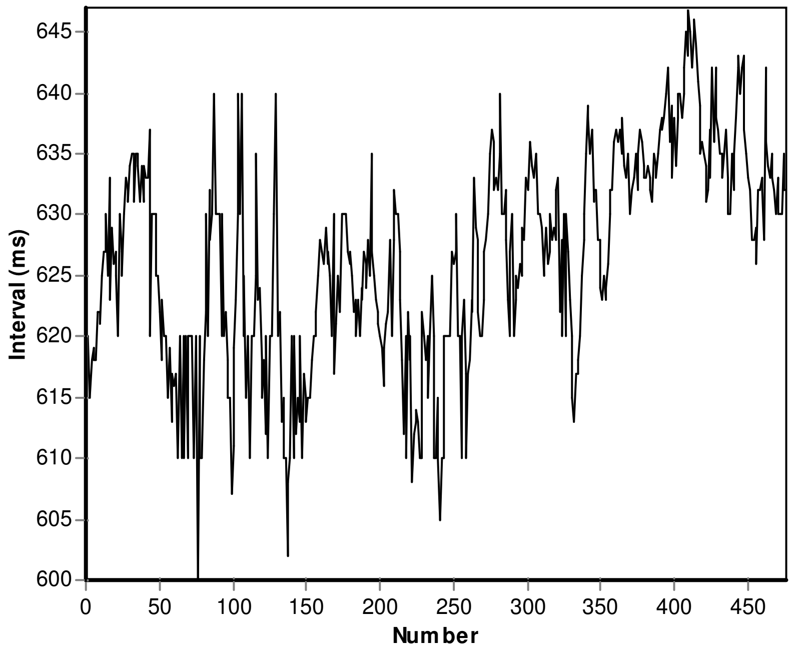

R-R Histogram

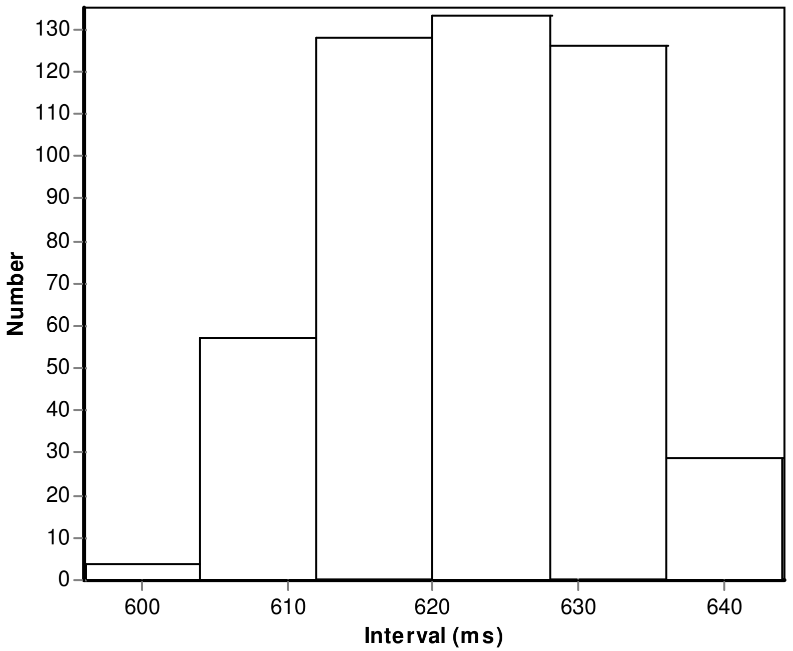

Cardio Control NV. User: Anton

Heart Rate Variability: Time Domain Analysis

**Name:**	012, 012 012	**Birthdate:**	28/12/1963
**Number:**	012	**Recorded:**	05/05/2018 12:11:40
**Gender:**	Male		

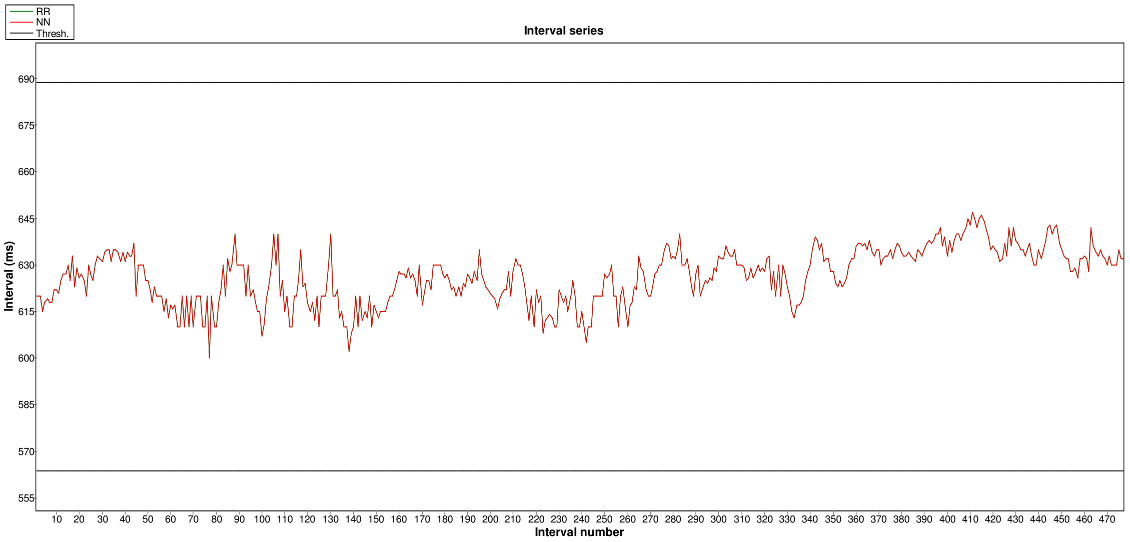

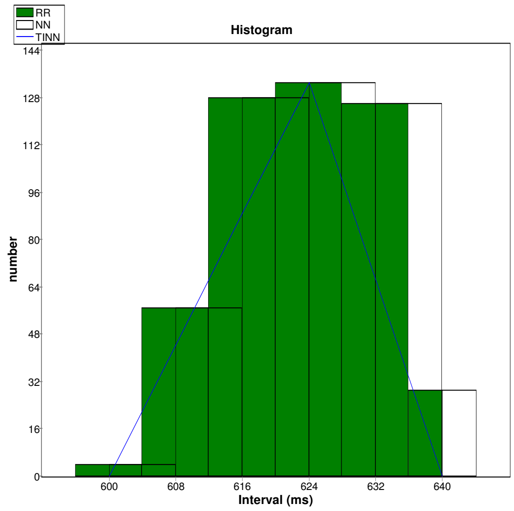

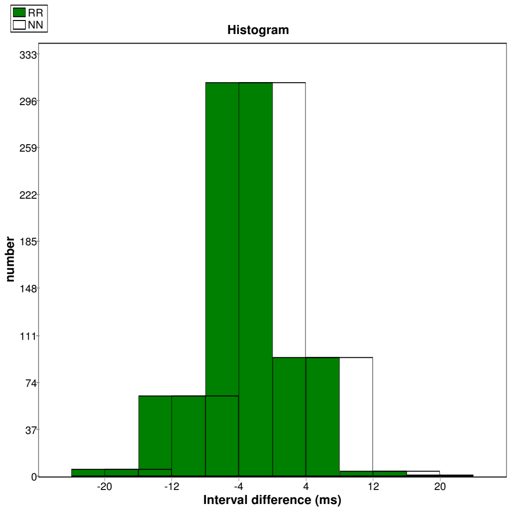

Binsize (ms) = 8

**HRV parameters**	**NN**	**RR**
SDNN (ms)	9	9
Triangular Interpolation (ms)	40	40
Triangular Index	3.59	3.59

**HRV parameters**	**NN**	**RR**
SDSD (ms)	5	5
RMSSD (ms)	5	5
NN50	0	0
NN50(1)	0	0
NN50(2)	0	0
pNN50	0.00	0.00
pNN50(1)	0.00	0.00
pNN50(2)	0.00	0.00
Logarithmic Index	2.19	2.19
SD(Logarithmic Index)	0.28	0.28

**Interval statistics**	**NN**	**RR**
Number	477	477
Minimum (ms)	600	600
Maximum (ms)	647	647
Range (ms)	47	47
Avg (ms)	626	626
SD (ms)	9	9
AvgDev (ms)	7	7
p5 (ms)	610	610
p50 (ms)	627	627
p95 (ms)	640	640
Skewness	-0.21	-0.21
Kurtosis	2.50	2.50

**Interval statistics**	**NN**	**RR**
Number	476	476
Minimum (ms)	-20	-20
Maximum (ms)	20	20
Range (ms)	40	40
Avg (ms)	0	0
SD (ms)	5	5
AvgDev (ms)	4	4
p5 (ms)	-10	-10
p50 (ms)	0	0
p95 (ms)	10	10
Skewness	-0.26	-0.26
Kurtosis	4.63	4.63

Cardio Control NV. User: Anton

Heart Rate Variability: Frequency Domain Analysis

**Name:**	012, 012 012	**Birthdate:**	28/12/1963
**Number:**	012	**Recorded:**	05/05/2018 12:11:40
**Gender:**	Male		

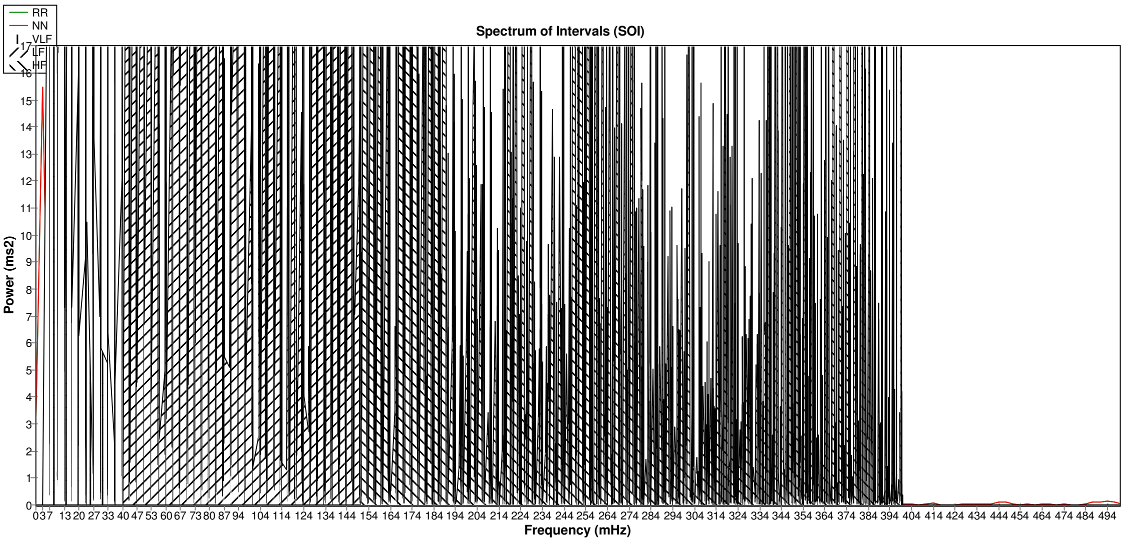

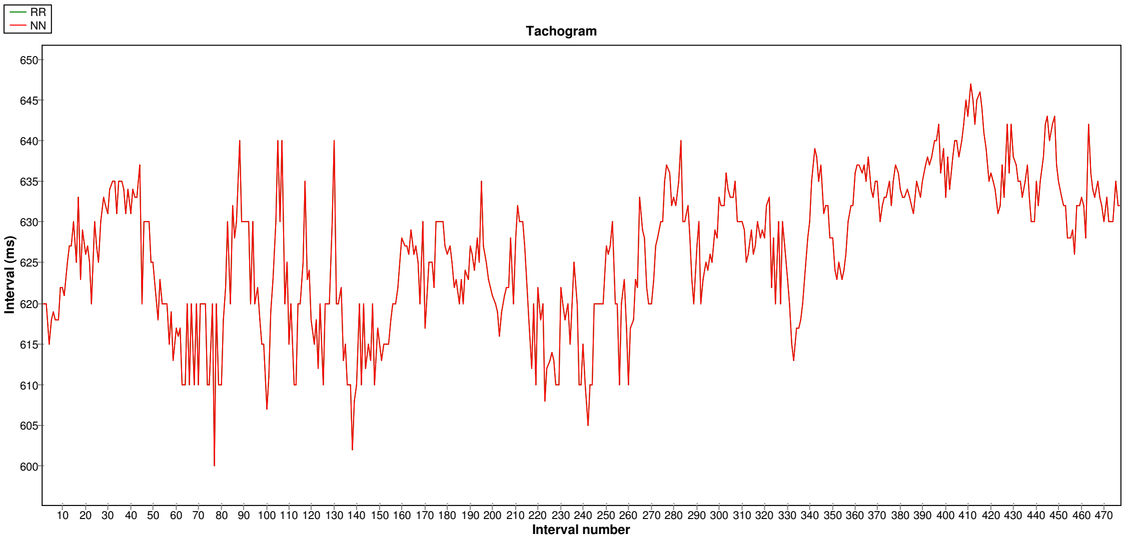

**HRV parameters**	**NN**	**RR**
TP (ms2)	41	41
VLF (ms2)	19	19
LF (ms2)	17	17
HF (ms2)	5	5
LF/HF	3.48	3.48
LF normalized	77.66	77.66
HF normalized	22.34	22.34
VLF peak (mHz)	17	17
LF peak (mHz)	87	87
HF peak (mHz)	150	150

**HRV spectral settings**	
Spectrum of Intervals (SOI)	
Frequency resolution (mHz)	3
VLF lower boundary (mHz)	3
VLF upper boundary (mHz)	40
LF upper boundary (mHz)	150
HF upper boundary (mHz)	400
Smoothing factor	1
Tapering	Hann
Fourier transform	DFT
Sample frequency (Hz)	1.60
Interval correction	Annotation
Interval threshold (%)	10
