# Supplementary material for: The effects of a 3-day mountain bike cycling race on the autonomic nervous system (ANS) and heart rate variability in amateur cyclists: a prospective quantitative research design
Source: BMC Sports Sci Med Rehabil. 2023 Jan 2;15:2. doi: 10.1186/s13102-022-00614-y (PMC9808932; doi:10.1186/s13102-022-00614-y)
Supplement: Supplementary file 1 — Additional file 1. Individual data of Participants. [file 13102_2022_614_MOESM1_ESM.zip › Individual data of Participants/HRV Data/011/ECG_011_20180506081057_.PDF]

# Anton Swart Biokinetic Rehabilitation Practice

UNCONFIRMED INTERPRETATION - MD SHOULD REVIEW

| | | | |
|---|---|---|---|
| Name: | 012 012 012 | Recorded: | 06/05/2018 08:10:57 |
| Number: | 012 | Recorded by: | Mr. Anton Swart |
| Gender: | Male | Referring physician: | |
| Birthdate: | 28/12/1963 54 years | Ordering physician: | |
| | | Attending physician: | |
| P / PQ: | 115 ms / 185 ms | Location: | Anton Swart Biokinetic Rehabilitation Practi |
| QRS: | 122 ms | Comment: | |
| QT / QTc / QTd: | 448 ms / 450 ms / - | | |
| P/QRS/T axis: | 80° / 79° / 85° | | |
| Heartrate: | 61 bpm | | |

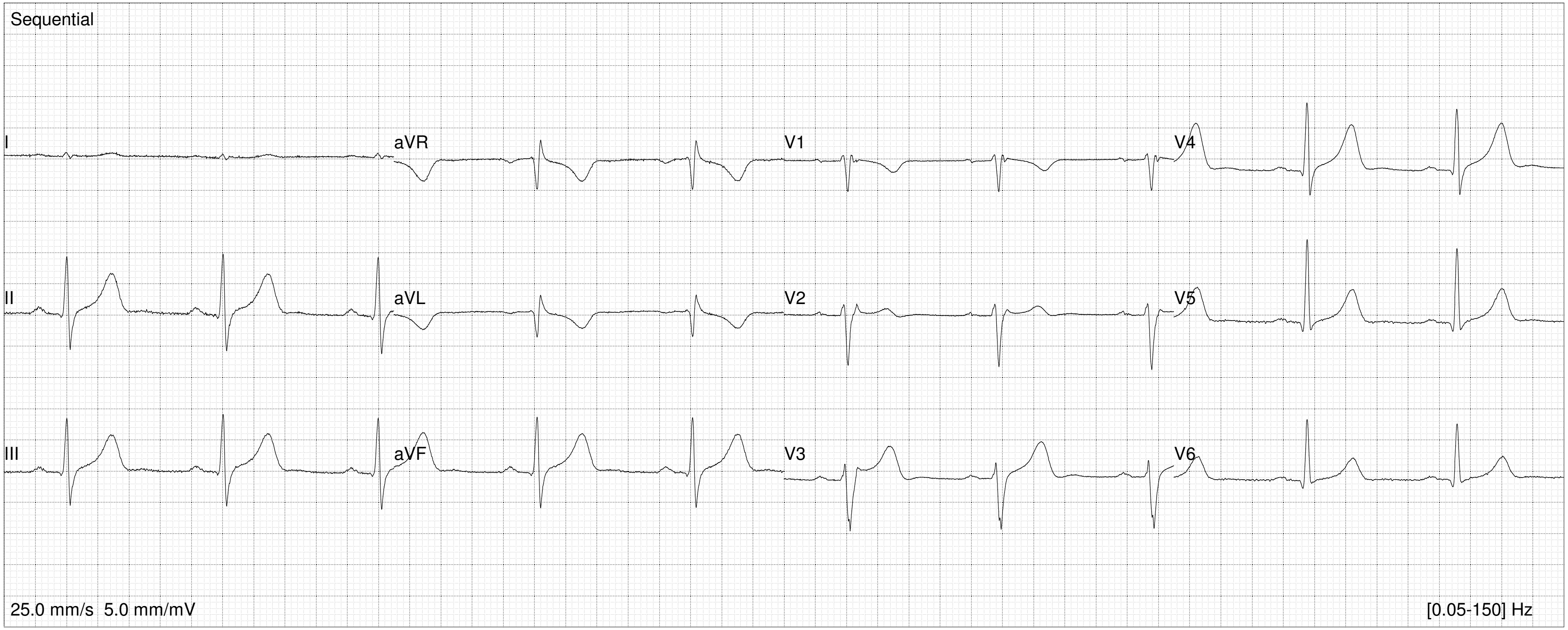


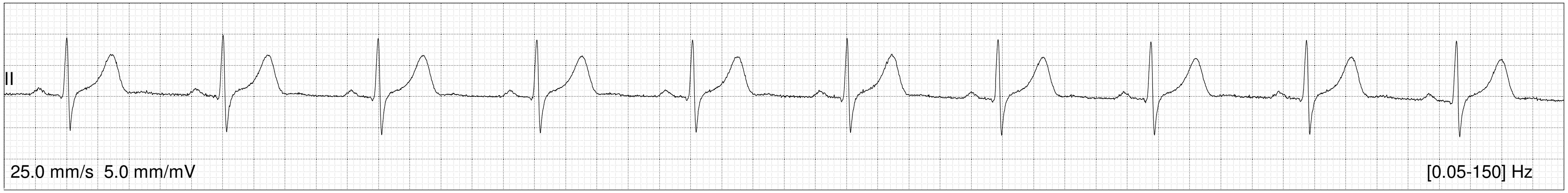

# Anton Swart Biokinetic Rehabilitation Practice

| | | | | |
|---|---|---|---|---|
| Name: | 012 012 012 | Recorded: | 06/05/2018 08:10:57 | UNCONFIRMED INTERPRETATION - MD SHOULD REVIEW |
| Number: | 012 | Recorded by: | Mr. Anton Swart | |
| Gender: | Male | Referring physician: | | |
| Birthdate: | 28/12/1963 54 years | Location: | Anton Swart Biokinetic Rehabilitation Practice | |
| P / PQ: | 115 ms / 185 ms | Ordering physician: | | |
| QRS: | 122 ms | Attending physician: | | |
| QT / QTc / QTd: | 448 ms / 450 ms / - | Comment: | | |
| P/QRS/T axis: | 80° / 79° / 85° | | | |
| Heartrate: | 61 bpm | | | |

| **Beats** | | **RR** | |
|---|---|---|---|
| Total: | 308 | Minimum | 530 ms |
| Normal: | 308 | Maximum | 1430 ms |
| Other: | 0 | Mean: | 969 ms |
| | | SD: | 53 ms |

## R-R Trend

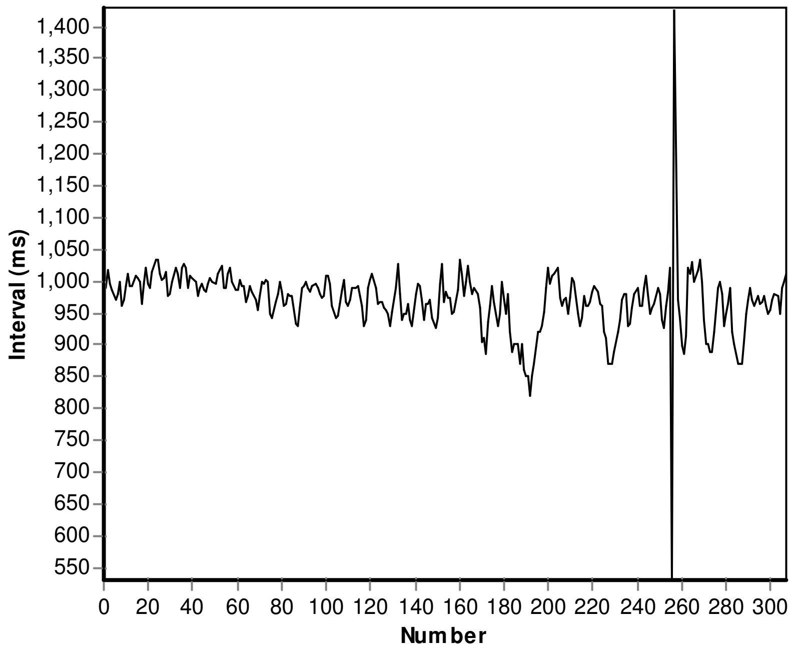


## R-R Histogram

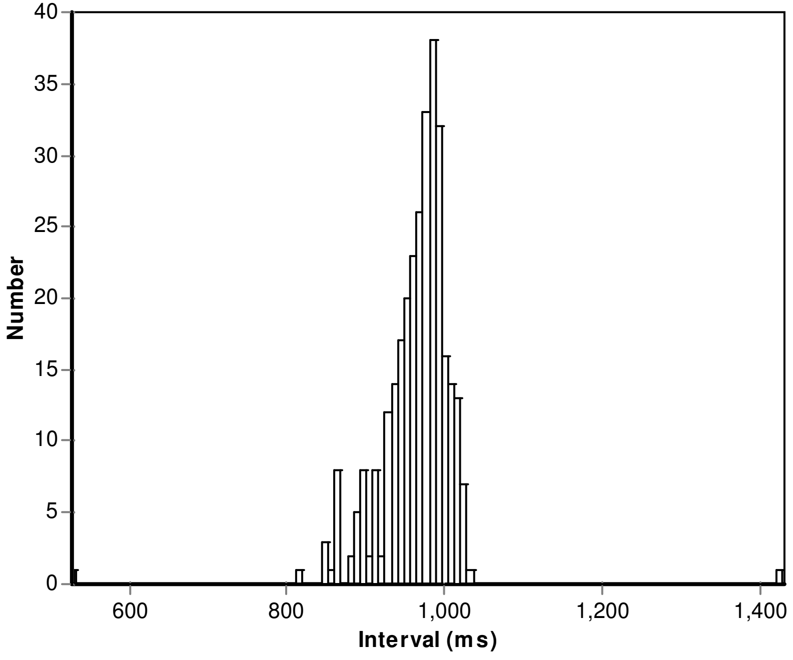

Cardio Control NV. User: Anton

## Heart Rate Variability: Time Domain Analysis

| | | | |
|---|---|---|---|
| **Name:** | 012, 012 012 | **Birthdate:** | 28/12/1963 |
| **Number:** | 012 | **Recorded:** | 06/05/2018 08:10:57 |
| **Gender:** | Male | | |

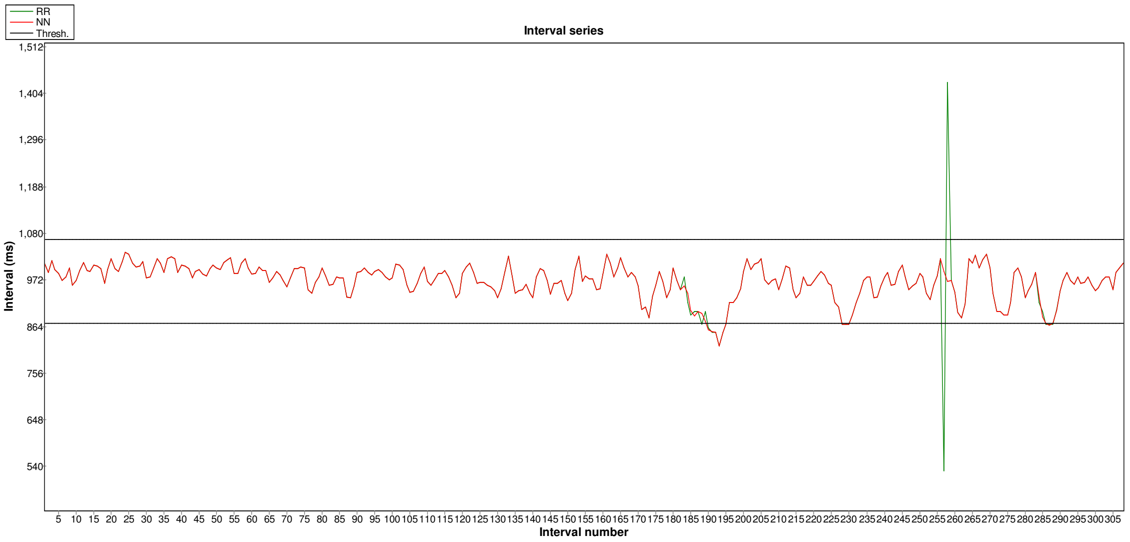


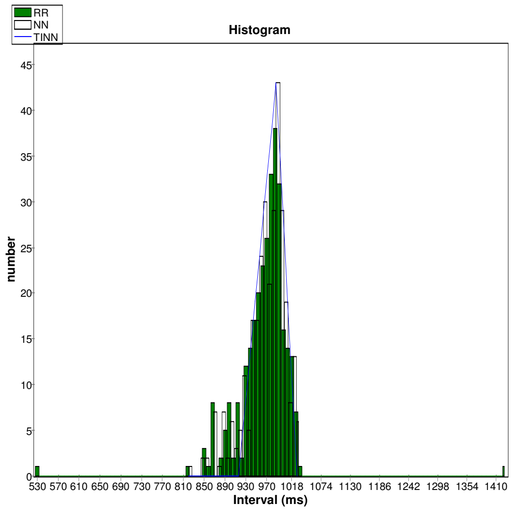


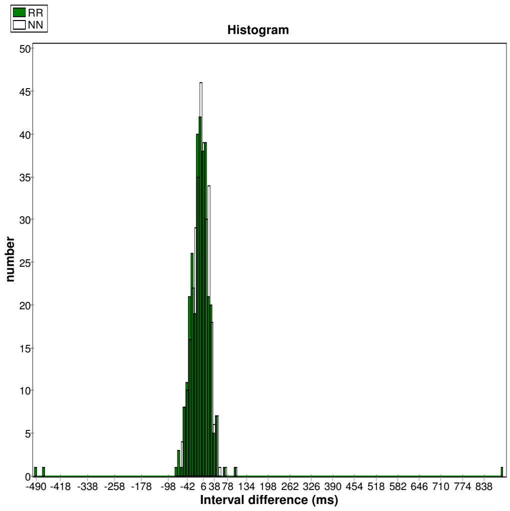


Binsize (ms) = 8

| **HRV parameters** | **NN** | **RR** |
|---|---|---|
| SDNN (ms) | 39 | 53 |
| Triangular Interpolation (ms) | 112 | 128 |
| Triangular Index | 7.16 | 8.11 |

| **HRV parameters** | **NN** | **RR** |
|---|---|---|
| SDSD (ms) | 24 | 69 |
| RMSSD (ms) | 24 | 69 |
| NN50 | 7 | 11 |
| NN50(1) | 4 | 7 |
| NN50(2) | 3 | 4 |
| pNN50 | 0.02 | 0.04 |
| pNN50(1) | 0.01 | 0.02 |
| pNN50(2) | 0.01 | 0.01 |
| Logarithmic Index | 0.54 | 0.45 |
| SD(Logarithmic Index) | 0.05 | 0.03 |

| **Interval statistics** | **NN** | **RR** |
|---|---|---|
| Number | 308 | 308 |
| Minimum (ms) | 820 | 530 |
| Maximum (ms) | 1035 | 1430 |
| Range (ms) | 215 | 900 |
| Avg (ms) | 969 | 969 |
| SD (ms) | 39 | 53 |
| AvgDev (ms) | 30 | 33 |
| p5 (ms) | 886 | 885 |
| p50 (ms) | 978 | 978 |
| p95 (ms) | 1020 | 1021 |
| Skewness | -1.07 | -0.13 |
| Kurtosis | 4.13 | 34.67 |

| **Interval statistics** | **NN** | **RR** |
|---|---|---|
| Number | 307 | 307 |
| Minimum (ms) | -60 | -490 |
| Maximum (ms) | 105 | 900 |
| Range (ms) | 165 | 1390 |
| Avg (ms) | 0 | 0 |
| SD (ms) | 24 | 69 |
| AvgDev (ms) | 19 | 25 |
| p5 (ms) | -40 | -40 |
| p50 (ms) | 0 | 0 |
| p95 (ms) | 36 | 38 |
| Skewness | 0.12 | 5.20 |
| Kurtosis | 3.70 | 111.85 |

Cardio Control NV. User: Anton

## Heart Rate Variability: Frequency Domain Analysis

| | | | |
|---|---|---|---|
| **Name:** | 012, 012 012 | **Birthdate:** | 28/12/1963 |
| **Number:** | 012 | **Recorded:** | 06/05/2018 08:10:57 |
| **Gender:** | Male | | |

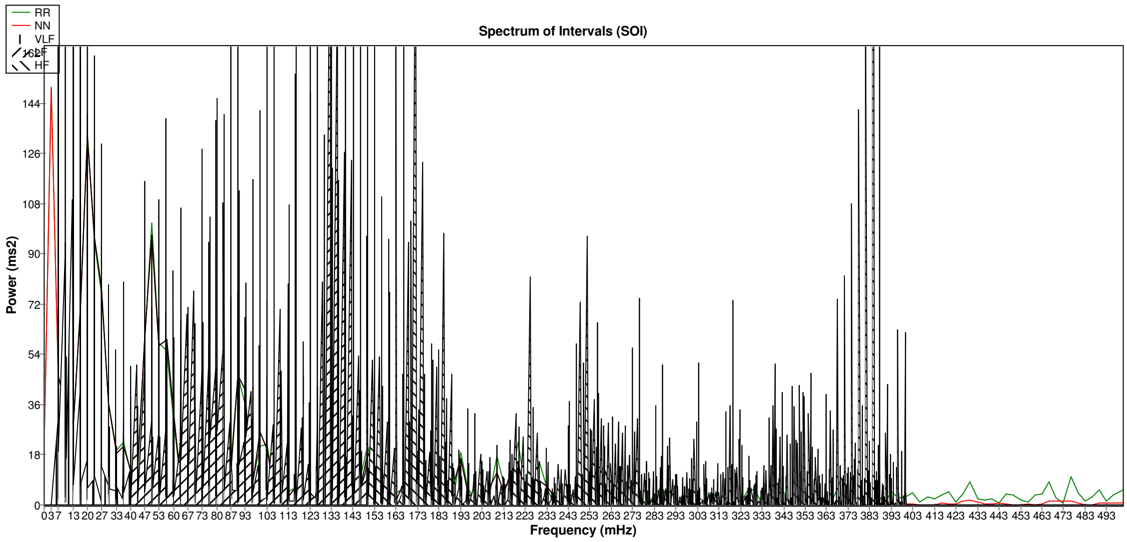


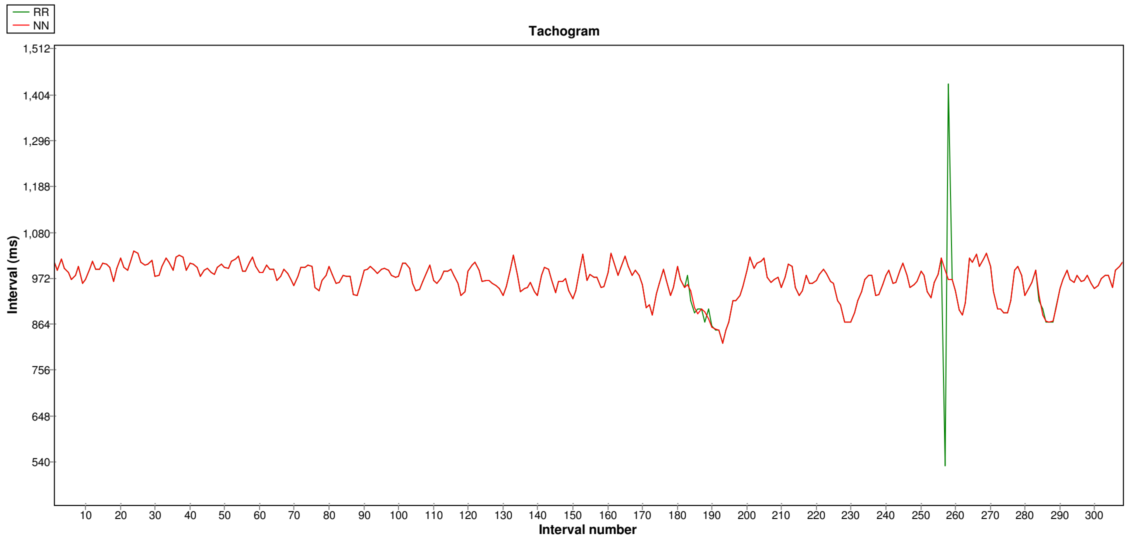


| **HRV parameters** | **NN** | **RR** |
|---|---|---|
| TP (ms2) | 1400 | 1526 |
| VLF (ms2) | 575 | 575 |
| LF (ms2) | 576 | 581 |
| HF (ms2) | 249 | 369 |
| LF/HF | 2.31 | 1.57 |
| LF normalized | 69.81 | 61.13 |
| HF normalized | 30.19 | 38.87 |
| VLF peak (mHz) | 20 | 20 |
| LF peak (mHz) | 50 | 50 |
| HF peak (mHz) | 193 | 220 |

| **HRV spectral settings** | |
|---|---|
| Spectrum of Intervals (SOI) | |
| Frequency resolution (mHz) | 3 |
| VLF lower boundary (mHz) | 3 |
| VLF upper boundary (mHz) | 40 |
| LF upper boundary (mHz) | 150 |
| HF upper boundary (mHz) | 400 |
| Smoothing factor | 1 |
| Tapering | Hann |
| Fourier transform | DFT |
| Sample frequency (Hz) | 1.03 |
| Interval correction | Annotation |
| Interval threshold (%) | 10 |
