# Supplementary material for: The effects of a 3-day mountain bike cycling race on the autonomic nervous system (ANS) and heart rate variability in amateur cyclists: a prospective quantitative research design
Source: BMC Sports Sci Med Rehabil. 2023 Jan 2;15:2. doi: 10.1186/s13102-022-00614-y (PMC9808932; doi:10.1186/s13102-022-00614-y)
Supplement: Supplementary file 1 — Additional file 1. Individual data of Participants. [file 13102_2022_614_MOESM1_ESM.zip › Individual data of Participants/HRV Data/012/ECG_012_20180501161753_.PDF]

Anton Swart Biokinetic Rehabilitation Practice

Name:	013 013	Recorded:	01/05/2018 16:17:53	UNCONFIRMED INTERPRETATION - MD SHOULD REVIEW
Number:	013	Recorded by:	Mr. Anton Swart	
Gender:	Male	Referring physician:		
Birthdate:	04/02/1971 47 years	Ordering physician:		
		Attending physician:		
		Location:	Anton Swart Biokinetic Rehabilitation Practi	
		Comment:		

P / PQ:	127 ms / 192 ms
QRS:	98 ms
QT / QTc / QTd:	391 ms / 412 ms / -
P/QRS/T axis:	65° / 95° / 62°
Heartrate:	72 bpm

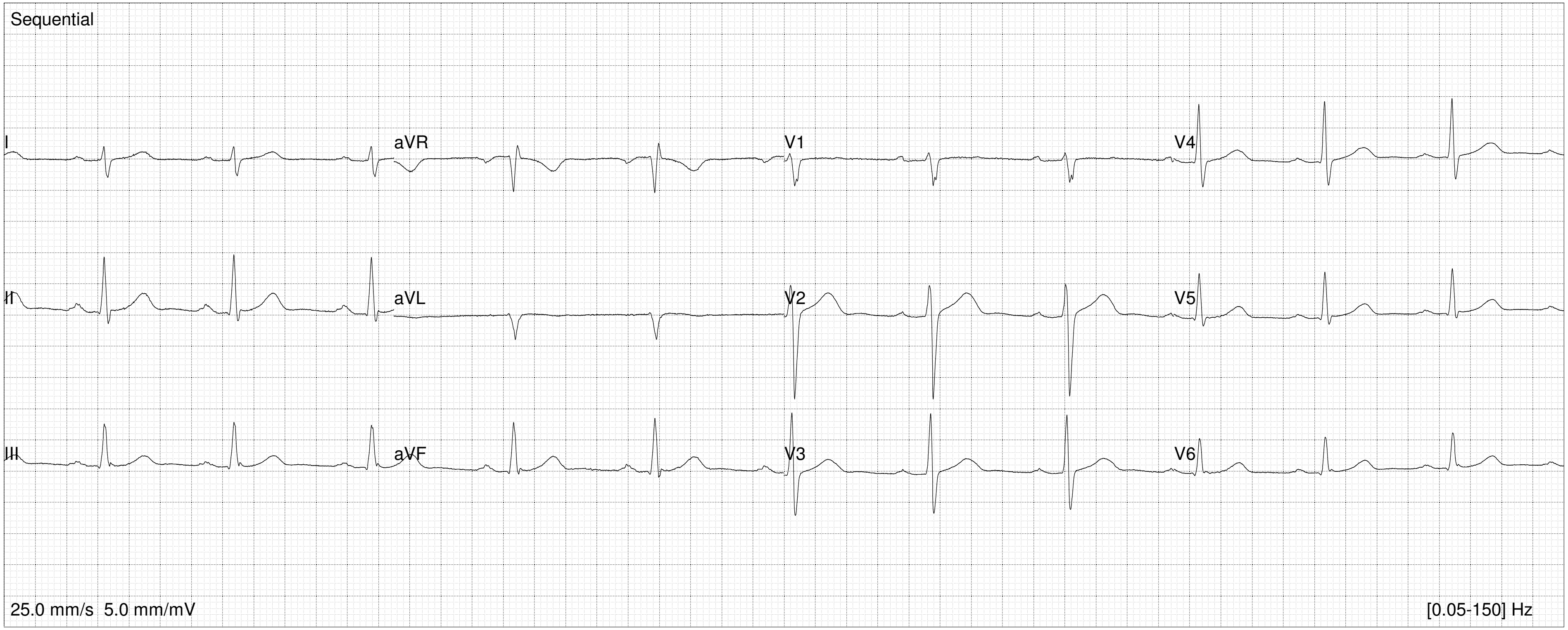

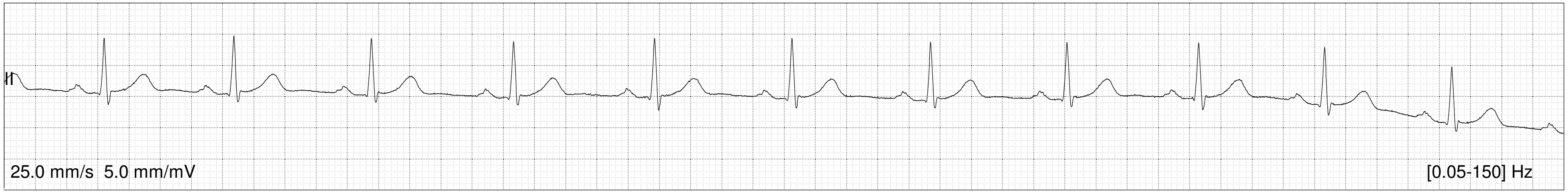

Anton Swart Biokinetic Rehabilitation Practice

Name:	013 013	Recorded:	01/05/2018 16:17:53	UNCONFIRMED INTERPRETATION - MD SHOULD REVIEW
Number:	013	Recorded by:	Mr. Anton Swart	
Gender:	Male	Referring physician:		
Birthdate:	04/02/1971 47 years	Location:	Anton Swart Biokinetic Rehabilitation Practice	
P / PQ:	127 ms / 192 ms	Ordering physician:		
QRS:	98 ms	Attending physician:		
QT / QTc / QTd:	391 ms / 412 ms / -	Comment:		
P/QRS/T axis:	65° / 95° / 62°			
Heartrate:	72 bpm			

Beats

Total:	357
Normal:	357
Other:	0

RR

Minimum	720 ms
Maximum	932 ms
Mean:	837 ms
SD:	31 ms

R-R Trend

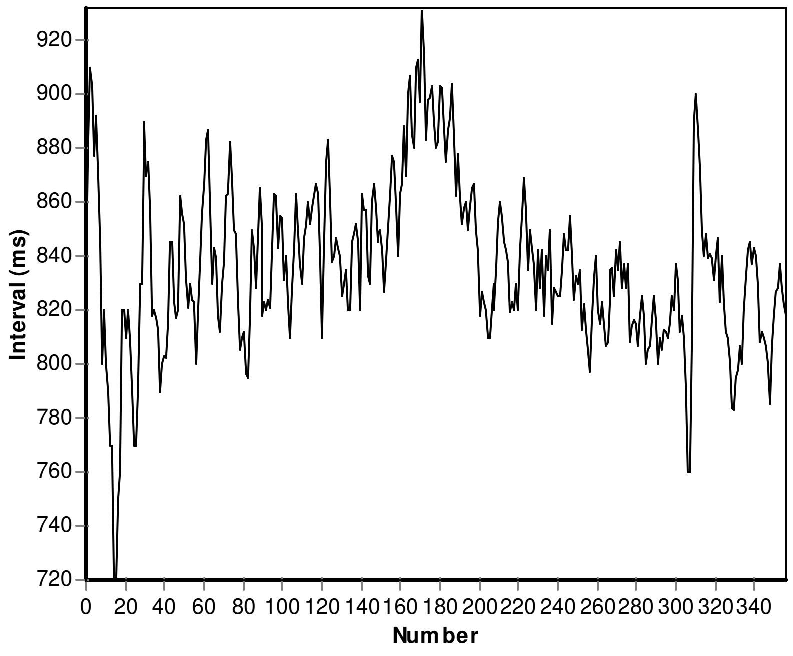

R-R Histogram

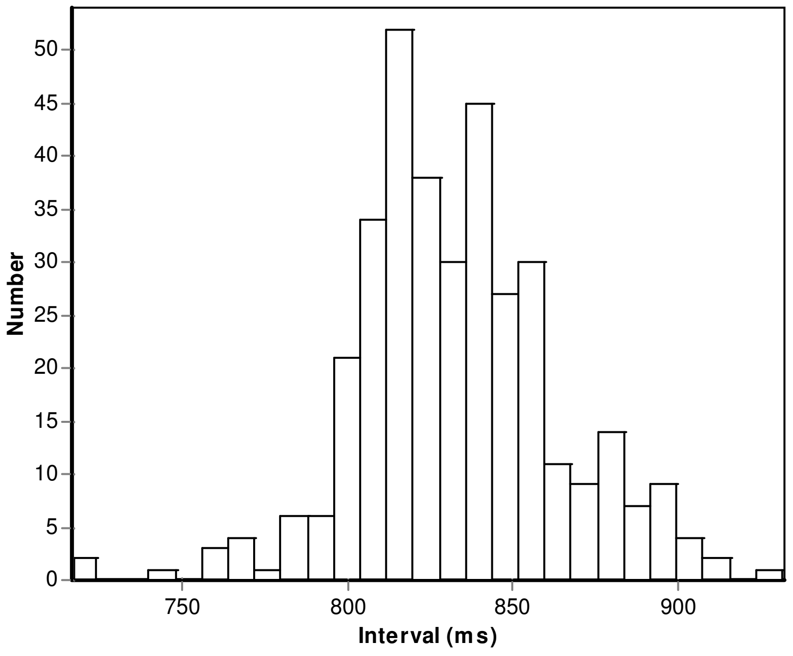

Cardio Control NV. User: Anton

Heart Rate Variability: Time Domain Analysis

Name: 013, 013 **Birthdate:** 04/02/1971
Number: 013 **Recorded:** 01/05/2018 16:17:53
Gender: Male

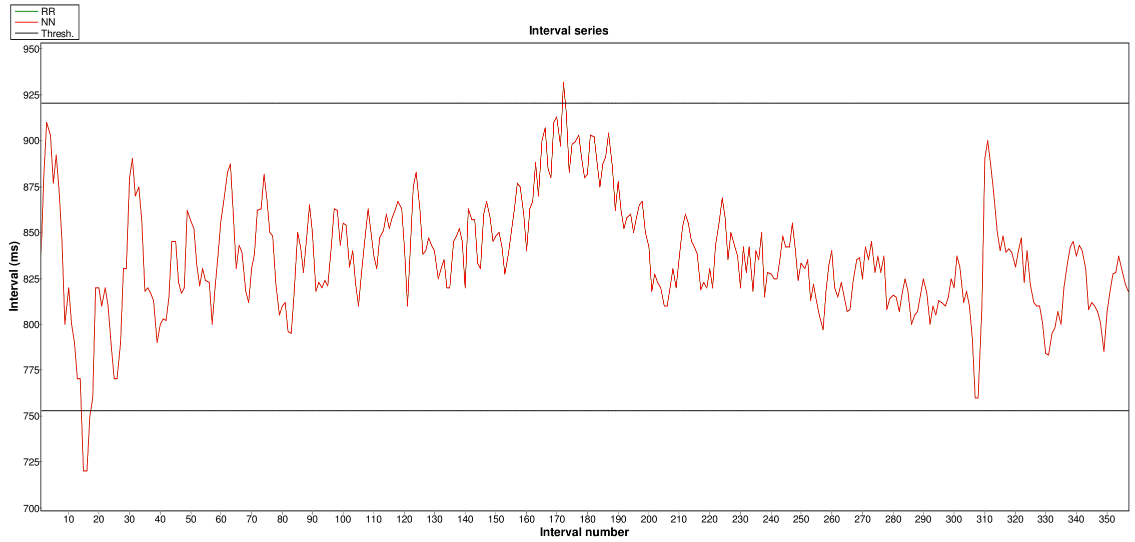

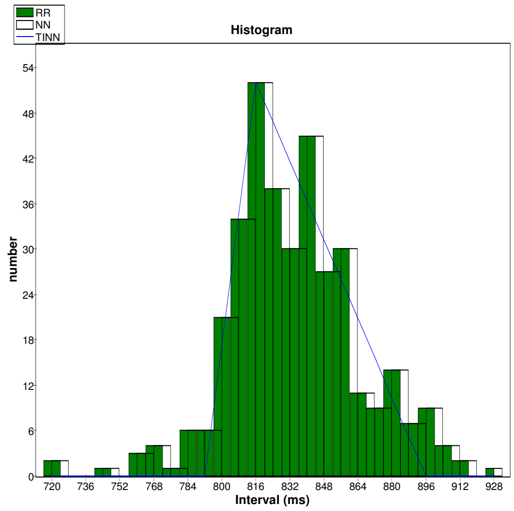

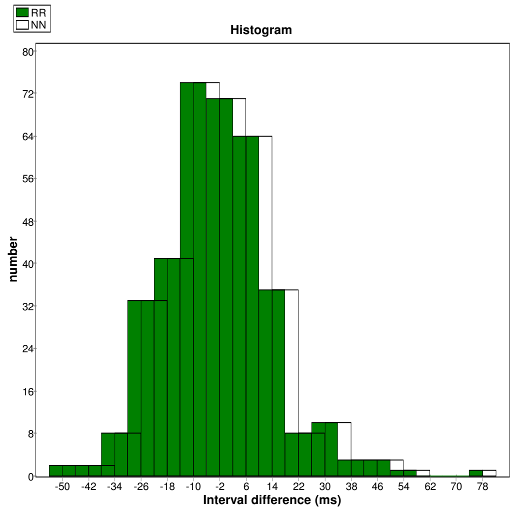

Binsize (ms) = 8

HRV parameters	NN	RR
SDNN (ms)	31	31
Triangular Interpolation (ms)	104	104
Triangular Index	6.87	6.87

HRV parameters	NN	RR
SDSD (ms)	17	17
RMSSD (ms)	17	17
NN50	2	2
NN50(1)	0	0
NN50(2)	2	2
pNN50	0.01	0.01
pNN50(1)	0.00	0.00
pNN50(2)	0.01	0.01
Logarithmic Index	0.69	0.69
SD(Logarithmic Index)	0.05	0.05

Interval statistics	NN	RR
Number	357	357
Minimum (ms)	720	720
Maximum (ms)	932	932
Range (ms)	212	212
Avg (ms)	837	837
SD (ms)	31	31
AvgDev (ms)	24	24
p5 (ms)	792	792
p50 (ms)	835	835
p95 (ms)	892	892
Skewness	0.02	0.02
Kurtosis	4.04	4.04

Interval statistics	NN	RR
Number	356	356
Minimum (ms)	-50	-50
Maximum (ms)	80	80
Range (ms)	130	130
Avg (ms)	-0	-0
SD (ms)	17	17
AvgDev (ms)	13	13
p5 (ms)	-24	-24
p50 (ms)	0	0
p95 (ms)	30	30
Skewness	0.56	0.56
Kurtosis	4.77	4.77

Cardio Control NV. User: Anton

Heart Rate Variability: Frequency Domain Analysis

**Name:**	013, 013	**Birthdate:**	04/02/1971
**Number:**	013	**Recorded:**	01/05/2018 16:17:53
**Gender:**	Male		

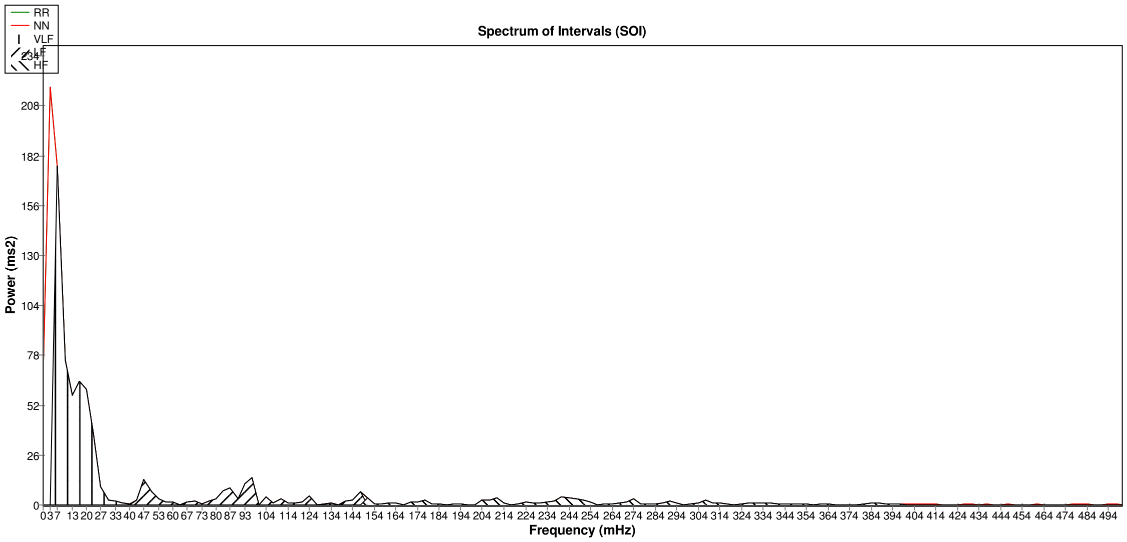

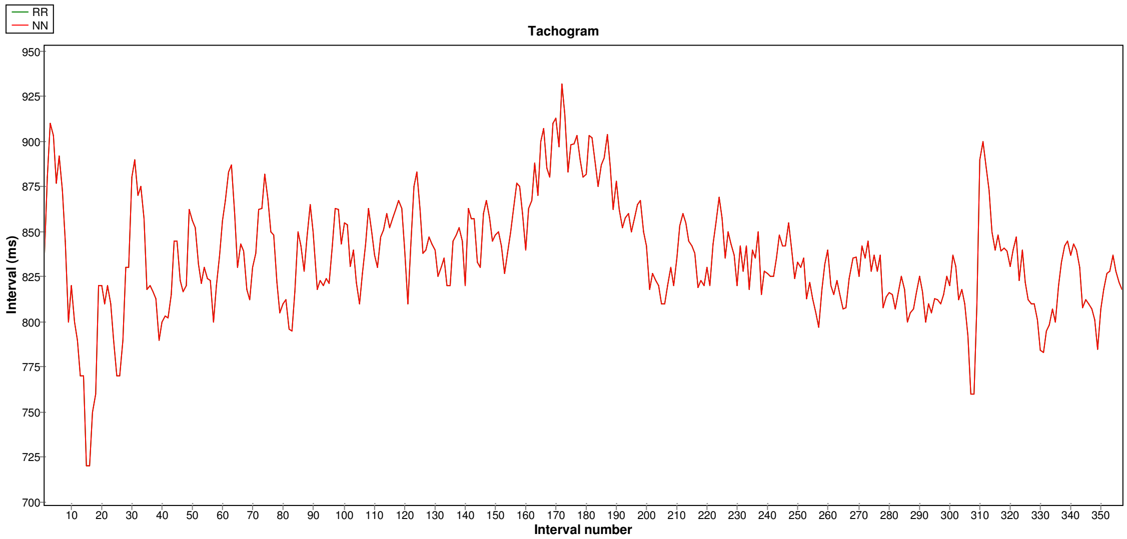

**HRV parameters**	**NN**	**RR**
TP (ms2)	683	683
VLF (ms2)	487	487
LF (ms2)	115	115
HF (ms2)	82	82
LF/HF	1.40	1.40
LF normalized	58.35	58.35
HF normalized	41.65	41.65
VLF peak (mHz)	7	7
LF peak (mHz)	97	97
HF peak (mHz)	240	240

**HRV spectral settings**	
Spectrum of Intervals (SOI)	
Frequency resolution (mHz)	3
VLF lower boundary (mHz)	3
VLF upper boundary (mHz)	40
LF upper boundary (mHz)	150
HF upper boundary (mHz)	400
Smoothing factor	1
Tapering	Hann
Fourier transform	DFT
Sample frequency (Hz)	1.20
Interval correction	Annotation
Interval threshold (%)	10
