# Supplementary material for: The effects of a 3-day mountain bike cycling race on the autonomic nervous system (ANS) and heart rate variability in amateur cyclists: a prospective quantitative research design
Source: BMC Sports Sci Med Rehabil. 2023 Jan 2;15:2. doi: 10.1186/s13102-022-00614-y (PMC9808932; doi:10.1186/s13102-022-00614-y)
Supplement: Supplementary file 1 — Additional file 1. Individual data of Participants. [file 13102_2022_614_MOESM1_ESM.zip › Individual data of Participants/HRV Data/012/ECG_012_20180503173001_.PDF]

Anton Swart Biokinetic Rehabilitation Practice

Name: 013 013
Number: 013
Gender: Male
Birthdate: 04/02/1971 47 years

Recorded: 03/05/2018 17:30:01
Recorded by: Mr. Anton Swart
Referring physician:
Ordering physician:
Attending physician:
Location: Anton Swart Biokinetic Rehabilitation Practi
Comment:

UNCONFIRMED INTERPRETATION - MD SHOULD REVIEW

P / PQ: 127 ms / 197 ms
QRS: 95 ms
QT / QTc / QTd: 370 ms / 410 ms / -
P/QRS/T axis: 67° / 93° / 62°
Heartrate: 83 bpm

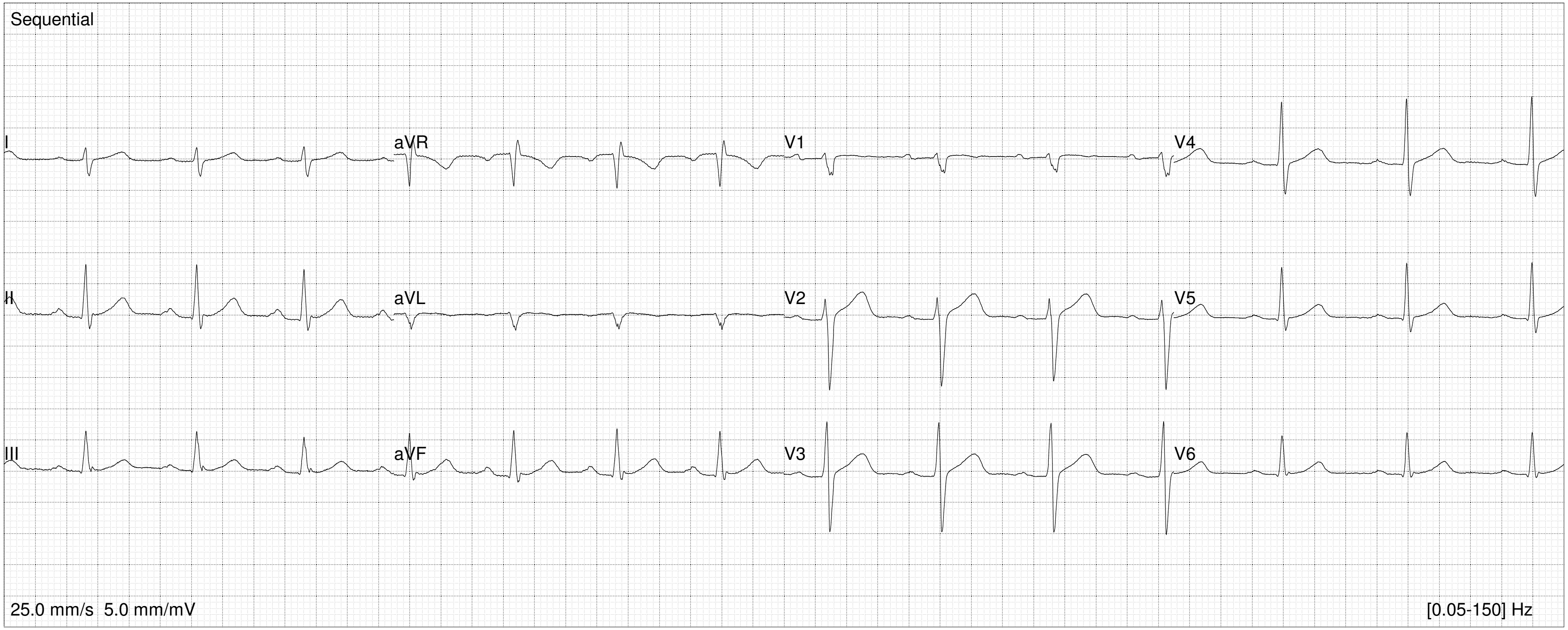

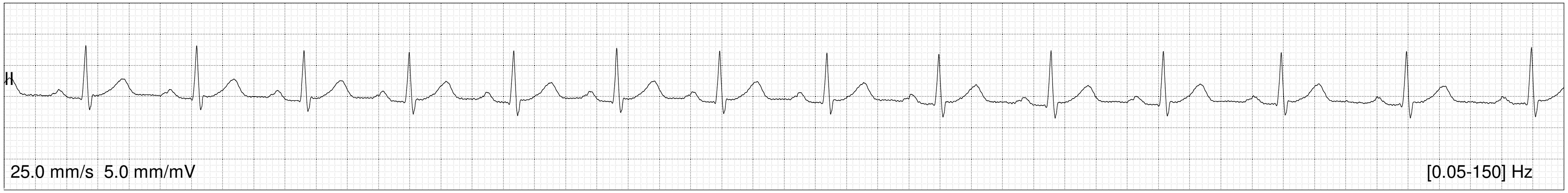

Anton Swart Biokinetic Rehabilitation Practice

Name:	013 013	Recorded:	03/05/2018 17:30:01	UNCONFIRMED INTERPRETATION - MD SHOULD REVIEW
Number:	013	Recorded by:	Mr. Anton Swart	
Gender:	Male	Referring physician:		
Birthdate:	04/02/1971 47 years	Location:	Anton Swart Biokinetic Rehabilitation Practice	
		Ordering physician:		
		Attending physician:		
		Comment:		

P / PQ:	127 ms / 197 ms
QRS:	95 ms
QT / QTc / QTd:	370 ms / 410 ms / -
P/QRS/T axis:	67° / 93° / 62°
Heartrate:	83 bpm

Beats

Total:	410
Normal:	410
Other:	0

RR

Minimum	650 ms
Maximum	860 ms
Mean:	728 ms
SD:	30 ms

R-R Trend

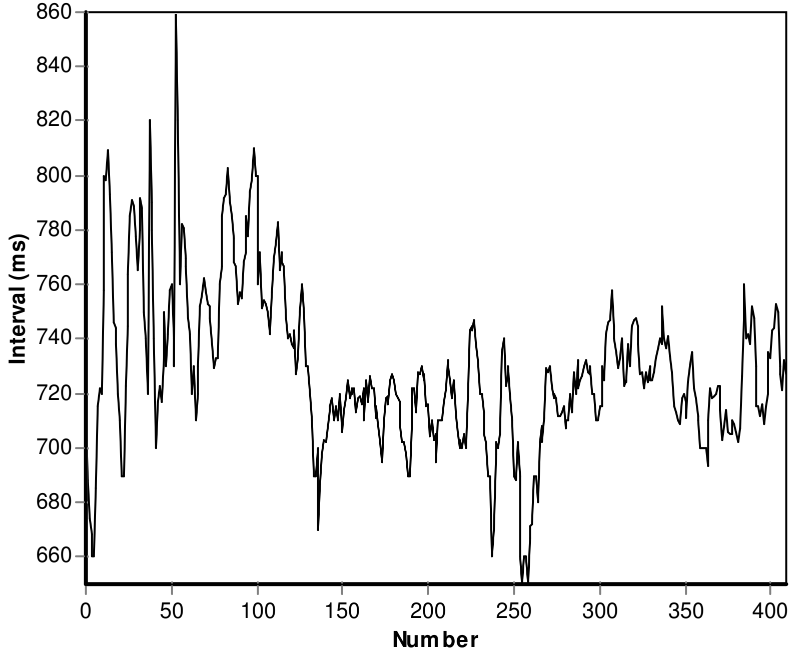

R-R Histogram

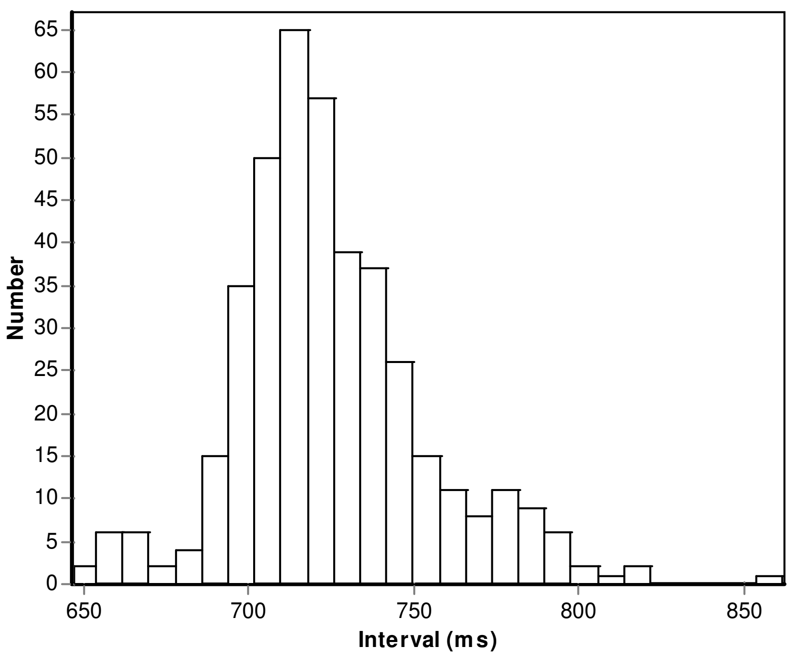

Heart Rate Variability: Time Domain Analysis

**Name:**	013, 013	**Birthdate:**	04/02/1971
**Number:**	013	**Recorded:**	03/05/2018 17:30:01
**Gender:**	Male		

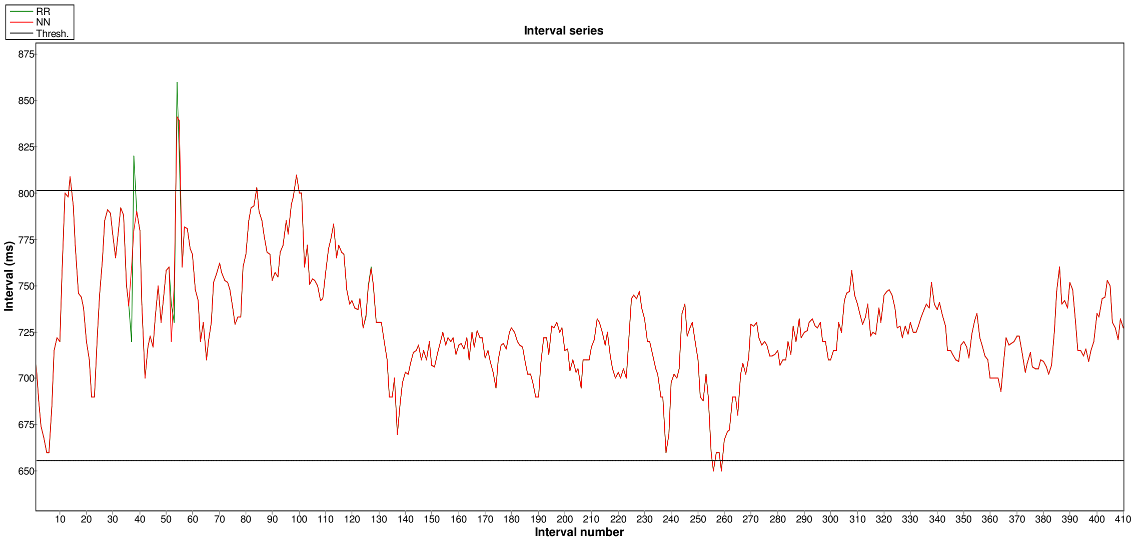

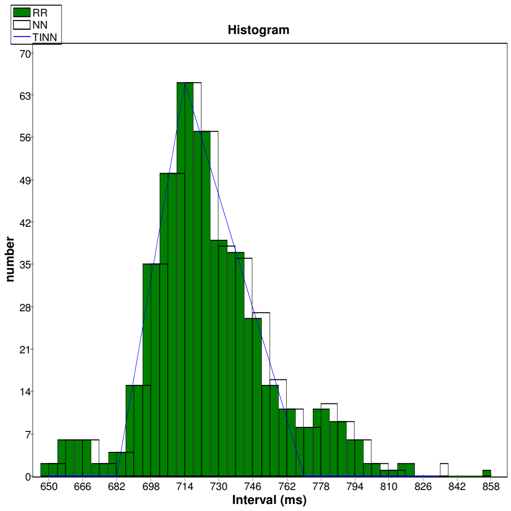

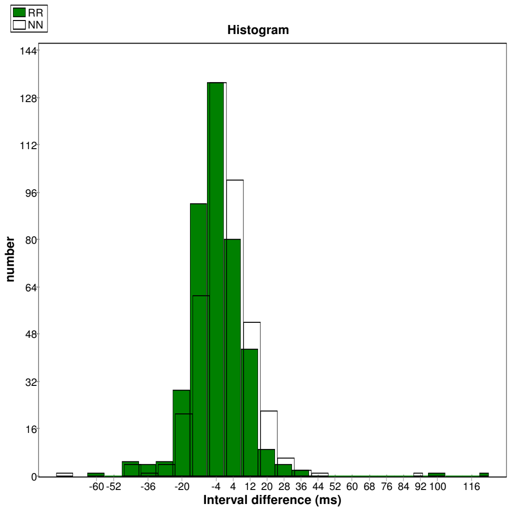

Binsize (ms) = 8

**HRV parameters**	**NN**	**RR**
SDNN (ms)	29	30
Triangular Interpolation (ms)	88	88
Triangular Index	6.31	6.31

**HRV parameters**	**NN**	**RR**
SDSD (ms)	13	14
RMSSD (ms)	13	14
NN50	2	3
NN50(1)	1	1
NN50(2)	1	2
pNN50	0.00	0.01
pNN50(1)	0.00	0.00
pNN50(2)	0.00	0.00
Logarithmic Index	0.83	0.74
SD(Logarithmic Index)	0.08	0.08

**Interval statistics**	**NN**	**RR**
Number	410	410
Minimum (ms)	650	650
Maximum (ms)	841	860
Range (ms)	191	210
Avg (ms)	728	728
SD (ms)	29	30
AvgDev (ms)	22	22
p5 (ms)	689	689
p50 (ms)	724	724
p95 (ms)	787	789
Skewness	0.57	0.64
Kurtosis	4.10	4.37

**Interval statistics**	**NN**	**RR**
Number	409	409
Minimum (ms)	-79	-60
Maximum (ms)	91	130
Range (ms)	170	190
Avg (ms)	0	0
SD (ms)	13	14
AvgDev (ms)	9	9
p5 (ms)	-20	-20
p50 (ms)	0	0
p95 (ms)	19	19
Skewness	0.21	2.28
Kurtosis	11.93	24.08

Cardio Control NV. User: Anton

Heart Rate Variability: Frequency Domain Analysis

**Name:**	013, 013	**Birthdate:**	04/02/1971
**Number:**	013	**Recorded:**	03/05/2018 17:30:01
**Gender:**	Male		

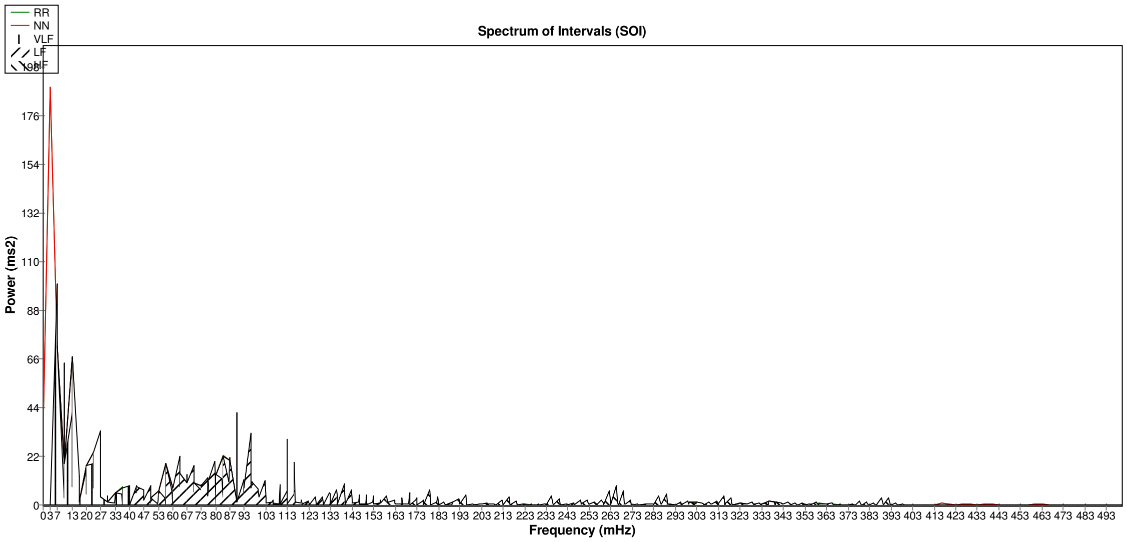

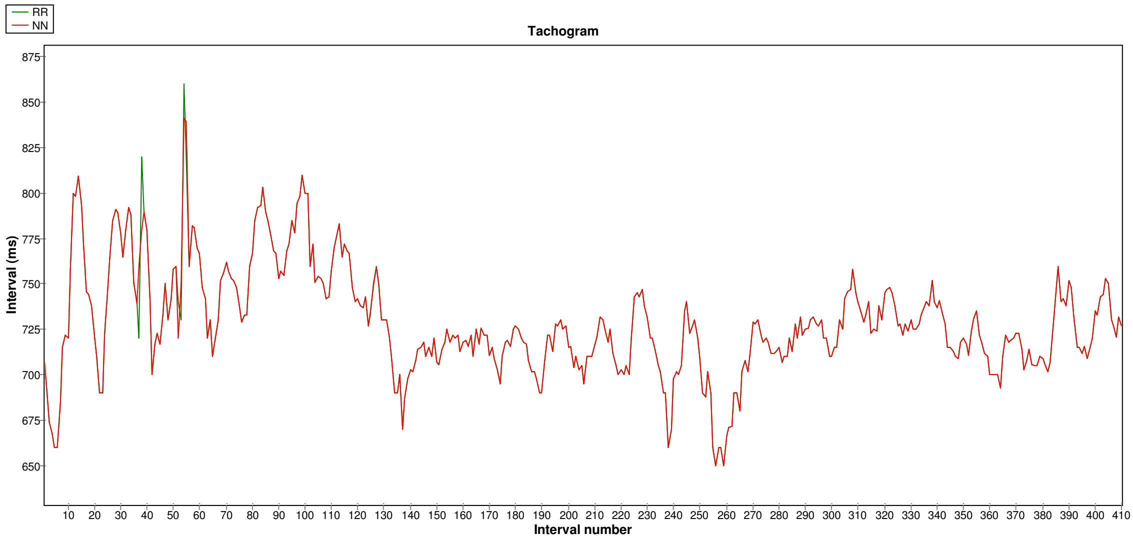

**HRV parameters**	**NN**	**RR**
TP (ms2)	434	434
VLF (ms2)	219	219
LF (ms2)	189	189
HF (ms2)	25	25
LF/HF	7.52	7.48
LF normalized	88.26	88.21
HF normalized	11.74	11.79
VLF peak (mHz)	7	7
LF peak (mHz)	83	83
HF peak (mHz)	337	337

**HRV spectral settings**	
Spectrum of Intervals (SOI)	
Frequency resolution (mHz)	3
VLF lower boundary (mHz)	3
VLF upper boundary (mHz)	40
LF upper boundary (mHz)	150
HF upper boundary (mHz)	400
Smoothing factor	1
Tapering	Hann
Fourier transform	DFT
Sample frequency (Hz)	1.37
Interval correction	Annotation
Interval threshold (%)	10
