# Supplementary material for: The effects of a 3-day mountain bike cycling race on the autonomic nervous system (ANS) and heart rate variability in amateur cyclists: a prospective quantitative research design
Source: BMC Sports Sci Med Rehabil. 2023 Jan 2;15:2. doi: 10.1186/s13102-022-00614-y (PMC9808932; doi:10.1186/s13102-022-00614-y)
Supplement: Supplementary file 1 — Additional file 1. Individual data of Participants. [file 13102_2022_614_MOESM1_ESM.zip › Individual data of Participants/HRV Data/012/ECG_012_20180504125205_.PDF]

Anton Swart Biokinetic Rehabilitation Practice

Name:	013 013	Recorded:	04/05/2018 12:52:05	UNCONFIRMED INTERPRETATION - MD SHOULD REVIEW
Number:	013	Recorded by:	Mr. Anton Swart	
Gender:	Male	Referring physician:		
Birthdate:	04/02/1971 47 years	Ordering physician:		
		Attending physician:		
		Location:	Anton Swart Biokinetic Rehabilitation Practi	
		Comment:		

P / PQ:	123 ms / 180 ms
QRS:	98 ms
QT / QTc / QTd:	365 ms / 430 ms / -
P/QRS/T axis:	75° / 90° / 65°
Heartrate:	97 bpm

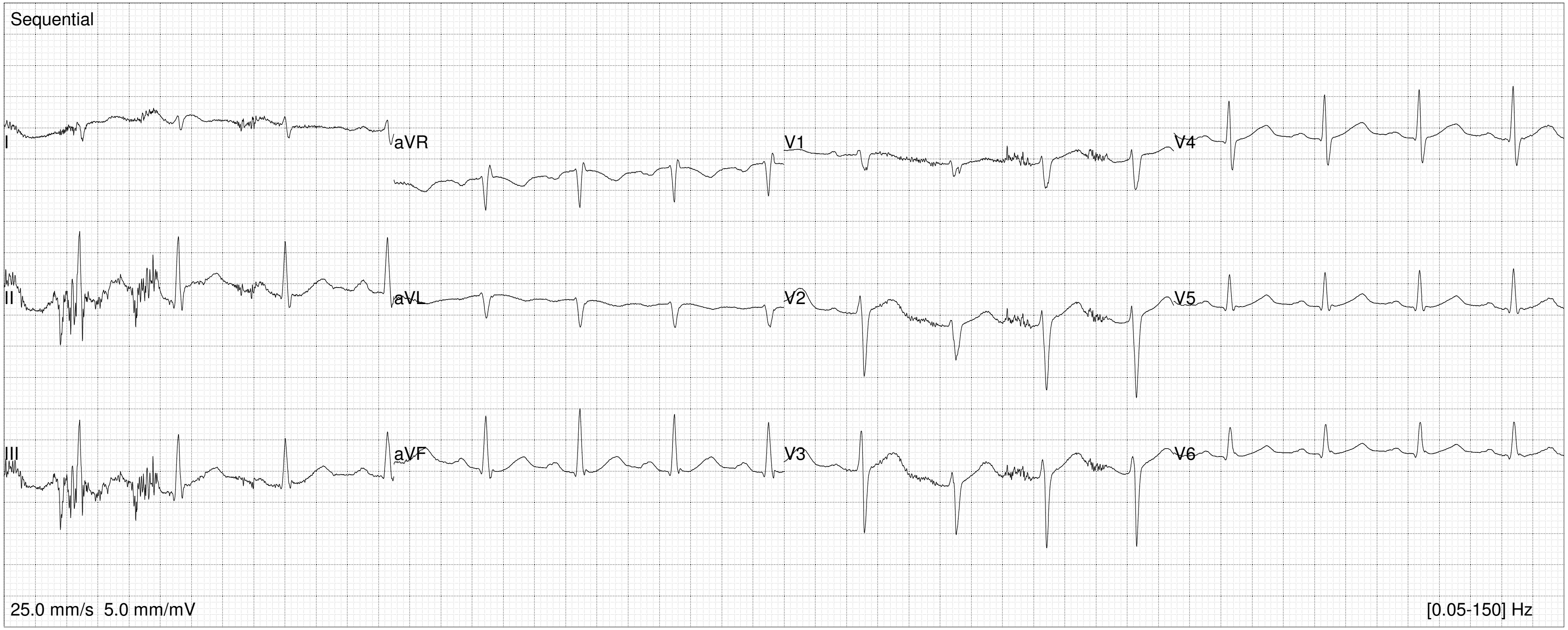

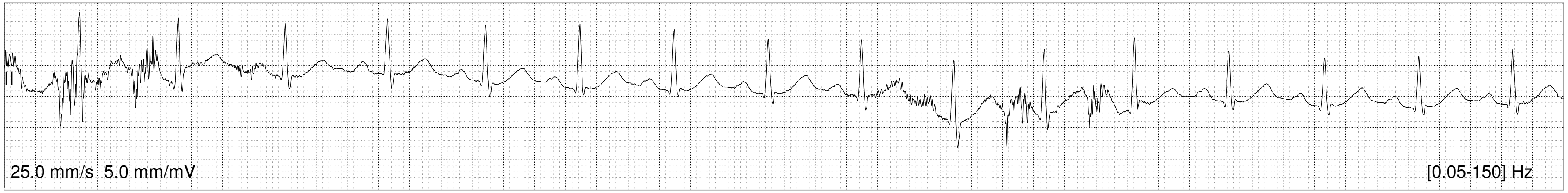

Anton Swart Biokinetic Rehabilitation Practice

Name:	013 013	Recorded:	04/05/2018 12:52:05	UNCONFIRMED INTERPRETATION - MD SHOULD REVIEW
Number:	013	Recorded by:	Mr. Anton Swart	
Gender:	Male	Referring physician:		
Birthdate:	04/02/1971 47 years	Location:	Anton Swart Biokinetic Rehabilitation Practice	
P / PQ:	123 ms / 180 ms	Ordering physician:		
QRS:	98 ms	Attending physician:		
QT / QTc / QTd:	365 ms / 430 ms / -	Comment:		
P/QRS/T axis:	75° / 90° / 65°			
Heartrate:	97 bpm			

**Beats**		**RR**	
Total:	478	Minimum	570 ms
Normal:	478	Maximum	1620 ms
Other:	0	Mean:	628 ms
		SD:	49 ms

R-R Trend

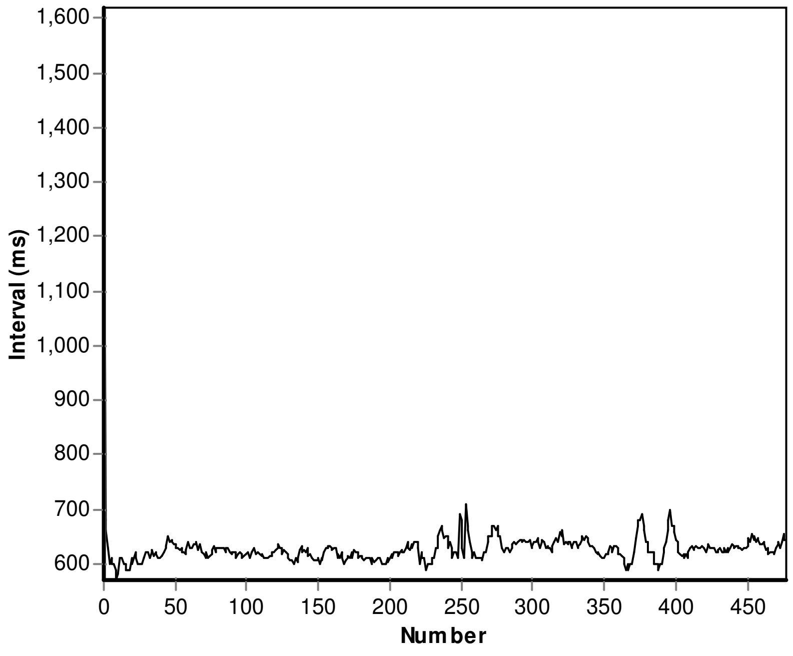

R-R Histogram

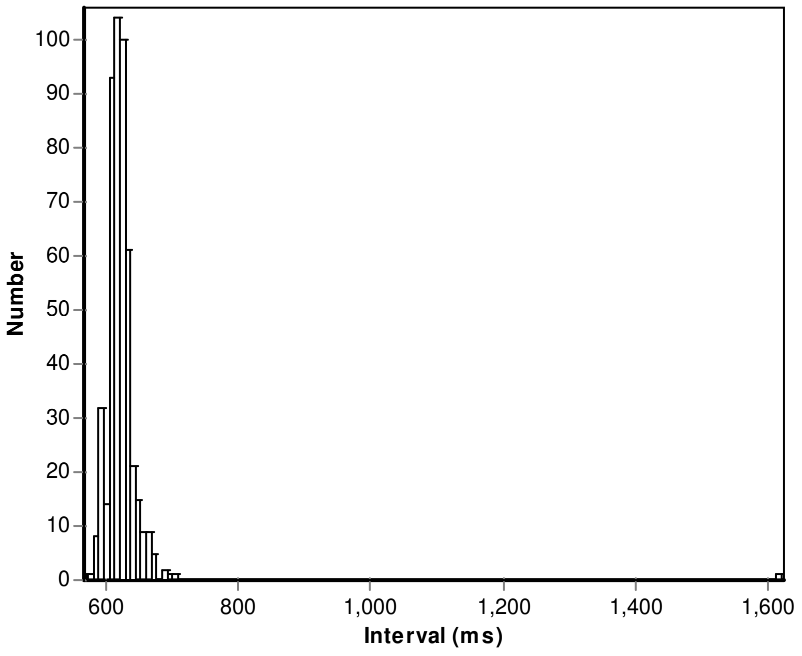

Cardio Control NV. User: Anton

Heart Rate Variability: Time Domain Analysis

**Name:**	013, 013	**Birthdate:**	04/02/1971
**Number:**	013	**Recorded:**	04/05/2018 12:52:05
**Gender:**	Male		

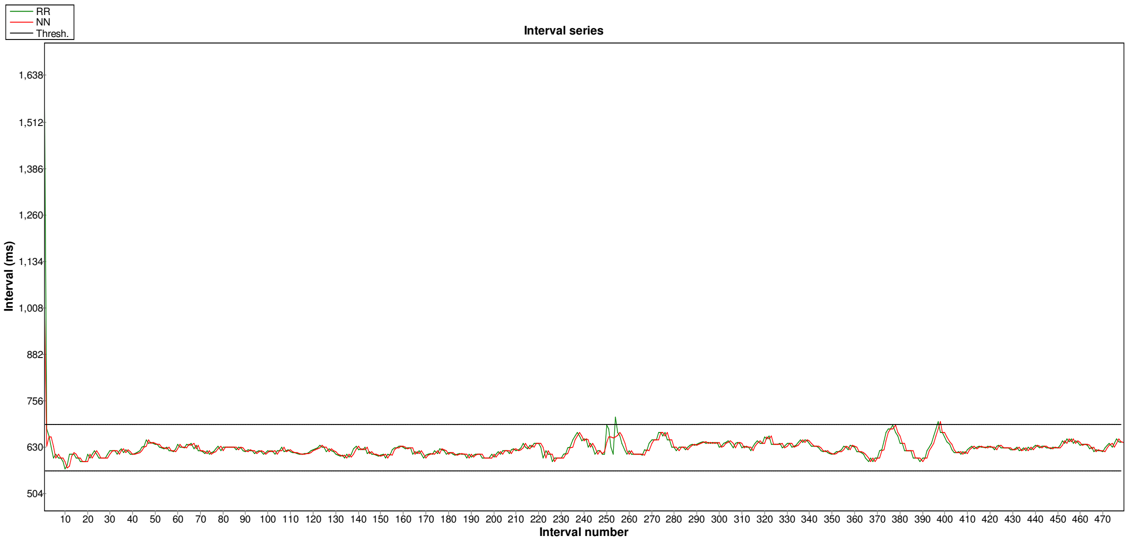

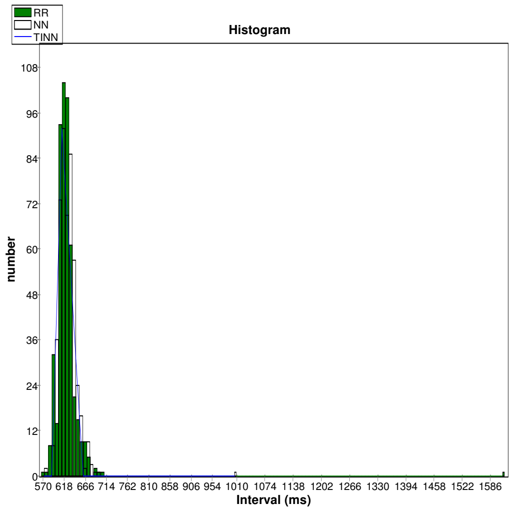

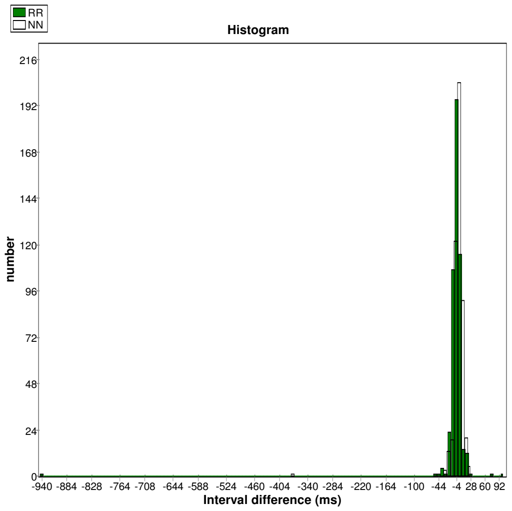

Binsize (ms) = 8

**HRV parameters**	**NN**	**RR**
SDNN (ms)	25	49
Triangular Interpolation (ms)	72	72
Triangular Index	5.21	4.60

**HRV parameters**	**NN**	**RR**
SDSD (ms)	19	44
RMSSD (ms)	19	44
NN50	1	3
NN50(1)	1	1
NN50(2)	0	2
pNN50	0.00	0.01
pNN50(1)	0.00	0.00
pNN50(2)	0.00	0.00
Logarithmic Index	1.28	0.77
SD(Logarithmic Index)	0.12	0.14

**Interval statistics**	**NN**	**RR**
Number	479	478
Minimum (ms)	573	570
Maximum (ms)	1010	1620
Range (ms)	437	1050
Avg (ms)	626	628
SD (ms)	25	49
AvgDev (ms)	14	16
p5 (ms)	600	600
p50 (ms)	624	624
p95 (ms)	660	660
Skewness	7.96	17.50
Kurtosis	121.96	354.54

**Interval statistics**	**NN**	**RR**
Number	478	477
Minimum (ms)	-378	-940
Maximum (ms)	27	100
Range (ms)	405	1040
Avg (ms)	-1	-2
SD (ms)	19	44
AvgDev (ms)	7	9
p5 (ms)	-14	-17
p50 (ms)	0	0
p95 (ms)	14	14
Skewness	-15.44	-19.79
Kurtosis	301.11	419.30

Cardio Control NV. User: Anton

Heart Rate Variability: Frequency Domain Analysis

**Name:**	013, 013	**Birthdate:**	04/02/1971
**Number:**	013	**Recorded:**	04/05/2018 12:52:05
**Gender:**	Male		

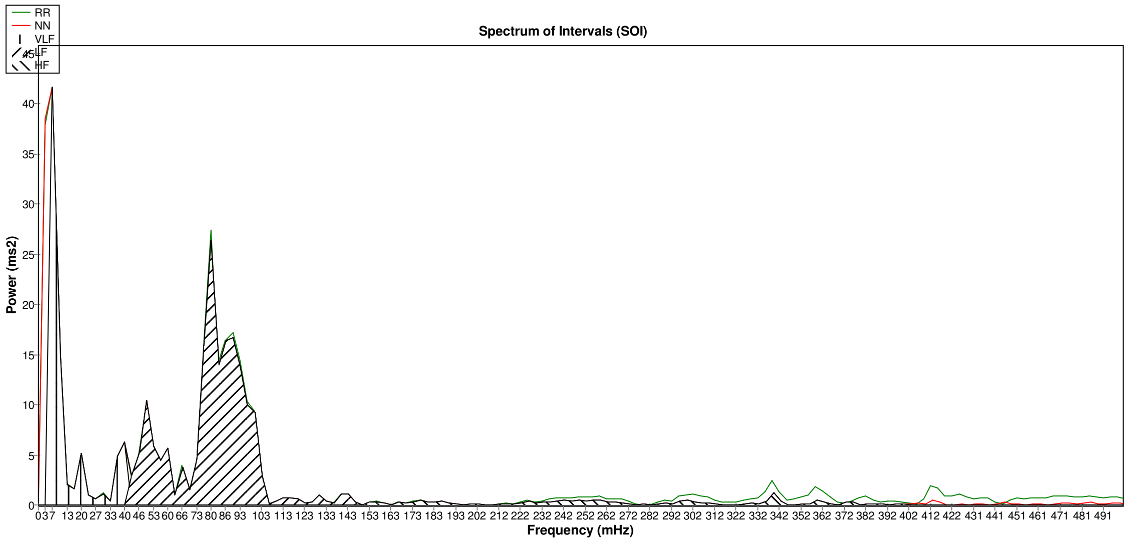

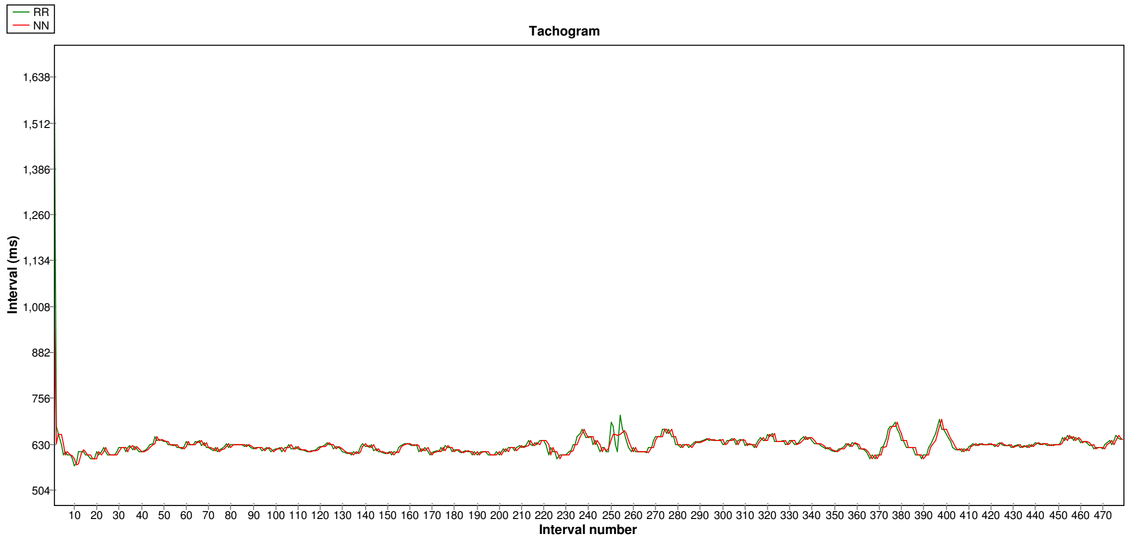

**HRV parameters**	**NN**	**RR**
TP (ms2)	274	299
VLF (ms2)	79	79
LF (ms2)	178	180
HF (ms2)	17	40
LF/HF	10.72	4.48
LF normalized	91.47	81.74
HF normalized	8.53	18.26
VLF peak (mHz)	7	7
LF peak (mHz)	80	80
HF peak (mHz)	339	339

**HRV spectral settings**	
Spectrum of Intervals (SOI)	
Frequency resolution (mHz)	3
VLF lower boundary (mHz)	3
VLF upper boundary (mHz)	40
LF upper boundary (mHz)	150
HF upper boundary (mHz)	400
Smoothing factor	1
Tapering	Hann
Fourier transform	DFT
Sample frequency (Hz)	1.60
Interval correction	Annotation
Interval threshold (%)	10
