# Supplementary material for: The effects of a 3-day mountain bike cycling race on the autonomic nervous system (ANS) and heart rate variability in amateur cyclists: a prospective quantitative research design
Source: BMC Sports Sci Med Rehabil. 2023 Jan 2;15:2. doi: 10.1186/s13102-022-00614-y (PMC9808932; doi:10.1186/s13102-022-00614-y)
Supplement: Supplementary file 1 — Additional file 1. Individual data of Participants. [file 13102_2022_614_MOESM1_ESM.zip › Individual data of Participants/HRV Data/012/ECG_012_20180505124721_.PDF]

Anton Swart Biokinetic Rehabilitation Practice

Name:	013 013	Recorded:	05/05/2018 12:47:21	UNCONFIRMED INTERPRETATION - MD SHOULD REVIEW
Number:	013	Recorded by:	Mr. Anton Swart	
Gender:	Male	Referring physician:		
Birthdate:	04/02/1971 47 years	Ordering physician:		
		Attending physician:		
P / PQ:	122 ms / 182 ms	Location:	Anton Swart Biokinetic Rehabilitation Practi	
QRS:	96 ms	Comment:		
QT / QTc / QTd:	366 ms / 432 ms / -			
P/QRS/T axis:	74° / 92° / 66°			
Heartrate:	98 bpm			

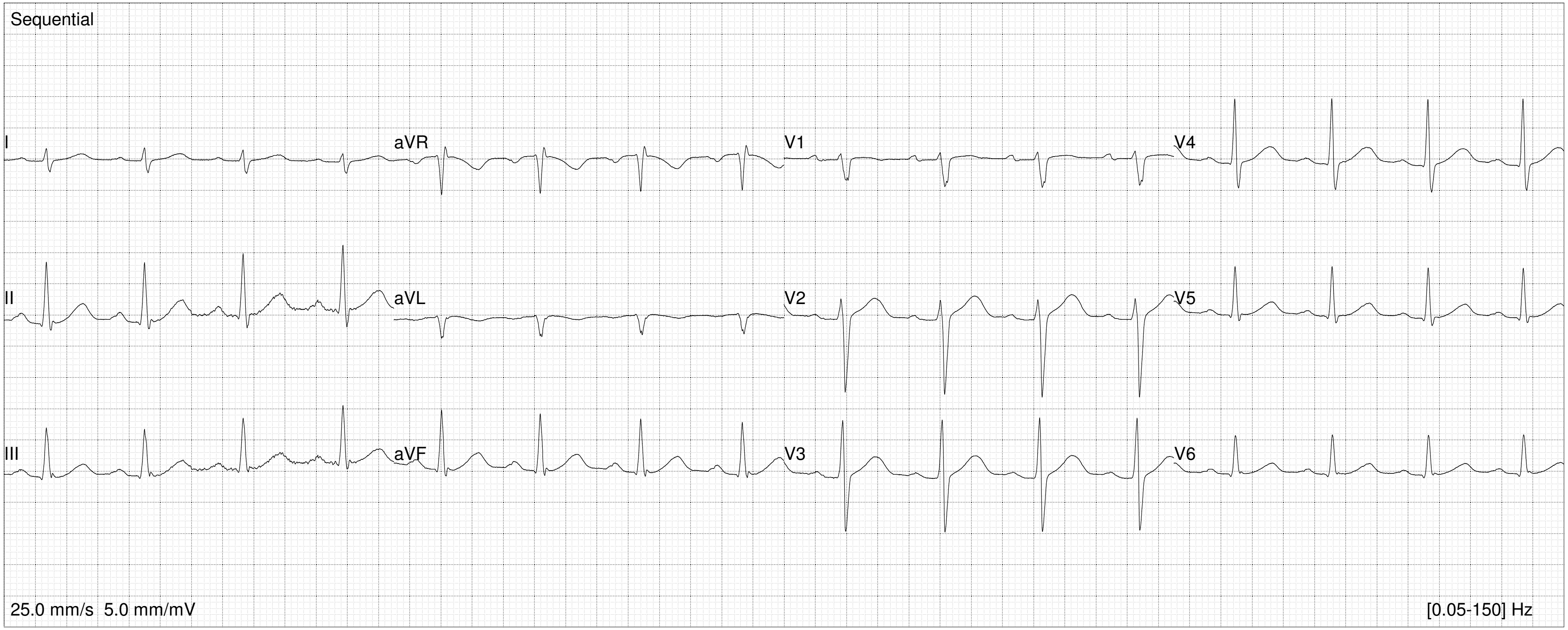

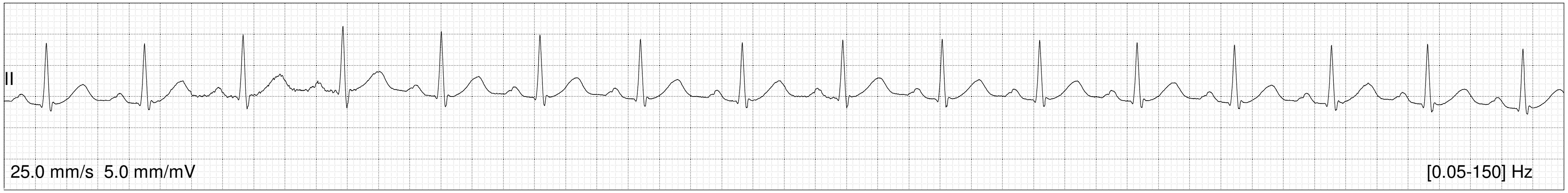

Anton Swart Biokinetic Rehabilitation Practice

Name:	013 013	Recorded:	05/05/2018 12:47:21	UNCONFIRMED INTERPRETATION - MD SHOULD REVIEW
Number:	013	Recorded by:	Mr. Anton Swart	
Gender:	Male	Referring physician:		
Birthdate:	04/02/1971 47 years	Location:	Anton Swart Biokinetic Rehabilitation Practice	
		Ordering physician:		
		Attending physician:		
		Comment:		

P / PQ:	122 ms / 182 ms
QRS:	96 ms
QT / QTc / QTd:	366 ms / 432 ms / -
P/QRS/T axis:	74° / 92° / 66°
Heartrate:	98 bpm

Beats

Total:	489
Normal:	489
Other:	0

RR

Minimum	580 ms
Maximum	650 ms
Mean:	612 ms
SD:	11 ms

R-R Trend

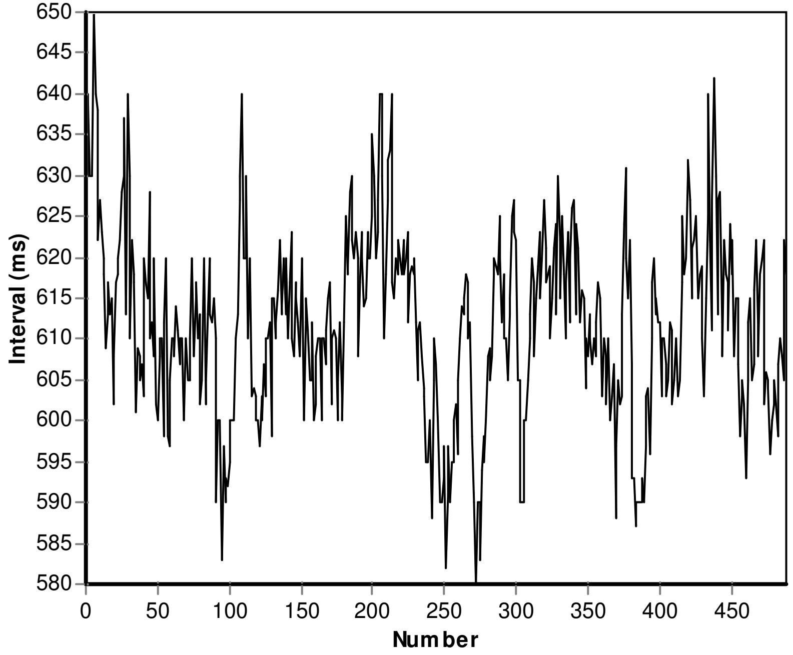

R-R Histogram

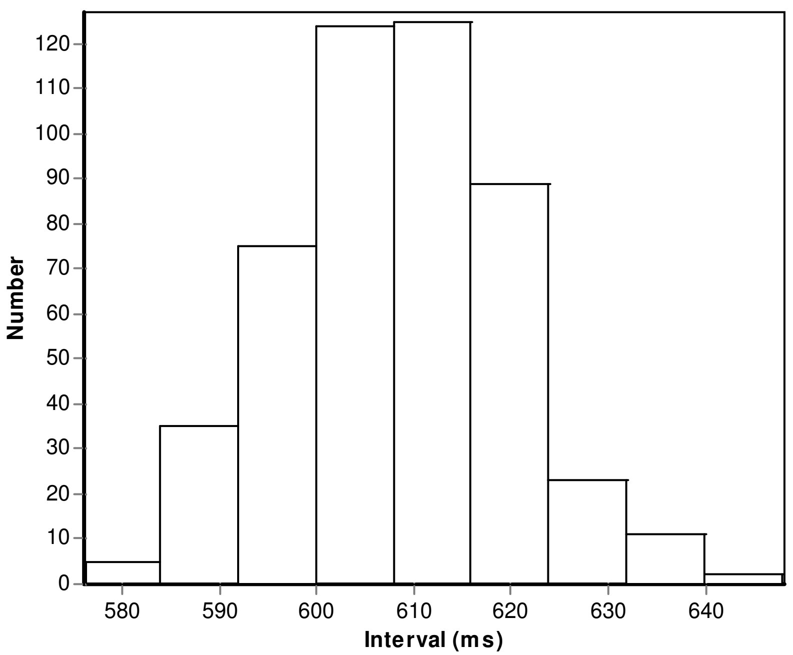

Cardio Control NV. User: Anton

Heart Rate Variability: Time Domain Analysis

**Name:**	013, 013	**Birthdate:**	04/02/1971
**Number:**	013	**Recorded:**	05/05/2018 12:47:21
**Gender:**	Male		

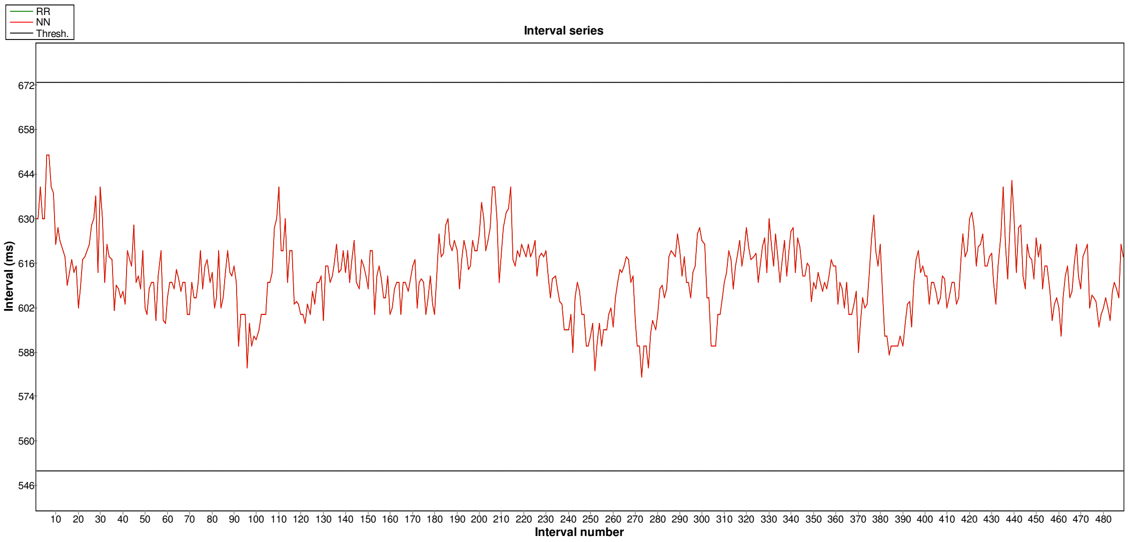

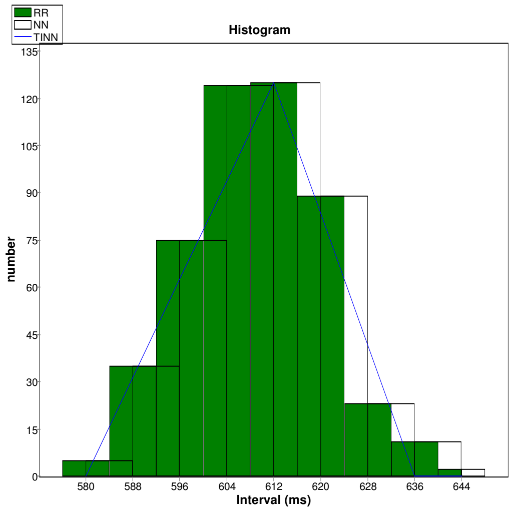

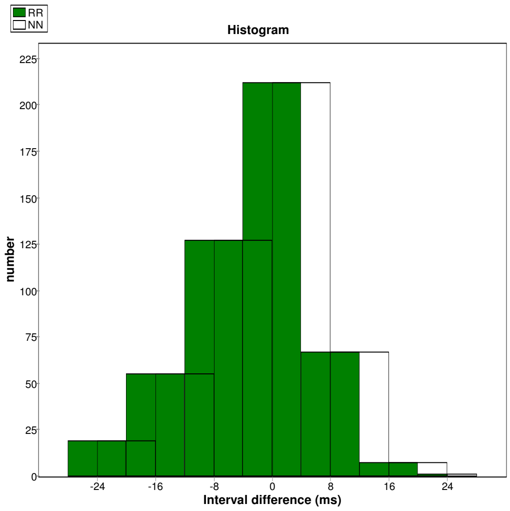

Binsize (ms) = 8

**HRV parameters**	**NN**	**RR**
SDNN (ms)	11	11
Triangular Interpolation (ms)	56	56
Triangular Index	3.91	3.91

**HRV parameters**	**NN**	**RR**
SDSD (ms)	8	8
RMSSD (ms)	8	8
NN50	0	0
NN50(1)	0	0
NN50(2)	0	0
pNN50	0.00	0.00
pNN50(1)	0.00	0.00
pNN50(2)	0.00	0.00
Logarithmic Index	1.68	1.68
SD(Logarithmic Index)	0.27	0.27

**Interval statistics**	**NN**	**RR**
Number	489	489
Minimum (ms)	580	580
Maximum (ms)	650	650
Range (ms)	70	70
Avg (ms)	612	612
SD (ms)	11	11
AvgDev (ms)	9	9
p5 (ms)	591	591
p50 (ms)	612	612
p95 (ms)	630	630
Skewness	0.11	0.11
Kurtosis	3.33	3.33

**Interval statistics**	**NN**	**RR**
Number	488	488
Minimum (ms)	-24	-24
Maximum (ms)	27	27
Range (ms)	51	51
Avg (ms)	-0	-0
SD (ms)	8	8
AvgDev (ms)	6	6
p5 (ms)	-15	-15
p50 (ms)	0	0
p95 (ms)	12	12
Skewness	-0.35	-0.35
Kurtosis	3.26	3.26

Heart Rate Variability: Frequency Domain Analysis

**Name:**	013, 013	**Birthdate:**	04/02/1971
**Number:**	013	**Recorded:**	05/05/2018 12:47:21
**Gender:**	Male		

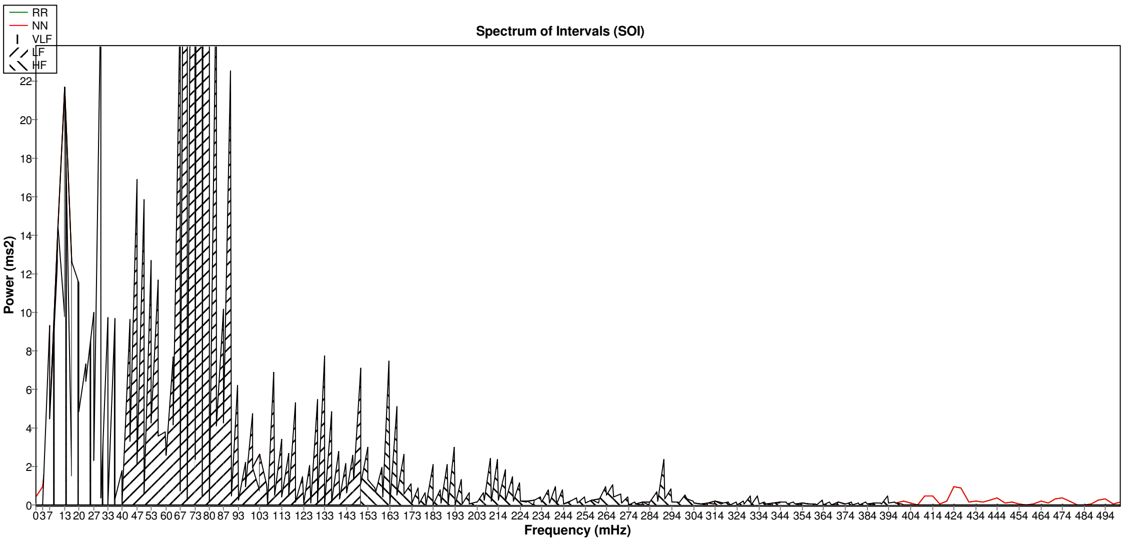

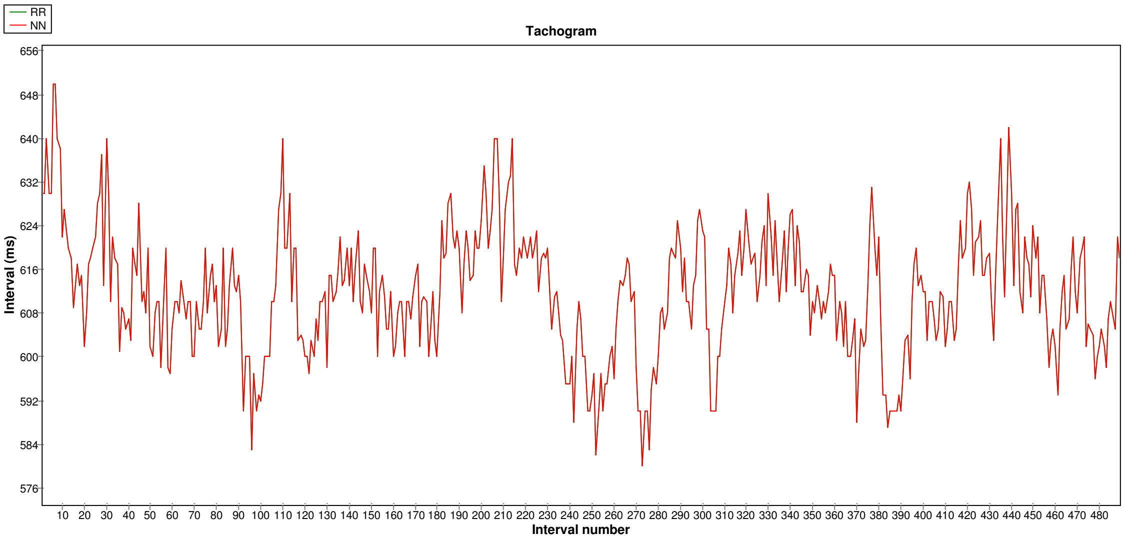

HRV parameters	NN	RR
TP (ms2)	125	125
VLF (ms2)	68	68
LF (ms2)	44	44
HF (ms2)	13	13
LF/HF	3.31	3.31
LF normalized	76.80	76.80
HF normalized	23.20	23.20
VLF peak (mHz)	13	13
LF peak (mHz)	87	87
HF peak (mHz)	153	153

HRV spectral settings	
Spectrum of Intervals (SOI)	
Frequency resolution (mHz)	3
VLF lower boundary (mHz)	3
VLF upper boundary (mHz)	40
LF upper boundary (mHz)	150
HF upper boundary (mHz)	400
Smoothing factor	1
Tapering	Hann
Fourier transform	DFT
Sample frequency (Hz)	1.63
Interval correction	Annotation
Interval threshold (%)	10
