# Supplementary material for: The effects of a 3-day mountain bike cycling race on the autonomic nervous system (ANS) and heart rate variability in amateur cyclists: a prospective quantitative research design
Source: BMC Sports Sci Med Rehabil. 2023 Jan 2;15:2. doi: 10.1186/s13102-022-00614-y (PMC9808932; doi:10.1186/s13102-022-00614-y)
Supplement: Supplementary file 1 — Additional file 1. Individual data of Participants. [file 13102_2022_614_MOESM1_ESM.zip › Individual data of Participants/HRV Data/012/ECG_012_20180506070104_.PDF]

Anton Swart Biokinetic Rehabilitation Practice

Name:	013 013	Recorded:	06/05/2018 07:01:04	UNCONFIRMED INTERPRETATION - MD SHOULD REVIEW
Number:	013	Recorded by:	Mr. Anton Swart	
Gender:	Male	Referring physician:		
Birthdate:	04/02/1971 47 years	Ordering physician:		
		Attending physician:		
P / PQ:	127 ms / 203 ms	Location:	Anton Swart Biokinetic Rehabilitation Practi	
QRS:	97 ms	Comment:		
QT / QTc / QTd:	427 ms / 438 ms / -			
P/QRS/T axis:	69° / 96° / 71°			
Heartrate:	66 bpm			

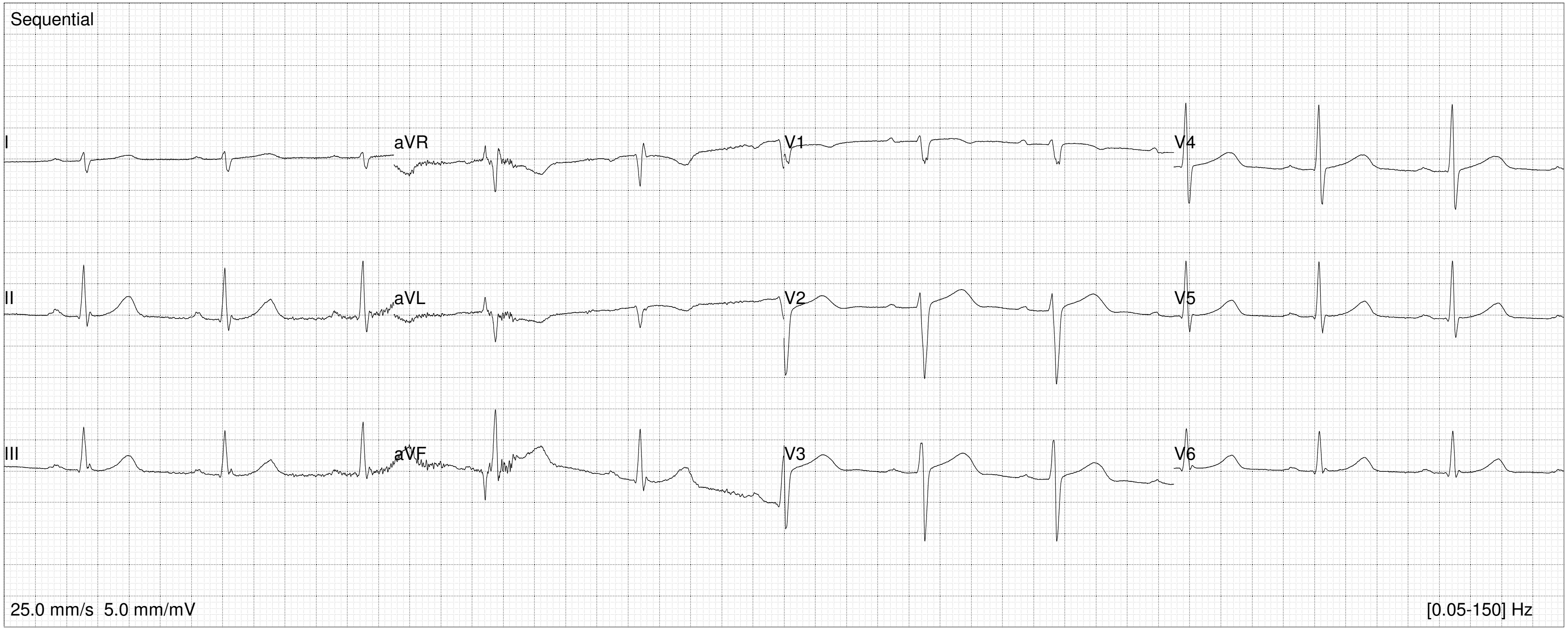

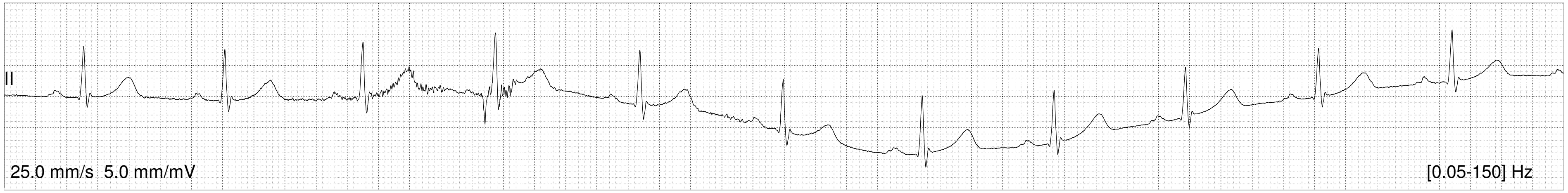

Anton Swart Biokinetic Rehabilitation Practice

Name:	013 013	Recorded:	06/05/2018 07:01:04	UNCONFIRMED INTERPRETATION - MD SHOULD REVIEW
Number:	013	Recorded by:	Mr. Anton Swart	
Gender:	Male	Referring physician:		
Birthdate:	04/02/1971 47 years	Location:	Anton Swart Biokinetic Rehabilitation Practice	
		Ordering physician:		
		Attending physician:		
		Comment:		

P / PQ:	127 ms / 203 ms
QRS:	97 ms
QT / QTc / QTd:	427 ms / 438 ms / -
P/QRS/T axis:	69° / 96° / 71°
Heartrate:	66 bpm

**Beats**		**RR**	
Total:	332	Minimum	340 ms
Normal:	332	Maximum	985 ms
Other:	0	Mean:	901 ms
		SD:	55 ms

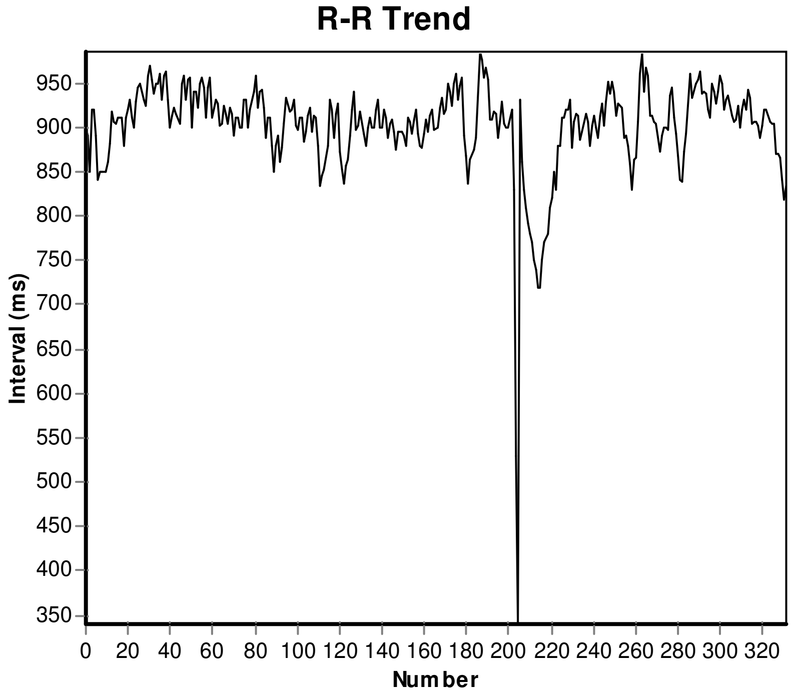

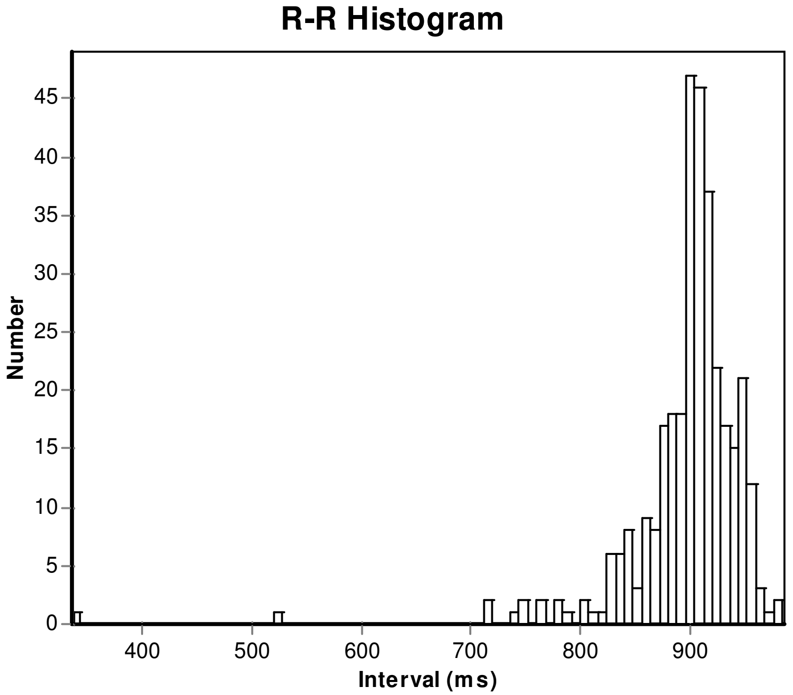

Cardio Control NV. User: Anton

Heart Rate Variability: Time Domain Analysis

**Name:**	013, 013	**Birthdate:**	04/02/1971
**Number:**	013	**Recorded:**	06/05/2018 07:01:04
**Gender:**	Male		

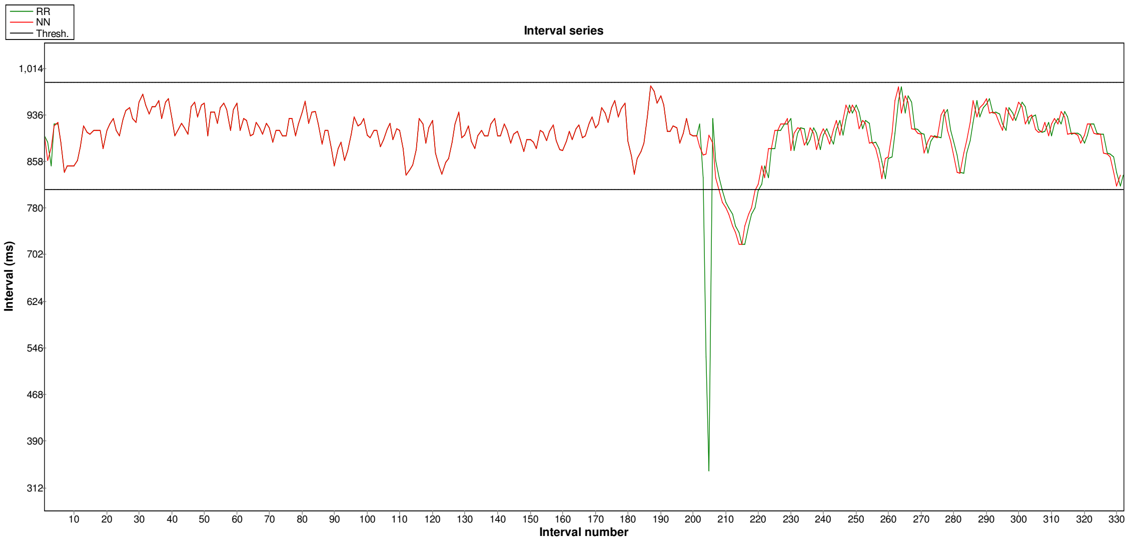

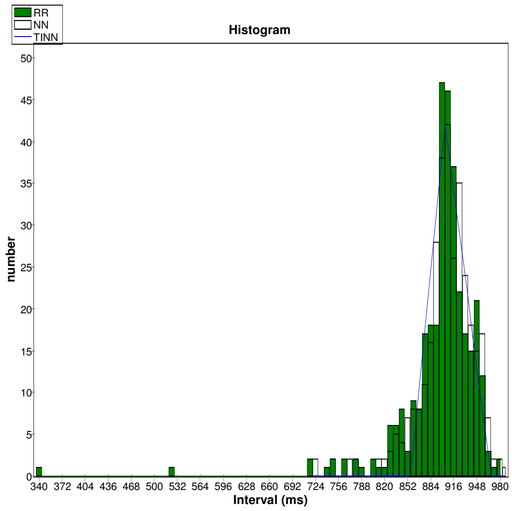

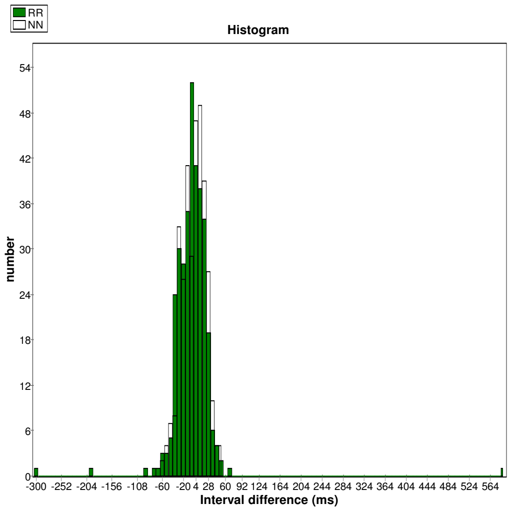

Binsize (ms) = 8

HRV parameters	NN	RR
SDNN (ms)	41	55
Triangular Interpolation (ms)	112	104
Triangular Index	7.88	7.06

HRV parameters	NN	RR
SDSD (ms)	22	44
RMSSD (ms)	22	44
NN50	8	13
NN50(1)	5	8
NN50(2)	3	5
pNN50	0.02	0.04
pNN50(1)	0.02	0.02
pNN50(2)	0.01	0.02
Logarithmic Index	0.61	0.50
SD(Logarithmic Index)	0.08	0.04

Interval statistics	NN	RR
Number	331	332
Minimum (ms)	720	340
Maximum (ms)	985	985
Range (ms)	265	645
Avg (ms)	904	901
SD (ms)	41	55
AvgDev (ms)	29	32
p5 (ms)	830	825
p50 (ms)	910	910
p95 (ms)	957	957
Skewness	-1.52	-4.59
Kurtosis	6.91	39.90

Interval statistics	NN	RR
Number	330	331
Minimum (ms)	-64	-300
Maximum (ms)	55	590
Range (ms)	119	890
Avg (ms)	-0	-0
SD (ms)	22	44
AvgDev (ms)	18	21
p5 (ms)	-35	-36
p50 (ms)	0	0
p95 (ms)	34	34
Skewness	-0.18	5.99
Kurtosis	2.70	104.12

Cardio Control NV. User: Anton

Heart Rate Variability: Frequency Domain Analysis

**Name:**	013, 013	**Birthdate:**	04/02/1971
**Number:**	013	**Recorded:**	06/05/2018 07:01:04
**Gender:**	Male		

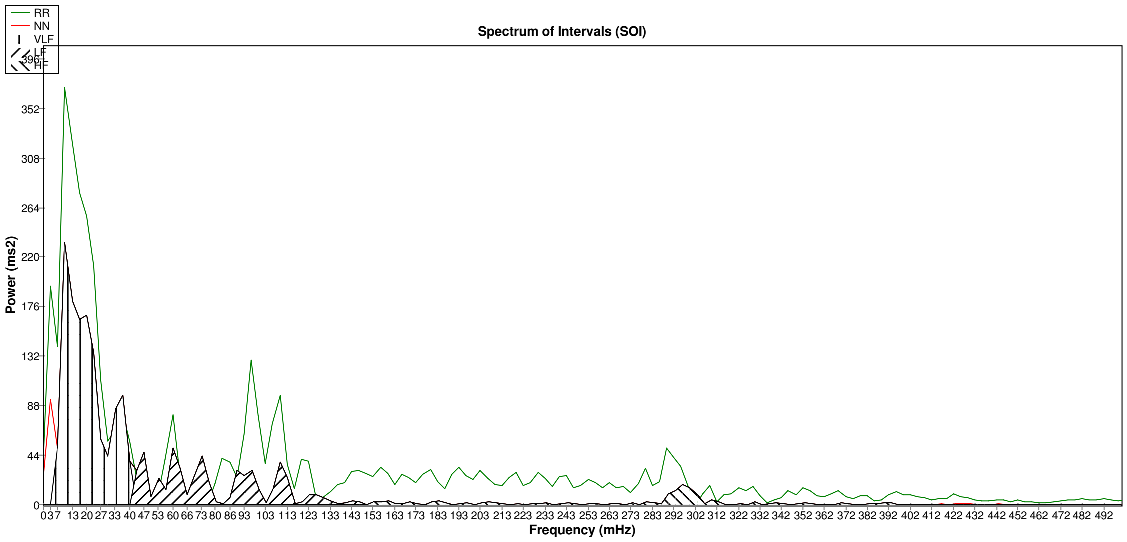

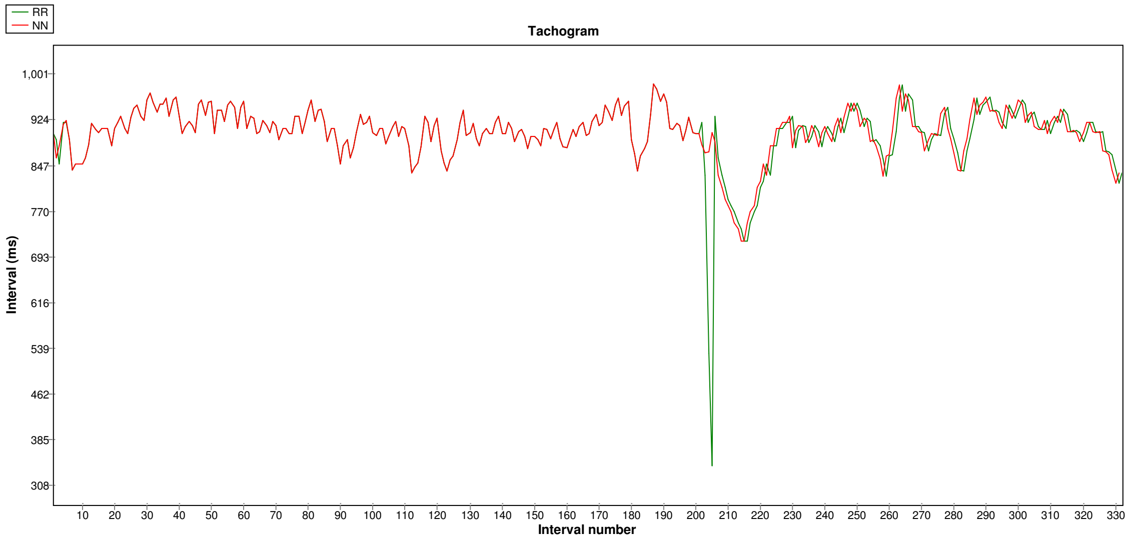

HRV parameters	NN	RR
TP (ms2)	1931	4395
VLF (ms2)	1256	1946
LF (ms2)	532	1124
HF (ms2)	143	1325
LF/HF	3.73	0.85
LF normalized	78.85	45.89
HF normalized	21.15	54.11
VLF peak (mHz)	10	10
LF peak (mHz)	60	96
HF peak (mHz)	297	289

HRV spectral settings	
Spectrum of Intervals (SOI)	
Frequency resolution (mHz)	3
VLF lower boundary (mHz)	3
VLF upper boundary (mHz)	40
LF upper boundary (mHz)	150
HF upper boundary (mHz)	400
Smoothing factor	1
Tapering	Hann
Fourier transform	DFT
Sample frequency (Hz)	1.11
Interval correction	Annotation
Interval threshold (%)	10
