# Supplementary material for: The effects of a 3-day mountain bike cycling race on the autonomic nervous system (ANS) and heart rate variability in amateur cyclists: a prospective quantitative research design
Source: BMC Sports Sci Med Rehabil. 2023 Jan 2;15:2. doi: 10.1186/s13102-022-00614-y (PMC9808932; doi:10.1186/s13102-022-00614-y)
Supplement: Supplementary file 1 — Additional file 1. Individual data of Participants. [file 13102_2022_614_MOESM1_ESM.zip › Individual data of Participants/HRV Data/013/ECG_013_20180501164657_.PDF]

Anton Swart Biokinetic Rehabilitation Practice

Name:	014 014 014	Recorded:	01/05/2018 16:46:57	UNCONFIRMED INTERPRETATION - MD SHOULD REVIEW
Number:	014	Recorded by:	Mr. Anton Swart	
Gender:	Male	Referring physician:		
Birthdate:	13/06/1972 45 years	Ordering physician:		
		Attending physician:		
P / PQ:	82 ms / 157 ms	Location:	Anton Swart Biokinetic Rehabilitation Practi	
QRS:	93 ms	Comment:		
QT / QTc / QTd:	410 ms / 412 ms / -			
P/QRS/T axis:	29° / 80° / 64°			
Heartrate:	61 bpm			

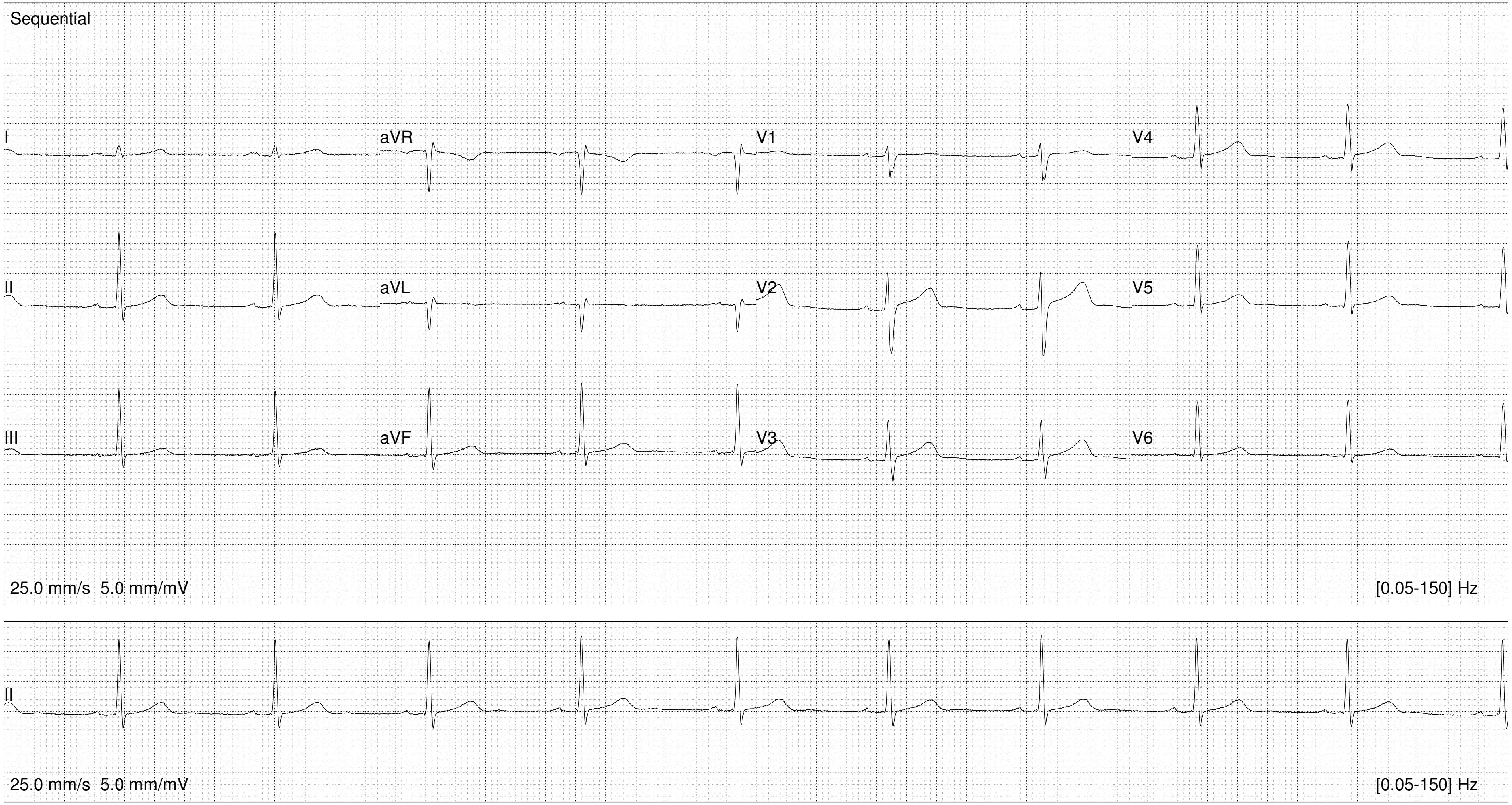

Anton Swart Biokinetic Rehabilitation Practice

Name:	014 014 014	Recorded:	01/05/2018 16:46:57	UNCONFIRMED INTERPRETATION - MD SHOULD REVIEW
Number:	014	Recorded by:	Mr. Anton Swart	
Gender:	Male	Referring physician:		
Birthdate:	13/06/1972 45 years	Location:	Anton Swart Biokinetic Rehabilitation Practice	
		Ordering physician:		
		Attending physician:		
		Comment:		

P / PQ:	82 ms / 157 ms
QRS:	93 ms
QT / QTc / QTd:	410 ms / 412 ms / -
P/QRS/T axis:	29° / 80° / 64°
Heartrate:	61 bpm

**Beats**		**RR**	
Total:	305	Minimum	911 ms
Normal:	305	Maximum	1050 ms
Other:	0	Mean:	980 ms
		SD:	28 ms

R-R Trend

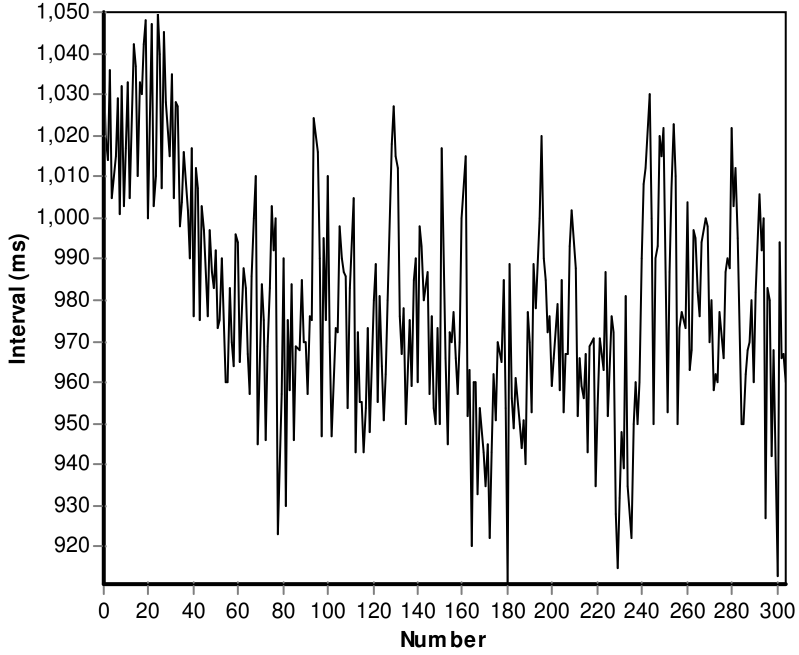

R-R Histogram

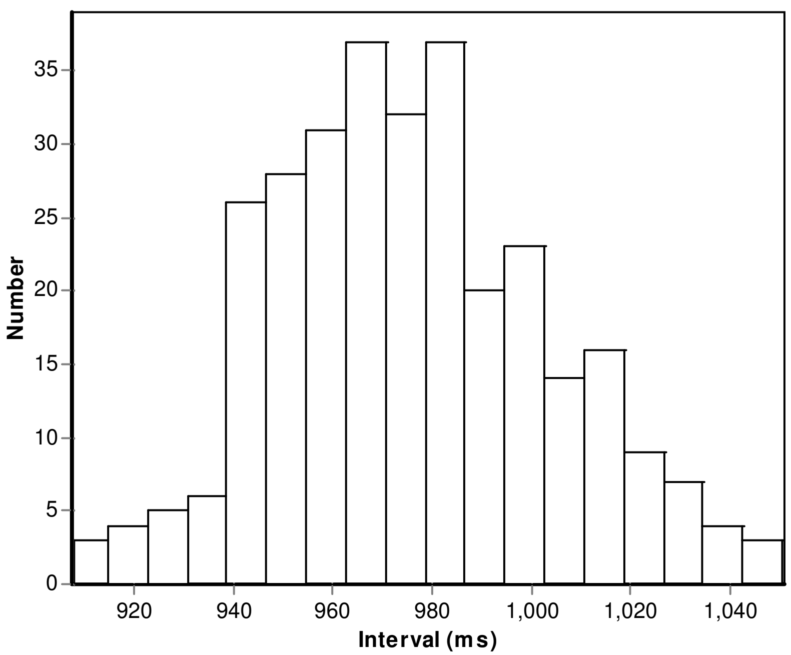

Cardio Control NV. User: Anton

Heart Rate Variability: Time Domain Analysis

**Name:**	014, 014 014	**Birthdate:**	13/06/1972
**Number:**	014	**Recorded:**	01/05/2018 16:46:57
**Gender:**	Male		

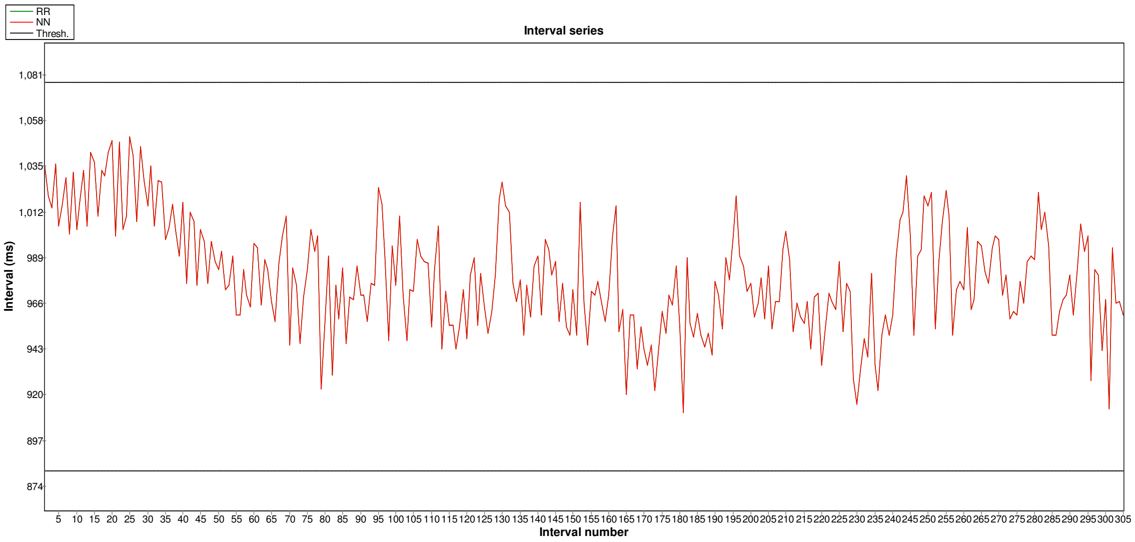

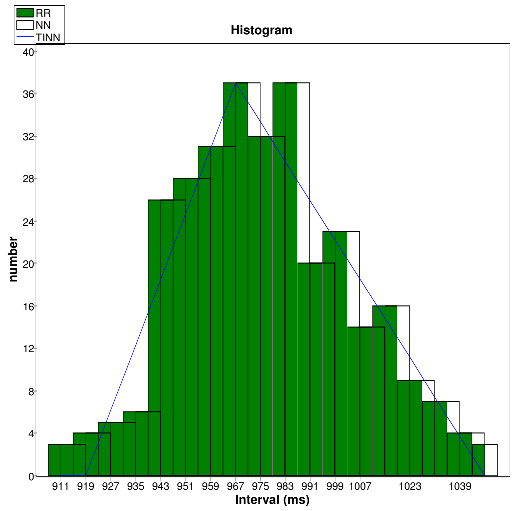

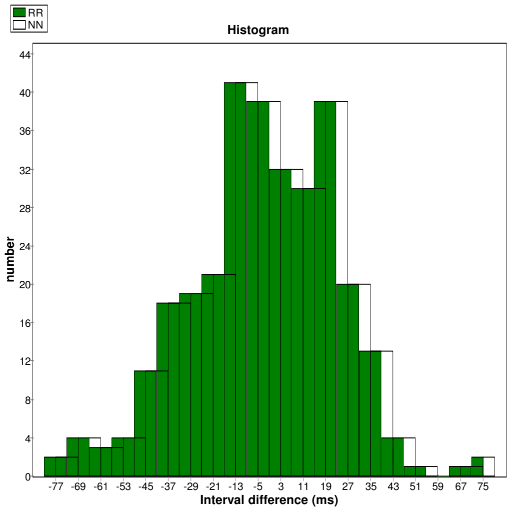

Binsize (ms) = 8

**HRV parameters**	**NN**	**RR**
SDNN (ms)	28	28
Triangular Interpolation (ms)	128	128
Triangular Index	8.24	8.24

**HRV parameters**	**NN**	**RR**
SDSD (ms)	26	26
RMSSD (ms)	26	26
NN50	14	14
NN50(1)	10	10
NN50(2)	4	4
pNN50	0.05	0.05
pNN50(1)	0.03	0.03
pNN50(2)	0.01	0.01
Logarithmic Index	0.44	0.44
SD(Logarithmic Index)	0.03	0.03

**Interval statistics**	**NN**	**RR**
Number	305	305
Minimum (ms)	911	911
Maximum (ms)	1050	1050
Range (ms)	139	139
Avg (ms)	980	980
SD (ms)	28	28
AvgDev (ms)	23	23
p5 (ms)	939	939
p50 (ms)	976	976
p95 (ms)	1030	1030
Skewness	0.24	0.24
Kurtosis	2.73	2.73

**Interval statistics**	**NN**	**RR**
Number	304	304
Minimum (ms)	-77	-77
Maximum (ms)	81	81
Range (ms)	158	158
Avg (ms)	-0	-0
SD (ms)	26	26
AvgDev (ms)	21	21
p5 (ms)	-44	-44
p50 (ms)	-1	-1
p95 (ms)	38	38
Skewness	-0.20	-0.20
Kurtosis	3.23	3.23

Cardio Control NV. User: Anton

Heart Rate Variability: Frequency Domain Analysis

**Name:**	014, 014 014	**Birthdate:**	13/06/1972
**Number:**	014	**Recorded:**	01/05/2018 16:46:57
**Gender:**	Male		

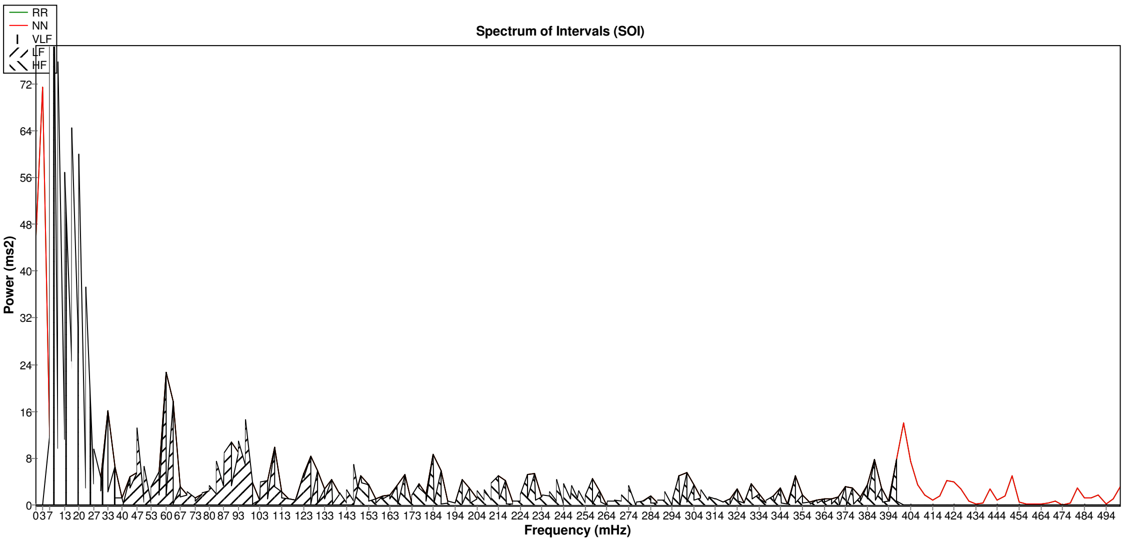

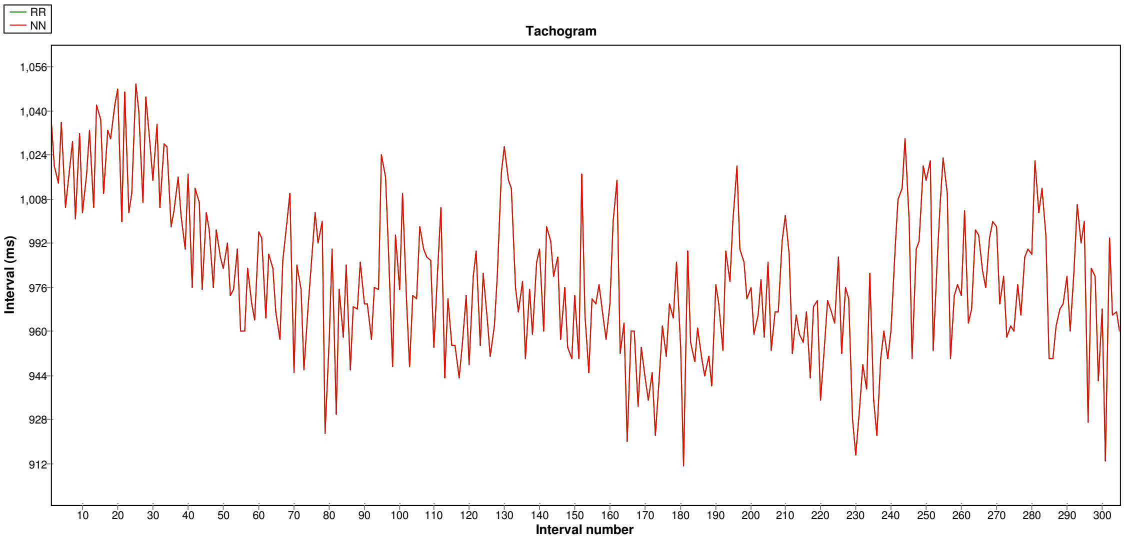

HRV parameters	NN	RR
TP (ms2)	436	436
VLF (ms2)	118	118
LF (ms2)	152	152
HF (ms2)	166	166
LF/HF	0.92	0.92
LF normalized	47.83	47.83
HF normalized	52.17	52.17
VLF peak (mHz)	20	20
LF peak (mHz)	60	60
HF peak (mHz)	184	184

HRV spectral settings	
Spectrum of Intervals (SOI)	
Frequency resolution (mHz)	3
VLF lower boundary (mHz)	3
VLF upper boundary (mHz)	40
LF upper boundary (mHz)	150
HF upper boundary (mHz)	400
Smoothing factor	1
Tapering	Hann
Fourier transform	DFT
Sample frequency (Hz)	1.02
Interval correction	Annotation
Interval threshold (%)	10
