# Supplementary material for: The effects of a 3-day mountain bike cycling race on the autonomic nervous system (ANS) and heart rate variability in amateur cyclists: a prospective quantitative research design
Source: BMC Sports Sci Med Rehabil. 2023 Jan 2;15:2. doi: 10.1186/s13102-022-00614-y (PMC9808932; doi:10.1186/s13102-022-00614-y)
Supplement: Supplementary file 1 — Additional file 1. Individual data of Participants. [file 13102_2022_614_MOESM1_ESM.zip › Individual data of Participants/HRV Data/013/ECG_013_20180503170749_.PDF]

# Anton Swart Biokinetic Rehabilitation Practice

| | | | | |
|---|---|---|---|---|
| Name: | 014 014 014 | Recorded: | 03/05/2018 17:07:49 | UNCONFIRMED INTERPRETATION - MD SHOULD REVIEW |
| Number: | 014 | Recorded by: | Mr. Anton Swart | |
| Gender: | Male | Referring physician: | | |
| Birthdate: | 13/06/1972 45 years | Ordering physician: | | |
| | | Attending physician: | | |
| P / PQ: | 112 ms / 173 ms | Location: | Anton Swart Biokinetic Rehabilitation Practi | |
| QRS: | 87 ms | Comment: | | |
| QT / QTc / QTd: | 359 ms / 415 ms / - | | | |
| P/QRS/T axis: | 76° / 78° / 50° | | | |
| Heartrate: | 92 bpm | | | |

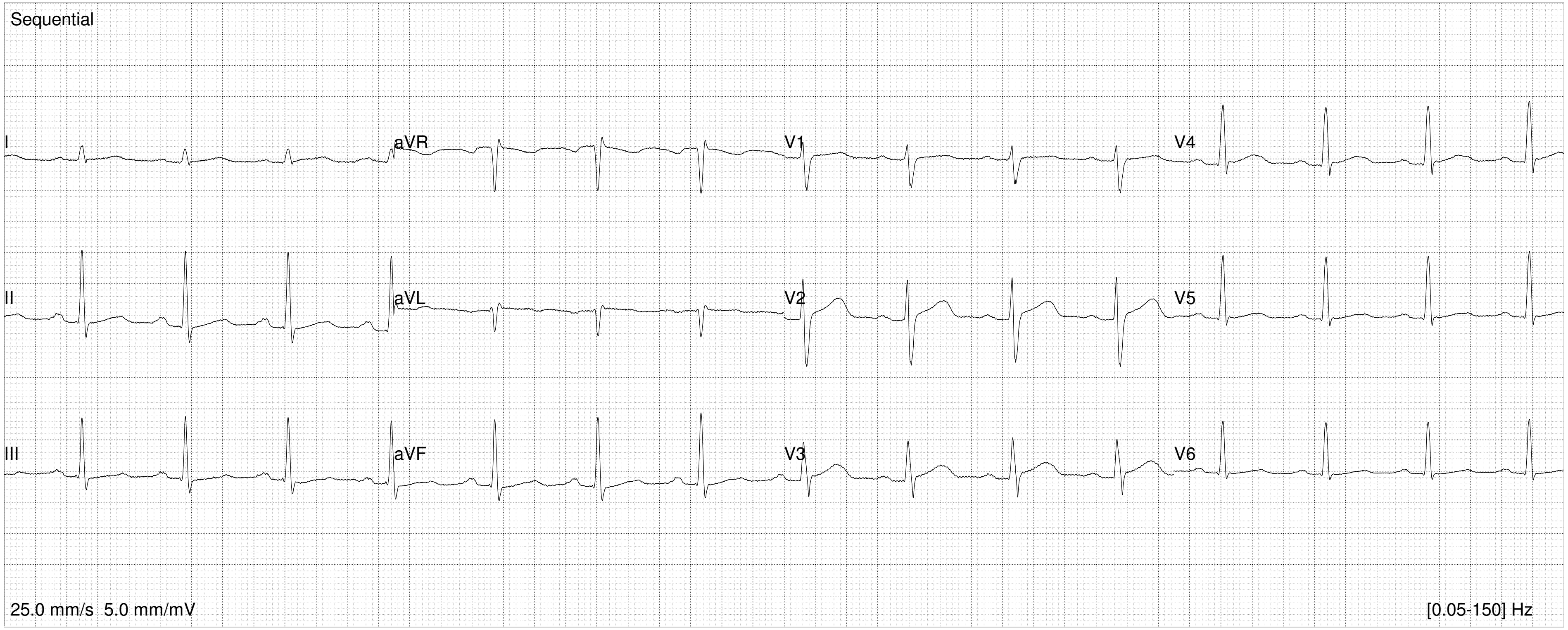


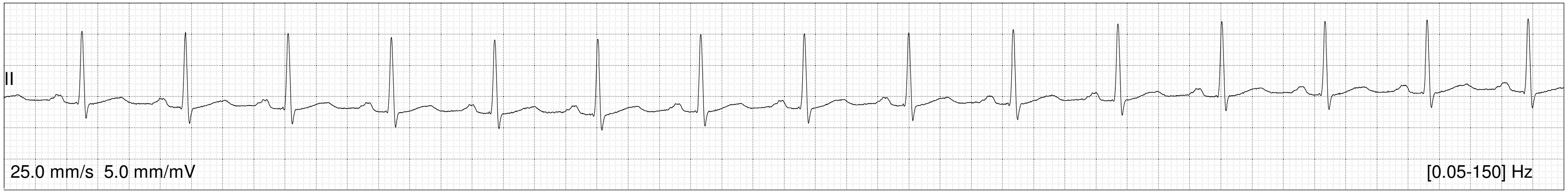

# Anton Swart Biokinetic Rehabilitation Practice

| | | | | |
|---|---|---|---|---|
| Name: | 014 014 014 | Recorded: | 03/05/2018 17:07:49 | UNCONFIRMED INTERPRETATION - MD SHOULD REVIEW |
| Number: | 014 | Recorded by: | Mr. Anton Swart | |
| Gender: | Male | Referring physician: | | |
| Birthdate: | 13/06/1972 45 years | Location: | Anton Swart Biokinetic Rehabilitation Practice | |
| | | Ordering physician: | | |
| | | Attending physician: | | |
| | | Comment: | | |

| | |
|---|---|
| P / PQ: | 112 ms / 173 ms |
| QRS: | 87 ms |
| QT / QTc / QTd: | 359 ms / 415 ms / - |
| P/QRS/T axis: | 76° / 78° / 50° |
| Heartrate: | 92 bpm |

| **Beats** | | **RR** | |
|---|---|---|---|
| Total: | 458 | Minimum | 590 ms |
| Normal: | 458 | Maximum | 720 ms |
| Other: | 0 | Mean: | 653 ms |
| | | SD: | 20 ms |

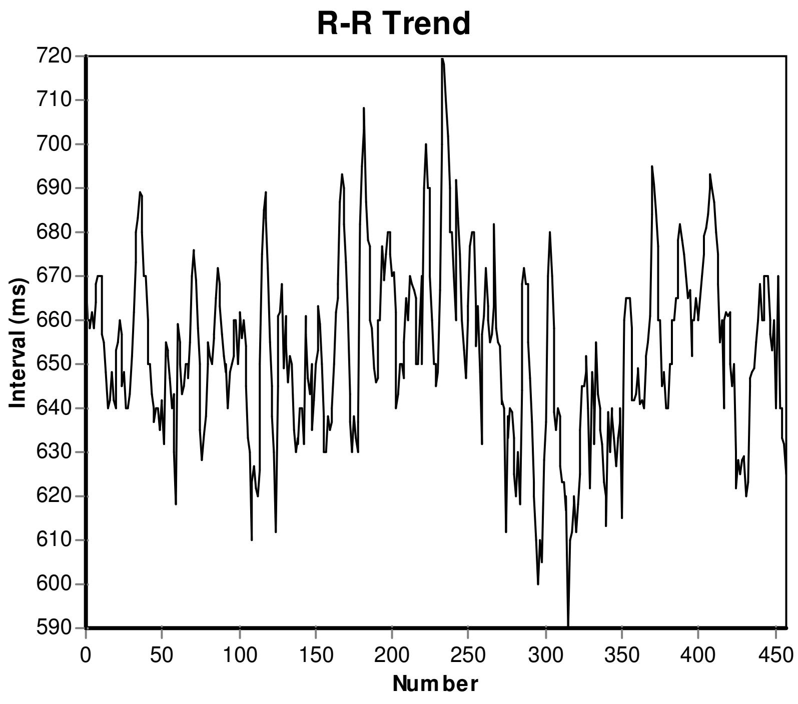


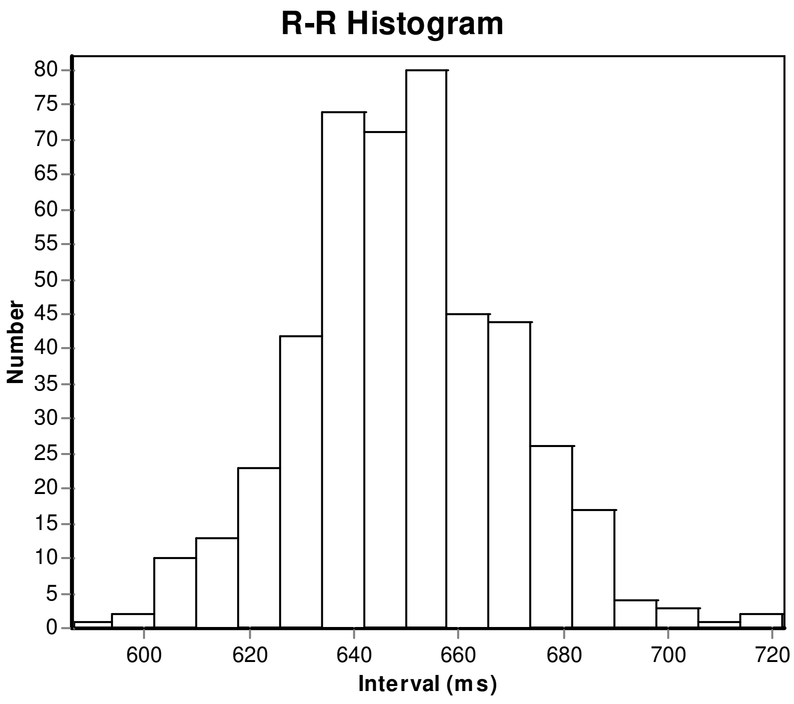

# Cardio Control NV. User: Anton

## Heart Rate Variability: Time Domain Analysis

| | | | |
|---|---|---|---|
| **Name:** | 014, 014 014 | **Birthdate:** | 13/06/1972 |
| **Number:** | 014 | **Recorded:** | 03/05/2018 17:07:49 |
| **Gender:** | Male | | |

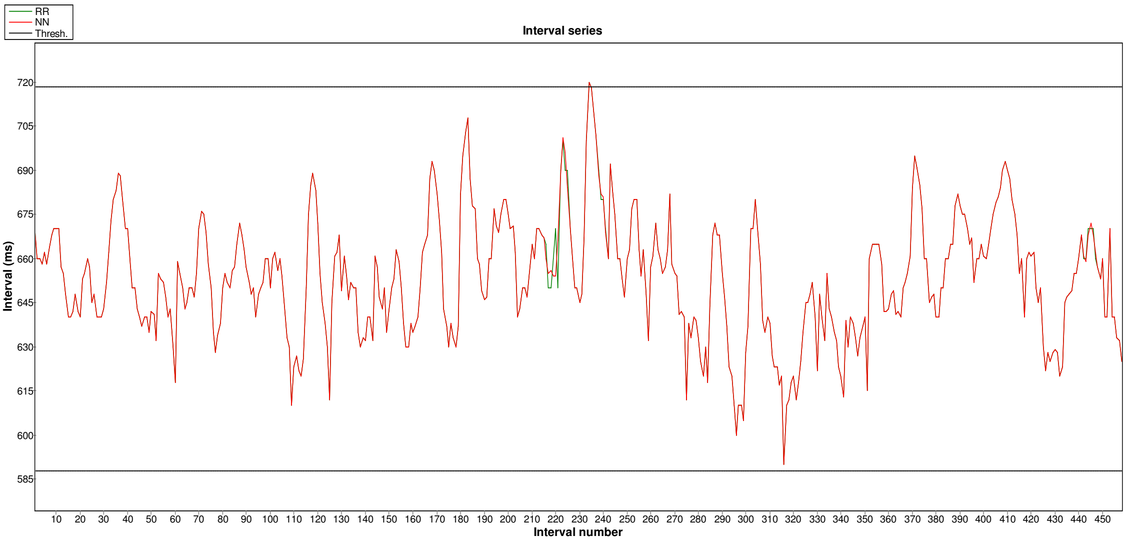


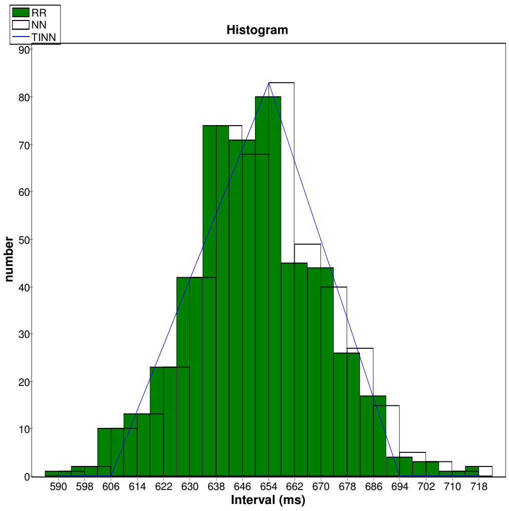


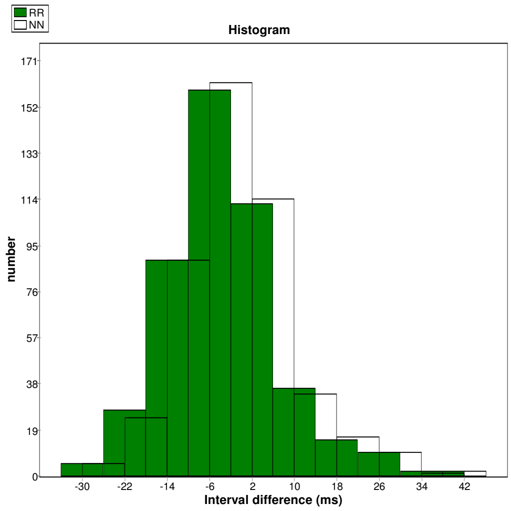


Binsize (ms) = 8

| **HRV parameters** | **NN** | **RR** |
|---|---|---|
| SDNN (ms) | 20 | 20 |
| Triangular Interpolation (ms) | 88 | 96 |
| Triangular Index | 5.52 | 5.72 |

| **HRV parameters** | **NN** | **RR** |
|---|---|---|
| SDSD (ms) | 11 | 11 |
| RMSSD (ms) | 11 | 11 |
| NN50 | 0 | 0 |
| NN50(1) | 0 | 0 |
| NN50(2) | 0 | 0 |
| pNN50 | 0.00 | 0.00 |
| pNN50(1) | 0.00 | 0.00 |
| pNN50(2) | 0.00 | 0.00 |
| Logarithmic Index | 0.96 | 0.92 |
| SD(Logarithmic Index) | 0.07 | 0.09 |

| **Interval statistics** | **NN** | **RR** |
|---|---|---|
| Number | 458 | 458 |
| Minimum (ms) | 590 | 590 |
| Maximum (ms) | 720 | 720 |
| Range (ms) | 130 | 130 |
| Avg (ms) | 653 | 653 |
| SD (ms) | 20 | 20 |
| AvgDev (ms) | 16 | 16 |
| p5 (ms) | 620 | 620 |
| p50 (ms) | 653 | 652 |
| p95 (ms) | 688 | 689 |
| Skewness | 0.15 | 0.15 |
| Kurtosis | 3.24 | 3.22 |

| **Interval statistics** | **NN** | **RR** |
|---|---|---|
| Number | 457 | 457 |
| Minimum (ms) | -30 | -30 |
| Maximum (ms) | 45 | 45 |
| Range (ms) | 75 | 75 |
| Avg (ms) | -0 | -0 |
| SD (ms) | 11 | 11 |
| AvgDev (ms) | 8 | 8 |
| p5 (ms) | -15 | -16 |
| p50 (ms) | 0 | 0 |
| p95 (ms) | 23 | 23 |
| Skewness | 0.82 | 0.85 |
| Kurtosis | 5.28 | 5.35 |

Cardio Control NV. User: Anton

## Heart Rate Variability: Frequency Domain Analysis

| | | | |
|---|---|---|---|
| **Name:** | 014, 014 014 | **Birthdate:** | 13/06/1972 |
| **Number:** | 014 | **Recorded:** | 03/05/2018 17:07:49 |
| **Gender:** | Male | | |

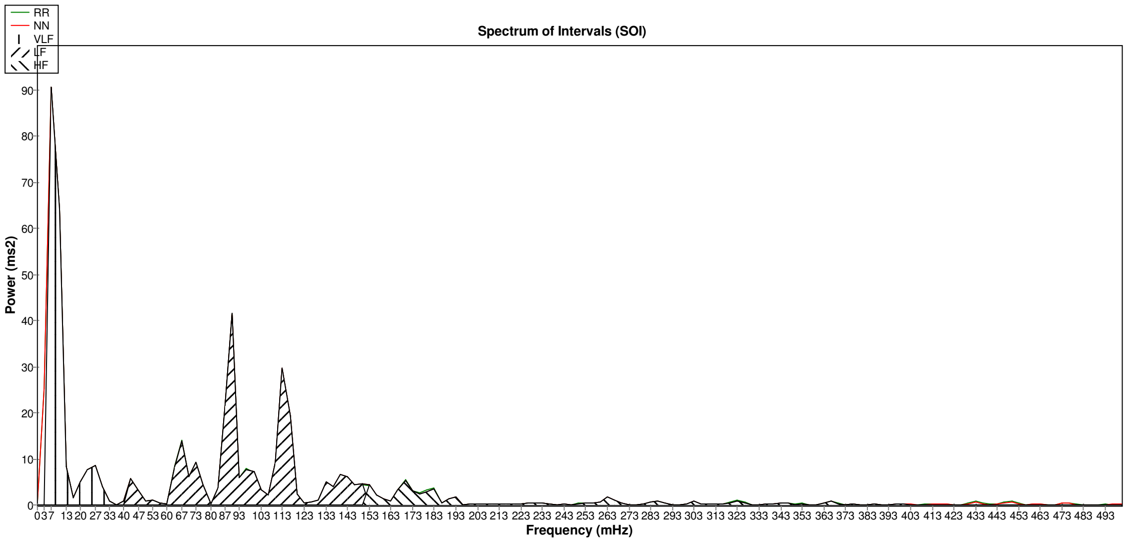


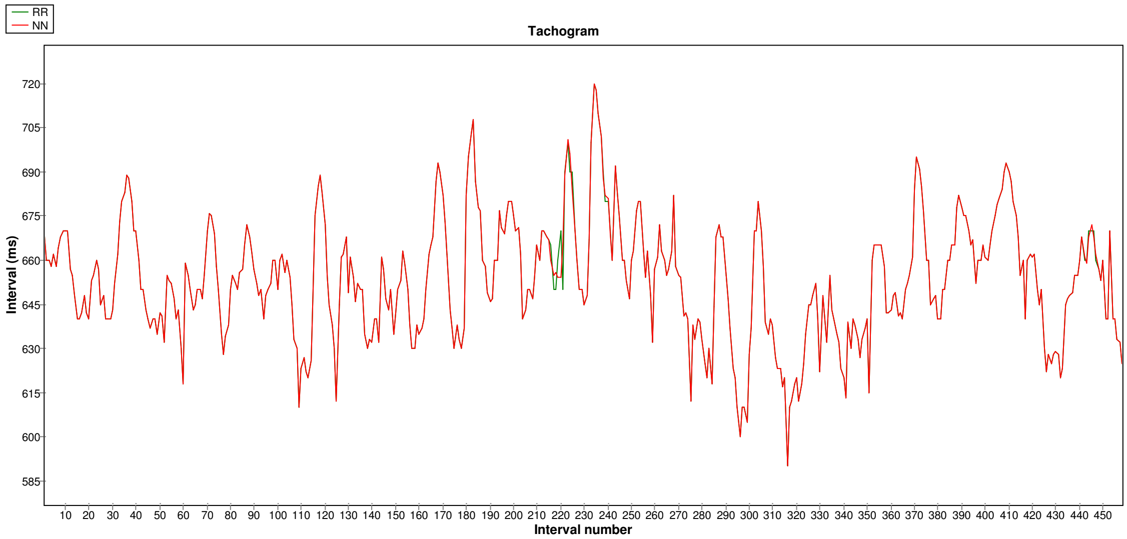


| **HRV parameters** | **NN** | **RR** |
|---|---|---|
| TP (ms2) | 484 | 481 |
| VLF (ms2) | 192 | 192 |
| LF (ms2) | 240 | 239 |
| HF (ms2) | 52 | 51 |
| LF/HF | 4.66 | 4.69 |
| LF normalized | 82.33 | 82.42 |
| HF normalized | 17.67 | 17.58 |
| VLF peak (mHz) | 7 | 7 |
| LF peak (mHz) | 90 | 90 |
| HF peak (mHz) | 170 | 170 |

| **HRV spectral settings** | |
|---|---|
| Spectrum of Intervals (SOI) | |
| Frequency resolution (mHz) | 3 |
| VLF lower boundary (mHz) | 3 |
| VLF upper boundary (mHz) | 40 |
| LF upper boundary (mHz) | 150 |
| HF upper boundary (mHz) | 400 |
| Smoothing factor | 1 |
| Tapering | Hann |
| Fourier transform | DFT |
| Sample frequency (Hz) | 1.53 |
| Interval correction | Annotation |
| Interval threshold (%) | 10 |
