# Supplementary material for: The effects of a 3-day mountain bike cycling race on the autonomic nervous system (ANS) and heart rate variability in amateur cyclists: a prospective quantitative research design
Source: BMC Sports Sci Med Rehabil. 2023 Jan 2;15:2. doi: 10.1186/s13102-022-00614-y (PMC9808932; doi:10.1186/s13102-022-00614-y)
Supplement: Supplementary file 1 — Additional file 1. Individual data of Participants. [file 13102_2022_614_MOESM1_ESM.zip › Individual data of Participants/HRV Data/013/ECG_013_20180504132543_.PDF]

Anton Swart Biokinetic Rehabilitation Practice

Name:	014 014 014	Recorded:	04/05/2018 13:25:43	UNCONFIRMED INTERPRETATION - MD SHOULD REVIEW
Number:	014	Recorded by:	Mr. Anton Swart	
Gender:	Male	Referring physician:		
Birthdate:	13/06/1972 45 years	Ordering physician:		
		Attending physician:		
P / PQ:	108 ms / 158 ms	Location:	Anton Swart Biokinetic Rehabilitation Practi	
QRS:	92 ms	Comment:		
QT / QTc / QTd:	349 ms / 414 ms / -			
P/QRS/T axis:	74° / 77° / 62°			
Heartrate:	97 bpm			

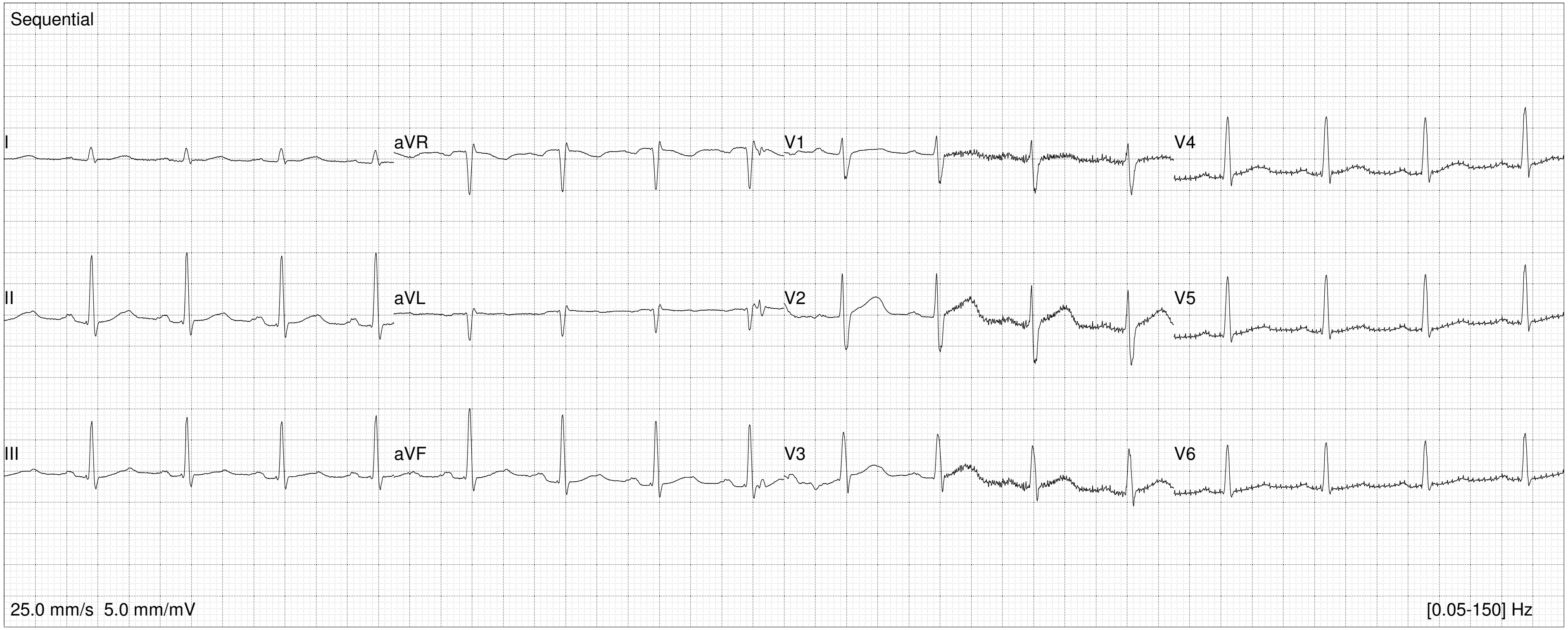

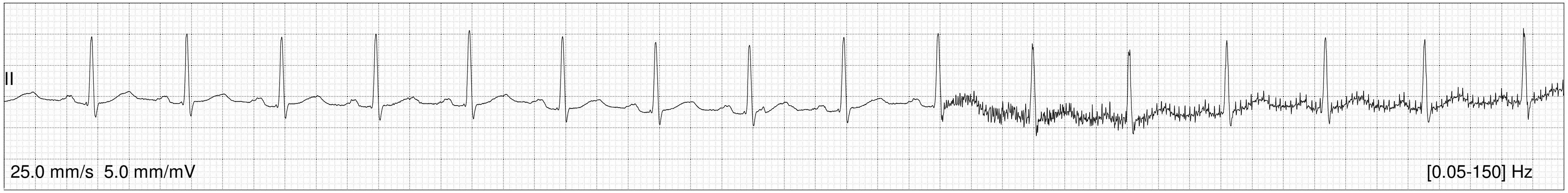

Anton Swart Biokinetic Rehabilitation Practice

Name:	014 014 014	Recorded:	04/05/2018 13:25:43	UNCONFIRMED INTERPRETATION - MD SHOULD REVIEW
Number:	014	Recorded by:	Mr. Anton Swart	
Gender:	Male	Referring physician:		
Birthdate:	13/06/1972 45 years	Location:	Anton Swart Biokinetic Rehabilitation Practice	
		Ordering physician:		
		Attending physician:		
		Comment:		

P / PQ:	108 ms / 158 ms
QRS:	92 ms
QT / QTc / QTd:	349 ms / 414 ms / -
P/QRS/T axis:	74° / 77° / 62°
Heartrate:	97 bpm

Beats

Total:	483
Normal:	483
Other:	0

RR

Minimum	590 ms
Maximum	650 ms
Mean:	619 ms
SD:	12 ms

R-R Trend

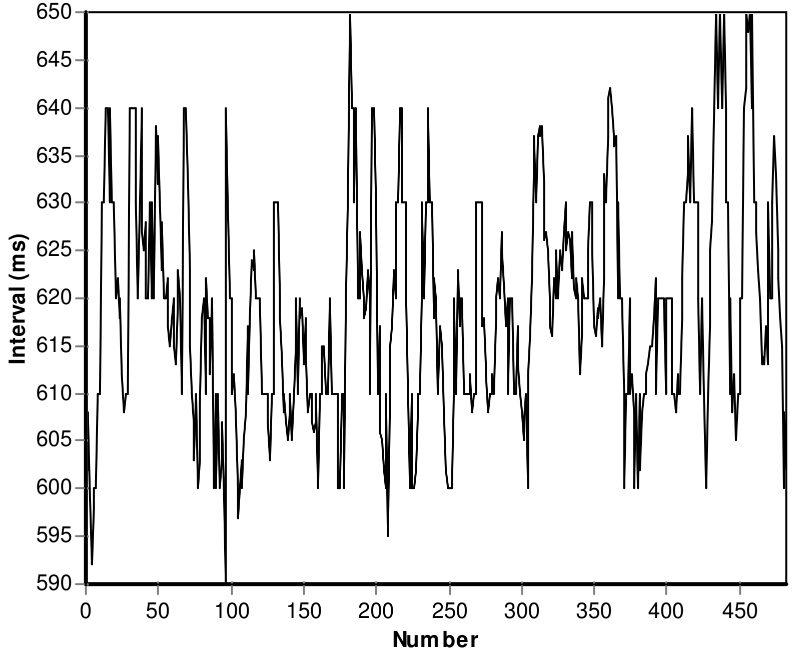

R-R Histogram

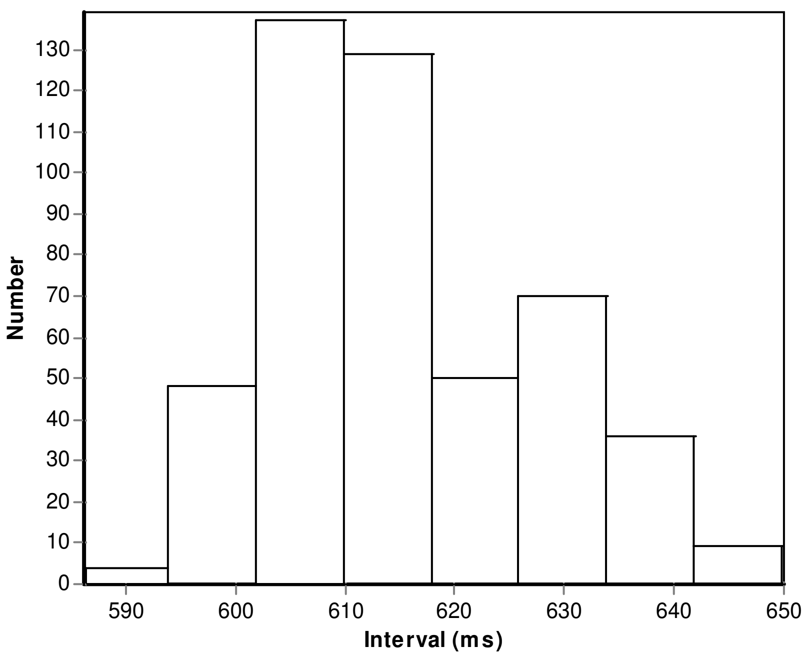

Cardio Control NV. User: Anton

Heart Rate Variability: Time Domain Analysis

**Name:**	014, 014 014	**Birthdate:**	13/06/1972
**Number:**	014	**Recorded:**	04/05/2018 13:25:43
**Gender:**	Male		

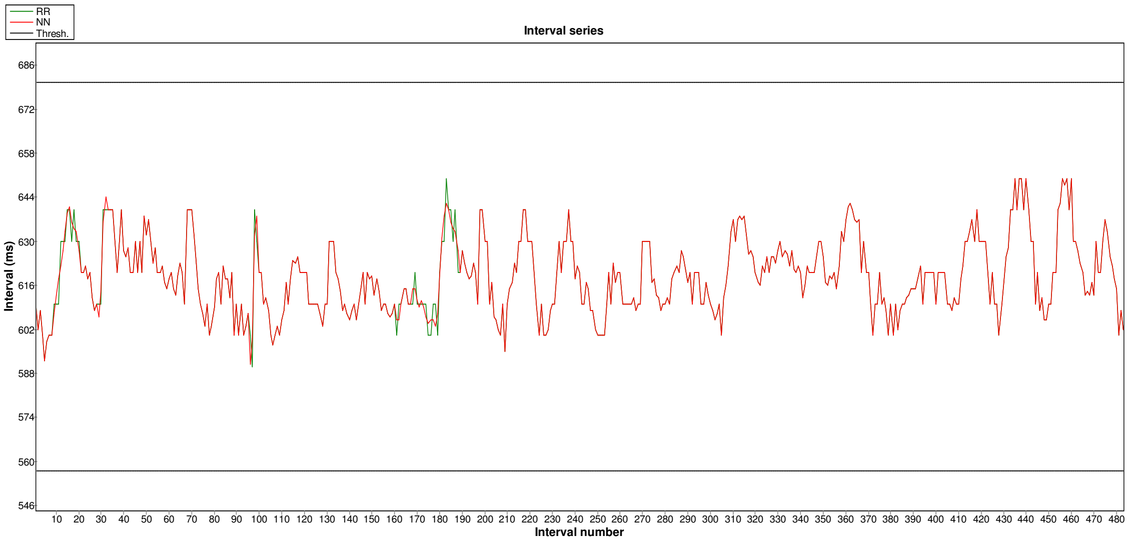

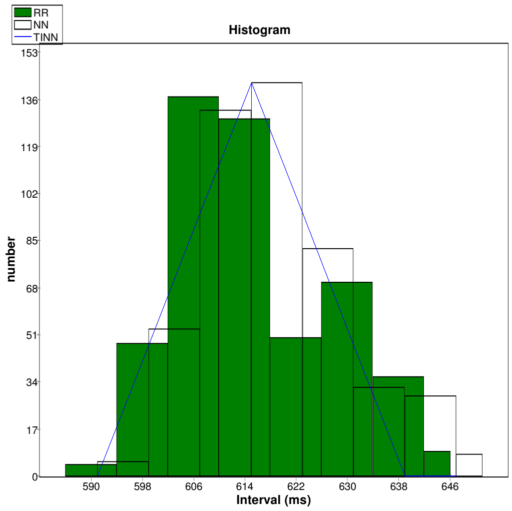

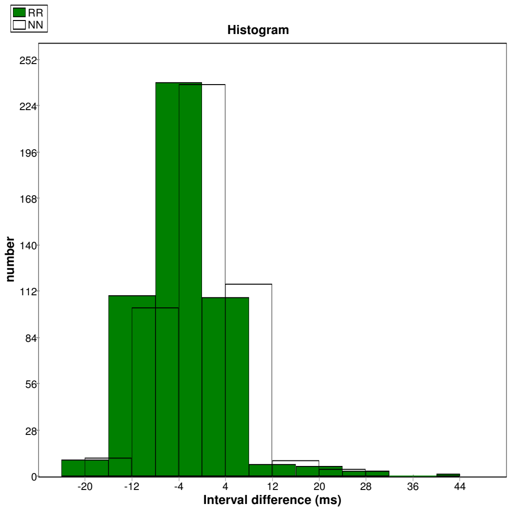

Binsize (ms) = 8

**HRV parameters**	**NN**	**RR**
SDNN (ms)	12	12
Triangular Interpolation (ms)	48	56
Triangular Index	3.40	3.53

**HRV parameters**	**NN**	**RR**
SDSD (ms)	7	8
RMSSD (ms)	7	8
NN50	0	0
NN50(1)	0	0
NN50(2)	0	0
pNN50	0.00	0.00
pNN50(1)	0.00	0.00
pNN50(2)	0.00	0.00
Logarithmic Index	1.58	1.54
SD(Logarithmic Index)	0.15	0.12

**Interval statistics**	**NN**	**RR**
Number	483	483
Minimum (ms)	591	590
Maximum (ms)	650	650
Range (ms)	59	60
Avg (ms)	619	619
SD (ms)	12	12
AvgDev (ms)	9	9
p5 (ms)	600	600
p50 (ms)	619	619
p95 (ms)	640	640
Skewness	0.41	0.42
Kurtosis	2.69	2.74

**Interval statistics**	**NN**	**RR**
Number	482	482
Minimum (ms)	-20	-20
Maximum (ms)	33	50
Range (ms)	53	70
Avg (ms)	-0	-0
SD (ms)	7	8
AvgDev (ms)	5	5
p5 (ms)	-10	-10
p50 (ms)	0	0
p95 (ms)	10	10
Skewness	0.45	0.93
Kurtosis	4.87	7.44

Cardio Control NV. User: Anton

Heart Rate Variability: Frequency Domain Analysis

**Name:**	014, 014 014	**Birthdate:**	13/06/1972
**Number:**	014	**Recorded:**	04/05/2018 13:25:43
**Gender:**	Male		

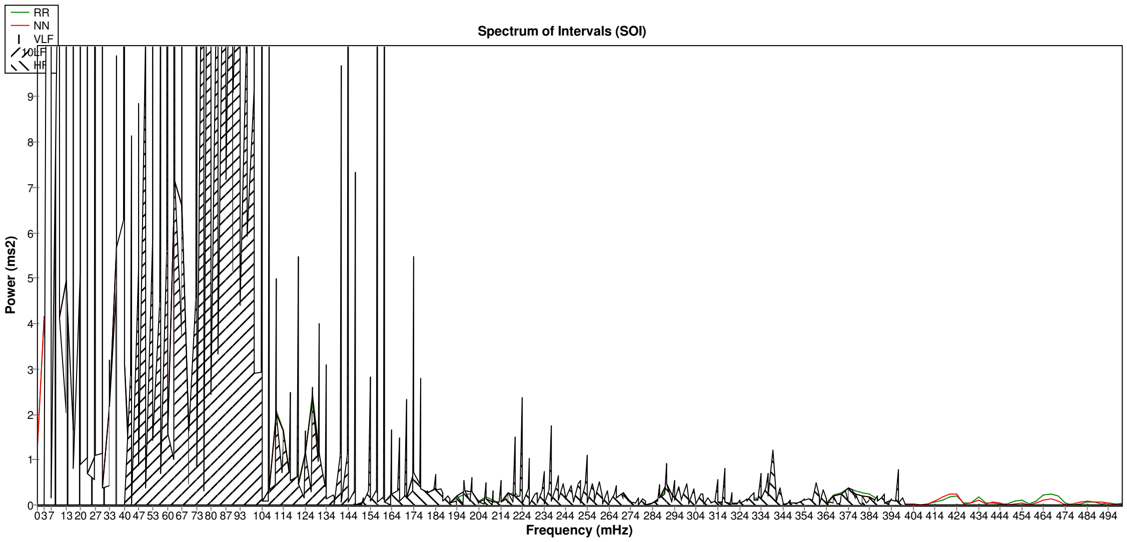

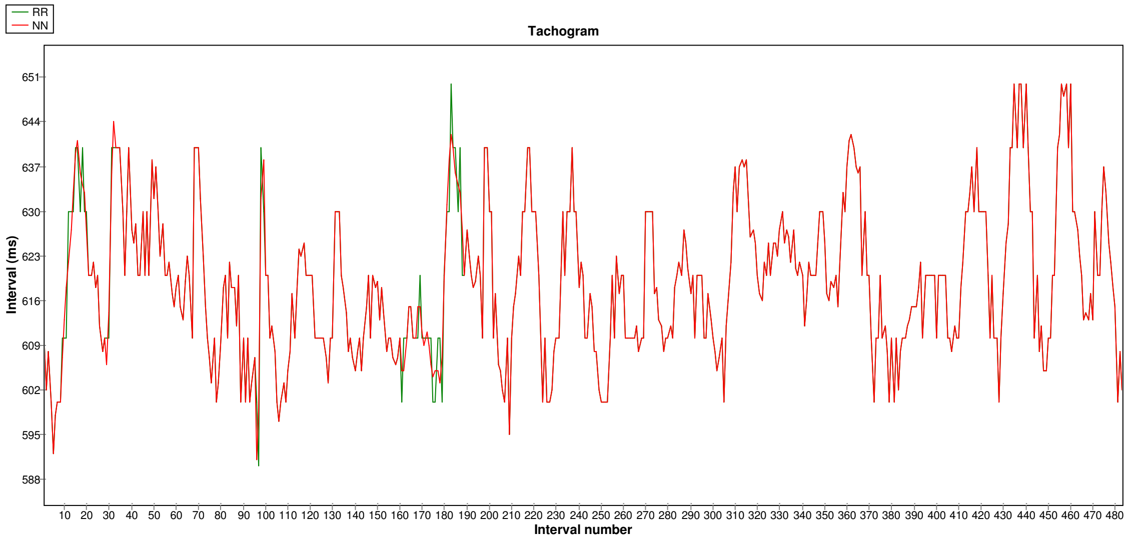

HRV parameters	NN	RR
TP (ms2)	100	101
VLF (ms2)	24	24
LF (ms2)	67	67
HF (ms2)	9	10
LF/HF	7.10	7.01
LF normalized	87.65	87.51
HF normalized	12.35	12.49
VLF peak (mHz)	37	37
LF peak (mHz)	90	90
HF peak (mHz)	174	174

HRV spectral settings	
Spectrum of Intervals (SOI)	
Frequency resolution (mHz)	3
VLF lower boundary (mHz)	3
VLF upper boundary (mHz)	40
LF upper boundary (mHz)	150
HF upper boundary (mHz)	400
Smoothing factor	1
Tapering	Hann
Fourier transform	DFT
Sample frequency (Hz)	1.62
Interval correction	Annotation
Interval threshold (%)	10
