# Supplementary material for: The effects of a 3-day mountain bike cycling race on the autonomic nervous system (ANS) and heart rate variability in amateur cyclists: a prospective quantitative research design
Source: BMC Sports Sci Med Rehabil. 2023 Jan 2;15:2. doi: 10.1186/s13102-022-00614-y (PMC9808932; doi:10.1186/s13102-022-00614-y)
Supplement: Supplementary file 1 — Additional file 1. Individual data of Participants. [file 13102_2022_614_MOESM1_ESM.zip › Individual data of Participants/HRV Data/013/ECG_013_20180505130615_.PDF]

Anton Swart Biokinetic Rehabilitation Practice

Name: 014 014 014
Number: 014
Gender: Male
Birthdate: 13/06/1972 45 years

P / PQ: 113 ms / 172 ms
QRS: 86 ms
QT / QTc / QTd: 350 ms / 406 ms / -
P/QRS/T axis: 77° / 77° / 64°
Heartrate: 92 bpm

Recorded: 05/05/2018 13:06:15
Recorded by: Mr. Anton Swart
Referring physician:
Ordering physician:
Attending physician:
Location: Anton Swart Biokinetic Rehabilitation Practi
Comment:

UNCONFIRMED INTERPRETATION - MD SHOULD REVIEW

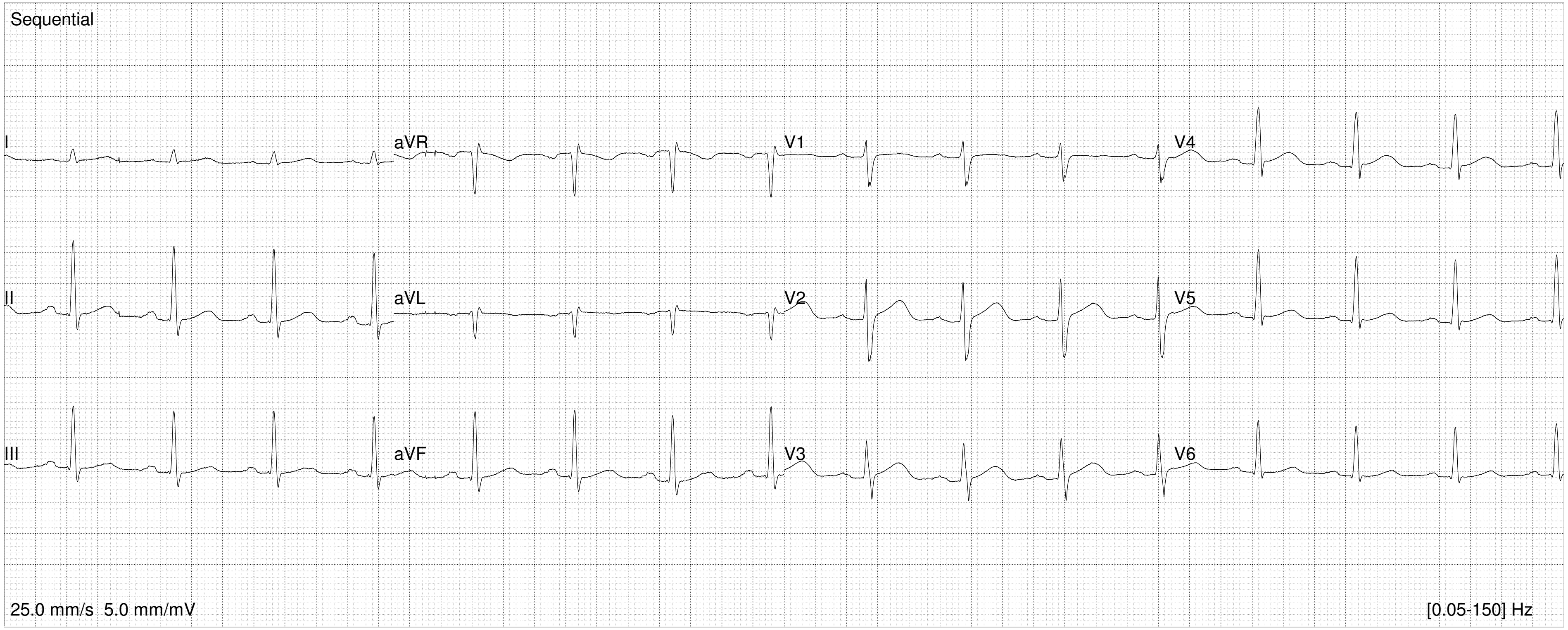

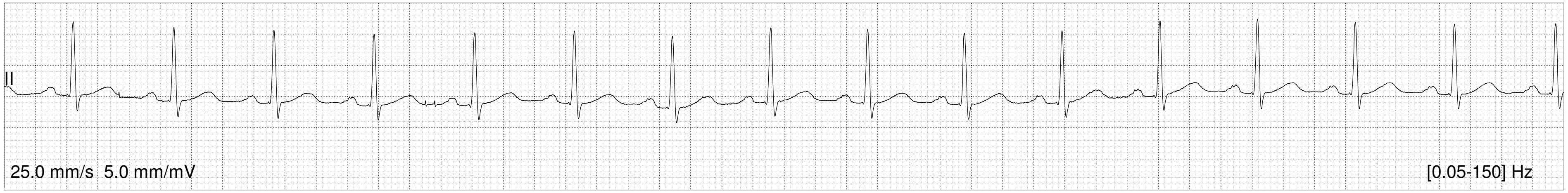

Anton Swart Biokinetic Rehabilitation Practice

Name:	014 014 014	Recorded:	05/05/2018 13:06:15	UNCONFIRMED INTERPRETATION - MD SHOULD REVIEW
Number:	014	Recorded by:	Mr. Anton Swart	
Gender:	Male	Referring physician:		
Birthdate:	13/06/1972 45 years	Location:	Anton Swart Biokinetic Rehabilitation Practice	
		Ordering physician:		
		Attending physician:		
		Comment:		

P / PQ:	113 ms / 172 ms
QRS:	86 ms
QT / QTc / QTd:	350 ms / 406 ms / -
P/QRS/T axis:	77° / 77° / 64°
Heartrate:	92 bpm

Beats

Total:	462
Normal:	462
Other:	0

RR

Minimum	600 ms
Maximum	685 ms
Mean:	647 ms
SD:	12 ms

R-R Trend

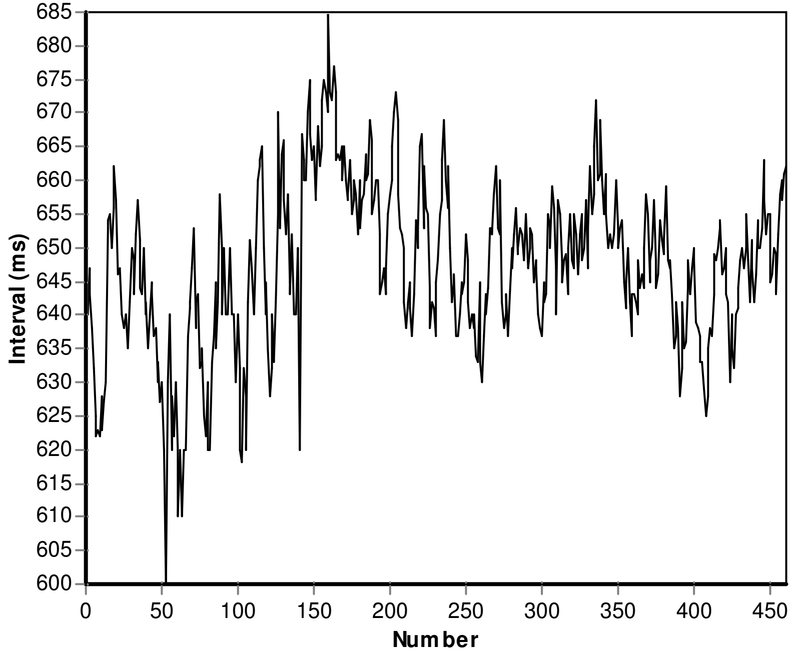

R-R Histogram

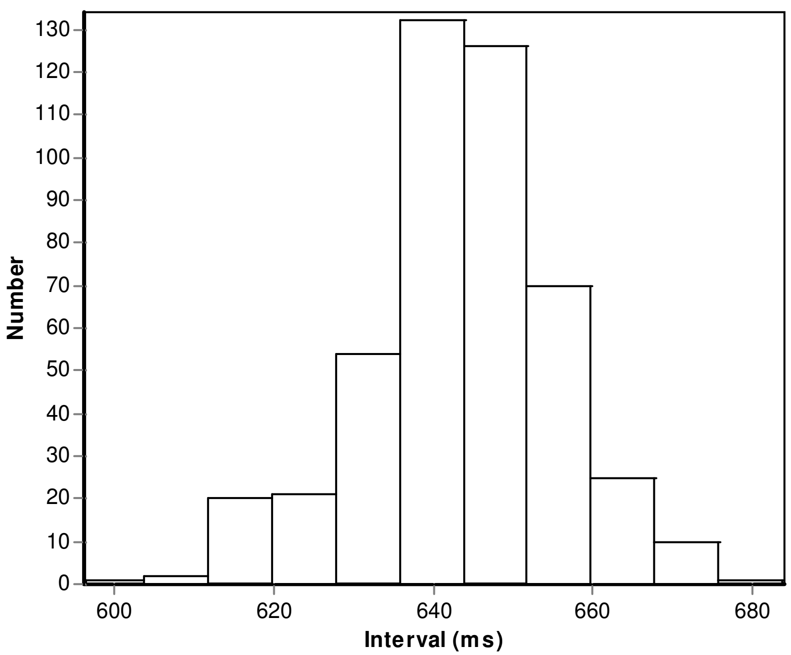

Cardio Control NV. User: Anton

Heart Rate Variability: Time Domain Analysis

**Name:**	014, 014 014	**Birthdate:**	13/06/1972
**Number:**	014	**Recorded:**	05/05/2018 13:06:15
**Gender:**	Male		

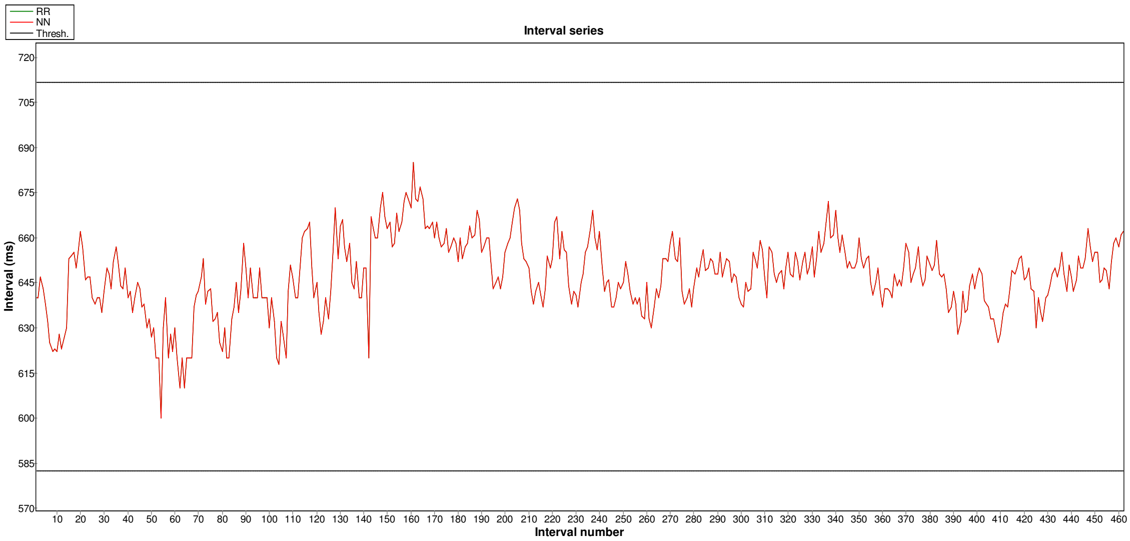

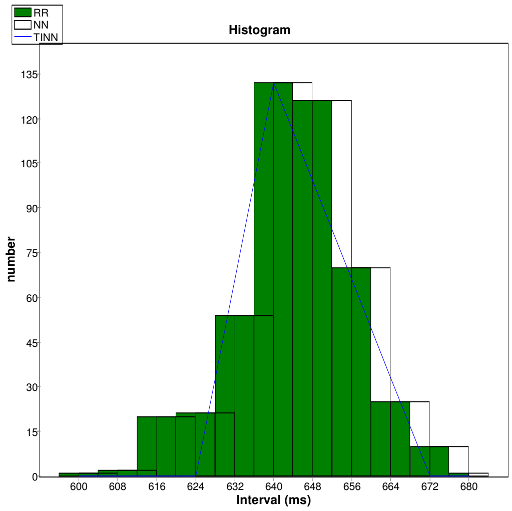

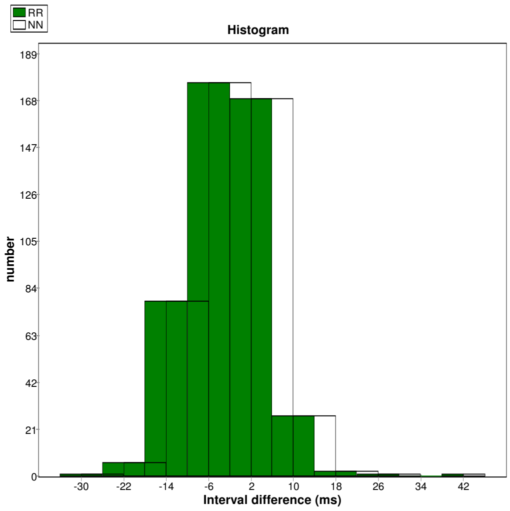

Binsize (ms) = 8

**HRV parameters**	**NN**	**RR**
SDNN (ms)	12	12
Triangular Interpolation (ms)	48	48
Triangular Index	3.50	3.50

**HRV parameters**	**NN**	**RR**
SDSD (ms)	7	7
RMSSD (ms)	7	7
NN50	0	0
NN50(1)	0	0
NN50(2)	0	0
pNN50	0.00	0.00
pNN50(1)	0.00	0.00
pNN50(2)	0.00	0.00
Logarithmic Index	1.57	1.57
SD(Logarithmic Index)	0.17	0.17

**Interval statistics**	**NN**	**RR**
Number	462	462
Minimum (ms)	600	600
Maximum (ms)	685	685
Range (ms)	85	85
Avg (ms)	647	647
SD (ms)	12	12
AvgDev (ms)	9	9
p5 (ms)	624	624
p50 (ms)	648	648
p95 (ms)	667	667
Skewness	-0.28	-0.28
Kurtosis	3.49	3.49

**Interval statistics**	**NN**	**RR**
Number	461	461
Minimum (ms)	-30	-30
Maximum (ms)	47	47
Range (ms)	77	77
Avg (ms)	0	0
SD (ms)	7	7
AvgDev (ms)	6	6
p5 (ms)	-10	-10
p50 (ms)	0	0
p95 (ms)	10	10
Skewness	0.56	0.56
Kurtosis	7.23	7.23

Heart Rate Variability: Frequency Domain Analysis

**Name:**	014, 014 014	**Birthdate:**	13/06/1972
**Number:**	014	**Recorded:**	05/05/2018 13:06:15
**Gender:**	Male		

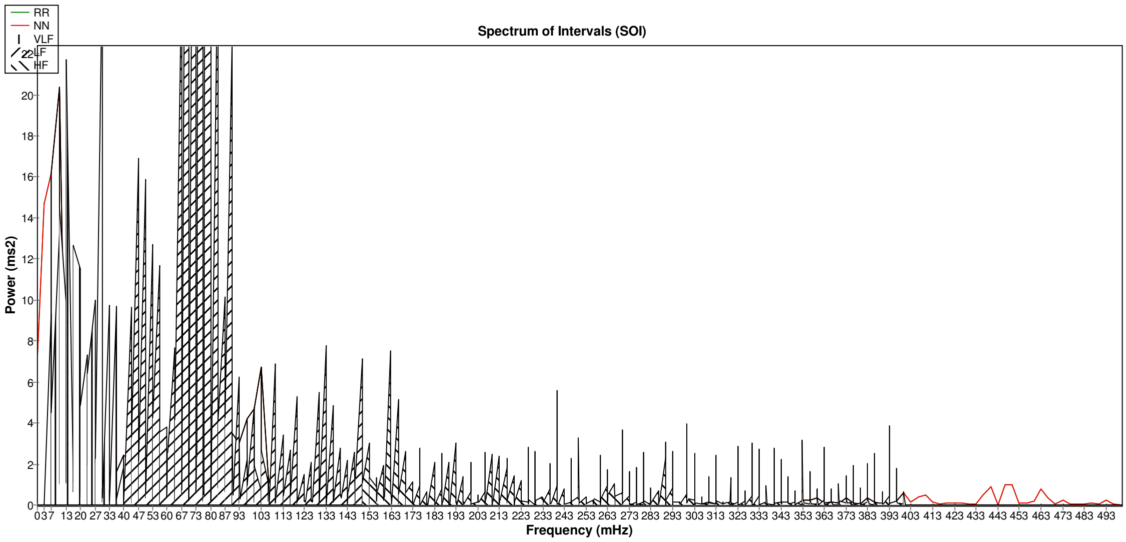

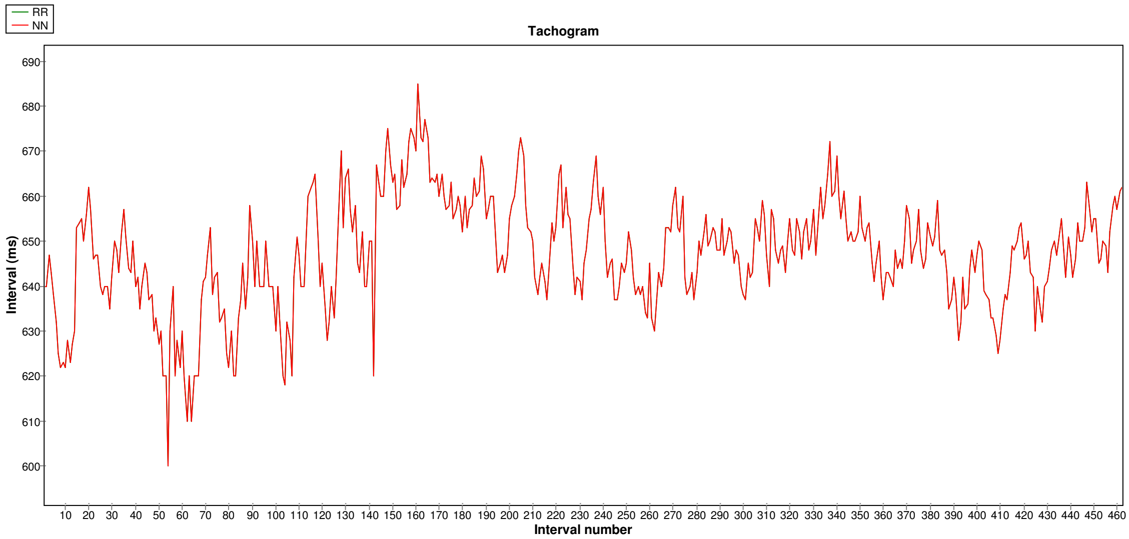

**HRV parameters**	**NN**	**RR**
TP (ms2)	101	101
VLF (ms2)	49	49
LF (ms2)	45	45
HF (ms2)	6	6
LF/HF	7.21	7.21
LF normalized	87.82	87.82
HF normalized	12.18	12.18
VLF peak (mHz)	10	10
LF peak (mHz)	103	103
HF peak (mHz)	400	400

**HRV spectral settings**	
Spectrum of Intervals (SOI)	
Frequency resolution (mHz)	3
VLF lower boundary (mHz)	3
VLF upper boundary (mHz)	40
LF upper boundary (mHz)	150
HF upper boundary (mHz)	400
Smoothing factor	1
Tapering	Hann
Fourier transform	DFT
Sample frequency (Hz)	1.55
Interval correction	Annotation
Interval threshold (%)	10
