# Supplementary material for: The effects of a 3-day mountain bike cycling race on the autonomic nervous system (ANS) and heart rate variability in amateur cyclists: a prospective quantitative research design
Source: BMC Sports Sci Med Rehabil. 2023 Jan 2;15:2. doi: 10.1186/s13102-022-00614-y (PMC9808932; doi:10.1186/s13102-022-00614-y)
Supplement: Supplementary file 1 — Additional file 1. Individual data of Participants. [file 13102_2022_614_MOESM1_ESM.zip › Individual data of Participants/HRV Data/013/ECG_013_20180506084708_.PDF]

Anton Swart Biokinetic Rehabilitation Practice

Name:	014 014 014	Recorded:	06/05/2018 08:47:08	UNCONFIRMED INTERPRETATION - MD SHOULD REVIEW
Number:	014	Recorded by:	Mr. Anton Swart	
Gender:	Male	Referring physician:		
Birthdate:	13/06/1972 45 years	Ordering physician:		
		Attending physician:		
P / PQ:	133 ms / 150 ms	Location:	Anton Swart Biokinetic Rehabilitation Practi	
QRS:	83 ms	Comment:		
QT / QTc / QTd:	408 ms / 413 ms / -			
P/QRS/T axis:	11° / 77° / 53°			
Heartrate:	63 bpm			

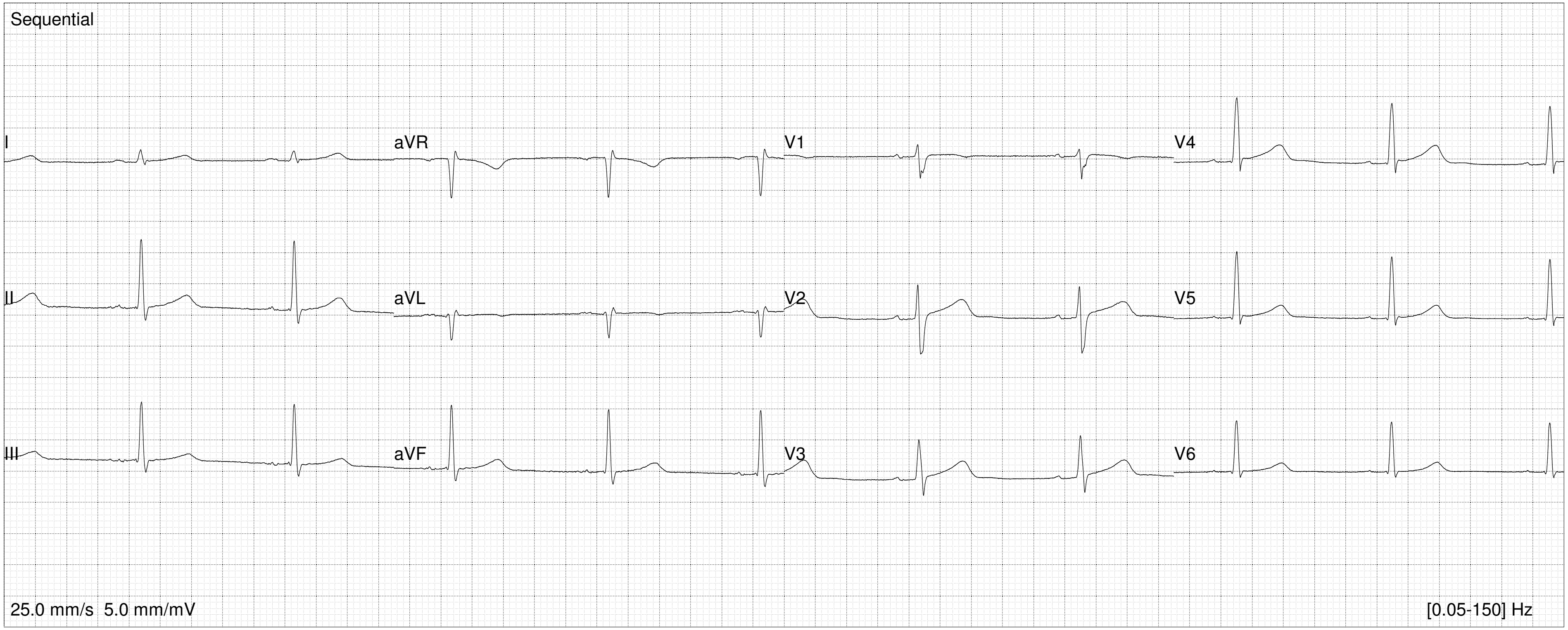

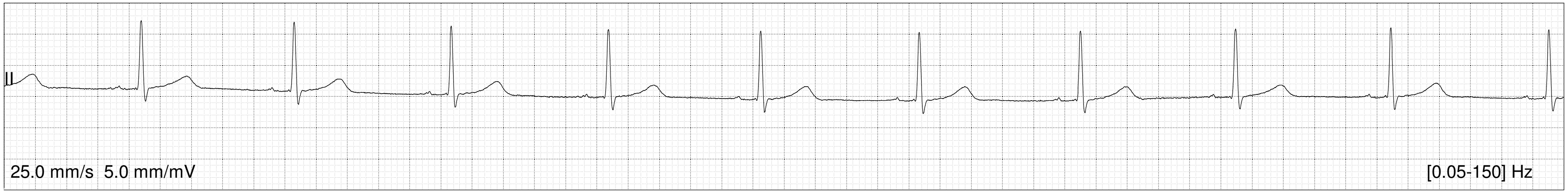

Anton Swart Biokinetic Rehabilitation Practice

Name:	014 014 014	Recorded:	06/05/2018 08:47:08	UNCONFIRMED INTERPRETATION - MD SHOULD REVIEW
Number:	014	Recorded by:	Mr. Anton Swart	
Gender:	Male	Referring physician:		
Birthdate:	13/06/1972 45 years	Location:	Anton Swart Biokinetic Rehabilitation Practice	
		Ordering physician:		
		Attending physician:		
		Comment:		

P / PQ:	133 ms / 150 ms
QRS:	83 ms
QT / QTc / QTd:	408 ms / 413 ms / -
P/QRS/T axis:	11° / 77° / 53°
Heartrate:	63 bpm

Beats

Total:	312
Normal:	312
Other:	0

RR

Minimum	890 ms
Maximum	1040 ms
Mean:	957 ms
SD:	28 ms

R-R Trend

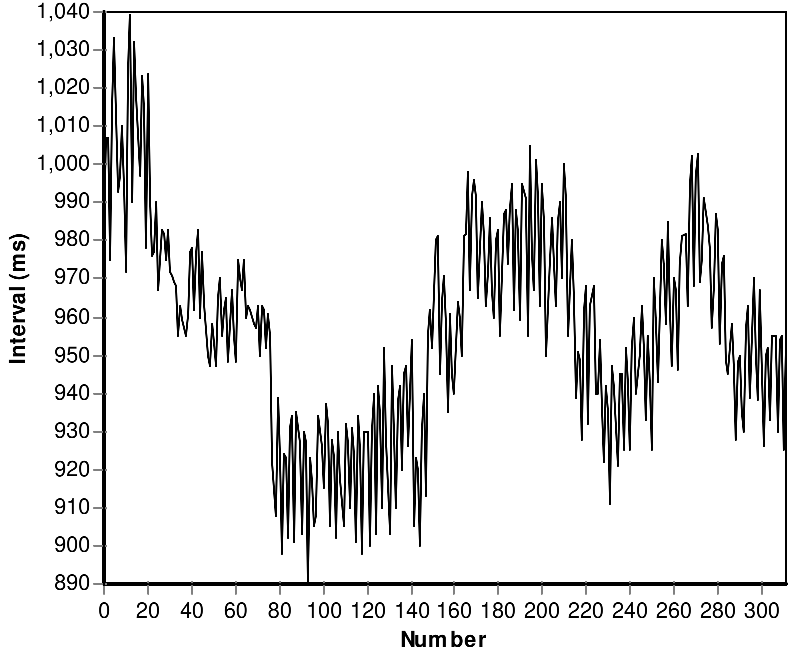

R-R Histogram

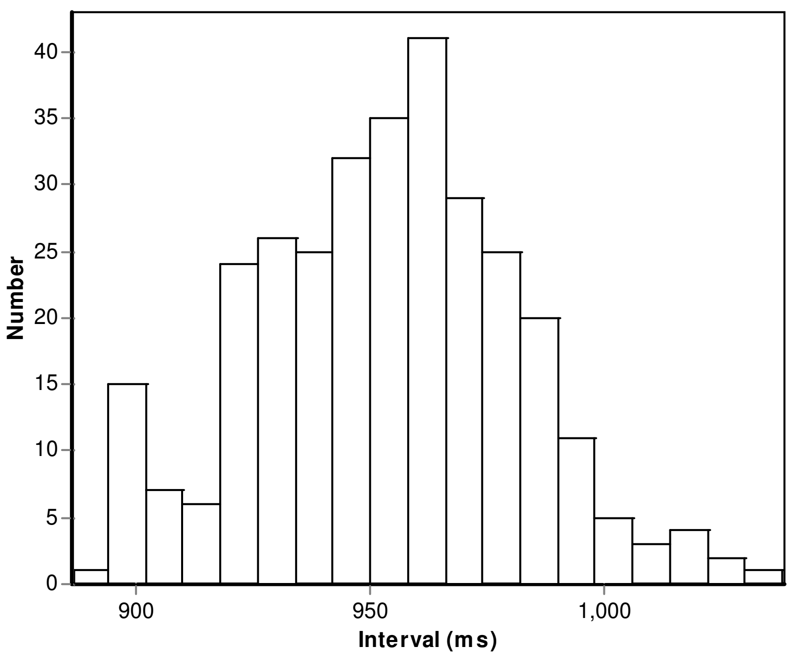

Cardio Control NV. User: Anton

Heart Rate Variability: Time Domain Analysis

**Name:**	014, 014 014	**Birthdate:**	13/06/1972
**Number:**	014	**Recorded:**	06/05/2018 08:47:08
**Gender:**	Male		

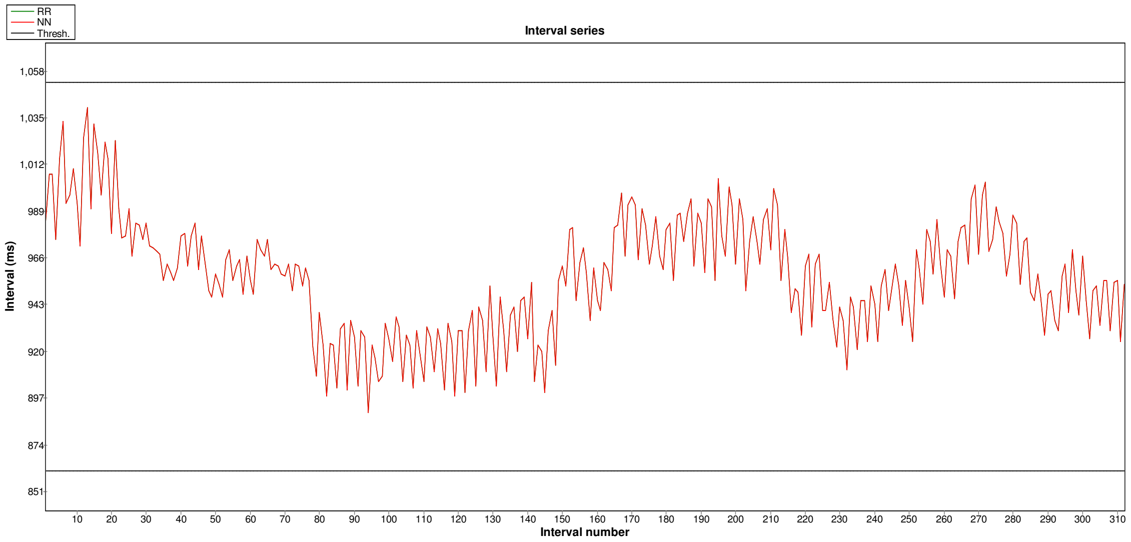

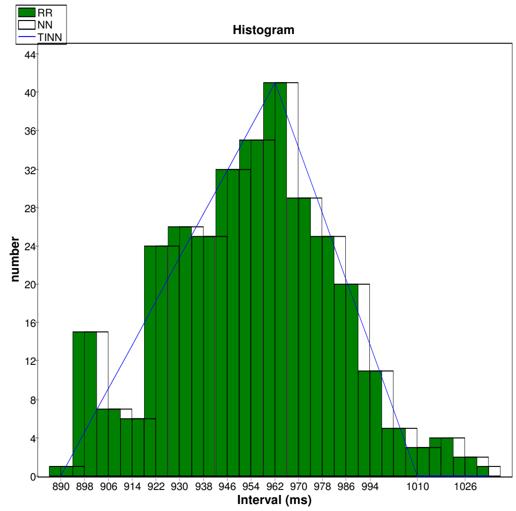

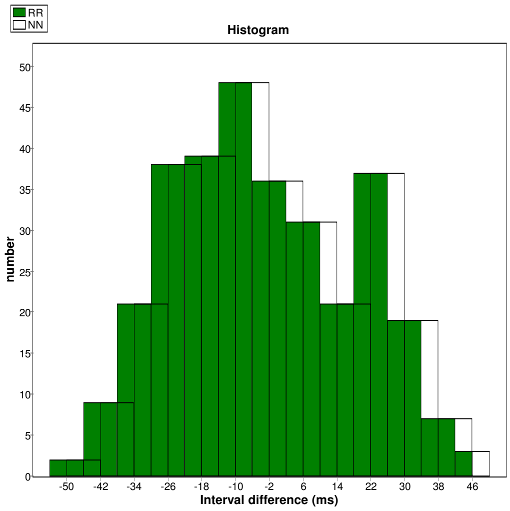

Binsize (ms) = 8

**HRV parameters**	**NN**	**RR**
SDNN (ms)	28	28
Triangular Interpolation (ms)	120	120
Triangular Index	7.61	7.61

**HRV parameters**	**NN**	**RR**
SDSD (ms)	21	21
RMSSD (ms)	21	21
NN50	1	1
NN50(1)	0	0
NN50(2)	1	1
pNN50	0.00	0.00
pNN50(1)	0.00	0.00
pNN50(2)	0.00	0.00
Logarithmic Index	0.50	0.50
SD(Logarithmic Index)	0.10	0.10

**Interval statistics**	**NN**	**RR**
Number	312	312
Minimum (ms)	890	890
Maximum (ms)	1040	1040
Range (ms)	150	150
Avg (ms)	957	957
SD (ms)	28	28
AvgDev (ms)	22	22
p5 (ms)	905	905
p50 (ms)	958	958
p95 (ms)	1002	1002
Skewness	0.08	0.08
Kurtosis	2.88	2.88

**Interval statistics**	**NN**	**RR**
Number	311	311
Minimum (ms)	-50	-50
Maximum (ms)	53	53
Range (ms)	103	103
Avg (ms)	-0	-0
SD (ms)	21	21
AvgDev (ms)	18	18
p5 (ms)	-33	-33
p50 (ms)	-3	-3
p95 (ms)	34	34
Skewness	0.20	0.20
Kurtosis	2.27	2.27

Cardio Control NV. User: Anton

Heart Rate Variability: Frequency Domain Analysis

**Name:**	014, 014 014	**Birthdate:**	13/06/1972
**Number:**	014	**Recorded:**	06/05/2018 08:47:08
**Gender:**	Male		

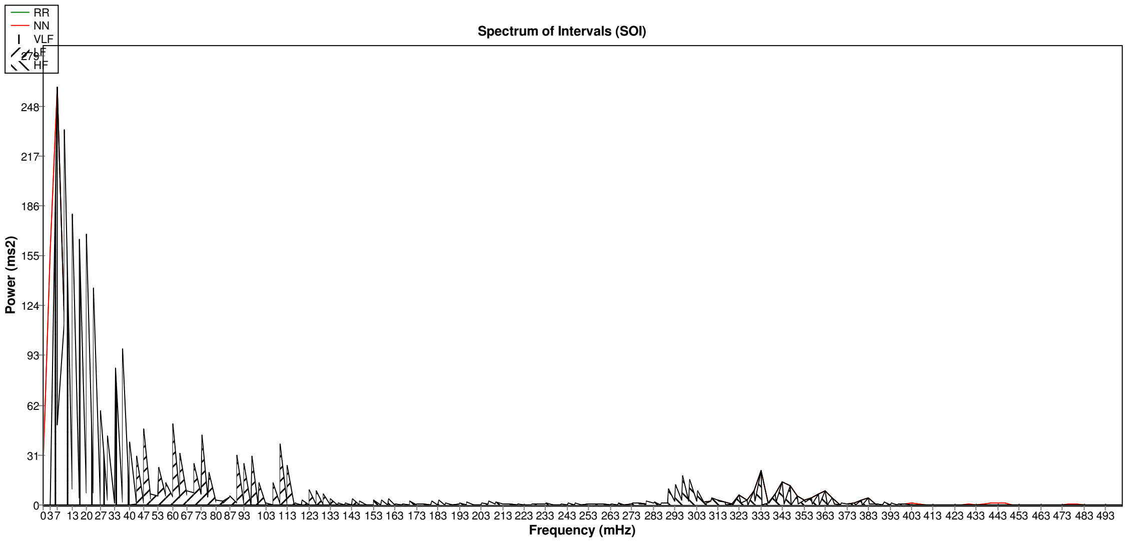

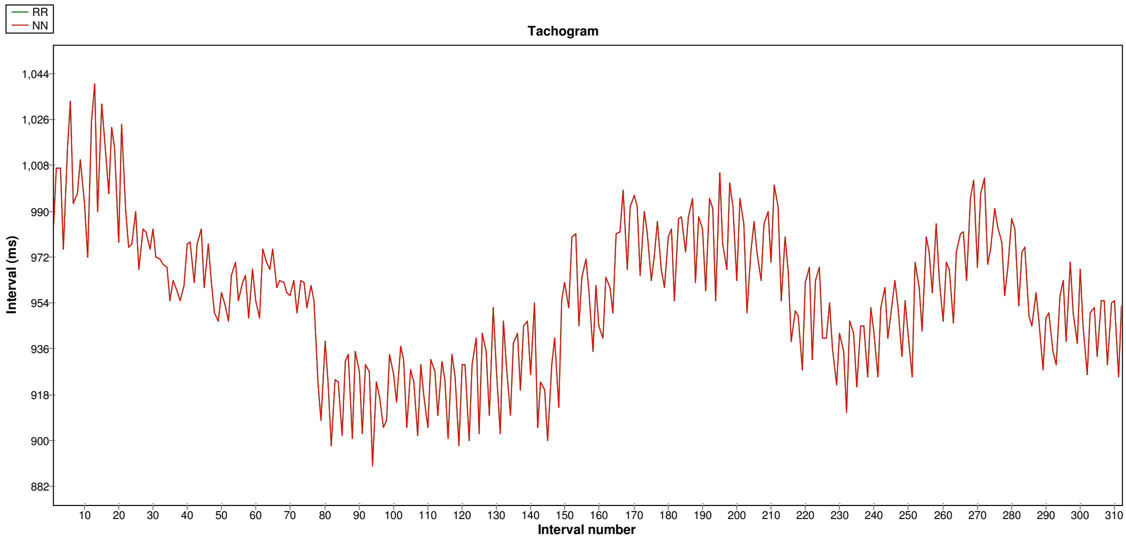

HRV parameters	NN	RR
TP (ms2)	628	628
VLF (ms2)	410	410
LF (ms2)	66	66
HF (ms2)	152	152
LF/HF	0.43	0.43
LF normalized	30.28	30.28
HF normalized	69.72	69.72
VLF peak (mHz)	7	7
LF peak (mHz)	70	70
HF peak (mHz)	333	333

HRV spectral settings	
Spectrum of Intervals (SOI)	
Frequency resolution (mHz)	3
VLF lower boundary (mHz)	3
VLF upper boundary (mHz)	40
LF upper boundary (mHz)	150
HF upper boundary (mHz)	400
Smoothing factor	1
Tapering	Hann
Fourier transform	DFT
Sample frequency (Hz)	1.05
Interval correction	Annotation
Interval threshold (%)	10
