# Supplementary material for: The effects of a 3-day mountain bike cycling race on the autonomic nervous system (ANS) and heart rate variability in amateur cyclists: a prospective quantitative research design
Source: BMC Sports Sci Med Rehabil. 2023 Jan 2;15:2. doi: 10.1186/s13102-022-00614-y (PMC9808932; doi:10.1186/s13102-022-00614-y)
Supplement: Supplementary file 1 — Additional file 1. Individual data of Participants. [file 13102_2022_614_MOESM1_ESM.zip › Individual data of Participants/HRV Data/014/ECG_014_20180501170336_.PDF]

Anton Swart Biokinetic Rehabilitation Practice

Name:	015 015 015	Recorded:	01/05/2018 17:03:36	UNCONFIRMED INTERPRETATION - MD SHOULD REVIEW
Number:	015	Recorded by:	Mr. Anton Swart	
Gender:	Male	Referring physician:		
Birthdate:	26/01/1964 54 years	Ordering physician:		
		Attending physician:		
P / PQ:	132 ms / 185 ms	Location:	Anton Swart Biokinetic Rehabilitation Practi	
QRS:	102 ms	Comment:		
QT / QTc / QTd:	413 ms / 432 ms / -			
P/QRS/T axis:	75° / 68° / 81°			
Heartrate:	71 bpm			

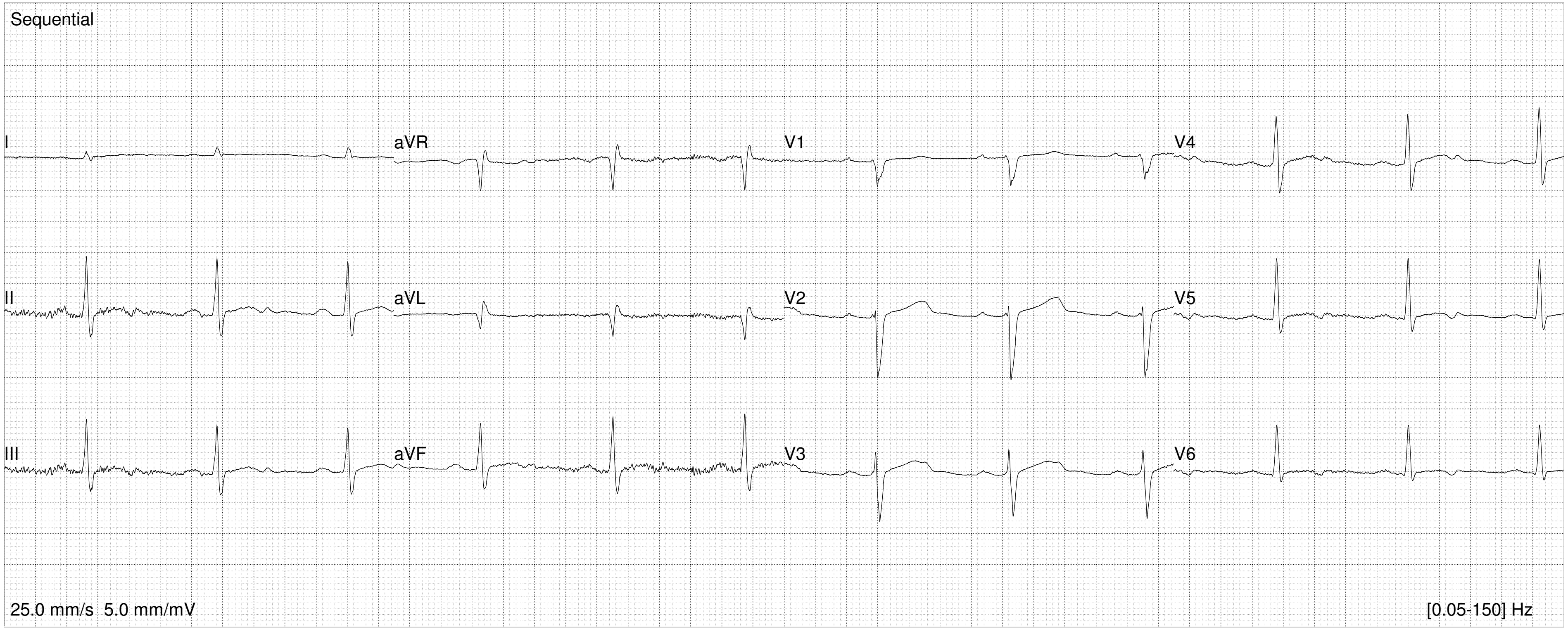

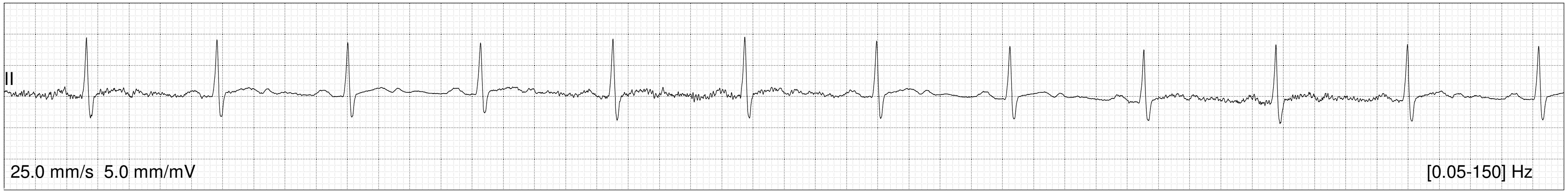

Anton Swart Biokinetic Rehabilitation Practice

Name:	015 015 015	Recorded:	01/05/2018 17:03:36	UNCONFIRMED INTERPRETATION - MD SHOULD REVIEW
Number:	015	Recorded by:	Mr. Anton Swart	
Gender:	Male	Referring physician:		
Birthdate:	26/01/1964 54 years	Location:	Anton Swart Biokinetic Rehabilitation Practice	
		Ordering physician:		
		Attending physician:		
		Comment:		

P / PQ:	132 ms / 185 ms
QRS:	102 ms
QT / QTc / QTd:	413 ms / 432 ms / -
P/QRS/T axis:	75° / 68° / 81°
Heartrate:	71 bpm

Beats

Total:	351
Normal:	351
Other:	0

RR

Minimum	810 ms
Maximum	896 ms
Mean:	852 ms
SD:	16 ms

R-R Trend

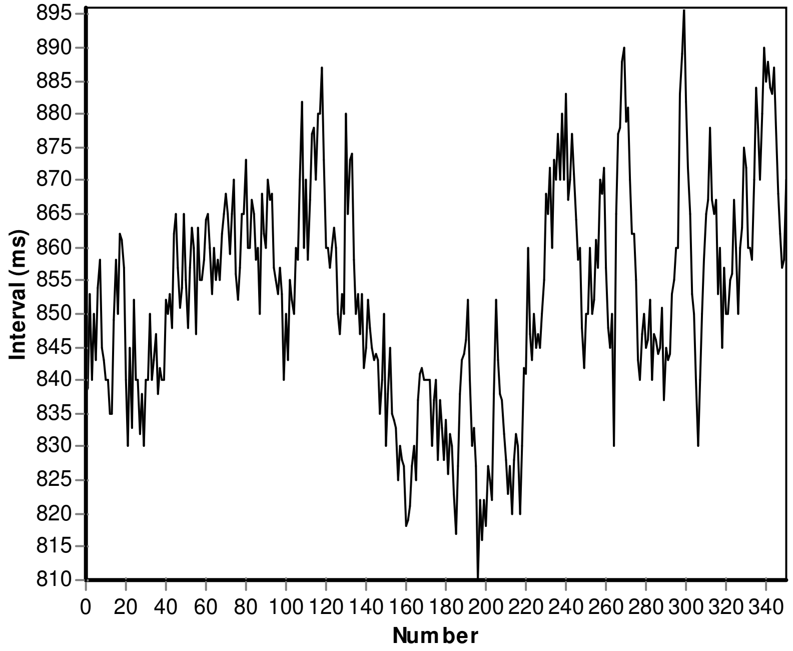

R-R Histogram

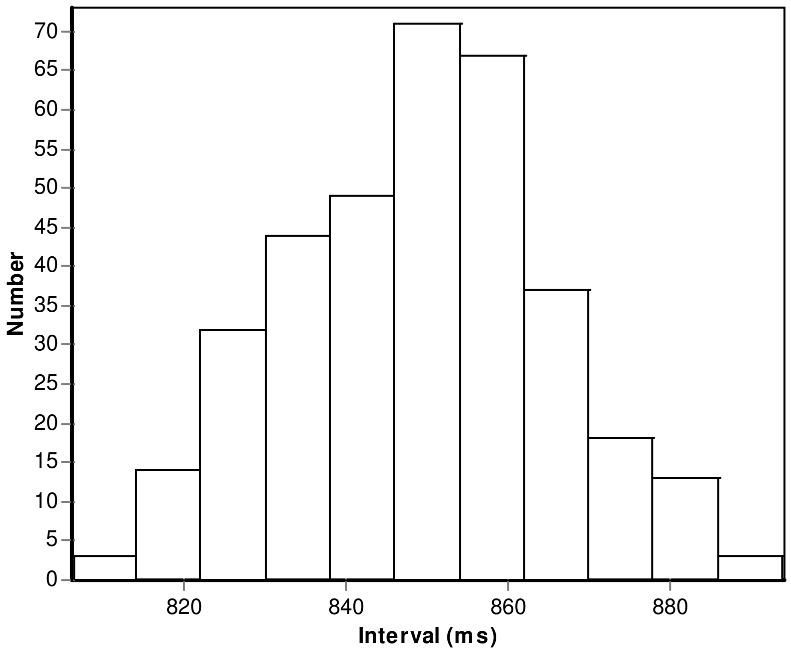

Heart Rate Variability: Time Domain Analysis

**Name:**	015, 015 015	**Birthdate:**	26/01/1964
**Number:**	015	**Recorded:**	01/05/2018 17:03:36
**Gender:**	Male		

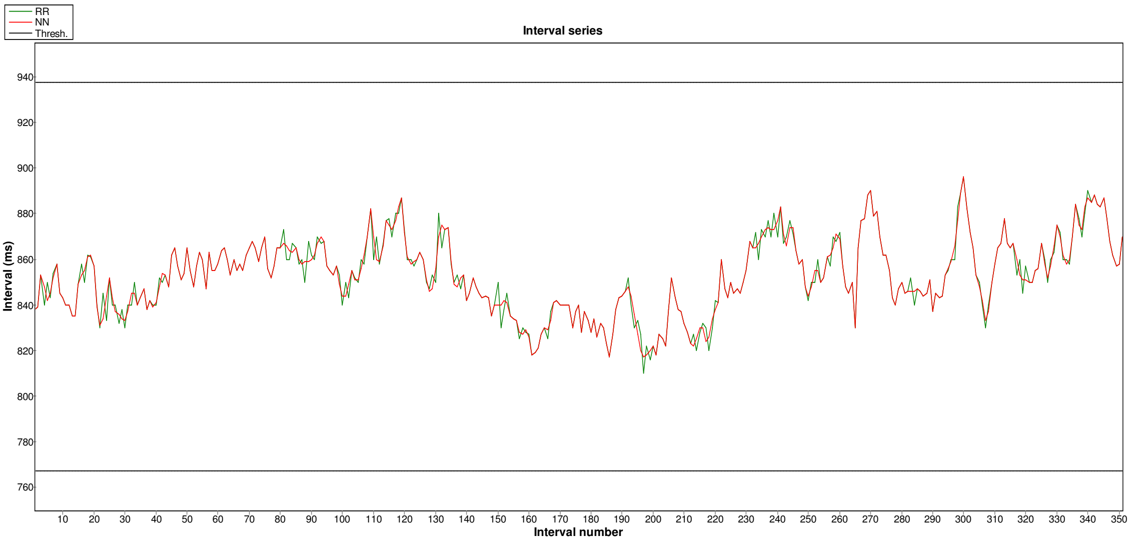

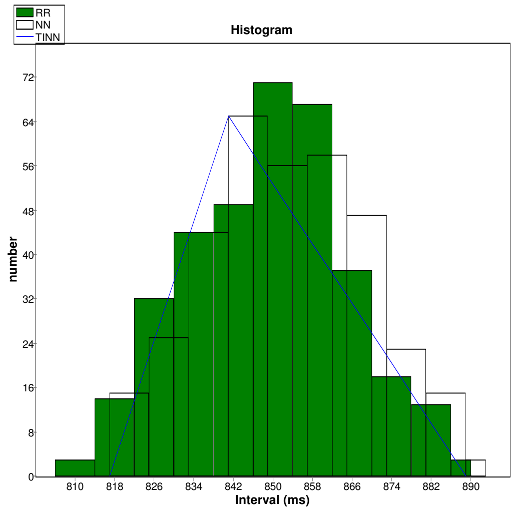

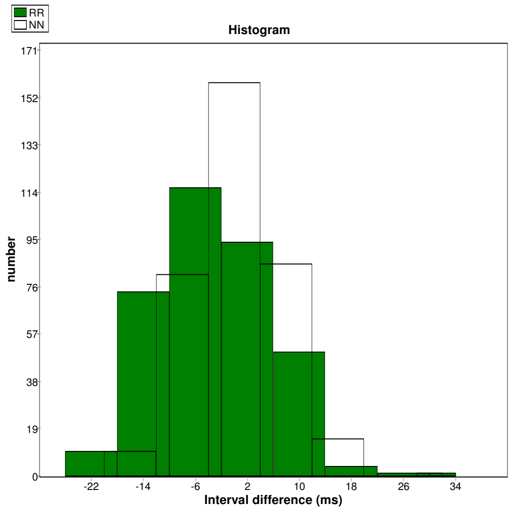

Binsize (ms) = 8

**HRV parameters**	**NN**	**RR**
SDNN (ms)	16	16
Triangular Interpolation (ms)	72	80
Triangular Index	5.40	4.94

**HRV parameters**	**NN**	**RR**
SDSD (ms)	7	9
RMSSD (ms)	7	9
NN50	0	0
NN50(1)	0	0
NN50(2)	0	0
pNN50	0.00	0.00
pNN50(1)	0.00	0.00
pNN50(2)	0.00	0.00
Logarithmic Index	1.95	1.55
SD(Logarithmic Index)	0.23	0.26

**Interval statistics**	**NN**	**RR**
Number	351	351
Minimum (ms)	817	810
Maximum (ms)	896	896
Range (ms)	79	86
Avg (ms)	852	852
SD (ms)	16	16
AvgDev (ms)	13	13
p5 (ms)	826	826
p50 (ms)	852	852
p95 (ms)	882	881
Skewness	0.05	0.05
Kurtosis	2.58	2.62

**Interval statistics**	**NN**	**RR**
Number	350	350
Minimum (ms)	-20	-22
Maximum (ms)	35	35
Range (ms)	55	57
Avg (ms)	0	0
SD (ms)	7	9
AvgDev (ms)	5	7
p5 (ms)	-12	-13
p50 (ms)	0	0
p95 (ms)	11	13
Skewness	0.28	0.24
Kurtosis	4.36	3.28

Cardio Control NV. User: Anton

Heart Rate Variability: Frequency Domain Analysis

**Name:**	015, 015 015	**Birthdate:**	26/01/1964
**Number:**	015	**Recorded:**	01/05/2018 17:03:36
**Gender:**	Male		

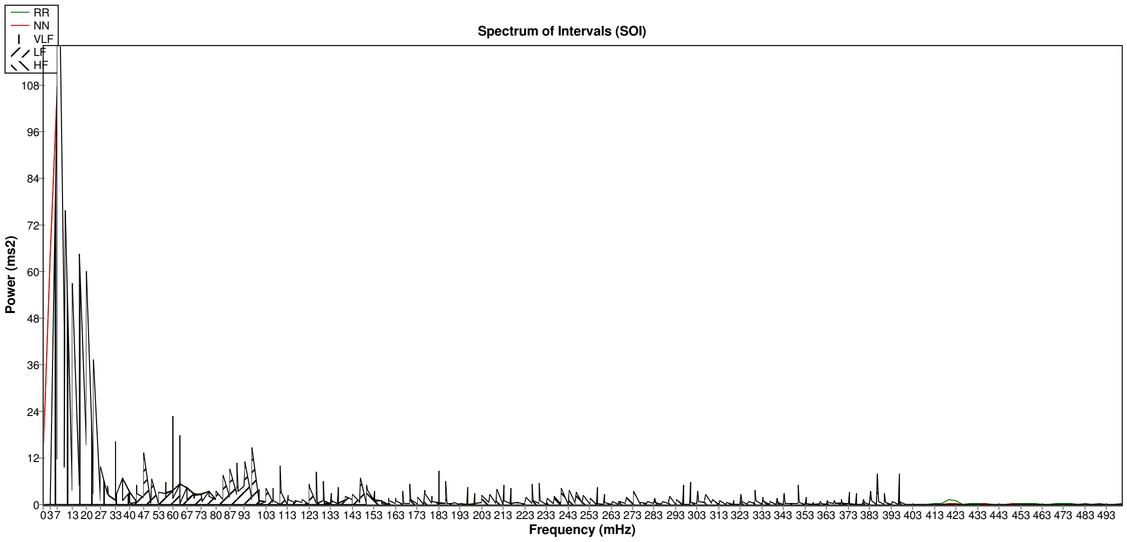

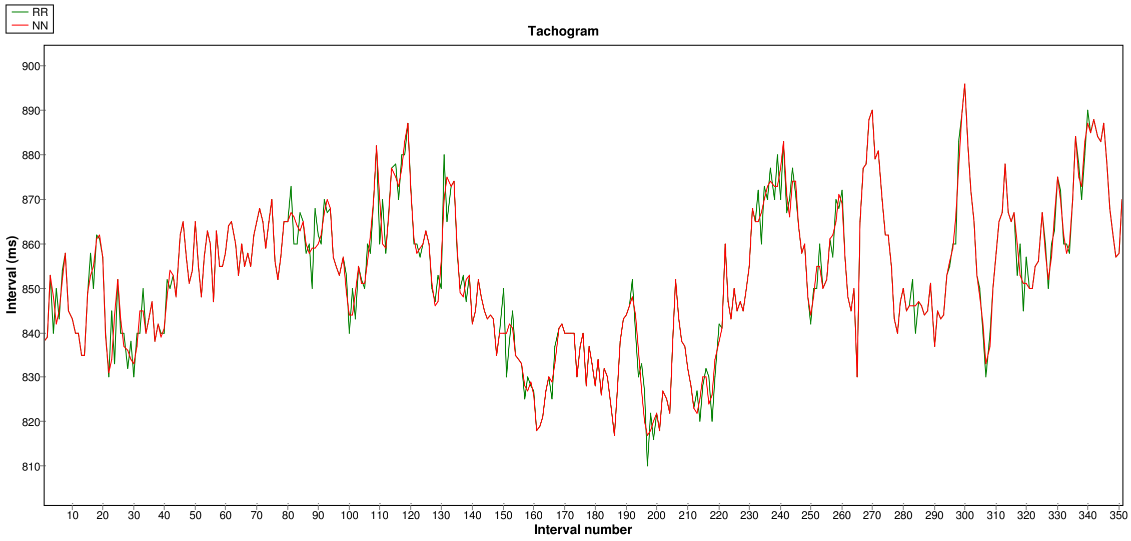

**HRV parameters**	**NN**	**RR**
TP (ms2)	262	261
VLF (ms2)	199	199
LF (ms2)	49	48
HF (ms2)	14	14
LF/HF	3.63	3.42
LF normalized	78.38	77.38
HF normalized	21.62	22.62
VLF peak (mHz)	7	7
LF peak (mHz)	63	63
HF peak (mHz)	153	153

**HRV spectral settings**	
Spectrum of Intervals (SOI)	
Frequency resolution (mHz)	3
VLF lower boundary (mHz)	3
VLF upper boundary (mHz)	40
LF upper boundary (mHz)	150
HF upper boundary (mHz)	400
Smoothing factor	1
Tapering	Hann
Fourier transform	DFT
Sample frequency (Hz)	1.17
Interval correction	Annotation
Interval threshold (%)	10
