# Supplementary material for: The effects of a 3-day mountain bike cycling race on the autonomic nervous system (ANS) and heart rate variability in amateur cyclists: a prospective quantitative research design
Source: BMC Sports Sci Med Rehabil. 2023 Jan 2;15:2. doi: 10.1186/s13102-022-00614-y (PMC9808932; doi:10.1186/s13102-022-00614-y)
Supplement: Supplementary file 1 — Additional file 1. Individual data of Participants. [file 13102_2022_614_MOESM1_ESM.zip › Individual data of Participants/HRV Data/014/ECG_014_20180503173757_.PDF]

Anton Swart Biokinetic Rehabilitation Practice

Name: 015 015 015
Number: 015
Gender: Male
Birthdate: 26/01/1964 54 years

P / PQ: 128 ms / 190 ms
QRS: 103 ms
QT / QTc / QTd: 392 ms / 441 ms / -
P/QRS/T axis: 75° / 71° / 81°
Heartrate: 88 bpm

Recorded: 03/05/2018 17:37:57
Recorded by: Mr. Anton Swart
Referring physician:
Ordering physician:
Attending physician:
Location: Anton Swart Biokinetic Rehabilitation Practi
Comment:

UNCONFIRMED INTERPRETATION - MD SHOULD REVIEW

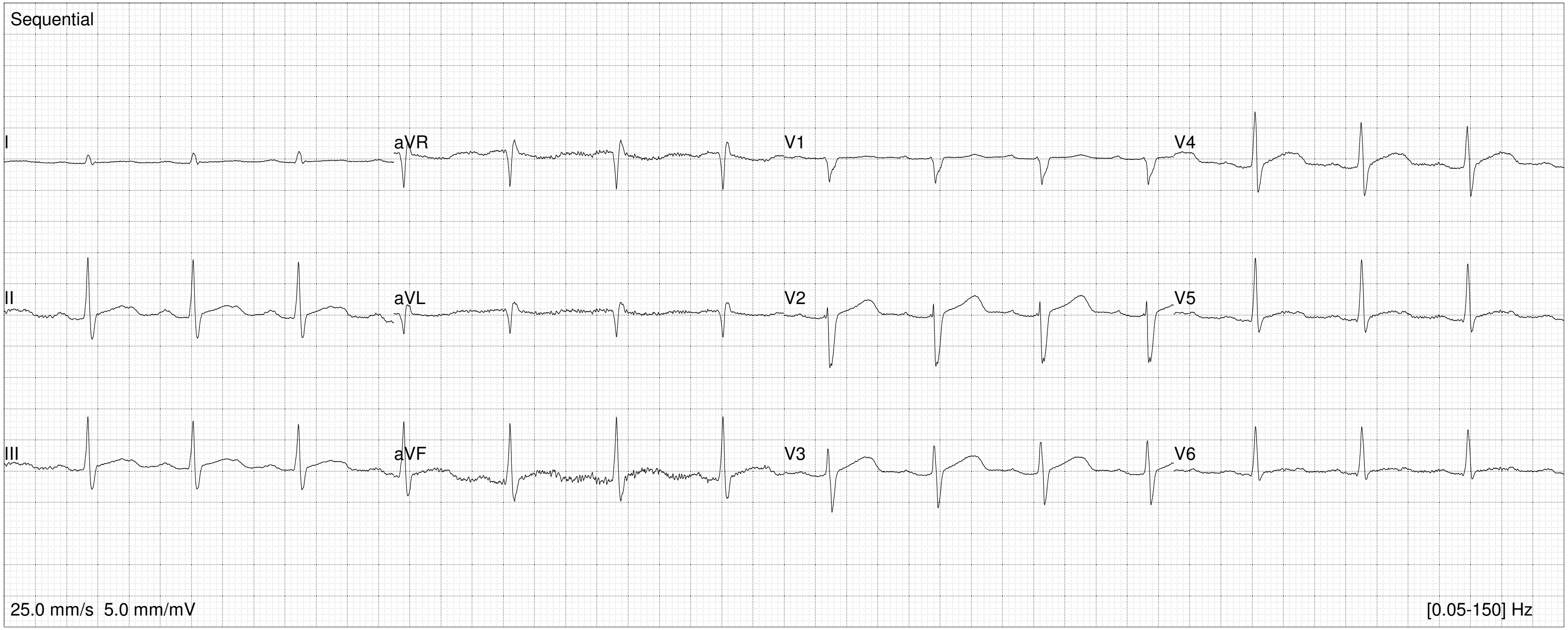

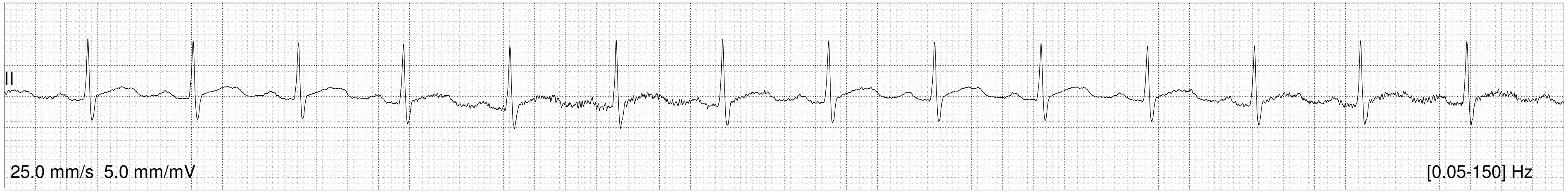

Anton Swart Biokinetic Rehabilitation Practice

Name:	015 015 015	Recorded:	03/05/2018 17:37:57	UNCONFIRMED INTERPRETATION - MD SHOULD REVIEW
Number:	015	Recorded by:	Mr. Anton Swart	
Gender:	Male	Referring physician:		
Birthdate:	26/01/1964 54 years	Location:	Anton Swart Biokinetic Rehabilitation Practice	
P / PQ:	128 ms / 190 ms	Ordering physician:		
QRS:	103 ms	Attending physician:		
QT / QTc / QTd:	392 ms / 441 ms / -	Comment:		
P/QRS/T axis:	75° / 71° / 81°			
Heartrate:	88 bpm			

**Beats**		**RR**	
Total:	439	Minimum	500 ms
Normal:	439	Maximum	870 ms
Other:	0	Mean:	680 ms
		SD:	21 ms

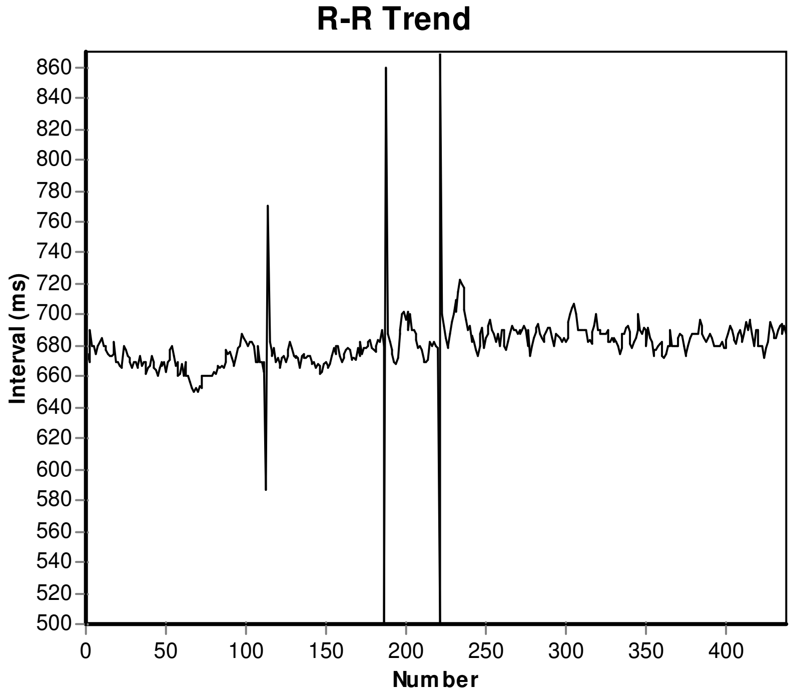

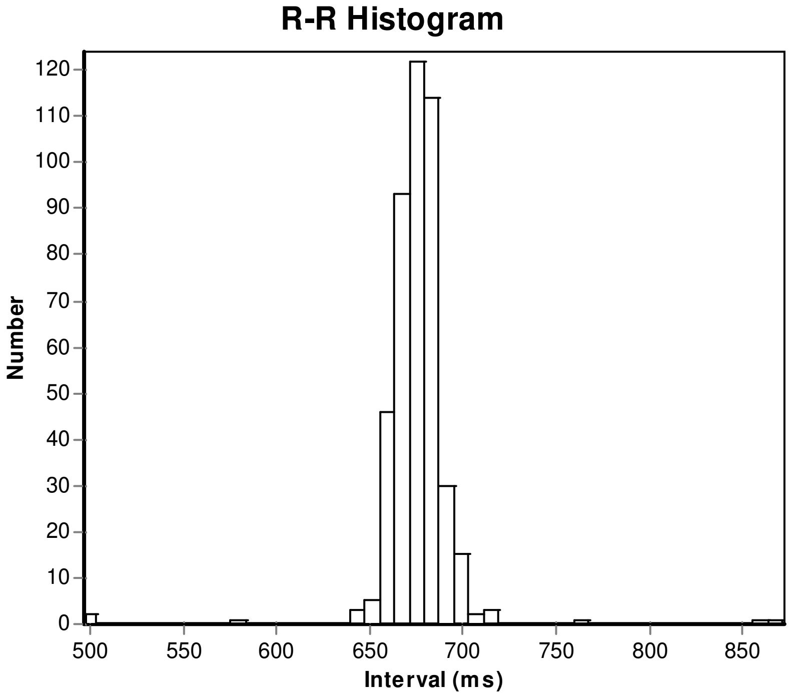

Cardio Control NV. User: Anton

Heart Rate Variability: Time Domain Analysis

**Name:**	015, 015 015	**Birthdate:**	26/01/1964
**Number:**	015	**Recorded:**	03/05/2018 17:37:57
**Gender:**	Male		

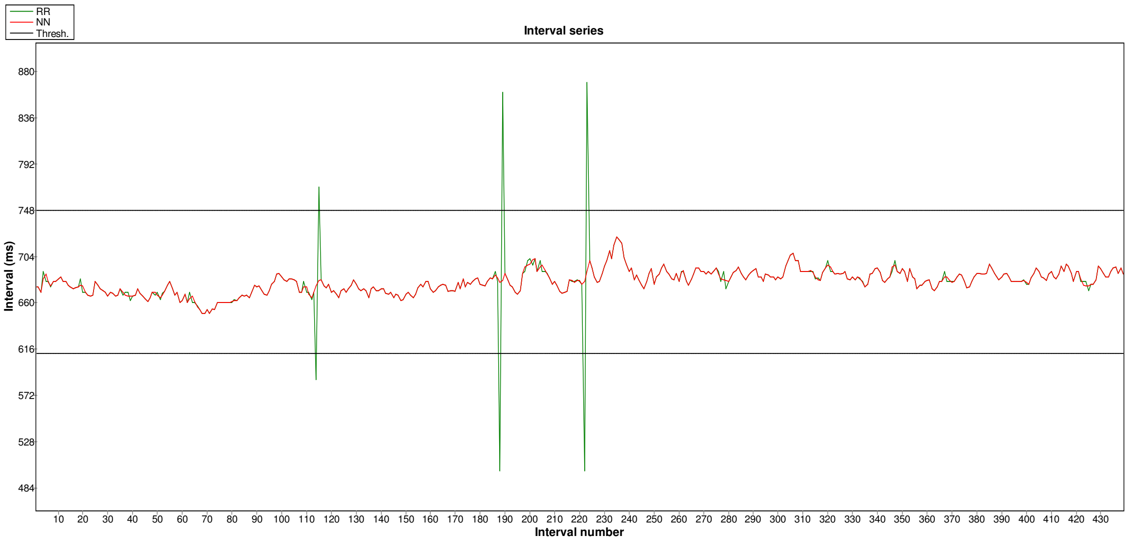

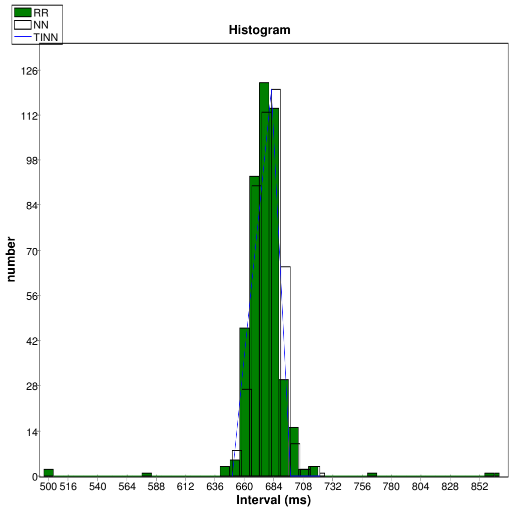

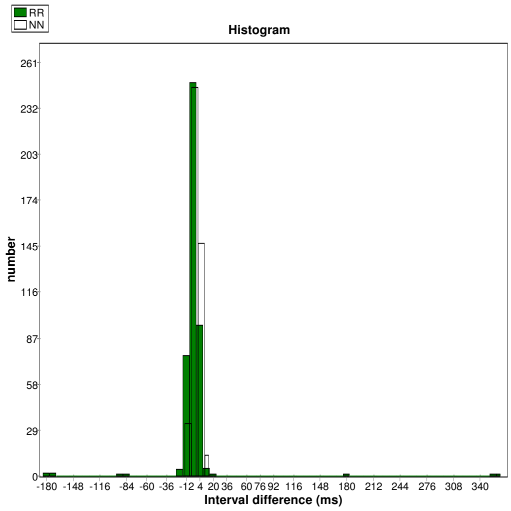

Binsize (ms) = 8

**HRV parameters**	**NN**	**RR**
SDNN (ms)	11	21
Triangular Interpolation (ms)	48	48
Triangular Index	3.66	3.60

**HRV parameters**	**NN**	**RR**
SDSD (ms)	5	32
RMSSD (ms)	5	32
NN50	0	9
NN50(1)	0	6
NN50(2)	0	3
pNN50	0.00	0.02
pNN50(1)	0.00	0.01
pNN50(2)	0.00	0.01
Logarithmic Index	2.99	0.59
SD(Logarithmic Index)	0.45	0.19

**Interval statistics**	**NN**	**RR**
Number	439	439
Minimum (ms)	650	500
Maximum (ms)	723	870
Range (ms)	73	370
Avg (ms)	680	680
SD (ms)	11	21
AvgDev (ms)	8	11
p5 (ms)	662	660
p50 (ms)	680	680
p95 (ms)	697	700
Skewness	0.16	0.23
Kurtosis	3.88	49.40

**Interval statistics**	**NN**	**RR**
Number	438	438
Minimum (ms)	-14	-180
Maximum (ms)	16	370
Range (ms)	30	550
Avg (ms)	0	0
SD (ms)	5	32
AvgDev (ms)	4	8
p5 (ms)	-7	-10
p50 (ms)	0	0
p95 (ms)	8	10
Skewness	0.13	5.61
Kurtosis	3.30	88.10

Cardio Control NV. User: Anton

Heart Rate Variability: Frequency Domain Analysis

**Name:**	015, 015 015	**Birthdate:**	26/01/1964
**Number:**	015	**Recorded:**	03/05/2018 17:37:57
**Gender:**	Male		

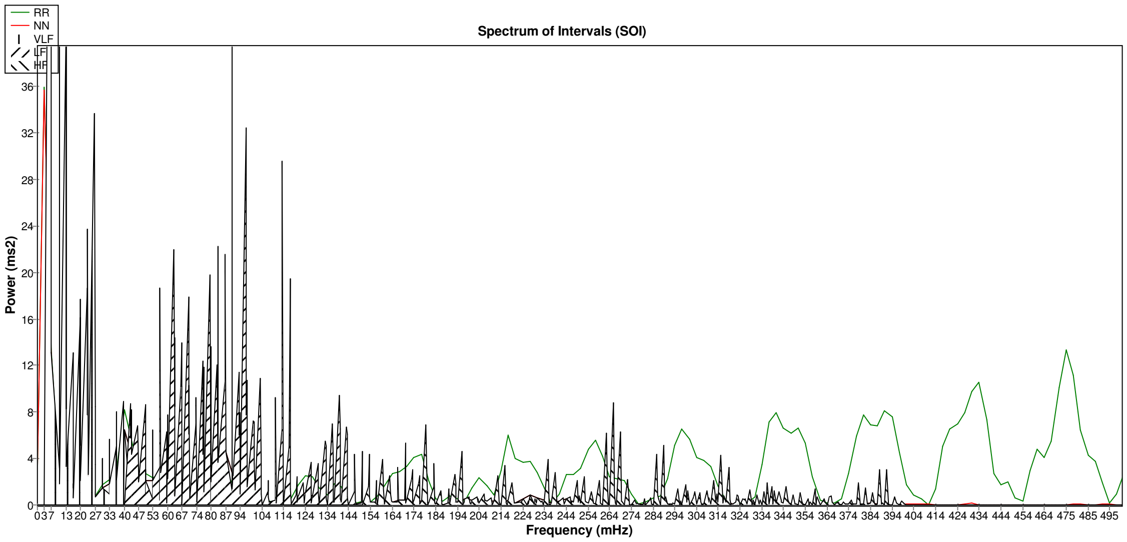

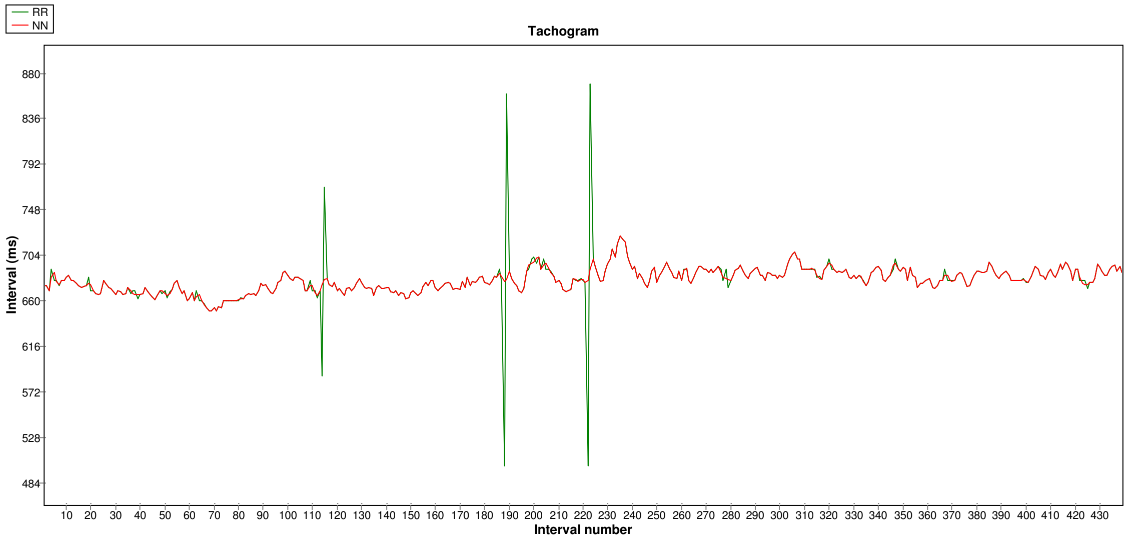

HRV parameters	NN	RR
TP (ms2)	87	306
VLF (ms2)	36	40
LF (ms2)	39	45
HF (ms2)	12	221
LF/HF	3.22	0.20
LF normalized	76.30	16.88
HF normalized	23.70	83.12
VLF peak (mHz)	7	7
LF peak (mHz)	87	43
HF peak (mHz)	227	391

HRV spectral settings	
Spectrum of Intervals (SOI)	
Frequency resolution (mHz)	3
VLF lower boundary (mHz)	3
VLF upper boundary (mHz)	40
LF upper boundary (mHz)	150
HF upper boundary (mHz)	400
Smoothing factor	1
Tapering	Hann
Fourier transform	DFT
Sample frequency (Hz)	1.47
Interval correction	Annotation
Interval threshold (%)	10
