# Supplementary material for: The effects of a 3-day mountain bike cycling race on the autonomic nervous system (ANS) and heart rate variability in amateur cyclists: a prospective quantitative research design
Source: BMC Sports Sci Med Rehabil. 2023 Jan 2;15:2. doi: 10.1186/s13102-022-00614-y (PMC9808932; doi:10.1186/s13102-022-00614-y)
Supplement: Supplementary file 1 — Additional file 1. Individual data of Participants. [file 13102_2022_614_MOESM1_ESM.zip › Individual data of Participants/HRV Data/014/ECG_014_20180504133244_.PDF]

Anton Swart Biokinetic Rehabilitation Practice

Name: 015 015 015
Number: 015
Gender: Male
Birthdate: 26/01/1964 54 years

Recorded: 04/05/2018 13:32:44
Recorded by: Mr. Anton Swart
Referring physician:
Ordering physician:
Attending physician:
Location: Anton Swart Biokinetic Rehabilitation Practi
Comment:

UNCONFIRMED INTERPRETATION - MD SHOULD REVIEW

P / PQ: 128 ms / 177 ms
QRS: 110 ms
QT / QTc / QTd: 380 ms / 459 ms / -
P/QRS/T axis: 76° / 67° / 85°
Heartrate: 105 bpm

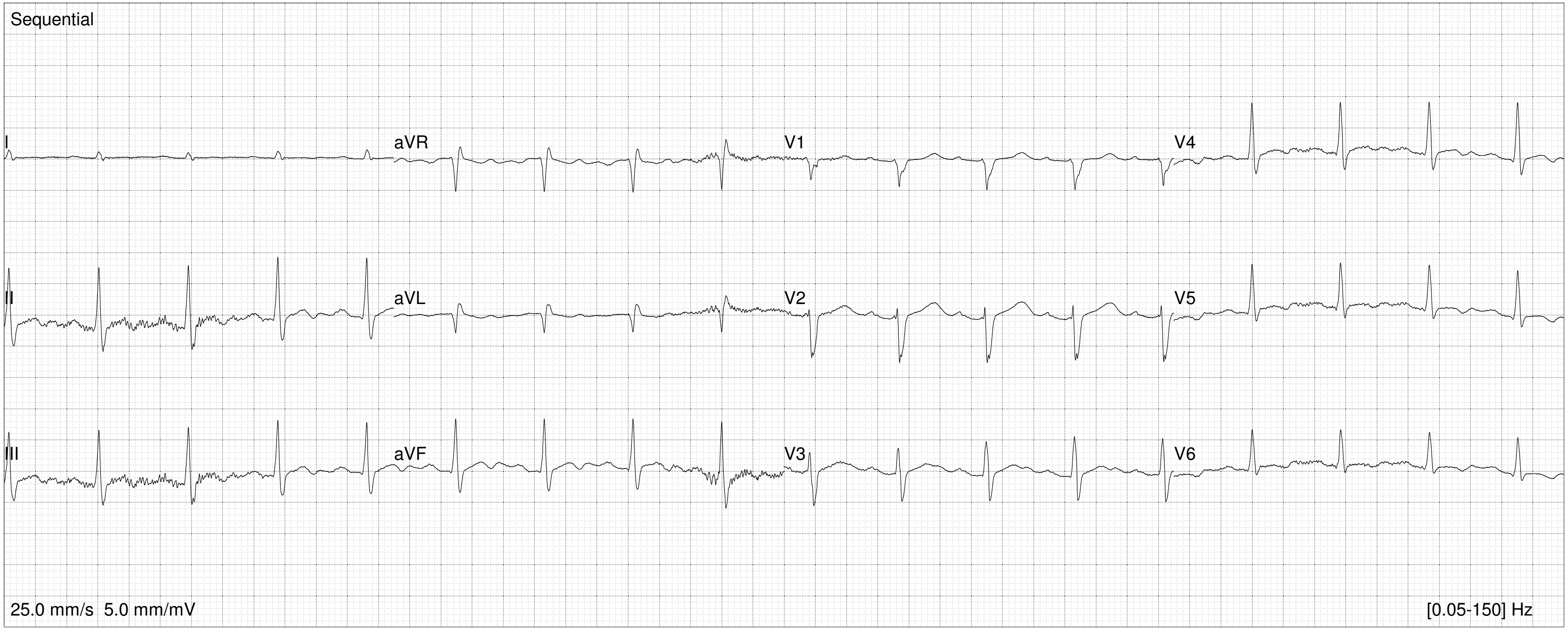

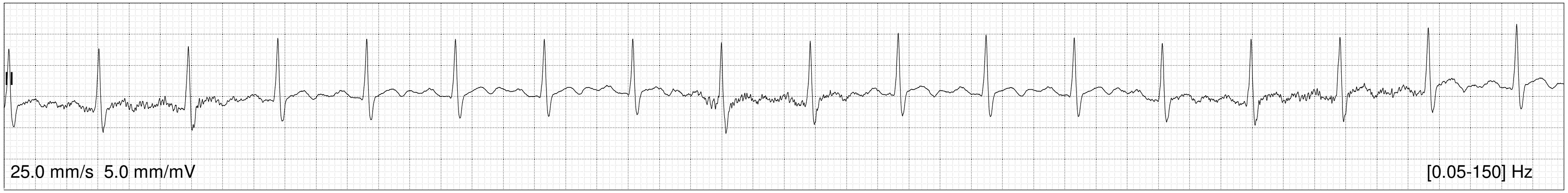

Anton Swart Biokinetic Rehabilitation Practice

Name:	015 015 015	Recorded:	04/05/2018 13:32:44	UNCONFIRMED INTERPRETATION - MD SHOULD REVIEW
Number:	015	Recorded by:	Mr. Anton Swart	
Gender:	Male	Referring physician:		
Birthdate:	26/01/1964 54 years	Location:	Anton Swart Biokinetic Rehabilitation Practice	
P / PQ:	128 ms / 177 ms	Ordering physician:		
QRS:	110 ms	Attending physician:		
QT / QTc / QTd:	380 ms / 459 ms / -	Comment:		
P/QRS/T axis:	76° / 67° / 85°			
Heartrate:	105 bpm			

**Beats**		**RR**	
Total:	523	Minimum	420 ms
Normal:	523	Maximum	730 ms
Other:	0	Mean:	573 ms
		SD:	28 ms

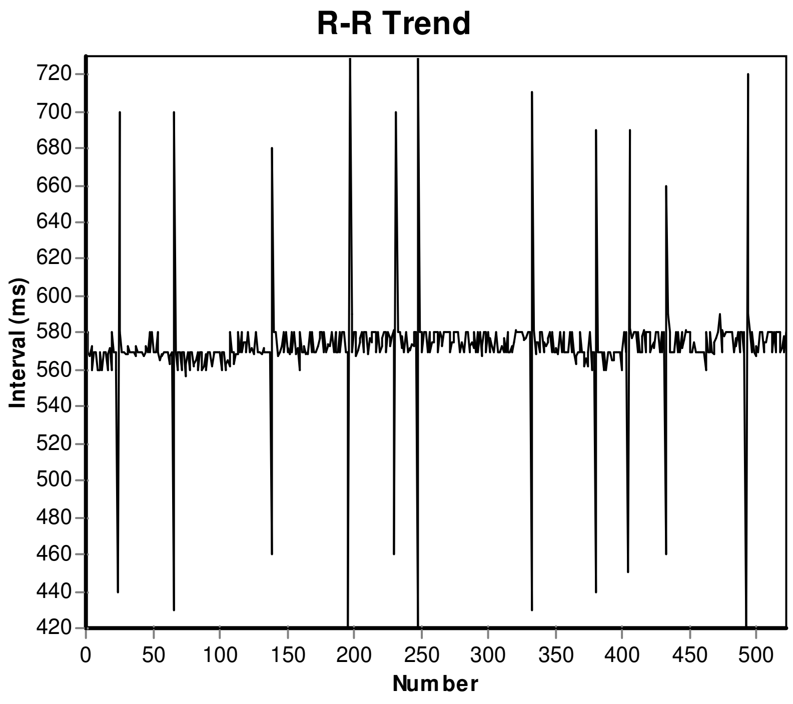

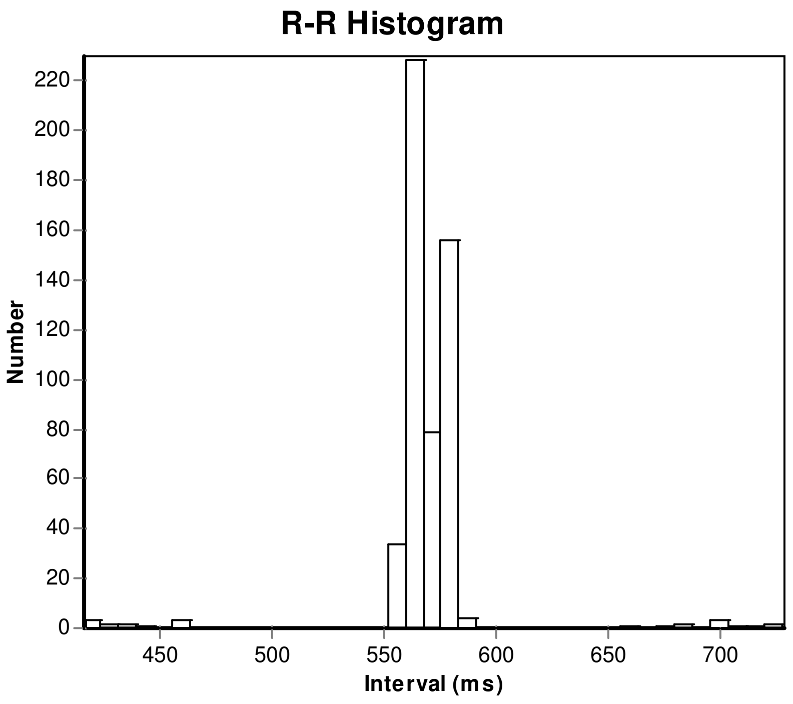

Heart Rate Variability: Time Domain Analysis

**Name:**	015, 015 015	**Birthdate:**	26/01/1964
**Number:**	015	**Recorded:**	04/05/2018 13:32:44
**Gender:**	Male		

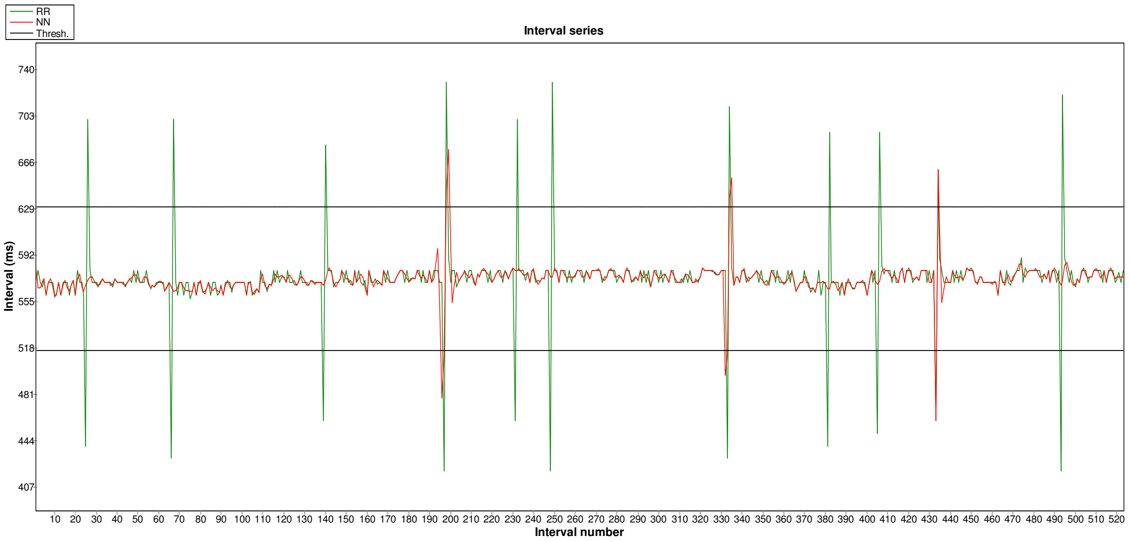

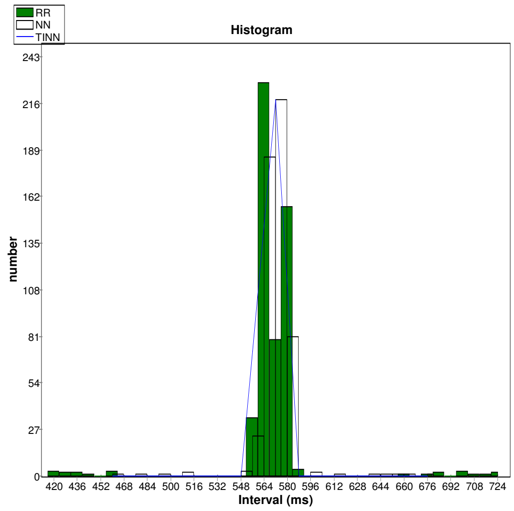

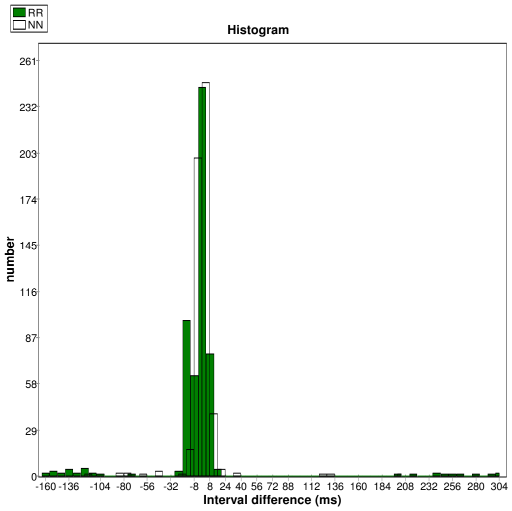

Binsize (ms) = 8

HRV parameters	NN	RR
SDNN (ms)	13	28
Triangular Interpolation (ms)	40	32
Triangular Index	2.40	2.29

HRV parameters	NN	RR
SDSD (ms)	16	47
RMSSD (ms)	16	47
NN50	9	33
NN50(1)	6	22
NN50(2)	3	11
pNN50	0.02	0.06
pNN50(1)	0.01	0.04
pNN50(2)	0.01	0.02
Logarithmic Index	0.46	0.18
SD(Logarithmic Index)	0.13	0.05

Interval statistics	NN	RR
Number	523	523
Minimum (ms)	460	420
Maximum (ms)	676	730
Range (ms)	216	310
Avg (ms)	573	573
SD (ms)	13	28
AvgDev (ms)	6	10
p5 (ms)	563	560
p50 (ms)	573	570
p95 (ms)	582	580
Skewness	-0.20	-0.26
Kurtosis	38.93	23.32

Interval statistics	NN	RR
Number	522	522
Minimum (ms)	-120	-160
Maximum (ms)	200	310
Range (ms)	320	470
Avg (ms)	-0	0
SD (ms)	16	47
AvgDev (ms)	6	16
p5 (ms)	-9	-14
p50 (ms)	0	0
p95 (ms)	10	10
Skewness	3.54	2.91
Kurtosis	70.00	24.80

Heart Rate Variability: Frequency Domain Analysis

**Name:**	015, 015 015	**Birthdate:**	26/01/1964
**Number:**	015	**Recorded:**	04/05/2018 13:32:44
**Gender:**	Male		

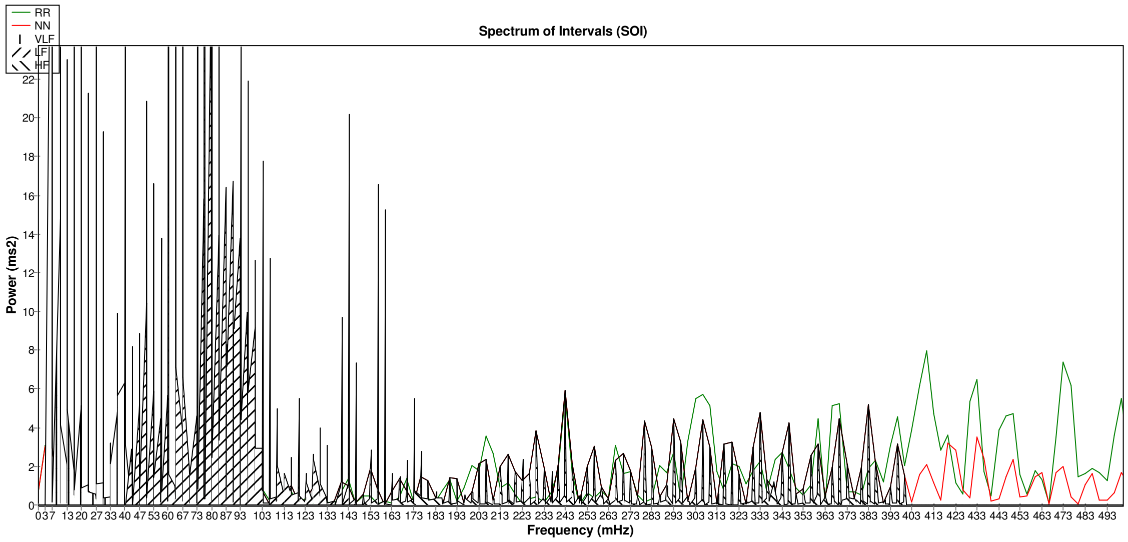

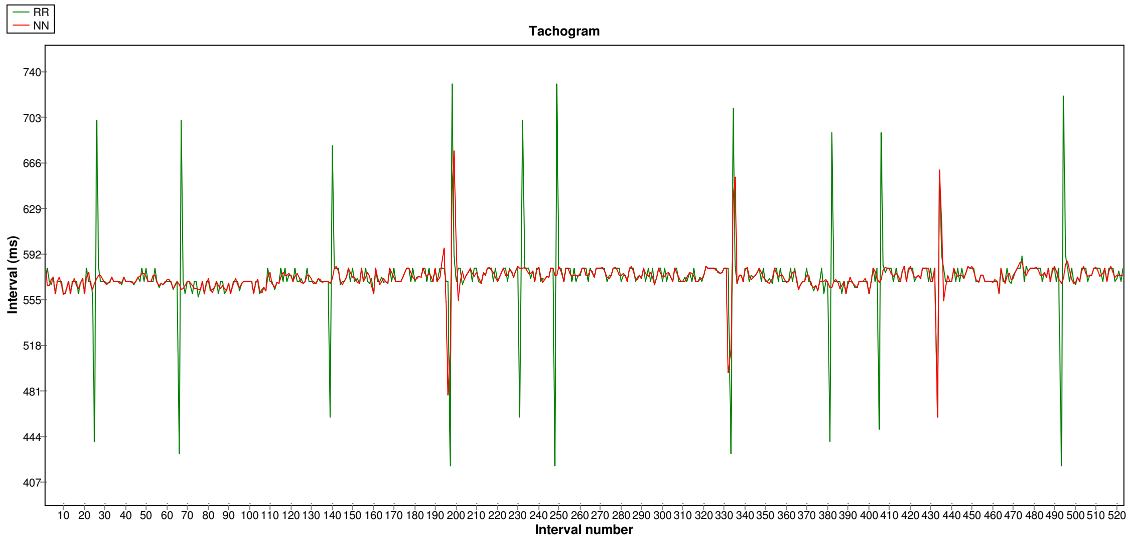

**HRV parameters**	**NN**	**RR**
TP (ms2)	154	135
VLF (ms2)	5	5
LF (ms2)	11	10
HF (ms2)	138	120
LF/HF	0.08	0.08
LF normalized	7.36	7.83
HF normalized	92.64	92.17
VLF peak (mHz)	7	7
LF peak (mHz)	140	143
HF peak (mHz)	243	306

**HRV spectral settings**	
Spectrum of Intervals (SOI)	
Frequency resolution (mHz)	3
VLF lower boundary (mHz)	3
VLF upper boundary (mHz)	40
LF upper boundary (mHz)	150
HF upper boundary (mHz)	400
Smoothing factor	1
Tapering	Hann
Fourier transform	DFT
Sample frequency (Hz)	1.74
Interval correction	Annotation
Interval threshold (%)	10
