# Supplementary material for: The effects of a 3-day mountain bike cycling race on the autonomic nervous system (ANS) and heart rate variability in amateur cyclists: a prospective quantitative research design
Source: BMC Sports Sci Med Rehabil. 2023 Jan 2;15:2. doi: 10.1186/s13102-022-00614-y (PMC9808932; doi:10.1186/s13102-022-00614-y)
Supplement: Supplementary file 1 — Additional file 1. Individual data of Participants. [file 13102_2022_614_MOESM1_ESM.zip › Individual data of Participants/HRV Data/014/ECG_014_20180505125552_.PDF]

Anton Swart Biokinetic Rehabilitation Practice

UNCONFIRMED INTERPRETATION - MD SHOULD REVIEW

Name:	015 015 015	Recorded:	05/05/2018 12:55:52
Number:	015	Recorded by:	Mr. Anton Swart
Gender:	Male	Referring physician:	
Birthdate:	26/01/1964 54 years	Ordering physician:	
		Attending physician:	
		Location:	Anton Swart Biokinetic Rehabilitation Practi
		Comment:	

P / PQ:	135 ms / 183 ms
QRS:	105 ms
QT / QTc / QTd:	394 ms / 464 ms / -
P/QRS/T axis:	76° / 56° / 81°
Heartrate:	100 bpm

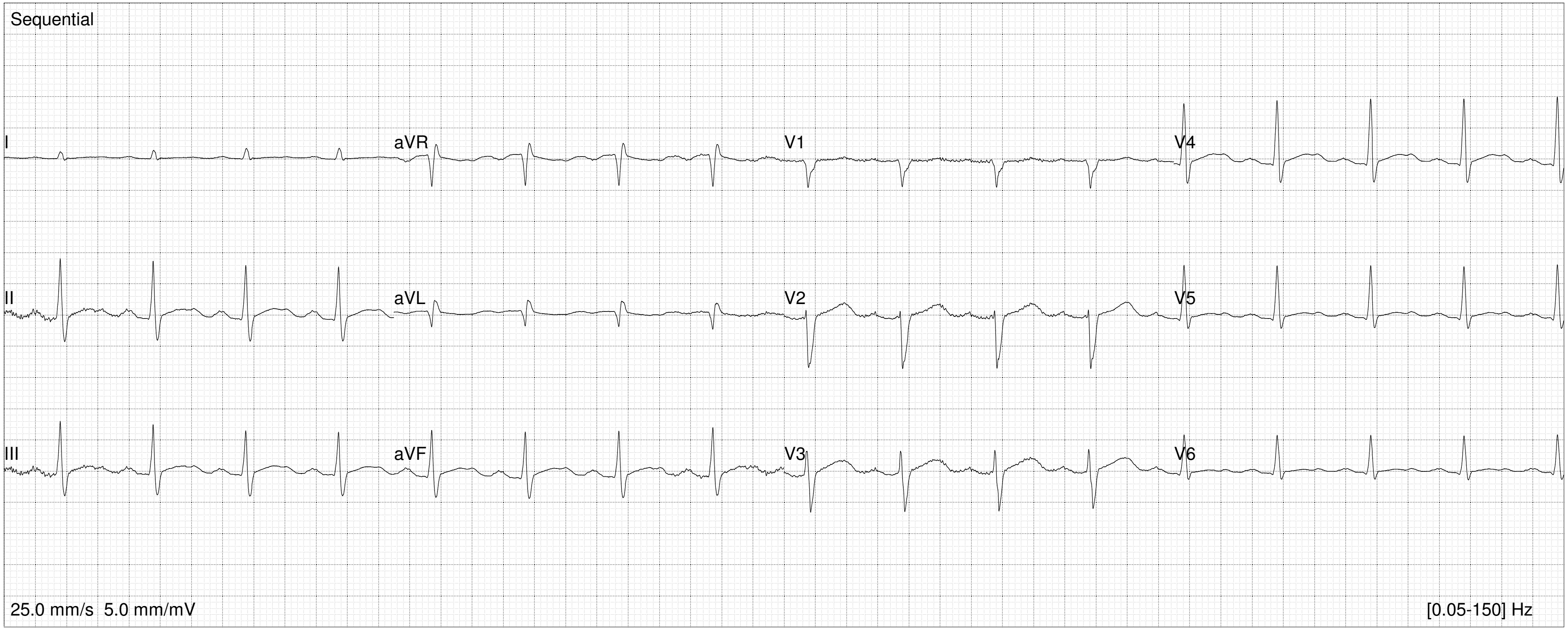

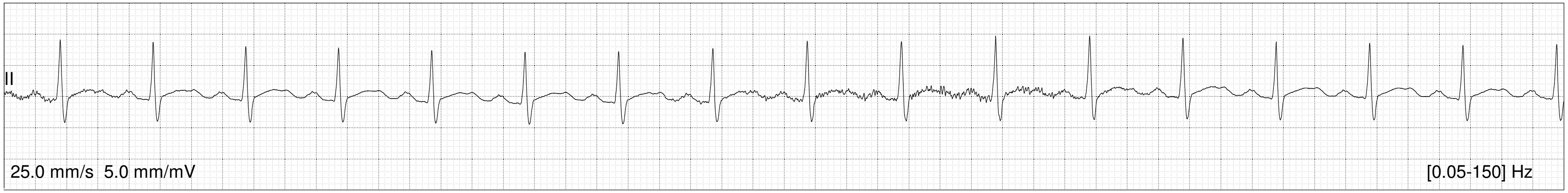

Anton Swart Biokinetic Rehabilitation Practice

Name:	015 015 015	Recorded:	05/05/2018 12:55:52	UNCONFIRMED INTERPRETATION - MD SHOULD REVIEW
Number:	015	Recorded by:	Mr. Anton Swart	
Gender:	Male	Referring physician:		
Birthdate:	26/01/1964 54 years	Location:	Anton Swart Biokinetic Rehabilitation Practice	
		Ordering physician:		
		Attending physician:		
		Comment:		

P / PQ:	135 ms / 183 ms
QRS:	105 ms
QT / QTc / QTd:	394 ms / 464 ms / -
P/QRS/T axis:	76° / 56° / 81°
Heartrate:	100 bpm

Beats

Total:	502
Normal:	502
Other:	0

RR

Minimum	390 ms
Maximum	790 ms
Mean:	596 ms
SD:	44 ms

R-R Trend

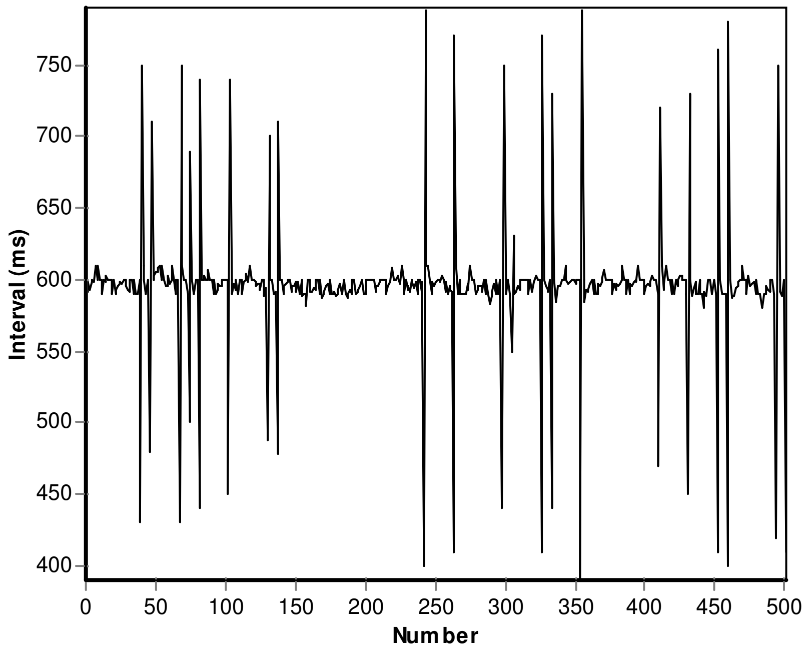

R-R Histogram

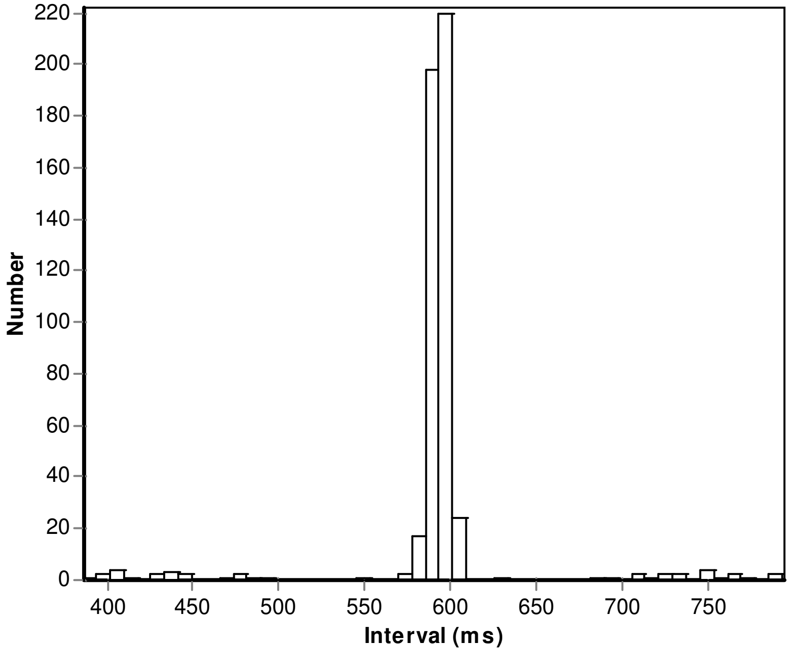

Heart Rate Variability: Time Domain Analysis

**Name:**	015, 015 015	**Birthdate:**	26/01/1964
**Number:**	015	**Recorded:**	05/05/2018 12:55:52
**Gender:**	Male		

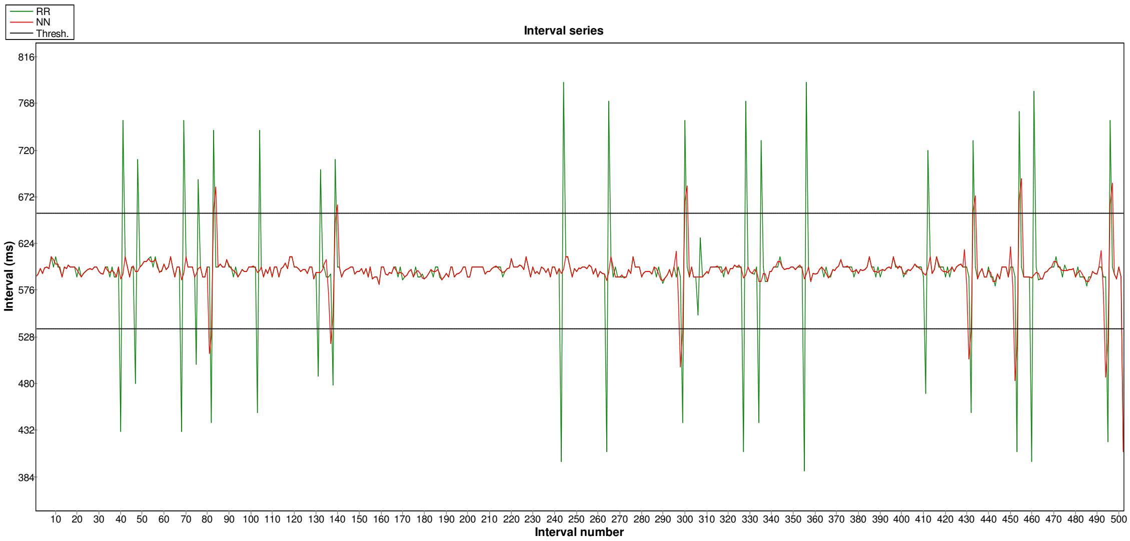

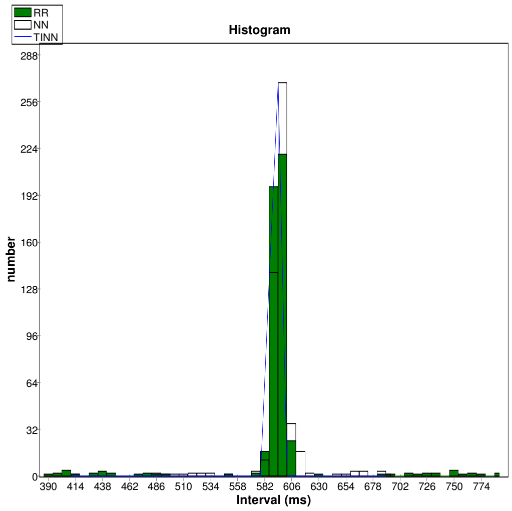

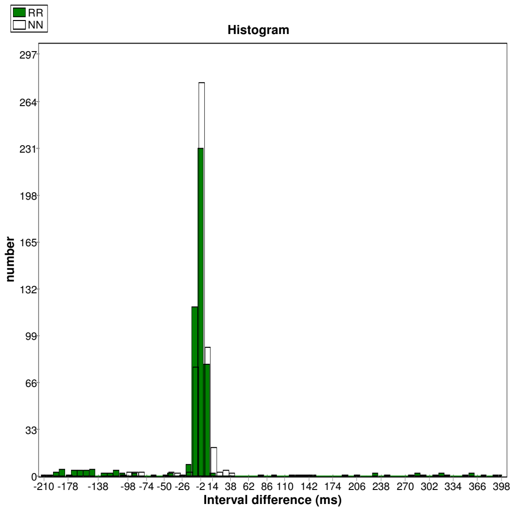

Binsize (ms) = 8

**HRV parameters**	**NN**	**RR**
SDNN (ms)	20	44
Triangular Interpolation (ms)	24	32
Triangular Index	1.87	2.28

**HRV parameters**	**NN**	**RR**
SDSD (ms)	22	74
RMSSD (ms)	22	74
NN50	19	59
NN50(1)	13	39
NN50(2)	6	20
pNN50	0.04	0.12
pNN50(1)	0.03	0.08
pNN50(2)	0.01	0.04
Logarithmic Index	0.25	0.07
SD(Logarithmic Index)	0.06	0.02

**Interval statistics**	**NN**	**RR**
Number	502	502
Minimum (ms)	410	390
Maximum (ms)	691	790
Range (ms)	281	400
Avg (ms)	596	596
SD (ms)	20	44
AvgDev (ms)	8	16
p5 (ms)	585	584
p50 (ms)	597	598
p95 (ms)	610	610
Skewness	-2.06	-0.48
Kurtosis	28.60	14.18

**Interval statistics**	**NN**	**RR**
Number	501	501
Minimum (ms)	-180	-210
Maximum (ms)	152	400
Range (ms)	332	610
Avg (ms)	-0	-0
SD (ms)	22	74
AvgDev (ms)	9	28
p5 (ms)	-10	-140
p50 (ms)	0	0
p95 (ms)	16	12
Skewness	0.12	2.21
Kurtosis	28.34	14.78

Cardio Control NV. User: Anton

Heart Rate Variability: Frequency Domain Analysis

**Name:**	015, 015 015	**Birthdate:**	26/01/1964
**Number:**	015	**Recorded:**	05/05/2018 12:55:52
**Gender:**	Male		

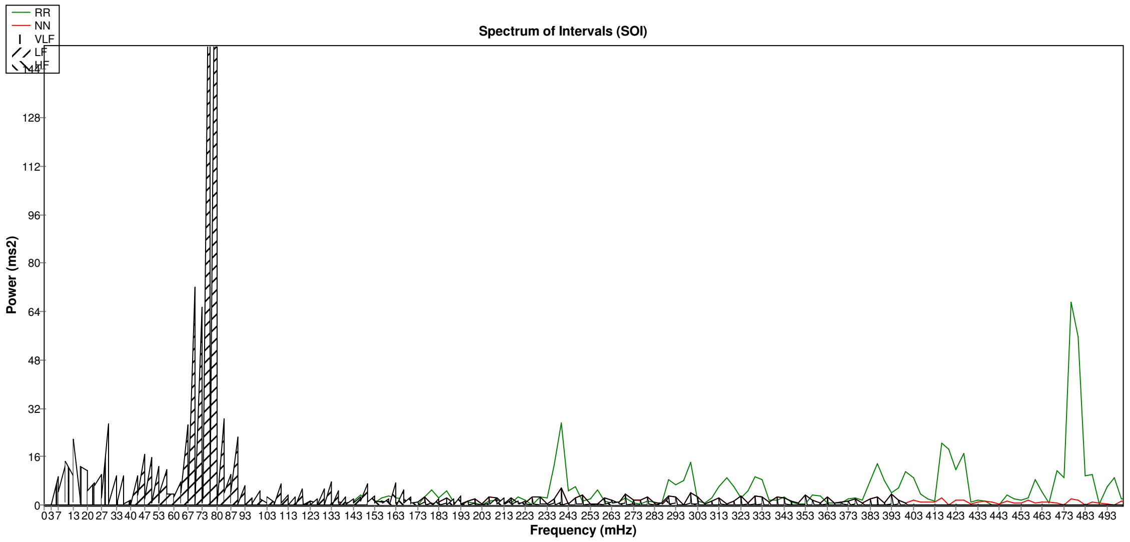

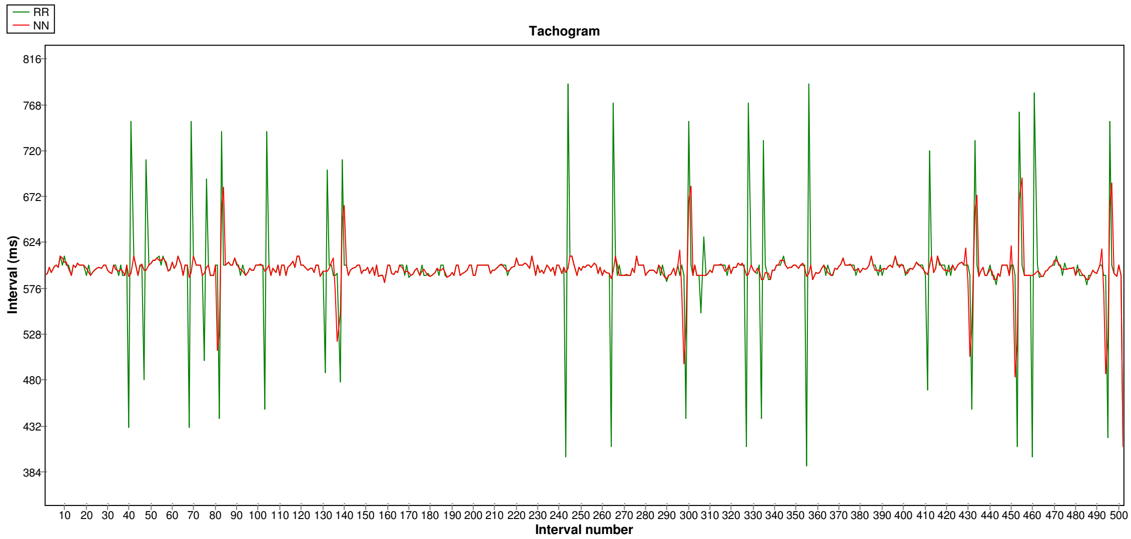

**HRV parameters**	**NN**	**RR**
TP (ms2)	144	291
VLF (ms2)	4	3
LF (ms2)	16	17
HF (ms2)	124	271
LF/HF	0.13	0.06
LF normalized	11.34	5.74
HF normalized	88.66	94.26
VLF peak (mHz)	13	13
LF peak (mHz)	147	147
HF peak (mHz)	240	240

**HRV spectral settings**	
Spectrum of Intervals (SOI)	
Frequency resolution (mHz)	3
VLF lower boundary (mHz)	3
VLF upper boundary (mHz)	40
LF upper boundary (mHz)	150
HF upper boundary (mHz)	400
Smoothing factor	1
Tapering	Hann
Fourier transform	DFT
Sample frequency (Hz)	1.68
Interval correction	Annotation
Interval threshold (%)	10
