# Supplementary material for: The effects of a 3-day mountain bike cycling race on the autonomic nervous system (ANS) and heart rate variability in amateur cyclists: a prospective quantitative research design
Source: BMC Sports Sci Med Rehabil. 2023 Jan 2;15:2. doi: 10.1186/s13102-022-00614-y (PMC9808932; doi:10.1186/s13102-022-00614-y)
Supplement: Supplementary file 1 — Additional file 1. Individual data of Participants. [file 13102_2022_614_MOESM1_ESM.zip › Individual data of Participants/HRV Data/014/ECG_014_20180506085709_.PDF]

Anton Swart Biokinetic Rehabilitation Practice

Name:	015 015 015	Recorded:	06/05/2018 08:57:09	UNCONFIRMED INTERPRETATION - MD SHOULD REVIEW
Number:	015	Recorded by:	Mr. Anton Swart	
Gender:	Male	Referring physician:		
Birthdate:	26/01/1964 54 years	Ordering physician:		
		Attending physician:		
P / PQ:	130 ms / 175 ms	Location:	Anton Swart Biokinetic Rehabilitation Practi	
QRS:	103 ms	Comment:		
QT / QTc / QTd:	423 ms / 444 ms / -			
P/QRS/T axis:	74° / 61° / 81°			
Heartrate:	72 bpm			

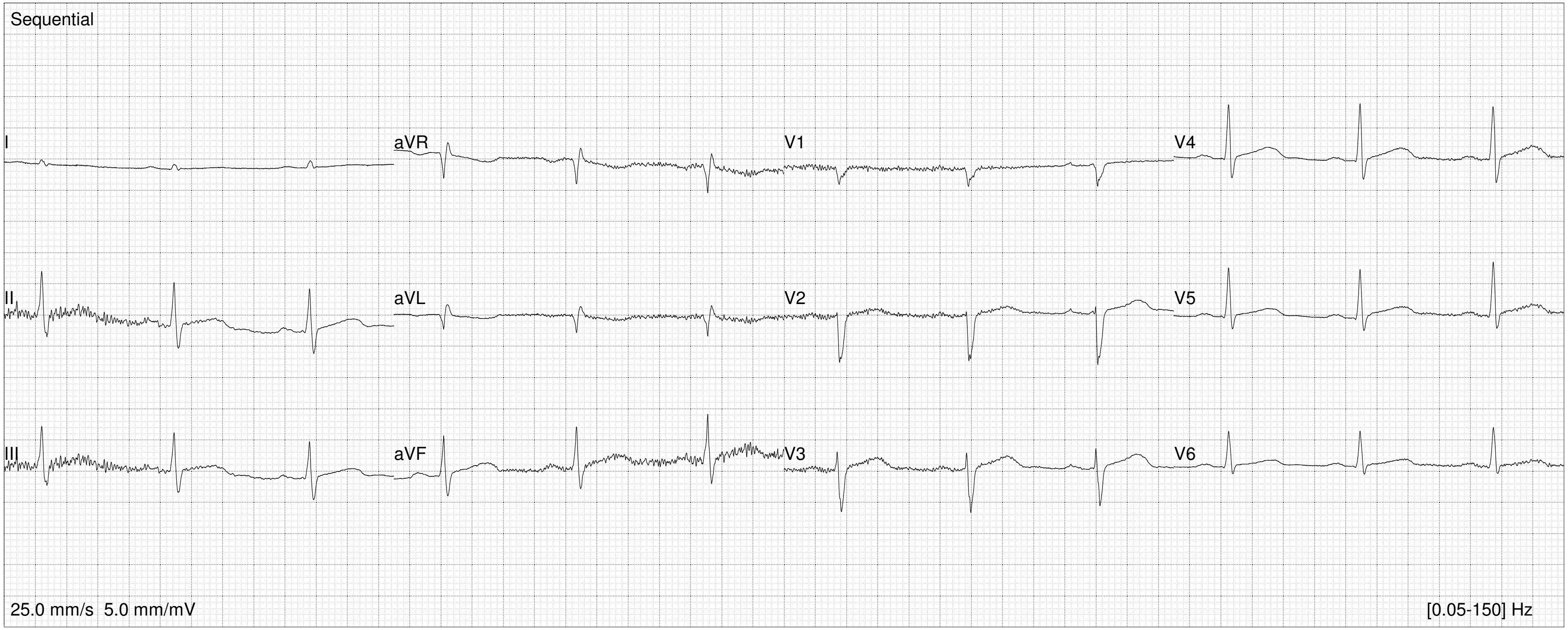

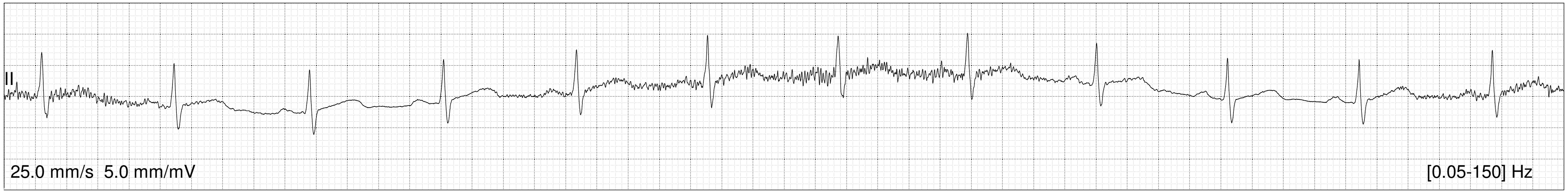

Anton Swart Biokinetic Rehabilitation Practice

Name:	015 015 015	Recorded:	06/05/2018 08:57:09	UNCONFIRMED INTERPRETATION - MD SHOULD REVIEW
Number:	015	Recorded by:	Mr. Anton Swart	
Gender:	Male	Referring physician:		
Birthdate:	26/01/1964 54 years	Location:	Anton Swart Biokinetic Rehabilitation Practice	
P / PQ:	130 ms / 175 ms	Ordering physician:		
QRS:	103 ms	Attending physician:		
QT / QTc / QTd:	423 ms / 444 ms / -	Comment:		
P/QRS/T axis:	74° / 61° / 81°			
Heartrate:	72 bpm			

**Beats**		**RR**	
Total:	365	Minimum	480 ms
Normal:	365	Maximum	1360 ms
Other:	0	Mean:	821 ms
		SD:	92 ms

R-R Trend

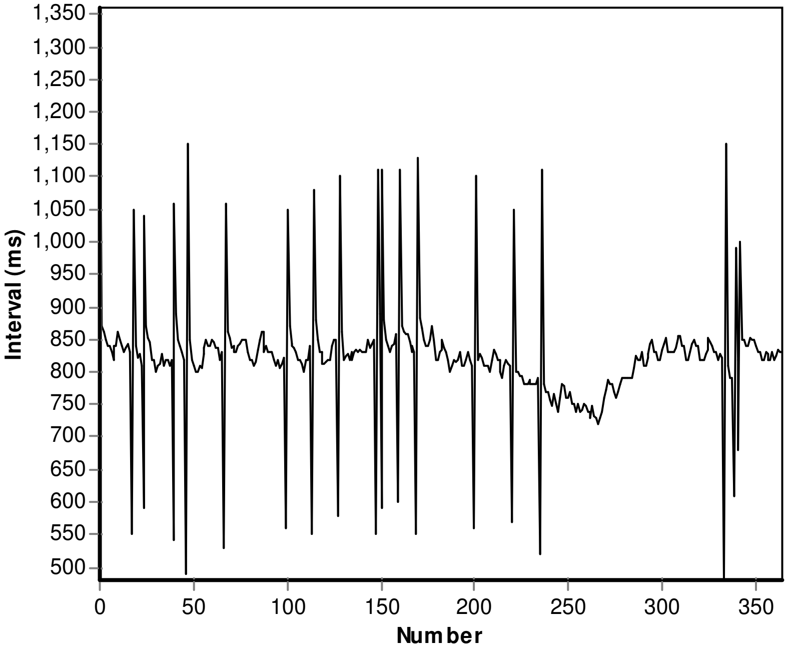

R-R Histogram

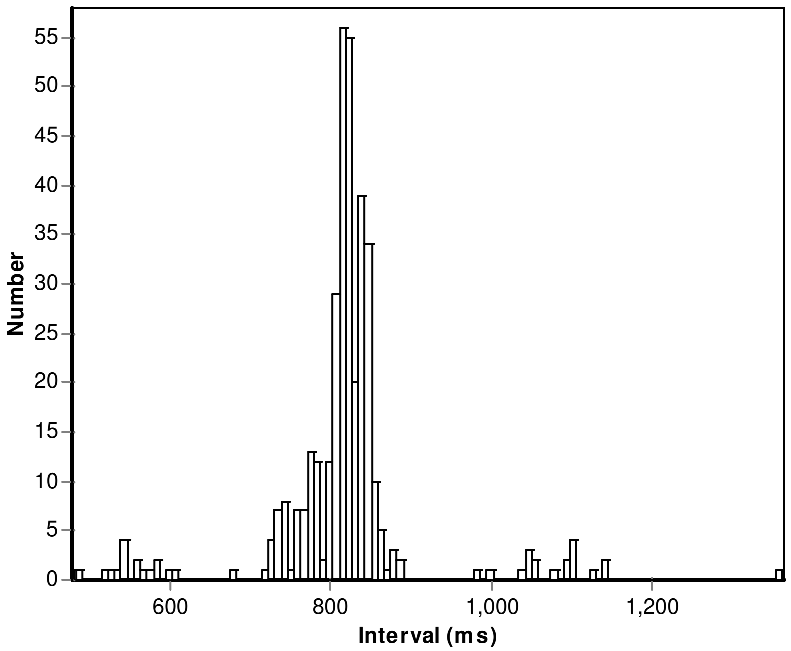

Heart Rate Variability: Time Domain Analysis

**Name:**	015, 015 015	**Birthdate:**	26/01/1964
**Number:**	015	**Recorded:**	06/05/2018 08:57:09
**Gender:**	Male		

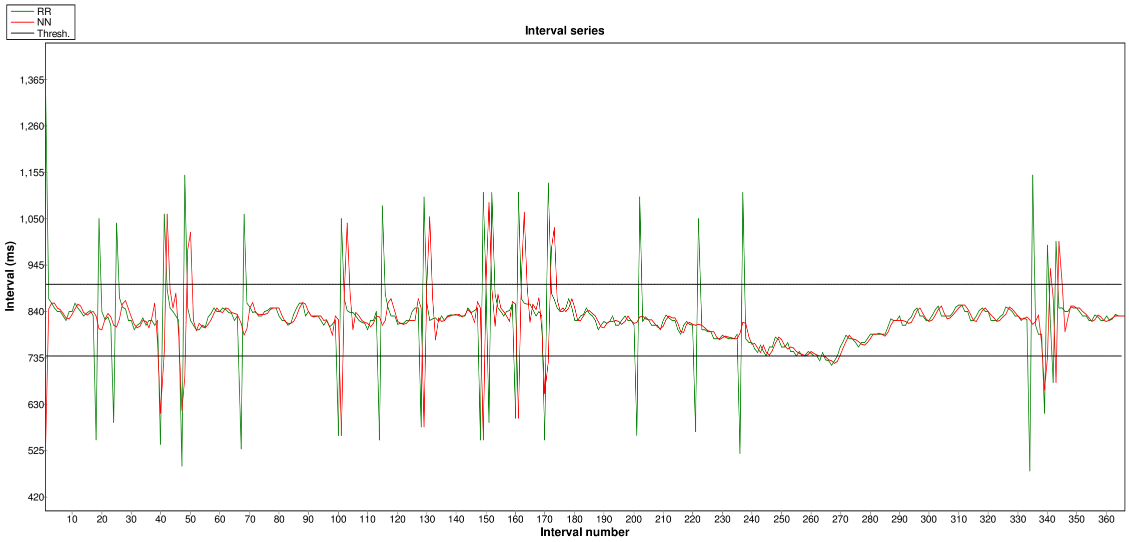

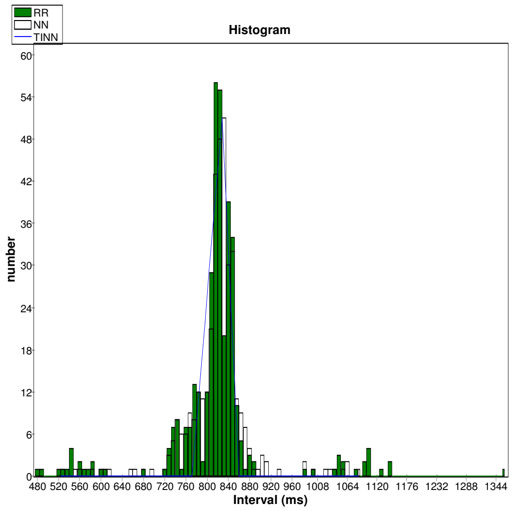

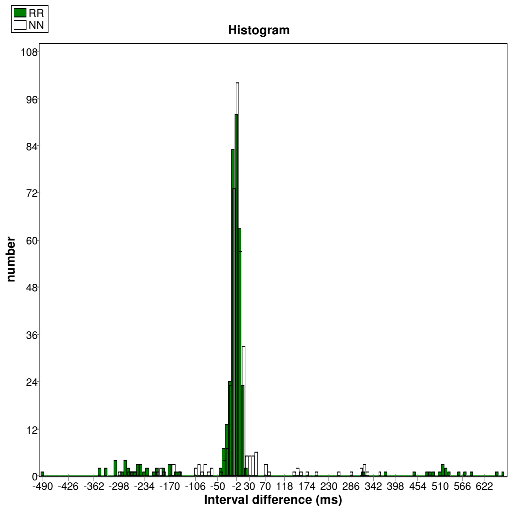

Binsize (ms) = 8

**HRV parameters**	**NN**	**RR**
SDNN (ms)	61	92
Triangular Interpolation (ms)	88	80
Triangular Index	7.18	6.52

**HRV parameters**	**NN**	**RR**
SDSD (ms)	71	144
RMSSD (ms)	71	144
NN50	47	55
NN50(1)	27	37
NN50(2)	20	18
pNN50	0.13	0.15
pNN50(1)	0.07	0.10
pNN50(2)	0.05	0.05
Logarithmic Index	0.11	0.07
SD(Logarithmic Index)	0.02	0.02

**Interval statistics**	**NN**	**RR**
Number	366	365
Minimum (ms)	524	480
Maximum (ms)	1087	1360
Range (ms)	563	880
Avg (ms)	819	821
SD (ms)	61	92
AvgDev (ms)	36	48
p5 (ms)	739	720
p50 (ms)	823	827
p95 (ms)	887	1000
Skewness	-0.21	0.50
Kurtosis	10.41	10.44

**Interval statistics**	**NN**	**RR**
Number	365	364
Minimum (ms)	-300	-490
Maximum (ms)	363	670
Range (ms)	663	1160
Avg (ms)	1	-1
SD (ms)	71	144
AvgDev (ms)	31	59
p5 (ms)	-94	-254
p50 (ms)	-2	0
p95 (ms)	72	172
Skewness	1.20	1.83
Kurtosis	13.60	11.06

Heart Rate Variability: Frequency Domain Analysis

**Name:**	015, 015 015	**Birthdate:**	26/01/1964
**Number:**	015	**Recorded:**	06/05/2018 08:57:09
**Gender:**	Male		

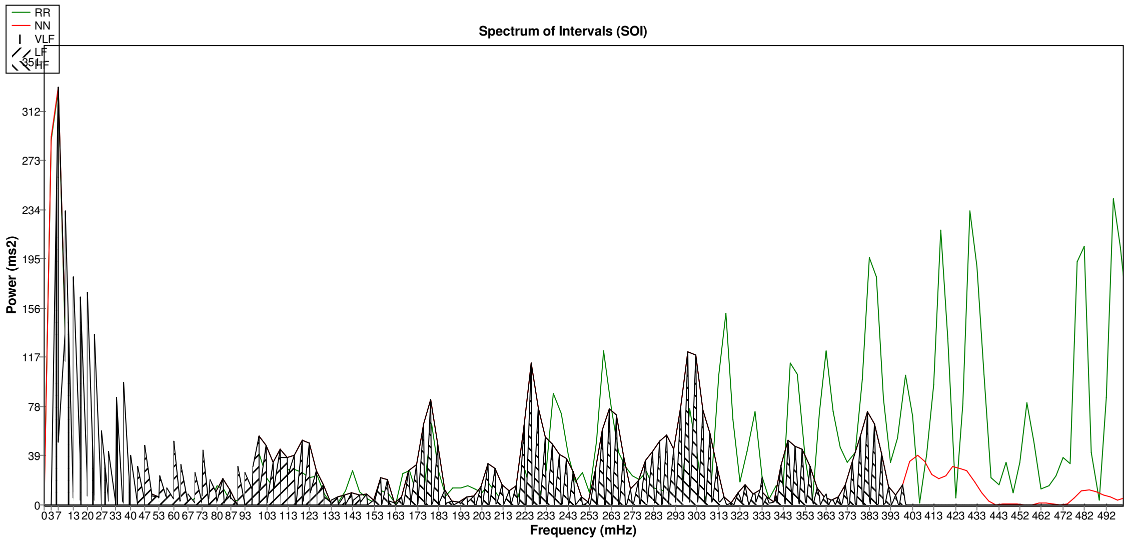

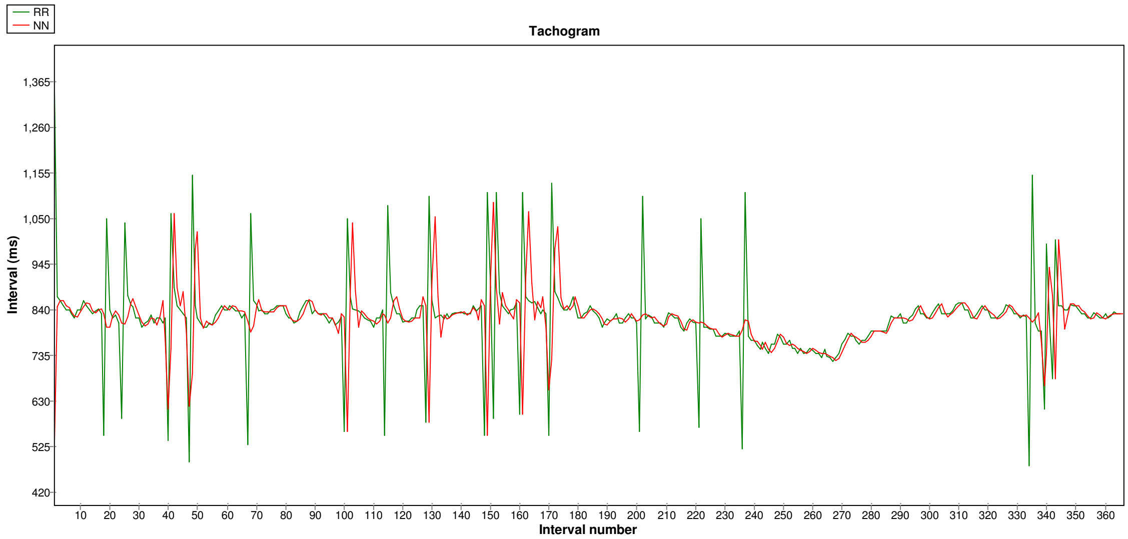

HRV parameters	NN	RR
TP (ms2)	3646	4047
VLF (ms2)	543	538
LF (ms2)	541	381
HF (ms2)	2562	3128
LF/HF	0.21	0.12
LF normalized	17.45	10.85
HF normalized	82.55	89.15
VLF peak (mHz)	7	7
LF peak (mHz)	100	100
HF peak (mHz)	299	383

HRV spectral settings	
Spectrum of Intervals (SOI)	
Frequency resolution (mHz)	3
VLF lower boundary (mHz)	3
VLF upper boundary (mHz)	40
LF upper boundary (mHz)	150
HF upper boundary (mHz)	400
Smoothing factor	1
Tapering	Hann
Fourier transform	DFT
Sample frequency (Hz)	1.22
Interval correction	Annotation
Interval threshold (%)	10
