# Supplementary material for: The effects of a 3-day mountain bike cycling race on the autonomic nervous system (ANS) and heart rate variability in amateur cyclists: a prospective quantitative research design
Source: BMC Sports Sci Med Rehabil. 2023 Jan 2;15:2. doi: 10.1186/s13102-022-00614-y (PMC9808932; doi:10.1186/s13102-022-00614-y)
Supplement: Supplementary file 1 — Additional file 1. Individual data of Participants. [file 13102_2022_614_MOESM1_ESM.zip › Individual data of Participants/HRV Data/015/ECG_015_20180501073845_.PDF]

Anton Swart Biokinetic Rehabilitation Practice

Name:	016 016	Recorded:	01/05/2018 07:38:45	UNCONFIRMED INTERPRETATION - MD SHOULD REVIEW
Number:	016	Recorded by:	Mr. Anton Swart	
Gender:	Female	Referring physician:		
Birthdate:	26/11/1970 47 years	Ordering physician:		
		Attending physician:		
		Location:	Anton Swart Biokinetic Rehabilitation Practi	
		Comment:		

P / PQ:	103 ms / 145 ms
QRS:	77 ms
QT / QTc / QTd:	432 ms / 428 ms / -
P/QRS/T axis:	55° / 90° / 73°
Heartrate:	58 bpm

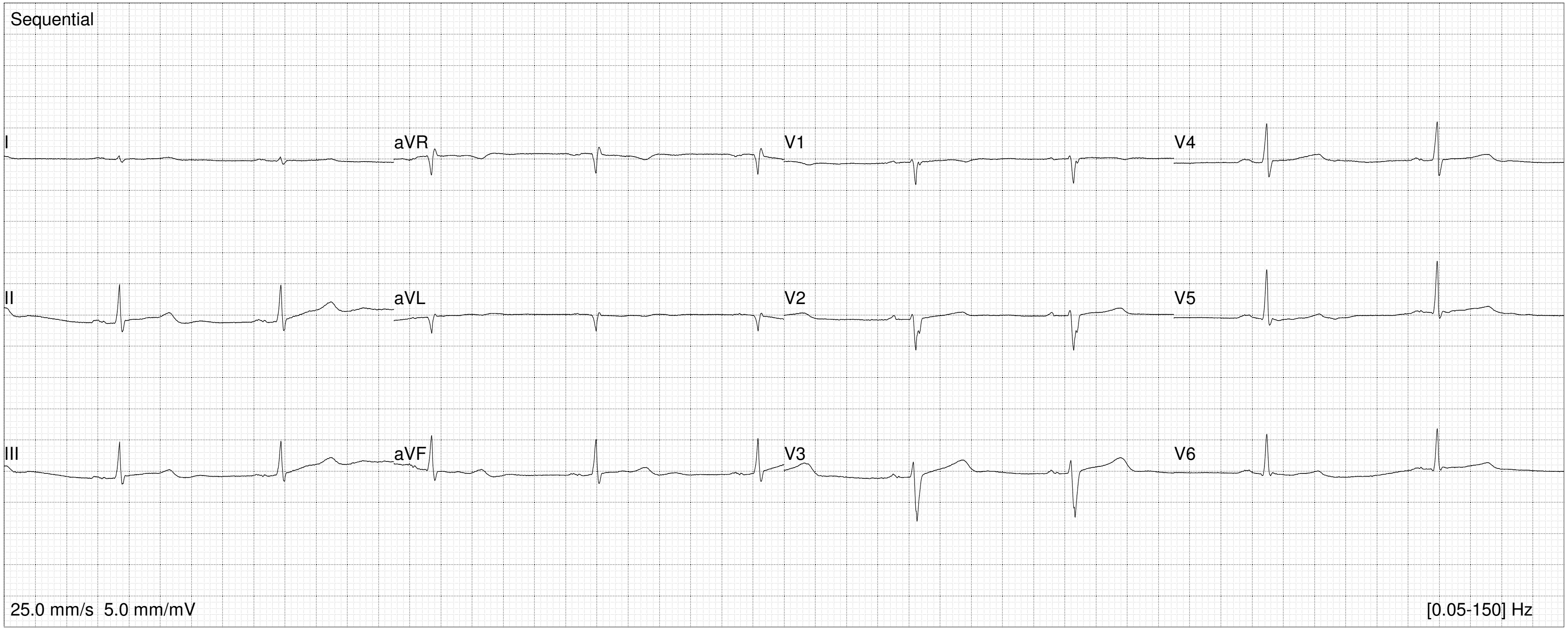

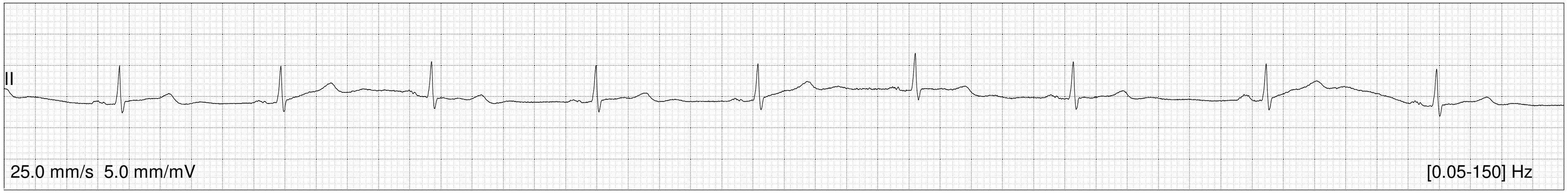

Anton Swart Biokinetic Rehabilitation Practice

Name:	016 016	Recorded:	01/05/2018 07:38:45	UNCONFIRMED INTERPRETATION - MD SHOULD REVIEW
Number:	016	Recorded by:	Mr. Anton Swart	
Gender:	Female	Referring physician:		
Birthdate:	26/11/1970 47 years	Location:	Anton Swart Biokinetic Rehabilitation Practice	
		Ordering physician:		
		Attending physician:		
		Comment:		

P / PQ:	103 ms / 145 ms
QRS:	77 ms
QT / QTc / QTd:	432 ms / 428 ms / -
P/QRS/T axis:	55° / 90° / 73°
Heartrate:	58 bpm

Beats

Total:	285
Normal:	285
Other:	0

RR

Minimum	890 ms
Maximum	1253 ms
Mean:	1046 ms
SD:	53 ms

R-R Trend

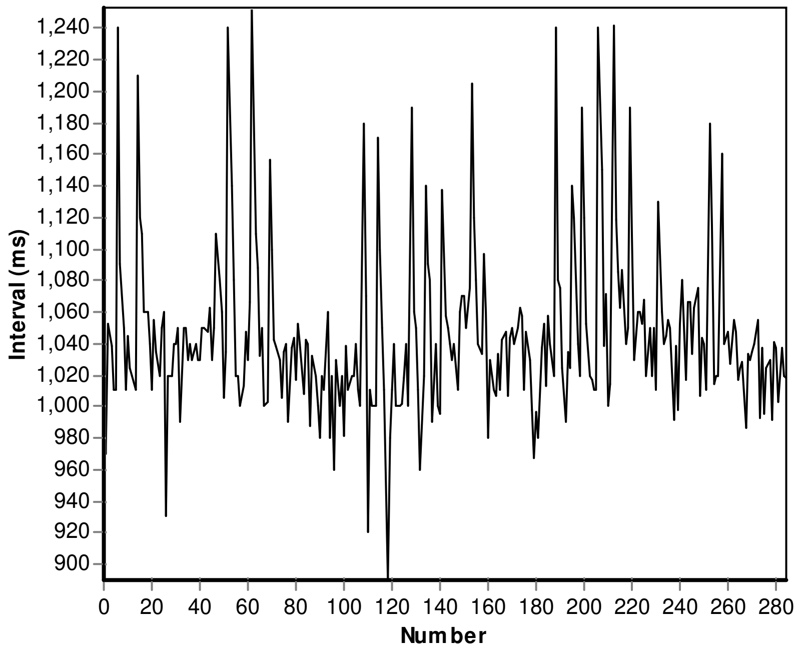

R-R Histogram

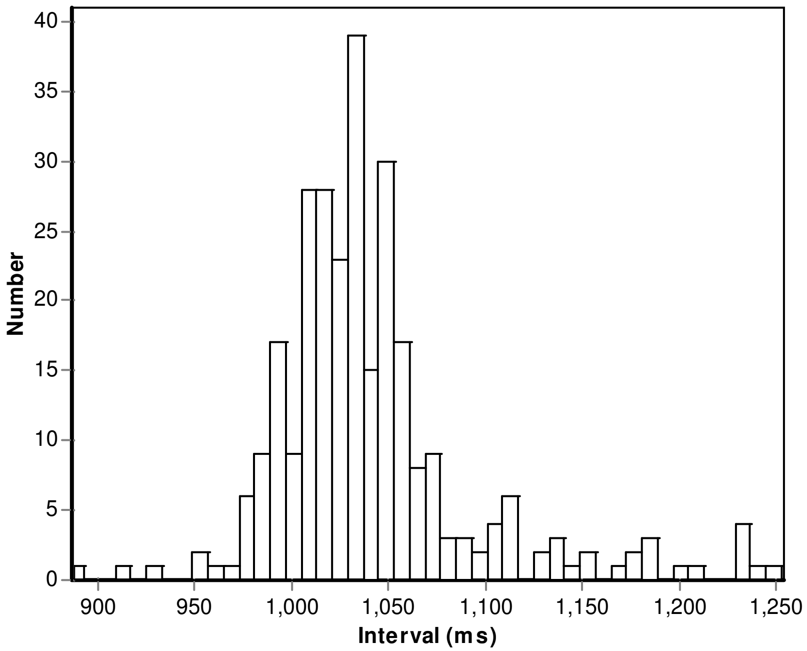

Cardio Control NV. User: Anton

Heart Rate Variability: Time Domain Analysis

**Name:**	016, 016	**Birthdate:**	26/11/1970
**Number:**	016	**Recorded:**	01/05/2018 07:38:45
**Gender:**	Female		

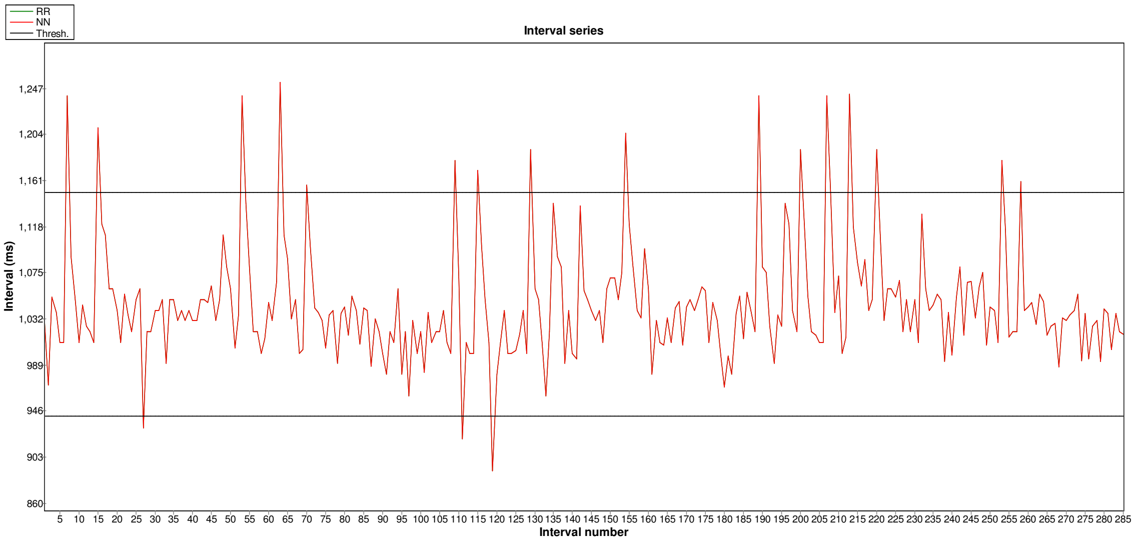

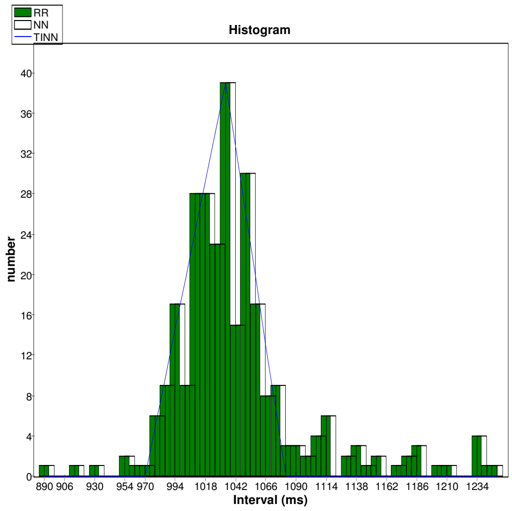

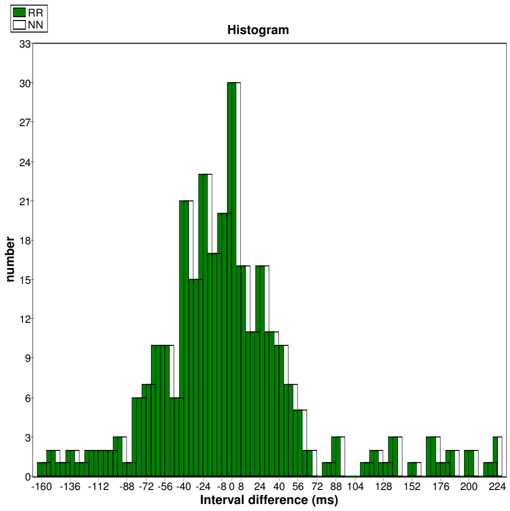

Binsize (ms) = 8

**HRV parameters**	**NN**	**RR**
SDNN (ms)	53	53
Triangular Interpolation (ms)	112	112
Triangular Index	7.31	7.31

**HRV parameters**	**NN**	**RR**
SDSD (ms)	65	65
RMSSD (ms)	65	65
NN50	75	75
NN50(1)	43	43
NN50(2)	32	32
pNN50	0.26	0.26
pNN50(1)	0.15	0.15
pNN50(2)	0.11	0.11
Logarithmic Index	0.14	0.14
SD(Logarithmic Index)	0.02	0.02

**Interval statistics**	**NN**	**RR**
Number	285	285
Minimum (ms)	890	890
Maximum (ms)	1253	1253
Range (ms)	363	363
Avg (ms)	1046	1046
SD (ms)	53	53
AvgDev (ms)	36	36
p5 (ms)	988	988
p50 (ms)	1040	1040
p95 (ms)	1170	1170
Skewness	1.54	1.54
Kurtosis	6.69	6.69

**Interval statistics**	**NN**	**RR**
Number	284	284
Minimum (ms)	-160	-160
Maximum (ms)	230	230
Range (ms)	390	390
Avg (ms)	-0	-0
SD (ms)	65	65
AvgDev (ms)	44	44
p5 (ms)	-90	-90
p50 (ms)	-5	-5
p95 (ms)	141	141
Skewness	1.05	1.05
Kurtosis	5.56	5.56

Cardio Control NV. User: Anton

Heart Rate Variability: Frequency Domain Analysis

**Name:**	016, 016	**Birthdate:**	26/11/1970
**Number:**	016	**Recorded:**	01/05/2018 07:38:45
**Gender:**	Female		

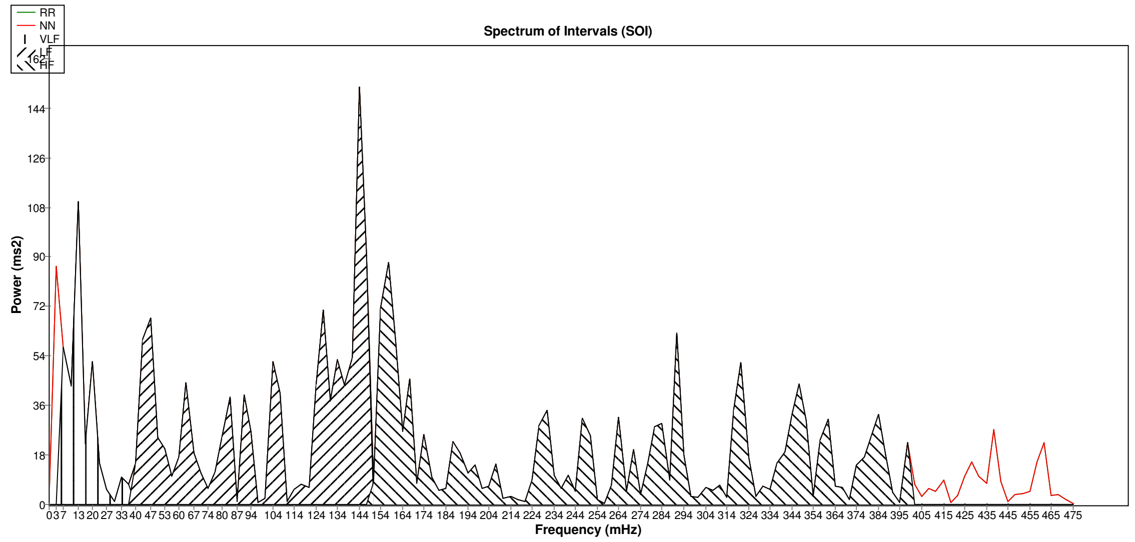

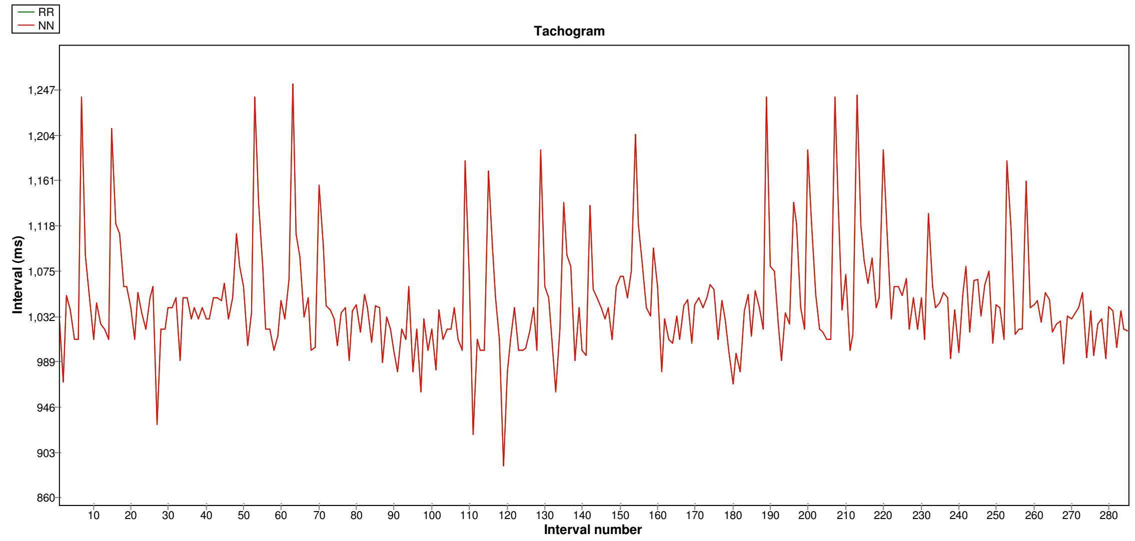

**HRV parameters**	**NN**	**RR**
TP (ms2)	2760	2760
VLF (ms2)	323	323
LF (ms2)	1097	1097
HF (ms2)	1340	1340
LF/HF	0.82	0.82
LF normalized	45.01	45.01
HF normalized	54.99	54.99
VLF peak (mHz)	13	13
LF peak (mHz)	144	144
HF peak (mHz)	157	157

**HRV spectral settings**	
Spectrum of Intervals (SOI)	
Frequency resolution (mHz)	3
VLF lower boundary (mHz)	3
VLF upper boundary (mHz)	40
LF upper boundary (mHz)	150
HF upper boundary (mHz)	400
Smoothing factor	1
Tapering	Hann
Fourier transform	DFT
Sample frequency (Hz)	0.96
Interval correction	Annotation
Interval threshold (%)	10
