# Supplementary material for: The effects of a 3-day mountain bike cycling race on the autonomic nervous system (ANS) and heart rate variability in amateur cyclists: a prospective quantitative research design
Source: BMC Sports Sci Med Rehabil. 2023 Jan 2;15:2. doi: 10.1186/s13102-022-00614-y (PMC9808932; doi:10.1186/s13102-022-00614-y)
Supplement: Supplementary file 1 — Additional file 1. Individual data of Participants. [file 13102_2022_614_MOESM1_ESM.zip › Individual data of Participants/HRV Data/015/ECG_015_20180503182815_.PDF]

Anton Swart Biokinetic Rehabilitation Practice

Name: 016 016
Number: 016
Gender: Female
Birthdate: 26/11/1970 47 years

P / PQ: 118 ms / 150 ms
QRS: 82 ms
QT / QTc / QTd: 388 ms / 411 ms / -
P/QRS/T axis: 81° / 91° / 77°
Heartrate: 73 bpm

Recorded: 03/05/2018 18:28:15
Recorded by: Mr. Anton Swart
Referring physician:
Ordering physician:
Attending physician:
Location: Anton Swart Biokinetic Rehabilitation Practi
Comment:

UNCONFIRMED INTERPRETATION - MD SHOULD REVIEW

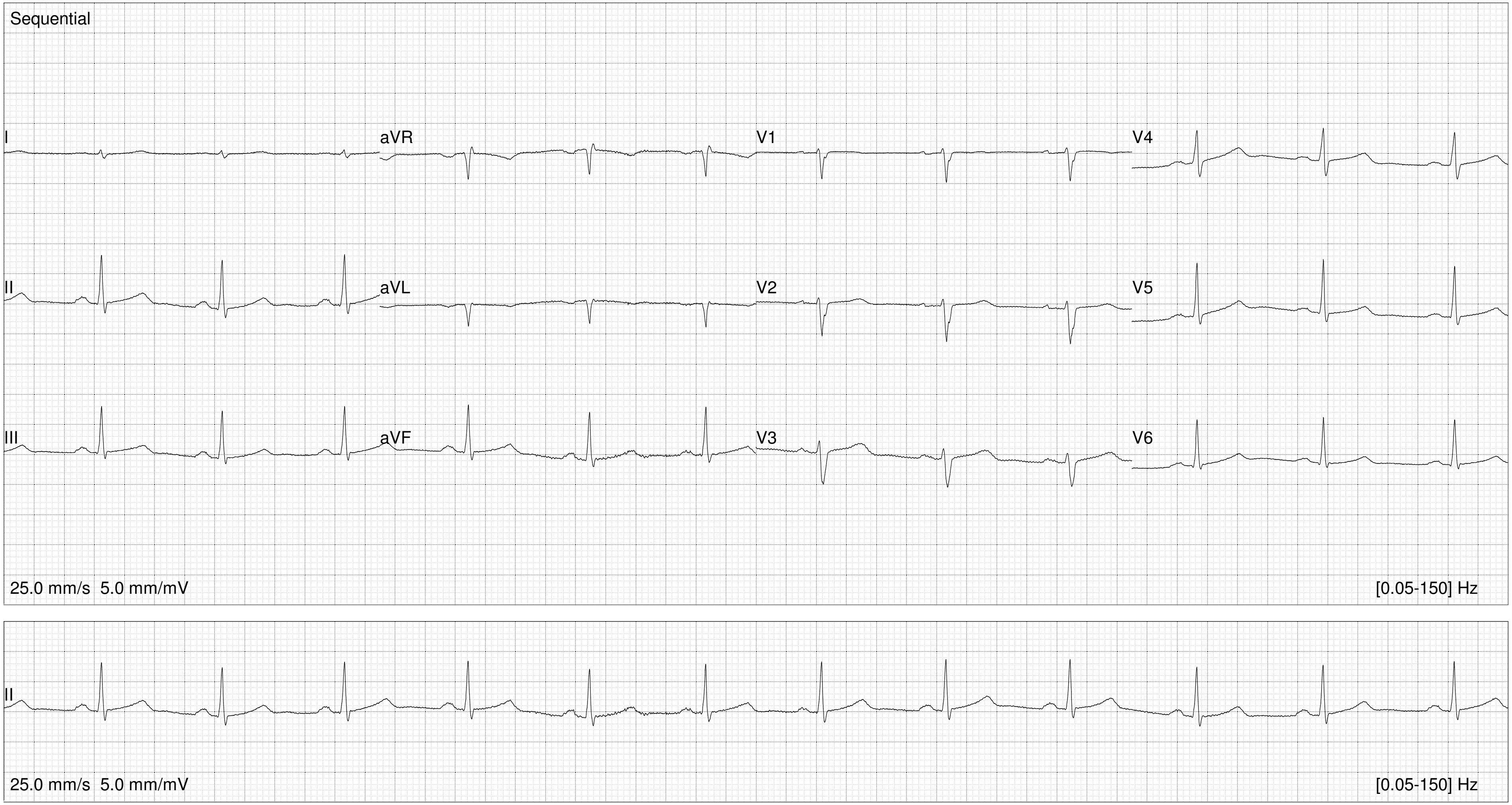

Anton Swart Biokinetic Rehabilitation Practice

Name:	016 016	Recorded:	03/05/2018 18:28:15	UNCONFIRMED INTERPRETATION - MD SHOULD REVIEW
Number:	016	Recorded by:	Mr. Anton Swart	
Gender:	Female	Referring physician:		
Birthdate:	26/11/1970 47 years	Location:	Anton Swart Biokinetic Rehabilitation Practice	
		Ordering physician:		
		Attending physician:		
		Comment:		

P / PQ:	118 ms / 150 ms
QRS:	82 ms
QT / QTc / QTd:	388 ms / 411 ms / -
P/QRS/T axis:	81° / 91° / 77°
Heartrate:	73 bpm

Beats

Total:	366
Normal:	366
Other:	0

RR

Minimum	710 ms
Maximum	892 ms
Mean:	817 ms
SD:	27 ms

R-R Trend

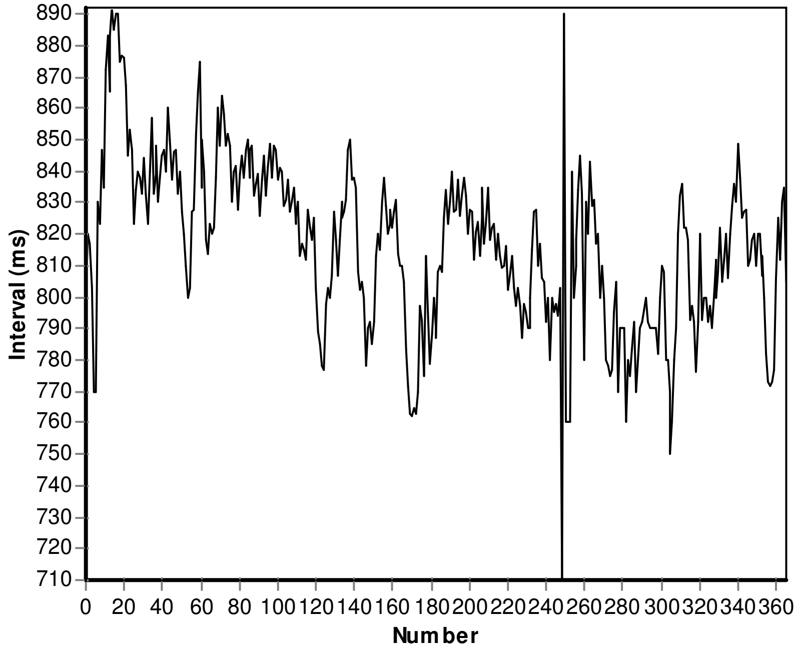

R-R Histogram

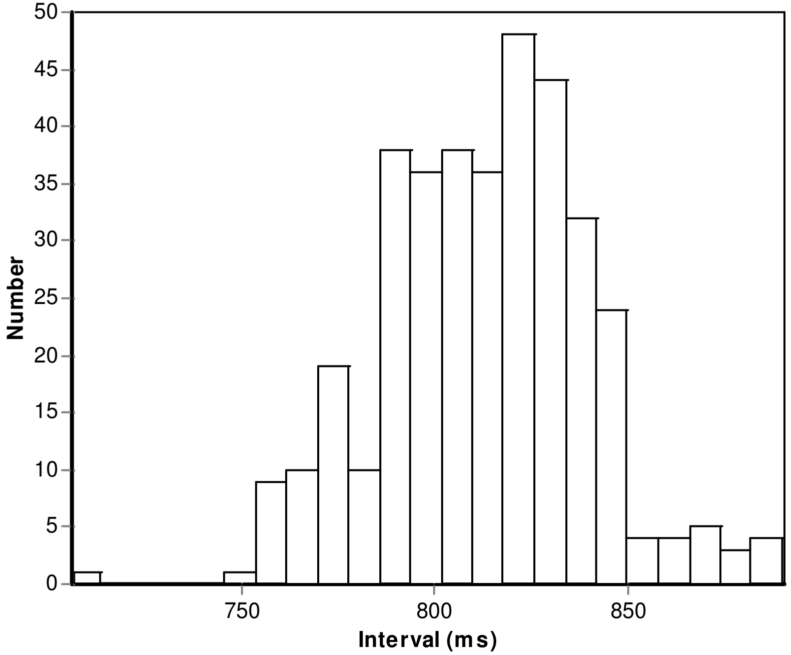

Heart Rate Variability: Time Domain Analysis

**Name:**	016, 016	**Birthdate:**	26/11/1970
**Number:**	016	**Recorded:**	03/05/2018 18:28:15
**Gender:**	Female		

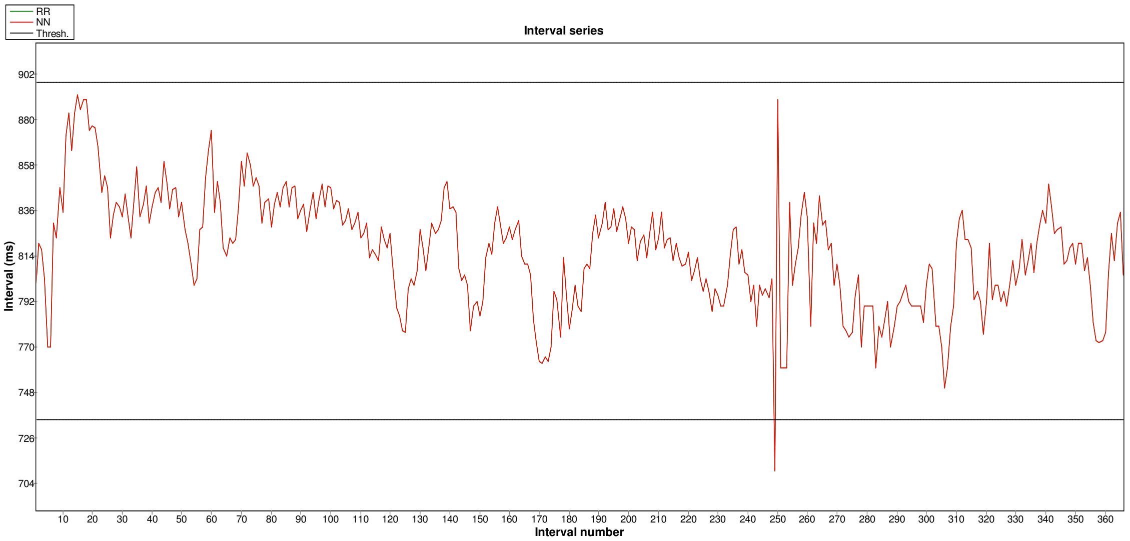

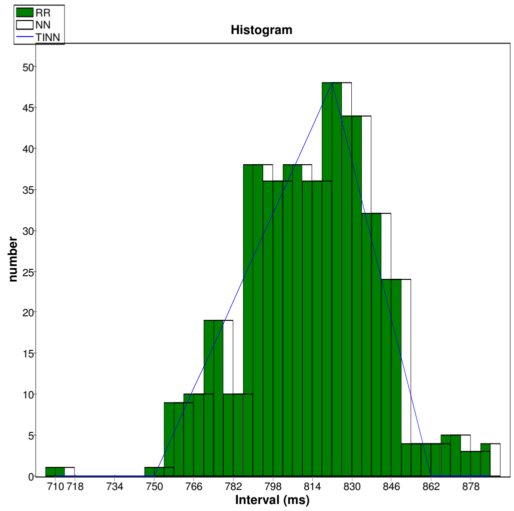

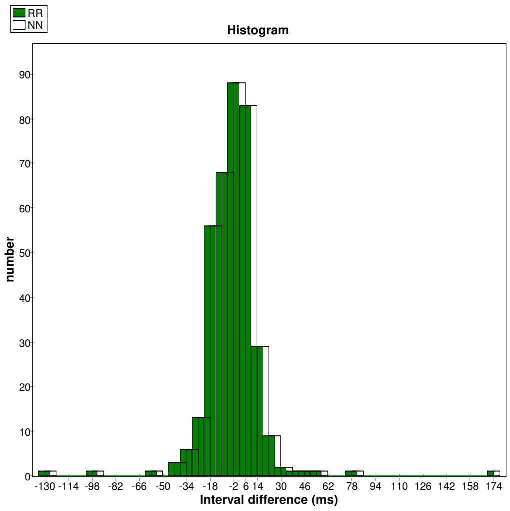

Binsize (ms) = 8

HRV parameters	NN	RR
SDNN (ms)	27	27
Triangular Interpolation (ms)	112	112
Triangular Index	7.63	7.63

HRV parameters	NN	RR
SDSD (ms)	19	19
RMSSD (ms)	19	19
NN50	6	6
NN50(1)	3	3
NN50(2)	3	3
pNN50	0.02	0.02
pNN50(1)	0.01	0.01
pNN50(2)	0.01	0.01
Logarithmic Index	0.51	0.51
SD(Logarithmic Index)	0.07	0.07

Interval statistics	NN	RR
Number	366	366
Minimum (ms)	710	710
Maximum (ms)	892	892
Range (ms)	182	182
Avg (ms)	817	817
SD (ms)	27	27
AvgDev (ms)	21	21
p5 (ms)	772	772
p50 (ms)	820	820
p95 (ms)	859	859
Skewness	-0.05	-0.05
Kurtosis	3.47	3.47

Interval statistics	NN	RR
Number	365	365
Minimum (ms)	-130	-130
Maximum (ms)	180	180
Range (ms)	310	310
Avg (ms)	0	0
SD (ms)	19	19
AvgDev (ms)	12	12
p5 (ms)	-22	-22
p50 (ms)	1	1
p95 (ms)	21	21
Skewness	1.38	1.38
Kurtosis	32.72	32.72

Heart Rate Variability: Frequency Domain Analysis

**Name:**	016, 016	**Birthdate:**	26/11/1970
**Number:**	016	**Recorded:**	03/05/2018 18:28:15
**Gender:**	Female		

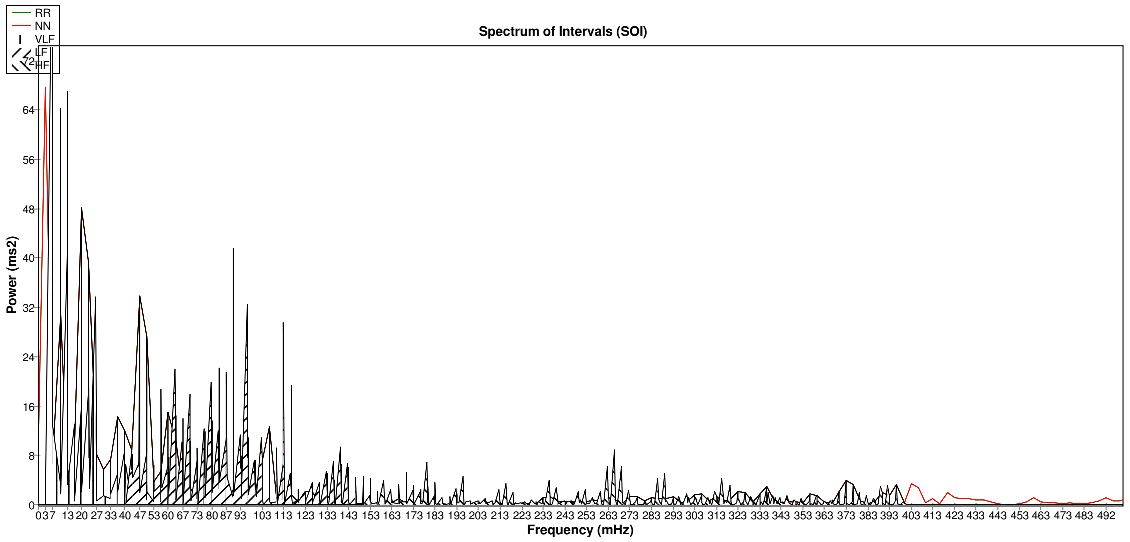

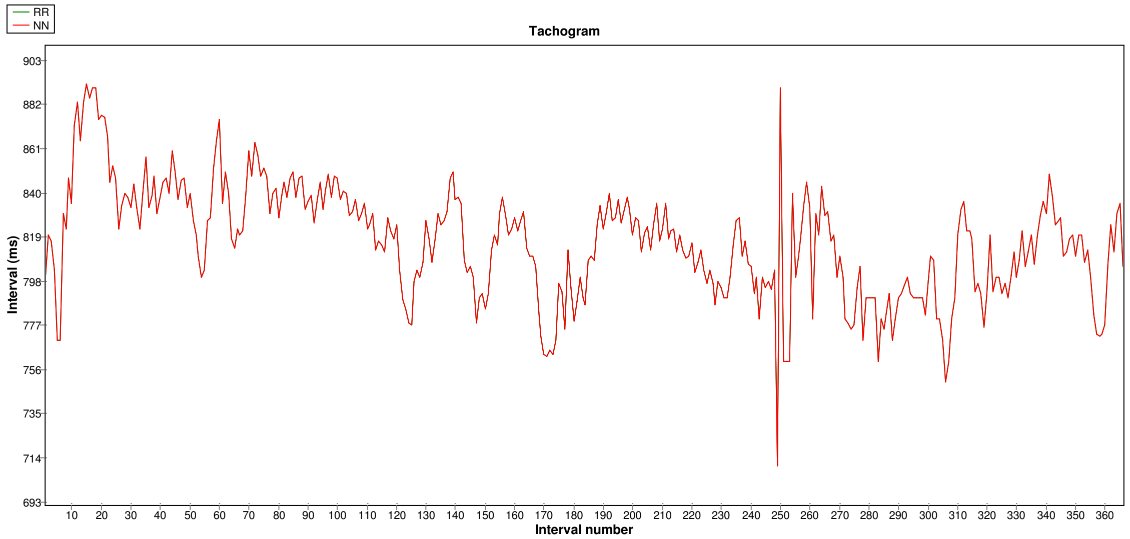

HRV parameters	NN	RR
TP (ms2)	401	401
VLF (ms2)	186	186
LF (ms2)	153	153
HF (ms2)	62	62
LF/HF	2.46	2.46
LF normalized	71.13	71.13
HF normalized	28.87	28.87
VLF peak (mHz)	20	20
LF peak (mHz)	47	47
HF peak (mHz)	373	373

HRV spectral settings	
Spectrum of Intervals (SOI)	
Frequency resolution (mHz)	3
VLF lower boundary (mHz)	3
VLF upper boundary (mHz)	40
LF upper boundary (mHz)	150
HF upper boundary (mHz)	400
Smoothing factor	1
Tapering	Hann
Fourier transform	DFT
Sample frequency (Hz)	1.22
Interval correction	Annotation
Interval threshold (%)	10
