# Supplementary material for: The effects of a 3-day mountain bike cycling race on the autonomic nervous system (ANS) and heart rate variability in amateur cyclists: a prospective quantitative research design
Source: BMC Sports Sci Med Rehabil. 2023 Jan 2;15:2. doi: 10.1186/s13102-022-00614-y (PMC9808932; doi:10.1186/s13102-022-00614-y)
Supplement: Supplementary file 1 — Additional file 1. Individual data of Participants. [file 13102_2022_614_MOESM1_ESM.zip › Individual data of Participants/HRV Data/015/ECG_015_20180504135101_.PDF]

Anton Swart Biokinetic Rehabilitation Practice

Name:	016 016	Recorded:	04/05/2018 13:51:01	UNCONFIRMED INTERPRETATION - MD SHOULD REVIEW
Number:	016	Recorded by:	Mr. Anton Swart	
Gender:	Female	Referring physician:		
Birthdate:	26/11/1970 47 years	Ordering physician:		
		Attending physician:		
P / PQ:	115 ms / 145 ms	Location:	Anton Swart Biokinetic Rehabilitation Practi	
QRS:	80 ms	Comment:		
QT / QTc / QTd:	392 ms / 425 ms / -			
P/QRS/T axis:	70° / 88° / 75°			
Heartrate:	79 bpm			

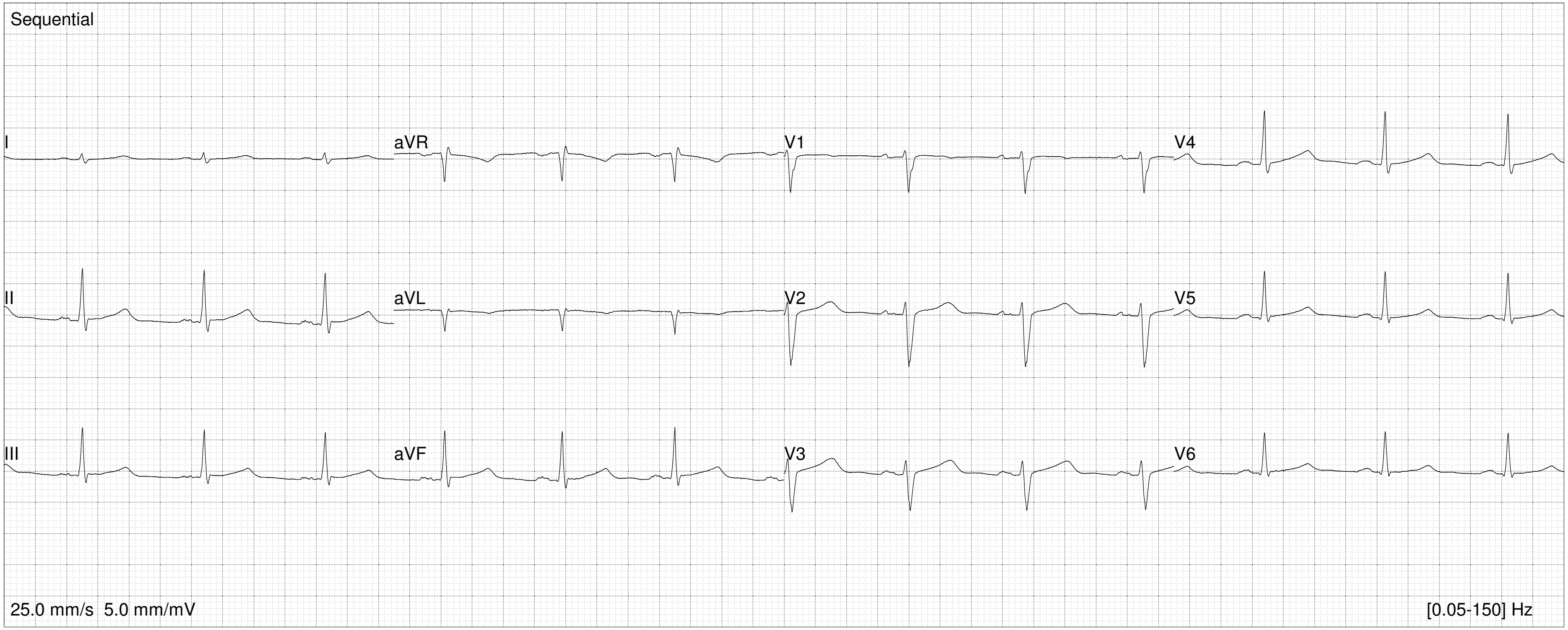

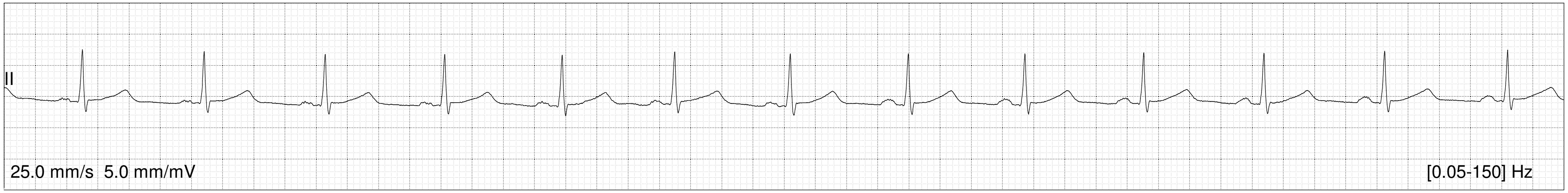

Anton Swart Biokinetic Rehabilitation Practice

Name:	016 016	Recorded:	04/05/2018 13:51:01	UNCONFIRMED INTERPRETATION - MD SHOULD REVIEW
Number:	016	Recorded by:	Mr. Anton Swart	
Gender:	Female	Referring physician:		
Birthdate:	26/11/1970 47 years	Location:	Anton Swart Biokinetic Rehabilitation Practice	
P / PQ:	115 ms / 145 ms	Ordering physician:		
QRS:	80 ms	Attending physician:		
QT / QTc / QTd:	392 ms / 425 ms / -	Comment:		
P/QRS/T axis:	70° / 88° / 75°			
Heartrate:	79 bpm			

**Beats**		**RR**	
Total:	395	Minimum	698 ms
Normal:	395	Maximum	804 ms
Other:	0	Mean:	757 ms
		SD:	22 ms

R-R Trend

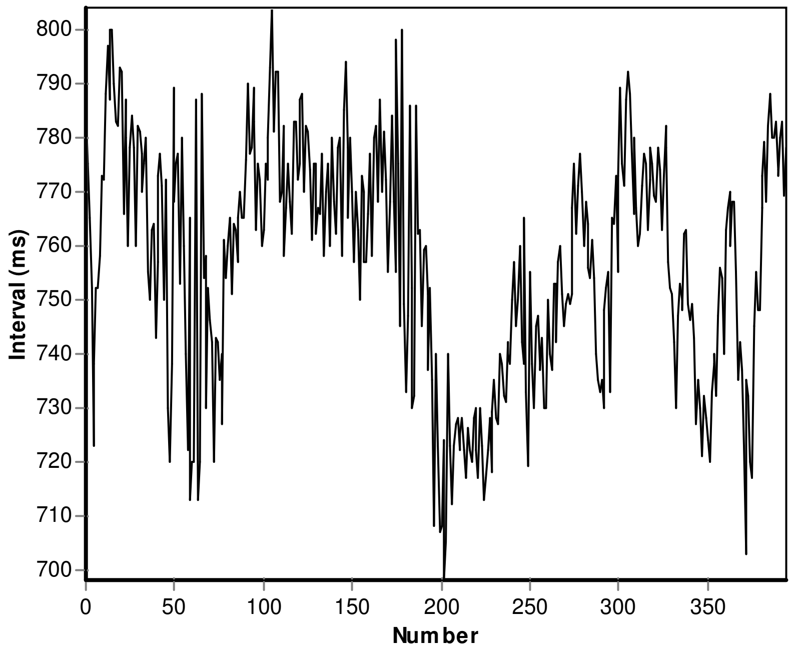

R-R Histogram

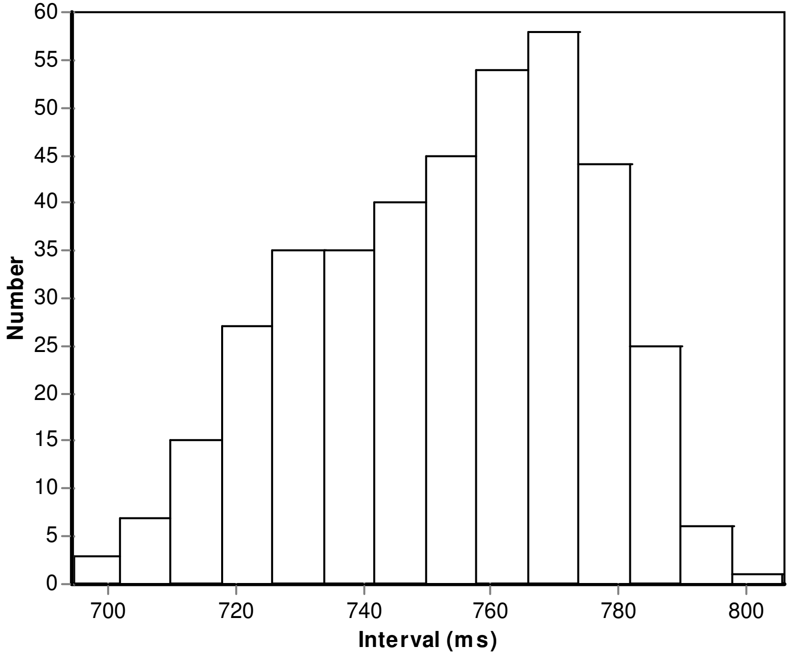

Heart Rate Variability: Time Domain Analysis

**Name:**	016, 016	**Birthdate:**	26/11/1970
**Number:**	016	**Recorded:**	04/05/2018 13:51:01
**Gender:**	Female		

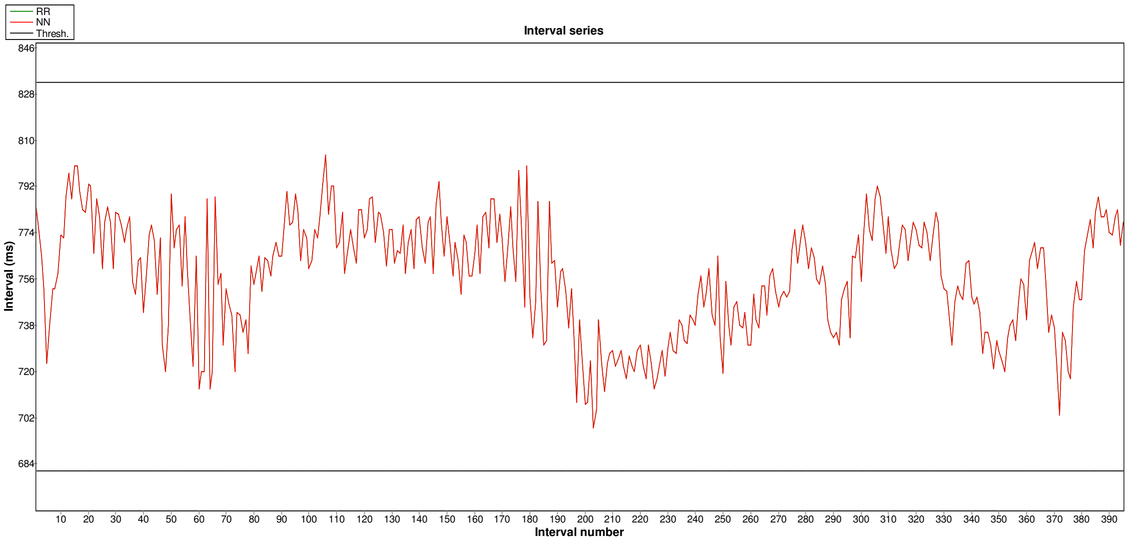

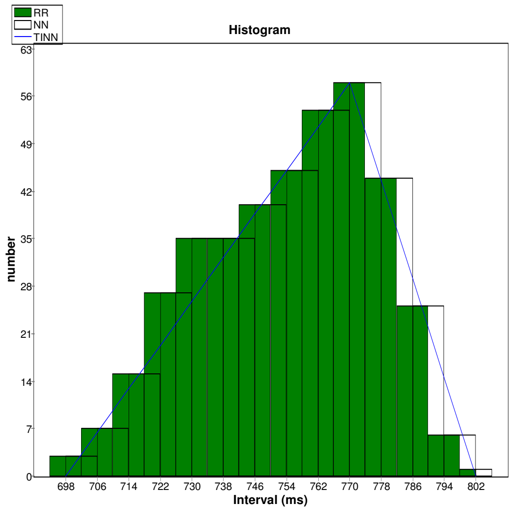

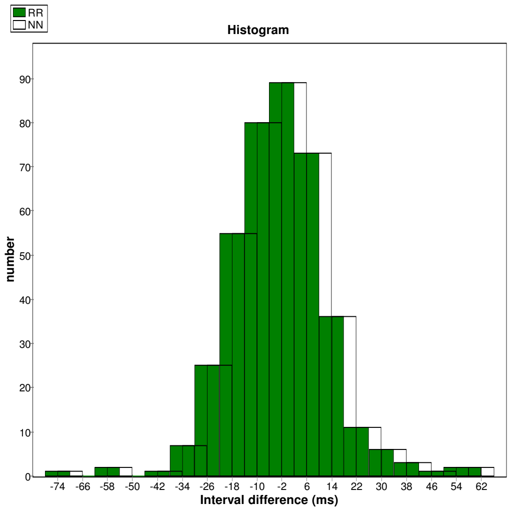

Binsize (ms) = 8

**HRV parameters**	**NN**	**RR**
SDNN (ms)	22	22
Triangular Interpolation (ms)	104	104
Triangular Index	6.81	6.81

**HRV parameters**	**NN**	**RR**
SDSD (ms)	16	16
RMSSD (ms)	16	16
NN50	8	8
NN50(1)	3	3
NN50(2)	5	5
pNN50	0.02	0.02
pNN50(1)	0.01	0.01
pNN50(2)	0.01	0.01
Logarithmic Index	0.66	0.66
SD(Logarithmic Index)	0.05	0.05

**Interval statistics**	**NN**	**RR**
Number	395	395
Minimum (ms)	698	698
Maximum (ms)	804	804
Range (ms)	106	106
Avg (ms)	757	757
SD (ms)	22	22
AvgDev (ms)	18	18
p5 (ms)	720	720
p50 (ms)	760	760
p95 (ms)	788	788
Skewness	-0.31	-0.31
Kurtosis	2.28	2.28

**Interval statistics**	**NN**	**RR**
Number	394	394
Minimum (ms)	-74	-74
Maximum (ms)	68	68
Range (ms)	142	142
Avg (ms)	-0	-0
SD (ms)	16	16
AvgDev (ms)	12	12
p5 (ms)	-23	-23
p50 (ms)	0	0
p95 (ms)	23	23
Skewness	0.31	0.31
Kurtosis	6.12	6.12

Cardio Control NV. User: Anton

Heart Rate Variability: Frequency Domain Analysis

**Name:**	016, 016	**Birthdate:**	26/11/1970
**Number:**	016	**Recorded:**	04/05/2018 13:51:01
**Gender:**	Female		

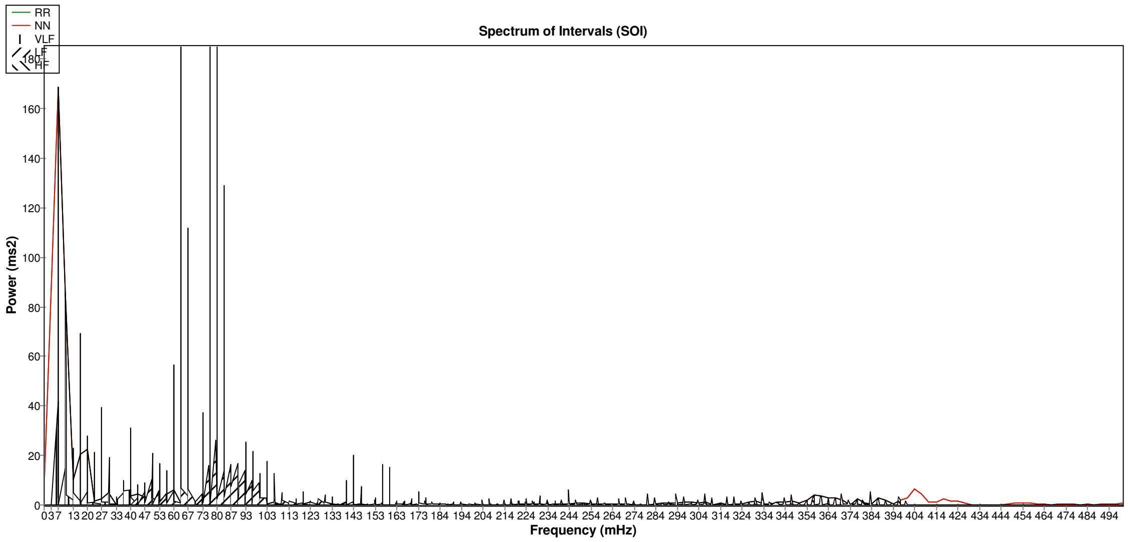

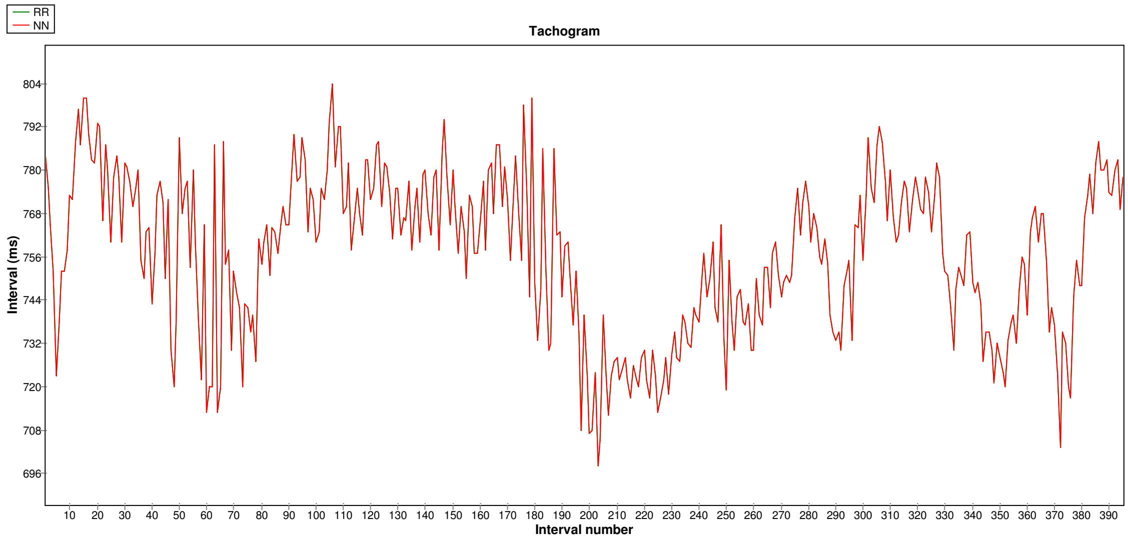

**HRV parameters**	**NN**	**RR**
TP (ms2)	426	426
VLF (ms2)	323	323
LF (ms2)	47	47
HF (ms2)	57	57
LF/HF	0.83	0.83
LF normalized	45.35	45.35
HF normalized	54.65	54.65
VLF peak (mHz)	7	7
LF peak (mHz)	60	60
HF peak (mHz)	357	357

**HRV spectral settings**	
Spectrum of Intervals (SOI)	
Frequency resolution (mHz)	3
VLF lower boundary (mHz)	3
VLF upper boundary (mHz)	40
LF upper boundary (mHz)	150
HF upper boundary (mHz)	400
Smoothing factor	1
Tapering	Hann
Fourier transform	DFT
Sample frequency (Hz)	1.32
Interval correction	Annotation
Interval threshold (%)	10
