# Supplementary material for: The effects of a 3-day mountain bike cycling race on the autonomic nervous system (ANS) and heart rate variability in amateur cyclists: a prospective quantitative research design
Source: BMC Sports Sci Med Rehabil. 2023 Jan 2;15:2. doi: 10.1186/s13102-022-00614-y (PMC9808932; doi:10.1186/s13102-022-00614-y)
Supplement: Supplementary file 1 — Additional file 1. Individual data of Participants. [file 13102_2022_614_MOESM1_ESM.zip › Individual data of Participants/HRV Data/015/ECG_015_20180505131435_.PDF]

Anton Swart Biokinetic Rehabilitation Practice

Name:	016 016	Recorded:	05/05/2018 13:14:35	UNCONFIRMED INTERPRETATION - MD SHOULD REVIEW
Number:	016	Recorded by:	Mr. Anton Swart	
Gender:	Female	Referring physician:		
Birthdate:	26/11/1970 47 years	Ordering physician:		
		Attending physician:		
P / PQ:	113 ms / 142 ms	Location:	Anton Swart Biokinetic Rehabilitation Practi	
QRS:	80 ms	Comment:		
QT / QTc / QTd:	391 ms / 424 ms / -			
P/QRS/T axis:	74° / 91° / 76°			
Heartrate:	79 bpm			

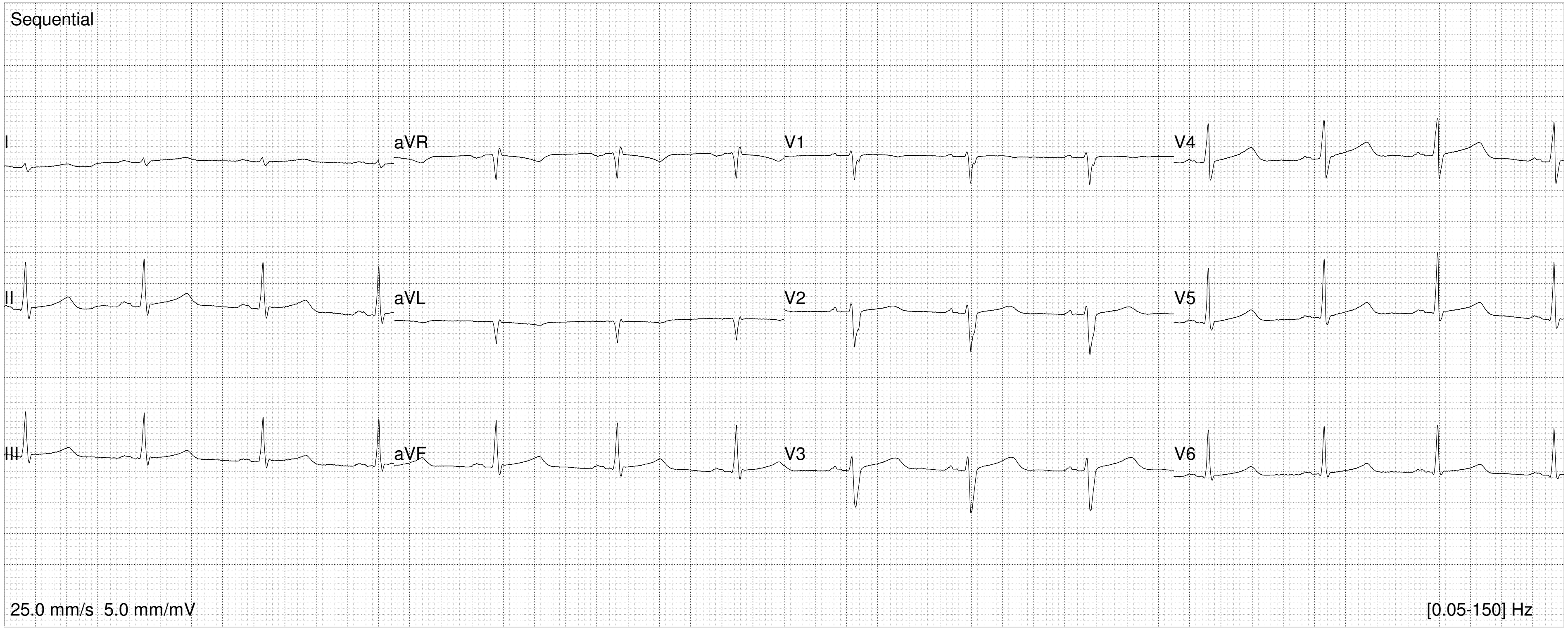

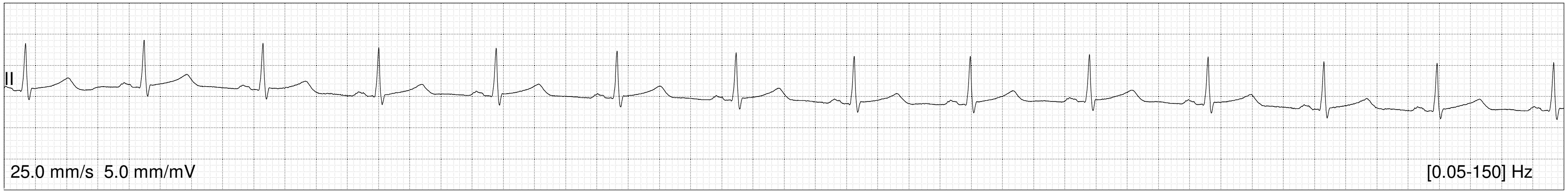

Anton Swart Biokinetic Rehabilitation Practice

Name:	016 016	Recorded:	05/05/2018 13:14:35	UNCONFIRMED INTERPRETATION - MD SHOULD REVIEW
Number:	016	Recorded by:	Mr. Anton Swart	
Gender:	Female	Referring physician:		
Birthdate:	26/11/1970 47 years	Location:	Anton Swart Biokinetic Rehabilitation Practice	
P / PQ:	113 ms / 142 ms	Ordering physician:		
QRS:	80 ms	Attending physician:		
QT / QTc / QTd:	391 ms / 424 ms / -	Comment:		
P/QRS/T axis:	74° / 91° / 76°			
Heartrate:	79 bpm			

Beats

Total:	393
Normal:	393
Other:	0

RR

Minimum	700 ms
Maximum	825 ms
Mean:	761 ms
SD:	21 ms

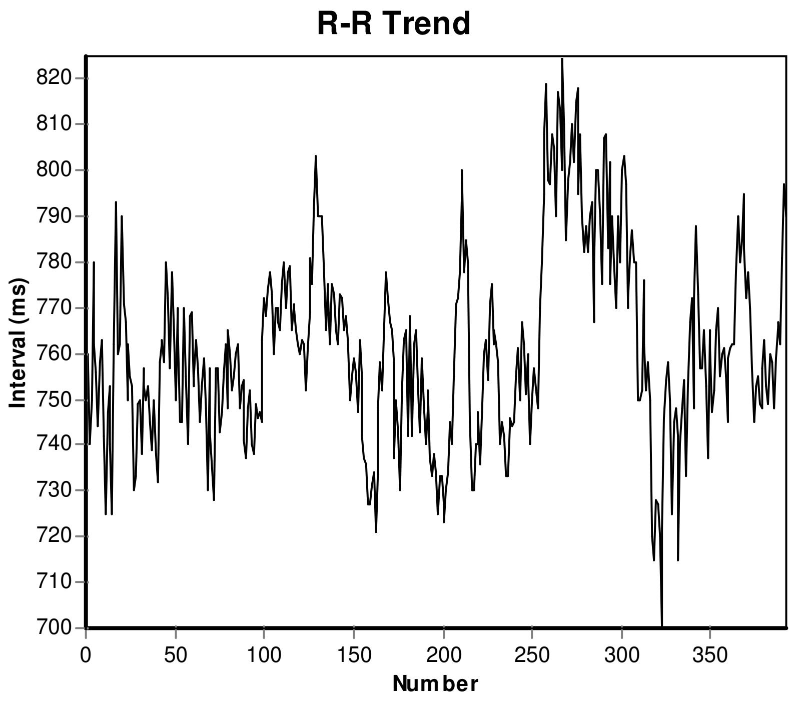

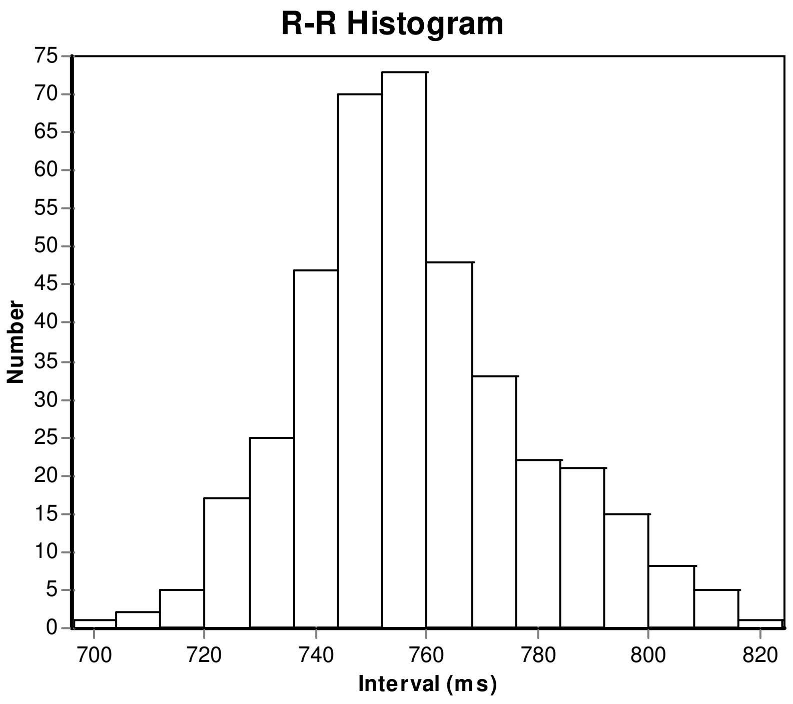

Cardio Control NV. User: Anton

Heart Rate Variability: Time Domain Analysis

**Name:**	016, 016	**Birthdate:**	26/11/1970
**Number:**	016	**Recorded:**	05/05/2018 13:14:35
**Gender:**	Female		

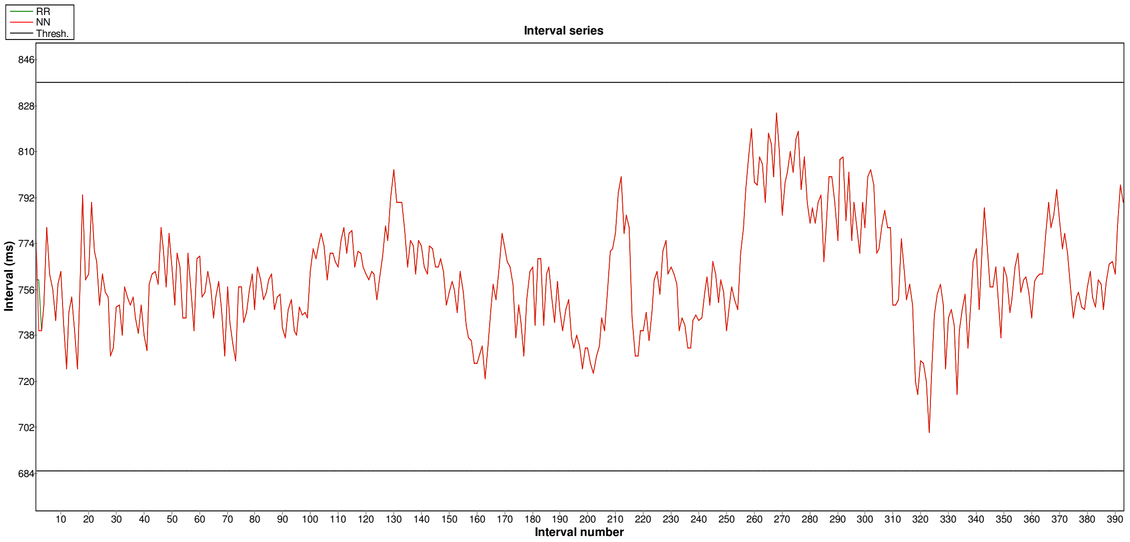

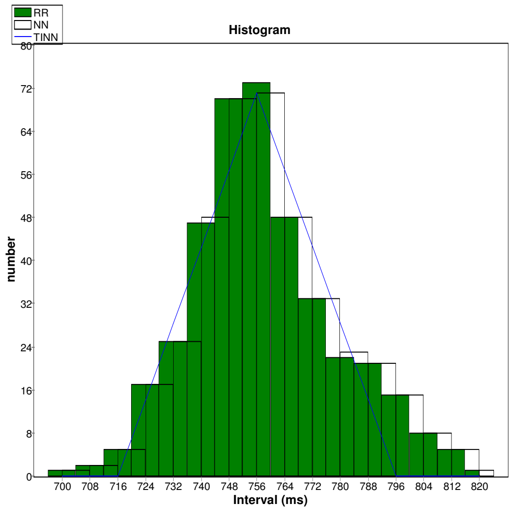

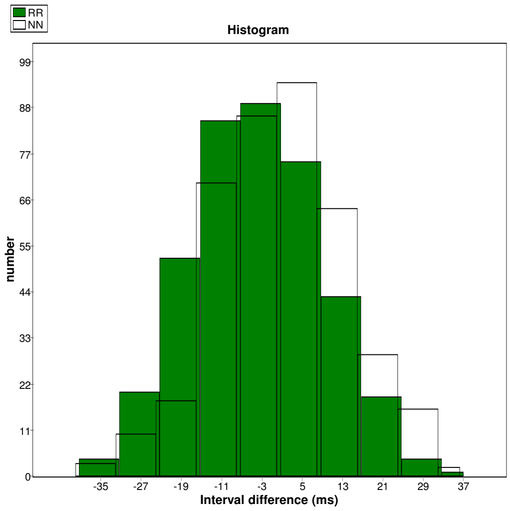

Binsize (ms) = 8

**HRV parameters**	**NN**	**RR**
SDNN (ms)	21	21
Triangular Interpolation (ms)	80	80
Triangular Index	5.54	5.38

**HRV parameters**	**NN**	**RR**
SDSD (ms)	13	13
RMSSD (ms)	13	13
NN50	0	0
NN50(1)	0	0
NN50(2)	0	0
pNN50	0.00	0.00
pNN50(1)	0.00	0.00
pNN50(2)	0.00	0.00
Logarithmic Index	1.06	0.91
SD(Logarithmic Index)	0.14	0.17

**Interval statistics**	**NN**	**RR**
Number	393	393
Minimum (ms)	700	700
Maximum (ms)	825	825
Range (ms)	125	125
Avg (ms)	761	761
SD (ms)	21	21
AvgDev (ms)	16	16
p5 (ms)	730	730
p50 (ms)	760	760
p95 (ms)	802	802
Skewness	0.43	0.44
Kurtosis	3.16	3.18

**Interval statistics**	**NN**	**RR**
Number	392	392
Minimum (ms)	-40	-35
Maximum (ms)	37	37
Range (ms)	77	72
Avg (ms)	0	0
SD (ms)	13	13
AvgDev (ms)	10	10
p5 (ms)	-21	-21
p50 (ms)	0	0
p95 (ms)	22	22
Skewness	0.04	0.10
Kurtosis	2.94	2.82

Cardio Control NV. User: Anton

Heart Rate Variability: Frequency Domain Analysis

**Name:**	016, 016	**Birthdate:**	26/11/1970
**Number:**	016	**Recorded:**	05/05/2018 13:14:35
**Gender:**	Female		

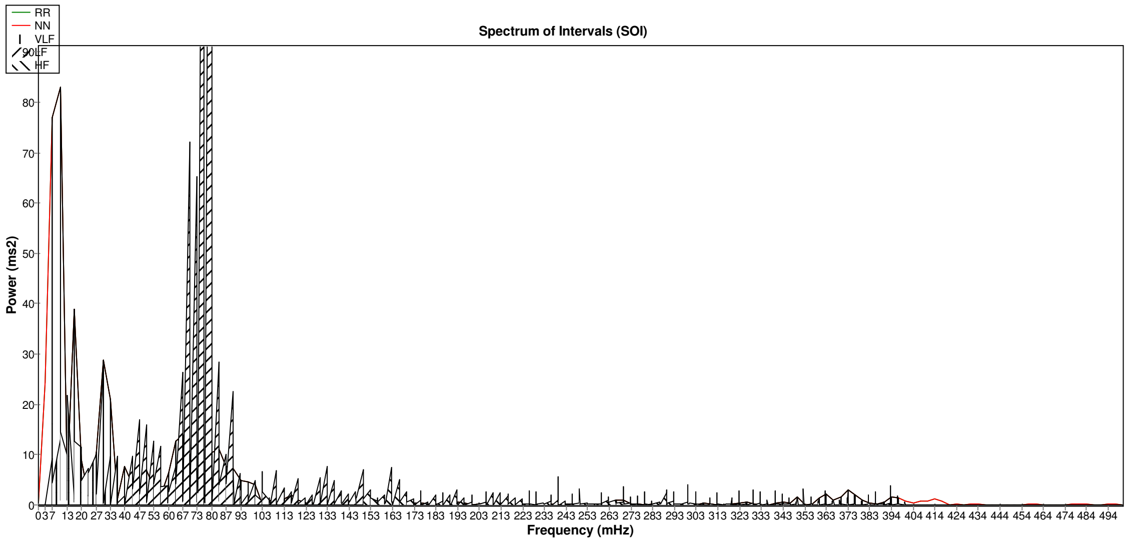

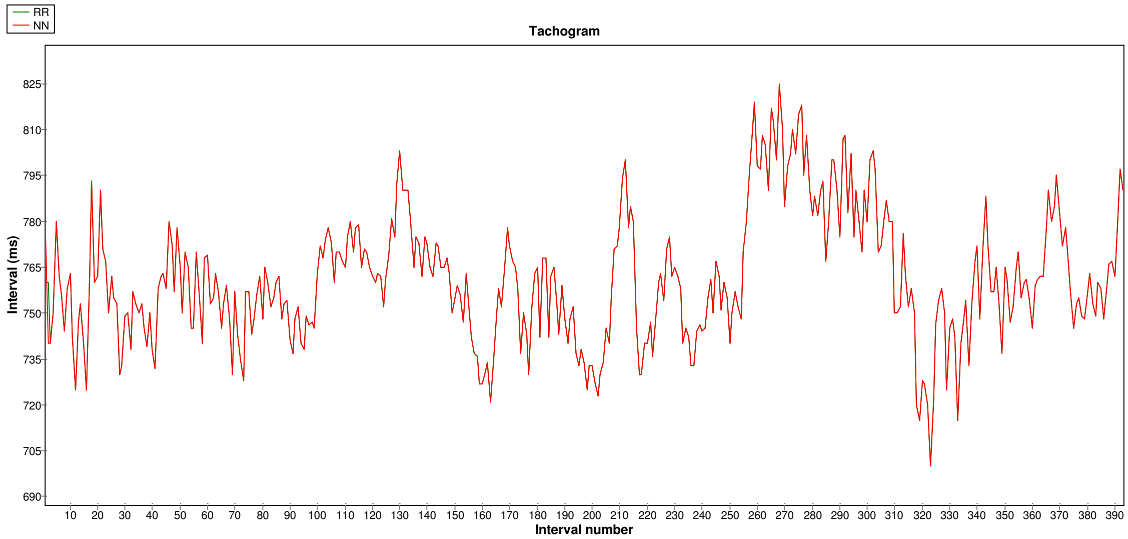

**HRV parameters**	**NN**	**RR**
TP (ms2)	449	449
VLF (ms2)	284	284
LF (ms2)	130	130
HF (ms2)	35	35
LF/HF	3.77	3.77
LF normalized	79.03	79.03
HF normalized	20.97	20.97
VLF peak (mHz)	10	10
LF peak (mHz)	67	67
HF peak (mHz)	373	373

**HRV spectral settings**	
Spectrum of Intervals (SOI)	
Frequency resolution (mHz)	3
VLF lower boundary (mHz)	3
VLF upper boundary (mHz)	40
LF upper boundary (mHz)	150
HF upper boundary (mHz)	400
Smoothing factor	1
Tapering	Hann
Fourier transform	DFT
Sample frequency (Hz)	1.31
Interval correction	Annotation
Interval threshold (%)	10
