# Supplementary material for: The effects of a 3-day mountain bike cycling race on the autonomic nervous system (ANS) and heart rate variability in amateur cyclists: a prospective quantitative research design
Source: BMC Sports Sci Med Rehabil. 2023 Jan 2;15:2. doi: 10.1186/s13102-022-00614-y (PMC9808932; doi:10.1186/s13102-022-00614-y)
Supplement: Supplementary file 1 — Additional file 1. Individual data of Participants. [file 13102_2022_614_MOESM1_ESM.zip › Individual data of Participants/HRV Data/015/ECG_015_20180506091836_.PDF]

Anton Swart Biokinetic Rehabilitation Practice

Name:	016 016	Recorded:	06/05/2018 09:18:36	UNCONFIRMED INTERPRETATION - MD SHOULD REVIEW
Number:	016	Recorded by:	Mr. Anton Swart	
Gender:	Female	Referring physician:		
Birthdate:	26/11/1970 47 years	Ordering physician:		
		Attending physician:		
P / PQ:	92 ms / 132 ms	Location:	Anton Swart Biokinetic Rehabilitation Practi	
QRS:	78 ms	Comment:		
QT / QTc / QTd:	451 ms / 448 ms / -			
P/QRS/T axis:	38° / 91° / 78°			
Heartrate:	58 bpm			

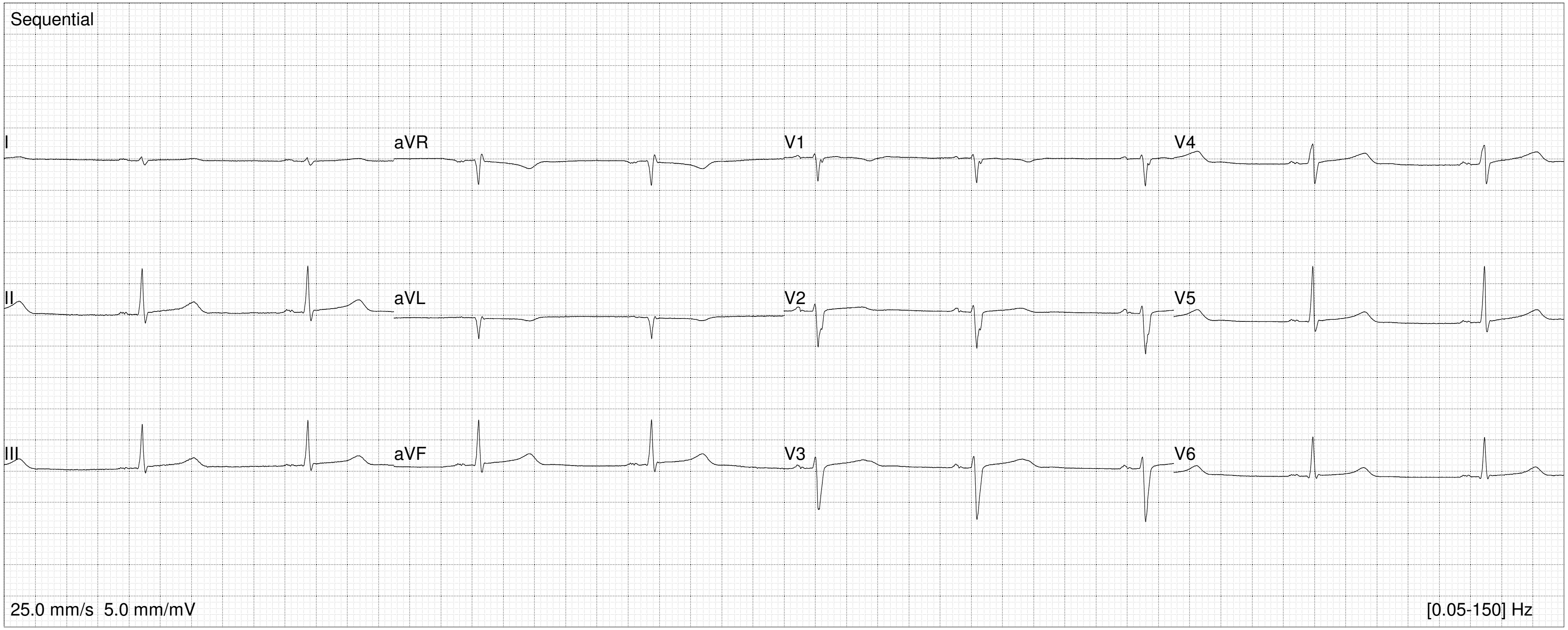

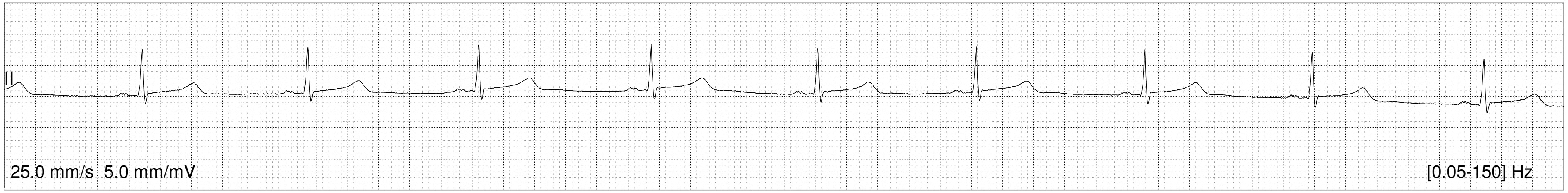

Anton Swart Biokinetic Rehabilitation Practice

Name:	016 016	Recorded:	06/05/2018 09:18:36	UNCONFIRMED INTERPRETATION - MD SHOULD REVIEW
Number:	016	Recorded by:	Mr. Anton Swart	
Gender:	Female	Referring physician:		
Birthdate:	26/11/1970 47 years	Location:	Anton Swart Biokinetic Rehabilitation Practice	
		Ordering physician:		
		Attending physician:		
		Comment:		

P / PQ:	92 ms / 132 ms
QRS:	78 ms
QT / QTc / QTd:	451 ms / 448 ms / -
P/QRS/T axis:	38° / 91° / 78°
Heartrate:	58 bpm

**Beats**		**RR**	
Total:	288	Minimum	910 ms
Normal:	288	Maximum	1188 ms
Other:	0	Mean:	1036 ms
		SD:	49 ms

R-R Trend

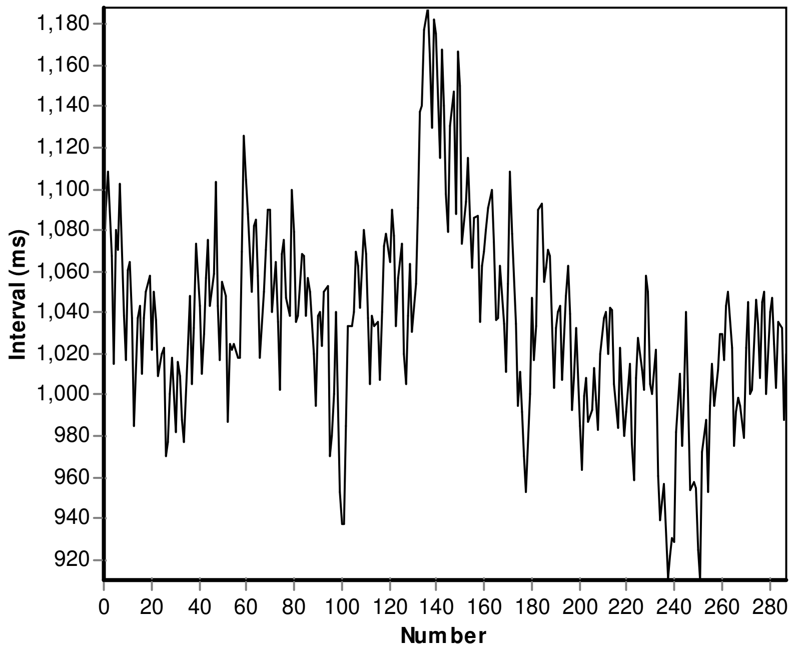

R-R Histogram

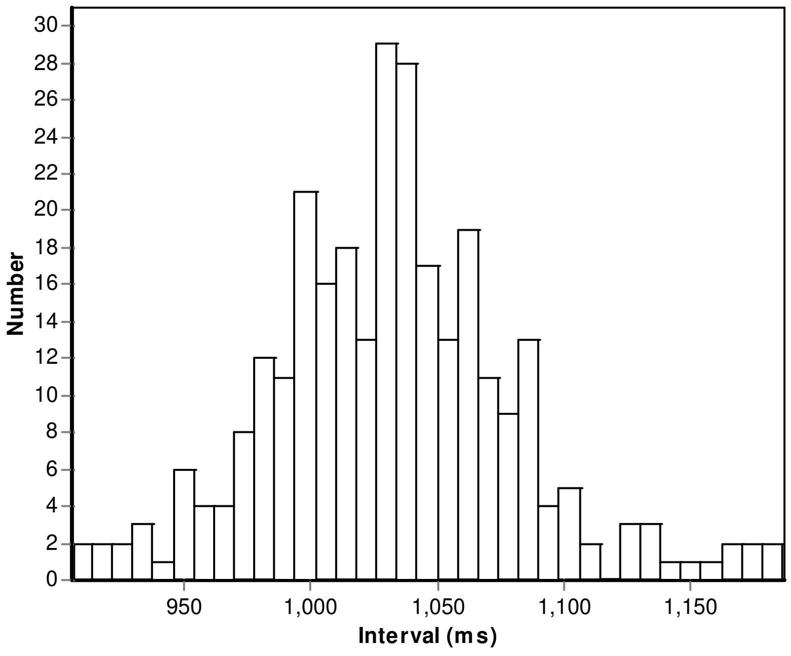

Cardio Control NV. User: Anton

Heart Rate Variability: Time Domain Analysis

**Name:**	016, 016	**Birthdate:**	26/11/1970
**Number:**	016	**Recorded:**	06/05/2018 09:18:36
**Gender:**	Female		

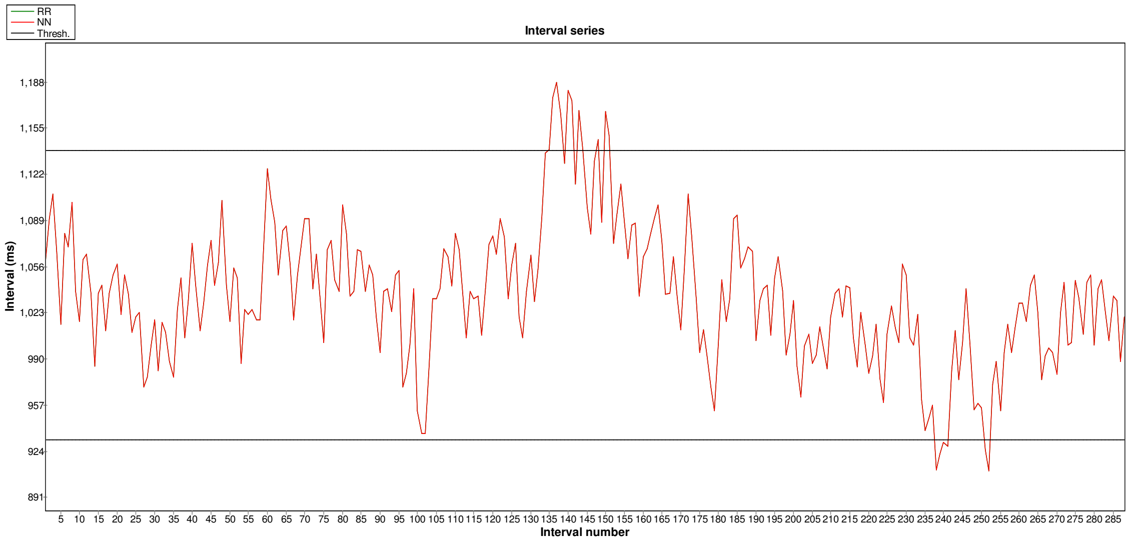

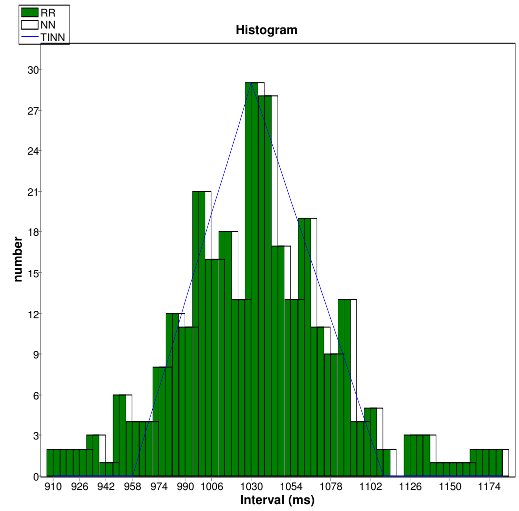

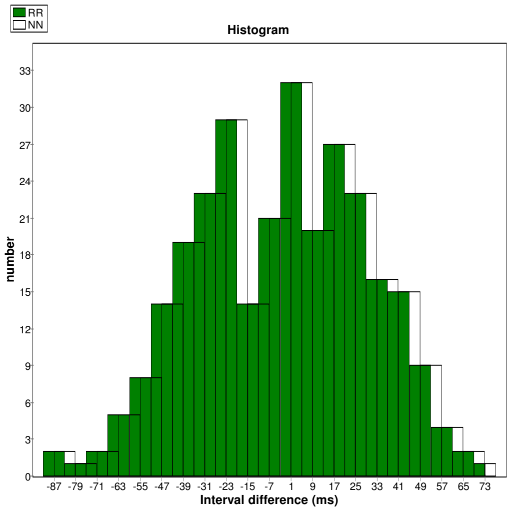

Binsize (ms) = 8

**HRV parameters**	**NN**	**RR**
SDNN (ms)	49	49
Triangular Interpolation (ms)	152	152
Triangular Index	9.93	9.93

**HRV parameters**	**NN**	**RR**
SDSD (ms)	32	32
RMSSD (ms)	32	32
NN50	30	30
NN50(1)	15	15
NN50(2)	15	15
pNN50	0.10	0.10
pNN50(1)	0.05	0.05
pNN50(2)	0.05	0.05
Logarithmic Index	0.35	0.35
SD(Logarithmic Index)	0.05	0.05

**Interval statistics**	**NN**	**RR**
Number	288	288
Minimum (ms)	910	910
Maximum (ms)	1188	1188
Range (ms)	278	278
Avg (ms)	1036	1036
SD (ms)	49	49
AvgDev (ms)	37	37
p5 (ms)	955	955
p50 (ms)	1036	1036
p95 (ms)	1128	1128
Skewness	0.35	0.35
Kurtosis	3.76	3.76

**Interval statistics**	**NN**	**RR**
Number	287	287
Minimum (ms)	-87	-87
Maximum (ms)	79	79
Range (ms)	166	166
Avg (ms)	-0	-0
SD (ms)	32	32
AvgDev (ms)	27	27
p5 (ms)	-52	-52
p50 (ms)	3	3
p95 (ms)	52	52
Skewness	-0.10	-0.10
Kurtosis	2.39	2.39

Cardio Control NV. User: Anton

Heart Rate Variability: Frequency Domain Analysis

**Name:**	016, 016	**Birthdate:**	26/11/1970
**Number:**	016	**Recorded:**	06/05/2018 09:18:36
**Gender:**	Female		

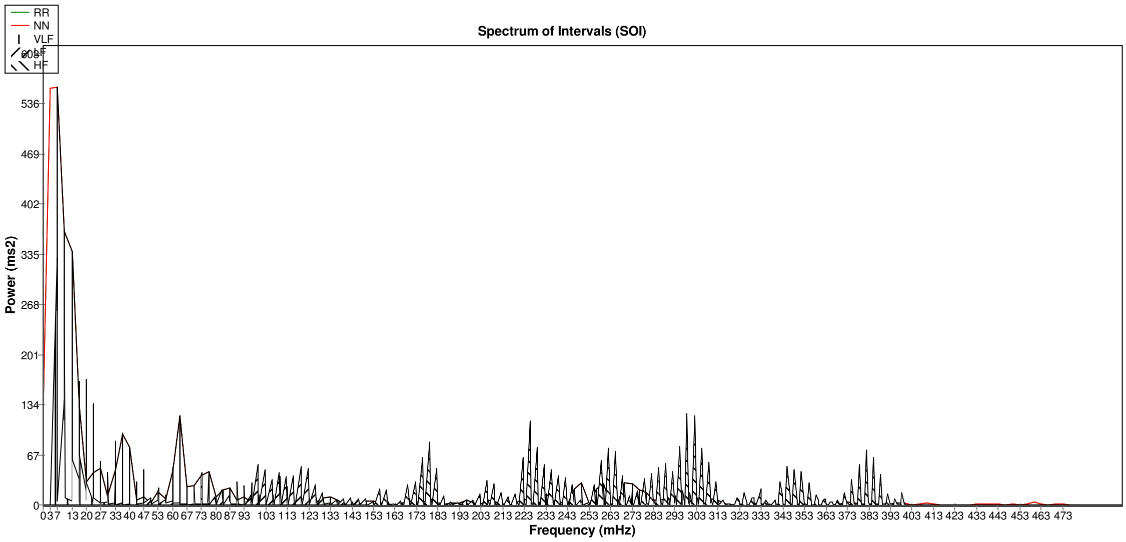

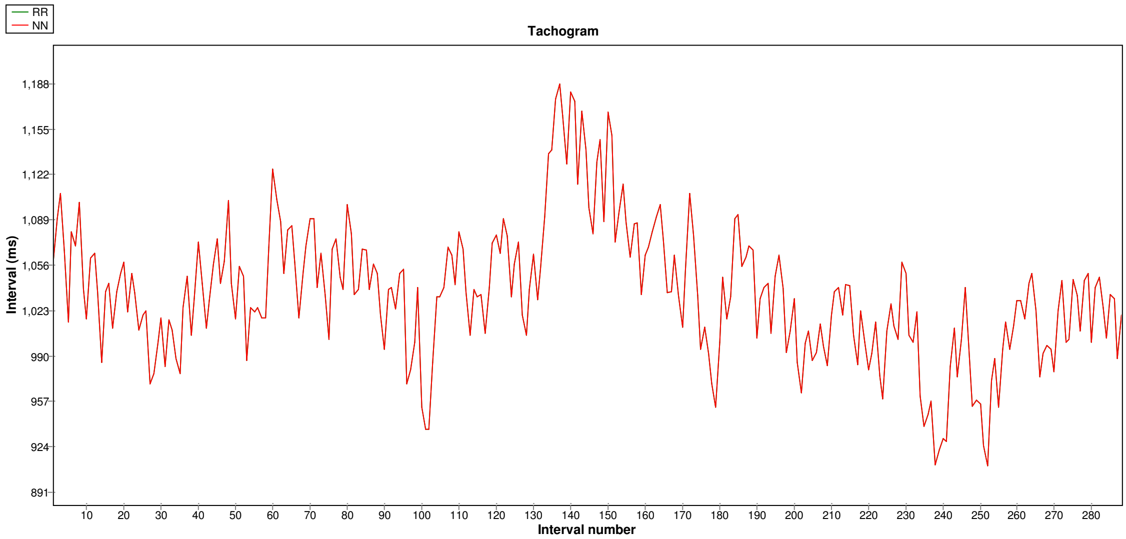

HRV parameters	NN	RR
TP (ms2)	2627	2627
VLF (ms2)	1751	1751
LF (ms2)	496	496
HF (ms2)	380	380
LF/HF	1.30	1.30
LF normalized	56.61	56.61
HF normalized	43.39	43.39
VLF peak (mHz)	7	7
LF peak (mHz)	63	63
HF peak (mHz)	250	250

HRV spectral settings	
Spectrum of Intervals (SOI)	
Frequency resolution (mHz)	3
VLF lower boundary (mHz)	3
VLF upper boundary (mHz)	40
LF upper boundary (mHz)	150
HF upper boundary (mHz)	400
Smoothing factor	1
Tapering	Hann
Fourier transform	DFT
Sample frequency (Hz)	0.97
Interval correction	Annotation
Interval threshold (%)	10
