# Supplementary material for: The effects of a 3-day mountain bike cycling race on the autonomic nervous system (ANS) and heart rate variability in amateur cyclists: a prospective quantitative research design
Source: BMC Sports Sci Med Rehabil. 2023 Jan 2;15:2. doi: 10.1186/s13102-022-00614-y (PMC9808932; doi:10.1186/s13102-022-00614-y)
Supplement: Supplementary file 1 — Additional file 1. Individual data of Participants. [file 13102_2022_614_MOESM1_ESM.zip › Individual data of Participants/HRV Data/016/ECG_016_20180501105052_.PDF]

Anton Swart Biokinetic Rehabilitation Practice

Name:	017 017	Recorded:	01/05/2018 10:50:52	UNCONFIRMED INTERPRETATION - MD SHOULD REVIEW
Number:	017	Recorded by:	Mr. Anton Swart	
Gender:	Male	Referring physician:		
Birthdate:	17/11/1971 46 years	Ordering physician:		
		Attending physician:		
P / PQ:	112 ms / 185 ms	Location:	Anton Swart Biokinetic Rehabilitation Practi	
QRS:	112 ms	Comment:		
QT / QTc / QTd:	403 ms / 398 ms / -			
P/QRS/T axis:	63° / 87° / 82°			
Heartrate:	57 bpm			

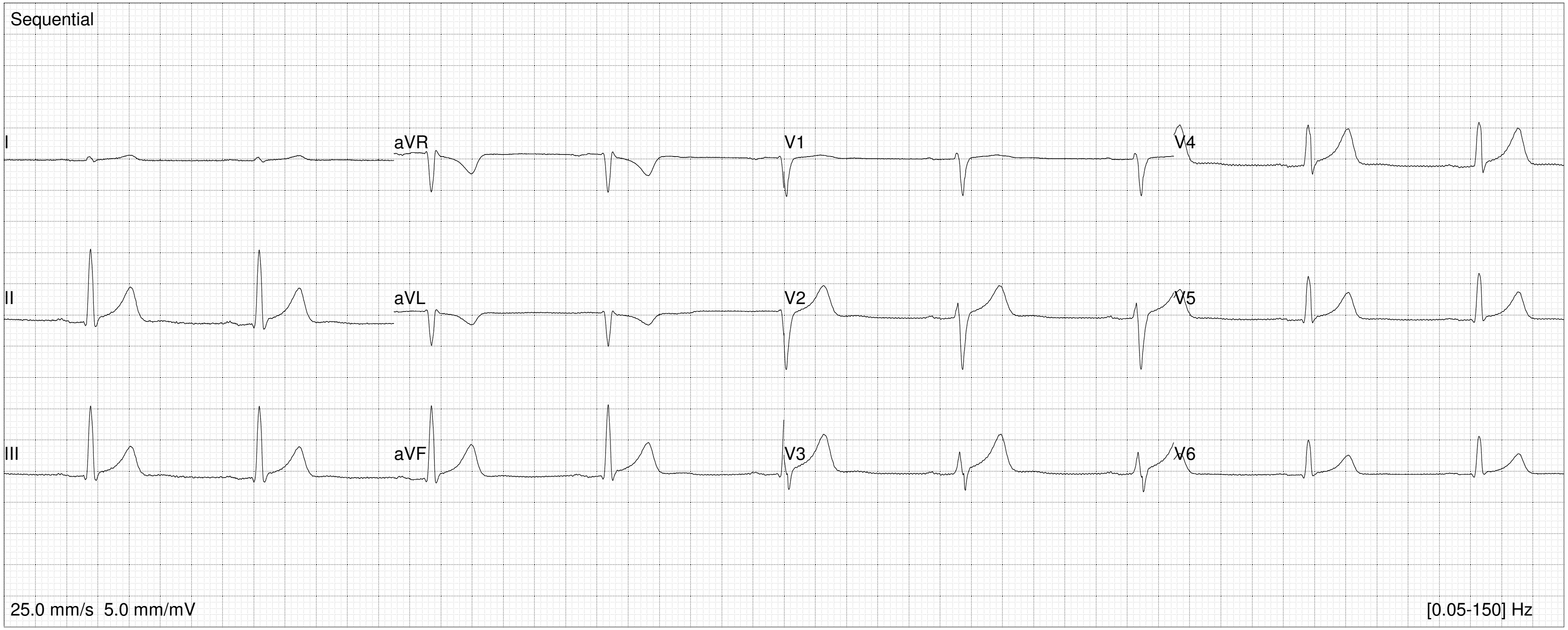

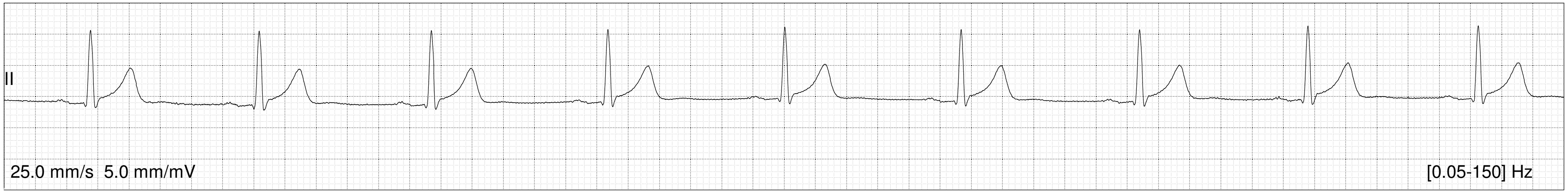

Anton Swart Biokinetic Rehabilitation Practice

Name:	017 017	Recorded:	01/05/2018 10:50:52	UNCONFIRMED INTERPRETATION - MD SHOULD REVIEW
Number:	017	Recorded by:	Mr. Anton Swart	
Gender:	Male	Referring physician:		
Birthdate:	17/11/1971 46 years	Location:	Anton Swart Biokinetic Rehabilitation Practice	
P / PQ:	112 ms / 185 ms	Ordering physician:		
QRS:	112 ms	Attending physician:		
QT / QTc / QTd:	403 ms / 398 ms / -	Comment:		
P/QRS/T axis:	63° / 87° / 82°			
Heartrate:	57 bpm			

**Beats**		**RR**	
Total:	285	Minimum	955 ms
Normal:	285	Maximum	1143 ms
Other:	0	Mean:	1047 ms
		SD:	39 ms

R-R Trend

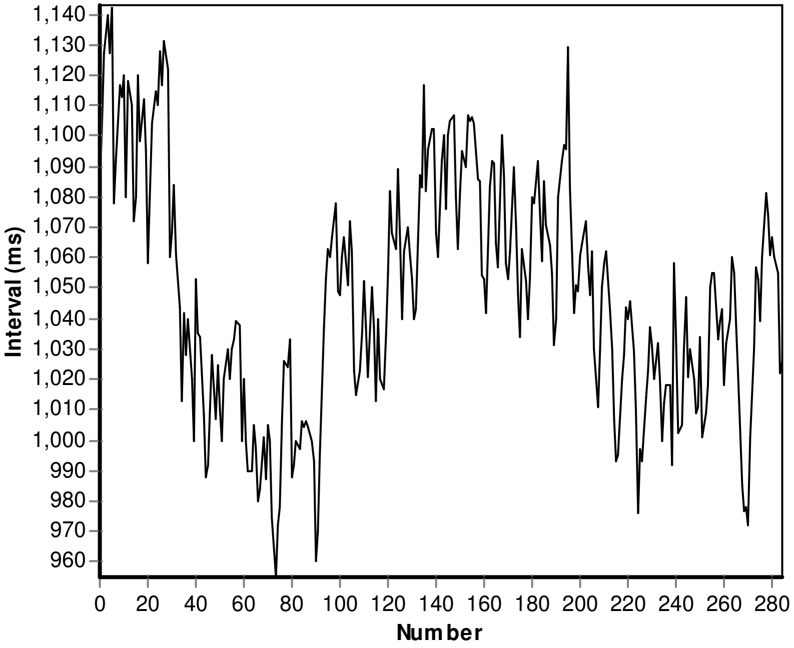

R-R Histogram

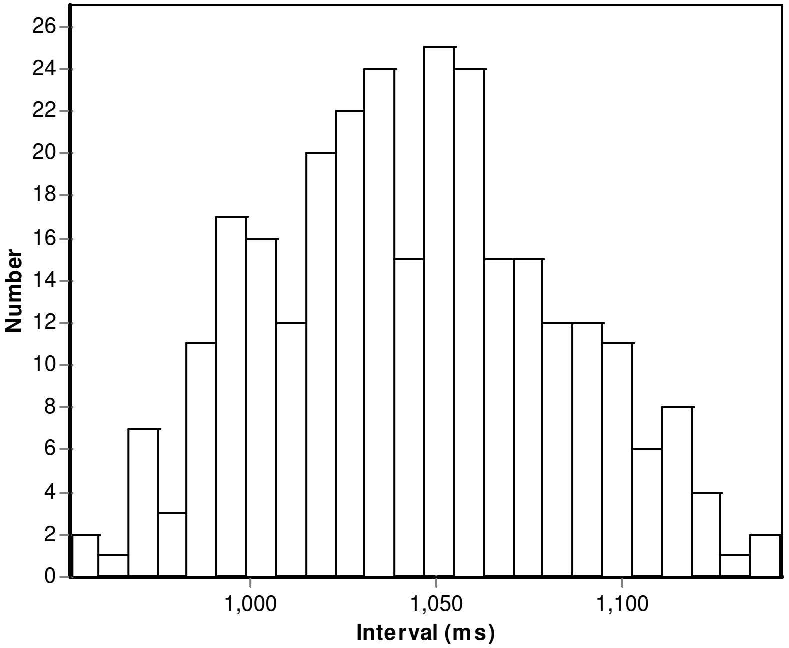

Cardio Control NV. User: Anton

Heart Rate Variability: Time Domain Analysis

**Name:**	017, 017	**Birthdate:**	17/11/1971
**Number:**	017	**Recorded:**	01/05/2018 10:50:52
**Gender:**	Male		

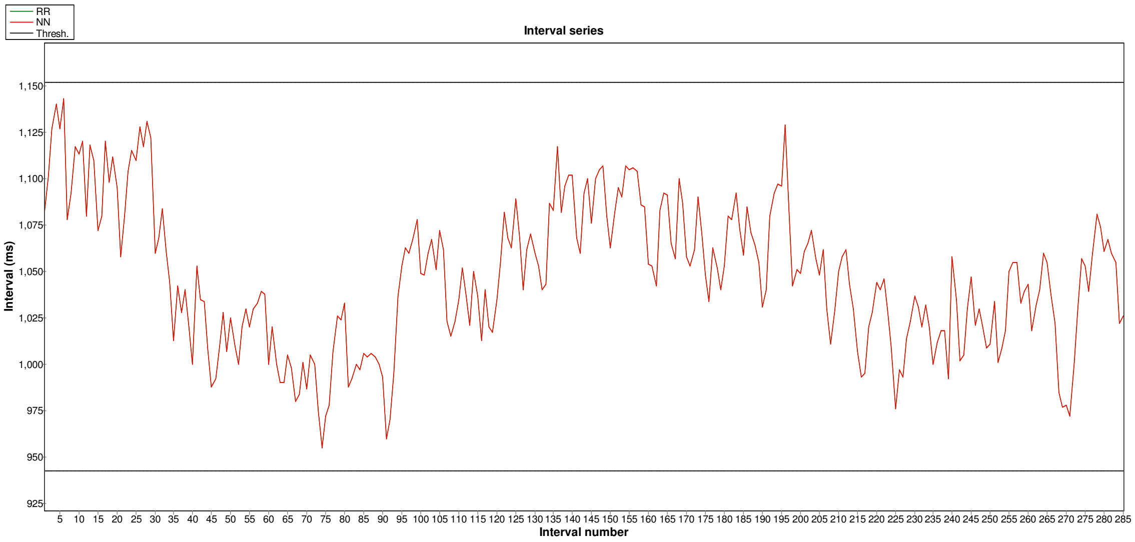

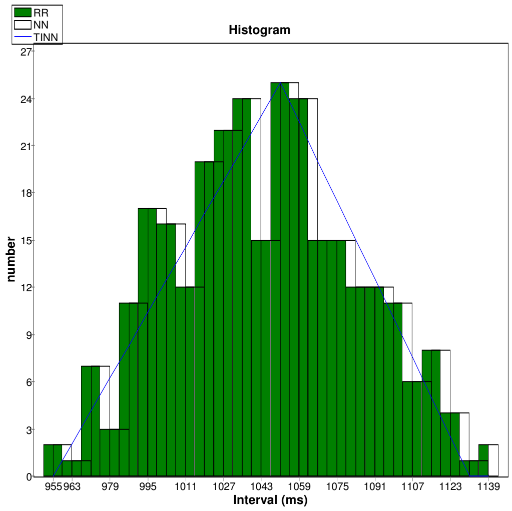

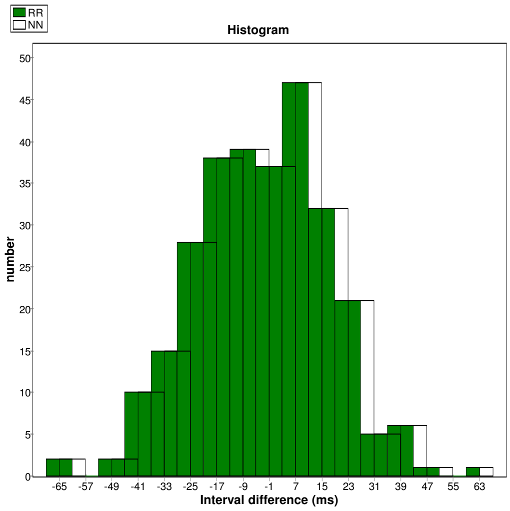

Binsize (ms) = 8

**HRV parameters**	**NN**	**RR**
SDNN (ms)	39	39
Triangular Interpolation (ms)	176	176
Triangular Index	11.40	11.40

**HRV parameters**	**NN**	**RR**
SDSD (ms)	20	20
RMSSD (ms)	20	20
NN50	4	4
NN50(1)	2	2
NN50(2)	2	2
pNN50	0.01	0.01
pNN50(1)	0.01	0.01
pNN50(2)	0.01	0.01
Logarithmic Index	0.62	0.62
SD(Logarithmic Index)	0.09	0.09

**Interval statistics**	**NN**	**RR**
Number	285	285
Minimum (ms)	955	955
Maximum (ms)	1143	1143
Range (ms)	188	188
Avg (ms)	1047	1047
SD (ms)	39	39
AvgDev (ms)	32	32
p5 (ms)	988	988
p50 (ms)	1048	1048
p95 (ms)	1117	1117
Skewness	0.13	0.13
Kurtosis	2.43	2.43

**Interval statistics**	**NN**	**RR**
Number	284	284
Minimum (ms)	-65	-65
Maximum (ms)	66	66
Range (ms)	131	131
Avg (ms)	-0	-0
SD (ms)	20	20
AvgDev (ms)	16	16
p5 (ms)	-34	-34
p50 (ms)	0	0
p95 (ms)	29	29
Skewness	-0.08	-0.08
Kurtosis	3.15	3.15

Heart Rate Variability: Frequency Domain Analysis

**Name:**	017, 017	**Birthdate:**	17/11/1971
**Number:**	017	**Recorded:**	01/05/2018 10:50:52
**Gender:**	Male		

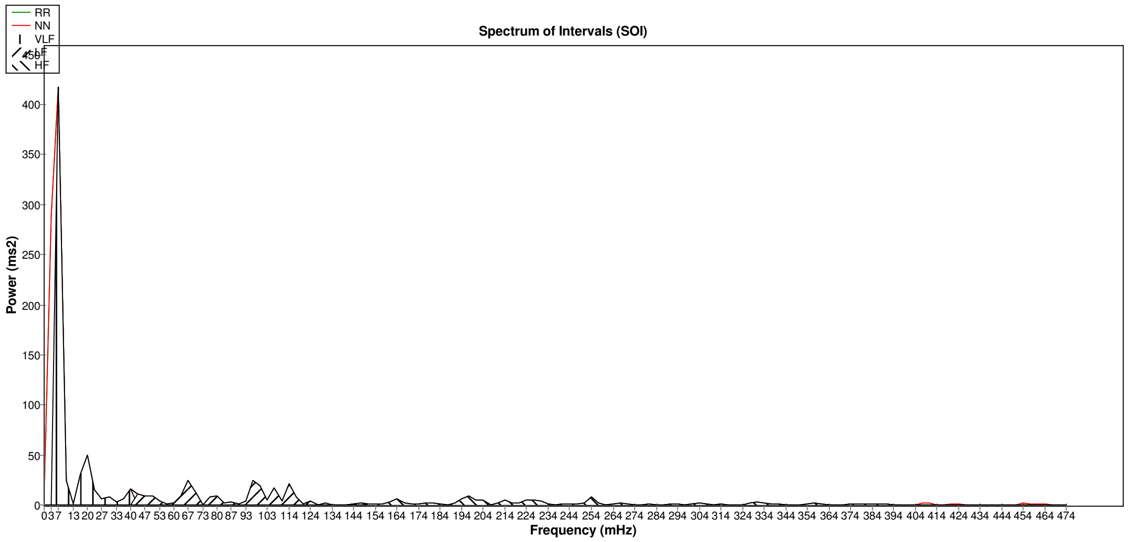

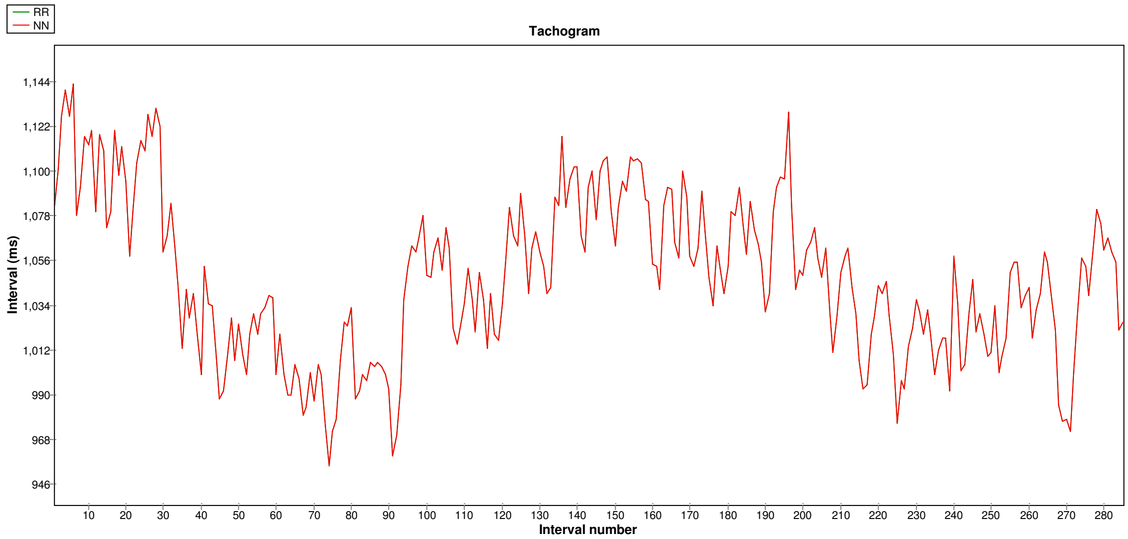

**HRV parameters**	**NN**	**RR**
TP (ms2)	924	924
VLF (ms2)	578	578
LF (ms2)	223	223
HF (ms2)	123	123
LF/HF	1.82	1.82
LF normalized	64.54	64.54
HF normalized	35.46	35.46
VLF peak (mHz)	7	7
LF peak (mHz)	67	67
HF peak (mHz)	197	197

**HRV spectral settings**	
Spectrum of Intervals (SOI)	
Frequency resolution (mHz)	3
VLF lower boundary (mHz)	3
VLF upper boundary (mHz)	40
LF upper boundary (mHz)	150
HF upper boundary (mHz)	400
Smoothing factor	1
Tapering	Hann
Fourier transform	DFT
Sample frequency (Hz)	0.95
Interval correction	Annotation
Interval threshold (%)	10
