# Supplementary material for: The effects of a 3-day mountain bike cycling race on the autonomic nervous system (ANS) and heart rate variability in amateur cyclists: a prospective quantitative research design
Source: BMC Sports Sci Med Rehabil. 2023 Jan 2;15:2. doi: 10.1186/s13102-022-00614-y (PMC9808932; doi:10.1186/s13102-022-00614-y)
Supplement: Supplementary file 1 — Additional file 1. Individual data of Participants. [file 13102_2022_614_MOESM1_ESM.zip › Individual data of Participants/HRV Data/016/ECG_016_20180503171449_.PDF]

Anton Swart Biokinetic Rehabilitation Practice

Name:	017 017	Recorded:	03/05/2018 17:14:49	UNCONFIRMED INTERPRETATION - MD SHOULD REVIEW
Number:	017	Recorded by:	Mr. Anton Swart	
Gender:	Male	Referring physician:		
Birthdate:	17/11/1971 46 years	Ordering physician:		
		Attending physician:		
P / PQ:	122 ms / 182 ms	Location:	Anton Swart Biokinetic Rehabilitation Practi	
QRS:	111 ms	Comment:		
QT / QTc / QTd:	375 ms / 394 ms / -			
P/QRS/T axis:	74° / 87° / 79°			
Heartrate:	71 bpm			

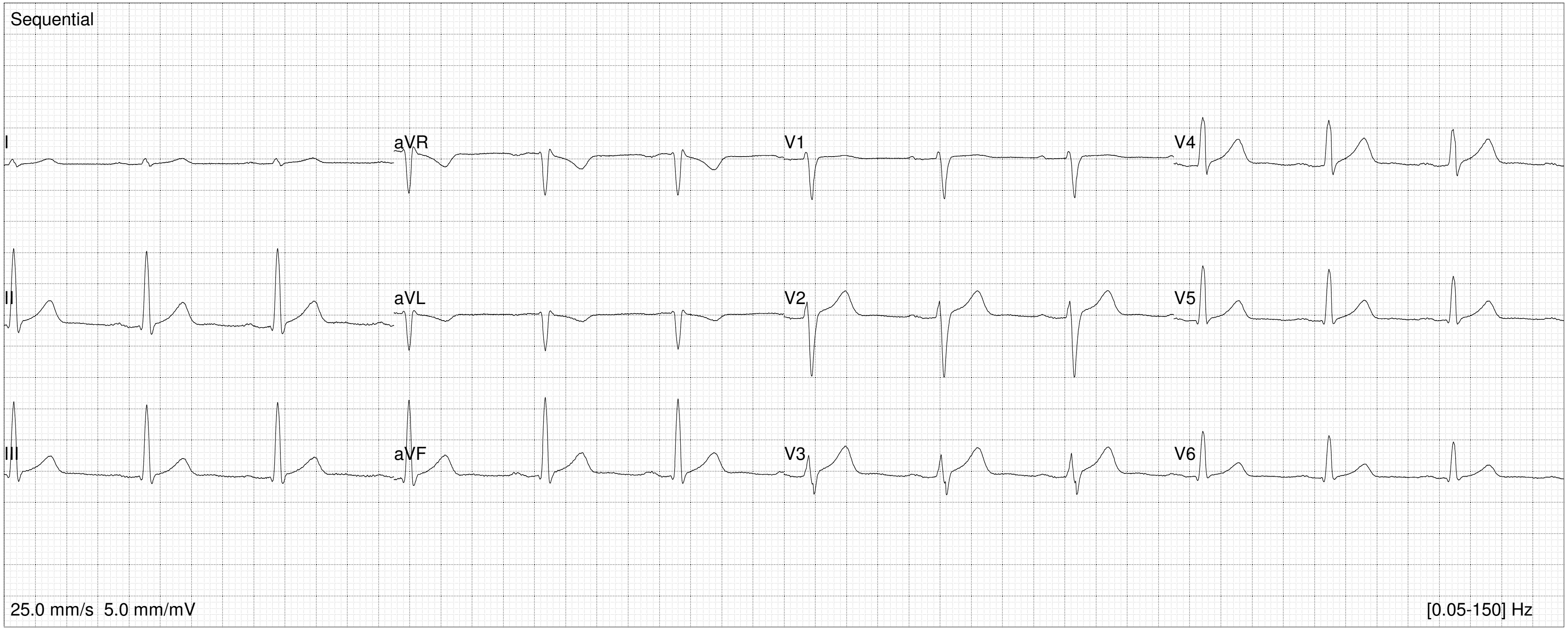

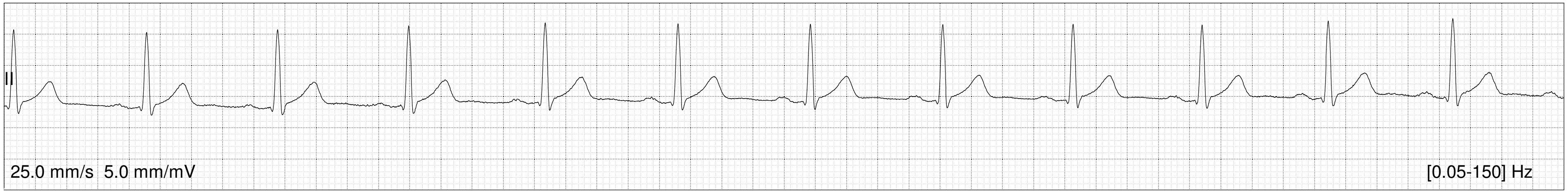

Anton Swart Biokinetic Rehabilitation Practice

Name:	017 017	Recorded:	03/05/2018 17:14:49	UNCONFIRMED INTERPRETATION - MD SHOULD REVIEW
Number:	017	Recorded by:	Mr. Anton Swart	
Gender:	Male	Referring physician:		
Birthdate:	17/11/1971 46 years	Location:	Anton Swart Biokinetic Rehabilitation Practice	
		Ordering physician:		
		Attending physician:		
		Comment:		

P / PQ:	122 ms / 182 ms
QRS:	111 ms
QT / QTc / QTd:	375 ms / 394 ms / -
P/QRS/T axis:	74° / 87° / 79°
Heartrate:	71 bpm

**Beats**		**RR**	
Total:	360	Minimum	733 ms
Normal:	360	Maximum	951 ms
Other:	0	Mean:	832 ms
		SD:	35 ms

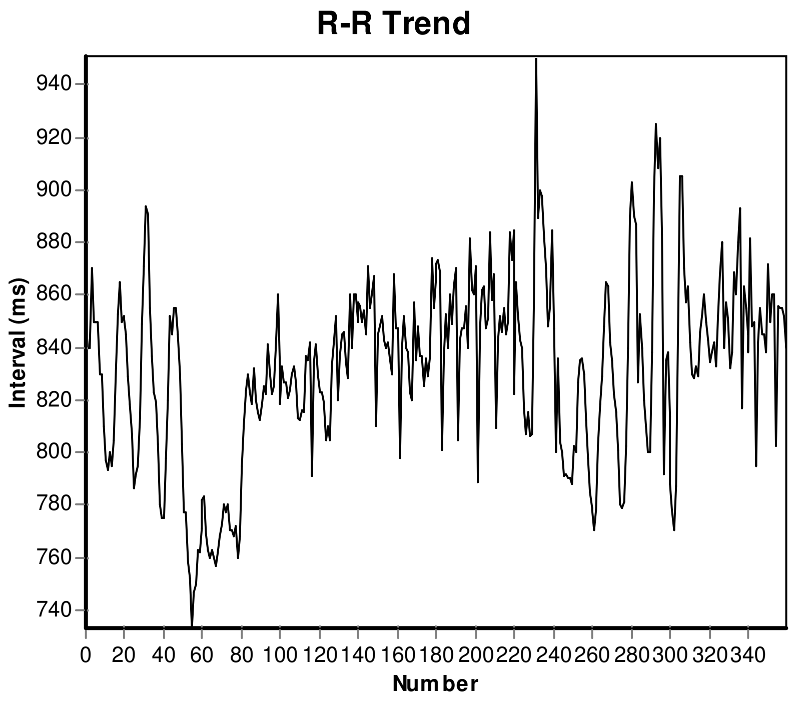

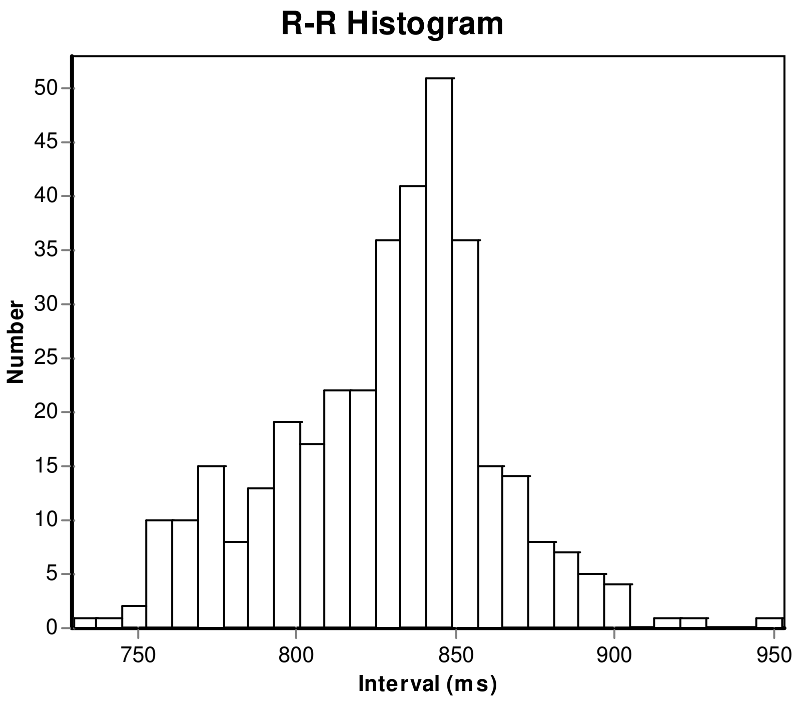

Heart Rate Variability: Time Domain Analysis

**Name:**	017, 017	**Birthdate:**	17/11/1971
**Number:**	017	**Recorded:**	03/05/2018 17:14:49
**Gender:**	Male		

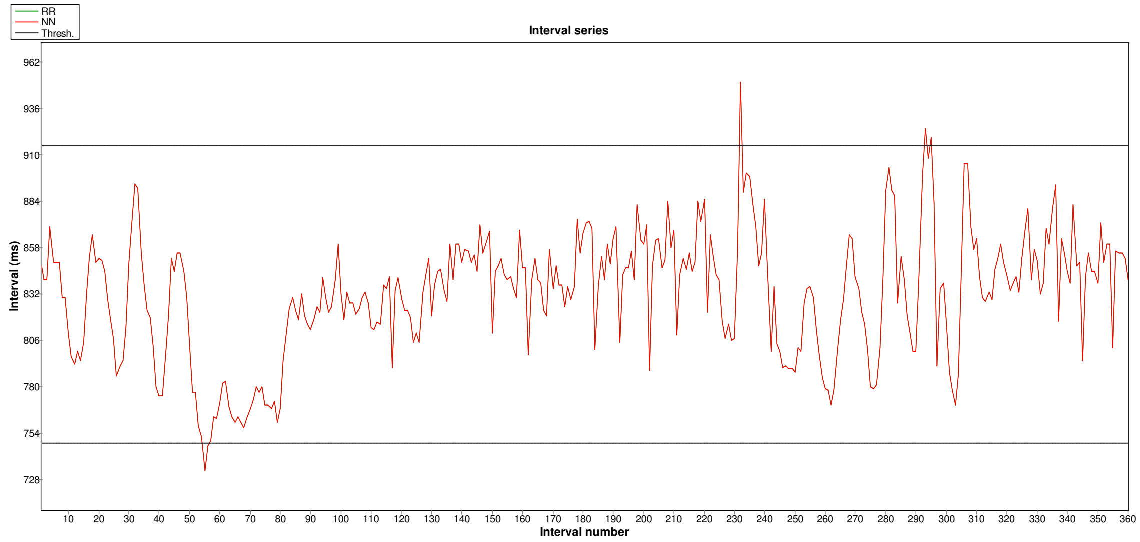

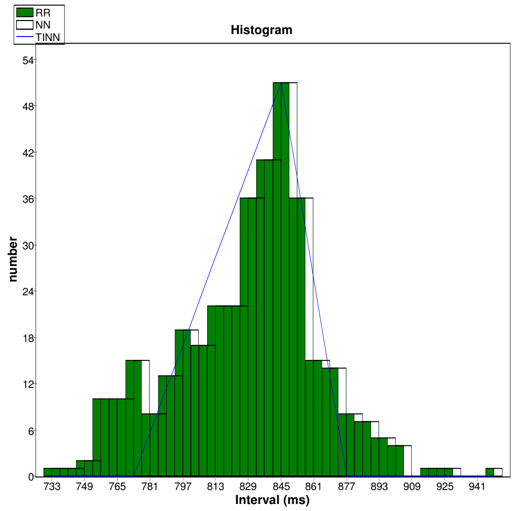

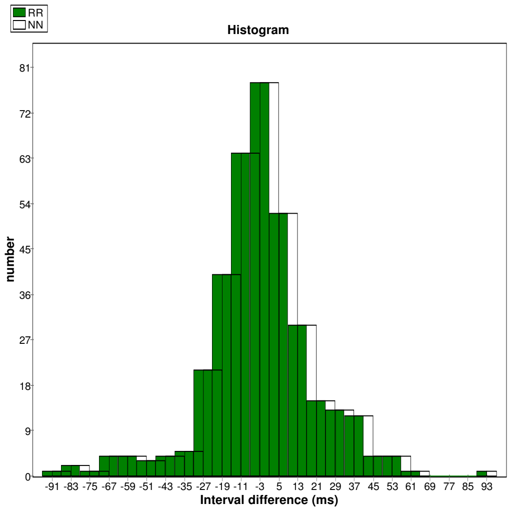

Binsize (ms) = 8

HRV parameters	NN	RR
SDNN (ms)	35	35
Triangular Interpolation (ms)	104	104
Triangular Index	7.06	7.06

HRV parameters	NN	RR
SDSD (ms)	23	23
RMSSD (ms)	23	23
NN50	19	19
NN50(1)	13	13
NN50(2)	6	6
pNN50	0.05	0.05
pNN50(1)	0.04	0.04
pNN50(2)	0.02	0.02
Logarithmic Index	0.45	0.45
SD(Logarithmic Index)	0.04	0.04

Interval statistics	NN	RR
Number	360	360
Minimum (ms)	733	733
Maximum (ms)	951	951
Range (ms)	218	218
Avg (ms)	832	832
SD (ms)	35	35
AvgDev (ms)	27	27
p5 (ms)	770	770
p50 (ms)	837	837
p95 (ms)	885	885
Skewness	-0.20	-0.20
Kurtosis	3.13	3.13

Interval statistics	NN	RR
Number	359	359
Minimum (ms)	-91	-91
Maximum (ms)	94	94
Range (ms)	185	185
Avg (ms)	-0	-0
SD (ms)	23	23
AvgDev (ms)	16	16
p5 (ms)	-37	-37
p50 (ms)	0	0
p95 (ms)	38	38
Skewness	-0.23	-0.23
Kurtosis	5.50	5.50

Heart Rate Variability: Frequency Domain Analysis

Name: 017, 017
Number: 017
Gender: Male
Birthdate: 17/11/1971
Recorded: 03/05/2018 17:14:49

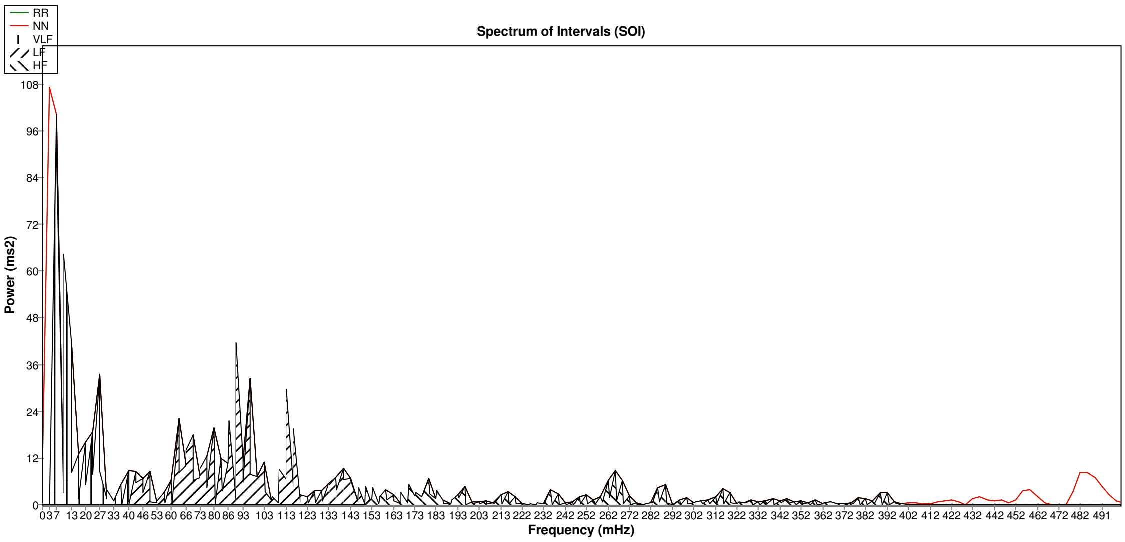

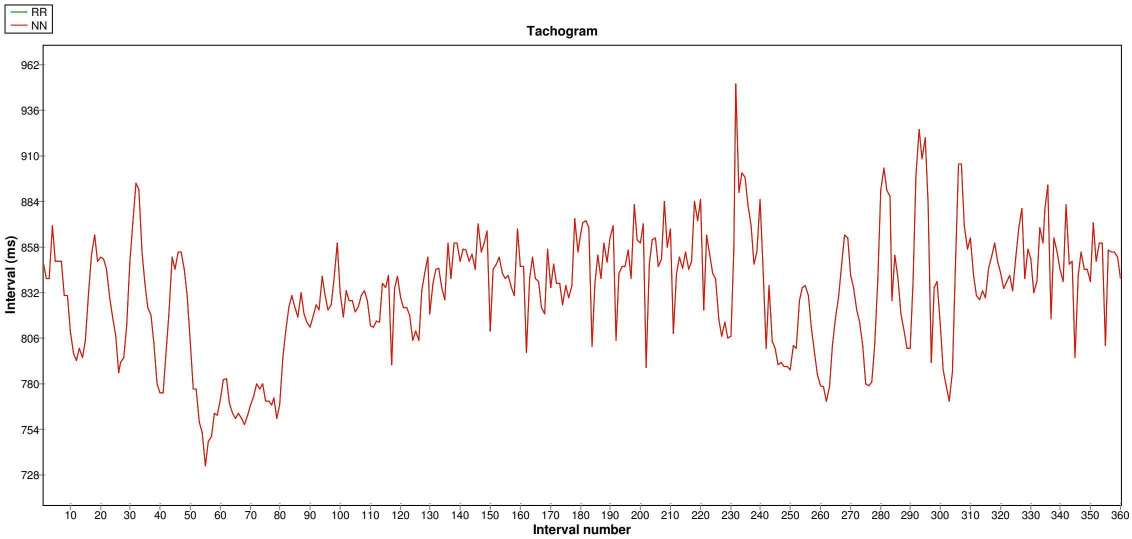

HRV parameters	NN	RR
TP (ms2)	638	638
VLF (ms2)	243	243
LF (ms2)	263	263
HF (ms2)	133	133
LF/HF	1.98	1.98
LF normalized	66.45	66.45
HF normalized	33.55	33.55
VLF peak (mHz)	7	7
LF peak (mHz)	96	96
HF peak (mHz)	266	266

HRV spectral settings	
Spectrum of Intervals (SOI)	
Frequency resolution (mHz)	3
VLF lower boundary (mHz)	3
VLF upper boundary (mHz)	40
LF upper boundary (mHz)	150
HF upper boundary (mHz)	400
Smoothing factor	1
Tapering	Hann
Fourier transform	DFT
Sample frequency (Hz)	1.20
Interval correction	Annotation
Interval threshold (%)	10
