# Supplementary material for: The effects of a 3-day mountain bike cycling race on the autonomic nervous system (ANS) and heart rate variability in amateur cyclists: a prospective quantitative research design
Source: BMC Sports Sci Med Rehabil. 2023 Jan 2;15:2. doi: 10.1186/s13102-022-00614-y (PMC9808932; doi:10.1186/s13102-022-00614-y)
Supplement: Supplementary file 1 — Additional file 1. Individual data of Participants. [file 13102_2022_614_MOESM1_ESM.zip › Individual data of Participants/HRV Data/016/ECG_016_20180504130512_.PDF]

Anton Swart Biokinetic Rehabilitation Practice

UNCONFIRMED INTERPRETATION - MD SHOULD REVIEW

Name:	017 017	Recorded:	04/05/2018 13:05:12
Number:	017	Recorded by:	Mr. Anton Swart
Gender:	Male	Referring physician:	
Birthdate:	17/11/1971 46 years	Ordering physician:	
		Attending physician:	
		Location:	Anton Swart Biokinetic Rehabilitation Practi
		Comment:	

P / PQ:	118 ms / 167 ms
QRS:	96 ms
QT / QTc / QTd:	378 ms / 408 ms / -
P/QRS/T axis:	80° / 86° / 80°
Heartrate:	77 bpm

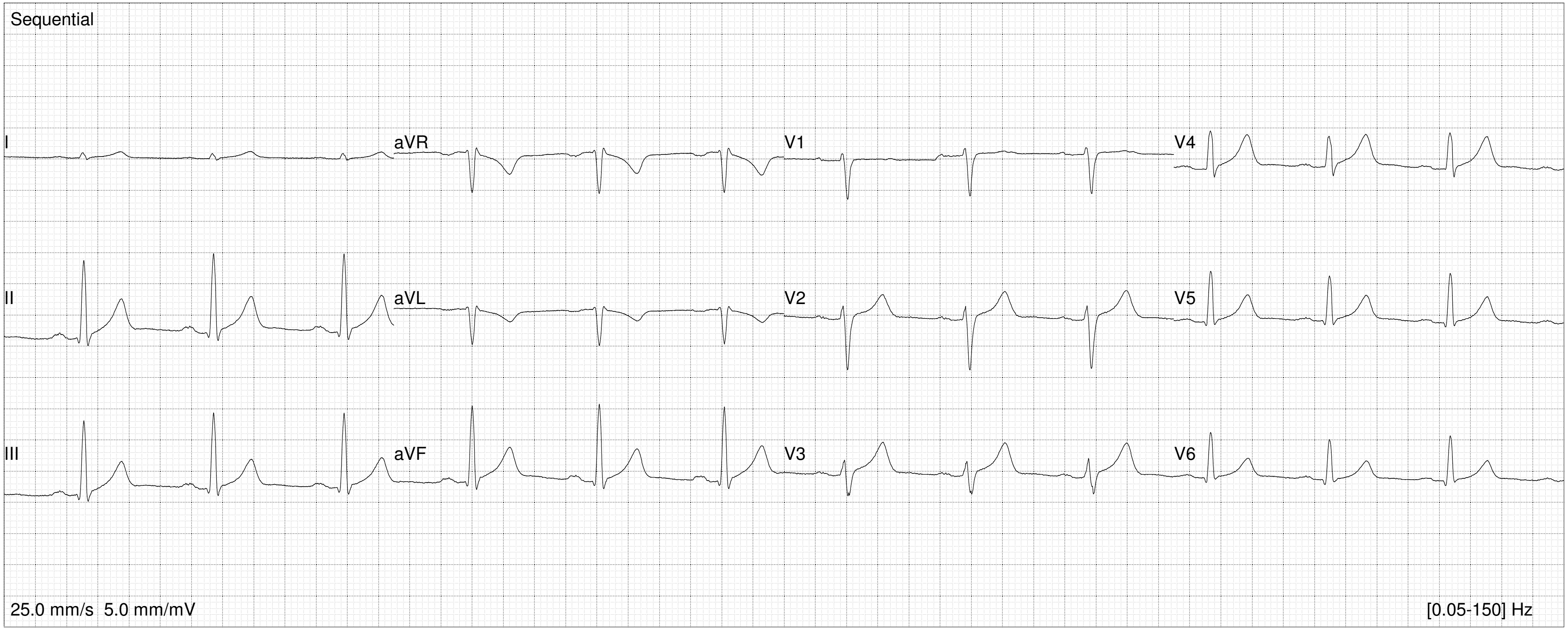

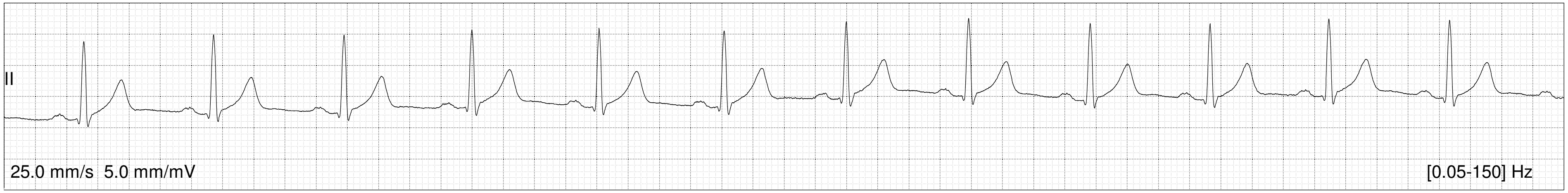

Anton Swart Biokinetic Rehabilitation Practice

Name:	017 017	Recorded:	04/05/2018 13:05:12	UNCONFIRMED INTERPRETATION - MD SHOULD REVIEW
Number:	017	Recorded by:	Mr. Anton Swart	
Gender:	Male	Referring physician:		
Birthdate:	17/11/1971 46 years	Location:	Anton Swart Biokinetic Rehabilitation Practice	
P / PQ:	118 ms / 167 ms	Ordering physician:		
QRS:	96 ms	Attending physician:		
QT / QTc / QTd:	378 ms / 408 ms / -	Comment:		
P/QRS/T axis:	80° / 86° / 80°			
Heartrate:	77 bpm			

**Beats**		**RR**	
Total:	377	Minimum	693 ms
Normal:	377	Maximum	960 ms
Other:	0	Mean:	792 ms
		SD:	49 ms

R-R Trend

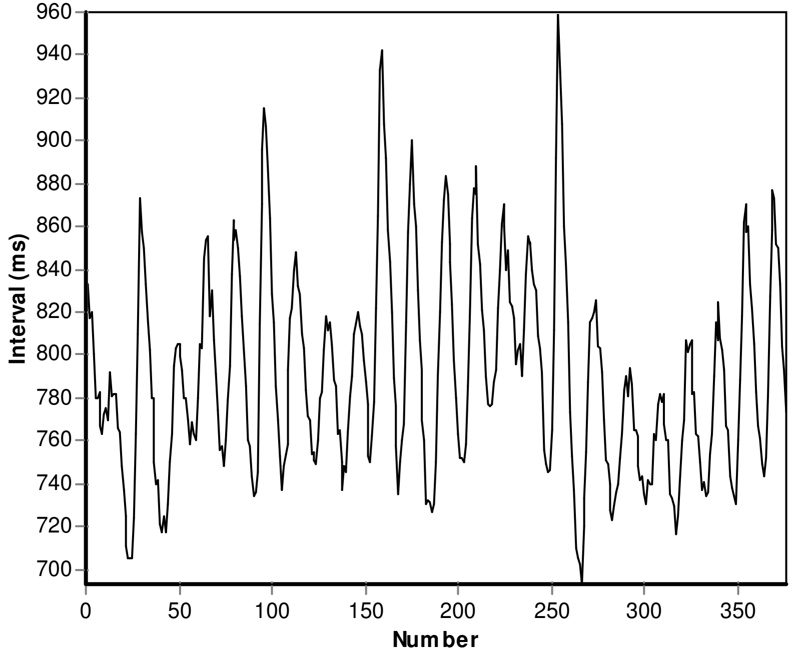

R-R Histogram

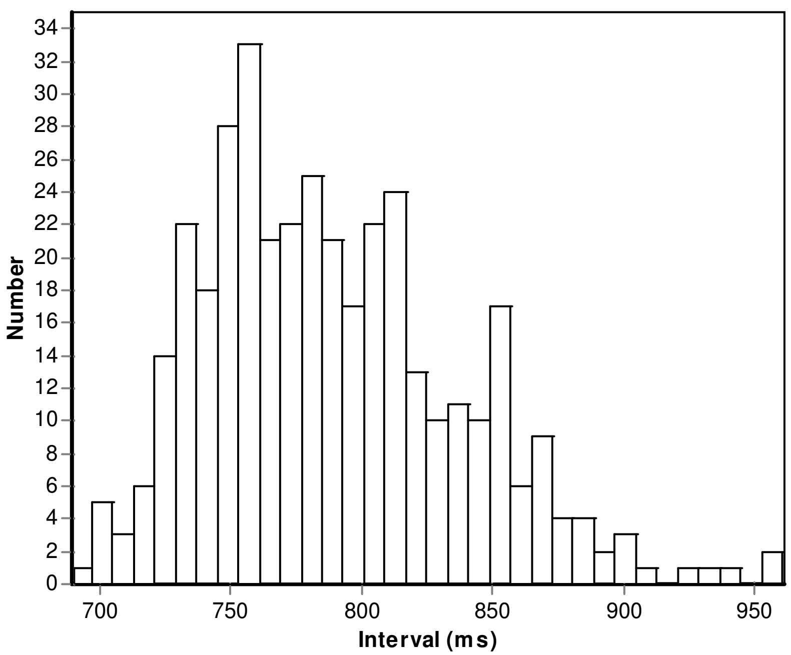

Heart Rate Variability: Time Domain Analysis

**Name:**	017, 017	**Birthdate:**	17/11/1971
**Number:**	017	**Recorded:**	04/05/2018 13:05:12
**Gender:**	Male		

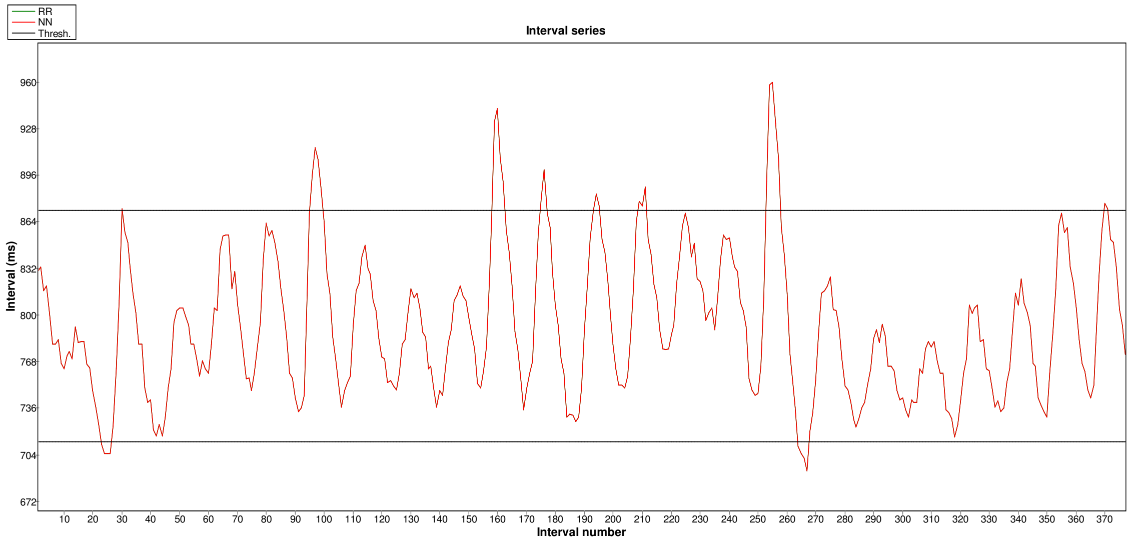

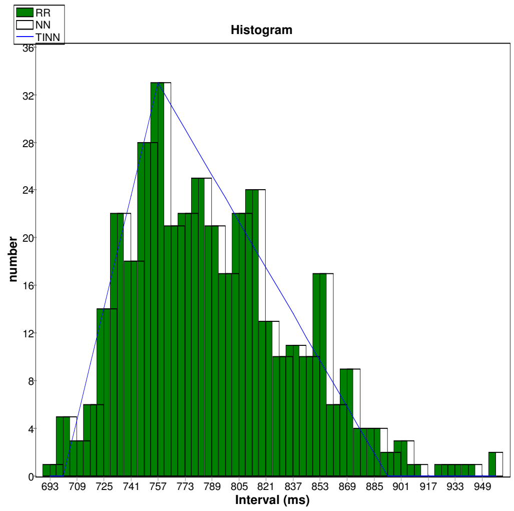

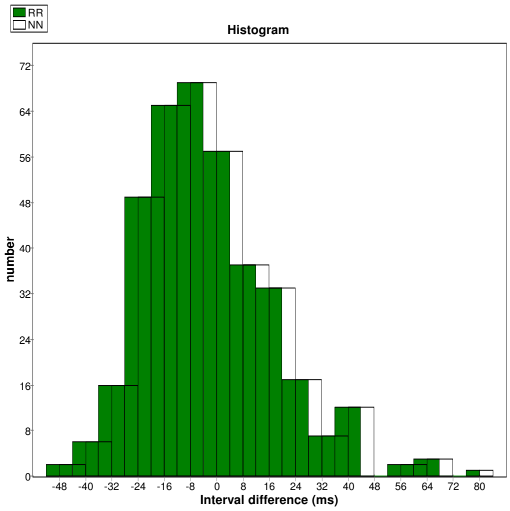

Binsize (ms) = 8

**HRV parameters**	**NN**	**RR**
SDNN (ms)	49	49
Triangular Interpolation (ms)	192	192
Triangular Index	11.42	11.42

**HRV parameters**	**NN**	**RR**
SDSD (ms)	20	20
RMSSD (ms)	20	20
NN50	6	6
NN50(1)	0	0
NN50(2)	6	6
pNN50	0.02	0.02
pNN50(1)	0.00	0.00
pNN50(2)	0.02	0.02
Logarithmic Index	0.59	0.59
SD(Logarithmic Index)	0.08	0.08

**Interval statistics**	**NN**	**RR**
Number	377	377
Minimum (ms)	693	693
Maximum (ms)	960	960
Range (ms)	267	267
Avg (ms)	792	792
SD (ms)	49	49
AvgDev (ms)	39	39
p5 (ms)	727	727
p50 (ms)	785	785
p95 (ms)	878	878
Skewness	0.65	0.65
Kurtosis	3.20	3.20

**Interval statistics**	**NN**	**RR**
Number	376	376
Minimum (ms)	-48	-48
Maximum (ms)	80	80
Range (ms)	128	128
Avg (ms)	-0	-0
SD (ms)	20	20
AvgDev (ms)	15	15
p5 (ms)	-27	-27
p50 (ms)	-3	-3
p95 (ms)	40	40
Skewness	0.89	0.89
Kurtosis	4.28	4.28

Heart Rate Variability: Frequency Domain Analysis

**Name:**	017, 017	**Birthdate:**	17/11/1971
**Number:**	017	**Recorded:**	04/05/2018 13:05:12
**Gender:**	Male		

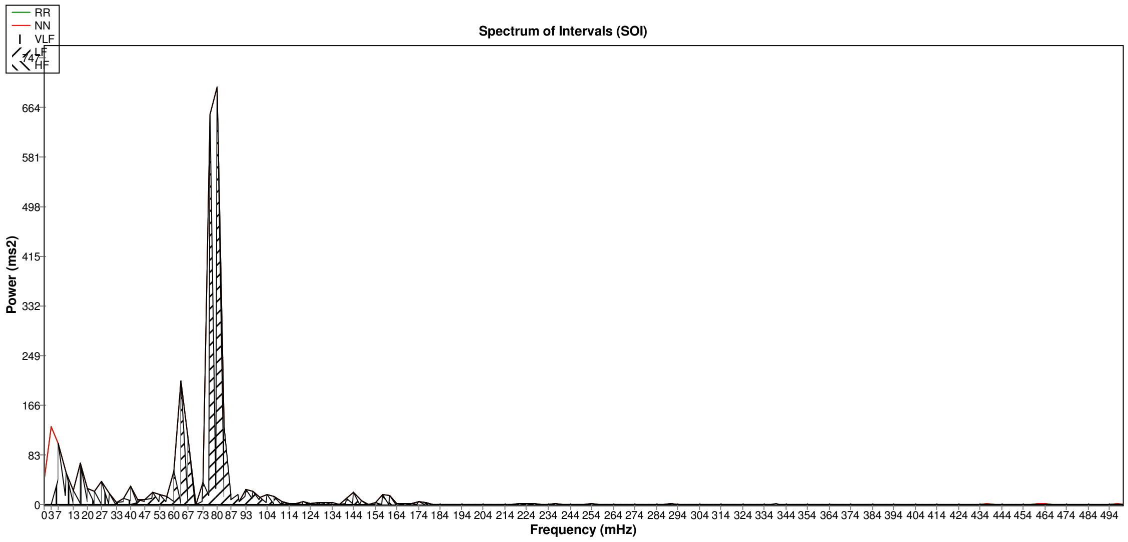

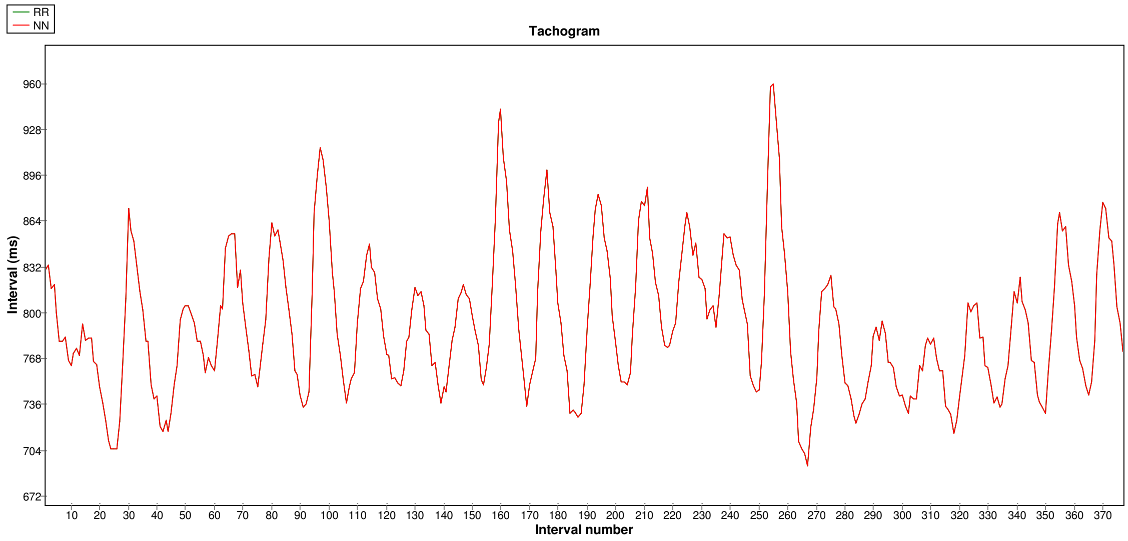

**HRV parameters**	**NN**	**RR**
TP (ms2)	2597	2597
VLF (ms2)	398	398
LF (ms2)	2127	2127
HF (ms2)	73	73
LF/HF	29.19	29.19
LF normalized	96.69	96.69
HF normalized	3.31	3.31
VLF peak (mHz)	7	7
LF peak (mHz)	80	80
HF peak (mHz)	157	157

**HRV spectral settings**	
Spectrum of Intervals (SOI)	
Frequency resolution (mHz)	3
VLF lower boundary (mHz)	3
VLF upper boundary (mHz)	40
LF upper boundary (mHz)	150
HF upper boundary (mHz)	400
Smoothing factor	1
Tapering	Hann
Fourier transform	DFT
Sample frequency (Hz)	1.26
Interval correction	Annotation
Interval threshold (%)	10
