# Supplementary material for: The effects of a 3-day mountain bike cycling race on the autonomic nervous system (ANS) and heart rate variability in amateur cyclists: a prospective quantitative research design
Source: BMC Sports Sci Med Rehabil. 2023 Jan 2;15:2. doi: 10.1186/s13102-022-00614-y (PMC9808932; doi:10.1186/s13102-022-00614-y)
Supplement: Supplementary file 1 — Additional file 1. Individual data of Participants. [file 13102_2022_614_MOESM1_ESM.zip › Individual data of Participants/HRV Data/016/ECG_016_20180505124027_.PDF]

Anton Swart Biokinetic Rehabilitation Practice

UNCONFIRMED INTERPRETATION - MD SHOULD REVIEW

Name:	017 017	Recorded:	05/05/2018 12:40:27
Number:	017	Recorded by:	Mr. Anton Swart
Gender:	Male	Referring physician:	
Birthdate:	17/11/1971 46 years	Ordering physician:	
		Attending physician:	
		Location:	Anton Swart Biokinetic Rehabilitation Practi
		Comment:	

P / PQ:	115 ms / 162 ms
QRS:	96 ms
QT / QTc / QTd:	388 ms / 416 ms / -
P/QRS/T axis:	80° / 87° / 82°
Heartrate:	76 bpm

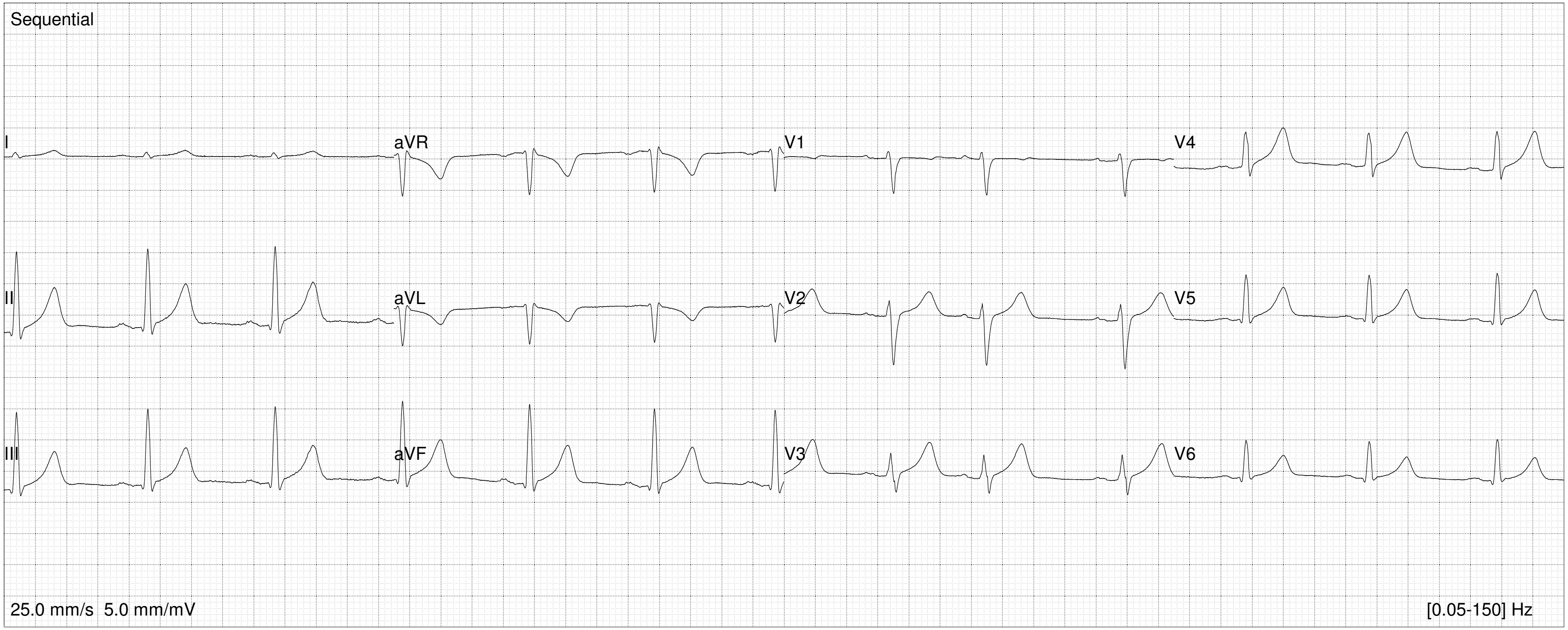

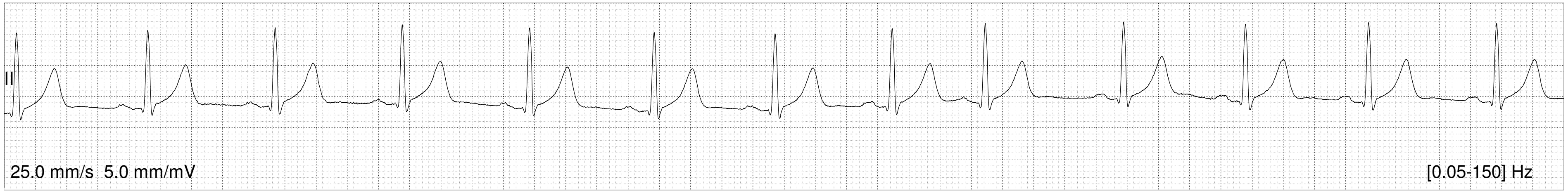

Anton Swart Biokinetic Rehabilitation Practice

Name:	017 017	Recorded:	05/05/2018 12:40:27	UNCONFIRMED INTERPRETATION - MD SHOULD REVIEW
Number:	017	Recorded by:	Mr. Anton Swart	
Gender:	Male	Referring physician:		
Birthdate:	17/11/1971 46 years	Location:	Anton Swart Biokinetic Rehabilitation Practice	
		Ordering physician:		
		Attending physician:		
		Comment:		

P / PQ:	115 ms / 162 ms
QRS:	96 ms
QT / QTc / QTd:	388 ms / 416 ms / -
P/QRS/T axis:	80° / 87° / 82°
Heartrate:	76 bpm

Beats

Total:	379
Normal:	379
Other:	0

RR

Minimum	590 ms
Maximum	905 ms
Mean:	790 ms
SD:	32 ms

R-R Trend

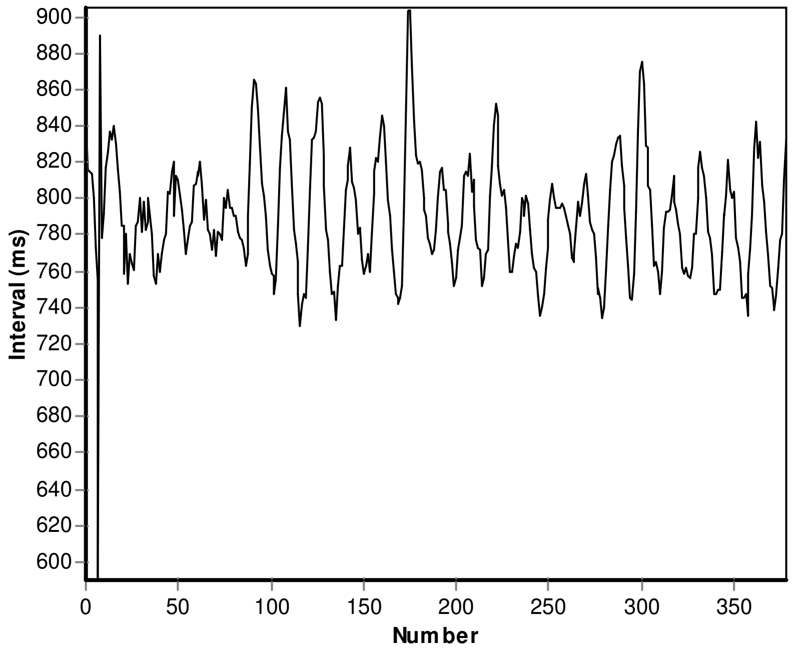

R-R Histogram

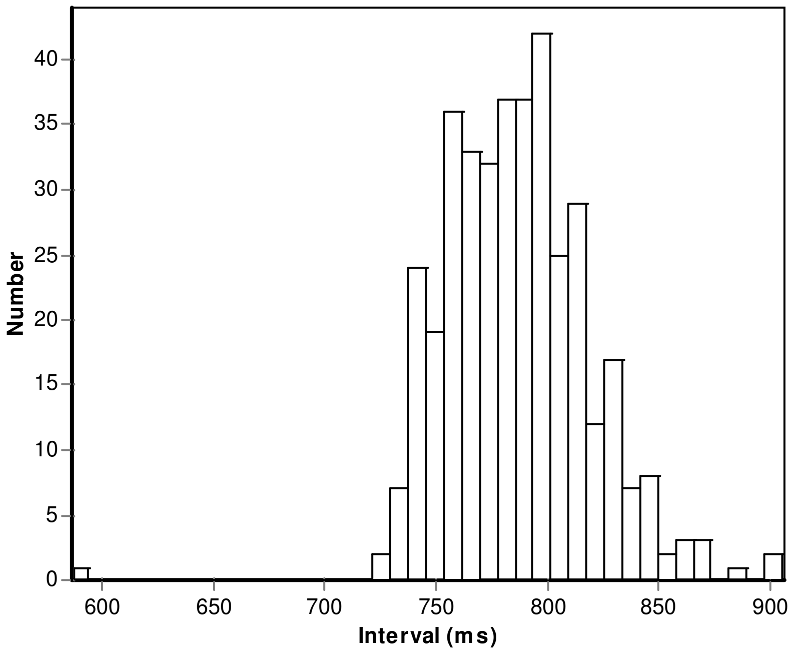

Cardio Control NV. User: Anton

Heart Rate Variability: Time Domain Analysis

**Name:**	017, 017	**Birthdate:**	17/11/1971
**Number:**	017	**Recorded:**	05/05/2018 12:40:27
**Gender:**	Male		

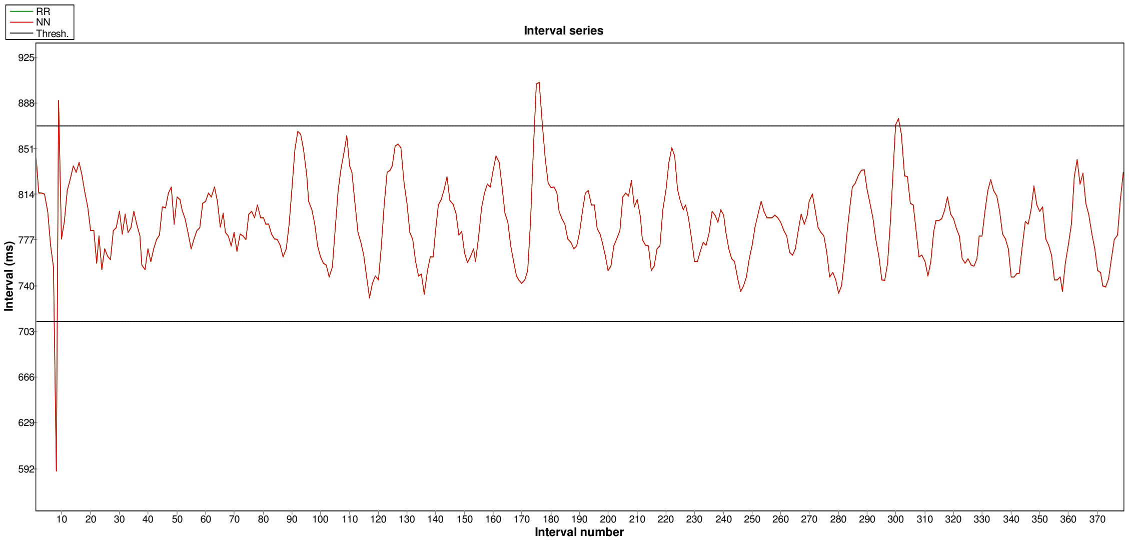

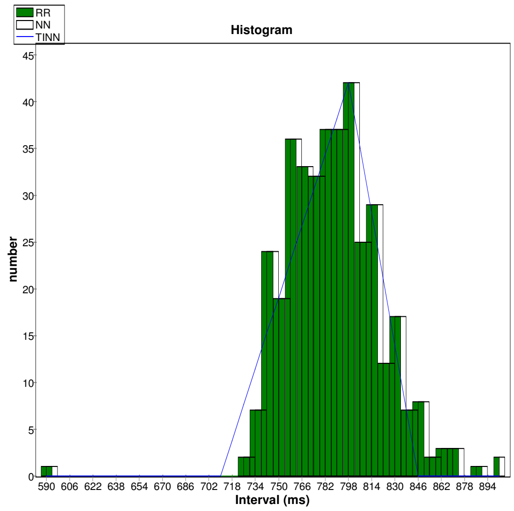

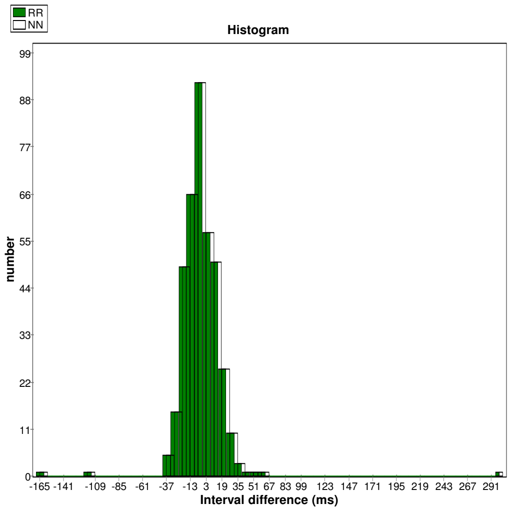

Binsize (ms) = 8

**HRV parameters**	**NN**	**RR**
SDNN (ms)	32	32
Triangular Interpolation (ms)	136	136
Triangular Index	9.02	9.02

**HRV parameters**	**NN**	**RR**
SDSD (ms)	24	24
RMSSD (ms)	24	24
NN50	5	5
NN50(1)	2	2
NN50(2)	3	3
pNN50	0.01	0.01
pNN50(1)	0.01	0.01
pNN50(2)	0.01	0.01
Logarithmic Index	0.59	0.59
SD(Logarithmic Index)	0.08	0.08

**Interval statistics**	**NN**	**RR**
Number	379	379
Minimum (ms)	590	590
Maximum (ms)	905	905
Range (ms)	315	315
Avg (ms)	790	790
SD (ms)	32	32
AvgDev (ms)	25	25
p5 (ms)	745	745
p50 (ms)	788	788
p95 (ms)	848	847
Skewness	-0.07	-0.07
Kurtosis	6.64	6.64

**Interval statistics**	**NN**	**RR**
Number	378	378
Minimum (ms)	-165	-165
Maximum (ms)	300	300
Range (ms)	465	465
Avg (ms)	-0	-0
SD (ms)	24	24
AvgDev (ms)	13	13
p5 (ms)	-24	-24
p50 (ms)	-1	-1
p95 (ms)	26	26
Skewness	4.30	4.30
Kurtosis	75.79	75.88

Heart Rate Variability: Frequency Domain Analysis

**Name:**	017, 017	**Birthdate:**	17/11/1971
**Number:**	017	**Recorded:**	05/05/2018 12:40:27
**Gender:**	Male		

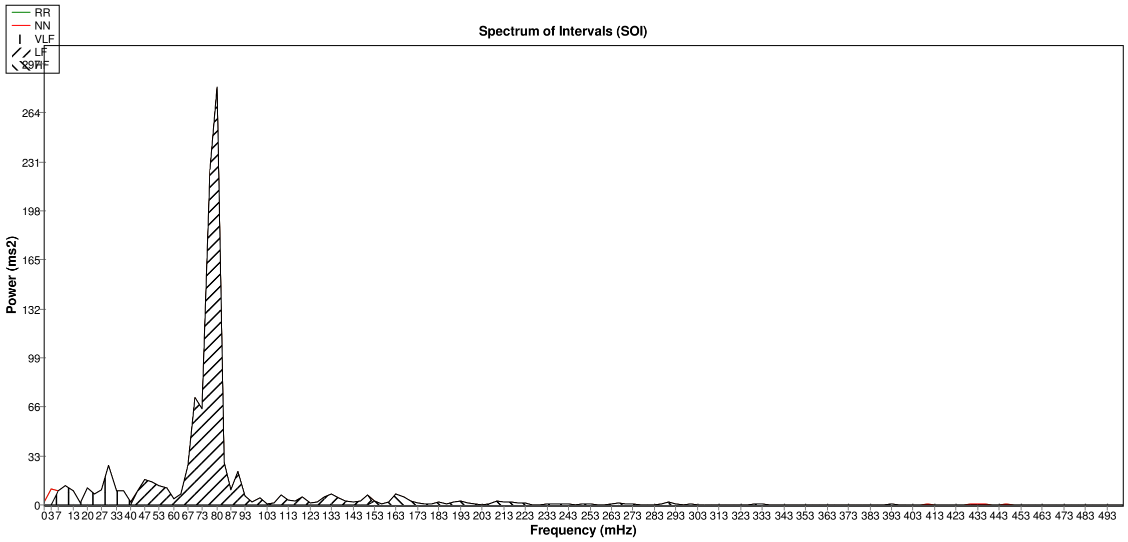

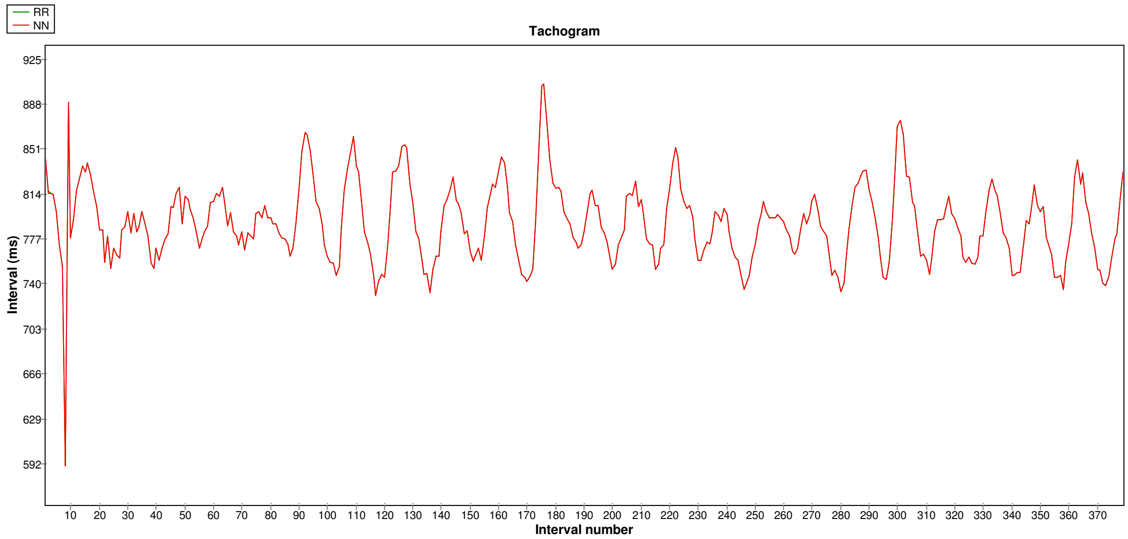

**HRV parameters**	**NN**	**RR**
TP (ms2)	1051	1051
VLF (ms2)	111	111
LF (ms2)	880	880
HF (ms2)	60	60
LF/HF	14.60	14.60
LF normalized	93.59	93.59
HF normalized	6.41	6.41
VLF peak (mHz)	30	30
LF peak (mHz)	80	80
HF peak (mHz)	163	163

**HRV spectral settings**	
Spectrum of Intervals (SOI)	
Frequency resolution (mHz)	3
VLF lower boundary (mHz)	3
VLF upper boundary (mHz)	40
LF upper boundary (mHz)	150
HF upper boundary (mHz)	400
Smoothing factor	1
Tapering	Hann
Fourier transform	DFT
Sample frequency (Hz)	1.27
Interval correction	Annotation
Interval threshold (%)	10
