# Supplementary material for: The effects of a 3-day mountain bike cycling race on the autonomic nervous system (ANS) and heart rate variability in amateur cyclists: a prospective quantitative research design
Source: BMC Sports Sci Med Rehabil. 2023 Jan 2;15:2. doi: 10.1186/s13102-022-00614-y (PMC9808932; doi:10.1186/s13102-022-00614-y)
Supplement: Supplementary file 1 — Additional file 1. Individual data of Participants. [file 13102_2022_614_MOESM1_ESM.zip › Individual data of Participants/HRV Data/016/ECG_016_20180506090731_.PDF]

Anton Swart Biokinetic Rehabilitation Practice

Name: 017 017
Number: 017
Gender: Male
Birthdate: 17/11/1971 46 years

P / PQ: 102 ms / 162 ms
QRS: 100 ms
QT / QTc / QTd: 410 ms / 431 ms / -
P/QRS/T axis: 55° / 88° / 82°
Heartrate: 72 bpm

Recorded: 06/05/2018 09:07:31
Recorded by: Mr. Anton Swart
Referring physician:
Ordering physician:
Attending physician:
Location: Anton Swart Biokinetic Rehabilitation Practi
Comment:

UNCONFIRMED INTERPRETATION - MD SHOULD REVIEW

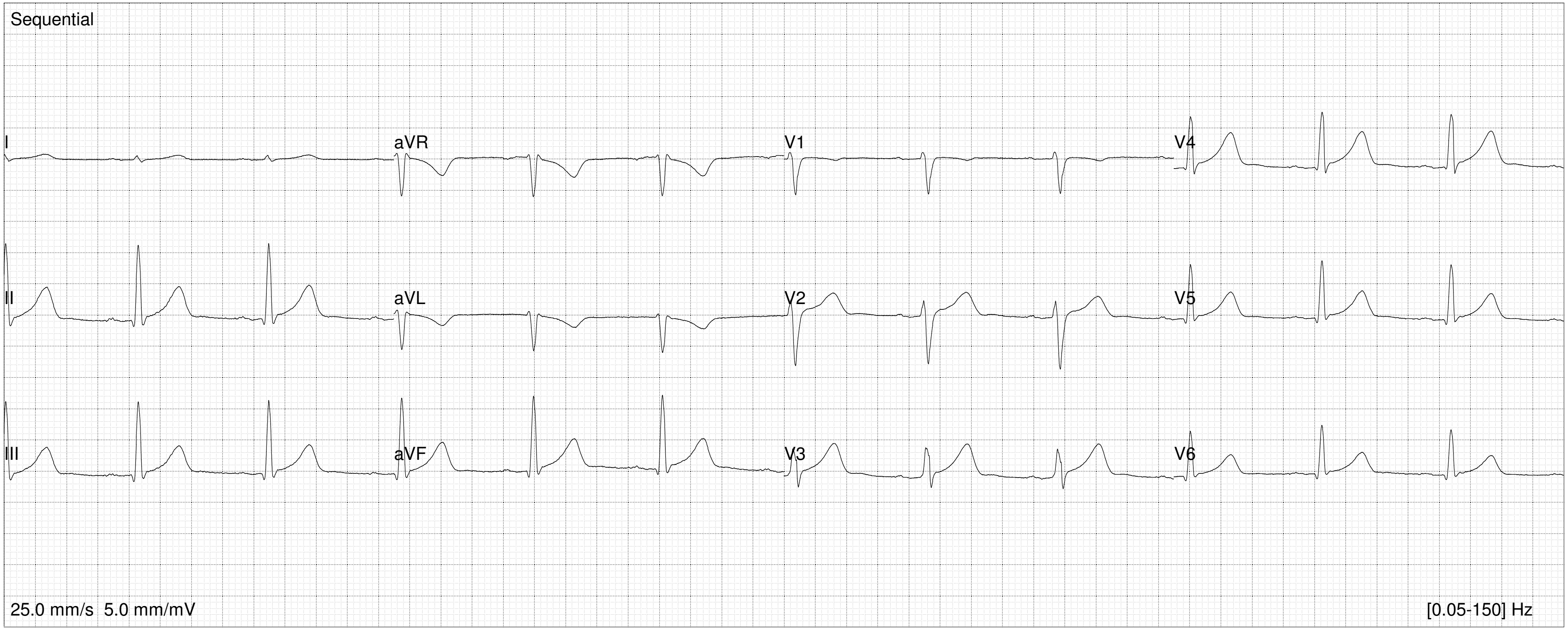

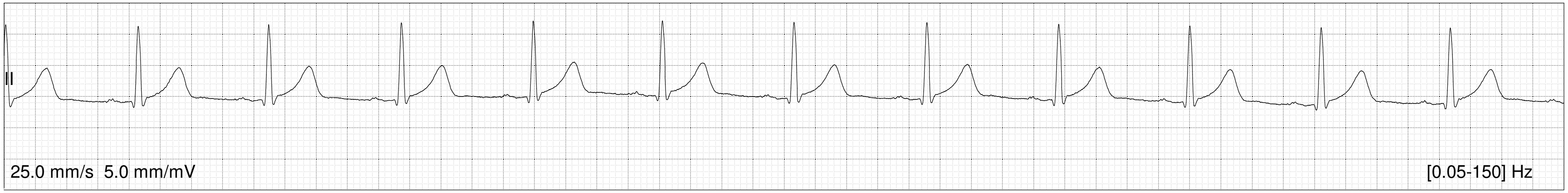

Anton Swart Biokinetic Rehabilitation Practice

Name:	017 017	Recorded:	06/05/2018 09:07:31	UNCONFIRMED INTERPRETATION - MD SHOULD REVIEW
Number:	017	Recorded by:	Mr. Anton Swart	
Gender:	Male	Referring physician:		
Birthdate:	17/11/1971 46 years	Location:	Anton Swart Biokinetic Rehabilitation Practice	
		Ordering physician:		
		Attending physician:		
		Comment:		

P / PQ:	102 ms / 162 ms
QRS:	100 ms
QT / QTc / QTd:	410 ms / 431 ms / -
P/QRS/T axis:	55° / 88° / 82°
Heartrate:	72 bpm

Beats

Total:	357
Normal:	357
Other:	0

RR

Minimum	803 ms
Maximum	877 ms
Mean:	836 ms
SD:	15 ms

R-R Trend

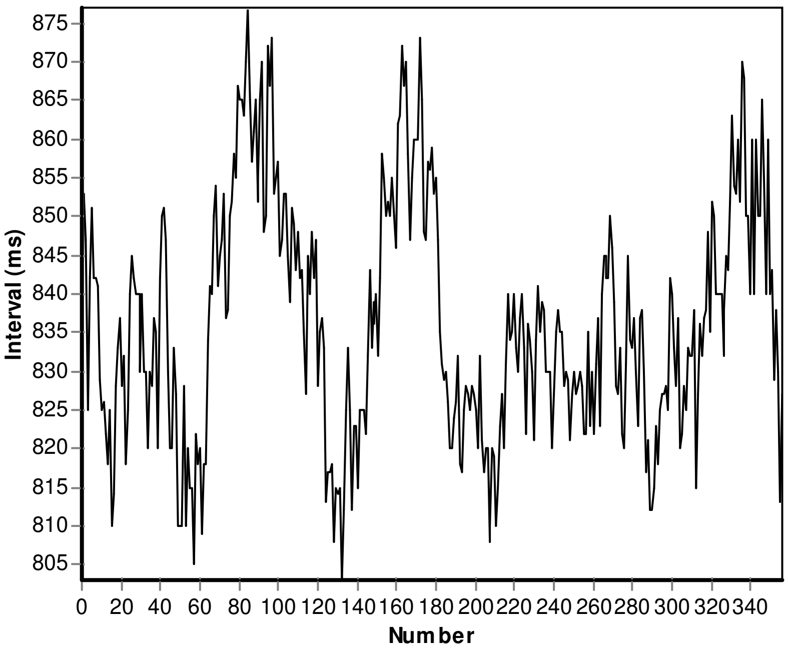

R-R Histogram

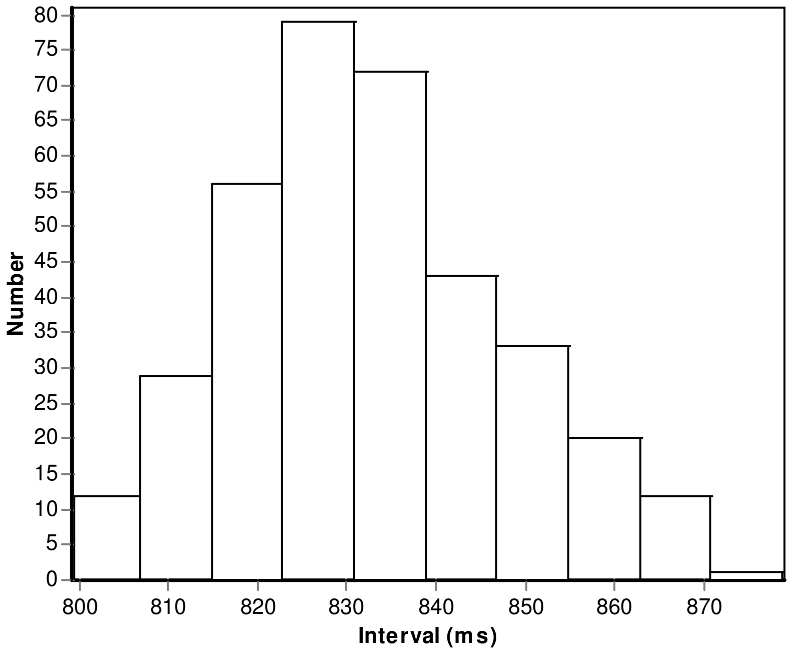

Cardio Control NV. User: Anton

Heart Rate Variability: Time Domain Analysis

Name: 017, 017 **Birthdate:** 17/11/1971
Number: 017 **Recorded:** 06/05/2018 09:07:31
Gender: Male

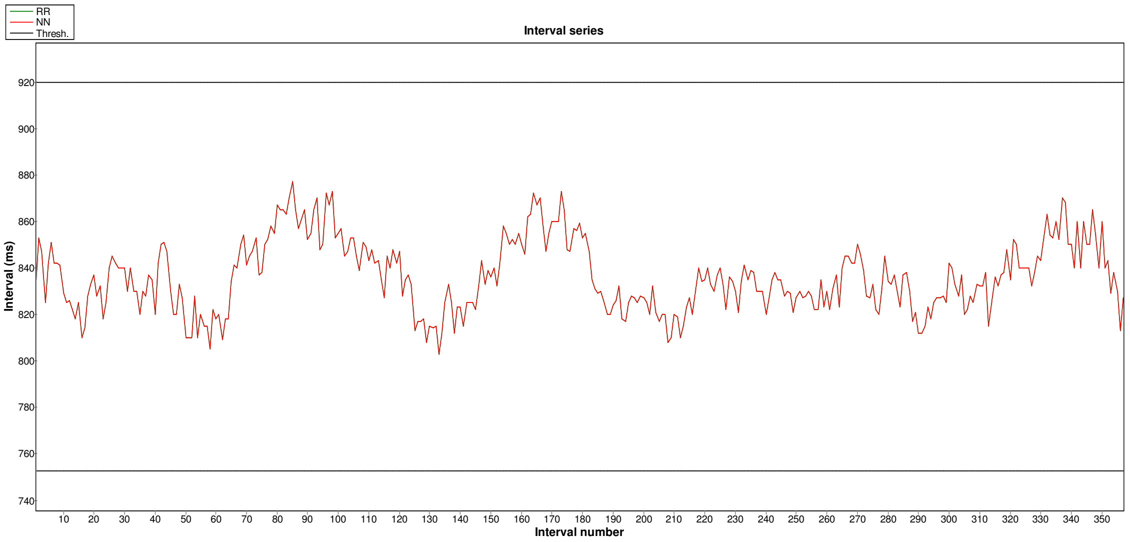

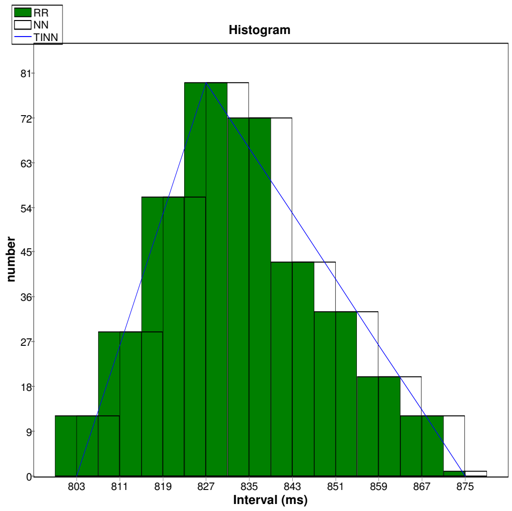

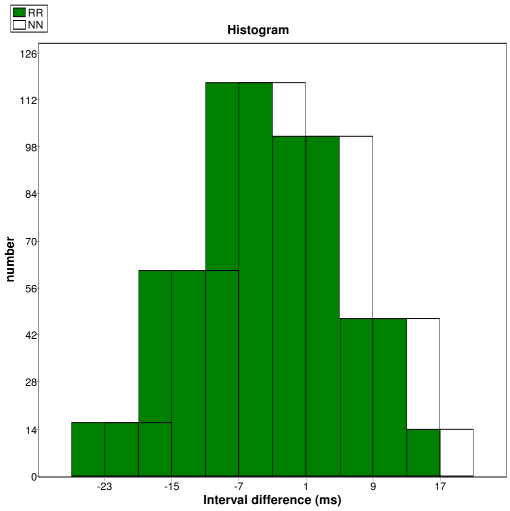

Binsize (ms) = 8

**HRV parameters**	**NN**	**RR**
SDNN (ms)	15	15
Triangular Interpolation (ms)	72	72
Triangular Index	4.52	4.52

**HRV parameters**	**NN**	**RR**
SDSD (ms)	9	9
RMSSD (ms)	9	9
NN50	0	0
NN50(1)	0	0
NN50(2)	0	0
pNN50	0.00	0.00
pNN50(1)	0.00	0.00
pNN50(2)	0.00	0.00
Logarithmic Index	0.92	0.92
SD(Logarithmic Index)	0.19	0.19

**Interval statistics**	**NN**	**RR**
Number	357	357
Minimum (ms)	803	803
Maximum (ms)	877	877
Range (ms)	74	74
Avg (ms)	836	836
SD (ms)	15	15
AvgDev (ms)	12	12
p5 (ms)	813	813
p50 (ms)	835	835
p95 (ms)	865	865
Skewness	0.36	0.36
Kurtosis	2.56	2.56

**Interval statistics**	**NN**	**RR**
Number	356	356
Minimum (ms)	-23	-23
Maximum (ms)	22	22
Range (ms)	45	45
Avg (ms)	-0	-0
SD (ms)	9	9
AvgDev (ms)	7	7
p5 (ms)	-15	-15
p50 (ms)	0	0
p95 (ms)	16	16
Skewness	-0.04	-0.04
Kurtosis	2.79	2.79

Cardio Control NV. User: Anton

Heart Rate Variability: Frequency Domain Analysis

**Name:**	017, 017	**Birthdate:**	17/11/1971
**Number:**	017	**Recorded:**	06/05/2018 09:07:31
**Gender:**	Male		

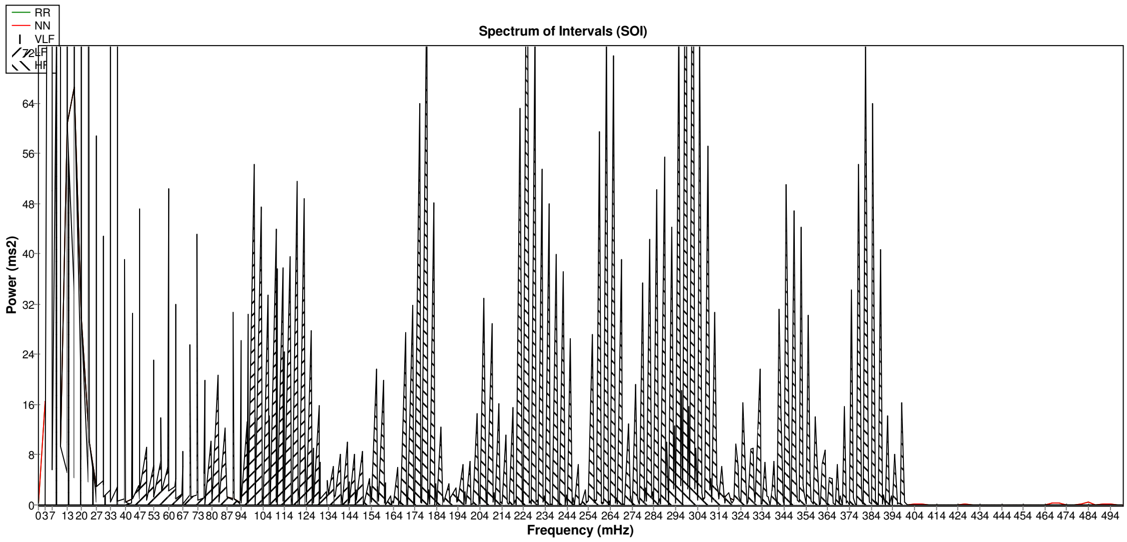

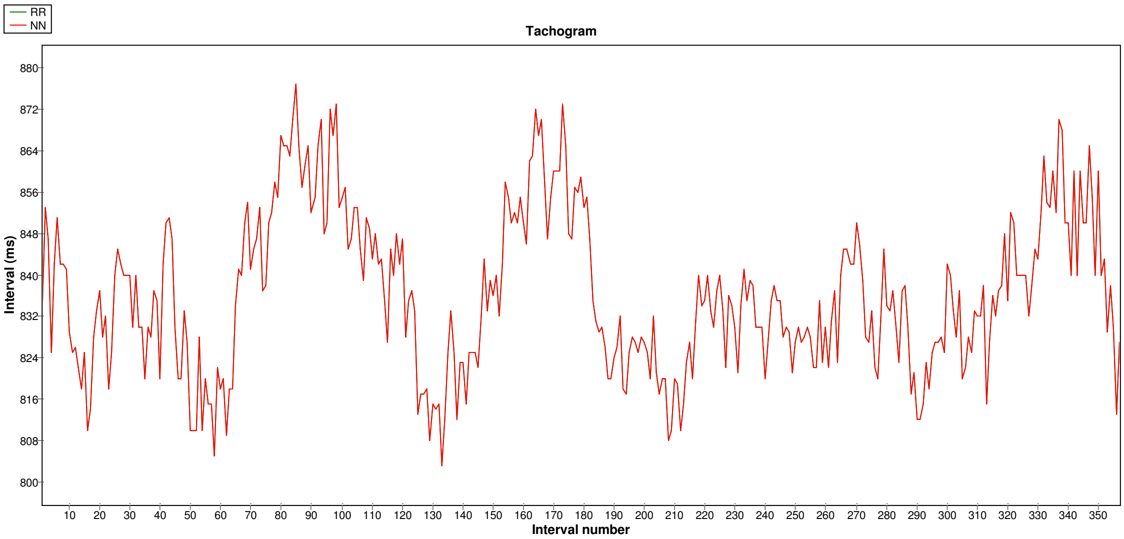

**HRV parameters**	**NN**	**RR**
TP (ms2)	232	232
VLF (ms2)	188	188
LF (ms2)	24	24
HF (ms2)	20	20
LF/HF	1.21	1.21
LF normalized	54.77	54.77
HF normalized	45.23	45.23
VLF peak (mHz)	17	17
LF peak (mHz)	47	47
HF peak (mHz)	317	317

**HRV spectral settings**	
Spectrum of Intervals (SOI)	
Frequency resolution (mHz)	3
VLF lower boundary (mHz)	3
VLF upper boundary (mHz)	40
LF upper boundary (mHz)	150
HF upper boundary (mHz)	400
Smoothing factor	1
Tapering	Hann
Fourier transform	DFT
Sample frequency (Hz)	1.20
Interval correction	Annotation
Interval threshold (%)	10
